# Supplementary material for: Collider bias correction for multiple covariates in GWAS using robust multivariable Mendelian randomization
Source: PLoS Genet. 2024 Apr 22;20(4):e1011246. doi: 10.1371/journal.pgen.1011246 (PMC11065275; doi:10.1371/journal.pgen.1011246)
Supplement: S1 Text — (PDF) [file pgen.1011246.s001.pdf]

Supplementary materials: Collider bias correction for multiple covariates in GWAS using robust multivariable Mendelian randomization

Peiyao Wang[1], Zhaotong Lin[1,2], Haoran Xue[1,3], Wei Pan[1]
[1] Division of Biostatistics, School of Public Health,
University of Minnesota, Minneapolis, MN, USA;
[2] Department of Statistics,
Florida State University, Tallahassee, FL, USA;
[3] Department of Biostatistics,
City University of Hong Kong, Hong Kong, China.

Contents

A Derivation of the bias term **1**

B Theory **6**
B.1 Assumptions 6
B.2 Proof of Theorem 1 7
B.3 Estimation of the covariance matrix 10

C Simulation without pleiotropy **13**
C.1 Simulation set up 13
C.2 Simulation results 14

D Simulation without collider bias **16**

E Comparison of UVMR and MVMR **19**
E.1 Simulation 19
E.2 Real GWAS data 21

F Simulation with (weakly) correlated SNPs and pleiotropy **26**
F.1 Simulation set up 26
F.2 30% invalid IVs 27
F.2.1 Tables of empirical type-I error rate and power 27
F.2.2 Estimates of slope vector 29
F.2.3 Effect estimates, empirical type-I error rate and power of representative SNPs 31
F.2.4 Figures of point estimates 33
F.3 50% invalid IVs 41
F.3.1 Tables of empirical type-I error rate and power 41
F.3.2 Estimates of slope vector 42
F.3.3 Effect estimates, empirical type-I error rate and power of representative SNPs 44
F.3.4 Figures of point estimates 46

G Real GWAS data **54**
G.1 GWAS of WHR adjusted for BMI . . . 54
G.1.1 Tables of significant SNPs and loci . . . 54
G.1.2 Comparison of SNP effect estimates before and after apply different bias-correction methods . . . 55
G.1.3 Comparison of Manhattan plots before and after apply different bias-correction methods . . . 58
G.1.4 Comparison of QQ plots before and after apply different bias-correction methods . . . 61
G.2 GWAS of BMI adjusted for metabolomic PCs . . . 62
G.2.1 Results after removing genetic components . . . 62
G.2.2 Tables of significant loci . . . 68
G.2.3 Comparison of SNP effect estimates before and after apply different bias-correction methods on M_1 . . . 70
G.2.4 Comparison of SNP effect estimates before and after apply different bias-correction methods on M_1, all 20 metabolomic PCs are used in M_1 . . . 81
G.2.5 Comparison of SNP effect estimates before and after apply different bias-correction methods on M_2 . . . 92
G.2.6 Comparison of SNP effect estimates befor and after apply different bias-correction methods on M_2, all 20 metabolomic PCs are used in M_2 . . . 103
G.2.7 Comparison of Manhattan plots before and after apply different bias-correction methods on M_1 . . . 114
G.2.8 Comparison of Manhattan plots before and after apply different bias-correction methods on M_1, all 20 metabolomic PCs are used in M_1 . . . 125
G.2.9 Comparison of Manhattan plots before and after apply different bias-correction methods on M_2 . . . 136
G.2.10 Comparison of Manhattan plots before and after apply different bias-correction methods on M_2, all 20 metabolomic PCs are used in M_2 . . . 147
G.2.11 Comparison of QQ plots before and after apply different bias-correction methods on M_1 . . . 158
G.2.12 Comparison of QQ plots before and after apply different bias-correction methods on M_1, all 20 metabolomic PCs are used in M_1 . . . 164
G.2.13 Comparison of QQ plots before and after apply different bias-correction methods on M_2 . . . 170
G.2.14 Comparison of QQ plots before and after apply different bias-correction methods on M_2, all 20 metabolomic PCs are used in M_2 . . . 176

A Derivation of the bias term

We give the derivation of the bias term, assuming the causal relationship below.

$$\boldsymbol{H} = \boldsymbol{\beta}_{G_i \boldsymbol{H}} G_i + \mathbf{B}_{\boldsymbol{VH}} \boldsymbol{V} + \boldsymbol{\beta}_{U\boldsymbol{H}} U + E_{\boldsymbol{H}}, \tag{1}$$

$$Y = \beta_{G_i Y} G_i + \boldsymbol{\beta}_{\boldsymbol{X}Y}^T \boldsymbol{X} + \beta_{UY} U + E_Y, \tag{2}$$

$$= \beta_{G_i Y} G_i + \boldsymbol{\beta}_{\boldsymbol{J}Y}^T \boldsymbol{J} + \boldsymbol{\beta}_{\boldsymbol{H}Y}^T \boldsymbol{H} + \beta_{UY} U + E_Y. \tag{3}$$

To obtain the bias term, we start from the regression equation:

$$Y = \beta^C_{G_iY} G_i + \boldsymbol{\beta}'^T_{\boldsymbol{J}Y} \boldsymbol{J} + \boldsymbol{\beta}'^T_{\boldsymbol{H}Y} \boldsymbol{H} + \epsilon. \tag{4}$$

We assume that $\boldsymbol{V}$ is uncorrelated with U and the SNPs, and that $\boldsymbol{J}$, G_i, U, $E_{\boldsymbol{H}}$, and E_Y are pairwise uncorrelated. Without losing generality, we assume U, $\boldsymbol{X}$, Y, G_i, $E_{\boldsymbol{H}}$, and E_Y have mean 0, and an asymptotic lease square approach [1] gives:

$$\begin{aligned}
\begin{pmatrix} \beta^C_{G_iY} \\ \boldsymbol{\beta}'_{\boldsymbol{X}Y} \end{pmatrix} &= \begin{pmatrix} Var(G_i) & Cov(G_i, \boldsymbol{X}) \\ Cov(G_i, \boldsymbol{X})^T & Cov(\boldsymbol{X}) \end{pmatrix}^{-1} \begin{pmatrix} Cov(G_i, Y) \\ Cov(\boldsymbol{X}, Y) \end{pmatrix} \\
&= \begin{pmatrix} \frac{1}{Var(G_i)} + \frac{Cov(G_i,\boldsymbol{X})\mathbf{M}Cov(G_i,\boldsymbol{X})^T}{Var^2(G_i)} & -\frac{Cov(G_i,\boldsymbol{X})\mathbf{M}}{Var(G_i)} \\ -\frac{\mathbf{M}Cov(G_i,\boldsymbol{X})^T}{Var(G_i)} & \mathbf{M} \end{pmatrix} \begin{pmatrix} Cov(G_i, Y) \\ Cov(\boldsymbol{X}, Y) \end{pmatrix},
\end{aligned}$$

where

$$\mathbf{M} = \left(Cov(\boldsymbol{X}) - \frac{Cov(G_i,\boldsymbol{X})^T Cov(G_i,\boldsymbol{X})}{Var(G_i)} \right)^{-1}.$$

Partition $\boldsymbol{X}$ into $\boldsymbol{J}$ and $\boldsymbol{H}$, using $Cov(\boldsymbol{J}, G_i) = \mathbf{0}$, we have

$$Cov(G_i, \boldsymbol{X}) = \begin{pmatrix} \mathbf{0}^T & Cov(G_i, \boldsymbol{H}) \end{pmatrix},$$

$$Cov(\boldsymbol{X}) = \begin{pmatrix} Cov(\boldsymbol{J}) & Cov(\boldsymbol{J}, \boldsymbol{H}) \\ Cov(\boldsymbol{J}, \boldsymbol{H})^T & Cov(\boldsymbol{H}) \end{pmatrix},$$

$$\mathbf{M} = \begin{pmatrix} Cov(\boldsymbol{J}) & Cov(\boldsymbol{J}, \boldsymbol{H}) \\ Cov(\boldsymbol{J}, \boldsymbol{H})^T & Cov(\boldsymbol{H}) - \frac{Cov(G_i,\boldsymbol{H})^T Cov(G_i,\boldsymbol{H})}{Var(G_i)} \end{pmatrix}^{-1} = \begin{pmatrix} \mathbf{M}_{11} & \mathbf{M}_{21}^T \\ \mathbf{M}_{21} & \mathbf{M}_{22} \end{pmatrix}.$$

Where

$$\mathbf{M}_{11} = Cov(\boldsymbol{J})^{-1} + Cov(\boldsymbol{J})^{-1} Cov(\boldsymbol{J}, \boldsymbol{H}) \mathbf{M}_{22} Cov(\boldsymbol{J}, \boldsymbol{H})^T Cov(\boldsymbol{J})^{-1},$$

$$\mathbf{M}_{21} = -\mathbf{M}_{22} Cov(\boldsymbol{J}, \boldsymbol{H})^T Cov(\boldsymbol{J})^{-1},$$

$$\mathbf{M}_{22} = \left(Cov(\boldsymbol{H}) - \frac{Cov(G_i,\boldsymbol{H})^T Cov(G_i,\boldsymbol{H})}{Var(G_i)} - Cov(\boldsymbol{J}, \boldsymbol{H})^T Cov(\boldsymbol{J})^{-1} Cov(\boldsymbol{J}, \boldsymbol{H}) \right)^{-1}. \tag{5}$$

Plugging $Cov(G_i, \boldsymbol{X})$ and $\mathbf{M}$ into the lease square approach, we have

$$\begin{pmatrix} \beta^C_{G_iY} \\ \boldsymbol{\beta}'_{\boldsymbol{J}Y} \\ \boldsymbol{\beta}'_{\boldsymbol{H}Y} \end{pmatrix} = \begin{pmatrix} \frac{1}{Var(G_i)} + \frac{Cov(G_i,\boldsymbol{H})\mathbf{M}_{22}Cov(G_i,\boldsymbol{H})^T}{Var^2(G_i)} & -\frac{Cov(G_i,\boldsymbol{H})\mathbf{M}_{21}}{Var(G_i)} & -\frac{Cov(G_i,\boldsymbol{H})\mathbf{M}_{22}}{Var(G_i)} \\ -\frac{\mathbf{M}_{21}^T Cov(G_i,\boldsymbol{H})^T}{Var(G_i)} & \mathbf{M}_{11} & \mathbf{M}_{21}^T \\ -\frac{\mathbf{M}_{22}Cov(G_i,\boldsymbol{H})^T}{Var(G_i)} & \mathbf{M}_{21} & \mathbf{M}_{22} \end{pmatrix} \begin{pmatrix} Cov(G_i, Y) \\ Cov(\boldsymbol{J}, Y) \\ Cov(\boldsymbol{H}, Y) \end{pmatrix}.$$

Hence,

$$\beta^C_{G_iY} = \frac{Cov(G_i, Y)}{Var(G_i)} + \frac{Cov(G_i, \boldsymbol{H})\mathbf{M}_{22}Cov(G_i, \boldsymbol{H})^T Cov(G_i, Y)}{Var^2(G_i)} \quad (6)$$

$$-\frac{Cov(G_i, \boldsymbol{H})\mathbf{M}_{21}Cov(\boldsymbol{J}, Y)}{Var(G_i)} - \frac{Cov(G_i, \boldsymbol{H})\mathbf{M}_{22}Cov(\boldsymbol{H}, Y)}{Var(G_i)}. \quad (7)$$

Also, using the linear relationship between variables, we have

$$Cov(G_i, Y) = \beta_{G_iY}Var(G_i) + Cov(G_i, \boldsymbol{H})\boldsymbol{\beta}_{\boldsymbol{H}Y},$$

$$Cov(\boldsymbol{J}, Y) = Cov(\boldsymbol{J})\boldsymbol{\beta}_{\boldsymbol{J}Y} + Cov(\boldsymbol{J}, \boldsymbol{H})\boldsymbol{\beta}_{\boldsymbol{H}Y},$$

$$Cov(\boldsymbol{H}, Y) = \beta_{G_iY}Cov(\boldsymbol{H}, G_i) + \beta_{UY}Cov(\boldsymbol{H}, U) + Cov(\boldsymbol{H})\boldsymbol{\beta}_{\boldsymbol{H}Y} + Cov(\boldsymbol{H}, \boldsymbol{J})\boldsymbol{\beta}_{\boldsymbol{J}Y}.$$

Plugging into 7, we have

$$\begin{aligned}\beta^C_{G_iY} = \beta_{G_iY} &+ \frac{Cov(G_i, \boldsymbol{H})\boldsymbol{\beta}_{\boldsymbol{H}Y}}{Var(G_i)} + \frac{Cov(G_i, \boldsymbol{H})\mathbf{M}_{22}Cov(G_i, \boldsymbol{H})^T\beta_{G_iY}}{Var(G_i)} \\ &+ \frac{Cov(G_i, \boldsymbol{H})\mathbf{M}_{22}Cov(G_i, \boldsymbol{H})^T Cov(G_i, \boldsymbol{H})\boldsymbol{\beta}_{\boldsymbol{H}Y}}{Var(G_i)} - \frac{Cov(G_i, \boldsymbol{H})\mathbf{M}_{21}Cov(\boldsymbol{J})\boldsymbol{\beta}_{\boldsymbol{J}Y}}{Var(G_i)} \\ &- \frac{Cov(G_i, \boldsymbol{H})\mathbf{M}_{21}Cov(\boldsymbol{J}, \boldsymbol{H})\boldsymbol{\beta}_{\boldsymbol{H}Y}}{Var(G_i)} - \frac{Cov(G_i, \boldsymbol{H})\mathbf{M}_{22}Cov(\boldsymbol{H}, G_i)\beta_{G_iY}}{Var(G_i)} \\ &- \frac{Cov(G_i, \boldsymbol{H})\mathbf{M}_{22}Cov(\boldsymbol{H}, U)\beta_{UY}}{Var(G_i)} - \frac{Cov(G_i, \boldsymbol{H})\mathbf{M}_{22}Cov(\boldsymbol{H})\boldsymbol{\beta}_{\boldsymbol{H}Y}}{Var(G_i)} \\ &- \frac{Cov(G_i, \boldsymbol{H})\mathbf{M}_{22}Cov(\boldsymbol{H}, \boldsymbol{J})\boldsymbol{\beta}_{\boldsymbol{J}Y}}{Var(G_i)}.\end{aligned}$$

Now, using $\mathbf{M}_{21} = -\mathbf{M}_{22}Cov(\boldsymbol{J}, \boldsymbol{H})^T Cov(\boldsymbol{J})^{-1}$, the expression of $\beta^C_{G_iY}$ becomes

$$\begin{aligned}\beta^C_{G_iY} = \beta_{G_iY} &+ \frac{Cov(G_i,\boldsymbol{H})\boldsymbol{\beta_{HY}}}{Var(G_i)} + \frac{Cov(G_i,\boldsymbol{H})\mathbf{M}_{22}Cov(G_i,\boldsymbol{H})^TCov(G_i,\boldsymbol{H})\boldsymbol{\beta_{HY}}}{Var(G_i)} \\ &+ \frac{Cov(G_i,\boldsymbol{H})\mathbf{M}_{22}Cov(\boldsymbol{J},\boldsymbol{H})^TCov(\boldsymbol{J})^{-1}Cov(\boldsymbol{J},\boldsymbol{H})\boldsymbol{\beta_{HY}}}{Var(G_i)} \\ &- \frac{Cov(G_i,\boldsymbol{H})\mathbf{M}_{22}Cov(\boldsymbol{H})\boldsymbol{\beta_{HY}}}{Var(G_i)} \\ &- \frac{Cov(G_i,\boldsymbol{H})\mathbf{M}_{22}Cov(\boldsymbol{H},U)\beta_{UY}}{Var(G_i)}.\end{aligned}$$

After reorganizing the terms, $\beta^C_{G_iY}$ is reduced to

$$\begin{aligned}\beta^C_{G_iY} = \beta_{G_iY} &+ \frac{Cov(G_i,\boldsymbol{H})}{Var(G_i)}\boldsymbol{\beta_{HY}} - \frac{Cov(G_i,\boldsymbol{H})\mathbf{M}_{22}Cov(\boldsymbol{H},U)\beta_{UY}}{Var(G_i)} \\ &+ \frac{Cov(G_i,\boldsymbol{H})}{Var(G_i)}\mathbf{M}_{22}\left[\frac{Cov(G_i,\boldsymbol{H})^TCov(G_i,\boldsymbol{H})}{Var(G_i)} + Cov(\boldsymbol{J},\boldsymbol{H})^TCov(\boldsymbol{J})^{-1}Cov(\boldsymbol{J},\boldsymbol{H}) \right. \\ &\left. - Cov(\boldsymbol{H})\right]\boldsymbol{\beta_{HY}}.\end{aligned}$$

Using Equation (5), we have

$$\begin{aligned}\beta^C_{G_iY} =& \beta_{G_iY} + \frac{Cov(G_i,\boldsymbol{H})}{Var(G_i)}(\boldsymbol{I} - \mathbf{M}_{22}\mathbf{M}_{22}^{-1})\boldsymbol{\beta_{HY}} - \frac{Cov(G_i,\boldsymbol{H})\mathbf{M}_{22}Cov(\boldsymbol{H},U)\beta_{UY}}{Var(G_i)} \\ =& \beta_{G_iY} - \frac{Cov(G_i,\boldsymbol{H})\beta_{UY}}{Var(G_i)}\mathbf{M}_{22}Cov(\boldsymbol{H},U).\end{aligned}$$

$\boldsymbol{I}$ is the identity matrix.

Using Equations (1) and (3), we have the covariance matrices

$$Cov(\boldsymbol{H}) = \boldsymbol{\beta}_{G_i\boldsymbol{H}}\boldsymbol{\beta}^T_{G_i\boldsymbol{H}}Var(G_i) + \mathbf{B}_{\boldsymbol{VH}}Cov(\boldsymbol{V})\mathbf{B}^T_{\boldsymbol{VH}} + \boldsymbol{\beta}_{U\boldsymbol{H}}\boldsymbol{\beta}^T_{U\boldsymbol{H}}Var(U) + Cov(E_{\boldsymbol{H}}),$$

$$Cov(\boldsymbol{J},\boldsymbol{H}) = Cov(\boldsymbol{J},\boldsymbol{V})\boldsymbol{\beta}^T_{\boldsymbol{VH}},$$

$$Cov(G_i,\boldsymbol{H}) = \boldsymbol{\beta}^T_{G_i\boldsymbol{H}}Var(G_i),$$

$$Cov(\boldsymbol{H},U) = \boldsymbol{\beta}_{U\boldsymbol{H}}Var(U).$$

Plugging into (5), the terms related to G_i are canceled out, and we have the expression of $\mathbf{M}_{22}$:

$$\begin{aligned}\mathbf{M}_{22} =&\Big\{\boldsymbol{\beta}_{U\boldsymbol{H}}\boldsymbol{\beta}_{U\boldsymbol{H}}^T Var(U) + Cov(\boldsymbol{E_H}) + \mathbf{B}_{\boldsymbol{VH}}Cov(\boldsymbol{V})\mathbf{B}_{\boldsymbol{VH}}^T \\ &- \mathbf{B}_{\boldsymbol{VH}}Cov(\boldsymbol{J},\boldsymbol{V})^T Cov(\boldsymbol{J})^{-1}Cov(\boldsymbol{J},\boldsymbol{V})\mathbf{B}_{\boldsymbol{VH}}^T\Big\}^{-1}.\end{aligned} \tag{8}$$

Finally, we can write $\beta_{G_iY}^C$ as

$$\beta_{G_iY}^C = \beta_{G_iY} - \frac{Cov(G_i,\boldsymbol{H})\beta_{UY}}{Var(G_i)}\mathbf{M}_{22}Cov(\boldsymbol{H},U) = \beta_{G_iY} + \boldsymbol{b}^T\boldsymbol{\beta}_{G_i\boldsymbol{H}}. \tag{9}$$

Where

$$\boldsymbol{b} = -\beta_{UY}Var(U)\mathbf{M}_{22}\boldsymbol{\beta}_{U\boldsymbol{H}}.$$

B Theory

Let $\mathcal{G}$ denote the collection of SNPs in the GWAS of Y, $\mathcal{G} = \{G_1, \cdots, G_m\}$, among which $Z_1, \cdots, Z_l$ are selected as independent IVs. Following the notations in the main paper, we use subscripts i and j for G and Z respectively. N denotes the minimum sample size of all GWAS datasets used for analysis. We also use a superscript 0 to denote true parameter values.

B.1 Assumptions

To prove Theorem 1, we need the Assumptions 1-3 stated below.

Assumption 1. *For each $G_i \in \mathcal{G}$, as $N \to +\infty$, the corresponding GWAS summary statistic*

$$\hat{\boldsymbol{\beta}}_{G_i} = (\hat{\beta}^C_{G_iY}, \hat{\beta}_{G_iH_1}, \cdots, \hat{\beta}_{G_iH_{p_2}})^T, \quad i = 1, \cdots, m,$$

satisfies:

$$\hat{\boldsymbol{\beta}}_{G_i} \xrightarrow{P} \boldsymbol{\beta}^0_{G_i}$$

and

$$\begin{pmatrix} \hat{\boldsymbol{\beta}}_{G_i} \\ \hat{\boldsymbol{\beta}}_{Z_1} \\ \vdots \\ \hat{\boldsymbol{\beta}}_{Z_l} \end{pmatrix} \xrightarrow{D} N\left\{ \begin{pmatrix} \boldsymbol{\beta}^0_{G_i} \\ \boldsymbol{\beta}^0_{Z_1} \\ \vdots \\ \boldsymbol{\beta}^0_{Z_l} \end{pmatrix}, \boldsymbol{\Psi} \right\},$$

where $\boldsymbol{\beta}^0_{G_i} = (\beta^{C0}_{G_iY}, \beta^0_{G_iH_1}, \cdots, \beta^0_{G_iH_{p_2}})^T$ and $\boldsymbol{\beta}^0_{Z_j} = (\beta^{C0}_{Z_jY}, \beta^0_{Z_jH_1}, \cdots, \beta^0_{Z_jH_{p_2}})^T$ denote the true SNP effects.

The covariance matrix $\boldsymbol{\Psi}$ can be approximated using GWAS output and the LD matrix of SNPs [2]. Following previous literature, we assume $\boldsymbol{\Psi}$ is known or well-estimated. Note that this assumption is reasonable given that GWAS summary data are obtained from marginal regressions.

Assumption 2. *The matrix $\boldsymbol{B}_{\mathcal{V}^*} = [\boldsymbol{\beta}^0_{Z_j\boldsymbol{H}}]_{j\in\mathcal{V}^*} \in \mathbb{R}^{p_2 \times l^0}$ has full column rank l^0, $\mathcal{V}^*$ is the true set of valid IVs. Moreover, the following multivariable plurality condition holds [3]:*

$$|\mathcal{V}^*| > \max_{\mathbf{c}\neq\mathbf{0}, \mathbf{c}\in\mathbb{R}^{p_2}} |\{j : r_j = \mathbf{c}^T\boldsymbol{\beta}^0_{Z_j\boldsymbol{H}}\}|.$$

Assumption 3. *Let $\sigma^{2C}_{Z_jY}$ and $\sigma^2_{Z_jH_k}$ denote the variance of $\hat{\beta}^C_{Z_jY}$ and $\hat{\beta}_{Z_jH_k}$ respectively. There exist two positive constants c_1, c_2 such that $c_1/N < \sigma^{2C}_{Z_jY} < c_2/N$ and $c_1/N < \sigma^2_{Z_jH_k} < c_2/N$ for $j = 1, \cdots, l$, $k = 1, \cdots, p_2$.*

The true parameter $\boldsymbol{b}^0$ is identifiable for MVMR-cML if and only if Assumption 2 holds [3]. Under Assumptions 1-3, MVMR-cML is consistent [3], i.e., as $N \to \infty$, $\hat{\boldsymbol{b}} \xrightarrow{P} \boldsymbol{b}^0$.

B.2 Proof of Theorem 1

We have the following theorem:

Theorem 1. *Under the Assumptions 1-3, as $N \to +\infty$, the bias-corrected estimator $\hat{\beta}_{G_iY} = \hat{\beta}^C_{G_iY} - \hat{\boldsymbol{b}}^T \hat{\boldsymbol{\beta}}_{G_i\boldsymbol{H}}$ is consistent and has an asymptotic normal distribution:*

$$(\hat{\beta}_{G_iY} - \beta^0_{G_iY})/\sigma_{G_iY} \xrightarrow{D} N(0,1), \tag{10}$$

where $\beta^0_{G_iY}$ is the true direct effect of G_i on Y, and $\sigma^2_{G_iY}$ is the variance of $\hat{\beta}_{G_iY}$.

Proof. Since MVMR-cML consistently select the true set of valid IVs [3], we can write down the negative log-likelihood function using all valid IVs $Z_1, \cdots, Z_l$:

$$L = \frac{1}{2}\sum_{j=1}^{l} (\hat{\boldsymbol{\beta}}_{Z_j} - \boldsymbol{\beta}_{Z_j})^T \boldsymbol{\Sigma}_j^{-1} (\hat{\boldsymbol{\beta}}_{Z_j} - \boldsymbol{\beta}_{Z_j}),$$

where $\hat{\boldsymbol{\beta}}_{Z_j} = (\hat{\beta}^C_{Z_jY}, \hat{\beta}_{Z_jH_1}, \cdots, \hat{\beta}_{Z_jH_{p_2}})$ and $\boldsymbol{\beta}_{Z_j} = (\boldsymbol{b}^T \boldsymbol{\beta}_{Z_j\boldsymbol{H}}, \beta_{Z_jH_1}, \cdots, \beta_{Z_jH_{p_2}})$. With

$$\boldsymbol{\theta} = \begin{pmatrix} \boldsymbol{\beta}_{Z_1} \\ \vdots \\ \boldsymbol{\beta}_{Z_l} \\ \boldsymbol{b} \end{pmatrix}, \quad \mathbf{W} = \begin{pmatrix} b_1 & 1 & & \\ \vdots & & \ddots & \\ b_{p_2} & & & 1 \end{pmatrix}, \quad \mathbf{V}_j = \begin{pmatrix} \beta_{Z_jH_1} & 0 & & \\ \vdots & & \ddots & \\ \beta_{Z_jH_{p_2}} & & & 0 \end{pmatrix},$$

the partial derivatives are

$$\phi_j(\boldsymbol{\theta}) = \frac{\partial L}{\partial \boldsymbol{\beta}_{Z_j\boldsymbol{H}}} = \mathbf{W}\boldsymbol{\Sigma}_j^{-1}(\boldsymbol{\beta}_{Z_j} - \hat{\boldsymbol{\beta}}_{Z_j}), \quad j = 1, \cdots, l$$

$$\phi_{l+1}(\boldsymbol{\theta}) = \begin{pmatrix} \frac{\partial L}{\partial b_1} \\ \vdots \\ \frac{\partial L}{\partial b_{p_2}} \end{pmatrix} = \sum_{j=1}^{l} \mathbf{V}_j \boldsymbol{\Sigma}_j (\boldsymbol{\beta}_{Z_j} - \hat{\boldsymbol{\beta}}_{Z_j}).$$

We get the estimated parameters

$$\hat{\boldsymbol{\theta}} = \begin{pmatrix} \tilde{\boldsymbol{\beta}}_{Z_1} \\ \vdots \\ \tilde{\boldsymbol{\beta}}_{Z_l} \\ \hat{\boldsymbol{b}} \end{pmatrix}$$

by minimizing L thus getting partial derivatives as 0's.

Expanding at the true parameters $\boldsymbol{b}^0 = (b_1^0, \cdots, b_{p_2}^0)^T, \boldsymbol{\beta}_{Z_j}^0 = (\boldsymbol{b}^{0T}\boldsymbol{\beta}_{Z_j\boldsymbol{H}}^0, \beta_{Z_jH_1}^0, \cdots, \beta_{Z_jH_{p_2}}^0)$ and combining higher order terms as $\mathbf{R}$, we have:

$$\mathbf{0} = \begin{pmatrix} \tilde{\mathbf{W}}\boldsymbol{\Sigma}_1^{-1}(\tilde{\boldsymbol{\beta}}_{Z_1} - \hat{\boldsymbol{\beta}}_{Z_1}) \\ \vdots \\ \tilde{\mathbf{W}}\boldsymbol{\Sigma}_l^{-1}(\tilde{\boldsymbol{\beta}}_{Z_l} - \hat{\boldsymbol{\beta}}_{Z_l}) \\ \sum_{j=1}^{l} \tilde{\mathbf{V}}_j\boldsymbol{\Sigma}_j^{-1}(\tilde{\boldsymbol{\beta}}_{Z_j} - \hat{\boldsymbol{\beta}}_{Z_j}) \end{pmatrix} = \begin{pmatrix} \mathbf{W}^0\boldsymbol{\Sigma}_1^{-1}(\boldsymbol{\beta}_{Z_1}^0 - \hat{\boldsymbol{\beta}}_{Z_1}) \\ \vdots \\ \mathbf{W}^0\boldsymbol{\Sigma}_l^{-1}(\boldsymbol{\beta}_{Z_l}^0 - \hat{\boldsymbol{\beta}}_{Z_l}) \\ \sum_{j=1}^{l} \mathbf{V}_j^0\boldsymbol{\Sigma}_j^{-1}(\boldsymbol{\beta}_{Z_j}^0 - \hat{\boldsymbol{\beta}}_{Z_j}) \end{pmatrix} - \boldsymbol{\mathcal{I}} \begin{pmatrix} \tilde{\boldsymbol{\beta}}_{Z_1\boldsymbol{H}} - \boldsymbol{\beta}_{Z_1\boldsymbol{H}}^0 \\ \vdots \\ \tilde{\boldsymbol{\beta}}_{Z_l\boldsymbol{H}} - \boldsymbol{\beta}_{Z_l\boldsymbol{H}}^0 \\ \hat{\boldsymbol{b}} - \boldsymbol{b}^0 \end{pmatrix} + \mathbf{R}, \tag{11}$$

here $\boldsymbol{\mathcal{I}} \in \mathbb{R}^{(lp_2+p_2)\times(lp_2+p_2)}$ is the Fisher Information matrix, i.e. the negative expectation of second order derivatives; this can be consistently estimated by MVMR-cML . The matrices $\tilde{\mathbf{W}}, \tilde{\mathbf{V}}_j$ are $\mathbf{W}, \mathbf{V}_j$ evaluated at the estimated values, and $\mathbf{W}^0, \mathbf{V}_j^0$ are $\mathbf{W}, \mathbf{V}_j$ evaluated at the true values. Each element of the reminder vector $\mathbf{R}$ is $(\hat{\boldsymbol{\theta}} - \boldsymbol{\theta}^0)^T \phi_t''(\boldsymbol{\theta}_t)(\hat{\boldsymbol{\theta}} - \boldsymbol{\theta}^0)$, $t = 1, \cdots, l+1$. $\boldsymbol{\theta}_t$ is on the line segment between $\boldsymbol{\theta}^0$ and $\hat{\boldsymbol{\theta}}$. Obviously $\phi_t''(\boldsymbol{\theta}_t)$ is $O(1)$. The consistency of MVMR-cML implies $(\hat{\boldsymbol{\theta}} - \boldsymbol{\theta}^0) \xrightarrow{P} \mathbf{0}$ and the remainder can be ignored.

Thus we have

$$\begin{pmatrix} \tilde{\boldsymbol{\beta}}_{Z_1\boldsymbol{H}} \\ \vdots \\ \tilde{\boldsymbol{\beta}}_{Z_l\boldsymbol{H}} \\ \hat{\boldsymbol{b}} \end{pmatrix} = \begin{pmatrix} \boldsymbol{\beta}_{Z_1\boldsymbol{H}}^0 \\ \vdots \\ \boldsymbol{\beta}_{Z_l\boldsymbol{H}}^0 \\ \boldsymbol{b}^0 \end{pmatrix} + \boldsymbol{\mathcal{I}}^{-1} \begin{pmatrix} \mathbf{W}^0\boldsymbol{\Sigma}_1^{-1}(\boldsymbol{\beta}_{Z_1}^0 - \hat{\boldsymbol{\beta}}_{Z_1}) \\ \vdots \\ \mathbf{W}^0\boldsymbol{\Sigma}_l^{-1}(\boldsymbol{\beta}_{Z_l}^0 - \hat{\boldsymbol{\beta}}_{Z_l}) \\ \sum_{j=1}^{l} \mathbf{V}_j^0\boldsymbol{\Sigma}_j^{-1}(\boldsymbol{\beta}_{Z_j}^0 - \hat{\boldsymbol{\beta}}_{Z_j}) \end{pmatrix} + o_p(1). \tag{12}$$

Partition $\boldsymbol{\mathcal{I}}^{-1}$ into

$$\boldsymbol{\mathcal{I}}^{-1} = \begin{pmatrix} \boldsymbol{\mathcal{I}}_1 \\ \boldsymbol{\mathcal{I}}_2 \end{pmatrix}, \tag{13}$$

with $\boldsymbol{\mathcal{I}}_1 \in \mathbb{R}^{lp_2 \times (lp_2+p_2)}$ and $\boldsymbol{\mathcal{I}}_2 \in \mathbb{R}^{p_2 \times (lp_2+p_2)}$, we have

$$\begin{pmatrix} \hat{b}_1 \\ \vdots \\ \hat{b}_p \end{pmatrix} = \begin{pmatrix} b_1^0 \\ \vdots \\ b_p^0 \end{pmatrix} + \boldsymbol{\mathcal{I}}_2 \begin{pmatrix} \mathbf{W}^0 \boldsymbol{\Sigma}_1^{-1} \boldsymbol{\beta}_{Z_1}^0 \\ \vdots \\ \mathbf{W}^0 \boldsymbol{\Sigma}_l^{-1} \boldsymbol{\beta}_{Z_l}^0 \\ \sum_{i=1}^l \mathbf{V}_j^0 \boldsymbol{\Sigma}_j^{-1} \boldsymbol{\beta}_{Z_j}^0 \end{pmatrix} + \boldsymbol{\Omega} \begin{pmatrix} \hat{\boldsymbol{\beta}}_{Z_1} \\ \vdots \\ \hat{\boldsymbol{\beta}}_{Z_l} \end{pmatrix} + o_p(1) = \mathbf{C} + \boldsymbol{\Omega} \begin{pmatrix} \hat{\boldsymbol{\beta}}_{Z_1} \\ \vdots \\ \hat{\boldsymbol{\beta}}_{Z_l} \end{pmatrix} + o_p(1), \tag{14}$$

here

$$\boldsymbol{\Omega} = -\boldsymbol{\mathcal{I}}_2 \begin{pmatrix} \mathbf{W}^0 \boldsymbol{\Sigma}_1^{-1} & & \\ & \ddots & \\ & & \mathbf{W}^0 \boldsymbol{\Sigma}_l^{-1} \\ \mathbf{V}_1^0 \boldsymbol{\Sigma}_1^{-1} & \cdots & \mathbf{V}_l^0 \boldsymbol{\Sigma}_l^{-1} \end{pmatrix}, \quad \mathbf{C} = \begin{pmatrix} b_1^0 \\ \vdots \\ b_p^0 \end{pmatrix} + \boldsymbol{\mathcal{I}}_2 \begin{pmatrix} \mathbf{W}^0 \boldsymbol{\Sigma}_1^{-1} \boldsymbol{\beta}_{Z_1}^0 \\ \vdots \\ \mathbf{W}^0 \boldsymbol{\Sigma}_l^{-1} \boldsymbol{\beta}_{Z_l}^0 \\ \sum_{i=1}^l \mathbf{V}_j^0 \boldsymbol{\Sigma}_j^{-1} \boldsymbol{\beta}_{Z_j}^0 \end{pmatrix}.$$

Now we can see, we have written the estimates $\hat{\boldsymbol{b}}$ as linear function of the estimated marginal effects $\hat{\boldsymbol{\beta}}_{Z_1}, \cdots, \hat{\boldsymbol{\beta}}_{Z_l}$, with the linear coefficients are elements in $\boldsymbol{\Omega}$. In practice, $\boldsymbol{\Omega}$ is substituted by $\hat{\boldsymbol{\Omega}}$, where the true values are replaced by $\hat{\boldsymbol{\beta}}_{Z_j}$ and $\hat{\boldsymbol{b}}$. And the consistency of $\hat{\boldsymbol{\beta}}_{Z_j}$ and $\hat{\boldsymbol{b}}$ guarantees $\hat{\boldsymbol{\Omega}} \xrightarrow{P} \boldsymbol{\Omega}$ as $N \to +\infty$.

Suppose our target SNP is denoted as G_i, and the corresponding effect estimates are

$$\hat{\boldsymbol{\beta}}_{G_i} = (\hat{\beta}_{G_iY}^C, \hat{\beta}_{G_iH_1}, \cdots, \hat{\beta}_{G_iH_{p_2}})^T.$$

G_i could be any SNP from the genome, thus it could be independent of all $Z_1, \cdots, Z_l$; or it could be identical to some SNP in $Z_1, \cdots, Z_l$, i.e. there exists $1 \le j \le l$ such that $G_i = Z_j$; or it could be in linkage disequilibrium (LD) with a subset of SNP(s) in $Z_1, \cdots, Z_l$. The asymptotic distribution is

$$\begin{pmatrix} \hat{\boldsymbol{\beta}}_{G_i} \\ \hat{\boldsymbol{\beta}}_{Z_1} \\ \vdots \\ \hat{\boldsymbol{\beta}}_{Z_l} \end{pmatrix} \xrightarrow{D} N \left[\begin{pmatrix} \boldsymbol{\beta}_{G_i}^0 \\ \boldsymbol{\beta}_{Z_1}^0 \\ \vdots \\ \boldsymbol{\beta}_{Z_l}^0 \end{pmatrix}, \boldsymbol{\Psi} = \begin{pmatrix} \boldsymbol{\Sigma}_{G_i} & \boldsymbol{\Phi}_1 & \boldsymbol{\Phi}_2 & \cdots & \boldsymbol{\Phi}_l \\ \boldsymbol{\Phi}_1 & \boldsymbol{\Sigma}_1 & & & \\ \boldsymbol{\Phi}_2 & & \boldsymbol{\Sigma}_2 & & \\ \vdots & & & \ddots & \\ \boldsymbol{\Phi}_l & & & & \boldsymbol{\Sigma}_l \end{pmatrix} \right]. \tag{15}$$

We will show how to obtain matrices $\boldsymbol{\Phi}$'s in the next section. The covariance matrix $\boldsymbol{\Sigma}_{G_i}$, $\boldsymbol{\Sigma}_1$, $\cdots$, $\boldsymbol{\Sigma}_l$ can be estimated using GWAS summary data and null z scores of

independent SNPs [2]. Now we have

$$\begin{pmatrix} \hat{\boldsymbol{\beta}}_{G_i} \\ \hat{\boldsymbol{b}} \end{pmatrix} = \begin{pmatrix} \mathbf{I}_{(p_2+1)\times(p_2+1)} & \\ & \boldsymbol{\Omega} \end{pmatrix} \begin{pmatrix} \hat{\boldsymbol{\beta}}_{G_i} \\ \hat{\boldsymbol{\beta}}_{Z_1} \\ \vdots \\ \hat{\boldsymbol{\beta}}_{Z_l} \end{pmatrix} + \begin{pmatrix} \mathbf{0} \\ \mathbf{C} \end{pmatrix} + o_p(1).$$

Hence, the vector of estimates $(\hat{\beta}^C_{G_iY}, \hat{\beta}_{G_iH_1}, \cdots, \hat{\beta}_{G_iH_{p_2}}, \hat{b}_1, \cdots, \hat{b}_{p_2})^T$ is a linear combination of GWAS summary statistics and has an asymptotic normal distribution. We have:

$$\begin{pmatrix} \hat{\boldsymbol{\beta}}_{G_i} \\ \hat{\boldsymbol{b}} \end{pmatrix} \xrightarrow{D} N\left\{ \begin{pmatrix} \boldsymbol{\beta}^0_{G_i} \\ \boldsymbol{b}^0 \end{pmatrix}, \boldsymbol{U} \right\},$$

and

$$\boldsymbol{U} = \begin{pmatrix} \mathbf{I}_{(p_2+1)\times(p_2+1)} & \\ & \boldsymbol{\Omega} \end{pmatrix} \boldsymbol{\Psi} \begin{pmatrix} \mathbf{I}_{(p_2+1)\times(p_2+1)} & \\ & \boldsymbol{\Omega} \end{pmatrix}^T.$$

In practice, we substitute $\boldsymbol{\Omega}$ with $\hat{\boldsymbol{\Omega}}$, and denote the corresponding estimator as $\hat{\boldsymbol{U}}$. Assuming $\boldsymbol{\Psi}$ is known or well-estimated, we have $\hat{\boldsymbol{U}} \xrightarrow{P} \boldsymbol{U}$ since $\hat{\boldsymbol{\Omega}} \xrightarrow{P} \boldsymbol{\Omega}$.

Applying Delta method, we have $\hat{\beta}_{G_iY} = \hat{\beta}^C_{G_iY} - \hat{\boldsymbol{b}}^T \hat{\boldsymbol{\beta}}_{G_i\boldsymbol{H}} \xrightarrow{D} N(\beta^0_{G_iY}, \sigma^2_{G_iY})$ where

$$\sigma^2_{G_iY} = \begin{pmatrix} 1 \\ -\boldsymbol{b}^0 \\ -\boldsymbol{\beta}^0_{G_i\boldsymbol{H}} \end{pmatrix}^T \boldsymbol{U} \begin{pmatrix} 1 \\ -\boldsymbol{b}^0 \\ -\boldsymbol{\beta}^0_{G_i\boldsymbol{H}} \end{pmatrix}. \tag{16}$$

Substitute the true values on the two sides of (16) by point estimates, we have

$$\hat{\sigma}^2_{G_iY} = \begin{pmatrix} 1 \\ -\hat{\boldsymbol{b}} \\ -\hat{\boldsymbol{\beta}}_{G_i\boldsymbol{H}} \end{pmatrix}^T \hat{\boldsymbol{U}} \begin{pmatrix} 1 \\ -\hat{\boldsymbol{b}} \\ -\hat{\boldsymbol{\beta}}_{G_i\boldsymbol{H}} \end{pmatrix},$$

and $\hat{\sigma}^2_{G_iY} \xrightarrow{P} \sigma^2_{G_iY}$. Finally by by Slutsky's Theorem we have $(\hat{\beta}_{G_iY} - \beta^0_{G_iY})/\hat{\sigma}_{G_iY} \xrightarrow{D} N(0, 1)$. □

B.3 Estimation of the covariance matrix

We can see, the elements in $\boldsymbol{\Phi}$'s are actually the covariance between estimated marginal effects of G_i on H's, Y and estimated marginal effects of IVs Z_j on H's, Y. So suppose

we have two traits H and Y, and two SNPs G and Z, here G and Z could be identical, or independent, or in linkage disequilibrium (LD). For simplicity, through out this section we use α and β to denote SNP effects. Then our model is

$$\begin{aligned} H &= \alpha \cdot G + \epsilon, \\ Y &= \beta \cdot Z + \delta, \end{aligned} \tag{17}$$

here ϵ, δ are random errors, we have $Var(\epsilon) = \sigma_1^2$, $Var(\delta) = \sigma_2^2$, and $Cov(\epsilon, \delta) = \sigma_{12}$, $Cor(\epsilon, \delta) = \rho$.

Now suppose we have two GWASs for H and Y respectively, and these two GWASs could have overlapped sample. We dissect all samples into three parts: (1) the first sample of size n_1 is for H only, denoted by $\boldsymbol{H}_1 \in \mathbb{R}^{n_1}$ and $\boldsymbol{G}_1 \in \mathbb{R}^{n_1}$; (2) the second sample of size n_2 is for both H and Y, denoted by $\boldsymbol{H}_2 \in \mathbb{R}^{n_2}$, $\boldsymbol{G}_2 \in \mathbb{R}^{n_2}$, $\boldsymbol{Y}_2 \in \mathbb{R}^{n_2}$, $\boldsymbol{Z}_2 \in \mathbb{R}^{n_2}$; (3) the third sample of size n_3 is for Y only, denoted by $\boldsymbol{Y}_3 \in \mathbb{R}^{n_3}$, $\boldsymbol{Z}_3 \in \mathbb{R}^{n_3}$. Thus we have

$$\begin{aligned} &\boldsymbol{H}_1 = \alpha \cdot \boldsymbol{G}_1 + \boldsymbol{\epsilon}_1, \\ &\boldsymbol{H}_2 = \alpha \cdot \boldsymbol{G}_2 + \boldsymbol{\epsilon}_2, \quad \boldsymbol{Y}_2 = \beta \cdot \boldsymbol{Z}_2 + \boldsymbol{\delta}_2, \\ &\qquad\qquad\qquad\qquad\quad \boldsymbol{Y}_3 = \beta \cdot \boldsymbol{Z}_3 + \boldsymbol{\delta}_3. \end{aligned} \tag{18}$$

Now we can get the estimated marginal effects as

$$\begin{aligned} \hat{\alpha} &= (\boldsymbol{G}_1^T\boldsymbol{G}_1 + \boldsymbol{G}_2^T\boldsymbol{G}_2)^{-1}(\boldsymbol{G}_1^T\boldsymbol{X}_1 + \boldsymbol{G}_2^T\boldsymbol{X}_2) = \alpha + (\boldsymbol{G}_1^T\boldsymbol{G}_1 + \boldsymbol{G}_2^T\boldsymbol{G}_2)^{-1}(\boldsymbol{G}_1^T\boldsymbol{\epsilon}_1 + \boldsymbol{G}_2^T\boldsymbol{\epsilon}_2), \\ \hat{\beta} &= (\boldsymbol{Z}_2^T\boldsymbol{Z}_2 + \boldsymbol{Z}_3^T\boldsymbol{Z}_3)^{-1}(\boldsymbol{Z}_2^T\boldsymbol{Y}_2 + \boldsymbol{Z}_3^T\boldsymbol{Y}_3) = \beta + (\boldsymbol{Z}_2^T\boldsymbol{Z}_2 + \boldsymbol{Z}_3^T\boldsymbol{Z}_3)^{-1}(\boldsymbol{Z}_2^T\boldsymbol{\delta}_2 + \boldsymbol{Z}_3^T\boldsymbol{\delta}_3), \end{aligned} \tag{19}$$

thus we have

$$\begin{aligned} Cov(\hat{\alpha}, \hat{\beta}) &= Cov((\boldsymbol{G}_1^T\boldsymbol{G}_1 + \boldsymbol{G}_2^T\boldsymbol{G}_2)^{-1}\boldsymbol{G}_2^T\boldsymbol{\epsilon}_2, (\boldsymbol{Z}_2^T\boldsymbol{Z}_2 + \boldsymbol{Z}_3^T\boldsymbol{Z}_3)^{-1}\boldsymbol{Z}_2^T\boldsymbol{\delta}_2) \\ &= (\boldsymbol{G}_1^T\boldsymbol{G}_1 + \boldsymbol{G}_2^T\boldsymbol{G}_2\boldsymbol{Z}_2 + \boldsymbol{Z}_3^T\boldsymbol{Z}_3)^{-1}Cov(\boldsymbol{G}_2^T\boldsymbol{\epsilon}_2, \boldsymbol{Z}_2^T\boldsymbol{\delta}_2) \\ &= (\boldsymbol{G}_1^T\boldsymbol{G}_1 + \boldsymbol{G}_2^T\boldsymbol{G}_2)^{-1}(\boldsymbol{Z}_2^T\boldsymbol{Z}_2 + \boldsymbol{Z}_3^T\boldsymbol{Z}_3)^{-1} \cdot \sigma_{12} \cdot \boldsymbol{G}_2^T\boldsymbol{Z}_2 \\ &\approx \frac{\sigma_{12} \cdot n_2 \cdot Cov(G, Z)}{(n_1 + n_2) \cdot Var(G) \cdot (n_2 + n_3) \cdot Var(Z)}, \end{aligned} \tag{20}$$

and

$$\begin{aligned} Var(\hat{\alpha}) &\approx \frac{\sigma_1^2}{(n_1 + n_2)Var(G)}, \\ Var(\hat{\beta}) &\approx \frac{\sigma_2^2}{(n_2 + n_3)Var(Z)}, \end{aligned} \tag{21}$$

thus we have

$$\begin{aligned} Cor(\hat{\alpha}, \hat{\beta}) &= \frac{n_2}{\sqrt{(n_1+n_2)(n_2+n_3)}} \cdot \frac{\sigma_{12}}{\sigma_1 \cdot \sigma_2} \cdot \frac{Cov(G,Z)}{\sqrt{Var(G) \cdot Var(Z)}} \\ &= \frac{n_2}{\sqrt{(n_1+n_2)(n_2+n_3)}} \cdot \rho \cdot Cor(G,Z). \end{aligned} \tag{22}$$

Equation (22) is consistent with previous literature [2]. Here $Cor(G, Z)$ is the LD between G and Z, which could be estimated with a reference panel. When the SNP effects α, β are small, we have $\rho = Cor(\epsilon, \delta) \approx Cor(H, Y)$. So in a special case when $G = Z$, we have $Cor(\hat{\alpha}, \hat{\beta}) = \frac{n_2}{\sqrt{(n_1+n_2)(n_2+n_3)}} \cdot \rho$.

In practice, we first use pairwise null z-scores in GWASs of H and Y, or the intercept of Linkage disequilibrium score (LDSC) regression to estimate $\frac{n_2}{\sqrt{(n_1+n_2)(n_2+n_3)}} \cdot \rho$ [2], then we could simply multiplying the LD $Cor(G, Z)$ to get $Cor(\hat{\alpha}, \hat{\beta})$.

C Simulation without pleiotropy

C.1 Simulation set up

In the absence of pleiotropy, our simulation involved the generation of 1000 independent SNPs from a binomial distribution $Bin(2, p)$. The genotypes G_i were centered to achieve a sample mean of 0. Among these SNPs, 50 affected $\boldsymbol{H}$ only, 50 affected Y only. Others were null SNPs having effect 0 on both covariates and the outcome. Minor allele frequencies (MAFs) for these SNPs were generated from a uniform distribution ranging between 0.01 and 0.49. All SNP effects on both $\boldsymbol{H}$ and Y as well as the causal effects of $\boldsymbol{H}$ on Y ($\boldsymbol{\beta}_{\boldsymbol{H}Y}$), were independently drawn from a standard normal distribution. All SNP effects and MAFs were predetermined before initiating the simulation. The confounder U and error terms $E_{\boldsymbol{H}}$, E_Y were drawn from normal distributions with a mean of 0. U accounted for 40% of the unknown variance in both Y and the covariates within $\boldsymbol{H}$. The error terms contributed to 10% of the unknown variance in the covariates and outcome, respectively. The values of $\boldsymbol{H}$ and Y were subsequently determined based on the equations below, incorporating the effects, confounders, and error terms.

$$\boldsymbol{H} = \sum_{i=1}^{1000} \boldsymbol{\beta}_{G_i \boldsymbol{H}} G_i + \boldsymbol{\beta}_{U\boldsymbol{H}} U + E_{\boldsymbol{H}}, \tag{23}$$

$$Y = \sum_{i=1}^{1000} \beta_{G_i Y} G_i + \beta_{UY} U + \boldsymbol{\beta}_{\boldsymbol{H}Y}^T \boldsymbol{H} + E_Y. \tag{24}$$

The GWAS analysis of Y was performed using $\boldsymbol{H}$ as covariates, while the GWAS analysis of the elements in $\boldsymbol{H}$ only included the SNPs. The p-value of $5e-8$ was used to select relative IVs. This simulation followed a similar structure to those of Dudbridge et al. (2019) [1]. We only presented the simulation result of SNPs suffering from collider bias, i.e., those SNPs affecting $\boldsymbol{H}$. The results were summarized in the same way in the main paper. Without pleiotropy, the power of SNPs affecting Y only did not increase after bias correction. Hence, we only identified the SNPs with greatest decrease in power.

C.2 Simulation results

Table A. Empirical type-I error rate (for SNPs underlined) and power with and without bias correction. Sample standard deviations (SD) are given in parenthesis. SNPs were independent and no pleiotropy effects were generated.

Dimension of $\boldsymbol{H}$	1								2						4					
Bias correction	No	cML	Egger	IVW	Lasso	Median	DHO	SH	No	cML	Egger	IVW	Lasso	Median	No	cML	Egger	IVW	Lasso	Median
Null SNPs	0.05	0.05	0.05	0.05	0.05	0.05	0.05	0.05	0.05	0.06	0.06	0.06	0.06	0.06	0.05	0.06	0.06	0.06	0.06	0.06
(SD)	(0.01)	(0.01)	(0.01)	(0.01)	(0.01)	(0.01)	(0.01)	(0.01)	(0.01)	(0.01)	(0.01)	(0.01)	(0.01)	(0.01)	(0.01)	(0.01)	(0.01)	(0.01)	(0.01)	(0.01)
All SNPs affecting $\boldsymbol{H}$ but not Y	0.85	0.05	0.05	0.05	0.05	0.05	0.05	0.05	0.81	0.05	0.05	0.05	0.05	0.05	0.76	0.06	0.06	0.06	0.06	0.05
(SD)	(0.03)	(0.03)	(0.04)	(0.03)	(0.03)	(0.03)	(0.04)	(0.03)	(0.03)	(0.03)	(0.03)	(0.03)	(0.03)	(0.03)	(0.03)	(0.03)	(0.03)	(0.03)	(0.03)	(0.03)
All SNPs affecting Y only	0.49	0.42	0.42	0.42	0.42	0.42	0.42	0.42	0.68	0.61	0.61	0.61	0.61	0.61	0.90	0.86	0.86	0.86	0.86	0.86
(SD)	(0.05)	(0.05)	(0.05)	(0.05)	(0.05)	(0.05)	(0.05)	(0.05)	(0.04)	(0.05)	(0.04)	(0.04)	(0.04)	(0.04)	(0.02)	(0.03)	(0.03)	(0.03)	(0.03)	(0.03)
SNP with greatest decrease in power	0.78	0.59	0.59	0.60	0.60	0.59	0.59	0.67	0.76	0.53	0.53	0.65	0.65	0.54	0.81	0.54	0.54	0.54	0.54	0.54
(SD)	(0.41)	(0.49)	(0.49)	(0.49)	(0.49)	(0.49)	(0.49)	(0.47)	(0.43)	(0.50)	(0.50)	(0.48)	(0.48)	(0.50)	(0.39)	(0.50)	(0.50)	(0.50)	(0.50)	(0.50)

Table B. Mean effect estimates, empirical type-I error rate (for those SNPs with true effect 0 and underlined) and power with and without bias correction for some representative SNPs. Sample standard deviations (SD) and mean standard errors (Mean SE) are given in parenthesis. SNPs were independent and no pleiotropy effects were generated.

p_2	True effects		Mean effect estimates								Type-I error rate							
			No	cML	Egger	IVW	Lasso	Median	DHO	SH	No	cML	Egger	IVW	Lasso	Median	DHO	SH
1			4.73	0.01	0.09	0.05	0.05	0.05	−0.03	−0.00	1.00	0.05	0.04	0.04	0.04	0.04	0.03	0.04
	0.00	(SD)	(0.28)	(0.34)	(0.39)	(0.34)	(0.34)	(0.34)	(0.40)	(0.35)	(0.00)	(0.21)	(0.20)	(0.19)	(0.19)	(0.19)	(0.17)	(0.18)
		(Mean SE)	(0.29)	(0.34)	(0.42)	(0.36)	(0.36)	(0.37)	(0.42)	(0.37)	NA	NA	NA	NA	NA	NA	NA	NA
			−1.48	0.01	−0.01	0.00	0.00	0.00	0.03	0.02	1.00	0.05	0.06	0.05	0.05	0.05	0.06	0.05
	0.00	(SD)	(0.33)	(0.39)	(0.41)	(0.39)	(0.39)	(0.39)	(0.42)	(0.39)	(0.06)	(0.22)	(0.23)	(0.22)	(0.22)	(0.22)	(0.24)	(0.23)
		(Mean SE)	(0.32)	(0.40)	(0.40)	(0.39)	(0.39)	(0.39)	(0.40)	(0.39)	NA	NA	NA	NA	NA	NA	NA	NA
2			1.23	−0.04	−0.03	−0.03	−0.03	−0.03			1.00	0.05	0.05	0.05	0.05	0.05		
	0.00	(SD)	(0.14)	(0.18)	(0.19)	(0.18)	(0.18)	(0.18)	NA	NA	(0.00)	(0.23)	(0.22)	(0.22)	(0.22)	(0.21)	NA	NA
		(Mean SE)	(0.14)	(0.18)	(0.18)	(0.18)	(0.18)	(0.19)			NA	NA	NA	NA	NA	NA		
			0.50	0.01	0.03	0.02	0.02	0.02			0.94	0.04	0.04	0.05	0.05	0.04		
	0.00	(SD)	(0.14)	(0.18)	(0.19)	(0.18)	(0.18)	(0.18)	NA	NA	(0.24)	(0.18)	(0.21)	(0.22)	(0.22)	(0.20)	NA	NA
		(Mean SE)	(0.14)	(0.19)	(0.19)	(0.18)	(0.18)	(0.19)			NA	NA	NA	NA	NA	NA		
4			1.31	0.02	0.04	0.04	0.04	0.03			1.00	0.06	0.05	0.04	0.04	0.03		
	0.00	(SD)	(0.10)	(0.14)	(0.14)	(0.14)	(0.14)	(0.14)	NA	NA	(0.00)	(0.24)	(0.21)	(0.21)	(0.21)	(0.18)	NA	NA
		(Mean SE)	(0.10)	(0.13)	(0.14)	(0.14)	(0.14)	(0.15)			NA	NA	NA	NA	NA	NA		
			−0.18	0.01	0.00	0.01	0.01	0.01			0.61	0.05	0.06	0.06	0.06	0.05		
	0.00	(SD)	(0.08)	(0.12)	(0.12)	(0.11)	(0.11)	(0.11)	NA	NA	(0.49)	(0.22)	(0.24)	(0.23)	(0.23)	(0.22)	NA	NA
		(Mean SE)	(0.08)	(0.12)	(0.11)	(0.11)	(0.11)	(0.11)			NA	NA	NA	NA	NA	NA		

Table C. True values and mean estimates of $\boldsymbol{b}$. Sample standard deviations (SD) and mean standard errors (Mean SE) are given in parenthesis. SNPs were independent and no pleiotropy effects were generated.

Dimension of $\boldsymbol{b}$	1	2		4			
$\boldsymbol{b}$	b_1	b_1	b_2	b_1	b_2	b_3	b_4
True value	−3.27	−1.26	−1.05	−0.48	−0.52	−0.50	−0.40
MVMR-cML	−3.31	−1.25	−1.12	−0.52	−0.54	−0.52	−0.42
(SD)	(0.05)	(0.03)	(0.02)	(0.02)	(0.02)	(0.02)	(0.02)
(Mean SE)	(0.06)	(0.03)	(0.03)	(0.02)	(0.02)	(0.02)	(0.02)
MVMR-Egger	−3.44	−1.25	−1.11	−0.52	−0.54	−0.51	−0.41
(SD)	(0.15)	(0.06)	(0.02)	(0.03)	(0.02)	(0.02)	(0.02)
(Mean SE)	(0.15)	(0.07)	(0.03)	(0.04)	(0.02)	(0.02)	(0.02)
MVMR-IVW	−3.30	−1.23	−1.11	−0.52	−0.54	−0.51	−0.41
(SD)	(0.05)	(0.03)	(0.02)	(0.02)	(0.02)	(0.02)	(0.02)
(Mean SE)	(0.06)	(0.03)	(0.03)	(0.02)	(0.02)	(0.02)	(0.02)
MVMR-Lasso	−3.30	−1.23	−1.11	−0.52	−0.54	−0.51	−0.41
(SD)	(0.05)	(0.03)	(0.02)	(0.02)	(0.02)	(0.02)	(0.02)
(Mean SE)	(0.06)	(0.03)	(0.03)	(0.02)	(0.02)	(0.02)	(0.02)
MVMR-Median	−3.32	−1.24	−1.12	−0.52	−0.54	−0.51	−0.41
(SD)	(0.05)	(0.03)	(0.02)	(0.02)	(0.02)	(0.02)	(0.02)
(Mean SE)	(0.08)	(0.05)	(0.04)	(0.03)	(0.03)	(0.03)	(0.03)
DHO	−3.56						
(SD)	(0.16)	NA	NA	NA	NA	NA	NA
(Mean SE)	(0.16)						
SH	−3.33						
(SD)	(0.06)	NA	NA	NA	NA	NA	NA
(Mean SE)	(0.06)						

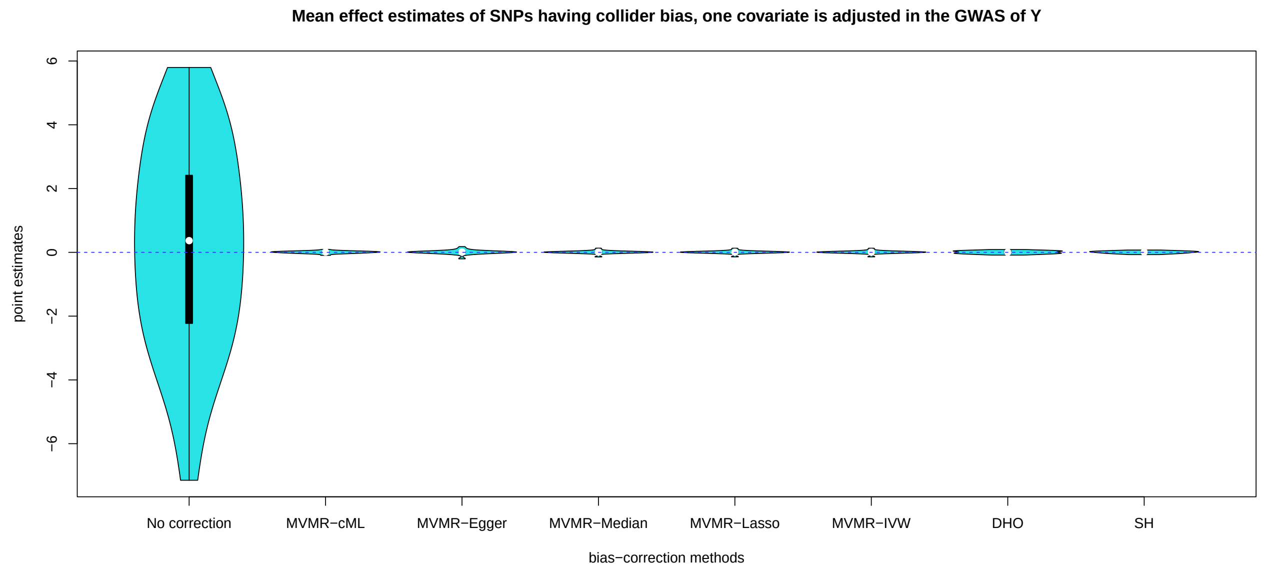

(a)

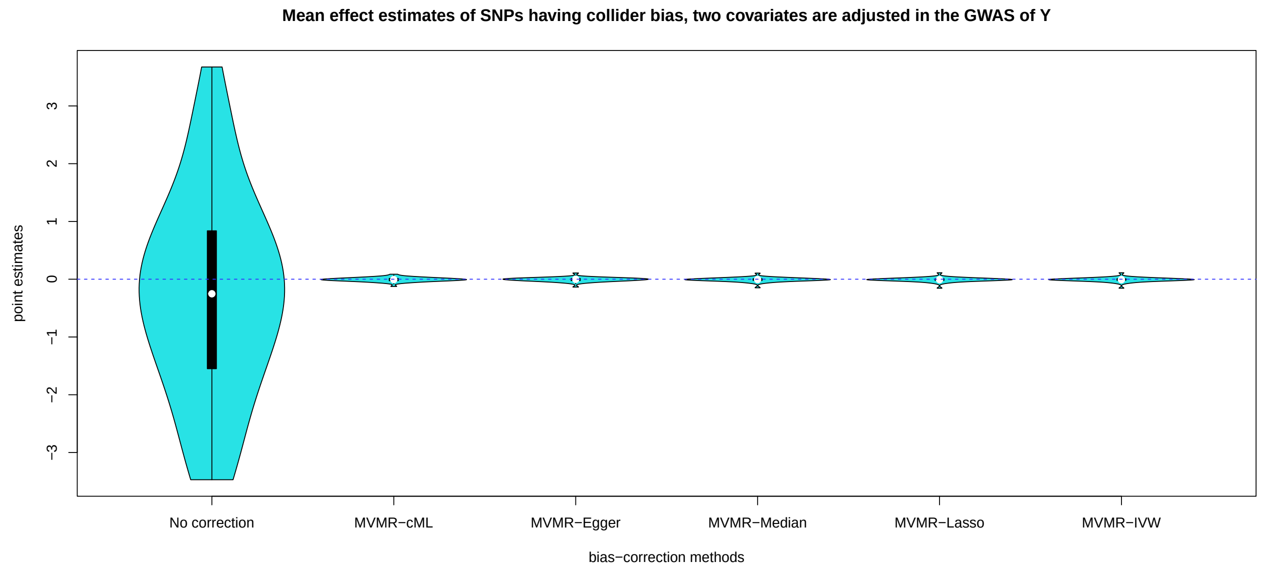

(b)

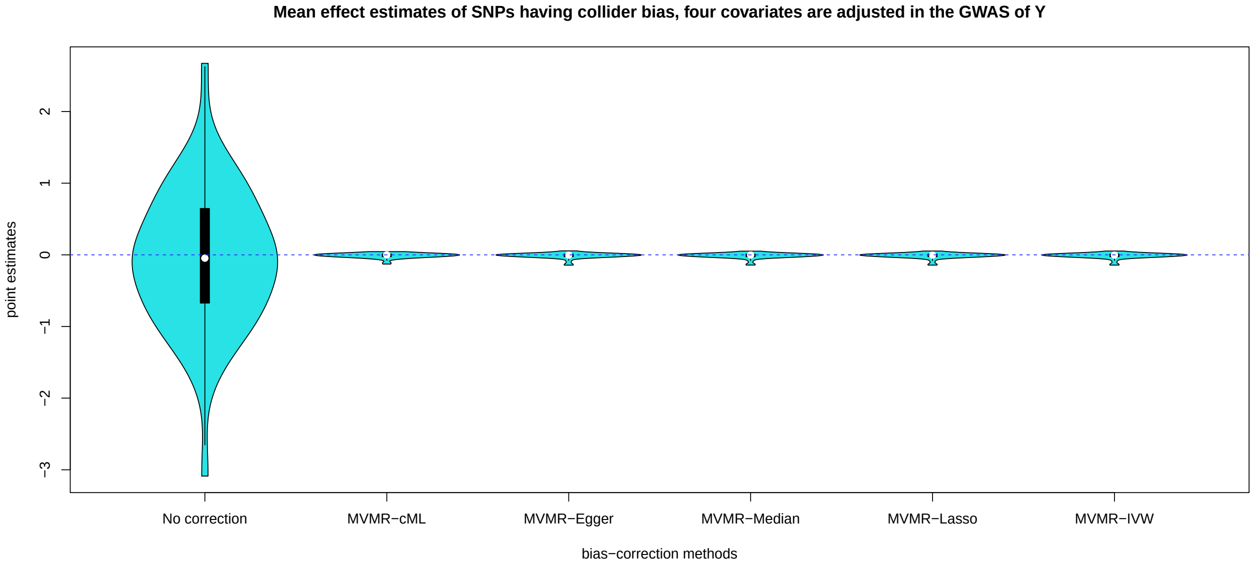

(c)

Fig A. Violin plots of mean effect estimates of the 50 SNPs affecting covariates only. The true effect 0 is highlighted by the dashed line. The labels of X-axis indicate the MVMR methods. (a): $p_2 = 1$, (b): $p_2 = 2$, (c): $p_2 = 4$. SNPs were independent and no pleiotropy effects were generated.

D Simulation without collider bias

In this section we gave the simulation without collider bias, as suggested by one of the reviewers. Specifically, we generated $U_{\boldsymbol{H}}$ and U_Y independently for $\boldsymbol{H}$ and Y, accounting for 40% of the variances of covariates and outcome:

$$\boldsymbol{H} = \sum_{i=1}^{1000} \boldsymbol{\beta}_{G_i \boldsymbol{H}} G_i + \boldsymbol{\beta}_{U\boldsymbol{H}} U_{\boldsymbol{H}} + E_{\boldsymbol{H}}, \tag{25}$$

$$Y = \sum_{i=1}^{1000} \beta_{G_i Y} G_i + \beta_{UY} U_Y + \boldsymbol{\beta}_{\boldsymbol{H}Y}^T \boldsymbol{H} + E_Y. \tag{26}$$

Hence, there is no common confounder for covariates and outcome ($\boldsymbol{b} = \boldsymbol{0}$), and hence no collider bias. Two covariates were included in $\boldsymbol{H}$. Other simulation set up were the same as those in section 3 of this supplementary. No pleiotropy effects were generated and all IVs are valid.

Table D. True values and mean estimates of $\boldsymbol{b}$. Sample standard deviations (SD) and mean standard errors (Mean SE) are given in parenthesis. $p_2 = 2$. No collider bias is generated.

Dimension of $\boldsymbol{b}$	2	
$\boldsymbol{b}$	b_1	b_2
True value	0.00	0.00
MVMR-cML	0.00	0.00
(SD)	(0.02)	(0.02)
(Mean SE)	(0.03)	(0.03)
MVMR-Egger	0.00	0.00
(SD)	(0.06)	(0.02)
(Mean SE)	(0.07)	(0.03)
MVMR-IVW	0.00	0.00
(SD)	(0.02)	(0.02)
(Mean SE)	(0.03)	(0.03)
MVMR-Lasso	0.00	0.00
(SD)	(0.02)	(0.02)
(Mean SE)	(0.03)	(0.03)
MVMR-Median	0.00	0.00
(SD)	(0.02)	(0.02)
(Mean SE)	(0.05)	(0.04)

Table E. Mean effect estimates, empirical type-I error rate and power with and without bias correction for some representative SNPs. Sample standard deviations (SD) and mean standard errors (Mean SE) are given in parenthesis. Type-I error rates were computed for SNPs not affecting Y, whose true effects were 0 and underlined. $p_2 = 2$. No collider bias is generated.

	True effects		Mean effect estimates						Type-I error rate or power					
			No	cML	Egger	IVW	Lasso	Median	No	cML	Egger	IVW	Lasso	Median
SNPs affect $\boldsymbol{H}$ only	$\underline{0.00}$		0.00	0.00	0.00	0.00	0.00	0.00	0.04	0.03	0.04	0.04	0.04	0.03
		(SD)	(0.22)	(0.21)	(0.22)	(0.21)	(0.21)	(0.22)	(0.20)	(0.18)	(0.19)	(0.18)	(0.18)	(0.18)
		(Mean SE)	(0.22)	(0.23)	(0.23)	(0.22)	(0.22)	(0.22)	NA	NA	NA	NA	NA	NA
	$\underline{0.00}$		−0.01	−0.01	−0.01	−0.01	−0.01	−0.01	0.06	0.06	0.06	0.06	0.06	0.06
		(SD)	(0.26)	(0.26)	(0.27)	(0.26)	(0.26)	(0.26)	(0.23)	(0.23)	(0.23)	(0.23)	(0.23)	(0.23)
		(Mean SE)	(0.27)	(0.27)	(0.27)	(0.27)	(0.27)	(0.27)	NA	NA	NA	NA	NA	NA
SNPs affect Y only	−2.55		−2.55	−2.55	−2.55	−2.55	−2.55	−2.55	1.00	1.00	1.00	1.00	1.00	1.00
		(SD)	(0.20)	(0.20)	(0.20)	(0.20)	(0.20)	(0.20)	(0.00)	(0.00)	(0.00)	(0.00)	(0.00)	(0.00)
		(Mean SE)	(0.21)	(0.21)	(0.21)	(0.21)	(0.21)	(0.21)	NA	NA	NA	NA	NA	NA
	0.57		0.56	0.56	0.56	0.56	0.56	0.56	0.57	0.58	0.57	0.58	0.58	0.58
		(SD)	(0.27)	(0.27)	(0.27)	(0.27)	(0.27)	(0.27)	(0.49)	(0.49)	(0.49)	(0.49)	(0.49)	(0.49)
		(Mean SE)	(0.26)	(0.26)	(0.26)	(0.26)	(0.26)	(0.26)	NA	NA	NA	NA	NA	NA
Null SNPs	$\underline{0.00}$		0.00	0.00	0.00	0.00	0.00	0.00	0.05	0.05	0.05	0.05	0.05	0.05
		(SD)	(0.18)	(0.18)	(0.18)	(0.18)	(0.18)	(0.18)	(0.22)	(0.22)	(0.22)	(0.22)	(0.22)	(0.22)
		(Mean SE)	(0.18)	(0.18)	(0.18)	(0.18)	(0.18)	(0.18)	NA	NA	NA	NA	NA	NA
	$\underline{0.00}$		0.01	0.01	0.01	0.01	0.01	0.01	0.04	0.04	0.04	0.04	0.04	0.04
		(SD)	(0.24)	(0.24)	(0.24)	(0.24)	(0.24)	(0.24)	(0.19)	(0.19)	(0.19)	(0.19)	(0.19)	(0.19)
		(Mean SE)	(0.25)	(0.25)	(0.25)	(0.25)	(0.25)	(0.25)	NA	NA	NA	NA	NA	NA

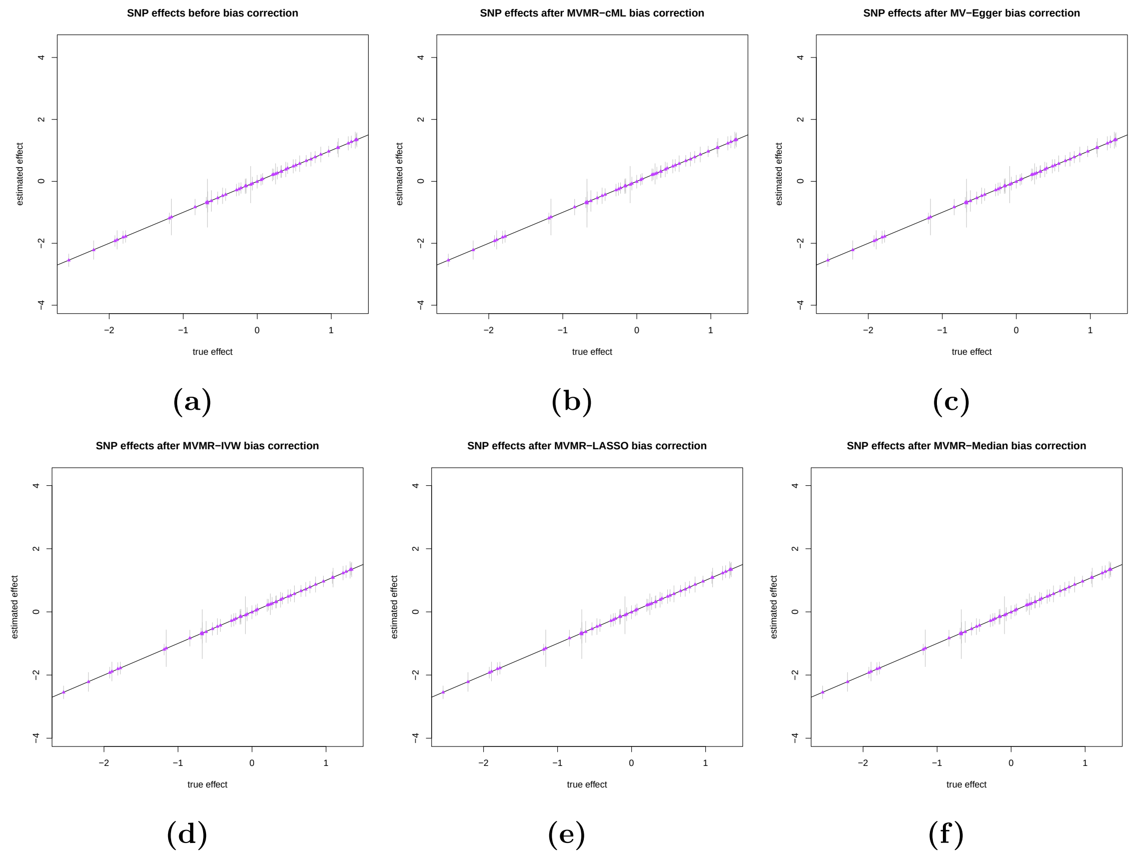

Fig B. Mean effect estimates of 50 SNPs affect Y only. No collider bias is simulated. Horizontal coordinates are for the true effects, vertical coordinates are for the estimated effects. Vertical bars are the means of standard errors averaged over 1000 repetitions. The identity line is included for comparison.

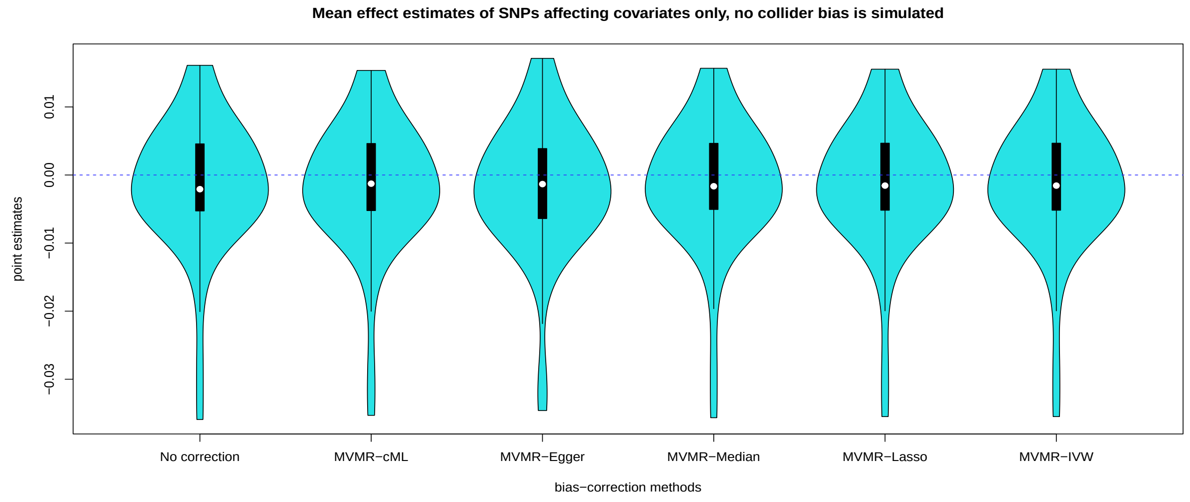

Fig C. Violin plots of the mean effect estimates of 50 SNPs affect covariates only. No collider bias is simulated. The true effect 0 is highlighted by a dashed line.

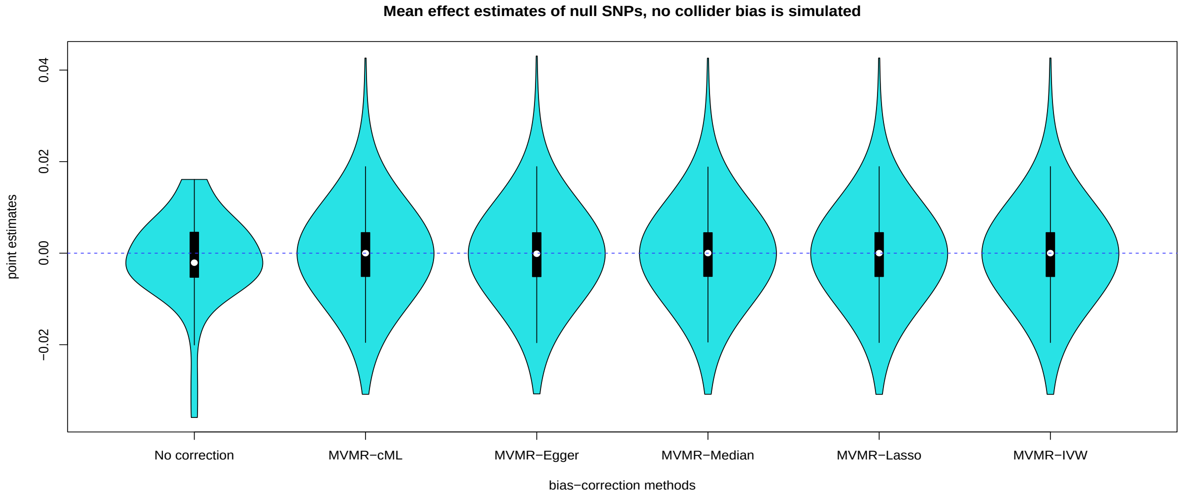

Fig D. Violin plots of the mean effect estimates of 900 null SNPs. No collider bias is simulated. The true effect 0 is highlighted by a dashed line.

E Comparison of UVMR and MVMR

E.1 Simulation

As recommended by one of the reviewers, we conducted a simulation to illustrate the limitations of univariate Mendelian randomization (UVMR) when collider bias was introduced by two covariates H_1 and H_2. We utilized the same simulation setup as in Section 3, where independent SNPs were generated. No pleiotropy effects were simulated, and all IVs were valid. In the application of UVMR, the two components in $\boldsymbol{b}$ were estimated separately. The variance of the bias-corrected estimator $\hat{\beta}_{G_iY}$ is computed using equation (29), assuming $\hat{\boldsymbol{\Sigma}}_{\hat{\boldsymbol{b}}}$ to be diagonal.

We compared the estimation results of UVMR-cML [4] and UVMR-Egger [5] regression with their corresponding MVMR approaches. The two previous approaches proposed by Dudbridge et al. (2019) [1] and Mahmoud et al. (2022) [6] were also included for comparison. The findings revealed that UVMR methods were unable to provide an accurate estimate of $\boldsymbol{b}$, consequently failing to address collider bias. In our simulation setup, each IV Z influenced both covariates H_1 and H_2. In UVMR, where only one covariate was considered, there existed a pleiotropy path either $Z \to H_1 \to Y$ or $Z \to H_2 \to Y$. Thus, although all IVs were valid for MVMR, none were valid for UVMR [3]. For UVMR-cML, the plurality condition was violated, and the information approach selected only three IVs for final estimation, resulting in a severely biased $\hat{\boldsymbol{b}}$.

When $\boldsymbol{b}$ is not well-estimated, the bias-correction procedure may also produce an inaccurate SNP effect estimate. The results of some representative SNPs are given in Table G. We only present the SNPs having collider bias, i.e., the SNPs affecting

covariates only.

Table F. True values and mean estimates of $\boldsymbol{b}$. Sample standard deviations (SD) and mean standard errors (Mean SE) are given in parenthesis. $p_2 = 2$.

Dimension of $\boldsymbol{b}$	2	
$\boldsymbol{b}$	b_1	b_2
True value	−1.26	−1.05
MVMR-cML	−1.24	−1.12
(SD)	(0.03)	(0.03)
(Mean SE)	(0.03)	(0.03)
UVMR-cML	−0.03	−0.30
(SD)	(0.09)	(0.30)
(Mean SE)	(0.06)	(0.05)
MVMR-Egger	−1.24	−1.11
(SD)	(0.06)	(0.02)
(Mean SE)	(0.07)	(0.03)
UVMR-Egger	−1.63	−1.63
(SD)	(0.09)	(0.09)
(Mean SE)	(0.12)	(0.12)
DHO	−1.66	−1.66
(SD)	(0.13)	(0.13)
(Mean SE)	(0.07)	(0.07)
SH	−2.21	−2.21
(SD)	(0.06)	(0.06)
(Mean SE)	(0.07)	(0.07)

Table G. Mean effect estimates and empirical type-I error rate with and without bias correction for some representative SNPs. Sample standard deviations (SD) and mean standard errors (Mean SE) are given in parenthesis. $p_2 = 2$.

True effects		Mean effect estimates							Type-I error rate						
		No	MVMR-cML	UVMR-cML	MVMR-Egger	UVMR-Egger	DHO	SH	No	MVMR-cML	UVMR-cML	MVMR-Egger	UVMR-Egger	DHO	SH
		−1.62	−0.05	−1.69	−0.05	0.36	0.4	1.07	1.00	0.03	1.00	0.03	0.19	0.36	0.97
0.00	(SD)	(0.15)	(0.19)	(0.25)	(0.19)	(0.23)	(0.26)	(0.28)	(0.00)	(0.17)	(0.00)	(0.18)	(0.39)	(0.48)	(0.16)
	(Mean SE)	(0.15)	(0.22)	(0.19)	(0.21)	(0.29)	(0.25)	(0.28)	NA	NA	NA	NA	NA	NA	NA
		1.4	−0.01	1.34	−0.01	−0.47	−0.5	−1.13	0.91	0.05	0.86	0.05	0.14	0.16	0.36
0.00	(SD)	(0.42)	(0.55)	(0.45)	(0.55)	(0.66)	(0.67)	(0.80)	(0.29)	(0.21)	(0.35)	(0.22)	(0.35)	(0.37)	(0.48)
	(Mean SE)	(0.43)	(0.55)	(0.44)	(0.53)	(0.61)	(0.61)	(0.71)	NA	NA	NA	NA	NA	NA	NA
		−1.06	0.02	−0.82	0.01	0.49	0.51	1.04	1.00	0.05	0.98	0.07	0.62	0.7	0.99
0.00	(SD)	(0.13)	(0.17)	(0.29)	(0.17)	(0.23)	(0.26)	(0.26)	(0.00)	(0.22)	(0.12)	(0.26)	(0.49)	(0.46)	(0.08)
	(Mean SE)	(0.13)	(0.18)	(0.14)	(0.17)	(0.21)	(0.2)	(0.23)	NA	NA	NA	NA	NA	NA	NA
		1.25	−0.02	1.17	−0.01	−0.44	−0.47	−1.04	1.00	0.06	1.00	0.07	0.36	0.43	0.89
0.00	(SD)	(0.19)	(0.24)	(0.22)	(0.25)	(0.3)	(0.31)	(0.35)	(0.00)	(0.25)	(0.00)	(0.26)	(0.48)	(0.50)	(0.31)
	(Mean SE)	(0.18)	(0.24)	(0.20)	(0.23)	(0.28)	(0.27)	(0.31)	NA	NA	NA	NA	NA	NA	NA

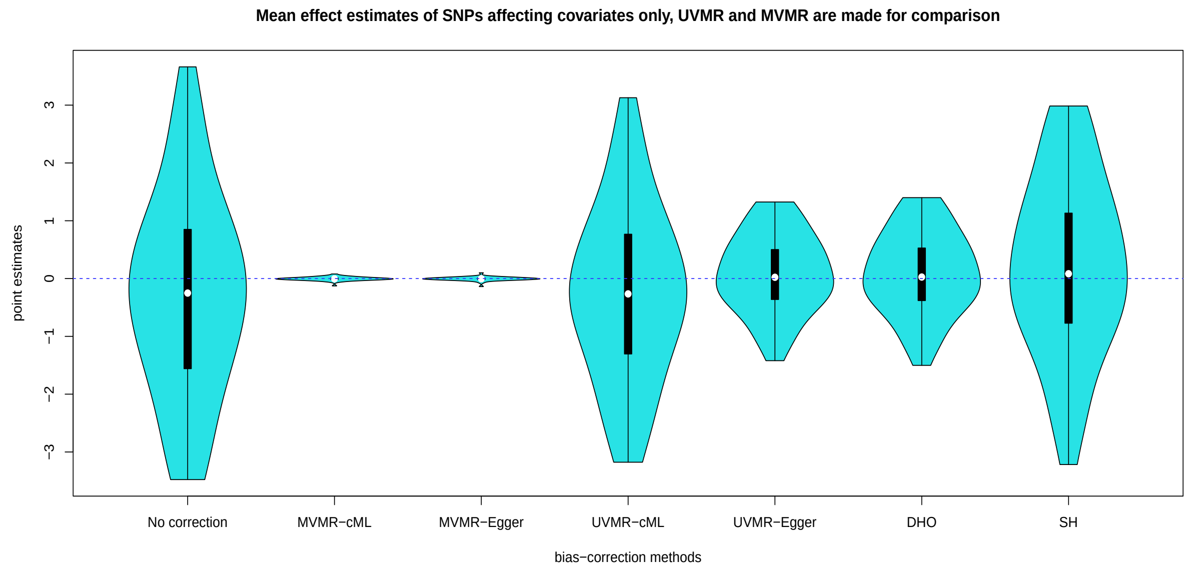

Fig E. Violin plot of mean effect estimates of 50 SNPs affecting covariates only. The true value 0 is highlighted by a dashed line.

E.2 Real GWAS data

We also provided a real GWAS data example to illustrate the insufficiency of UVMR, where two metabolomic principle components (PCs) were used as covariates in the GWAS of BMI to improve statistical power, as mentioned in the paper. MVMR methods were implemented in the same way in the paper. In the application of UVMR, the two components in $\boldsymbol{b}$ were estimated separately. The variance of the bias-corrected estimator $\hat{\beta}_{G_iY}$ is computed using equation (29), assuming $\hat{\boldsymbol{\Sigma}}_{\hat{\boldsymbol{b}}}$ to be diagonal.

Among the selected IVs, some were associated with both two metabolomic PCs with p-values $5e-10$. As discussed in the above simulation, they were not valid for UVMR. Hence, while MVMR methods produced similar results, the results of UVMR were not consistent. According to the simulation above, when two covariates were adjusted, MVMR had advantage in accounting more pleiotropy paths through covariates, and we believe the results of MVMR were more reliable.

Table H. Comparison of point estimates of $\boldsymbol{b}$ of UMVR and MVMR. Standard errors (SE) are in parenthesis.

$\hat{\boldsymbol{b}}$	MVMR-cML	UVMR-cML	MVMR-Egger	UVMR-Egger	DHO	SH
$\hat{b}_1$	0.24	0.24	0.22	0.22	0.24	0.32
(SE)	(0.03)	(0.03)	(0.06)	(0.06)	(0.03)	(0.11)
$\hat{b}_2$	-0.17	-0.41	-0.14	0.11	-0.15	-0.53
(SE)	(0.05)	(0.06)	(0.05)	(0.14)	(0.05)	(0.23)

Table I. Comparison of numbers of significant loci identified by UMVR and MVMR.

	No correction	MVMR-cML	UVMR-cML	MVMR-Egger	UVMR-Egger	DHO	SH
# of significant SNPs	483	192	166	193	101	177	74
# of significant loci	60	32	24	31	20	25	12
UKB validation	45	32	23	31	20	25	11
other validation	46	29	24	28	19	25	12

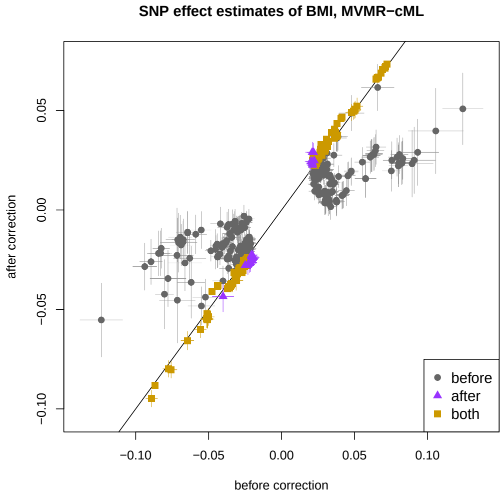
SNP effect estimates of BMI, MVMR−cML
after correction
before correction
before
after
both

(a)

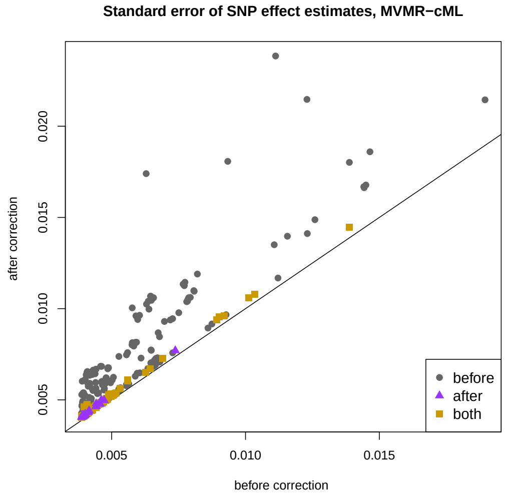
Standard error of SNP effect estimates, MVMR−cML
after correction
before correction
before
after
both

(b)

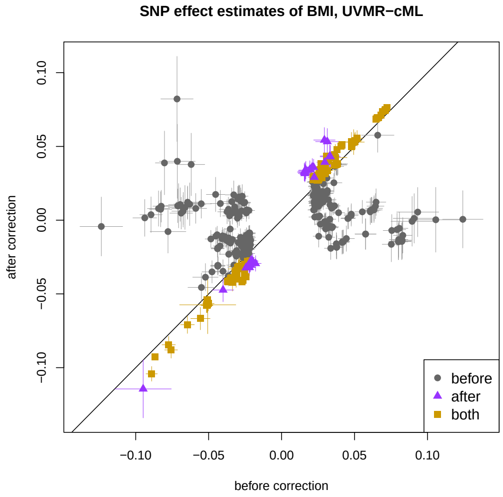
SNP effect estimates of BMI, UVMR−cML
after correction
before correction
before
after
both

(c)

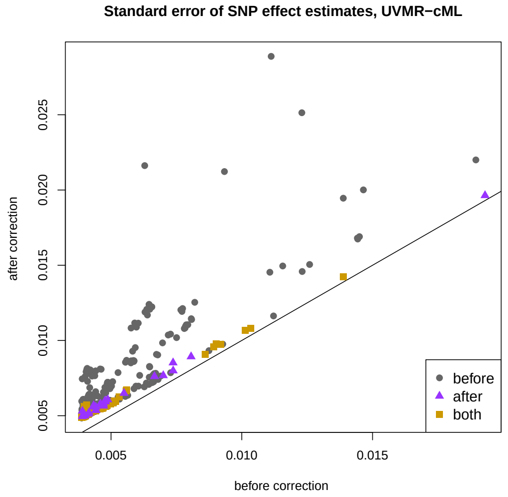
Standard error of SNP effect estimates, UVMR−cML
after correction
before correction
before
after
both

(d)

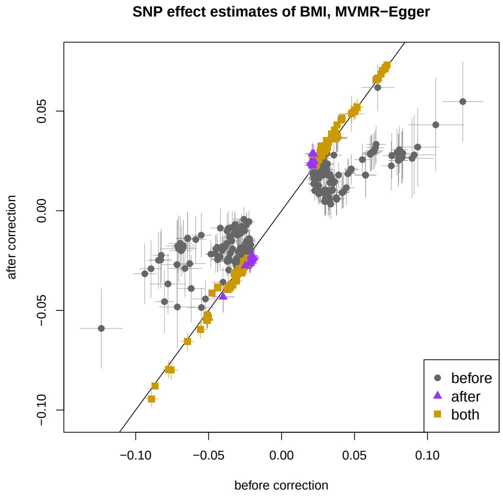
SNP effect estimates of BMI, MVMR−Egger
after correction
before correction
before
after
both

(e)

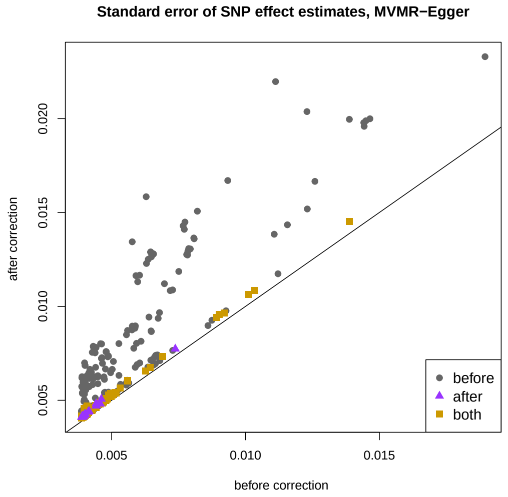
Standard error of SNP effect estimates, MVMR−Egger
after correction
before correction
before
after
both

(f)

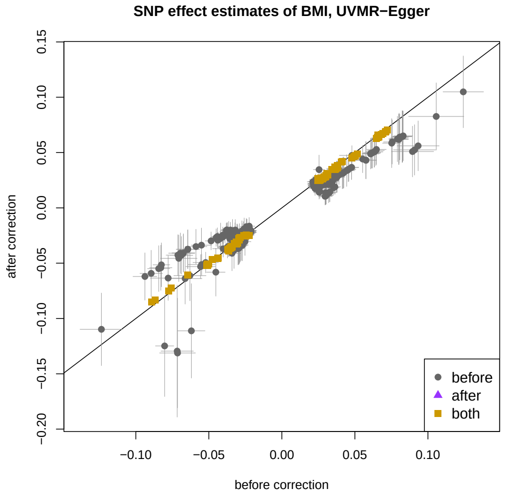

(g)

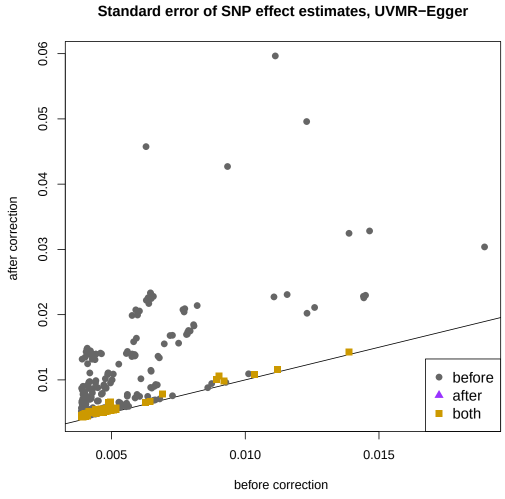

(h)

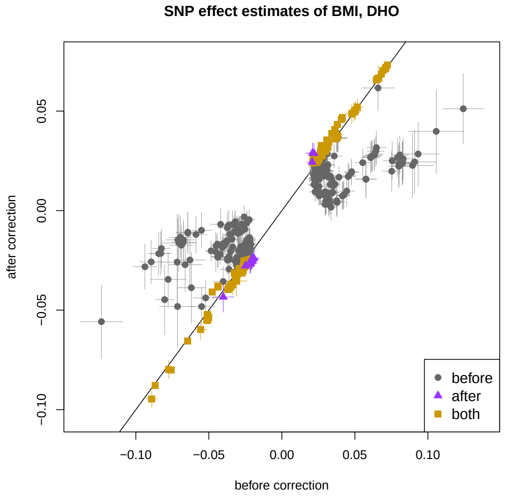

(i)

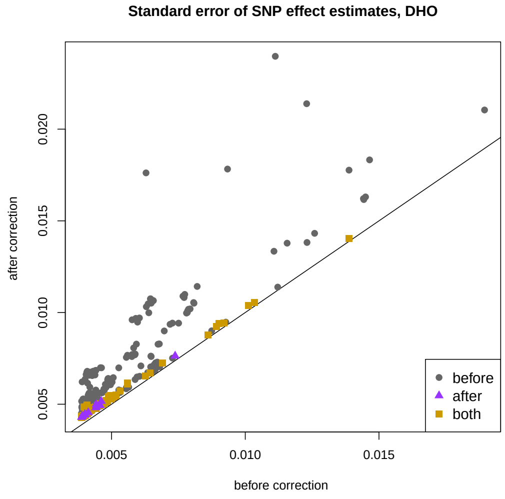

(j)

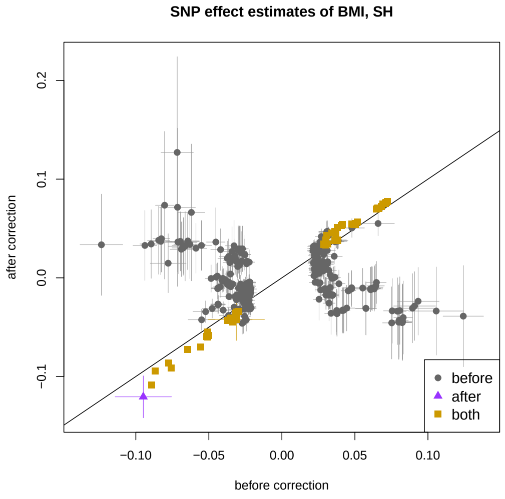

(k)

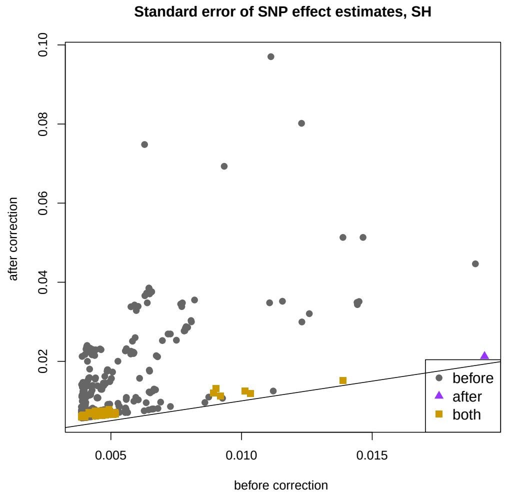

(l)

Fig F. Effect estimates (in M_1) of BMI before and after bias correction. UVMR and MVMR are made for comparison. Horizontal and vertical bars represent 1 SE of an estimate before and after correction respectively. SEs are given in the right column. 2 metabolomic PCs are used. In the legends, “before” refers to the SNPs that are significant only before applying bias correction, “after” refers to the SNPs that are significant only after bias correction, “both” refers to the SNPs that are significant both before and after bias correction.

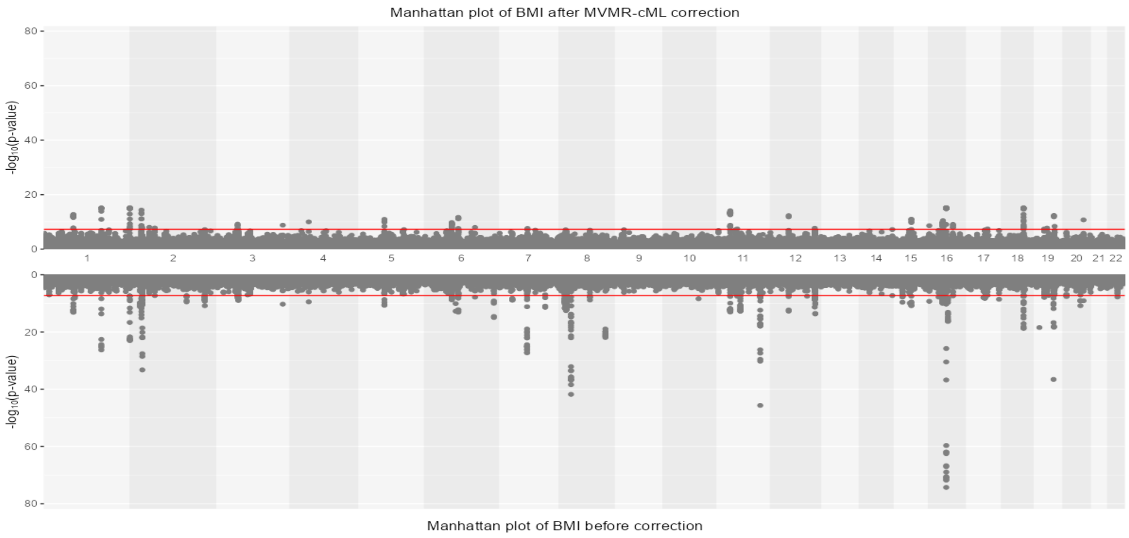
Manhattan plot of BMI after MVMR-cML correction
-log10(p-value)
1 2 3 4 5 6 7 8 9 10 11 12 13 14 15 16 17 18 19 20 21 22
-log10(p-value)
Manhattan plot of BMI before correction

(a)

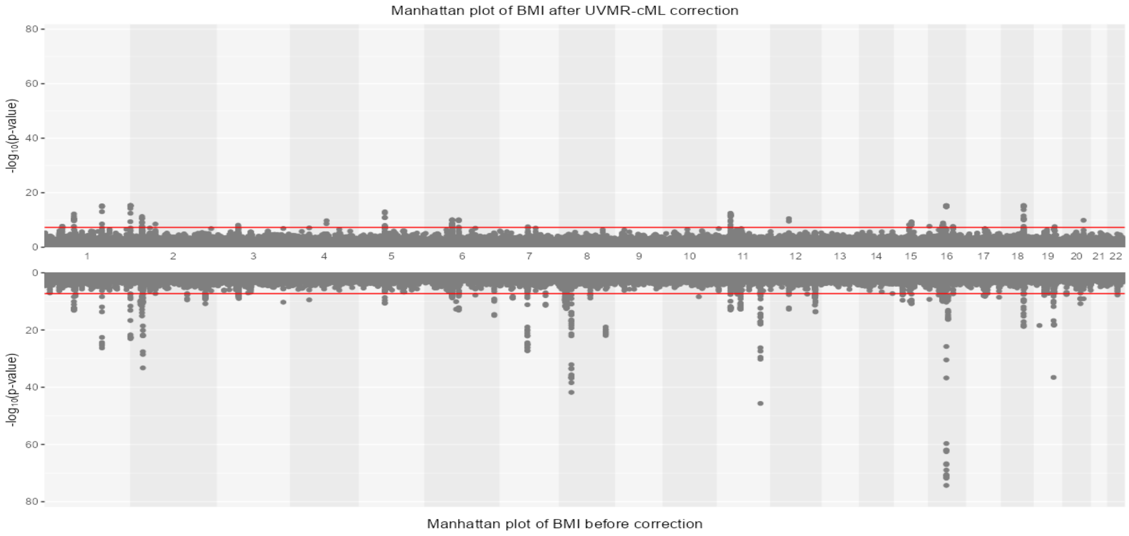
Manhattan plot of BMI after UVMR-cML correction
-log10(p-value)
1 2 3 4 5 6 7 8 9 10 11 12 13 14 15 16 17 18 19 20 21 22
-log10(p-value)
Manhattan plot of BMI before correction

(b)

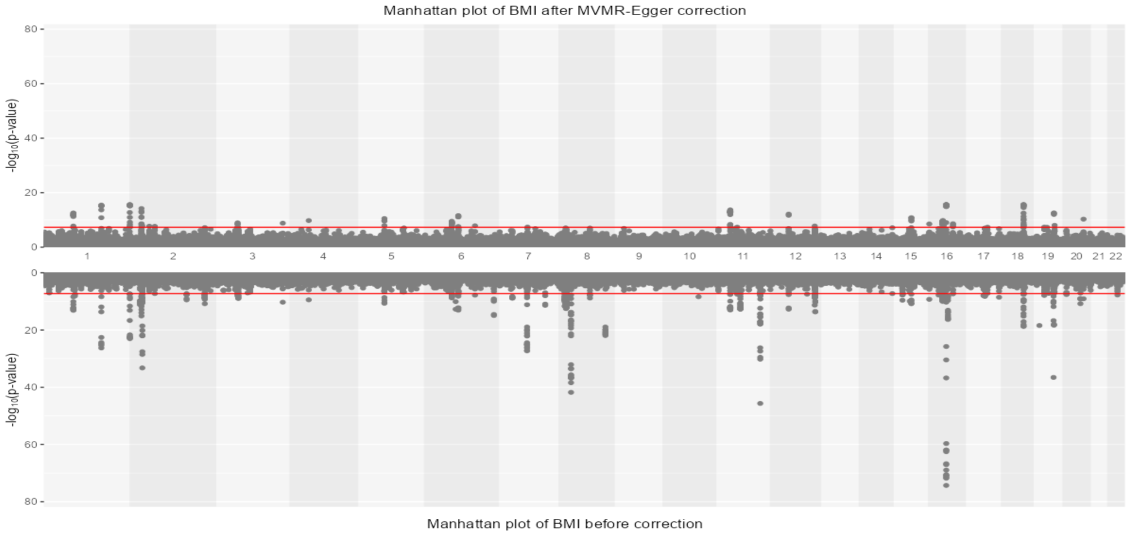
Manhattan plot of BMI after MVMR-Egger correction
-log10(p-value)
1 2 3 4 5 6 7 8 9 10 11 12 13 14 15 16 17 18 19 20 21 22
-log10(p-value)
Manhattan plot of BMI before correction

(c)

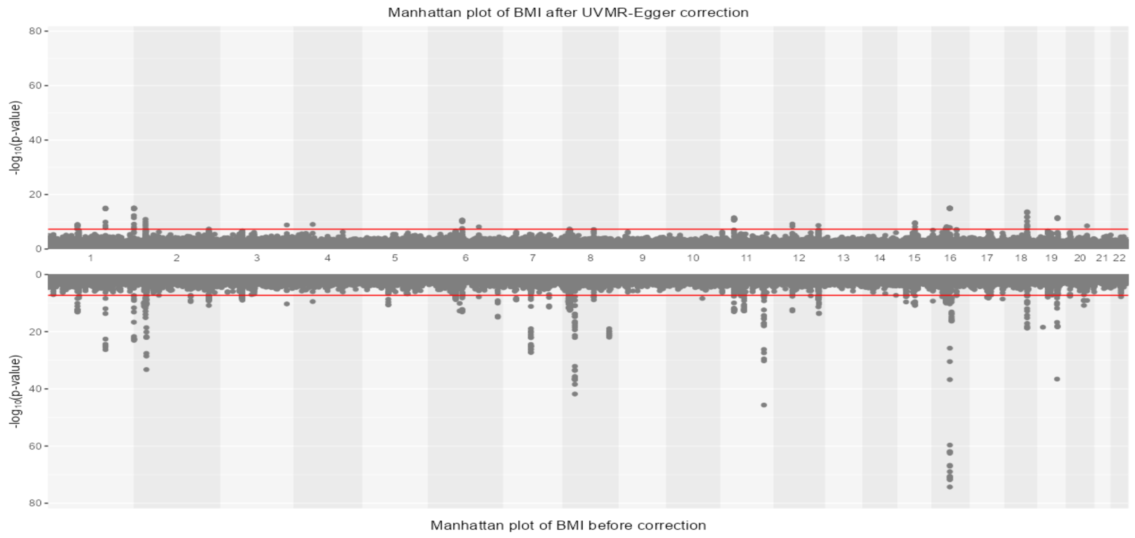

(d)

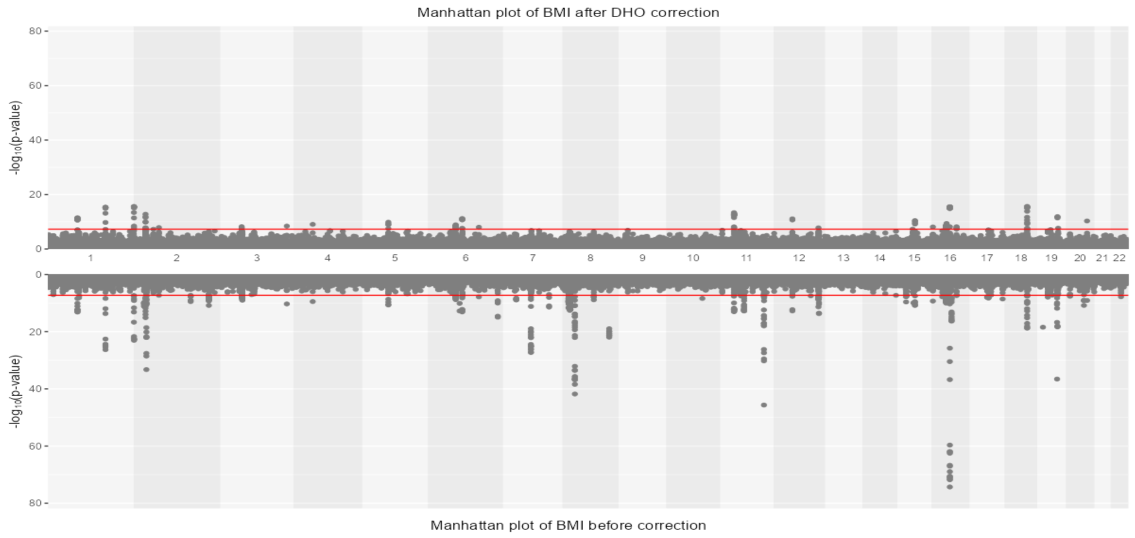

(e)

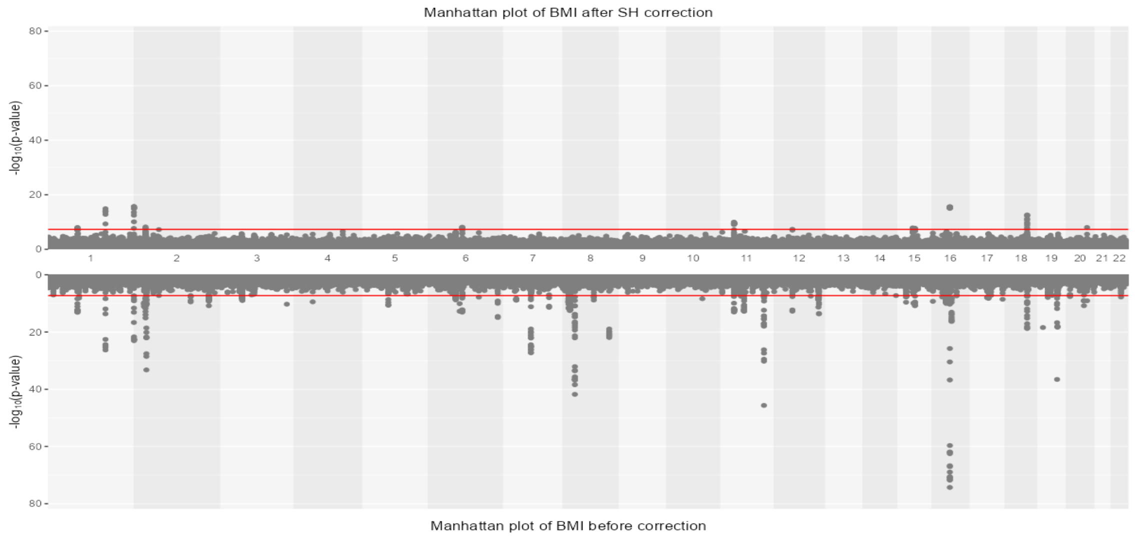

(f)

Fig G. Manhattan plot of BMI before (upper panel) and after (lower panel) applying bias correction on M_1. UVMR and MVMR are made for comparison. 2 metabolomic PC are adjusted.

F Simulation with (weakly) correlated SNPs and pleiotropy

F.1 Simulation set up

In order to generate weakly correlated SNPs, we first pruned the UK Biobank (UKB) genotype data with correlation coefficients 0.1, then we randomly sampled 1000 SNPs from chromosome 1. Among which 30 independent SNPs were used as instrumental variables (IVs). Among these SNPs, 50 affected $\boldsymbol{H}$ only, 50 affected Y only, 50 affected both $\boldsymbol{H}$ and Y. Others were null SNPs having effect 0 on both covariates and the outcome. Before drawing the SNPs, we partitioned chromosome 1 into 133 independent blocks [7]. The 150 effective SNPs were drawn from the first 10 blocks, and the 850 null SNPs were drawn from the last 10 blocks. Hence, the effective SNPs were independent of null SNPs. We also drawn 3000 independent null SNPs from other chromosomes, whose z-scores were used to estimate the correlations of summary statistics [2]. These 3000 null SNPs are not presented in simulation results. When estimating the correlation of summary statistics, we used a p-value 0.1 to select null z-cores for covariates and outcome, and approximately 2000 null z-scores were selected for each pair of summary data. All genotypes G_i were centered to achieve a sample mean of 0. In order to simulate correlated pleiotropy, for the 50 SNPs affecting both $\boldsymbol{H} = (H_1, \cdots, H_{p_2})^T$ and Y, their effects on Y (β_{G_iY}) and the first covariate H_1 ($\beta_{G_iH_1}$) were generated from a bivariate normal distribution with mean 0, variance 1, and the correlation ρ took different values of 0, 0.5, and -0.5. All other SNP effects, as well as the causal effects from covariates to outcome ($\boldsymbol{\beta}_{\boldsymbol{H}Y}$) were independently drawn from a standard normal distribution. All SNP effects were predetermined before initiating the simulation. The confounder U and error terms $E_{\boldsymbol{H}}$, E_Y were drawn from normal distributions with a mean of 0. U accounted for 40% of the unknown variance in both Y and the covariates within $\boldsymbol{H}$. The error terms contributed to 10% of the unknown variance in the covariates and outcome, respectively. The values of $\boldsymbol{H}$ and Y were subsequently determined based on the equations below, incorporating the effects, confounders, and error terms.

$$\boldsymbol{H} = \sum_{i=1}^{4000} \boldsymbol{\beta}_{G_i\boldsymbol{H}} G_i + \boldsymbol{\beta}_{U\boldsymbol{H}} U + E_{\boldsymbol{H}}, \tag{27}$$

$$Y = \sum_{i=1}^{4000} \beta_{G_iY} G_i + \beta_{UY} U + \boldsymbol{\beta}_{\boldsymbol{H}Y}^T \boldsymbol{H} + E_Y. \tag{28}$$

We conducted the simulation under two scenarios, respectively when 30% and 50% invalid IVs were used for estimation. The invalid IVs were taken from those having pleiotropy effects, while the valid IVs were taken from those affecting covariates only. The GWAS of Y was performed using $\boldsymbol{H}$ as covariates, while the GWAS of the elements in $\boldsymbol{H}$ only included the SNPs. This simulation followed a similar structure to those of Dudbridge et al. (2019) [1]. We summarized the result in the same way as the main paper. We only presented the simulation result of SNPs suffering from collider bias, i.e., those SNPs affecting $\boldsymbol{H}$.

In Table L and Table M, we also present the result when the equation:

$$\hat{\sigma}^2_{G_iY} = \hat{\boldsymbol{\beta}}^T_{G_i\boldsymbol{H}}\hat{\boldsymbol{\Sigma}}_{\hat{b}}\hat{\boldsymbol{\beta}}_{G_i\boldsymbol{H}} + \sum_{k=1}^{p_2}\hat{b}_k^2\hat{\sigma}^2_{G_iH_k} + \sum_{k=1}^{p_2}\hat{\sigma}^2_{\hat{b}_k}\hat{\sigma}^2_{G_iH_k} + \hat{\sigma}^{2C}_{G_iY} \tag{29}$$

is used to estimate the variance of $\hat{\beta}_{G_iY}$ after MVMR-cML-bias-correction.

F.2 30% invalid IVs

F.2.1 Tables of empirical type-I error rate and power

Table J. Empirical type-I error rate (for SNPs underlined) and power with and without bias correction in the presence of 30% invalid IVs. Sample standard deviations (SD) are given in parenthesis. $p_2 = 1$.

ρ	Dimension of $\boldsymbol{H}$	1							
	Bias correction	No	cML	Egger	IVW	Lasso	Median	DHO	SH
	Null SNPs	0.05	0.05	0.05	0.05	0.05	0.05	0.05	0.05
	(SD)	(0.01)	(0.01)	(0.01)	(0.01)	(0.01)	(0.01)	(0.01)	(0.01)
0	All SNPs affecting $\boldsymbol{H}$ but not Y	0.73	0.05	0.05	0.05	0.05	0.05	0.07	0.05
	(SD)	(0.04)	(0.03)	(0.03)	(0.03)	(0.03)	(0.03)	(0.05)	(0.03)
	All SNPs affecting Y only	0.34	0.26	0.26	0.26	0.26	0.26	0.26	0.26
	(SD)	(0.05)	(0.05)	(0.05)	(0.05)	(0.05)	(0.05)	(0.05)	(0.05)
	All SNPs affecting both $\boldsymbol{H}$ and Y	0.76	0.26	0.24	0.26	0.26	0.25	0.26	0.25
	(SD)	(0.04)	(0.05)	(0.04)	(0.05)	(0.05)	(0.05)	(0.05)	(0.05)
	SNP with greatest increase in power	0.23	0.87	0.87	0.87	0.87	0.87	0.87	0.87
	(SD)	(0.42)	(0.34)	(0.34)	(0.34)	(0.34)	(0.34)	(0.33)	(0.34)
	SNP with greatest decrease in power	1.00	0.05	0.01	0.03	0.03	0.02	0.03	0.05
	(SD)	(0.00)	(0.22)	(0.10)	(0.18)	(0.18)	(0.13)	(0.18)	(0.21)
	Null SNPs	0.05	0.05	0.05	0.05	0.05	0.05	0.05	0.05
	(SD)	(0.01)	(0.01)	(0.01)	(0.01)	(0.01)	(0.01)	(0.01)	(0.01)
0.5	All SNPs affecting $\boldsymbol{H}$ but not Y	0.70	0.07	0.08	0.09	0.09	0.08	0.05	0.05
	(SD)	(0.04)	(0.04)	(0.05)	(0.04)	(0.04)	(0.04)	(0.04)	(0.03)
	All SNPs affecting Y only	0.42	0.37	0.38	0.38	0.38	0.38	0.37	0.37
	(SD)	(0.04)	(0.05)	(0.05)	(0.05)	(0.05)	(0.05)	(0.05)	(0.05)
	All SNPs affecting both $\boldsymbol{H}$ and Y	0.67	0.25	0.22	0.24	0.24	0.23	0.23	0.24
	(SD)	(0.04)	(0.05)	(0.05)	(0.05)	(0.05)	(0.05)	(0.05)	(0.05)
	SNP with greatest increase in power	0.09	0.99	0.99	0.99	0.99	0.99	0.99	0.99
	(SD)	(0.28)	(0.09)	(0.11)	(0.10)	(0.10)	(0.10)	(0.07)	(0.08)
	SNP with greatest decrease in power	0.99	0.04	0.04	0.04	0.04	0.04	0.06	0.04
	(SD)	(0.08)	(0.20)	(0.18)	(0.20)	(0.20)	(0.19)	(0.23)	(0.20)
	Null SNPs	0.05	0.05	0.05	0.05	0.05	0.05	0.05	0.05
	(SD)	(0.01)	(0.01)	(0.01)	(0.01)	(0.01)	(0.01)	(0.01)	(0.01)
−0.5	All SNPs affecting $\boldsymbol{H}$ but not Y	0.70	0.06	0.05	0.05	0.05	0.04	0.05	0.05
	(SD)	(0.04)	(0.03)	(0.03)	(0.03)	(0.03)	(0.03)	(0.03)	(0.03)
	All SNPs affecting Y only	0.35	0.26	0.27	0.26	0.26	0.27	0.26	0.26
	(SD)	(0.05)	(0.05)	(0.05)	(0.05)	(0.05)	(0.05)	(0.05)	(0.05)
	All SNPs affecting both $\boldsymbol{H}$ and Y	0.77	0.22	0.24	0.23	0.23	0.23	0.23	0.22
	(SD)	(0.04)	(0.05)	(0.06)	(0.05)	(0.05)	(0.05)	(0.06)	(0.05)
	SNP with greatest increase in power	0.04	0.19	0.18	0.18	0.18	0.18	0.18	0.18
	(SD)	(0.20)	(0.39)	(0.38)	(0.39)	(0.39)	(0.38)	(0.38)	(0.39)
	SNP with greatest decrease in power	1.00	0.05	0.05	0.04	0.04	0.05	0.05	0.04
	(SD)	(0.00)	(0.21)	(0.21)	(0.20)	(0.20)	(0.21)	(0.21)	(0.20)

Table K. Empirical type-I error rate (for SNPs underlined) and power with and without bias correction in the presence of 30% invalid IVs. Sample standard deviations (SD) are given in parenthesis. $p_2 > 1$.

ρ	Dimension of $\boldsymbol{H}$	2						4					
	Bias correction	No	cML	Egger	IVW	Lasso	Median	No	cML	Egger	IVW	Lasso	Median
0	Null SNPs	0.05	0.06	0.06	0.06	0.06	0.06	0.05	0.06	0.10	0.10	0.10	0.10
	(SD)	(0.01)	(0.01)	(0.01)	(0.01)	(0.01)	(0.01)	(0.01)	(0.01)	(0.01)	(0.01)	(0.01)	(0.01)
	All SNPs affecting $\boldsymbol{H}$ but not Y	0.74	0.06	0.07	0.09	0.09	0.06	0.71	0.08	0.17	0.12	0.12	0.09
	(SD)	(0.03)	(0.04)	(0.04)	(0.04)	(0.04)	(0.04)	(0.04)	(0.04)	(0.07)	(0.04)	(0.04)	(0.04)
	All SNPs affecting Y only	0.65	0.54	0.56	0.55	0.55	0.56	0.77	0.70	0.72	0.73	0.73	0.73
	(SD)	(0.04)	(0.05)	(0.05)	(0.05)	(0.05)	(0.05)	(0.03)	(0.04)	(0.04)	(0.04)	(0.04)	(0.04)
	All SNPs affecting both $\boldsymbol{H}$ and Y	0.69	0.52	0.54	0.53	0.53	0.52	0.82	0.66	0.63	0.66	0.66	0.62
	(SD)	(0.04)	(0.05)	(0.05)	(0.05)	(0.05)	(0.05)	(0.03)	(0.05)	(0.04)	(0.04)	(0.04)	(0.05)
	SNP with greatest increase in power	0.05	0.98	0.99	0.98	0.98	0.98	0.28	0.95	0.99	0.98	0.98	0.97
	(SD)	(0.22)	(0.13)	(0.09)	(0.13)	(0.13)	(0.14)	(0.45)	(0.21)	(0.11)	(0.13)	(0.13)	(0.16)
	SNP with greatest decrease in power	1.00	0.05	0.04	0.06	0.06	0.04	1.00	0.06	0.07	0.09	0.09	0.03
	(SD)	(0.00)	(0.23)	(0.19)	(0.24)	(0.24)	(0.20)	(0.00)	(0.24)	(0.26)	(0.28)	(0.28)	(0.18)
0.5	Null SNPs	0.05	0.06	0.07	0.07	0.07	0.07	0.05	0.06	0.10	0.10	0.10	0.10
	(SD)	(0.01)	(0.01)	(0.01)	(0.01)	(0.01)	(0.01)	(0.01)	(0.01)	(0.01)	(0.01)	(0.01)	(0.01)
	All SNPs affecting $\boldsymbol{H}$ but not Y	0.75	0.08	0.06	0.09	0.09	0.06	0.76	0.09	0.18	0.21	0.21	0.10
	(SD)	(0.03)	(0.05)	(0.04)	(0.04)	(0.04)	(0.03)	(0.04)	(0.05)	(0.05)	(0.06)	(0.06)	(0.04)
	All SNPs affecting Y only	0.62	0.51	0.53	0.53	0.53	0.53	0.75	0.67	0.69	0.70	0.70	0.70
	(SD)	(0.05)	(0.05)	(0.05)	(0.05)	(0.05)	(0.05)	(0.04)	(0.04)	(0.04)	(0.04)	(0.04)	(0.04)
	All SNPs affecting both $\boldsymbol{H}$ and Y	0.67	0.39	0.40	0.41	0.41	0.39	0.71	0.53	0.56	0.57	0.57	0.54
	(SD)	(0.04)	(0.05)	(0.05)	(0.05)	(0.05)	(0.05)	(0.04)	(0.05)	(0.05)	(0.05)	(0.05)	(0.05)
	SNP with greatest increase in power	0.19	0.99	1.00	0.99	0.99	0.99	0.06	0.97	0.90	0.85	0.85	0.92
	(SD)	(0.39)	(0.12)	(0.06)	(0.09)	(0.09)	(0.08)	(0.24)	(0.17)	(0.30)	(0.36)	(0.36)	(0.28)
	SNP with greatest decrease in power	1.00	0.08	0.04	0.04	0.04	0.02	1.00	0.06	0.04	0.08	0.08	0.07
	(SD)	(0.00)	(0.26)	(0.20)	(0.19)	(0.19)	(0.15)	(0.00)	(0.24)	(0.18)	(0.27)	(0.27)	(0.26)
−0.5	Null SNPs	0.05	0.06	0.06	0.07	0.07	0.07	0.05	0.06	0.10	0.10	0.10	0.10
	(SD)	(0.01)	(0.01)	(0.01)	(0.01)	(0.01)	(0.01)	(0.01)	(0.01)	(0.01)	(0.01)	(0.01)	(0.01)
	All SNPs affecting $\boldsymbol{H}$ but not Y	0.78	0.07	0.06	0.12	0.12	0.07	0.76	0.08	0.35	0.34	0.34	0.25
	(SD)	(0.04)	(0.04)	(0.04)	(0.05)	(0.05)	(0.04)	(0.04)	(0.04)	(0.05)	(0.05)	(0.05)	(0.08)
	All SNPs affecting Y only	0.54	0.42	0.44	0.43	0.43	0.44	0.70	0.65	0.66	0.66	0.66	0.66
	(SD)	(0.05)	(0.05)	(0.05)	(0.05)	(0.05)	(0.05)	(0.04)	(0.04)	(0.04)	(0.04)	(0.04)	(0.04)
	All SNPs affecting both $\boldsymbol{H}$ and Y	0.73	0.45	0.47	0.45	0.45	0.45	0.69	0.54	0.58	0.59	0.59	0.51
	(SD)	(0.04)	(0.05)	(0.06)	(0.05)	(0.05)	(0.05)	(0.04)	(0.05)	(0.05)	(0.05)	(0.05)	(0.05)
	SNP with greatest increase in power	0.10	1.00	1.00	1.00	1.00	1.00	0.20	0.92	0.99	1.00	1.00	0.99
	(SD)	(0.30)	(0.00)	(0.00)	(0.00)	(0.00)	(0.00)	(0.40)	(0.27)	(0.08)	(0.06)	(0.06)	(0.08)
	SNP with greatest decrease in power	1.00	0.07	0.07	0.12	0.12	0.04	1.00	0.07	0.10	0.10	0.10	0.05
	(SD)	(0.00)	(0.26)	(0.26)	(0.32)	(0.32)	(0.18)	(0.00)	(0.25)	(0.30)	(0.30)	(0.30)	(0.23)

Table L. Empirical type-I error rate (for SNPs underlined) and power with and without bias correction in the presence of 30% invalid IVs. Sample standard deviations (SD) are given in parenthesis. Equation (29) is used for the variance estimate of MVMR-cML. $p_2 = 1$.

ρ	Dimension of $\boldsymbol{H}$	1							
	Bias correction	No	cML	Egger	IVW	Lasso	Median	DHO	SH
0	Null SNPs	0.05	0.05	0.05	0.05	0.05	0.05	0.05	0.05
	(SD)	(0.01)	(0.01)	(0.01)	(0.01)	(0.01)	(0.01)	(0.01)	(0.01)
	All SNPs affecting $\boldsymbol{H}$ but not Y	0.73	0.05	0.05	0.05	0.05	0.05	0.07	0.05
	(SD)	(0.04)	(0.03)	(0.03)	(0.03)	(0.03)	(0.03)	(0.05)	(0.03)
	All SNPs affecting Y only	0.34	0.26	0.26	0.26	0.26	0.26	0.26	0.26
	(SD)	(0.05)	(0.05)	(0.05)	(0.05)	(0.05)	(0.05)	(0.05)	(0.05)
	All SNPs affecting both $\boldsymbol{H}$ and Y	0.76	0.25	0.24	0.26	0.26	0.25	0.26	0.25
	(SD)	(0.04)	(0.05)	(0.04)	(0.05)	(0.05)	(0.05)	(0.05)	(0.05)
	SNP with greatest increase in power	0.23	0.87	0.87	0.87	0.87	0.87	0.87	0.87
	(SD)	(0.42)	(0.34)	(0.34)	(0.34)	(0.34)	(0.34)	(0.33)	(0.34)
	SNP with greatest decrease in power	1.00	0.03	0.01	0.03	0.03	0.02	0.03	0.05
	(SD)	(0.00)	(0.17)	(0.10)	(0.18)	(0.18)	(0.13)	(0.18)	(0.21)
0.5	Null SNPs	0.05	0.05	0.05	0.05	0.05	0.05	0.05	0.05
	(SD)	(0.01)	(0.01)	(0.01)	(0.01)	(0.01)	(0.01)	(0.01)	(0.01)
	All SNPs affecting $\boldsymbol{H}$ but not Y	0.70	0.07	0.08	0.09	0.09	0.08	0.05	0.05
	(SD)	(0.04)	(0.04)	(0.05)	(0.04)	(0.04)	(0.04)	(0.04)	(0.03)
	All SNPs affecting Y only	0.42	0.37	0.38	0.38	0.38	0.38	0.37	0.37
	(SD)	(0.04)	(0.05)	(0.05)	(0.05)	(0.05)	(0.05)	(0.05)	(0.05)
	All SNPs affecting both $\boldsymbol{H}$ and Y	0.67	0.25	0.22	0.24	0.24	0.23	0.23	0.24
	(SD)	(0.04)	(0.05)	(0.05)	(0.05)	(0.05)	(0.05)	(0.05)	(0.05)
	SNP with greatest increase in power	0.09	0.99	0.99	0.99	0.99	0.99	0.99	0.99
	(SD)	(0.28)	(0.09)	(0.11)	(0.10)	(0.10)	(0.10)	(0.07)	(0.08)
	SNP with greatest decrease in power	0.99	0.04	0.04	0.04	0.04	0.04	0.06	0.04
	(SD)	(0.08)	(0.21)	(0.18)	(0.20)	(0.20)	(0.19)	(0.23)	(0.20)
−0.5	Null SNPs	0.05	0.05	0.05	0.05	0.05	0.05	0.05	0.05
	(SD)	(0.01)	(0.01)	(0.01)	(0.01)	(0.01)	(0.01)	(0.01)	(0.01)
	All SNPs affecting $\boldsymbol{H}$ but not Y	0.70	0.05	0.05	0.05	0.05	0.04	0.05	0.05
	(SD)	(0.04)	(0.03)	(0.03)	(0.03)	(0.03)	(0.03)	(0.03)	(0.03)
	All SNPs affecting Y only	0.35	0.26	0.27	0.26	0.26	0.27	0.26	0.26
	(SD)	(0.05)	(0.05)	(0.05)	(0.05)	(0.05)	(0.05)	(0.05)	(0.05)
	All SNPs affecting both $\boldsymbol{H}$ and Y	0.77	0.22	0.24	0.23	0.23	0.23	0.23	0.22
	(SD)	(0.04)	(0.05)	(0.06)	(0.05)	(0.05)	(0.05)	(0.06)	(0.05)
	SNP with greatest increase in power	0.04	0.18	0.18	0.18	0.18	0.18	0.18	0.18
	(SD)	(0.20)	(0.39)	(0.38)	(0.39)	(0.39)	(0.38)	(0.38)	(0.39)
	SNP with greatest decrease in power	1.00	0.05	0.05	0.04	0.04	0.05	0.05	0.04
	(SD)	(0.00)	(0.21)	(0.21)	(0.20)	(0.20)	(0.21)	(0.21)	(0.20)

Table M. Empirical type-I error rate (for SNPs underlined) and power with and without bias correction in the presence of 30% invalid IVs. Sample standard deviations (SD) are given in parenthesis. Equation (29) is used for the variance estimate of MVMR-cML. $p_2 > 1$.

ρ	Dimension of $\boldsymbol{H}$	2						4					
	Bias correction	No	cML	Egger	IVW	Lasso	Median	No	cML	Egger	IVW	Lasso	Median
	Null SNPs	0.05	0.06	0.06	0.06	0.06	0.06	0.05	0.10	0.10	0.10	0.10	0.10
	(SD)	(0.01)	(0.01)	(0.01)	(0.01)	(0.01)	(0.01)	(0.01)	(0.01)	(0.01)	(0.01)	(0.01)	(0.01)
0	All SNPs affecting $\boldsymbol{H}$ but not Y	0.74	0.07	0.07	0.09	0.09	0.06	0.71	0.10	0.17	0.12	0.12	0.09
	(SD)	(0.03)	(0.04)	(0.04)	(0.04)	(0.04)	(0.04)	(0.04)	(0.04)	(0.07)	(0.04)	(0.04)	(0.04)
	All SNPs affecting Y only	0.65	0.56	0.56	0.55	0.55	0.56	0.77	0.73	0.72	0.73	0.73	0.73
	(SD)	(0.04)	(0.05)	(0.05)	(0.05)	(0.05)	(0.05)	(0.03)	(0.04)	(0.04)	(0.04)	(0.04)	(0.04)
	All SNPs affecting both $\boldsymbol{H}$ and Y	0.69	0.54	0.54	0.53	0.53	0.52	0.82	0.69	0.63	0.66	0.66	0.62
	(SD)	(0.04)	(0.05)	(0.05)	(0.05)	(0.05)	(0.05)	(0.03)	(0.05)	(0.04)	(0.04)	(0.04)	(0.05)
	SNP with greatest increase in power	0.05	0.98	0.99	0.98	0.98	0.98	0.28	0.96	0.99	0.98	0.98	0.97
	(SD)	(0.22)	(0.13)	(0.09)	(0.13)	(0.13)	(0.14)	(0.45)	(0.19)	(0.11)	(0.13)	(0.13)	(0.16)
	SNP with greatest decrease in power	1.00	0.06	0.04	0.06	0.06	0.04	1.00	0.09	0.07	0.09	0.09	0.03
	(SD)	(0.00)	(0.24)	(0.19)	(0.24)	(0.24)	(0.20)	(0.00)	(0.28)	(0.26)	(0.28)	(0.28)	(0.18)
	Null SNPs	0.05	0.07	0.07	0.07	0.07	0.07	0.05	0.09	0.10	0.10	0.10	0.10
0.5	(SD)	(0.01)	(0.01)	(0.01)	(0.01)	(0.01)	(0.01)	(0.01)	(0.01)	(0.01)	(0.01)	(0.01)	(0.01)
	All SNPs affecting $\boldsymbol{H}$ but not Y	0.75	0.08	0.06	0.09	0.09	0.06	0.76	0.12	0.18	0.21	0.21	0.10
	(SD)	(0.03)	(0.05)	(0.04)	(0.04)	(0.04)	(0.03)	(0.04)	(0.05)	(0.05)	(0.06)	(0.06)	(0.04)
	All SNPs affecting Y only	0.62	0.53	0.53	0.53	0.53	0.53	0.75	0.70	0.69	0.70	0.70	0.70
	(SD)	(0.05)	(0.05)	(0.05)	(0.05)	(0.05)	(0.05)	(0.04)	(0.04)	(0.04)	(0.04)	(0.04)	(0.04)
	All SNPs affecting both $\boldsymbol{H}$ and Y	0.67	0.41	0.40	0.41	0.41	0.39	0.71	0.57	0.56	0.57	0.57	0.54
	(SD)	(0.04)	(0.05)	(0.05)	(0.05)	(0.05)	(0.05)	(0.04)	(0.05)	(0.05)	(0.05)	(0.05)	(0.05)
	SNP with greatest increase in power	0.19	0.99	1.00	0.99	0.99	0.99	0.06	0.97	0.90	0.85	0.85	0.92
	(SD)	(0.39)	(0.10)	(0.06)	(0.09)	(0.09)	(0.08)	(0.24)	(0.16)	(0.30)	(0.36)	(0.36)	(0.28)
	SNP with greatest decrease in power	1.00	0.06	0.04	0.04	0.04	0.02	1.00	0.09	0.04	0.08	0.08	0.07
	(SD)	(0.00)	(0.24)	(0.20)	(0.19)	(0.19)	(0.15)	(0.00)	(0.29)	(0.18)	(0.27)	(0.27)	(0.26)
	Null SNPs	0.05	0.07	0.06	0.07	0.07	0.07	0.05	0.09	0.10	0.10	0.10	0.10
	(SD)	(0.01)	(0.01)	(0.01)	(0.01)	(0.01)	(0.01)	(0.01)	(0.01)	(0.01)	(0.01)	(0.01)	(0.01)
-0.5	All SNPs affecting $\boldsymbol{H}$ but not Y	0.78	0.08	0.06	0.12	0.12	0.07	0.76	0.10	0.35	0.34	0.34	0.25
	(SD)	(0.04)	(0.04)	(0.04)	(0.05)	(0.05)	(0.04)	(0.04)	(0.04)	(0.05)	(0.05)	(0.05)	(0.08)
	All SNPs affecting Y only	0.54	0.44	0.44	0.43	0.43	0.44	0.70	0.68	0.66	0.66	0.66	0.66
	(SD)	(0.05)	(0.05)	(0.05)	(0.05)	(0.05)	(0.05)	(0.04)	(0.04)	(0.04)	(0.04)	(0.04)	(0.04)
	All SNPs affecting both $\boldsymbol{H}$ and Y	0.73	0.46	0.47	0.45	0.45	0.45	0.69	0.58	0.58	0.59	0.59	0.51
	(SD)	(0.04)	(0.05)	(0.06)	(0.05)	(0.05)	(0.05)	(0.04)	(0.05)	(0.05)	(0.05)	(0.05)	(0.05)
	SNP with greatest increase in power	0.10	1.00	1.00	1.00	1.00	1.00	0.20	0.94	0.99	1.00	1.00	0.99
	(SD)	(0.30)	(0.00)	(0.00)	(0.00)	(0.00)	(0.00)	(0.40)	(0.23)	(0.08)	(0.06)	(0.06)	(0.08)
	SNP with greatest decrease in power	1.00	0.09	0.07	0.12	0.12	0.04	1.00	0.11	0.10	0.10	0.10	0.05
	(SD)	(0.00)	(0.29)	(0.26)	(0.32)	(0.32)	(0.18)	(0.00)	(0.32)	(0.30)	(0.30)	(0.30)	(0.23)

F.2.2 Estimates of slope vector

Table N. True values and mean estimates of $\boldsymbol{b}$ with 30% invalid IVs. Sample standard deviations (SD), mean standard errors (Mean SE) are given in parenthesis. $p_2 = 1$.

ρ		True value	MVMR-cML	MVMR-Egger	MVMR-IVW	MVMR-Lasso	MVMR-Median	DHO	SH
		−3.26	−3.36	−3.41	−3.32	−3.32	−3.36	−3.54	−3.33
0	(SD)	(0.00)	(0.10)	(0.16)	(0.09)	(0.09)	(0.10)	(0.23)	(0.11)
	(Mean SE)	(0.00)	(0.09)	(0.21)	(0.09)	(0.09)	(0.13)	(0.17)	(0.12)
		−3.34	−3.26	−3.15	−3.17	−3.17	−3.20	−3.38	−3.34
0.5	(SD)	(0.00)	(0.13)	(0.19)	(0.11)	(0.11)	(0.12)	(0.22)	(0.14)
	(Mean SE)	(0.00)	(0.11)	(0.23)	(0.11)	(0.11)	(0.18)	(0.25)	(0.21)
		−3.18	−3.21	−3.02	−3.16	−3.16	−3.12	−3.14	−3.19
−0.5	(SD)	(0.00)	(0.11)	(0.15)	(0.10)	(0.10)	(0.11)	(0.17)	(0.12)
	(Mean SE)	(0.00)	(0.10)	(0.18)	(0.10)	(0.10)	(0.14)	(0.18)	(0.15)

Table O. True values and mean estimates of $\boldsymbol{b}$ with 30% invalid IVs. Sample standard deviations (SD), mean standard errors (Mean SE) are given in parenthesis. $p_2 > 1$.

ρ	Dimension of $\boldsymbol{b}$	2		4			
	$\boldsymbol{b}$	b_1	b_2	b_1	b_2	b_3	b_4
0	True value	−1.19	−1.22	−0.48	−0.64	−0.46	−0.39
	MVMR-cML	−1.17	−1.26	−0.51	−0.83	−0.41	−0.37
	(SD)	(0.07)	(0.07)	(0.06)	(0.06)	(0.04)	(0.07)
	(Mean SE)	(0.05)	(0.06)	(0.04)	(0.05)	(0.04)	(0.03)
	MVMR-Egger	−1.05	−1.25	−0.69	−0.74	−0.36	−0.42
	(SD)	(0.10)	(0.05)	(0.07)	(0.05)	(0.04)	(0.03)
	(Mean SE)	(0.11)	(0.06)	(0.08)	(0.05)	(0.05)	(0.03)
	MVMR-IVW	−1.26	−1.24	−0.56	−0.72	−0.41	−0.42
	(SD)	(0.05)	(0.05)	(0.04)	(0.05)	(0.03)	(0.03)
	(Mean SE)	(0.05)	(0.06)	(0.03)	(0.05)	(0.04)	(0.03)
	MVMR-Lasso	−1.26	−1.24	−0.56	−0.72	−0.41	−0.42
	(SD)	(0.05)	(0.05)	(0.04)	(0.05)	(0.03)	(0.03)
	(Mean SE)	(0.05)	(0.06)	(0.03)	(0.05)	(0.04)	(0.03)
	MVMR-Median	−1.21	−1.22	−0.56	−0.73	−0.42	−0.43
	(SD)	(0.06)	(0.06)	(0.04)	(0.05)	(0.04)	(0.04)
	(Mean SE)	(0.08)	(0.09)	(0.06)	(0.08)	(0.05)	(0.06)
0.5	True value	−1.22	−1.18	−0.53	−0.48	−0.44	−0.54
	MVMR-cML	−1.28	−1.18	−0.74	−0.44	−0.61	−0.30
	(SD)	(0.11)	(0.05)	(0.05)	(0.07)	(0.06)	(0.07)
	(Mean SE)	(0.05)	(0.04)	(0.04)	(0.04)	(0.04)	(0.04)
	MVMR-Egger	−1.33	−1.14	−0.69	−0.48	−0.64	−0.49
	(SD)	(0.07)	(0.04)	(0.05)	(0.04)	(0.04)	(0.04)
	(Mean SE)	(0.08)	(0.04)	(0.06)	(0.04)	(0.04)	(0.04)
	MVMR-IVW	−1.24	−1.15	−0.64	−0.50	−0.62	−0.47
	(SD)	(0.05)	(0.04)	(0.04)	(0.04)	(0.04)	(0.04)
	(Mean SE)	(0.05)	(0.04)	(0.04)	(0.04)	(0.04)	(0.04)
	MVMR-Lasso	−1.24	−1.15	−0.64	−0.50	−0.62	−0.47
	(SD)	(0.05)	(0.04)	(0.04)	(0.04)	(0.04)	(0.04)
	(Mean SE)	(0.05)	(0.04)	(0.04)	(0.04)	(0.04)	(0.04)
	MVMR-Median	−1.29	−1.16	−0.70	−0.51	−0.60	−0.38
	(SD)	(0.06)	(0.04)	(0.05)	(0.05)	(0.05)	(0.05)
	(Mean SE)	(0.09)	(0.06)	(0.06)	(0.06)	(0.06)	(0.07)
−0.5	True value	−1.16	−1.18	−0.53	−0.56	−0.39	−0.40
	MVMR-cML	−1.13	−1.30	−0.55	−0.51	−0.54	−0.40
	(SD)	(0.08)	(0.08)	(0.06)	(0.07)	(0.04)	(0.06)
	(Mean SE)	(0.06)	(0.05)	(0.05)	(0.05)	(0.04)	(0.05)
	MVMR-Egger	−1.03	−1.34	−0.88	−0.92	−0.34	−0.40
	(SD)	(0.11)	(0.04)	(0.07)	(0.04)	(0.03)	(0.05)
	(Mean SE)	(0.14)	(0.04)	(0.09)	(0.04)	(0.04)	(0.06)
	MVMR-IVW	−1.25	−1.35	−0.77	−0.92	−0.34	−0.43
	(SD)	(0.06)	(0.04)	(0.04)	(0.04)	(0.03)	(0.05)
	(Mean SE)	(0.06)	(0.04)	(0.04)	(0.04)	(0.04)	(0.05)
	MVMR-Lasso	−1.25	−1.35	−0.77	−0.92	−0.34	−0.43
	(SD)	(0.06)	(0.04)	(0.04)	(0.04)	(0.03)	(0.05)
	(Mean SE)	(0.06)	(0.04)	(0.04)	(0.04)	(0.04)	(0.05)
	MVMR-Median	−1.17	−1.35	−0.76	−0.93	−0.33	−0.42
	(SD)	(0.07)	(0.05)	(0.07)	(0.10)	(0.06)	(0.07)
	(Mean SE)	(0.09)	(0.07)	(0.10)	(0.14)	(0.08)	(0.08)

F.2.3 Effect estimates, empirical type-I error rate and power of representative SNPs

Table P. Mean effect estimates, empirical type-I error rate (for those SNPs with true effect 0 and underlined) and power with and without bias correction for some representative SNPs in the presence of 30% invalid IVs. Sample standard deviations (SD) and mean standard errors (Mean SE) are given in parenthesis. $p_2 = 1$.

ρ	True effects		Mean effect estimates								Type-I error rate or power							
			No	cML	Egger	IVW	Lasso	Median	DHO	SH	No	cML	Egger	IVW	Lasso	Median	DHO	SH
0	0.00		−8.96	0.19	0.31	0.07	0.07	0.18	0.67	0.10	1.00	0.03	0.02	0.04	0.04	0.03	0.09	0.01
		(SD)	(0.74)	(0.87)	(0.90)	(0.88)	(0.88)	(0.88)	(1.01)	(0.73)	(0.00)	(0.16)	(0.14)	(0.18)	(0.18)	(0.18)	(0.29)	(0.10)
		(Mean SE)	(0.76)	(1.02)	(1.10)	(0.95)	(0.95)	(0.99)	(1.05)	(0.97)	*NA*	*NA*	*NA*	*NA*	*NA*	*NA*	*NA*	*NA*
	0.00		−3.91	0.07	0.12	0.01	0.01	0.06	0.28	0.03	1.00	0.02	0.04	0.03	0.03	0.03	0.10	0.03
		(SD)	(0.33)	(0.38)	(0.42)	(0.38)	(0.38)	(0.39)	(0.47)	(0.38)	(0.00)	(0.15)	(0.18)	(0.17)	(0.17)	(0.17)	(0.30)	(0.18)
		(Mean SE)	(0.33)	(0.45)	(0.48)	(0.41)	(0.41)	(0.43)	(0.46)	(0.42)	*NA*	*NA*	*NA*	*NA*	*NA*	*NA*	*NA*	*NA*
	−0.40		−2.60	−1.06	−1.04	−1.08	−1.08	−1.06	−0.98	−1.08	0.90	0.16	0.15	0.16	0.16	0.16	0.14	0.17
		(SD)	(0.75)	(0.93)	(0.94)	(0.93)	(0.93)	(0.93)	(0.95)	(0.93)	(0.30)	(0.37)	(0.36)	(0.37)	(0.37)	(0.37)	(0.35)	(0.37)
		(Mean SE)	(0.82)	(1.01)	(1.02)	(1.00)	(1.00)	(1.01)	(1.03)	(1.01)	*NA*	*NA*	*NA*	*NA*	*NA*	*NA*	*NA*	*NA*
	0.31		1.56	−1.09	−1.12	−1.05	−1.05	−1.08	−1.23	−1.06	0.59	0.22	0.22	0.21	0.21	0.22	0.27	0.22
		(SD)	(0.71)	(0.89)	(0.90)	(0.89)	(0.89)	(0.89)	(0.92)	(0.89)	(0.49)	(0.42)	(0.42)	(0.41)	(0.41)	(0.41)	(0.44)	(0.41)
		(Mean SE)	(0.73)	(0.89)	(0.91)	(0.89)	(0.89)	(0.89)	(0.91)	(0.89)	*NA*	*NA*	*NA*	*NA*	*NA*	*NA*	*NA*	*NA*
0.5	0.00		3.85	0.17	0.31	0.28	0.28	0.24	0.04	0.09	1.00	0.05	0.06	0.06	0.06	0.05	0.03	0.03
		(SD)	(0.71)	(0.88)	(0.86)	(0.87)	(0.87)	(0.88)	(0.88)	(0.85)	(0.00)	(0.22)	(0.23)	(0.23)	(0.23)	(0.22)	(0.18)	(0.18)
		(Mean SE)	(0.74)	(0.88)	(0.92)	(0.89)	(0.89)	(0.91)	(0.95)	(0.93)	*NA*	*NA*	*NA*	*NA*	*NA*	*NA*	*NA*	*NA*
	0.00		−1.60	−0.12	−0.17	−0.17	−0.17	−0.15	−0.07	−0.09	1.00	0.06	0.09	0.08	0.08	0.07	0.07	0.06
		(SD)	(0.32)	(0.39)	(0.41)	(0.39)	(0.39)	(0.39)	(0.42)	(0.40)	(0.05)	(0.23)	(0.29)	(0.27)	(0.27)	(0.26)	(0.25)	(0.24)
		(Mean SE)	(0.32)	(0.40)	(0.40)	(0.39)	(0.39)	(0.39)	(0.41)	(0.40)	*NA*	*NA*	*NA*	*NA*	*NA*	*NA*	*NA*	*NA*
	0.18		−0.07	0.33	0.32	0.32	0.32	0.33	0.35	0.34	0.05	0.07	0.07	0.07	0.07	0.07	0.07	0.07
		(SD)	(0.80)	(1.01)	(1.00)	(1.00)	(1.00)	(1.00)	(1.02)	(1.02)	(0.22)	(0.25)	(0.25)	(0.25)	(0.25)	(0.25)	(0.26)	(0.25)
		(Mean SE)	(0.81)	(0.98)	(0.97)	(0.97)	(0.97)	(0.97)	(0.99)	(0.98)	*NA*	*NA*	*NA*	*NA*	*NA*	*NA*	*NA*	*NA*
	0.02		5.45	1.13	1.29	1.25	1.25	1.21	0.97	1.03	1.00	0.26	0.29	0.31	0.31	0.28	0.18	0.19
		(SD)	(0.69)	(0.88)	(0.89)	(0.86)	(0.86)	(0.87)	(0.92)	(0.88)	(0.00)	(0.44)	(0.45)	(0.46)	(0.46)	(0.45)	(0.38)	(0.39)
		(Mean SE)	(0.71)	(0.87)	(0.90)	(0.86)	(0.86)	(0.89)	(0.93)	(0.91)	*NA*	*NA*	*NA*	*NA*	*NA*	*NA*	*NA*	*NA*
-0.5	0.00		1.80	−0.15	−0.03	−0.12	−0.12	−0.09	−0.11	−0.14	0.60	0.05	0.04	0.05	0.05	0.04	0.04	0.04
		(SD)	(0.79)	(0.99)	(0.98)	(0.99)	(0.99)	(0.98)	(0.99)	(0.99)	(0.49)	(0.22)	(0.20)	(0.22)	(0.22)	(0.20)	(0.21)	(0.21)
		(Mean SE)	(0.83)	(1.00)	(0.99)	(1.00)	(1.00)	(0.99)	(1.00)	(1.00)	*NA*	*NA*	*NA*	*NA*	*NA*	*NA*	*NA*	*NA*
	0.00		−1.66	0.11	0.01	0.08	0.08	0.06	0.07	0.10	1.00	0.04	0.05	0.04	0.04	0.04	0.05	0.04
		(SD)	(0.34)	(0.42)	(0.43)	(0.42)	(0.42)	(0.42)	(0.44)	(0.42)	(0.03)	(0.20)	(0.23)	(0.20)	(0.20)	(0.20)	(0.22)	(0.21)
		(Mean SE)	(0.36)	(0.45)	(0.44)	(0.44)	(0.44)	(0.44)	(0.44)	(0.44)	*NA*	*NA*	*NA*	*NA*	*NA*	*NA*	*NA*	*NA*
	0.99		1.98	1.40	1.43	1.41	1.41	1.42	1.41	1.40	0.62	0.25	0.27	0.26	0.26	0.26	0.26	0.25
		(SD)	(0.87)	(1.09)	(1.07)	(1.09)	(1.09)	(1.08)	(1.09)	(1.09)	(0.49)	(0.43)	(0.44)	(0.44)	(0.44)	(0.44)	(0.44)	(0.43)
		(Mean SE)	(0.91)	(1.09)	(1.07)	(1.09)	(1.09)	(1.08)	(1.09)	(1.09)	*NA*	*NA*	*NA*	*NA*	*NA*	*NA*	*NA*	*NA*
	0.72		1.87	0.51	0.59	0.53	0.53	0.55	0.54	0.52	0.65	0.07	0.09	0.08	0.08	0.08	0.08	0.07
		(SD)	(0.75)	(0.93)	(0.92)	(0.93)	(0.93)	(0.92)	(0.93)	(0.93)	(0.48)	(0.26)	(0.28)	(0.27)	(0.27)	(0.27)	(0.27)	(0.26)
		(Mean SE)	(0.80)	(0.96)	(0.95)	(0.96)	(0.96)	(0.96)	(0.96)	(0.96)	*NA*	*NA*	*NA*	*NA*	*NA*	*NA*	*NA*	*NA*

Table Q. Mean effect estimates, empirical type-I error rate (for those SNPs with true effect 0 and underlined) and power with and without bias correction for some representative SNPs in the presence of 30% invalid IVs. Sample standard deviations (SD) and mean standard errors (Mean SE) are given in parenthesis. $p_2 = 2$.

ρ	True effects		Mean effect estimates						Type-I error rate or power					
			No	cML	Egger	IVW	Lasso	Median	No	cML	Egger	IVW	Lasso	Median
0	0.00		−3.54	0.09	−0.11	0.24	0.24	0.12	1.00	0.02	0.03	0.10	0.10	0.04
		(SD)	(0.21)	(0.27)	(0.27)	(0.27)	(0.27)	(0.28)	(0.00)	(0.14)	(0.16)	(0.31)	(0.31)	(0.20)
		(Mean SE)	(0.22)	(0.34)	(0.33)	(0.30)	(0.30)	(0.32)	*NA*	*NA*	*NA*	*NA*	*NA*	*NA*
	0.00		2.81	0.02	0.17	−0.08	−0.08	0.01	1.00	0.05	0.06	0.06	0.06	0.06
		(SD)	(0.38)	(0.50)	(0.49)	(0.50)	(0.50)	(0.50)	(0.00)	(0.22)	(0.23)	(0.23)	(0.23)	(0.23)
		(Mean SE)	(0.39)	(0.51)	(0.51)	(0.51)	(0.51)	(0.51)	*NA*	*NA*	*NA*	*NA*	*NA*	*NA*
	−0.58		−1.08	−0.51	−0.59	−0.43	−0.43	−0.47	1.00	0.79	0.88	0.68	0.68	0.73
		(SD)	(0.13)	(0.19)	(0.19)	(0.18)	(0.18)	(0.18)	(0.00)	(0.41)	(0.33)	(0.47)	(0.47)	(0.45)
		(Mean SE)	(0.13)	(0.18)	(0.18)	(0.18)	(0.18)	(0.18)	*NA*	*NA*	*NA*	*NA*	*NA*	*NA*
	−1.48		0.13	−1.17	−1.11	−1.21	−1.21	−1.17	0.09	1.00	0.99	1.00	1.00	1.00
		(SD)	(0.17)	(0.25)	(0.25)	(0.25)	(0.25)	(0.25)	(0.29)	(0.05)	(0.07)	(0.03)	(0.03)	(0.03)
		(Mean SE)	(0.18)	(0.24)	(0.24)	(0.24)	(0.24)	(0.24)	*NA*	*NA*	*NA*	*NA*	*NA*	*NA*
0.5	0.00		3.10	0.04	0.01	0.13	0.13	0.04	1.00	0.07	0.04	0.07	0.07	0.05
		(SD)	(0.25)	(0.36)	(0.35)	(0.34)	(0.34)	(0.35)	(0.00)	(0.25)	(0.21)	(0.25)	(0.25)	(0.21)
		(Mean SE)	(0.26)	(0.33)	(0.35)	(0.34)	(0.34)	(0.36)	*NA*	*NA*	*NA*	*NA*	*NA*	*NA*
	0.00		2.79	−0.09	−0.06	−0.02	−0.02	−0.07	1.00	0.05	0.05	0.05	0.05	0.05
		(SD)	(0.43)	(0.61)	(0.61)	(0.60)	(0.60)	(0.61)	(0.00)	(0.21)	(0.22)	(0.23)	(0.23)	(0.22)
		(Mean SE)	(0.47)	(0.62)	(0.61)	(0.60)	(0.60)	(0.61)	*NA*	*NA*	*NA*	*NA*	*NA*	*NA*
	0.47		0.30	0.43	0.27	0.45	0.45	0.38	0.43	0.39	0.21	0.47	0.47	0.21
		(SD)	(0.17)	(0.32)	(0.29)	(0.27)	(0.27)	(0.29)	(0.50)	(0.49)	(0.41)	(0.50)	(0.50)	(0.41)
		(Mean SE)	(0.17)	(0.25)	(0.27)	(0.24)	(0.24)	(0.30)	*NA*	*NA*	*NA*	*NA*	*NA*	*NA*
	−0.28		1.83	−0.29	−0.35	−0.22	−0.22	−0.30	1.00	0.19	0.23	0.14	0.14	0.17
		(SD)	(0.21)	(0.35)	(0.32)	(0.31)	(0.31)	(0.32)	(0.00)	(0.39)	(0.42)	(0.34)	(0.34)	(0.38)
		(Mean SE)	(0.22)	(0.30)	(0.30)	(0.29)	(0.29)	(0.31)	*NA*	*NA*	*NA*	*NA*	*NA*	*NA*
-0.5	0.00		−1.12	0.02	0.16	0.00	0.00	0.07	0.99	0.06	0.09	0.06	0.06	0.06
		(SD)	(0.26)	(0.36)	(0.37)	(0.37)	(0.37)	(0.37)	(0.10)	(0.24)	(0.29)	(0.24)	(0.24)	(0.24)
		(Mean SE)	(0.26)	(0.34)	(0.35)	(0.35)	(0.35)	(0.36)	*NA*	*NA*	*NA*	*NA*	*NA*	*NA*
	0.00		−0.71	0.03	−0.09	0.14	0.14	0.05	0.30	0.04	0.04	0.06	0.06	0.05
		(SD)	(0.43)	(0.60)	(0.60)	(0.62)	(0.62)	(0.61)	(0.46)	(0.19)	(0.20)	(0.23)	(0.23)	(0.22)
		(Mean SE)	(0.47)	(0.64)	(0.61)	(0.62)	(0.62)	(0.62)	*NA*	*NA*	*NA*	*NA*	*NA*	*NA*
	−0.67		−1.49	−0.79	−0.53	−0.90	−0.90	−0.75	1.00	0.85	0.39	0.94	0.94	0.76
		(SD)	(0.16)	(0.28)	(0.30)	(0.27)	(0.27)	(0.28)	(0.00)	(0.35)	(0.49)	(0.24)	(0.24)	(0.43)
		(Mean SE)	(0.17)	(0.25)	(0.31)	(0.25)	(0.25)	(0.28)	*NA*	*NA*	*NA*	*NA*	*NA*	*NA*
	0.17		1.45	0.28	0.25	0.24	0.24	0.24	1.00	0.17	0.15	0.14	0.14	0.13
		(SD)	(0.21)	(0.31)	(0.29)	(0.31)	(0.31)	(0.30)	(0.00)	(0.38)	(0.36)	(0.34)	(0.34)	(0.34)
		(Mean SE)	(0.22)	(0.30)	(0.28)	(0.29)	(0.29)	(0.29)	*NA*	*NA*	*NA*	*NA*	*NA*	*NA*

Table R. Mean effect estimates, empirical type-I error rate (for those SNPs with true effect 0 and underlined) and power with and without bias correction for some representative SNPs in the presence of 30% invalid IVs. Sample standard deviations (SD) and mean standard errors (Mean SE) are given in parenthesis. $p_2 = 4$.

ρ	True effects		Mean effect estimates						Type-I error rate or power					
			No	cML	Egger	IVW	Lasso	Median	No	cML	Egger	IVW	Lasso	Median
0	0.00		−0.78	0.03	−0.26	−0.14	−0.14	−0.11	1.00	0.08	0.25	0.12	0.12	0.07
		(SD)	(0.15)	(0.21)	(0.23)	(0.21)	(0.21)	(0.22)	(0.00)	(0.28)	(0.43)	(0.32)	(0.32)	(0.26)
		(Mean SE)	(0.15)	(0.19)	(0.22)	(0.20)	(0.20)	(0.22)	NA	NA	NA	NA	NA	NA
	0.00		−0.95	0.01	−0.02	−0.24	−0.24	−0.24	0.95	0.02	0.05	0.12	0.12	0.10
		(SD)	(0.26)	(0.37)	(0.39)	(0.37)	(0.37)	(0.40)	(0.23)	(0.15)	(0.22)	(0.32)	(0.32)	(0.30)
		(Mean SE)	(0.27)	(0.44)	(0.39)	(0.35)	(0.35)	(0.40)	NA	NA	NA	NA	NA	NA
	−0.64		−1.47	−0.73	−0.96	−0.81	−0.81	−0.78	1.00	1.00	1.00	1.00	1.00	1.00
		(SD)	(0.09)	(0.15)	(0.17)	(0.15)	(0.15)	(0.15)	(0.00)	(0.04)	(0.00)	(0.00)	(0.00)	(0.03)
		(Mean SE)	(0.09)	(0.15)	(0.16)	(0.13)	(0.13)	(0.16)	NA	NA	NA	NA	NA	NA
	0.65		0.59	0.85	1.09	0.97	0.97	0.97	1.00	0.98	1.00	1.00	1.00	1.00
		(SD)	(0.12)	(0.24)	(0.22)	(0.20)	(0.20)	(0.21)	(0.03)	(0.14)	(0.00)	(0.03)	(0.03)	(0.03)
		(Mean SE)	(0.13)	(0.19)	(0.19)	(0.17)	(0.17)	(0.20)	NA	NA	NA	NA	NA	NA
0.5	0.00		−0.08	0.16	−0.09	−0.20	−0.20	−0.09	0.06	0.03	0.06	0.15	0.15	0.04
		(SD)	(0.16)	(0.24)	(0.25)	(0.24)	(0.24)	(0.25)	(0.24)	(0.18)	(0.23)	(0.36)	(0.36)	(0.19)
		(Mean SE)	(0.17)	(0.30)	(0.26)	(0.23)	(0.23)	(0.27)	NA	NA	NA	NA	NA	NA
	0.00		−0.46	−0.15	−0.25	−0.19	−0.19	−0.14	0.34	0.06	0.11	0.09	0.09	0.08
		(SD)	(0.28)	(0.41)	(0.43)	(0.42)	(0.42)	(0.43)	(0.47)	(0.23)	(0.31)	(0.28)	(0.28)	(0.27)
		(Mean SE)	(0.30)	(0.42)	(0.40)	(0.39)	(0.39)	(0.40)	NA	NA	NA	NA	NA	NA
	−0.78		−0.30	−0.62	−0.52	−0.55	−0.55	−0.61	0.83	0.97	0.90	0.95	0.95	0.97
		(SD)	(0.10)	(0.17)	(0.17)	(0.17)	(0.17)	(0.17)	(0.38)	(0.17)	(0.30)	(0.22)	(0.22)	(0.18)
		(Mean SE)	(0.10)	(0.15)	(0.15)	(0.14)	(0.14)	(0.15)	NA	NA	NA	NA	NA	NA
	−0.17		1.29	−0.23	−0.07	0.04	0.04	−0.14	1.00	0.19	0.08	0.09	0.09	0.09
		(SD)	(0.13)	(0.24)	(0.25)	(0.24)	(0.24)	(0.24)	(0.00)	(0.39)	(0.27)	(0.28)	(0.28)	(0.28)
		(Mean SE)	(0.14)	(0.22)	(0.23)	(0.20)	(0.20)	(0.24)	NA	NA	NA	NA	NA	NA
-0.5	0.00		−1.97	0.04	0.10	0.13	0.13	0.11	1.00	0.03	0.08	0.09	0.09	0.06
		(SD)	(0.17)	(0.22)	(0.26)	(0.24)	(0.24)	(0.26)	(0.00)	(0.18)	(0.27)	(0.29)	(0.29)	(0.25)
		(Mean SE)	(0.17)	(0.23)	(0.24)	(0.24)	(0.24)	(0.26)	NA	NA	NA	NA	NA	NA
	0.00		0.04	0.00	0.15	0.16	0.16	0.17	0.05	0.07	0.11	0.11	0.11	0.10
		(SD)	(0.30)	(0.43)	(0.48)	(0.47)	(0.47)	(0.48)	(0.21)	(0.25)	(0.31)	(0.31)	(0.31)	(0.31)
		(Mean SE)	(0.31)	(0.41)	(0.41)	(0.40)	(0.40)	(0.42)	NA	NA	NA	NA	NA	NA
	−0.07		0.19	0.12	0.47	0.48	0.48	0.50	0.41	0.14	0.82	0.86	0.86	0.71
		(SD)	(0.10)	(0.17)	(0.18)	(0.17)	(0.17)	(0.19)	(0.49)	(0.35)	(0.38)	(0.34)	(0.34)	(0.45)
		(Mean SE)	(0.11)	(0.15)	(0.15)	(0.15)	(0.15)	(0.20)	NA	NA	NA	NA	NA	NA
	−0.91		−2.17	−0.51	−0.14	−0.09	−0.09	−0.11	1.00	0.66	0.15	0.13	0.13	0.09
		(SD)	(0.14)	(0.23)	(0.24)	(0.24)	(0.24)	(0.26)	(0.00)	(0.47)	(0.36)	(0.34)	(0.34)	(0.28)
		(Mean SE)	(0.15)	(0.21)	(0.21)	(0.20)	(0.20)	(0.25)	NA	NA	NA	NA	NA	NA

F.2.4 Figures of point estimates

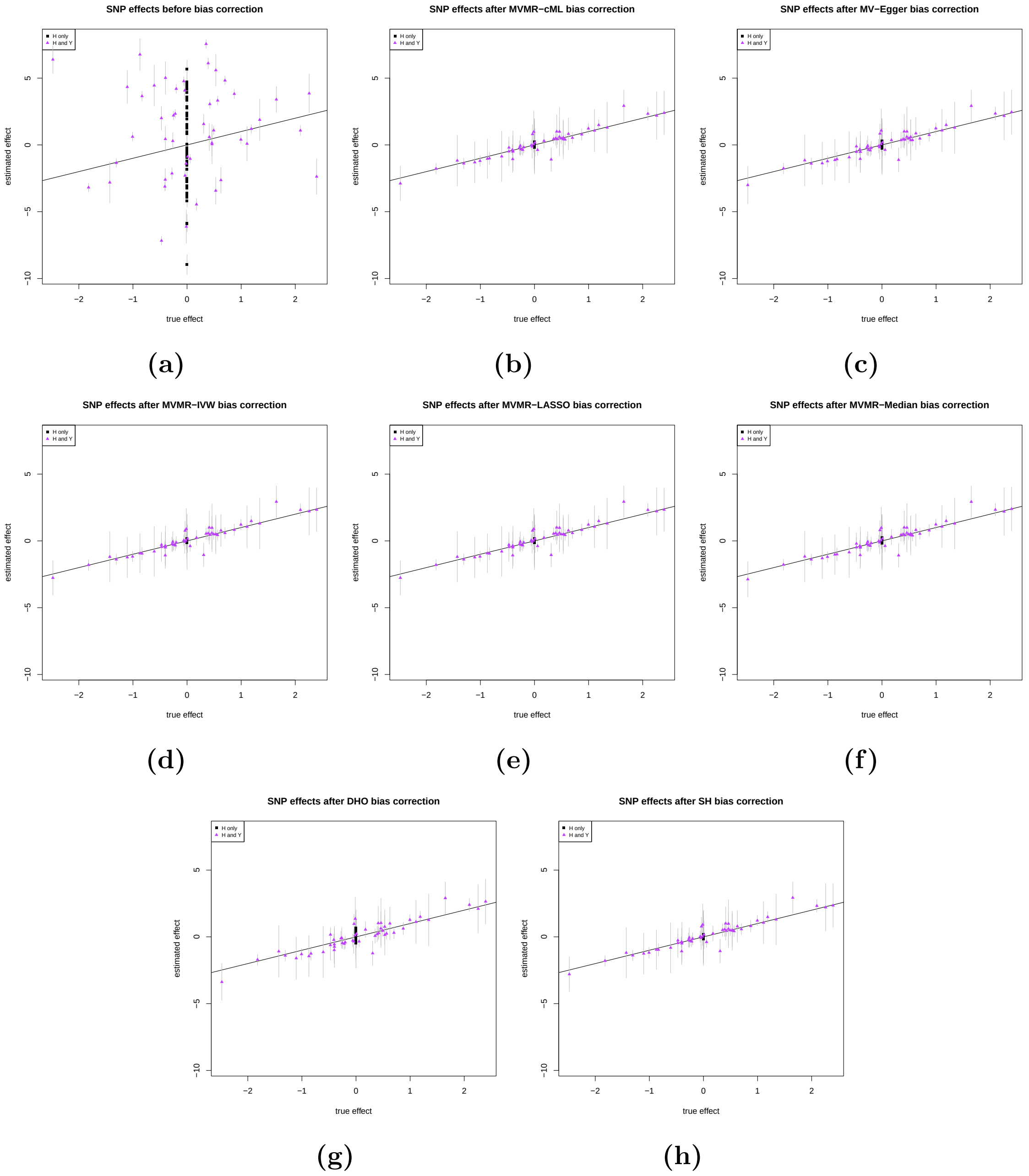

Fig H. Mean estimates of the effects of SNPs having collider bias with $\rho = 0$, $p_2 = 1$ and 30% invalid IVs. Horizontal coordinates are for the true effects, vertical coordinates are for the estimated effects. Vertical bars are the means of standard errors averaged over 1000 repetitions. In the legends, "$\boldsymbol{H}$ only" means the SNPs affecting only the covariates; "$\boldsymbol{H}$ and Y" means the SNPs affecting both the covariates and outcome.

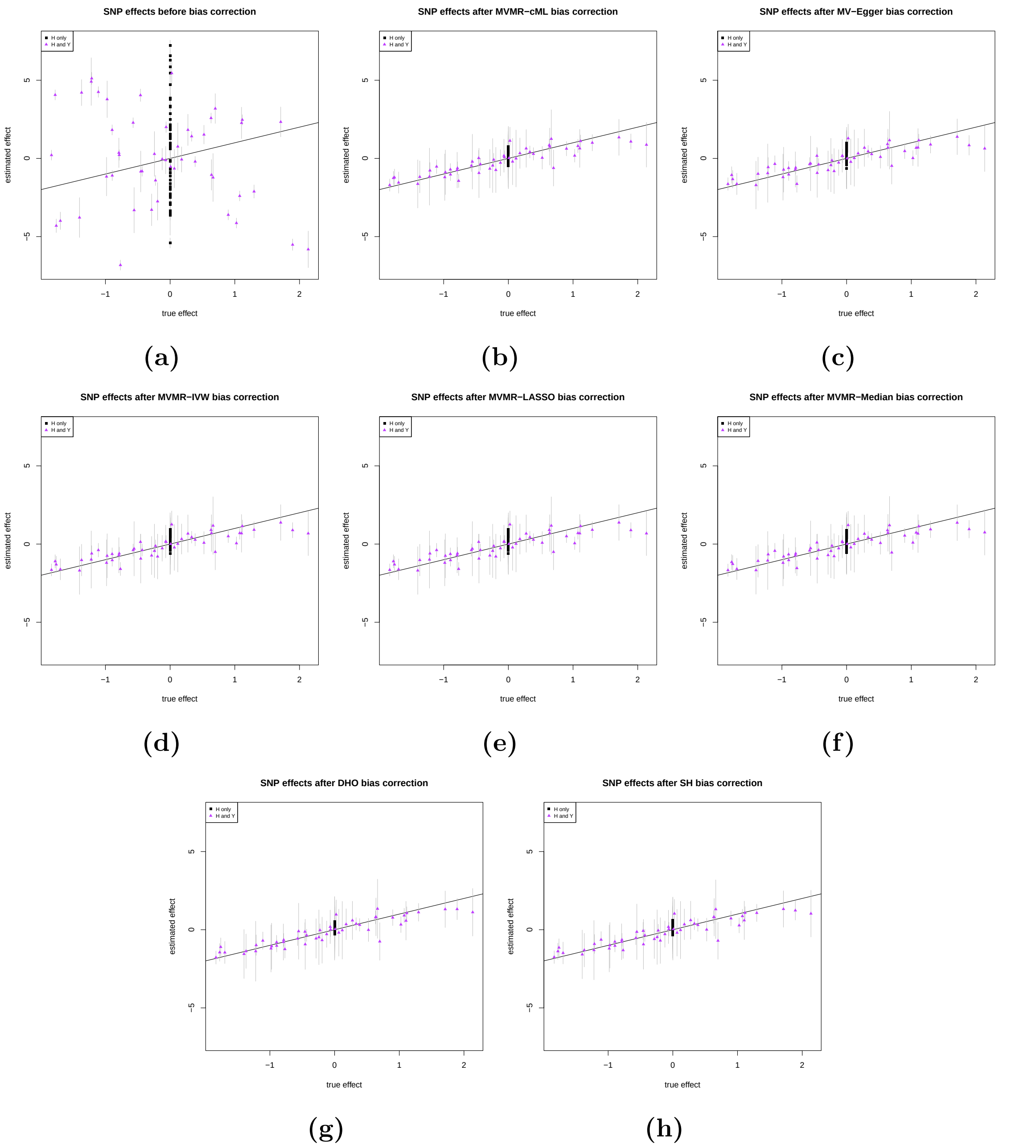

Fig I. Mean estimates of the effects of SNPs having collider bias with $\rho = 0.5$, $p_2 = 1$ and 30% invalid IVs. Horizontal coordinates are for the true effects, vertical coordinates are for the estimated effects. Vertical bars are the means of standard errors averaged over 1000 repetitions. In the legends, "$\boldsymbol{H}$ only" means the SNPs affecting only the covariates; "$\boldsymbol{H}$ and Y" means the SNPs affecting both the covariates and outcome.

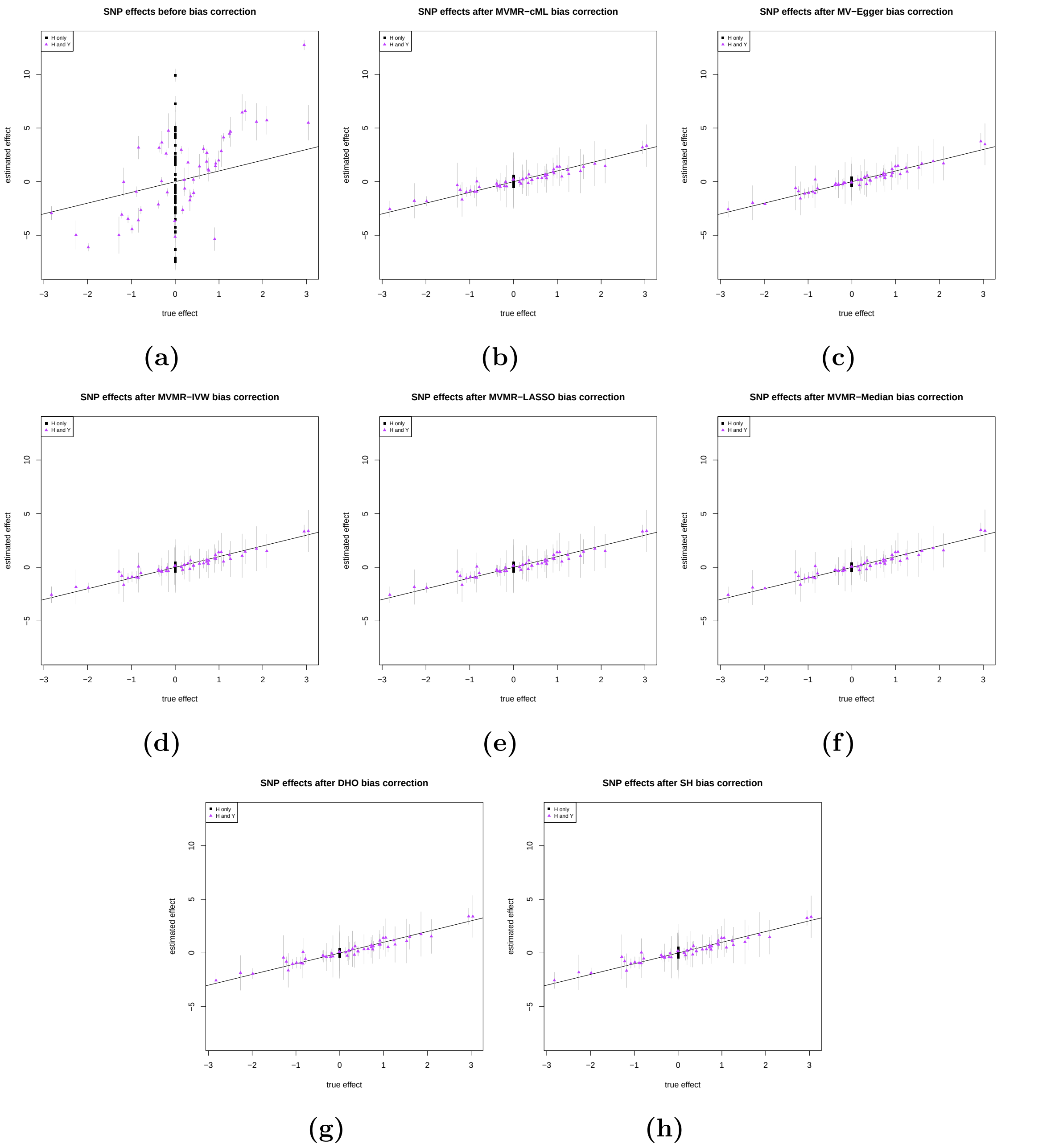

Fig J. Mean estimates of the effects of SNPs having collider bias with $\rho = -0.5$, $p_2 = 1$ and 30% invalid IVs. Horizontal coordinates are for the true effects, vertical coordinates are for the estimated effects. Vertical bars are the means of standard errors averaged over 1000 repetitions. In the legends, "$\boldsymbol{H}$ only" means the SNPs affecting only the covariates; "$\boldsymbol{H}$ and Y" means the SNPs affecting both the covariates and outcome.

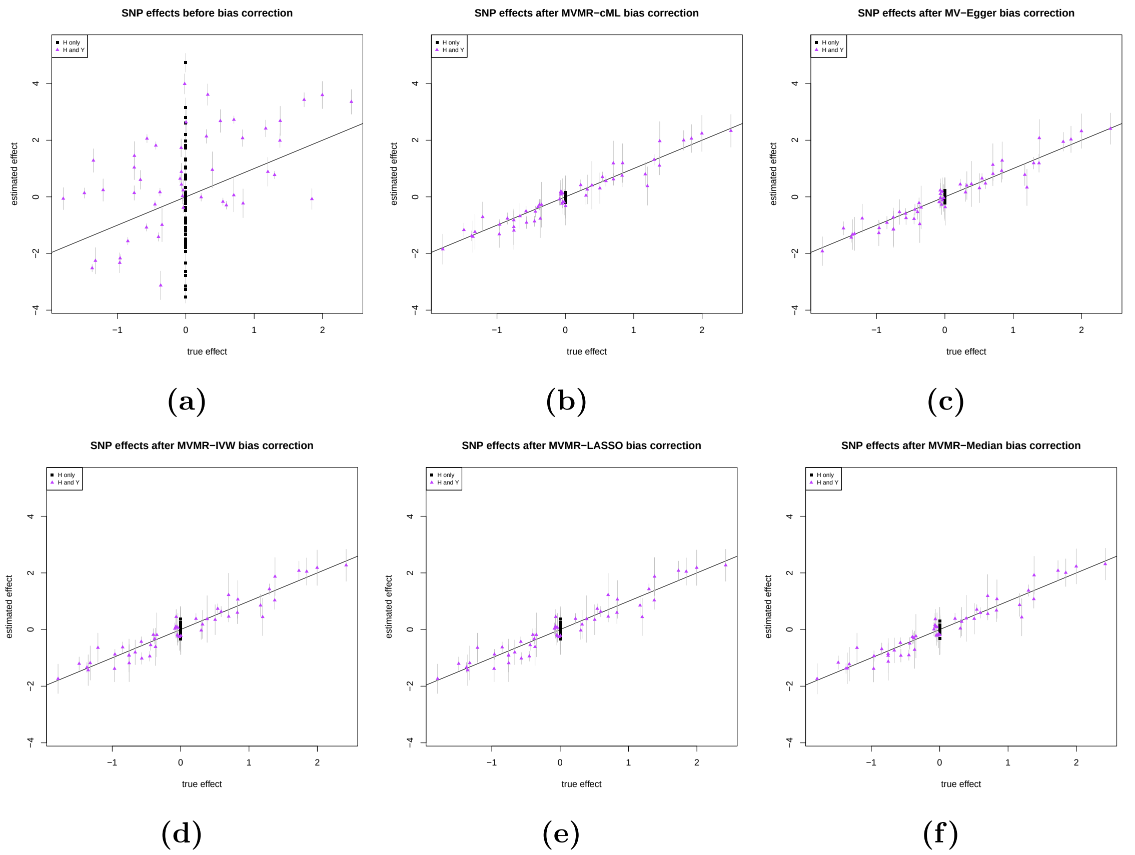

Fig K. Mean estimates of the effects of SNPs having collider bias with $\rho = 0$, $p_2 = 2$ and 30% invalid IVs. Horizontal coordinates are for the true effects, vertical coordinates are for the estimated effects. Vertical bars are the means of standard errors averaged over 1000 repetitions. In the legends, "$\boldsymbol{H}$ only" means the SNPs affecting only the covariates; "$\boldsymbol{H}$ and Y" means the SNPs affecting both the covariates and outcome.

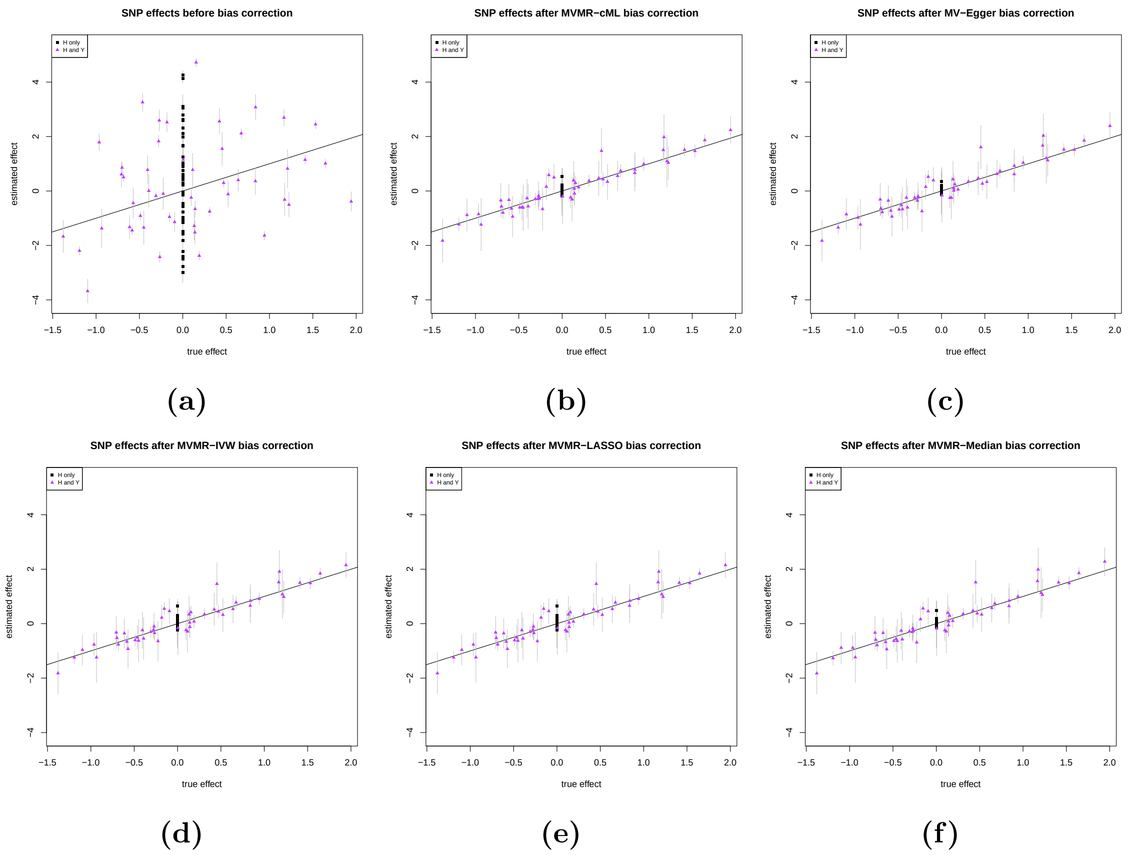

Fig L. Mean estimates of the effects of SNPs having collider bias with $\rho = 0.5$, $p_2 = 2$ and 30% invalid IVs. Horizontal coordinates are for the true effects, vertical coordinates are for the estimated effects. Vertical bars are the means of standard errors averaged over 1000 repetitions. In the legends, "$\boldsymbol{H}$ only" means the SNPs affecting only the covariates; "$\boldsymbol{H}$ and Y" means the SNPs affecting both the covariates and outcome.

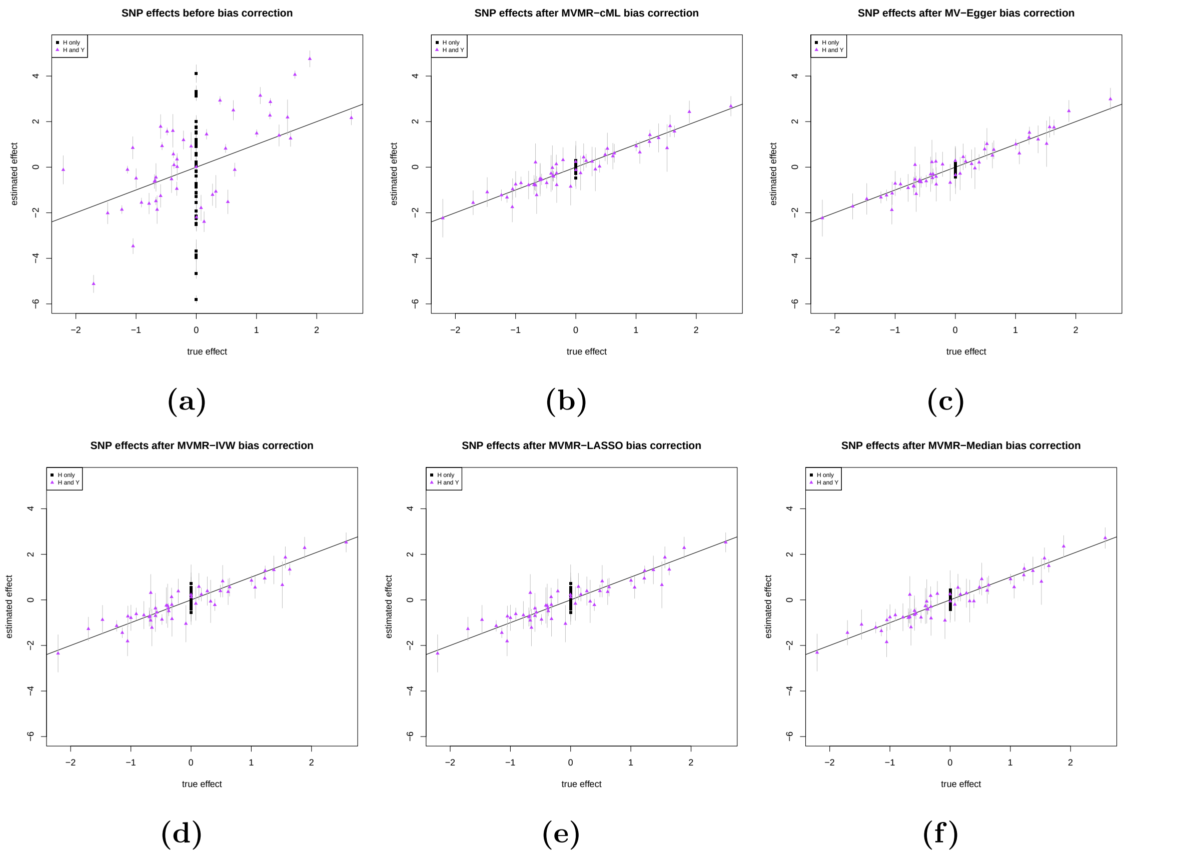

Fig M. Mean estimates of the effects of SNPs having collider bias with $\rho = -0.5$, $p_2 = 2$ and 30% invalid IVs. Horizontal coordinates are for the true effects, vertical coordinates are for the estimated effects. Vertical bars are the means of standard errors averaged over 1000 repetitions. In the legends, "$\boldsymbol{H}$ only" means the SNPs affecting only the covariates; "$\boldsymbol{H}$ and Y" means the SNPs affecting both the covariates and outcome

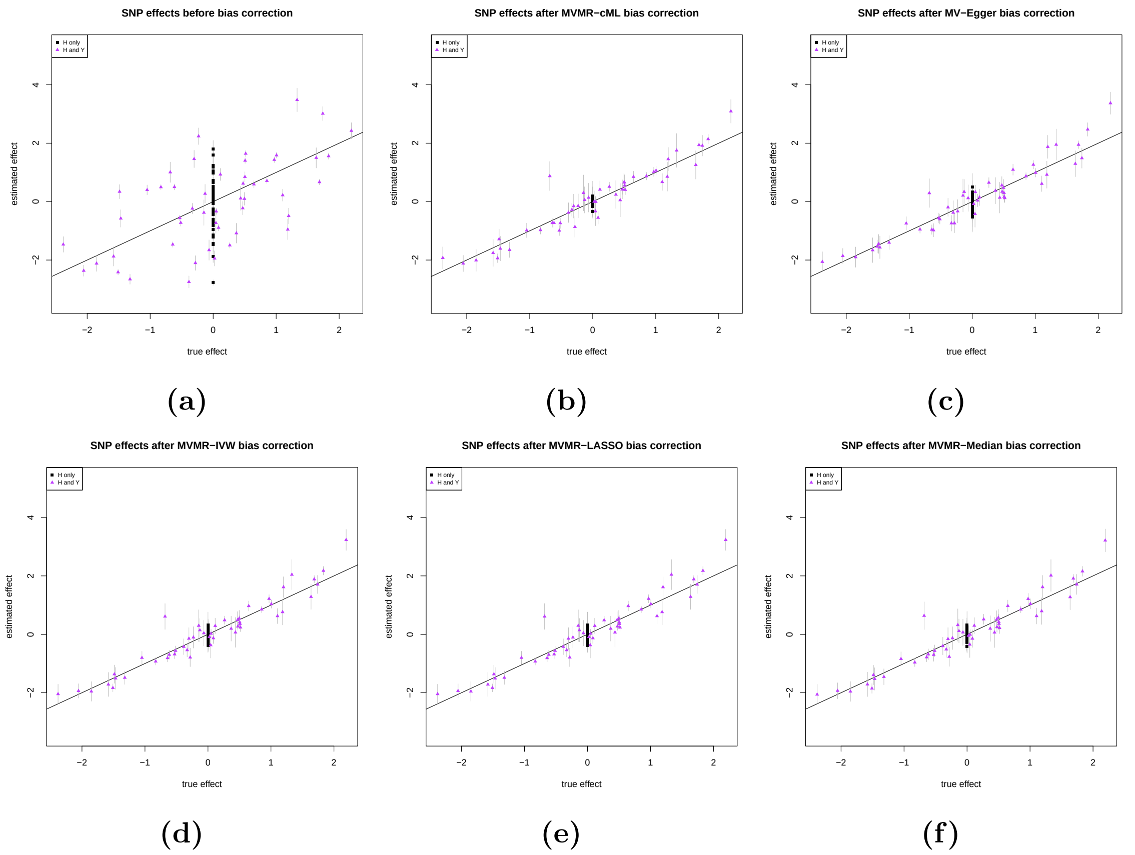

Fig N. Mean estimates of the effects of SNPs having collider bias with $\rho = 0$, $p_2 = 4$ and 30% invalid IVs. Horizontal coordinates are for the true effects, vertical coordinates are for the estimated effects. Vertical bars are the means of standard errors averaged over 1000 repetitions. In the legends, "$\boldsymbol{H}$ only" means the SNPs affecting only the covariates; "$\boldsymbol{H}$ and Y" means the SNPs affecting both the covariates and outcome

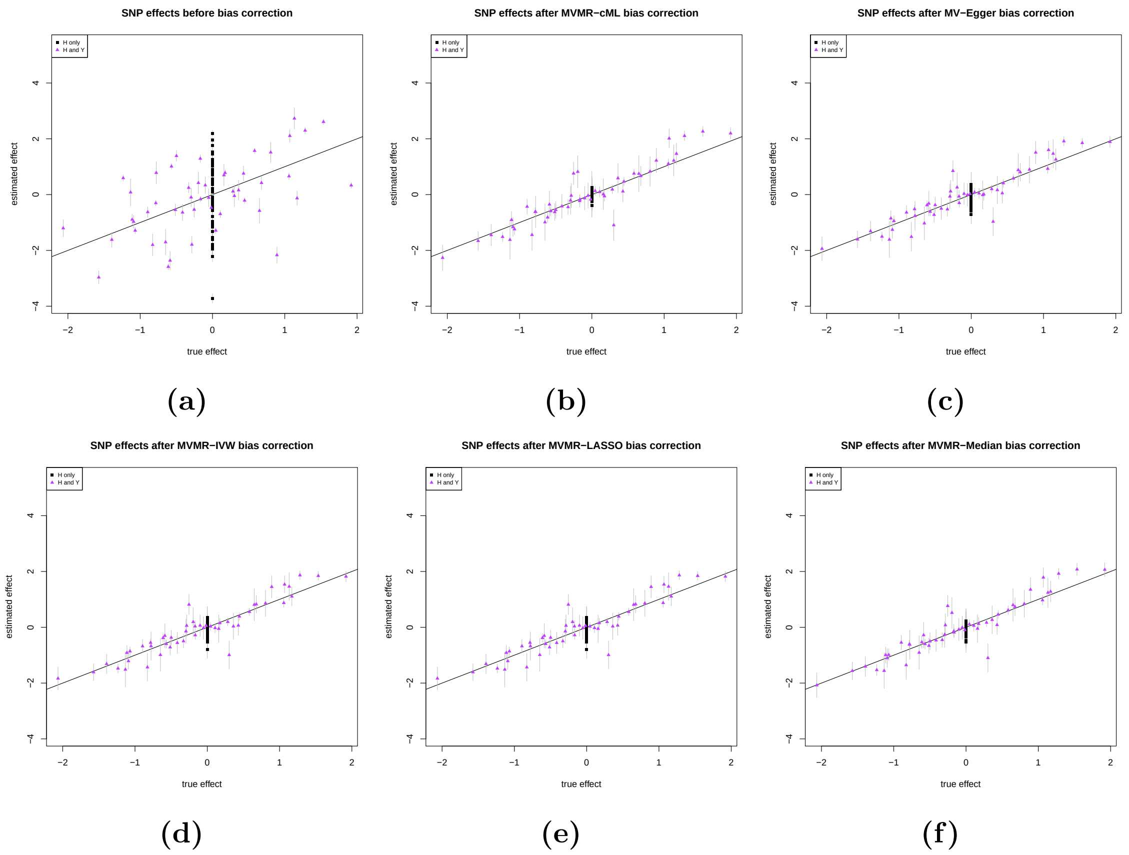

Fig O. Mean estimates of the effects of SNPs having collider bias with $\rho = 0.5$, $p_2 = 4$ and 30% invalid IVs. Horizontal coordinates are for the true effects, vertical coordinates are for the estimated effects. Vertical bars are the means of standard errors averaged over 1000 repetitions. In the legends, "$\boldsymbol{H}$ only" means the SNPs affecting only the covariates; "$\boldsymbol{H}$ and Y" means the SNPs affecting both the covariates and outcome

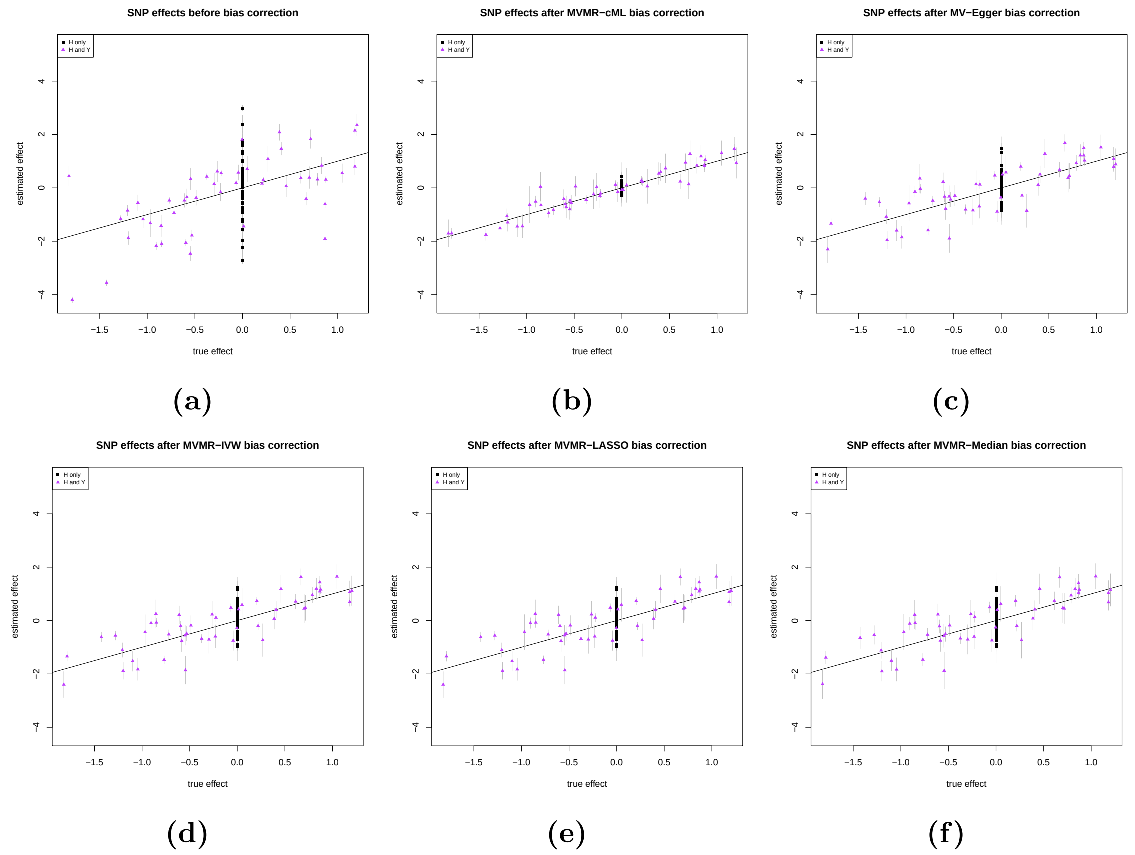

Fig P. Mean estimates of the effects of SNPs having collider bias with $\rho = -0.5$, $p_2 = 4$ and 30% invalid IVs. Horizontal coordinates are for the true effects, vertical coordinates are for the estimated effects. Vertical bars are the means of standard errors averaged over 1000 repetitions. In the legends, "$\boldsymbol{H}$ only" means the SNPs affecting only the covariates; "$\boldsymbol{H}$ and Y" means the SNPs affecting both the covariates and outcome

F.3 50% invalid IVs

F.3.1 Tables of empirical type-I error rate and power

Table S. Empirical type-I error rate (for SNPs underlined) and power with and without bias correction in the presence of 50% invalid IVs. Sample standard deviations (SD) are given in parenthesis. $p_2 = 1$.

ρ	Dimension of $\boldsymbol{H}$	1							
	Bias correction	No	cML	Egger	IVW	Lasso	Median	DHO	SH
0	Null SNPs	0.05	0.05	0.05	0.05	0.05	0.05	0.05	0.05
	(SD)	(0.01)	(0.01)	(0.01)	(0.01)	(0.01)	(0.01)	(0.01)	(0.01)
	All SNPs affecting $\boldsymbol{H}$ but not Y	0.74	0.06	0.07	0.06	0.06	0.05	0.14	0.05
	(SD)	(0.04)	(0.04)	(0.05)	(0.04)	(0.04)	(0.03)	(0.07)	(0.03)
	All SNPs affecting Y only	0.43	0.36	0.36	0.36	0.36	0.36	0.35	0.36
	(SD)	(0.04)	(0.05)	(0.05)	(0.05)	(0.05)	(0.05)	(0.05)	(0.05)
	All SNPs affecting both $\boldsymbol{H}$ and Y	0.74	0.32	0.31	0.32	0.32	0.30	0.35	0.30
	(SD)	(0.03)	(0.05)	(0.05)	(0.05)	(0.05)	(0.05)	(0.06)	(0.05)
	SNP with greatest increase in power	0.07	1.00	1.00	1.00	1.00	1.00	1.00	1.00
	(SD)	(0.26)	(0.00)	(0.00)	(0.00)	(0.00)	(0.00)	(0.00)	(0.00)
	SNP with greatest decrease in power	1.00	0.05	0.04	0.04	0.04	0.04	0.02	0.03
	(SD)	(0.00)	(0.22)	(0.19)	(0.20)	(0.20)	(0.20)	(0.15)	(0.16)
0.5	Null SNPs	0.05	0.05	0.05	0.05	0.05	0.05	0.05	0.05
	(SD)	(0.01)	(0.01)	(0.01)	(0.01)	(0.01)	(0.01)	(0.01)	(0.01)
	All SNPs affecting $\boldsymbol{H}$ but not Y	0.69	0.11	0.08	0.13	0.13	0.12	0.05	0.06
	(SD)	(0.04)	(0.05)	(0.05)	(0.05)	(0.05)	(0.06)	(0.03)	(0.04)
	All SNPs affecting Y only	0.45	0.36	0.36	0.37	0.37	0.37	0.34	0.35
	(SD)	(0.05)	(0.05)	(0.05)	(0.05)	(0.05)	(0.05)	(0.05)	(0.05)
	All SNPs affecting both $\boldsymbol{H}$ and Y	0.68	0.23	0.22	0.22	0.22	0.21	0.29	0.20
	(SD)	(0.04)	(0.05)	(0.06)	(0.05)	(0.05)	(0.05)	(0.07)	(0.05)
	SNP with greatest increase in power	0.27	0.99	0.99	0.98	0.98	0.98	0.84	0.98
	(SD)	(0.45)	(0.11)	(0.12)	(0.13)	(0.13)	(0.14)	(0.37)	(0.12)
	SNP with greatest decrease in power	1.00	0.06	0.01	0.00	0.00	0.00	0.06	0.00
	(SD)	(0.00)	(0.23)	(0.10)	(0.04)	(0.04)	(0.00)	(0.23)	(0.06)
−0.5	Null SNPs	0.05	0.05	0.05	0.05	0.05	0.05	0.05	0.05
	(SD)	(0.01)	(0.01)	(0.01)	(0.01)	(0.01)	(0.01)	(0.01)	(0.01)
	All SNPs affecting $\boldsymbol{H}$ but not Y	0.73	0.11	0.09	0.10	0.10	0.11	0.13	0.07
	(SD)	(0.04)	(0.05)	(0.05)	(0.05)	(0.05)	(0.06)	(0.07)	(0.05)
	All SNPs affecting Y only	0.36	0.27	0.27	0.27	0.27	0.27	0.27	0.27
	(SD)	(0.05)	(0.05)	(0.05)	(0.05)	(0.05)	(0.05)	(0.05)	(0.05)
	All SNPs affecting both $\boldsymbol{H}$ and Y	0.78	0.26	0.24	0.26	0.26	0.25	0.23	0.24
	(SD)	(0.03)	(0.06)	(0.05)	(0.05)	(0.05)	(0.05)	(0.05)	(0.06)
	SNP with greatest increase in power	0.57	0.85	0.85	0.85	0.85	0.86	0.88	0.82
	(SD)	(0.50)	(0.35)	(0.36)	(0.36)	(0.36)	(0.35)	(0.33)	(0.39)
	SNP with greatest decrease in power	1.00	0.03	0.00	0.04	0.04	0.00	0.01	0.01
	(SD)	(0.00)	(0.16)	(0.07)	(0.19)	(0.19)	(0.06)	(0.12)	(0.12)

Table T. Empirical type-I error rate (for SNPs underlined) and power with and without bias correction in the presence of 50% invalid IVs. Sample standard deviations (SD) are given in parenthesis. $p_2 > 1$.

ρ	Dimension of $\boldsymbol{H}$	2						4					
	Bias correction	No	cML	Egger	IVW	Lasso	Median	No	cML	Egger	IVW	Lasso	Median
0	Null SNPs	0.05	0.06	0.06	0.06	0.06	0.06	0.05	0.06	0.10	0.10	0.10	0.09
	(SD)	(0.01)	(0.01)	(0.01)	(0.01)	(0.01)	(0.01)	(0.01)	(0.01)	(0.01)	(0.01)	(0.01)	(0.01)
	All SNPs affecting $\boldsymbol{H}$ but not Y	0.76	0.12	0.05	0.06	0.06	0.06	0.65	0.13	0.23	0.24	0.23	0.09
	(SD)	(0.04)	(0.07)	(0.05)	(0.04)	(0.04)	(0.04)	(0.04)	(0.06)	(0.08)	(0.07)	(0.06)	(0.06)
	All SNPs affecting Y only	0.61	0.52	0.51	0.52	0.52	0.53	0.78	0.70	0.71	0.71	0.71	0.72
	(SD)	(0.05)	(0.05)	(0.05)	(0.05)	(0.05)	(0.05)	(0.03)	(0.04)	(0.05)	(0.04)	(0.04)	(0.04)
	All SNPs affecting both $\boldsymbol{H}$ and Y	0.77	0.50	0.45	0.52	0.52	0.49	0.75	0.64	0.69	0.70	0.70	0.64
	(SD)	(0.04)	(0.05)	(0.05)	(0.05)	(0.05)	(0.05)	(0.03)	(0.04)	(0.06)	(0.05)	(0.05)	(0.05)
	SNP with greatest increase in power	0.09	0.83	0.91	0.90	0.90	0.85	0.08	1.00	1.00	1.00	1.00	1.00
	(SD)	(0.29)	(0.38)	(0.29)	(0.29)	(0.29)	(0.35)	(0.28)	(0.00)	(0.00)	(0.00)	(0.00)	(0.00)
	SNP with greatest decrease in power	1.00	0.01	0.05	0.06	0.06	0.06	1.00	0.08	0.10	0.11	0.10	0.08
	(SD)	(0.00)	(0.12)	(0.23)	(0.23)	(0.23)	(0.23)	(0.06)	(0.27)	(0.30)	(0.31)	(0.29)	(0.28)
0.5	Null SNPs	0.05	0.05	0.06	0.06	0.06	0.06	0.05	0.06	0.10	0.10	0.10	0.10
	(SD)	(0.01)	(0.01)	(0.01)	(0.01)	(0.01)	(0.01)	(0.01)	(0.01)	(0.01)	(0.01)	(0.01)	(0.01)
	All SNPs affecting $\boldsymbol{H}$ but not Y	0.74	0.10	0.07	0.10	0.10	0.06	0.71	0.14	0.43	0.47	0.47	0.18
	(SD)	(0.04)	(0.06)	(0.04)	(0.04)	(0.04)	(0.04)	(0.04)	(0.11)	(0.06)	(0.06)	(0.06)	(0.09)
	All SNPs affecting Y only	0.64	0.57	0.58	0.58	0.58	0.58	0.70	0.64	0.67	0.67	0.67	0.67
	(SD)	(0.04)	(0.05)	(0.05)	(0.05)	(0.05)	(0.05)	(0.04)	(0.05)	(0.04)	(0.04)	(0.04)	(0.04)
	All SNPs affecting both $\boldsymbol{H}$ and Y	0.73	0.40	0.42	0.42	0.42	0.40	0.80	0.54	0.66	0.67	0.67	0.58
	(SD)	(0.04)	(0.06)	(0.05)	(0.05)	(0.05)	(0.05)	(0.04)	(0.05)	(0.05)	(0.05)	(0.05)	(0.05)
	SNP with greatest increase in power	0.16	0.98	0.98	0.98	0.98	0.98	0.08	0.96	0.95	0.96	0.96	1.00
	(SD)	(0.36)	(0.13)	(0.13)	(0.13)	(0.13)	(0.13)	(0.28)	(0.20)	(0.21)	(0.20)	(0.20)	(0.06)
	SNP with greatest decrease in power	1.00	0.02	0.03	0.02	0.02	0.01	1.00	0.09	0.10	0.12	0.12	0.06
	(SD)	(0.00)	(0.16)	(0.16)	(0.14)	(0.14)	(0.08)	(0.03)	(0.29)	(0.30)	(0.33)	(0.33)	(0.24)
−0.5	Null SNPs	0.05	0.06	0.06	0.06	0.06	0.06	0.05	0.06	0.10	0.10	0.10	0.09
	(SD)	(0.01)	(0.01)	(0.01)	(0.01)	(0.01)	(0.01)	(0.01)	(0.01)	(0.02)	(0.02)	(0.02)	(0.01)
	All SNPs affecting $\boldsymbol{H}$ but not Y	0.76	0.06	0.09	0.06	0.06	0.06	0.68	0.27	0.30	0.29	0.28	0.17
	(SD)	(0.04)	(0.03)	(0.07)	(0.03)	(0.03)	(0.03)	(0.04)	(0.09)	(0.07)	(0.06)	(0.06)	(0.08)
	All SNPs affecting Y only	0.57	0.48	0.50	0.49	0.49	0.49	0.71	0.63	0.65	0.66	0.66	0.66
	(SD)	(0.04)	(0.05)	(0.05)	(0.05)	(0.05)	(0.05)	(0.03)	(0.04)	(0.04)	(0.04)	(0.04)	(0.04)
	All SNPs affecting both $\boldsymbol{H}$ and Y	0.83	0.42	0.48	0.44	0.44	0.43	0.71	0.58	0.64	0.66	0.66	0.57
	(SD)	(0.04)	(0.05)	(0.07)	(0.05)	(0.05)	(0.05)	(0.05)	(0.05)	(0.05)	(0.05)	(0.05)	(0.04)
	SNP with greatest increase in power	0.06	0.48	0.39	0.51	0.51	0.49	0.11	1.00	0.97	1.00	1.00	1.00
	(SD)	(0.24)	(0.50)	(0.49)	(0.50)	(0.50)	(0.50)	(0.31)	(0.00)	(0.16)	(0.00)	(0.00)	(0.00)
	SNP with greatest decrease in power	1.00	0.04	0.05	0.05	0.05	0.05	1.00	0.10	0.14	0.14	0.14	0.08
	(SD)	(0.00)	(0.19)	(0.22)	(0.22)	(0.22)	(0.22)	(0.00)	(0.30)	(0.34)	(0.35)	(0.35)	(0.27)

F.3.2 Estimates of slope vector

Table U. True values and mean estimates of $\boldsymbol{b}$ with 50% invalid IVs. Sample standard deviations (SD), mean standard errors (Mean SE) are given in parenthesis. $p_2 = 1$.

ρ		True value	MVMR-cML	MVMR-Egger	MVMR-IVW	MVMR-Lasso	MVMR-Median	DHO	SH
0		−3.28	−3.56	−3.80	−3.64	−3.64	−3.56	−4.18	−3.65
	(SD)	(0.00)	(0.19)	(0.23)	(0.14)	(0.14)	(0.16)	(0.28)	(0.18)
	(Mean SE)	(0.00)	(0.14)	(0.28)	(0.14)	(0.14)	(0.21)	(0.32)	(0.27)
0.5		−3.38	−3.14	−3.25	−3.07	−3.07	−3.06	−3.65	−3.29
	(SD)	(0.00)	(0.15)	(0.19)	(0.12)	(0.12)	(0.17)	(0.25)	(0.16)
	(Mean SE)	(0.00)	(0.12)	(0.22)	(0.12)	(0.12)	(0.18)	(0.26)	(0.32)
−0.5		−3.17	−3.53	−3.55	−3.52	−3.52	−3.60	−3.69	−3.50
	(SD)	(0.00)	(0.16)	(0.16)	(0.10)	(0.10)	(0.14)	(0.20)	(0.17)
	(Mean SE)	(0.00)	(0.11)	(0.22)	(0.11)	(0.11)	(0.17)	(0.21)	(0.26)

Table V. True values and mean estimates of $\boldsymbol{b}$ with 50% invalid IVs. Sample standard deviations (SD), mean standard errors (Mean SE) are given in parenthesis. $p_2 > 1$.

ρ	Dimension of $\boldsymbol{b}$	2		4			
	$\boldsymbol{b}$	b_1	b_2	b_1	b_2	b_3	b_4
0	True value	−1.38	−1.16	−0.43	−0.65	−0.48	−0.37
	MVMR-cML	−1.27	−1.02	−0.41	−0.59	−0.62	−0.27
	(SD)	(0.11)	(0.09)	(0.09)	(0.11)	(0.08)	(0.07)
	(Mean SE)	(0.07)	(0.06)	(0.05)	(0.05)	(0.04)	(0.04)
	MVMR-Egger	−1.38	−1.23	−0.80	−0.70	−0.60	−0.21
	(SD)	(0.19)	(0.06)	(0.11)	(0.08)	(0.05)	(0.07)
	(Mean SE)	(0.24)	(0.06)	(0.10)	(0.06)	(0.04)	(0.05)
	MVMR-IVW	−1.25	−1.22	−0.75	−0.70	−0.60	−0.21
	(SD)	(0.06)	(0.05)	(0.06)	(0.07)	(0.04)	(0.08)
	(Mean SE)	(0.07)	(0.06)	(0.04)	(0.05)	(0.04)	(0.04)
	MVMR-Lasso	−1.25	−1.22	−0.75	−0.70	−0.60	−0.21
	(SD)	(0.06)	(0.05)	(0.06)	(0.08)	(0.04)	(0.08)
	(Mean SE)	(0.07)	(0.06)	(0.05)	(0.05)	(0.04)	(0.04)
	MVMR-Median	−1.25	−1.14	−0.57	−0.61	−0.62	−0.28
	(SD)	(0.08)	(0.09)	(0.08)	(0.11)	(0.06)	(0.09)
	(Mean SE)	(0.10)	(0.12)	(0.08)	(0.08)	(0.06)	(0.08)
0.5	True value	−1.10	−1.28	−0.44	−0.49	−0.46	−0.44
	MVMR-cML	−1.08	−1.21	−0.48	−0.56	−0.54	−0.43
	(SD)	(0.12)	(0.06)	(0.13)	(0.11)	(0.09)	(0.13)
	(Mean SE)	(0.05)	(0.05)	(0.04)	(0.05)	(0.04)	(0.04)
	MVMR-Egger	−1.13	−1.18	−0.34	−0.38	−0.59	−0.65
	(SD)	(0.06)	(0.04)	(0.07)	(0.04)	(0.04)	(0.09)
	(Mean SE)	(0.07)	(0.05)	(0.06)	(0.04)	(0.04)	(0.03)
	MVMR-IVW	−1.07	−1.19	−0.31	−0.39	−0.60	−0.65
	(SD)	(0.04)	(0.04)	(0.03)	(0.04)	(0.05)	(0.08)
	(Mean SE)	(0.04)	(0.04)	(0.03)	(0.04)	(0.03)	(0.03)
	MVMR-Lasso	−1.07	−1.19	−0.31	−0.39	−0.60	−0.65
	(SD)	(0.04)	(0.04)	(0.03)	(0.04)	(0.05)	(0.08)
	(Mean SE)	(0.04)	(0.04)	(0.03)	(0.04)	(0.03)	(0.03)
	MVMR-Median	−1.12	−1.20	−0.44	−0.51	−0.63	−0.51
	(SD)	(0.06)	(0.05)	(0.05)	(0.06)	(0.06)	(0.10)
	(Mean SE)	(0.09)	(0.07)	(0.06)	(0.08)	(0.07)	(0.07)
−0.5	True value	−1.15	−1.27	−0.48	−0.48	−0.35	−0.56
	MVMR-cML	−1.09	−1.33	−0.16	−0.79	−0.26	−0.84
	(SD)	(0.07)	(0.06)	(0.09)	(0.18)	(0.15)	(0.11)
	(Mean SE)	(0.06)	(0.05)	(0.05)	(0.06)	(0.05)	(0.06)
	MVMR-Egger	−0.89	−1.35	−0.61	−0.86	−0.23	−0.77
	(SD)	(0.12)	(0.05)	(0.08)	(0.12)	(0.12)	(0.12)
	(Mean SE)	(0.16)	(0.05)	(0.10)	(0.06)	(0.05)	(0.06)
	MVMR-IVW	−1.09	−1.36	−0.40	−0.85	−0.22	−0.81
	(SD)	(0.06)	(0.05)	(0.06)	(0.08)	(0.10)	(0.13)
	(Mean SE)	(0.06)	(0.05)	(0.05)	(0.06)	(0.05)	(0.06)
	MVMR-Lasso	−1.09	−1.36	−0.40	−0.85	−0.22	−0.80
	(SD)	(0.06)	(0.05)	(0.06)	(0.08)	(0.10)	(0.13)
	(Mean SE)	(0.06)	(0.05)	(0.05)	(0.06)	(0.05)	(0.06)
	MVMR-Median	−1.06	−1.32	−0.21	−0.78	−0.29	−0.83
	(SD)	(0.07)	(0.06)	(0.06)	(0.08)	(0.15)	(0.09)
	(Mean SE)	(0.10)	(0.08)	(0.09)	(0.10)	(0.08)	(0.11)

F.3.3 Effect estimates, empirical type-I error rate and power of representative SNPs

Table W. Mean effect estimates, empirical type-I error rate (for those SNPs with true effect 0 and underlined) and power with and without bias correction for some representative SNPs in the presence of 50% invalid IVs. Sample standard deviations (SD) and mean standard errors (Mean SE) are given in parenthesis. $p_2 = 1$.

ρ	True effects		Mean effect estimates								Type-I error rate or power							
			No	cML	Egger	IVW	Lasso	Median	DHO	SH	No	cML	Egger	IVW	Lasso	Median	DHO	SH
0			−0.56	0.05	0.09	0.06	0.06	0.05	0.15	0.06	0.13	0.05	0.05	0.05	0.05	0.05	0.06	0.05
	0.00	(SD)	(0.64)	(0.83)	(0.85)	(0.83)	(0.83)	(0.82)	(0.88)	(0.83)	(0.33)	(0.23)	(0.22)	(0.22)	(0.22)	(0.23)	(0.23)	(0.22)
		(Mean SE)	(0.65)	(0.81)	(0.83)	(0.82)	(0.82)	(0.81)	(0.86)	(0.82)	*NA*	*NA*	*NA*	*NA*	*NA*	*NA*	*NA*	*NA*
			−3.00	0.36	0.59	0.44	0.44	0.36	0.94	0.44	0.55	0.06	0.06	0.06	0.06	0.06	0.07	0.06
	0.00	(SD)	(1.42)	(1.82)	(1.87)	(1.82)	(1.82)	(1.82)	(1.94)	(1.83)	(0.50)	(0.23)	(0.24)	(0.24)	(0.24)	(0.23)	(0.26)	(0.23)
		(Mean SE)	(1.45)	(1.81)	(1.86)	(1.82)	(1.82)	(1.81)	(1.94)	(1.84)	*NA*	*NA*	*NA*	*NA*	*NA*	*NA*	*NA*	*NA*
			−3.26	−1.02	−0.87	−0.97	−0.97	−1.02	−0.64	−0.97	1.00	0.57	0.41	0.53	0.53	0.56	0.22	0.49
	−0.28	(SD)	(0.37)	(0.48)	(0.50)	(0.48)	(0.48)	(0.48)	(0.54)	(0.49)	(0.00)	(0.50)	(0.49)	(0.50)	(0.50)	(0.50)	(0.41)	(0.50)
		(Mean SE)	(0.38)	(0.48)	(0.52)	(0.48)	(0.48)	(0.49)	(0.54)	(0.51)	*NA*	*NA*	*NA*	*NA*	*NA*	*NA*	*NA*	*NA*
			4.21	2.00	1.85	1.95	1.95	2.00	1.62	1.95	1.00	1.00	0.98	1.00	1.00	1.00	0.90	0.99
	2.26	(SD)	(0.32)	(0.44)	(0.46)	(0.42)	(0.42)	(0.43)	(0.49)	(0.42)	(0.00)	(0.04)	(0.15)	(0.04)	(0.04)	(0.05)	(0.30)	(0.07)
		(Mean SE)	(0.33)	(0.42)	(0.45)	(0.42)	(0.42)	(0.43)	(0.48)	(0.45)	*NA*	*NA*	*NA*	*NA*	*NA*	*NA*	*NA*	*NA*
0.5			2.74	0.40	0.31	0.45	0.45	0.46	0.02	0.29	0.99	0.09	0.07	0.09	0.09	0.10	0.05	0.05
	0.00	(SD)	(0.62)	(0.76)	(0.76)	(0.75)	(0.75)	(0.75)	(0.80)	(0.77)	(0.10)	(0.28)	(0.26)	(0.29)	(0.29)	(0.29)	(0.21)	(0.23)
		(Mean SE)	(0.63)	(0.76)	(0.78)	(0.75)	(0.75)	(0.76)	(0.82)	(0.81)	*NA*	*NA*	*NA*	*NA*	*NA*	*NA*	*NA*	*NA*
			−3.98	−0.57	−0.45	−0.65	−0.65	−0.66	−0.02	−0.41	0.80	0.07	0.06	0.07	0.07	0.07	0.05	0.05
	0.00	(SD)	(1.39)	(1.68)	(1.71)	(1.67)	(1.67)	(1.69)	(1.77)	(1.70)	(0.40)	(0.25)	(0.23)	(0.26)	(0.26)	(0.25)	(0.22)	(0.22)
		(Mean SE)	(1.40)	(1.69)	(1.71)	(1.66)	(1.66)	(1.67)	(1.78)	(1.74)	*NA*	*NA*	*NA*	*NA*	*NA*	*NA*	*NA*	*NA*
			−3.36	0.53	0.67	0.44	0.44	0.43	1.16	0.71	1.00	0.21	0.23	0.15	0.15	0.14	0.52	0.15
	1.30	(SD)	(0.36)	(0.48)	(0.50)	(0.47)	(0.47)	(0.49)	(0.55)	(0.50)	(0.00)	(0.41)	(0.42)	(0.36)	(0.36)	(0.35)	(0.50)	(0.36)
		(Mean SE)	(0.37)	(0.47)	(0.52)	(0.46)	(0.46)	(0.49)	(0.57)	(0.61)	*NA*	*NA*	*NA*	*NA*	*NA*	*NA*	*NA*	*NA*
			0.61	0.09	0.07	0.10	0.10	0.10	−0.00	0.06	0.48	0.05	0.05	0.05	0.05	0.05	0.05	0.05
	−0.24	(SD)	(0.31)	(0.37)	(0.39)	(0.37)	(0.37)	(0.37)	(0.41)	(0.38)	(0.50)	(0.22)	(0.23)	(0.23)	(0.23)	(0.22)	(0.22)	(0.22)
		(Mean SE)	(0.32)	(0.38)	(0.39)	(0.38)	(0.38)	(0.38)	(0.40)	(0.39)	*NA*	*NA*	*NA*	*NA*	*NA*	*NA*	*NA*	*NA*
-0.5			−0.10	0.02	0.02	0.02	0.02	0.03	0.03	0.02	0.05	0.05	0.05	0.05	0.05	0.05	0.04	0.05
	0.00	(SD)	(0.68)	(0.87)	(0.87)	(0.87)	(0.87)	(0.87)	(0.88)	(0.87)	(0.22)	(0.21)	(0.21)	(0.21)	(0.21)	(0.21)	(0.21)	(0.21)
		(Mean SE)	(0.70)	(0.88)	(0.88)	(0.88)	(0.88)	(0.88)	(0.89)	(0.87)	*NA*	*NA*	*NA*	*NA*	*NA*	*NA*	*NA*	*NA*
			2.77	−0.75	−0.77	−0.74	−0.74	−0.82	−0.91	−0.72	0.44	0.06	0.06	0.06	0.06	0.06	0.07	0.06
	0.00	(SD)	(1.51)	(1.90)	(1.90)	(1.89)	(1.89)	(1.91)	(1.93)	(1.90)	(0.50)	(0.24)	(0.23)	(0.24)	(0.24)	(0.24)	(0.25)	(0.23)
		(Mean SE)	(1.56)	(1.94)	(1.96)	(1.94)	(1.94)	(1.97)	(1.99)	(1.96)	*NA*	*NA*	*NA*	*NA*	*NA*	*NA*	*NA*	*NA*
			−0.25	−0.75	−0.75	−0.75	−0.75	−0.76	−0.77	−0.75	0.08	0.32	0.32	0.32	0.32	0.32	0.33	0.32
	−0.89	(SD)	(0.41)	(0.53)	(0.53)	(0.53)	(0.53)	(0.53)	(0.53)	(0.53)	(0.28)	(0.47)	(0.47)	(0.47)	(0.47)	(0.47)	(0.47)	(0.47)
		(Mean SE)	(0.41)	(0.51)	(0.52)	(0.51)	(0.51)	(0.52)	(0.52)	(0.51)	*NA*	*NA*	*NA*	*NA*	*NA*	*NA*	*NA*	*NA*
			2.98	−0.47	−0.49	−0.46	−0.46	−0.54	−0.63	−0.44	1.00	0.18	0.14	0.15	0.15	0.18	0.24	0.10
	−0.16	(SD)	(0.34)	(0.43)	(0.45)	(0.43)	(0.43)	(0.44)	(0.47)	(0.45)	(0.00)	(0.39)	(0.34)	(0.36)	(0.36)	(0.38)	(0.43)	(0.30)
		(Mean SE)	(0.36)	(0.44)	(0.49)	(0.46)	(0.46)	(0.48)	(0.49)	(0.51)	*NA*	*NA*	*NA*	*NA*	*NA*	*NA*	*NA*	*NA*

Table X. Mean effect estimates, empirical type-I error rate (for those SNPs with true effect 0 and underlined) and power with and without bias correction for some representative SNPs in the presence of 50% invalid IVs. Sample standard deviations (SD) and mean standard errors (Mean SE) are given in parenthesis. $p_2 = 2$.

ρ	True effects		Mean effect estimates						Type-I error rate or power					
			No	cML	Egger	IVW	Lasso	Median	No	cML	Egger	IVW	Lasso	Median
0			0.90	0.33	0.13	0.06	0.06	0.17	0.26	0.08	0.06	0.07	0.07	0.06
	0.00	(SD)	(0.65)	(0.89)	(0.95)	(0.92)	(0.92)	(0.91)	(0.44)	(0.27)	(0.24)	(0.25)	(0.25)	(0.25)
		(Mean SE)	(0.67)	(0.85)	(0.88)	(0.85)	(0.85)	(0.85)	*NA*	*NA*	*NA*	*NA*	*NA*	*NA*
			1.09	0.01	−0.11	−0.01	−0.01	0.00	1.00	0.06	0.06	0.06	0.06	0.04
	0.00	(SD)	(0.16)	(0.21)	(0.26)	(0.21)	(0.21)	(0.21)	(0.00)	(0.24)	(0.24)	(0.24)	(0.24)	(0.20)
		(Mean SE)	(0.16)	(0.20)	(0.27)	(0.21)	(0.21)	(0.22)	*NA*	*NA*	*NA*	*NA*	*NA*	*NA*
			3.21	2.00	1.58	1.44	1.44	1.68	1.00	0.71	0.44	0.46	0.46	0.53
	1.73	(SD)	(0.59)	(0.81)	(0.88)	(0.82)	(0.82)	(0.83)	(0.03)	(0.45)	(0.50)	(0.50)	(0.50)	(0.50)
		(Mean SE)	(0.61)	(0.79)	(0.86)	(0.78)	(0.78)	(0.83)	*NA*	*NA*	*NA*	*NA*	*NA*	*NA*
			0.01	−1.20	−1.42	−1.39	−1.39	−1.31	0.04	0.75	0.86	0.85	0.85	0.81
	−1.21	(SD)	(0.35)	(0.48)	(0.49)	(0.48)	(0.48)	(0.48)	(0.20)	(0.43)	(0.35)	(0.35)	(0.35)	(0.39)
		(Mean SE)	(0.36)	(0.46)	(0.47)	(0.46)	(0.46)	(0.46)	*NA*	*NA*	*NA*	*NA*	*NA*	*NA*
0.5			0.34	0.09	0.18	0.11	0.11	0.14	0.09	0.05	0.06	0.06	0.06	0.06
	0.00	(SD)	(0.56)	(0.81)	(0.81)	(0.79)	(0.79)	(0.80)	(0.28)	(0.22)	(0.23)	(0.23)	(0.23)	(0.23)
		(Mean SE)	(0.58)	(0.80)	(0.78)	(0.77)	(0.77)	(0.79)	*NA*	*NA*	*NA*	*NA*	*NA*	*NA*
			−1.99	−0.14	−0.17	−0.18	−0.18	−0.15	1.00	0.07	0.12	0.13	0.13	0.08
	0.00	(SD)	(0.14)	(0.20)	(0.18)	(0.18)	(0.18)	(0.18)	(0.00)	(0.25)	(0.33)	(0.34)	(0.34)	(0.28)
		(Mean SE)	(0.14)	(0.22)	(0.20)	(0.20)	(0.20)	(0.21)	*NA*	*NA*	*NA*	*NA*	*NA*	*NA*
			−0.42	−0.33	−0.26	−0.32	−0.32	−0.29	0.12	0.09	0.08	0.09	0.09	0.09
	0.14	(SD)	(0.52)	(0.76)	(0.76)	(0.75)	(0.75)	(0.76)	(0.32)	(0.29)	(0.28)	(0.29)	(0.29)	(0.28)
		(Mean SE)	(0.53)	(0.73)	(0.71)	(0.70)	(0.70)	(0.71)	*NA*	*NA*	*NA*	*NA*	*NA*	*NA*
			0.51	0.07	0.06	0.08	0.08	0.06	0.38	0.06	0.07	0.07	0.07	0.07
	0.42	(SD)	(0.31)	(0.45)	(0.46)	(0.45)	(0.45)	(0.45)	(0.49)	(0.24)	(0.25)	(0.25)	(0.25)	(0.25)
		(Mean SE)	(0.31)	(0.43)	(0.42)	(0.41)	(0.41)	(0.42)	*NA*	*NA*	*NA*	*NA*	*NA*	*NA*
-0.5			−0.78	−0.16	−0.11	−0.14	−0.14	−0.16	0.23	0.06	0.07	0.07	0.07	0.07
	0.00	(SD)	(0.65)	(0.90)	(0.88)	(0.91)	(0.91)	(0.89)	(0.42)	(0.23)	(0.25)	(0.26)	(0.26)	(0.26)
		(Mean SE)	(0.65)	(0.89)	(0.84)	(0.86)	(0.86)	(0.85)	*NA*	*NA*	*NA*	*NA*	*NA*	*NA*
			1.49	0.20	−0.06	0.16	0.16	0.19	1.00	0.05	0.03	0.07	0.07	0.06
	0.00	(SD)	(0.16)	(0.21)	(0.22)	(0.21)	(0.21)	(0.20)	(0.00)	(0.21)	(0.16)	(0.26)	(0.26)	(0.24)
		(Mean SE)	(0.16)	(0.27)	(0.27)	(0.23)	(0.23)	(0.26)	*NA*	*NA*	*NA*	*NA*	*NA*	*NA*
			2.57	2.78	3.41	2.82	2.82	2.83	0.99	0.91	0.96	0.93	0.93	0.92
	2.57	(SD)	(0.59)	(0.87)	(0.90)	(0.87)	(0.87)	(0.86)	(0.07)	(0.29)	(0.19)	(0.26)	(0.26)	(0.27)
		(Mean SE)	(0.60)	(0.83)	(0.88)	(0.81)	(0.81)	(0.83)	*NA*	*NA*	*NA*	*NA*	*NA*	*NA*
			−1.02	0.37	0.18	0.38	0.38	0.34	0.86	0.11	0.06	0.14	0.14	0.12
	0.27	(SD)	(0.33)	(0.48)	(0.47)	(0.48)	(0.48)	(0.48)	(0.35)	(0.32)	(0.23)	(0.35)	(0.35)	(0.33)
		(Mean SE)	(0.35)	(0.49)	(0.48)	(0.47)	(0.47)	(0.47)	*NA*	*NA*	*NA*	*NA*	*NA*	*NA*

Table Y. Mean effect estimates, empirical type-I error rate (for those SNPs with true effect 0 and underlined) and power with and without bias correction for some representative SNPs in the presence of 50% invalid IVs. Sample standard deviations (SD) and mean standard errors (Mean SE) are given in parenthesis. $p_2 = 4$.

ρ	True effects		Mean effect estimates						Type-I error rate or power					
			No	cML	Egger	IVW	Lasso	Median	No	cML	Egger	IVW	Lasso	Median
0	<u>0.00</u>		0.04	−0.08	−0.57	−0.50	−0.50	−0.26	0.05	0.07	0.21	0.20	0.20	0.11
		(SD)	(0.40)	(0.58)	(0.65)	(0.64)	(0.64)	(0.62)	(0.22)	(0.25)	(0.41)	(0.40)	(0.40)	(0.32)
		(Mean SE)	(0.41)	(0.56)	(0.56)	(0.54)	(0.54)	(0.53)	*NA*	*NA*	*NA*	*NA*	*NA*	*NA*
	<u>0.00</u>		0.91	−0.09	−0.05	−0.07	−0.07	−0.06	0.92	0.04	0.06	0.05	0.05	0.02
		(SD)	(0.19)	(0.26)	(0.27)	(0.26)	(0.26)	(0.26)	(0.26)	(0.20)	(0.23)	(0.22)	(0.22)	(0.13)
		(Mean SE)	(0.14)	(0.24)	(0.21)	(0.20)	(0.20)	(0.22)	*NA*	*NA*	*NA*	*NA*	*NA*	*NA*
	0.50		1.87	1.16	0.85	0.90	0.90	0.98	0.93	0.65	0.42	0.46	0.46	0.52
		(SD)	(0.77)	(0.57)	(0.61)	(0.60)	(0.60)	(0.57)	(0.25)	(0.48)	(0.49)	(0.50)	(0.50)	(0.50)
		(Mean SE)	(0.37)	(0.50)	(0.50)	(0.48)	(0.49)	(0.48)	*NA*	*NA*	*NA*	*NA*	*NA*	*NA*
	−1.85		−2.59	−2.14	−1.90	−1.91	−1.91	−2.06	1.00	1.00	1.00	1.00	1.00	1.00
		(SD)	(0.24)	(0.35)	(0.37)	(0.37)	(0.37)	(0.38)	(0.00)	(0.00)	(0.00)	(0.00)	(0.00)	(0.00)
		(Mean SE)	(0.22)	(0.30)	(0.30)	(0.29)	(0.29)	(0.29)	*NA*	*NA*	*NA*	*NA*	*NA*	*NA*
0.5	<u>0.00</u>		−2.59	0.01	−0.62	−0.64	−0.64	0.00	1.00	0.13	0.32	0.33	0.33	0.09
		(SD)	(0.40)	(0.83)	(0.58)	(0.58)	(0.58)	(0.61)	(0.00)	(0.34)	(0.46)	(0.47)	(0.47)	(0.29)
		(Mean SE)	(0.37)	(0.57)	(0.49)	(0.48)	(0.48)	(0.53)	*NA*	*NA*	*NA*	*NA*	*NA*	*NA*
	<u>0.00</u>		0.84	0.01	0.15	0.16	0.16	0.05	0.97	0.07	0.21	0.26	0.26	0.08
		(SD)	(0.19)	(0.25)	(0.26)	(0.26)	(0.26)	(0.26)	(0.16)	(0.26)	(0.41)	(0.44)	(0.44)	(0.26)
		(Mean SE)	(0.13)	(0.18)	(0.18)	(0.17)	(0.17)	(0.18)	*NA*	*NA*	*NA*	*NA*	*NA*	*NA*
	−0.50		1.82	−0.03	0.50	0.48	0.48	−0.03	0.99	0.14	0.31	0.32	0.32	0.17
		(SD)	(0.58)	(0.61)	(0.59)	(0.61)	(0.61)	(0.64)	(0.10)	(0.34)	(0.46)	(0.47)	(0.47)	(0.38)
		(Mean SE)	(0.33)	(0.51)	(0.44)	(0.43)	(0.43)	(0.48)	*NA*	*NA*	*NA*	*NA*	*NA*	*NA*
	−0.29		−1.24	−0.32	−0.37	−0.39	−0.39	−0.38	1.00	0.17	0.33	0.34	0.34	0.28
		(SD)	(0.21)	(0.32)	(0.33)	(0.30)	(0.30)	(0.32)	(0.00)	(0.37)	(0.47)	(0.47)	(0.47)	(0.45)
		(Mean SE)	(0.20)	(0.32)	(0.26)	(0.26)	(0.26)	(0.29)	*NA*	*NA*	*NA*	*NA*	*NA*	*NA*
-0.5	<u>0.00</u>		0.10	−0.49	−0.12	−0.30	−0.30	−0.43	0.25	0.16	0.11	0.14	0.13	0.15
		(SD)	(0.59)	(0.55)	(0.62)	(0.60)	(0.60)	(0.55)	(0.43)	(0.37)	(0.31)	(0.34)	(0.34)	(0.36)
		(Mean SE)	(0.35)	(0.52)	(0.51)	(0.49)	(0.49)	(0.50)	*NA*	*NA*	*NA*	*NA*	*NA*	*NA*
	<u>0.00</u>		0.38	−0.16	−0.32	−0.28	−0.28	−0.16	0.81	0.14	0.54	0.42	0.41	0.05
		(SD)	(0.22)	(0.43)	(0.34)	(0.31)	(0.31)	(0.34)	(0.39)	(0.35)	(0.50)	(0.49)	(0.49)	(0.22)
		(Mean SE)	(0.17)	(0.23)	(0.24)	(0.23)	(0.23)	(0.27)	*NA*	*NA*	*NA*	*NA*	*NA*	*NA*
	0.01		−1.55	−0.22	0.30	0.09	0.09	−0.15	0.96	0.17	0.22	0.14	0.14	0.12
		(SD)	(0.68)	(0.56)	(0.56)	(0.54)	(0.54)	(0.53)	(0.20)	(0.38)	(0.41)	(0.35)	(0.35)	(0.32)
		(Mean SE)	(0.30)	(0.45)	(0.45)	(0.43)	(0.43)	(0.43)	*NA*	*NA*	*NA*	*NA*	*NA*	*NA*
	1.18		0.13	0.92	0.95	0.90	0.90	0.99	0.23	0.66	0.90	0.91	0.90	0.85
		(SD)	(0.26)	(0.50)	(0.31)	(0.32)	(0.32)	(0.34)	(0.42)	(0.47)	(0.29)	(0.29)	(0.29)	(0.36)
		(Mean SE)	(0.19)	(0.33)	(0.29)	(0.28)	(0.28)	(0.34)	*NA*	*NA*	*NA*	*NA*	*NA*	*NA*

F.3.4 Figures of point estimates

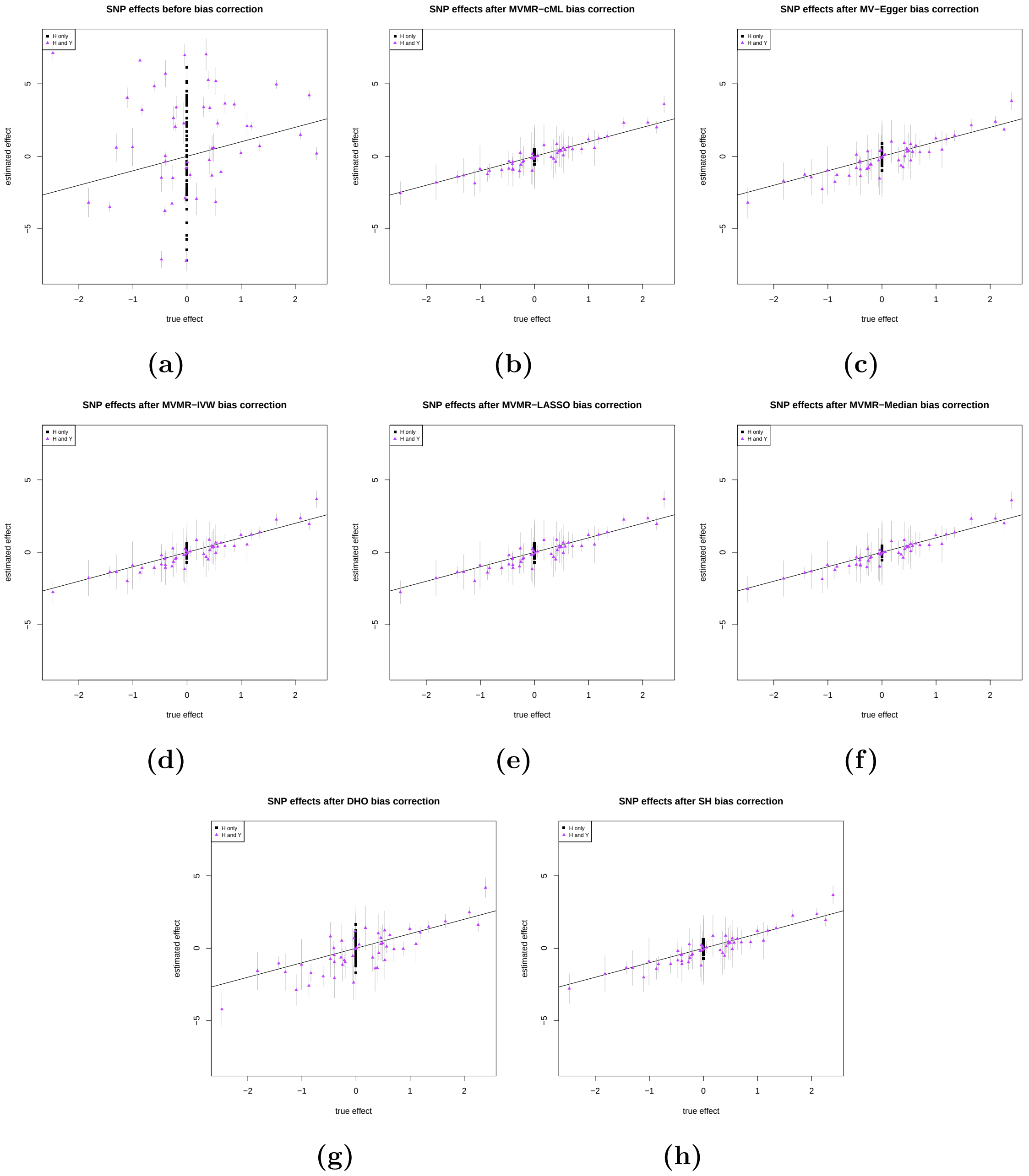

Fig Q. Mean estimates of the effects of SNPs having collider bias with $\rho = 0$, $p_2 = 1$ and 50% invalid IVs. Horizontal coordinates are for the true effects, vertical coordinates are for the estimated effects. Vertical bars are the means of standard errors averaged over 1000 repetitions. In the legends, "$\boldsymbol{H}$ only" means the SNPs affecting only the covariates; "$\boldsymbol{H}$ and Y" means the SNPs affecting both the covariates and outcome.

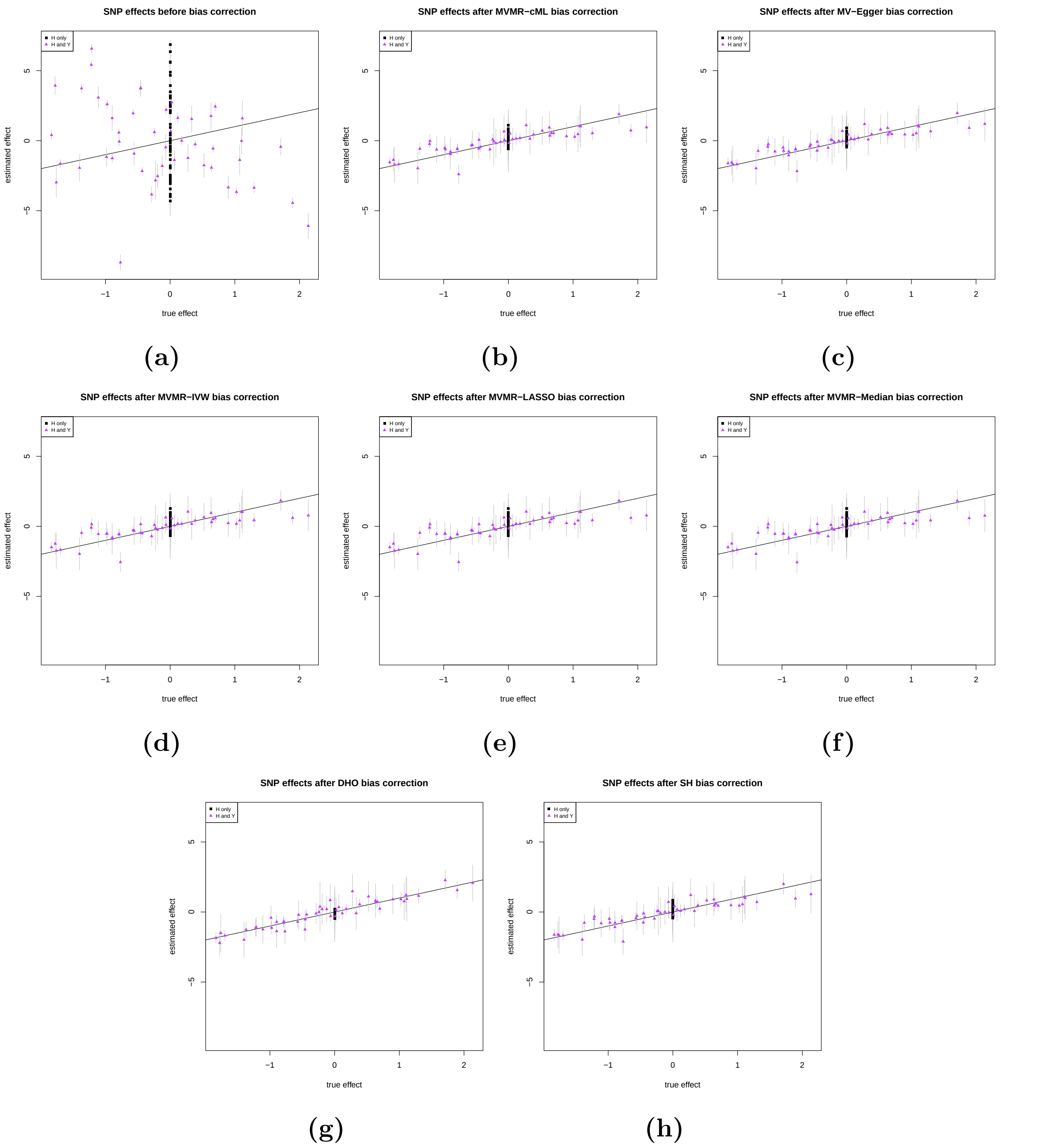

Fig R. Mean estimates of the effects of SNPs having collider bias with $\rho = 0.5$, $p_2 = 1$ and 50% invalid IVs. Horizontal coordinates are for the true effects, vertical coordinates are for the estimated effects. Vertical bars are the means of standard errors averaged over 1000 repetitions. In the legends, "$\boldsymbol{H}$ only" means the SNPs affecting only the covariates; "$\boldsymbol{H}$ and Y" means the SNPs affecting both the covariates and outcome.

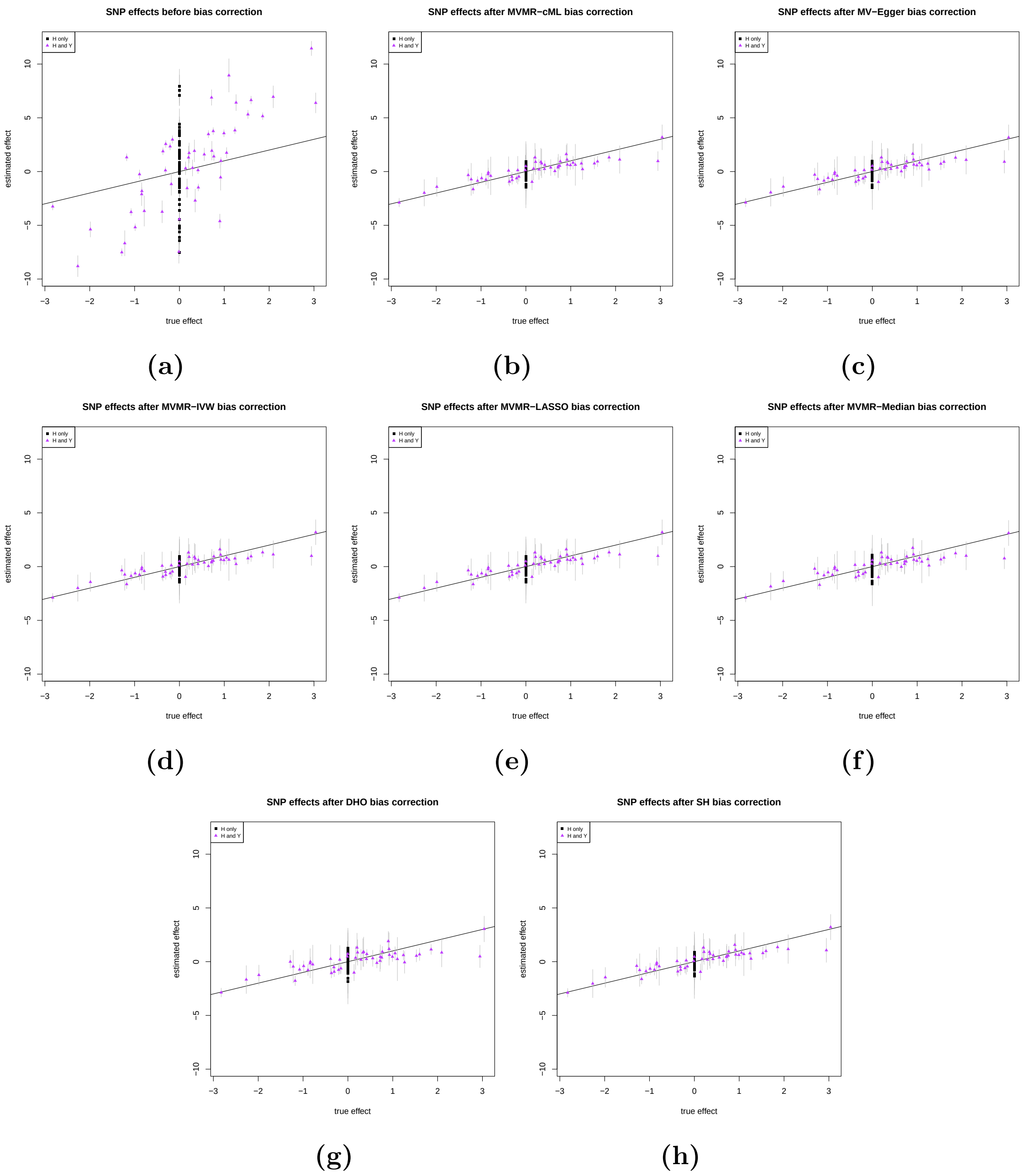

Fig S. Mean estimates of the effects of SNPs having collider bias with $\rho = -0.5$, $p_2 = 1$ and 50% invalid IVs. Horizontal coordinates are for the true effects, vertical coordinates are for the estimated effects. Vertical bars are the means of standard errors averaged over 1000 repetitions. In the legends, "$\boldsymbol{H}$ only" means the SNPs affecting only the covariates; "$\boldsymbol{H}$ and Y" means the SNPs affecting both the covariates and outcome.

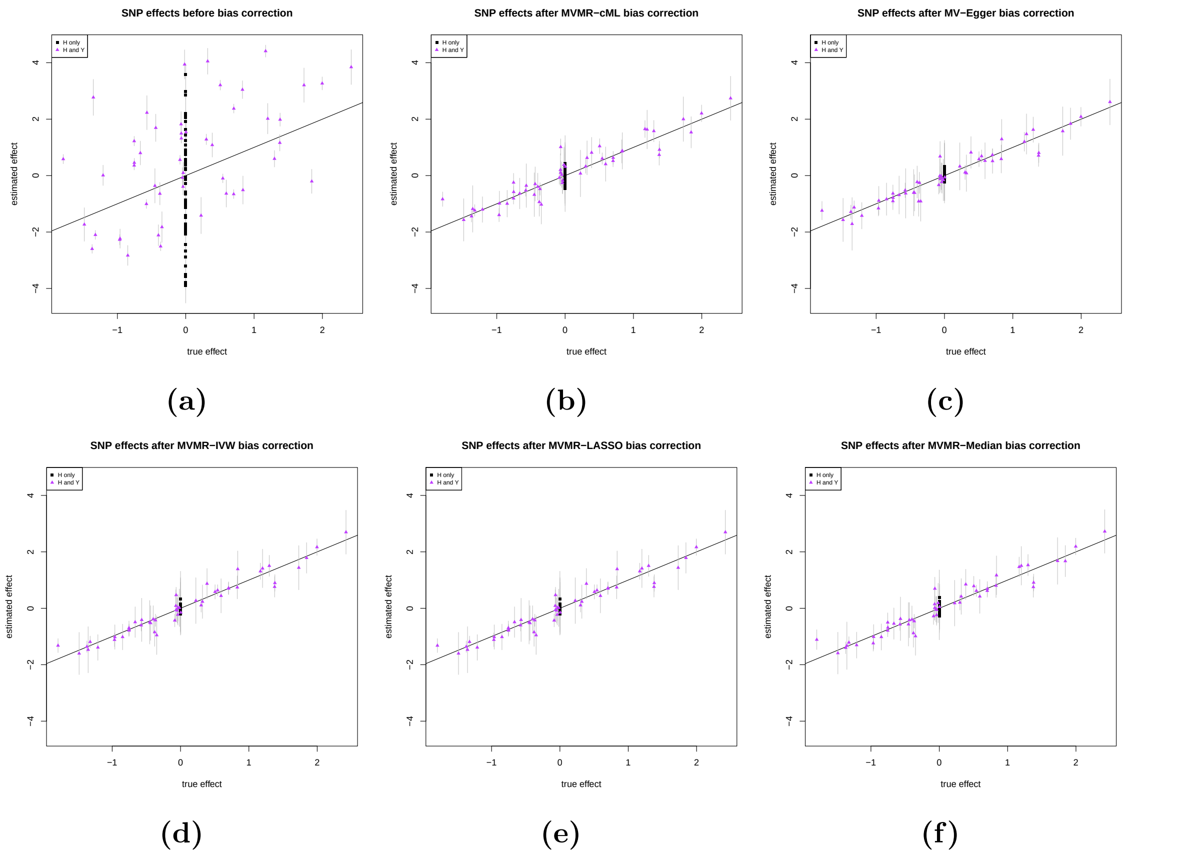

Fig T. Mean estimates of the effects of SNPs having collider bias with $\rho = 0$, $p_2 = 2$ and 50% invalid IVs. Horizontal coordinates are for the true effects, vertical coordinates are for the estimated effects. Vertical bars are the means of standard errors averaged over 1000 repetitions. In the legends, "$\boldsymbol{H}$ only" means the SNPs affecting only the covariates; "$\boldsymbol{H}$ and Y" means the SNPs affecting both the covariates and outcome.

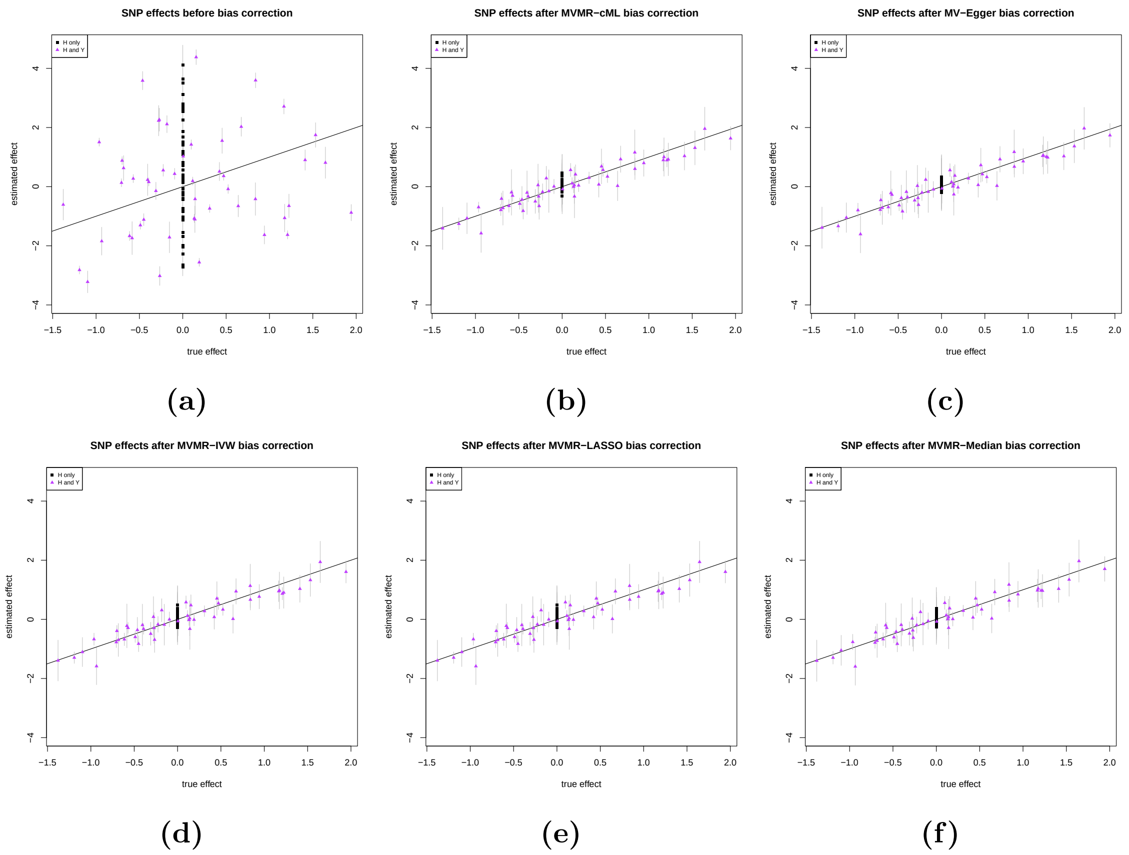

Fig U. Mean estimates of the effects of SNPs having collider bias with $\rho = 0.5$, $p_2 = 2$ and 50% invalid IVs. Horizontal coordinates are for the true effects, vertical coordinates are for the estimated effects. Vertical bars are the means of standard errors averaged over 1000 repetitions. In the legends, "$\boldsymbol{H}$ only" means the SNPs affecting only the covariates; "$\boldsymbol{H}$ and Y" means the SNPs affecting both the covariates and outcome.

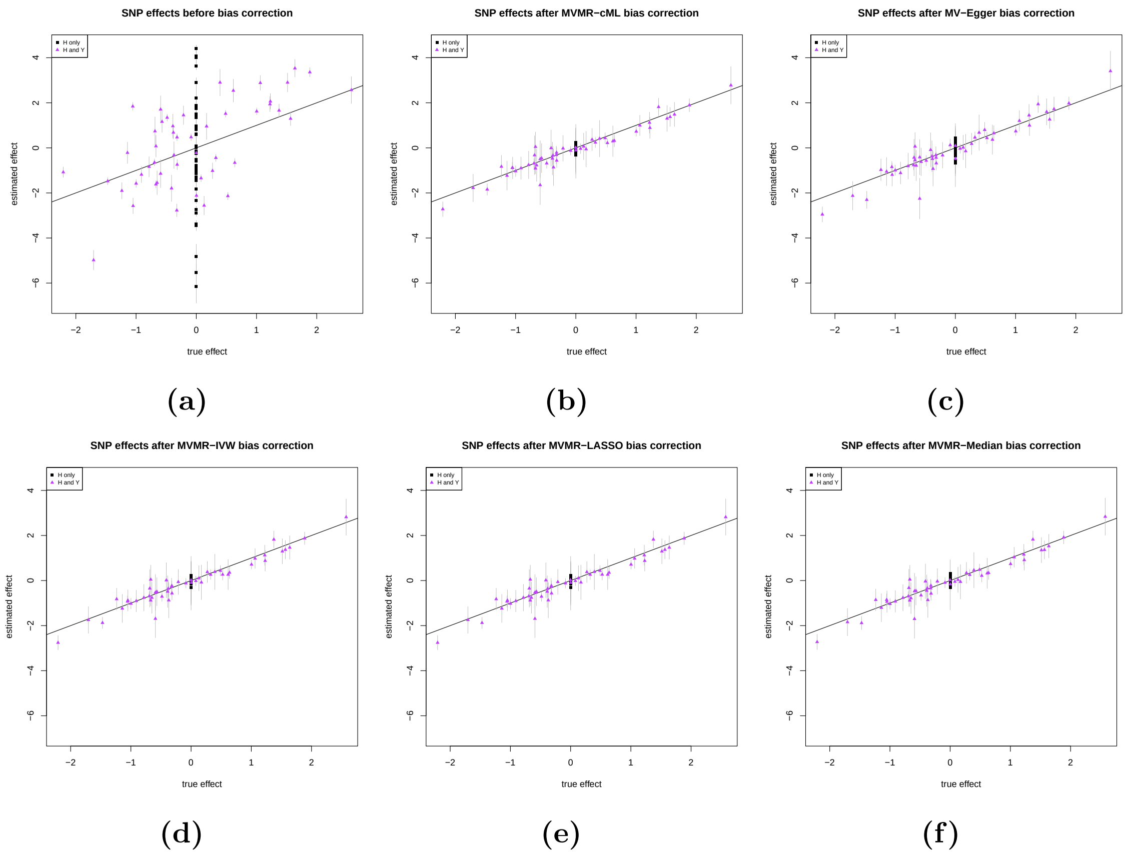

Fig V. Mean estimates of the effects of SNPs having collider bias with $\rho = -0.5$, $p_2 = 2$ and 50% invalid IVs. Horizontal coordinates are for the true effects, vertical coordinates are for the estimated effects. Vertical bars are the means of standard errors averaged over 1000 repetitions. In the legends, "$\boldsymbol{H}$ only" means the SNPs affecting only the covariates; "$\boldsymbol{H}$ and Y" means the SNPs affecting both the covariates and outcome.

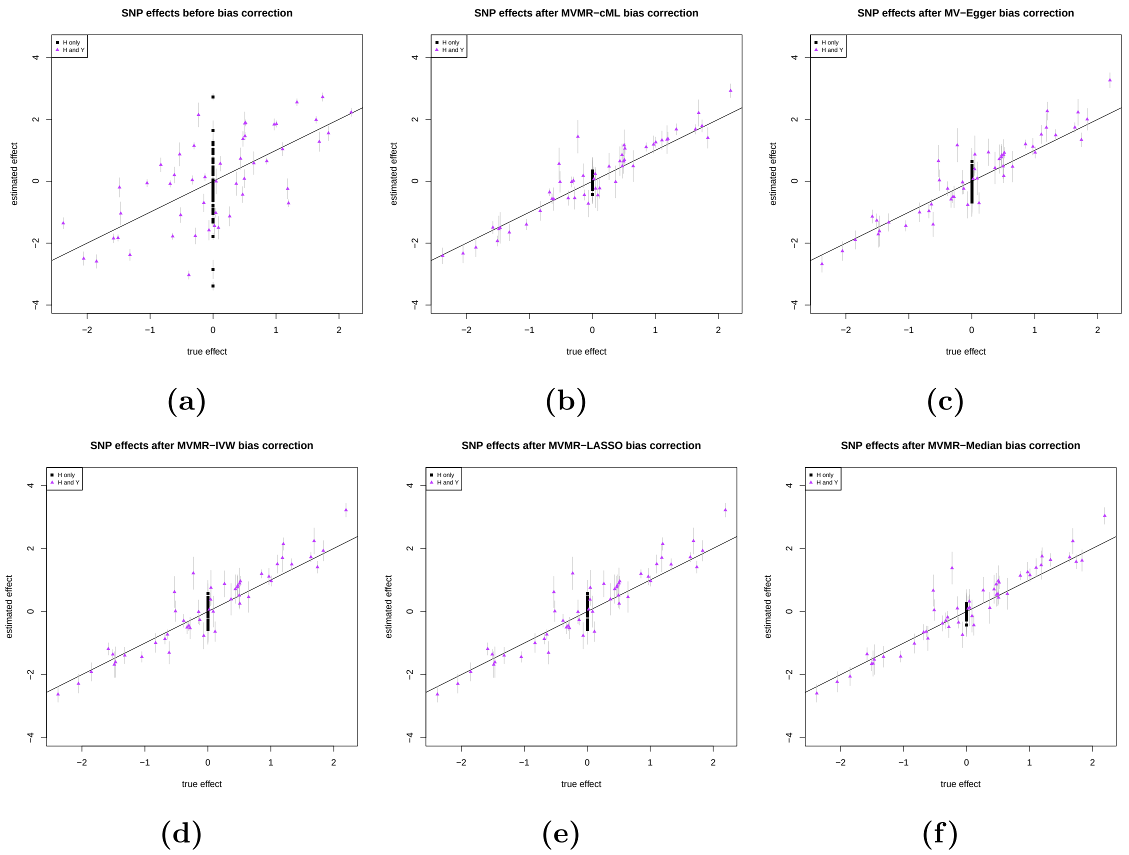

Fig W. Mean estimates of the effects of SNPs having collider bias with $\rho = 0$, $p_2 = 4$ and 50% invalid IVs. Horizontal coordinates are for the true effects, vertical coordinates are for the estimated effects. Vertical bars are the means of standard errors averaged over 1000 repetitions. In the legends, "$\boldsymbol{H}$ only" means the SNPs affecting only the covariates; "$\boldsymbol{H}$ and Y" means the SNPs affecting both the covariates and outcome.

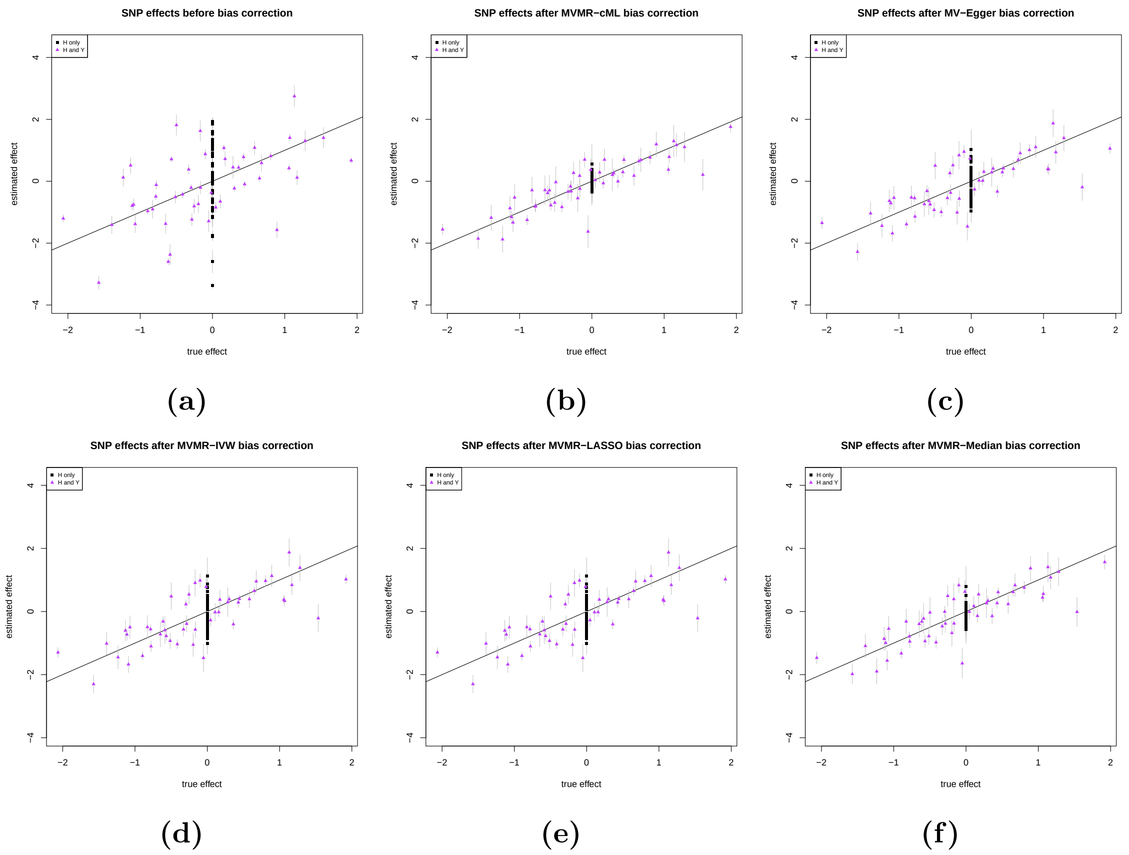

Fig X. Mean estimates of the effects of SNPs having collider bias with $\rho = 0.5$, $p_2 = 4$ and 50% invalid IVs. Horizontal coordinates are for the true effects, vertical coordinates are for the estimated effects. Vertical bars are the means of standard errors averaged over 1000 repetitions. In the legends, "$\boldsymbol{H}$ only" means the SNPs affecting only the covariates; "$\boldsymbol{H}$ and Y" means the SNPs affecting both the covariates and outcome.

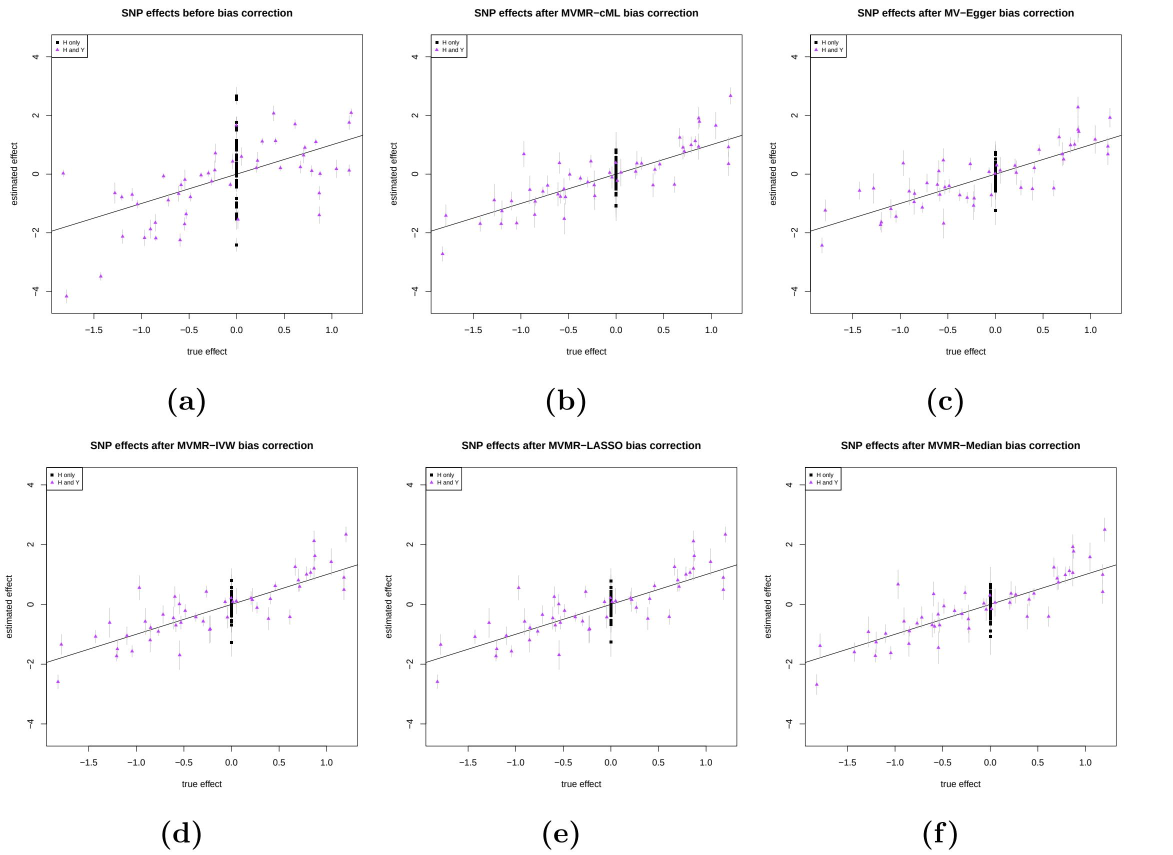

Fig Y. Mean estimates of the effects of SNPs having collider bias with $\rho = -0.5$, $p_2 = 4$ and 50% invalid IVs. Horizontal coordinates are for the true effects, vertical coordinates are for the estimated effects. Vertical bars are the means of standard errors averaged over 1000 repetitions. In the legends, "$\boldsymbol{H}$ only" means the SNPs affecting only the covariates; "$\boldsymbol{H}$ and Y" means the SNPs affecting both the covariates and outcome.

G Real GWAS data

G.1 GWAS of WHR adjusted for BMI

G.1.1 Tables of significant SNPs and loci

Table Z. Point estimates of $\boldsymbol{b}$ and number of significant loci obtained by applying different MVMR methods. Standard errors (SE) are given in parenthesis.

	No correction	MVMR-cML	MVMR-Egger	MVMR-IVW	MVMR-Median	MVMR-Lasso	SH	DHO
$\hat{\boldsymbol{b}}$	NA	−0.079	0.047	−0.058	−0.063	−0.063	−0.11	−0.068
(SE)	NA	(0.001)	(0.322)	(0.003)	(0.004)	(0.003)	(0.002)	(0.013)
# of significant SNPs	315	269	11	260	260	260	209	229
# of significant loci	42	34	2	42	42	42	37	39

G.1.2 Comparison of SNP effect estimates before and after apply different bias-correction methods

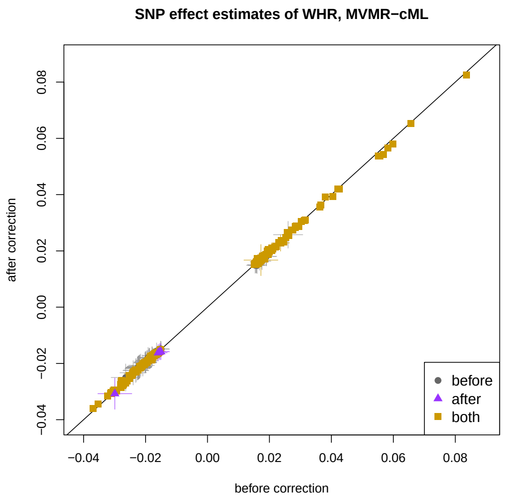

(a)

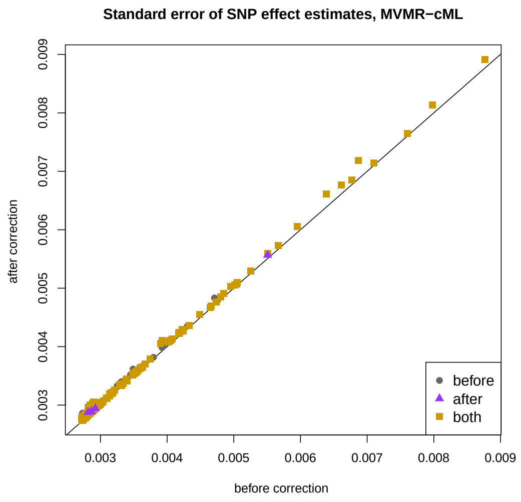

(b)

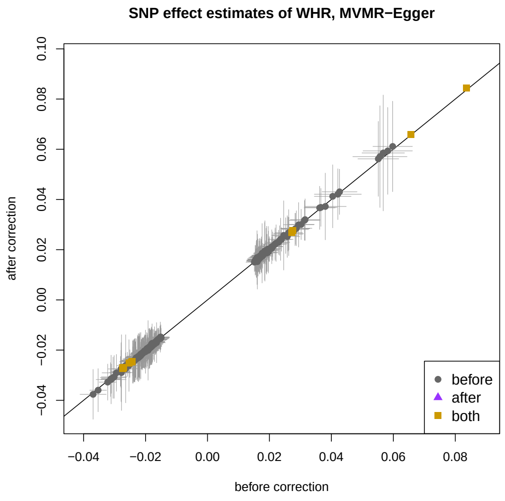

(c)

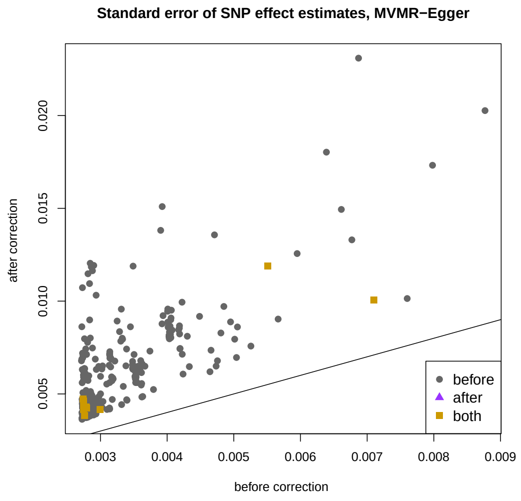

(d)

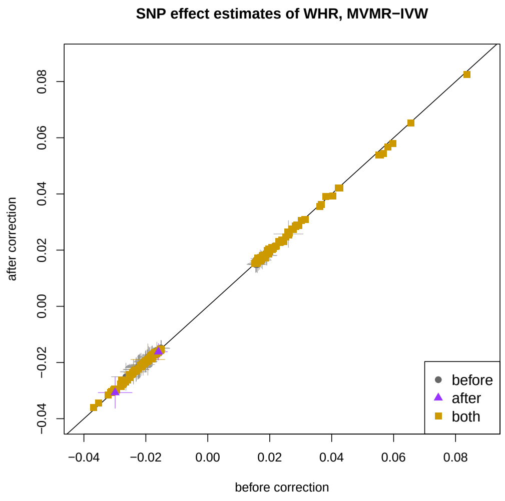

(e)

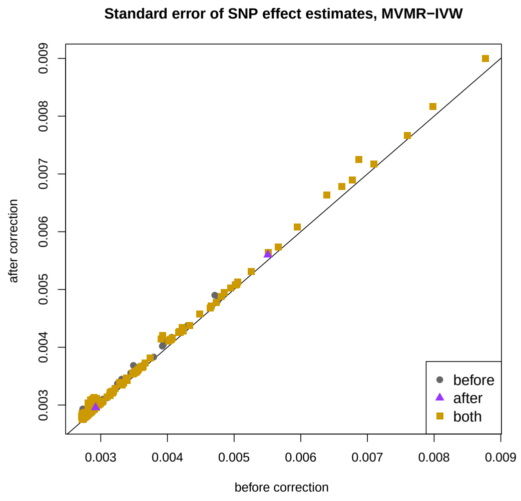

(f)

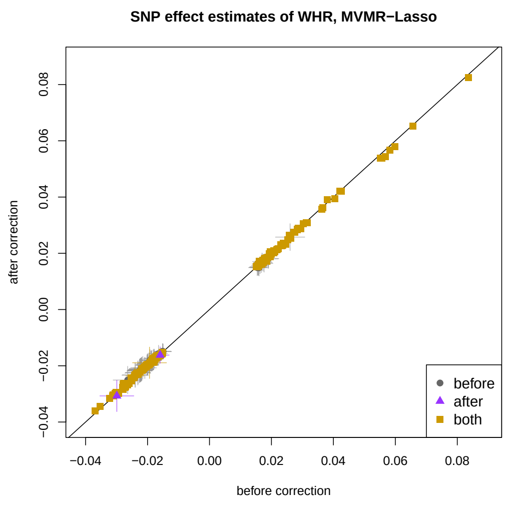
SNP effect estimates of WHR, MVMR−Lasso
after correction
before correction
before
after
both

(g)

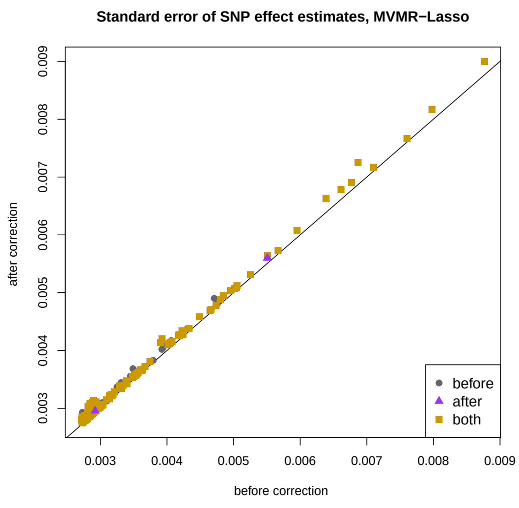
Standard error of SNP effect estimates, MVMR−Lasso
after correction
before correction
before
after
both

(h)

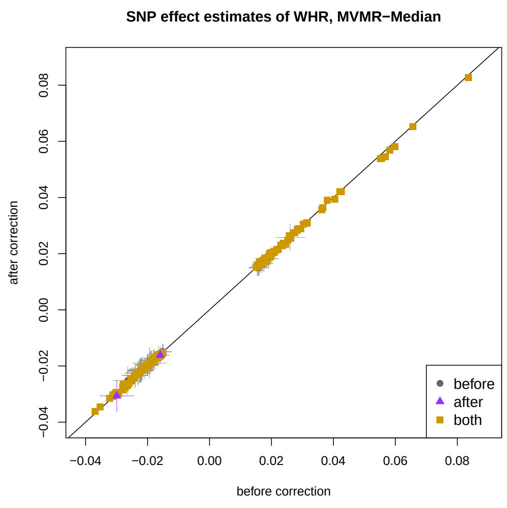
SNP effect estimates of WHR, MVMR−Median
after correction
before correction
before
after
both

(i)

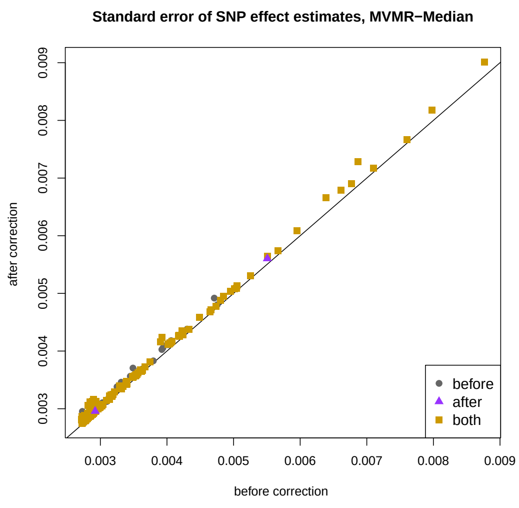
Standard error of SNP effect estimates, MVMR−Median
after correction
before correction
before
after
both

(j)

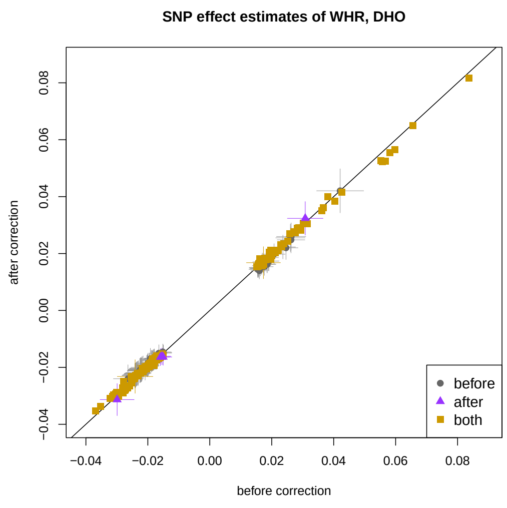
SNP effect estimates of WHR, DHO
after correction
before correction
before
after
both

(k)

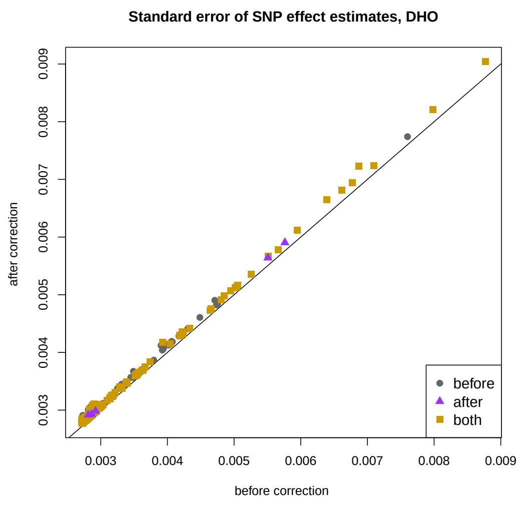
Standard error of SNP effect estimates, DHO
after correction
before correction
before
after
both

(l)

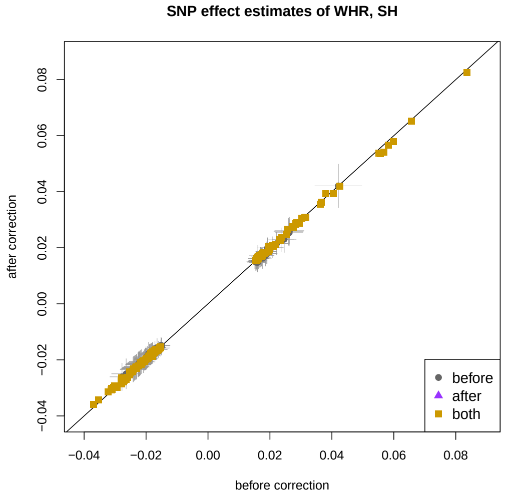

(m)

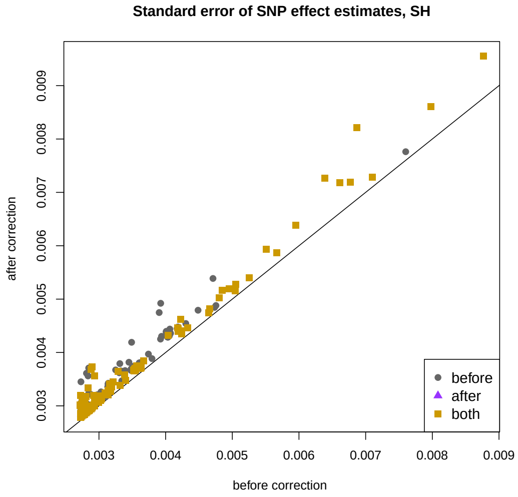

(n)

Fig Z. Effect estimates of WHR before and after bias correction. Horizontal and vertical bars represent 1 SE of an estimate before and after correction respectively. SEs are given in the right column. In the legends, "before" refers to the SNPs that were significant only before applying bias correction, "after" refers to the SNPs significant only after bias correction, "both" refers to the SNPs significant both before and after bias correction.

G.1.3 Comparison of Manhattan plots before and after apply different bias-correction methods

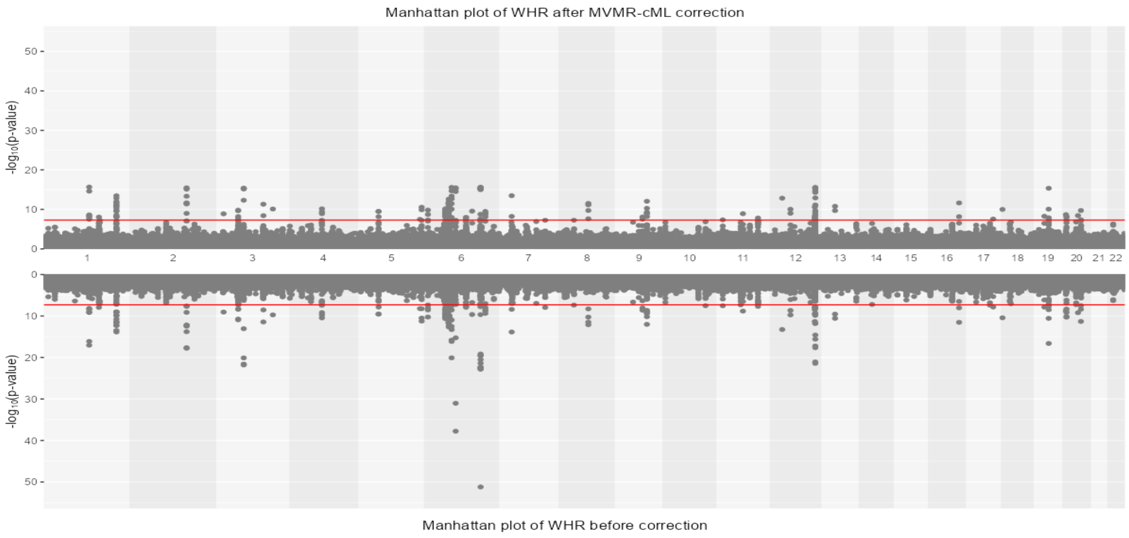

(a)

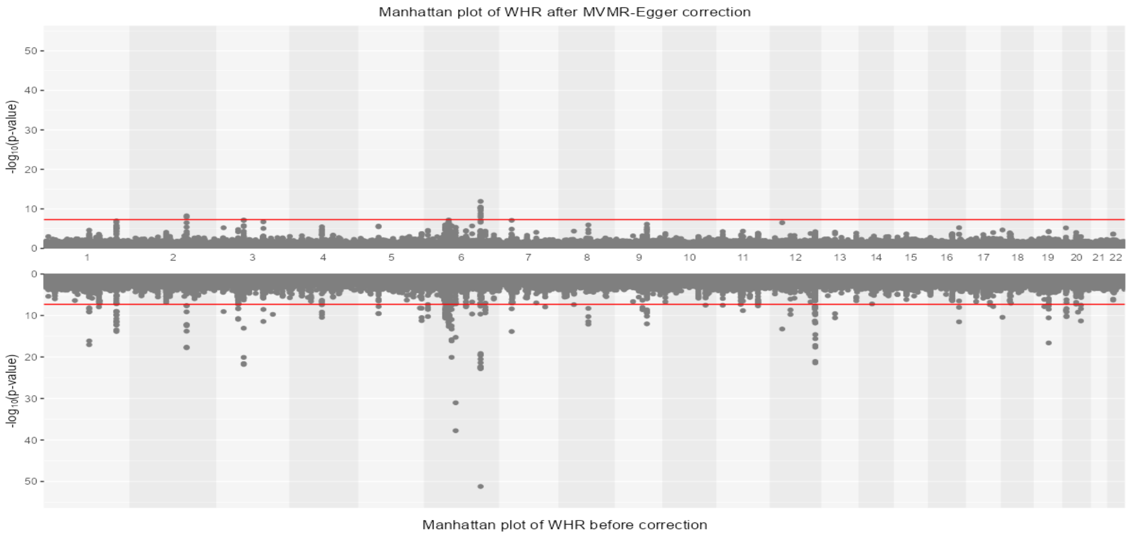

(b)

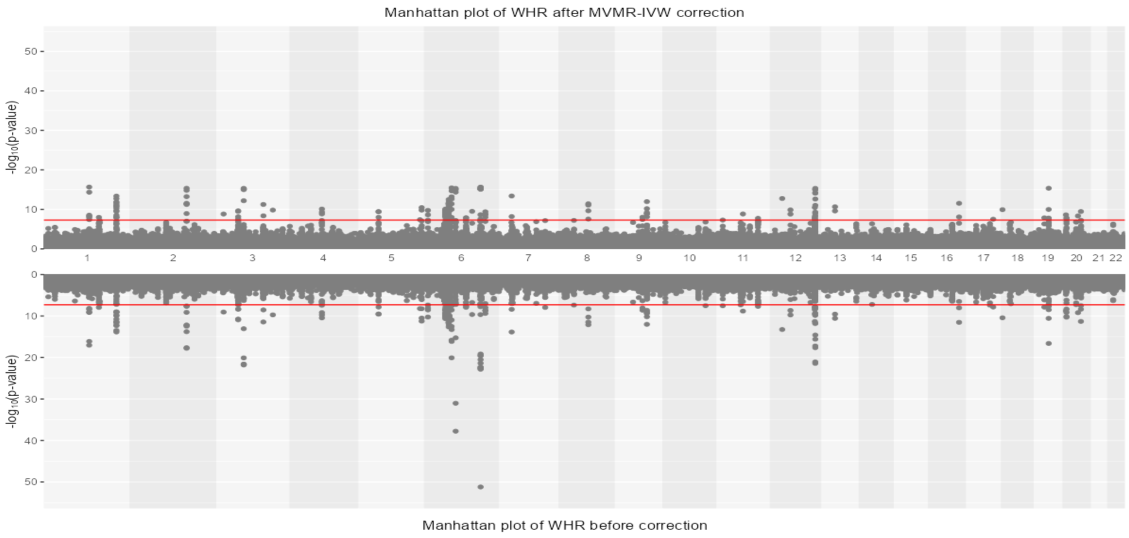

(c)

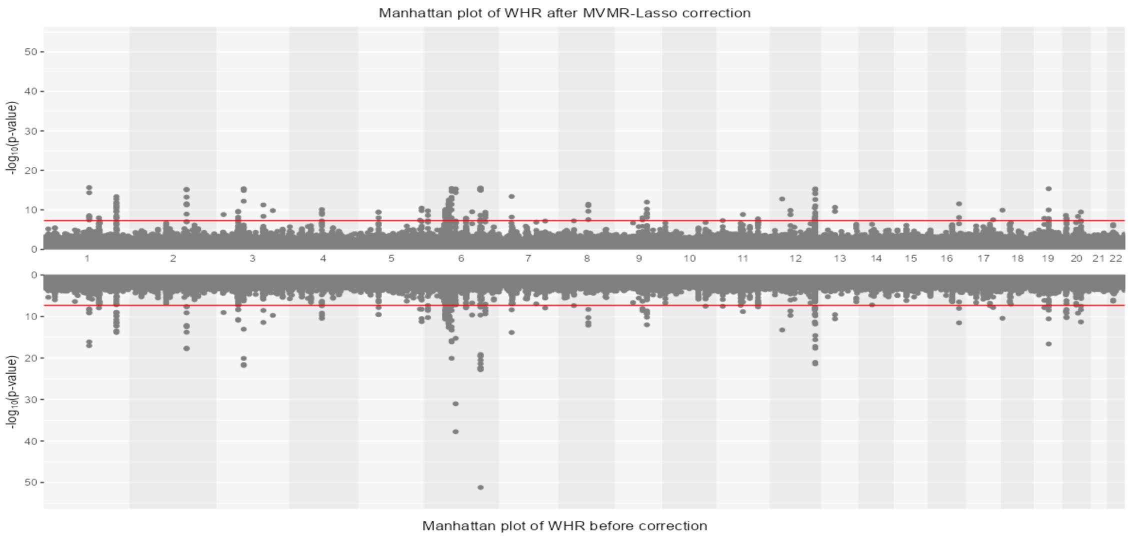
Manhattan plot of WHR after MVMR-Lasso correction
-log10(p-value)
1 2 3 4 5 6 7 8 9 10 11 12 13 14 15 16 17 18 19 20 21 22
Manhattan plot of WHR before correction

(d)

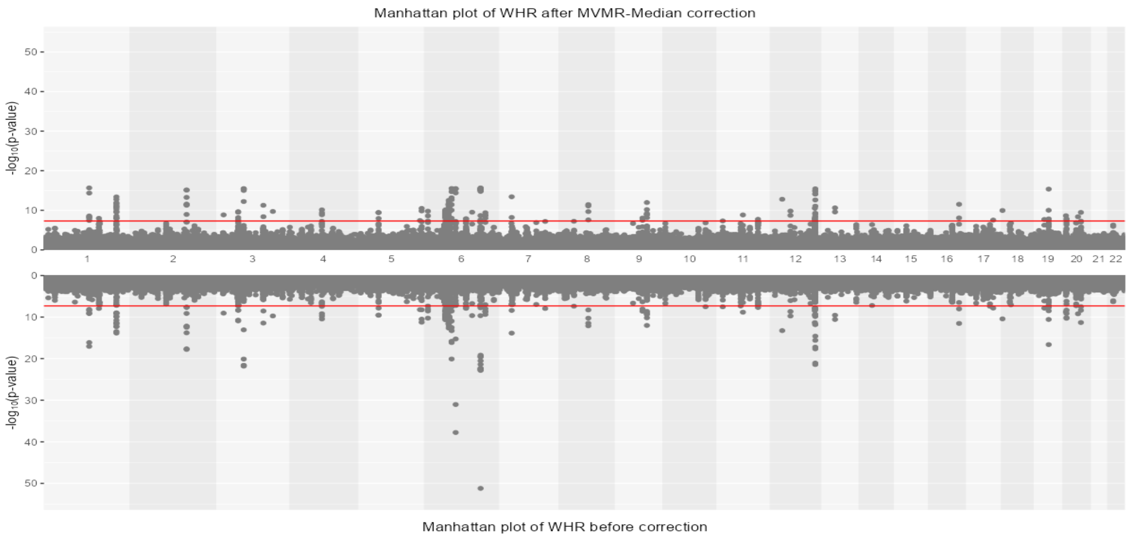
Manhattan plot of WHR after MVMR-Median correction
-log10(p-value)
1 2 3 4 5 6 7 8 9 10 11 12 13 14 15 16 17 18 19 20 21 22
Manhattan plot of WHR before correction

(e)

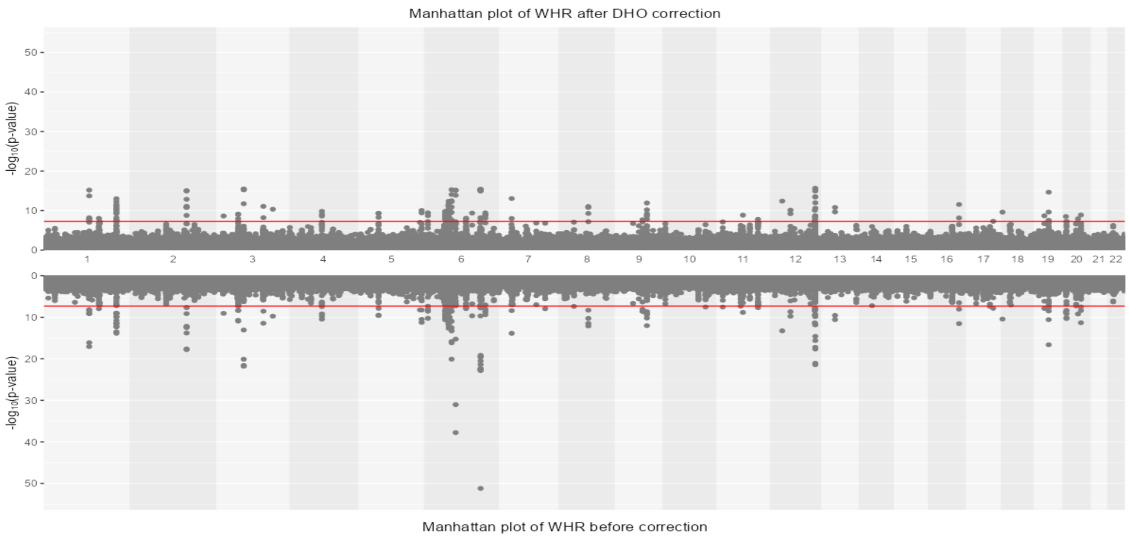
Manhattan plot of WHR after DHO correction
-log10(p-value)
1 2 3 4 5 6 7 8 9 10 11 12 13 14 15 16 17 18 19 20 21 22
Manhattan plot of WHR before correction

(f)

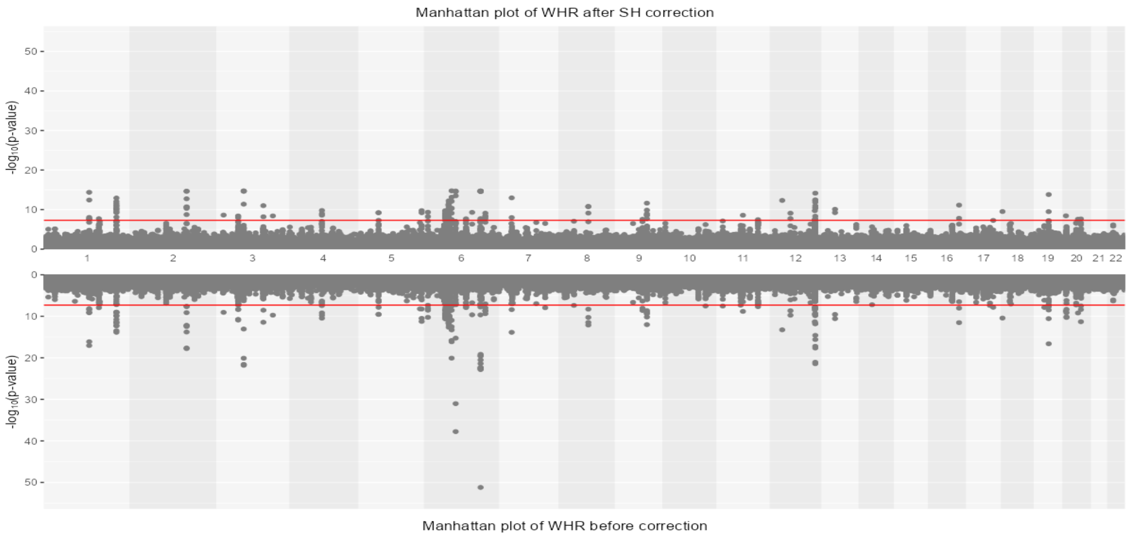

(g)

Fig AA. Manhattan plot of WHR before (upper panel) and after (lower panel) applying bias correction.

G.1.4 Comparison of QQ plots before and after apply different bias-correction methods

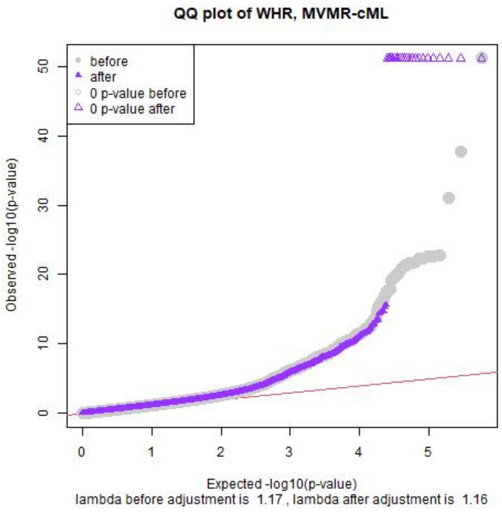

(a)

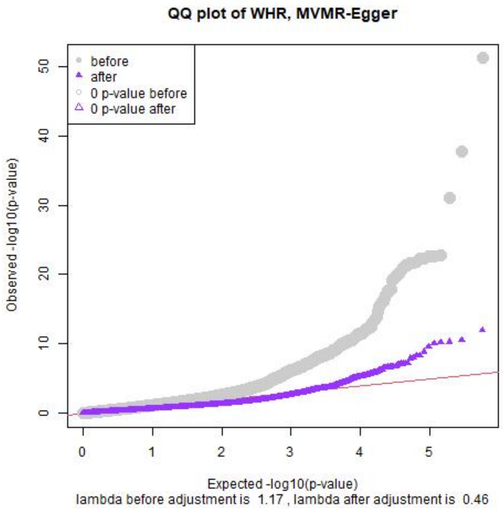

(b)

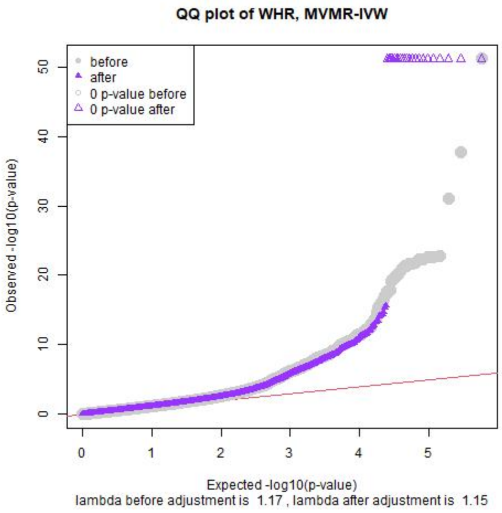

(c)

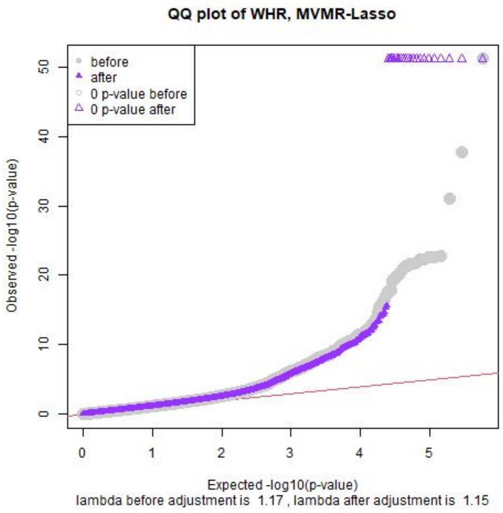

(d)

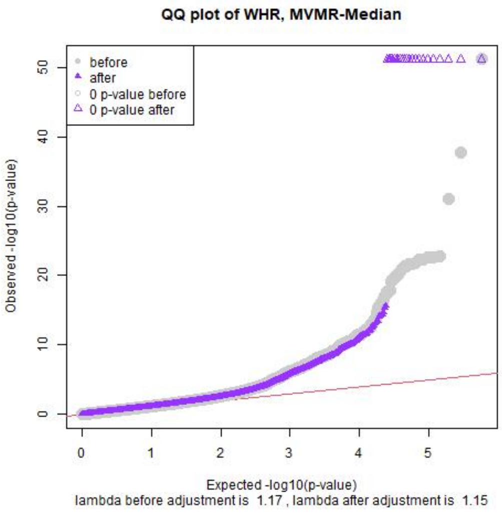

(e)

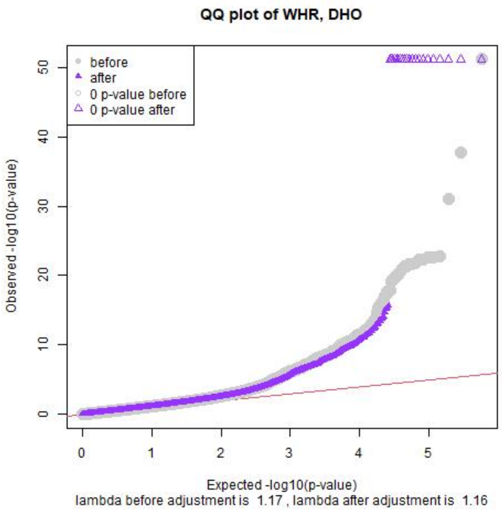

(f)

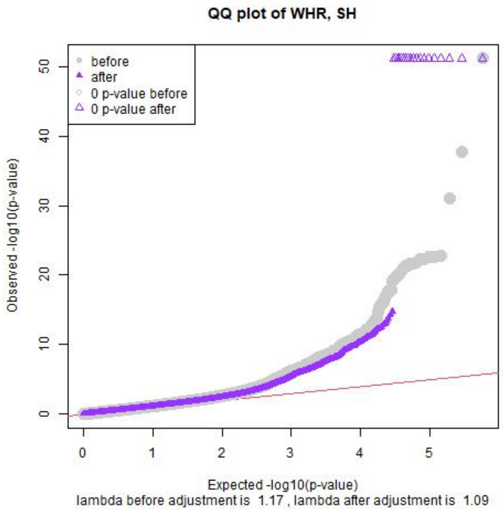

(g)

Fig AB. QQ plots of WHR. In the legends, "before" means the result before correction, "after" means the result after correction. "0 p-value before" represents the SNPs having a p-value of 0 before correction. "0 p-value after" represents the SNPs having a p-value of 0 after correction. The SNPs having p-values of 0 before or after correction are truncated at the top of plots.

G.2 GWAS of BMI adjusted for metabolomic PCs

G.2.1 Results after removing genetic components

In order to remove the genetic components from metabolomic PCs, we computed the polygenic risk scores (PRS) of the standardized metabolomic PCs with the continuous shrinkage prior (PRS-CS) model [8]. Using the UKB European sample as the LD reference panel [8], we obtained posterior SNP effect estimates. Then we calculated the genetic components as the sum of genetic markers across the genome, weighted by the posterior SNP effect estimates. Next, we subtracted the genetic components from standardized metabolomic PCs and utilized the resulting residuals as the covariates, denoted as $\boldsymbol{H}^*$. The covariates in $\boldsymbol{H}^*$ captured the environmental components represented by the metabolomic variables [9]. Subsequently, we conducted the GWAS of BMI using the following Equation [9]:

$$Y = \beta^*_{G_iY} G_i + \boldsymbol{J}^T \boldsymbol{\beta}^*_{G_i\boldsymbol{J}} + \boldsymbol{H}^{*T} \boldsymbol{\beta}^*_{G_i\boldsymbol{H}^*} + \epsilon. \tag{30}$$

We refer to Equation (30) as Model 2 (M_2). We compared the effect estimates before and after applying our bias-correction methods on M_2. In this section, we consider including up to 5 covariates in the analysis. The results when all 20 metabolomic PCs were included are given in later sections.

Table AA presents the point estimates of $\boldsymbol{b}$ along with the corresponding standard errors (SE). Notably, different MVMR methods yielded inconsistent outcomes, primarily attributed to the presence of weak instrument bias,which will bias causal estimates

towards or away from 0 [10]. The weak instrument bias arised due to the removal of genetic components, resulting in few or no SNPs demonstrating strong associations with the covariates. This is further substantiated by the fact that most conditional F statistics in Table AH are below 10 [10], indicating a substantial weak instrument bias in the analysis.

Table AA. Point estimates of $\boldsymbol{b}$ when applying different MVMR methods on M_2. Standard errors (SE) are given in parenthesis.

Dimension of $\hat{\boldsymbol{b}}$	1	2		3			4				5				
$\hat{\boldsymbol{b}}$	$\hat{b}_1$	$\hat{b}_1$	$\hat{b}_2$	$\hat{b}_1$	$\hat{b}_2$	$\hat{b}_3$	$\hat{b}_1$	$\hat{b}_2$	$\hat{b}_3$	$\hat{b}_4$	$\hat{b}_1$	$\hat{b}_2$	$\hat{b}_3$	$\hat{b}_4$	$\hat{b}_5$
MVMR-cML	0.22	0.15	0.62	1.23	−1.08	0.68	1.59	−1.99	0.65	−0.99	1.75	−1.86	0.58	−1.02	−0.19
(SE)	(0.05)	(0.05)	(0.18)	(0.29)	(0.41)	(0.14)	(0.50)	(0.81)	(0.16)	(0.38)	(0.63)	(0.90)	(0.24)	(0.45)	(0.38)
MVMR-Egger	0.83	−0.1	0.35	0.12	0.21	0.15	0.14	0.13	0.16	0.03	0.14	0.13	0.12	0.02	−0.04
(SE)	(1.21)	(0.23)	(0.13)	(0.09)	(0.07)	(0.03)	(0.09)	(0.08)	(0.03)	(0.05)	(0.09)	(0.09)	(0.04)	(0.04)	(0.07)
MVMR-IVW	0.20	0.14	0.38	0.07	0.21	0.16	0.1	0.14	0.17	0.03	0.1	0.14	0.12	0.02	−0.04
(SE)	(0.08)	(0.05)	(0.13)	(0.06)	(0.07)	(0.03)	(0.06)	(0.07)	(0.02)	(0.04)	(0.06)	(0.09)	(0.04)	(0.04)	(0.07)
MVMR-Lasso	0.26	0.14	0.38	0.07	0.21	0.16	0.1	0.14	0.17	0.03	0.1	0.14	0.12	0.02	−0.04
(SE)	(0.08)	(0.05)	(0.13)	(0.06)	(0.07)	(0.03)	(0.06)	(0.07)	(0.02)	(0.04)	(0.06)	(0.09)	(0.04)	(0.04)	(0.07)
MVMR-Median	0.20	0.11	0.4	0.05	0.28	0.15	0.09	0.17	0.16	0.02	0.09	0.17	0.11	0.01	−0.07
(SE)	(0.08)	(0.08)	(0.15)	(0.09)	(0.1)	(0.04)	(0.09)	(0.1)	(0.03)	(0.06)	(0.08)	(0.12)	(0.05)	(0.05)	(0.09)
DHO	−0.68														
(SE)	(20.97)	NA		NA			NA				NA				
SH	0.27														
(SE)	(0.13)														

Table AB presents numbers of significant loci after applying our bias-reduction method to M_2. Overall, there was a reduction in the number of identified loci after correction. In Fig AC, the majority of effect estimates remained consistent after bias correction, indicating that M_2 successfully mitigated collider bias by eliminating the genetic components of metabolomic PCs. Consequently, the decrease in significant loci was primarily attributed to reduced statistical power rather than the rectification of type-I errors induced by collider bias, as our bias-correction approach increased variance. For example, when considering only the first metabolomic PC, M_2 identified 255 significant SNPs, and after MVMR-cML-bias correction, only 182 remained significant. Similarly, with the inclusion of two metabolomic PCs, M_2 identified 269 significant SNPs, among these 269 SNPs, only 89 remained significant after MVMR-cML-bias-correction. As more covariates were incorporated, fewer significant SNPs were identified after bias correction. In comparison to other MVMR methods, MVMR-cML tended to yield fewer significant SNPs after bias correction. This could be attributed to MVMR-cML's tendency to produce a larger standard error for $\hat{\boldsymbol{b}}$, as indicated in Table AA, and hence a larger standard error for the bias-corrected estimator $\hat{\beta}_{G_iY}$. For some MVMR methods, such as MVMR-Egger, when 3 or 4 covariates were used in analysis, the amount of significant SNPs after bias correction were more than that when two covariates are adjusted. There are two reasons. First, when $p_2 = 3$ or 4, the magnitude of $\hat{\boldsymbol{b}}$ and its standard errors were smaller than those when $p_2 = 2$. Second, the inclusion of more covariates in the analysis accounted for more environmental variance in BMI. This resulted in a smaller

residual variance in the summary statistic. According to equation (29), the variance of the bias-corrected estimator $\hat{\beta}_{G_iY}$ might not be severely inflated. On the contrary, DHO produced a large standard error of $\hat{\boldsymbol{b}}$, resulting in a substantial loss of power and no identified significant SNPs after bias correction.

Table AB. Number of significant loci after applying different bias-correction methods on M_2.

# of metabolomic PCs in analysis		No correction	MVMR-cML	MVMR-Egger	MVMR-Lasso	MVMR-Median	MVMR-IVW	DHO	SH
1	# of significant SNPs	225	227	16	221	219	221	0	210
	# of significant loci	43	35	2	35	33	35	0	30
	UKB validation	42	32	2	35	33	35	0	30
	other validation	40	32	2	32	30	32	0	28
2	# of significant SNPs	269	58	102	118	105	118		
	# of significant loci	50	22	11	26	25	26	NA	NA
	UKB validation	44	11	19	24	24	24		
	other validation	46	11	20	26	25	26		
3	# of significant SNPs	272	8	175	189	148	189		
	# of significant loci	47	1	34	38	29	38	NA	NA
	UKB validation	41	1	31	33	26	33		
	other validation	43	1	34	37	29	37		
4	# of significant SNPs	281	0	183	196	176	196		
	# of significant loci	49	0	29	35	30	35	NA	NA
	UKB validation	39	1	0	32	27	32		
	other validation	49	0	28	34	29	34		
5	# of significant SNPs	281	0	177	188	168	188		
	# of significant loci	50	0	30	31	28	31	NA	NA
	UKB validation	40	0	27	28	25	28		
	other validation	43	0	29	30	27	30		

Fig AC represents the SNP effect estimates before (horizontal coordinate) and after (vertical coordinate) applying bias correction on M_2, along with their corresponding standard errors (SEs) shown as horizontal (before correction) and vertical (after correction) bars. We only present the figures of MVMR-cML,and focus on scenarios involving 1, 2, or 5 metabolomic PCs, while the comprehensive results are available in subsequent sections. Overall all MVMR methods produced similar figures. In Fig AC (a), where only the first metabolomic PC was considered, both approaches yielded similar results. The effect estimates aligned closely with the identity line, indicating a strong agreement between the two methods. In this case, both approaches effectively mitigated collider bias, but some SNPs might not be significant after bias correction due to reduced power. Fig AC (c) and Fig AC (e) illustrate cases involving more covariates. While most dots remained close to the identity line, a few SNPs exhibited different effect estimates after bias correction. Even after removing genetic components, collider bias might persist for these SNPs. Since our bias-correction approach pulled their effect estimates away from 0, these SNPs might be genuinely associated with BMI. But they were not deemed significant due to variance inflation, as evidenced by the long vertical bars. Therefore, our approach appeared more effective in reducing this bias compared to M_2, albeit with a trade-off of power. It's crucial to interpret these results with caution, given that $\hat{\boldsymbol{b}}$ was susceptible to weak instrument bias. Furthermore, discrepancies between effect estimates were observed only for a few SNPs. As more covariates were included, an increasing number of SNPs not significant after bias correction aligned with the identity line, indicating SNPs not identified due to reduced power. When $p_2 = 5$, no SNPs remained significant

after MVMR-cML-bias-correction. When $p_2 = 1$, some SNPs not identified by M_2 were significant after bias correction, likely due to random noise rather than collider bias, as their effect estimates remained close to the identity line. For the SNPs identified to be significant both before and after bias correction, the effect estimates exhibited high consistency. The clustered effect estimates in Fig AC near the identity line implied a substantial similarity between the two bias-correction approaches.

The scatter plots in the right column of Fig AC illustrate the SEs of SNP effect estimates before (horizontal coordinate) and after (vertical coordinate) bias correction. As anticipated, when only one covariate was involoved, the SEs of two approaches were similar. However, when more covariates were included in the analysis, our bias-reduction approach led to an inflation of the SEs of $\hat{\beta}_{G_iY}$. This inflation of SEs could potentially reduce the statistical power of detecting direct SNP effects. It highlighted the trade-off between bias reduction and power reduction when adjusting for an increasing number of covariates.

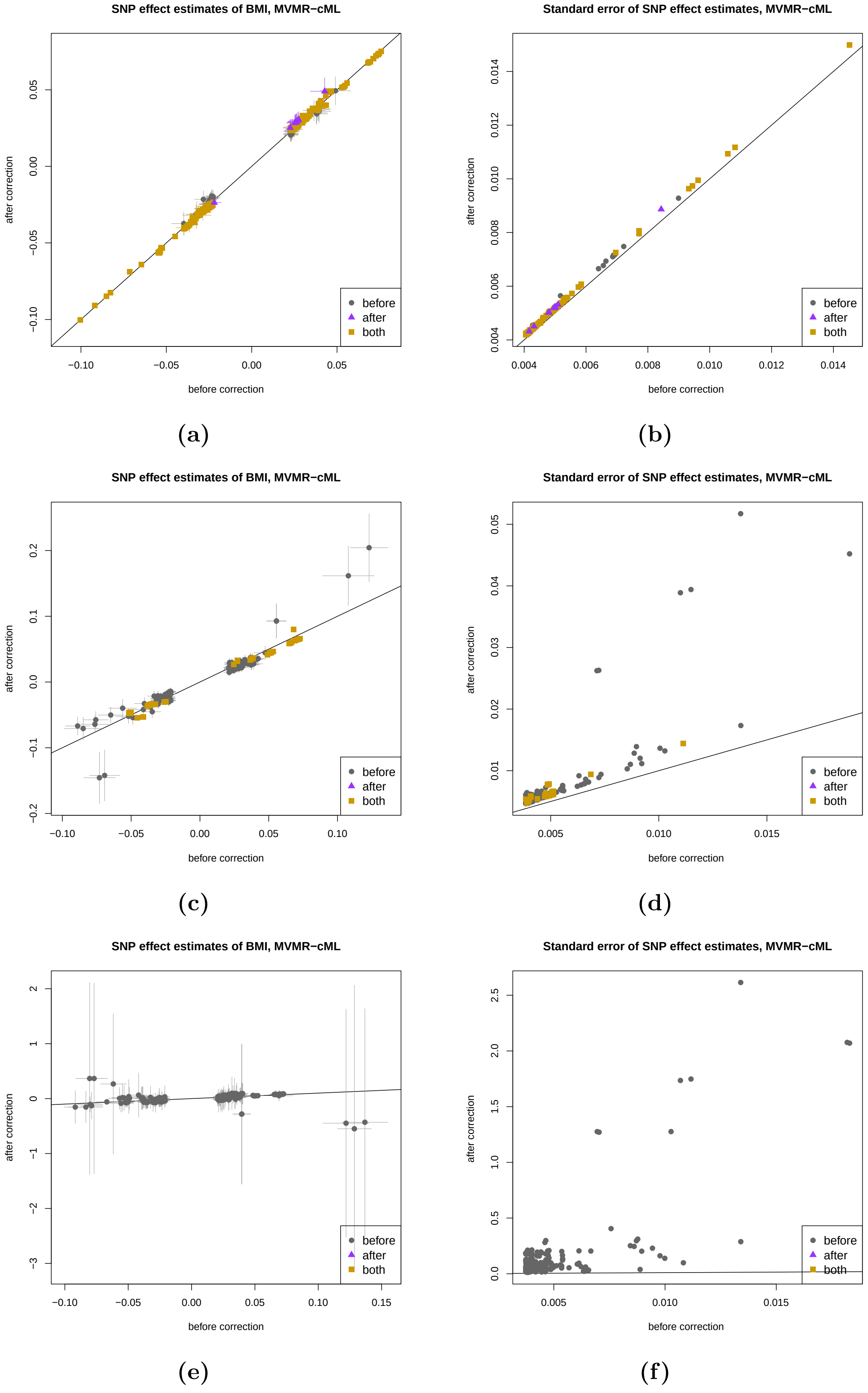

Fig AC. Effect estimates (in M_2) of BMI before and after bias correction. Horizontal and vertical bars represent 1 SE of an estimate before and after correction respectively. SEs are given in the right column. (a)-(b):1 metabolomic PC is used. (c)-(d): 2 metabolomic PCs are used. (e)-(f): 5 metabolomic PCs are used. In the legends, “before” refers to the SNPs that are significant only before applying bias correction, “after” refers to the SNPs that are significant only after bias correction, “both” refers to the SNPs that are significant both before and after bias correction.

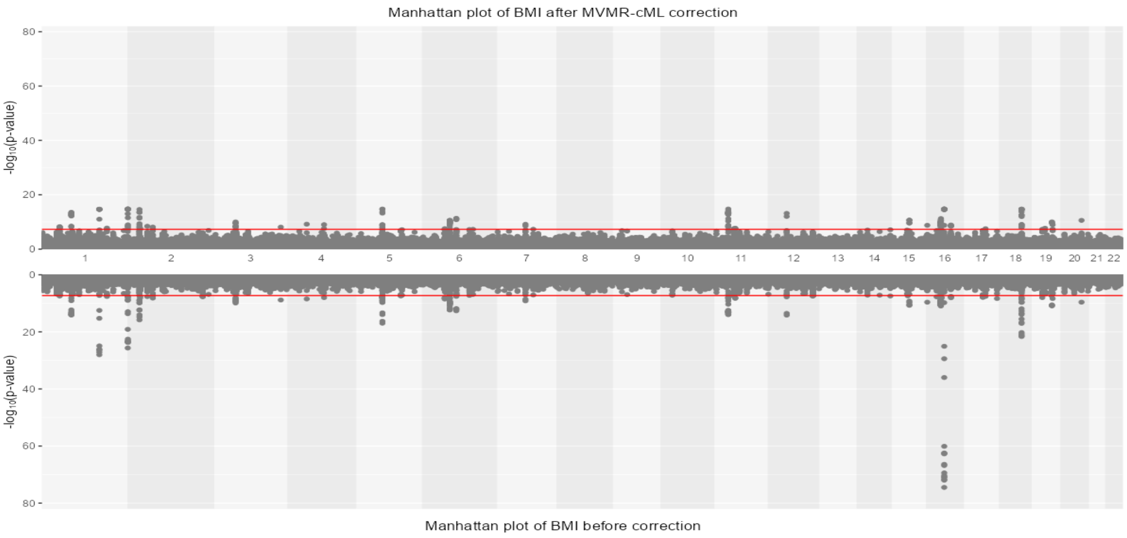

(a)

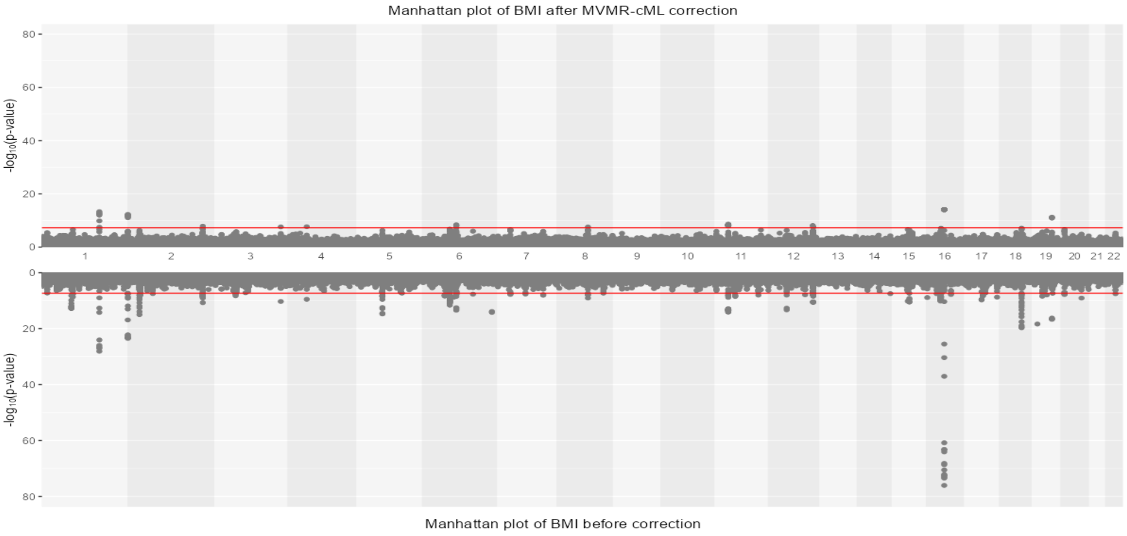

(b)

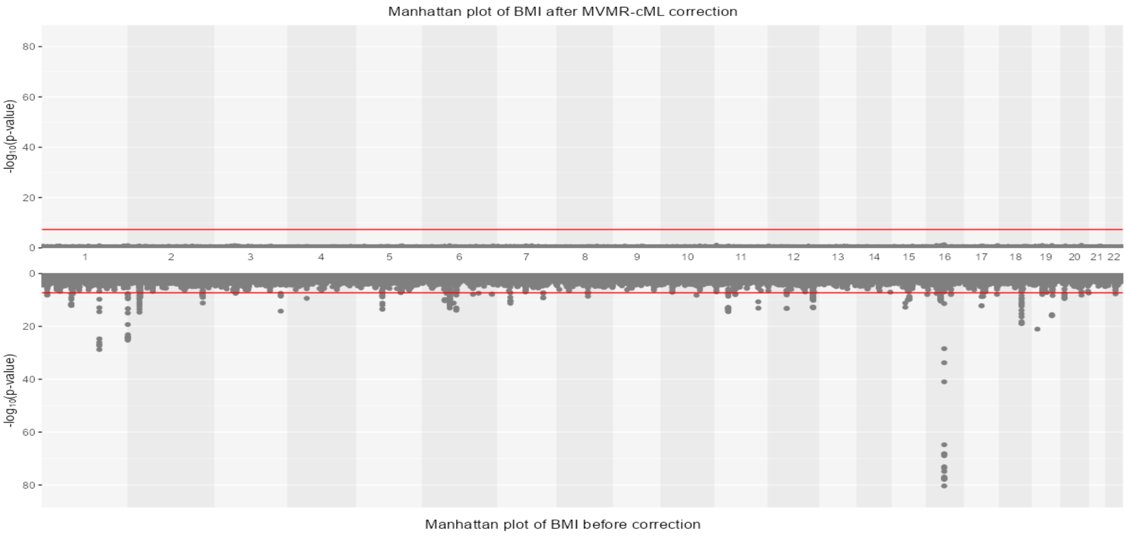

(c)

Fig AD. Manhattan plot of BMI before (upper panel) and after (lower panel) applying MVMR-cML-bias-correction (in M_2). (a): one metabolomic PC is adjusted, (b): two metabolomic PCs are adjusted, (c): five metabolomic PCs are adjusted.

Fig AD presents the Manhattan plots for different dimensions of $\boldsymbol{H}^*$: $p_2 = 1$, 2, or 5. The upper panels display the result after bias correction, while the lower panels show the result of M_2. It could be observed that when $p_2 \leq 2$, the upper and lower Manhattan

plots exhibited a similar profile. However, the p-values in the upper plots were generally larger due to the inflation of SEs. As more covariates were included, our bias-correction method tended to yield fewer significant loci, primarily due to a reduction in statistical power.

In conclusion, both M_2 and our proposed method demonstrated the ability to reduce collider bias. When only one covariate was considered, the two approaches produced similar results. However, as more covariates were introduced into the analysis, the SEs produced by our method became significantly inflated, leading to a reduction in statistical power.

G.2.2 Tables of significant loci

Table AC. Number of significant loci after applying different bias-correction methods on M_1. More than 5 metabolomics are used.

# of metabolomic PCs corrected		No correction	MVMR-cML	MVMR-Egger	MVMR-Lasso	MVMR-Median	MVMR-IVW
6	# of significant SNPs	554	76	148	149	126	149
	# of significant loci	61	11	21	21	18	21
	UKB validation	43	11	21	21	18	21
	other validation	45	11	20	20	18	20
7	# of significant SNPs	521	82	161	162	147	162
	# of significant loci	59	13	23	24	24	24
	UKB validation	43	13	23	24	24	24
	other validation	45	13	22	23	23	23
8	# of significant SNPs	519	38	111	118	104	118
	# of significant loci	59	6	18	19	16	19
	UKB validation	43	6	18	19	16	19
	other validation	45	6	17	18	15	18
9	# of significant SNPs	476	40	86	96	89	96
	# of significant loci	61	6	14	14	13	14
	UKB validation	43	6	14	14	13	14
	other validation	48	6	14	14	13	14
10	# of significant SNPs	473	0	69	80	65	80
	# of significant loci	61	0	11	13	10	13
	UKB validation	43	0	11	13	10	13
	other validation	48	0	11	13	10	13

Table AD. Point estimates of $\boldsymbol{b}$ when applying different MVMR methods on M_1. Standard errors (SE) are given in parenthesis. All 20 metabolomic PCs are used in the GWAS of BMI.

Dimension of $\boldsymbol{b}$	1	2		3			4				5				
$\hat{\boldsymbol{b}}$	$\hat{b}_1$	$\hat{b}_1$	$\hat{b}_2$	$\hat{b}_1$	$\hat{b}_2$	$\hat{b}_3$	$\hat{b}_1$	$\hat{b}_2$	$\hat{b}_3$	$\hat{b}_4$	$\hat{b}_1$	$\hat{b}_2$	$\hat{b}_3$	$\hat{b}_4$	$\hat{b}_5$
MVMR-cML	0.15	0.15	0.00	0.10	−0.31	0.25	0.24	0.04	0.03	0.30	0.19	−0.17	0.16	0.28	0.18
(SE)	(0.03)	(0.03)	(0.05)	(0.03)	(0.05)	(0.02)	(0.03)	(0.07)	(0.04)	(0.04)	(0.03)	(0.05)	(0.03)	(0.04)	(0.04)
MVMR-Egger	−0.03	0.00	0.10	0.12	−0.24	0.23	0.23	−0.27	0.15	0.16	0.2	−0.16	0.15	0.26	0.15
(SE)	(0.09)	(0.07)	(0.07)	(0.06)	(0.05)	(0.03)	(0.07)	(0.06)	(0.03)	(0.03)	(0.07)	(0.06)	(0.03)	(0.04)	(0.04)
MVMR-IVW	0.13	0.1	0.07	0.11	−0.24	0.23	0.23	−0.26	0.15	0.17	0.17	−0.16	0.15	0.26	0.15
(SE)	0.04	(0.03)	(0.06)	(0.03)	(0.05)	(0.03)	(0.04)	(0.06)	(0.03)	(0.03)	(0.03)	(0.05)	(0.03)	(0.04)	(0.04)
MVMR-Lasso	0.13	0.1	0.07	0.11	−0.24	0.23	0.23	−0.26	0.15	0.17	0.17	−0.16	0.15	0.26	0.15
(SE)	(0.04)	(0.03)	(0.06)	(0.03)	(0.05)	(0.03)	(0.04)	(0.06)	(0.03)	(0.03)	(0.03)	(0.05)	(0.03)	(0.04)	(0.04)
MVMR-Median	0.13	0.09	0.07	0.09	−0.25	0.23	0.23	−0.29	0.15	0.17	0.18	−0.14	0.14	0.25	0.14
(SE)	(0.04)	(0.05)	(0.08)	(0.04)	(0.09)	(0.04)	(0.05)	(0.11)	(0.05)	(0.07)	(0.05)	(0.1)	(0.04)	(0.06)	(0.05)
DHO	0.07														
(SE)	(0.11)	NA		NA			NA				NA				
SH	−0.05														
(SE)	(0.09)														

Table AE. Number of significant loci after applying different bias-correction methods on M_1. All 20 metabolomic PCs are used in the GWAS of BMI.

# of metabolomic PCs corrected		No correction	MVMR-cML	MVMR-Egger	MVMR-Lasso	MVMR-Median	MVMR-IVW	DHO	SH
1	# of significant SNPs	463	336	356	343	369	343	352	286
	# of significant loci	68	56	56	56	61	56	56	47
	UKB validation	49	43	42	43	47	43	42	38
	other validation	53	44	45	45	49	45	45	42
2	# of significant SNPs	463	301	320	335	293	335		
	# of significant loci	68	52	51	56	50	56	NA	NA
	UKB validation	49	41	37	44	40	44		
	other validation	53	43	40	46	42	46		
3	# of significant SNPs	463	234	220	237	206	237		
	# of significant loci	68	38	38	38	35	38	NA	NA
	UKB validation	49	34	34	34	32	34		
	other validation	53	33	33	33	32	33		
4	# of significant SNPs	463	125	147	157	126	157		
	# of significant loci	68	24	28	26	19	26	NA	NA
	UKB validation	49	22	24	25	19	25		
	other validation	53	23	27	26	19	26		
5	# of significant SNPs	463	154	151	162	132	162		
	# of significant loci	68	28	26	30	19	30	NA	NA
	UKB validation	49	27	26	22	19	29		
	other validation	53	27	26	23	19	29		

Table AF. Point estimates of $\boldsymbol{b}$ when applying different MVMR methods on M_2. Standard errors (SE) are given in parenthesis. All 20 metabolomic PCs are used in the GWAS of BMI.

Dimension of $\boldsymbol{b}$	1	2		3			4				5				
$\hat{\boldsymbol{b}}$	$\hat{b}_1$	$\hat{b}_1$	$\hat{b}_2$	$\hat{b}_1$	$\hat{b}_2$	$\hat{b}_3$	$\hat{b}_1$	$\hat{b}_2$	$\hat{b}_3$	$\hat{b}_4$	$\hat{b}_1$	$\hat{b}_2$	$\hat{b}_3$	$\hat{b}_4$	$\hat{b}_5$
MVMR-cML	−0.17	−0.18	0.21	1.10	−0.89	0.82	3.70	−4.81	1.51	−2.44	0.46	0.41	0.19	−0.03	−0.71
(SE)	(0.04)	(0.05)	(0.19)	(0.21)	(0.30)	(0.10)	(3.62)	(5.53)	(1.12)	(2.69)	(0.24)	(0.36)	(0.11)	(0.17)	(0.18)
MVMR-Egger	0.12	−0.35	0.09	0.21	0.15	0.35	0.13	0.2	0.35	0.05	0.15	0.24	0.29	0.04	−0.16
(SE)	(0.76)	(0.26)	(0.12)	(0.1)	(0.07)	(0.04)	(0.09)	(0.08)	(0.03)	(0.04)	(0.09)	(0.08)	(0.04)	(0.05)	(0.06)
MVMR-IVW	−0.12	−0.15	0.09	0.13	0.16	0.37	0.08	0.21	0.36	0.05	0.1	0.24	0.3	0.04	−0.17
(SE)	(0.07)	(0.04)	(0.11)	(0.07)	(0.07)	(0.03)	(0.06)	(0.08)	(0.02)	(0.04)	(0.06)	(0.08)	(0.04)	(0.05)	(0.06)
MVMR-Lasso	−0.17	−0.15	0.09	0.13	0.16	0.37	0.08	0.21	0.36	0.05	0.1	0.24	0.3	0.04	−0.17
(SE)	(0.07)	(0.04)	(0.11)	(0.07)	(0.07)	(0.03)	(0.06)	(0.08)	(0.02)	(0.04)	(0.06)	(0.08)	(0.04)	(0.05)	(0.06)
MVMR-Median	−0.12	−0.14	0.06	0.13	0.21	0.38	0.06	0.28	0.36	0.07	0.1	0.3	0.28	0.04	−0.19
(SE)	(0.07)	(0.07)	(0.17)	(0.09)	(0.1)	(0.04)	(0.09)	(0.11)	(0.04)	(0.06)	(0.09)	(0.11)	(0.05)	(0.07)	(0.1)
DHO	−3.23														
(SE)	(46.39)	NA		NA			NA				NA				
SH	−0.19														
(SE)	(0.17)														

Table AG. Number of significant loci after applying different bias-correction methods on M_2. All 20 metabolomic PCs are used in the GWAS of BMI.

# of metabolomic PCs corrected		No correction	MVMR-cML	MVMR-Egger	MVMR-Lasso	MVMR-Median	MVMR-IVW	DHO	SH
1	# of significant SNPs	960	419	28	469	445	496	0	496
	# of significant loci	64	60	6	61	59	61	0	66
	UKB validation	50	46	6	47	45	47	0	48
	other validation	49	44	6	46	44	46	0	48
2	# of significant SNPs	960	105	125	412	287	412	NA	NA
	# of significant loci	64	20	21	54	41	54		
	UKB validation	50	19	20	41	34	41		
	other validation	49	19	21	40	35	40		
3	# of significant SNPs	940	14	155	182	150	182	NA	NA
	# of significant loci	64	1	29	35	29	35		
	UKB validation	50	1	28	33	27	33		
	other validation	49	1	28	33	27	33		
4	# of significant SNPs	940	0	149	170	125	170	NA	NA
	# of significant loci	64	0	28	35	25	35		
	UKB validation	50	0	27	31	23	31		
	other validation	49	0	27	33	23	33		
5	# of significant SNPs	940	0	130	147	120	147	NA	NA
	# of significant loci	64	0	24	28	25	28		
	UKB validation	50	0	22	23	22	23		
	other validation	49	0	23	25	20	25		

Table AH. Conditional F statistic for metabolomic PCs.

GWAS model	# of PCs in analysis	PC1	PC2	PC3	PC4	PC5
M_1	2	74.26	26.17			
	3	56.35	24.01	70.72		
	4	43.08	14.78	49.58	22.81	
	5	36.82	12.83	45.57	14.83	20.09
M_2	2	15.01	3.04			
	3	1.90	1.64	2.14		
	4	1.39	1.09	2.26	1.42	
	5	1.38	1.08	1.59	1.45	1.50

Table AI. Conditional F statistic for metabolomic PCs, all 20 metabolomic PCs are used in the GWAS of BMI.

GWAS model	# of PCs corrected	PC1	PC2	PC3	PC4	PC5
M_1	2	65.89	23.00			
	3	59.71	24.41	77.60		
	4	38.19	9.79	18.69	12.86	
	5	31.15	12.47	43.13	14.43	19.73
M_2	2	16.18	3.17			
	3	1.90	1.65	2.14		
	4	1.41	1.20	2.33	1.55	
	5	1.26	1.25	1.83	1.41	1.35

G.2.3 Comparison of SNP effect estimates before and after apply different bias-correction methods on M_1

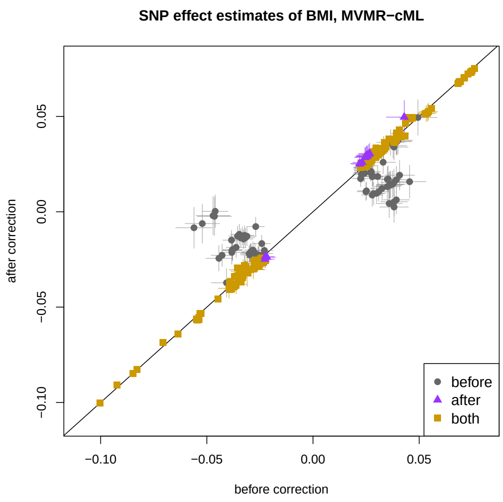

(a)

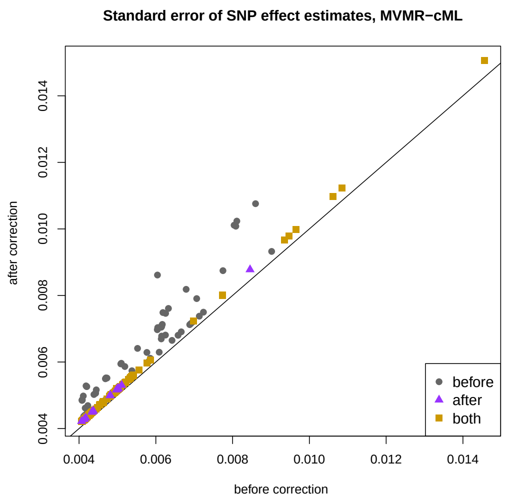

(b)

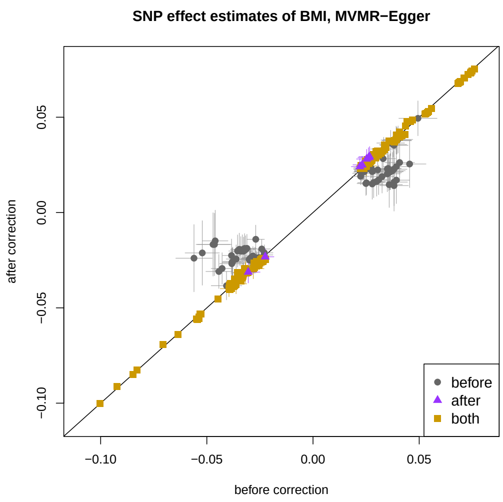

(c)

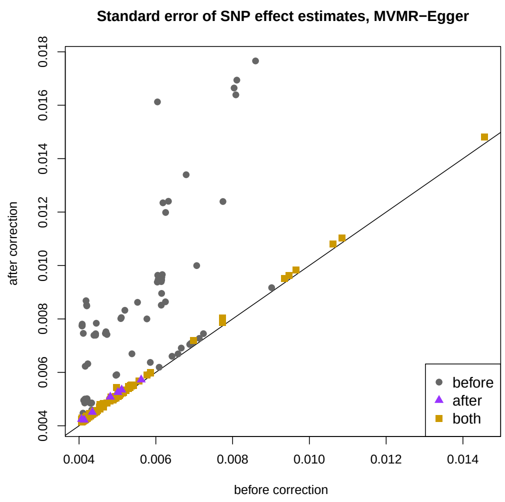

(d)

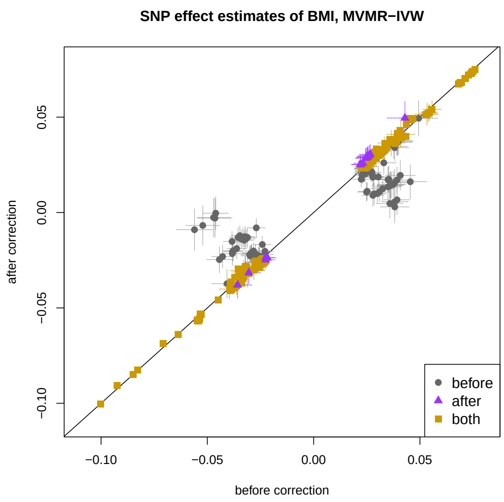

(e)

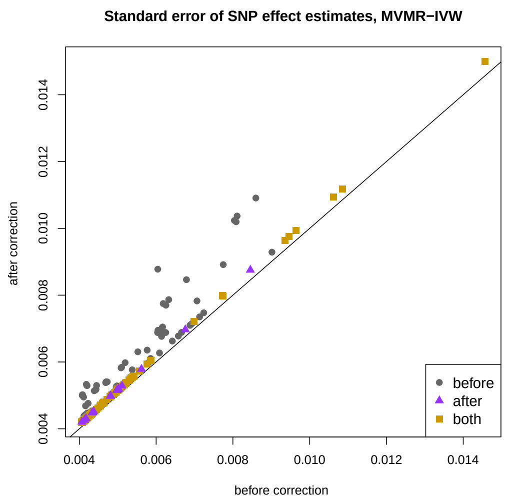

(f)

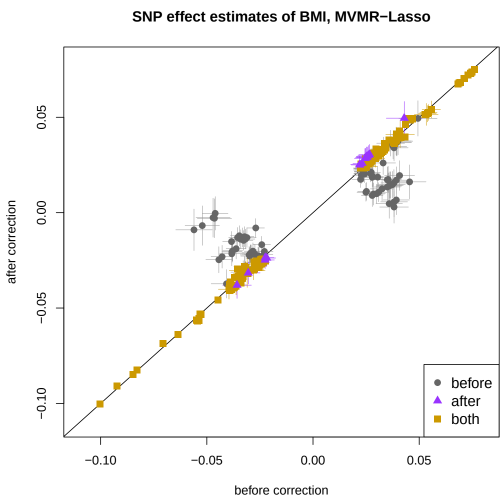
SNP effect estimates of BMI, MVMR−Lasso
after correction
before correction
before
after
both
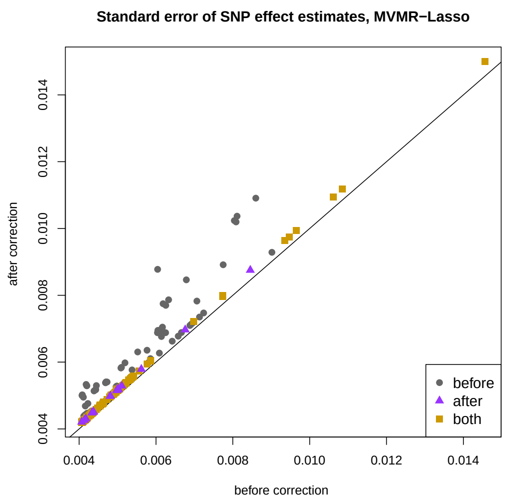
Standard error of SNP effect estimates, MVMR−Lasso
after correction
before correction
before
after
both

(g)

(h)

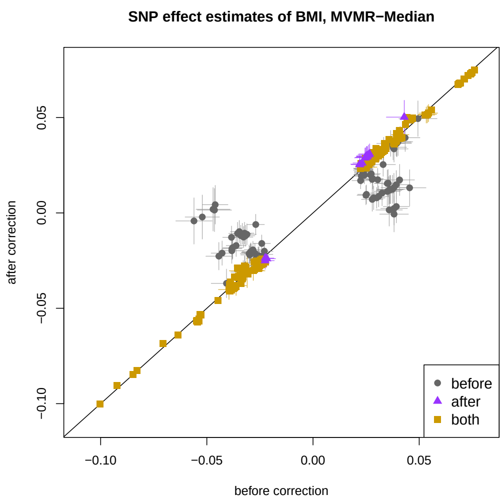
SNP effect estimates of BMI, MVMR−Median
after correction
before correction
before
after
both
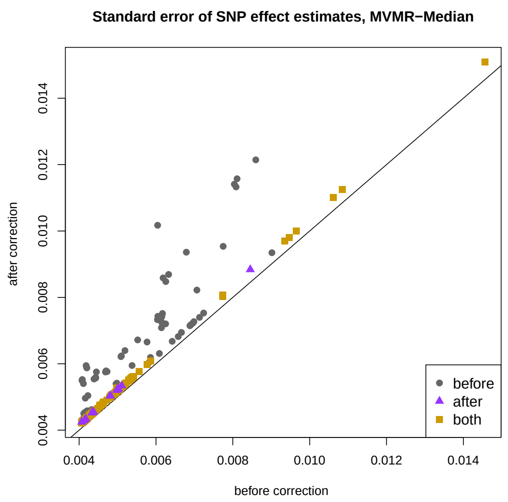
Standard error of SNP effect estimates, MVMR−Median
after correction
before correction
before
after
both

(i)

(j)

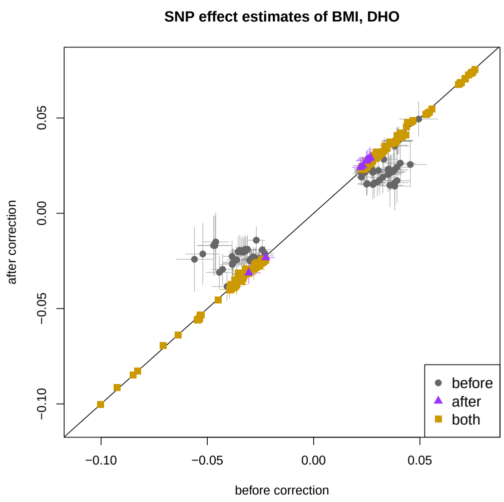
SNP effect estimates of BMI, DHO
after correction
before correction
before
after
both
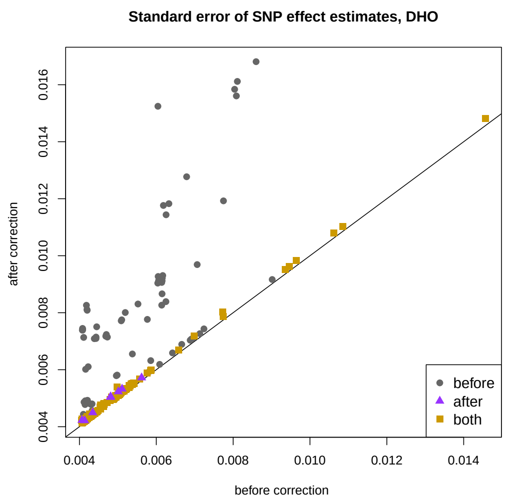
Standard error of SNP effect estimates, DHO
after correction
before correction
before
after
both

(k)

(l)

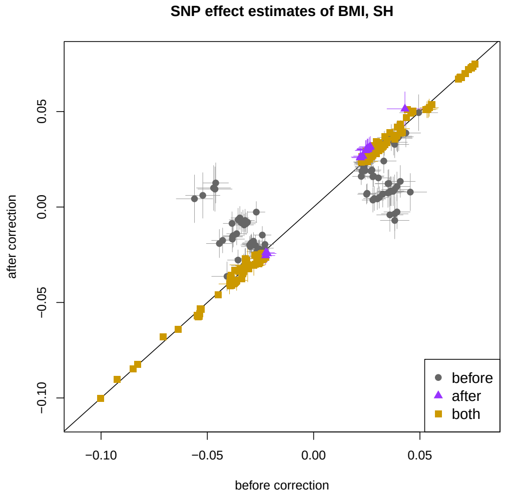

(m)

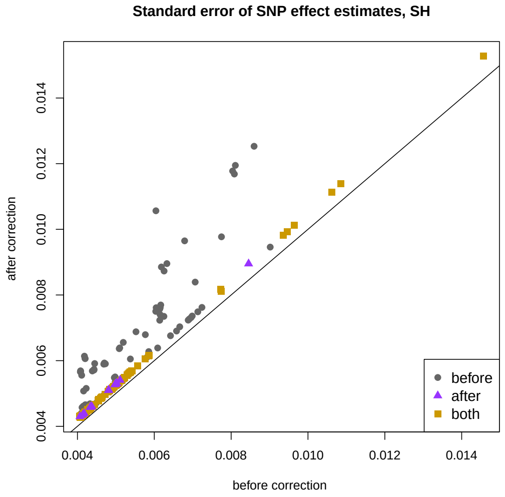

(n)

Fig AE. Effect estimates (in M_1) of BMI before and after bias correction. Horizontal and vertical bars represent 1 SE of an estimate before and after correction respectively. SEs are given in the right column. 1 metabolomic PC is used. In the legends, "before" refers to the SNPs that are significant only before applying bias correction, "after" refers to the SNPs that are significant only after bias correction, "both" refers to the SNPs that are significant both before and after bias correction.

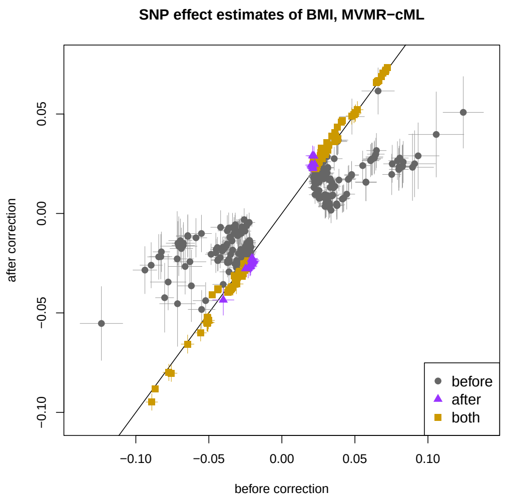
SNP effect estimates of BMI, MVMR−cML
after correction
before correction
−0.10
−0.05
0.00
0.05
0.10
before
after
both

(a)

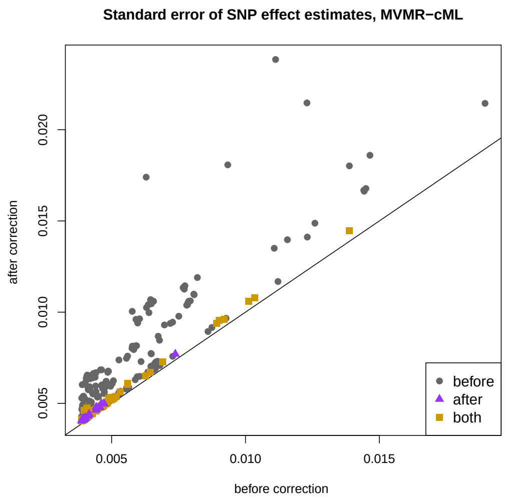
Standard error of SNP effect estimates, MVMR−cML
after correction
before correction
0.005
0.010
0.015
0.020
before
after
both

(b)

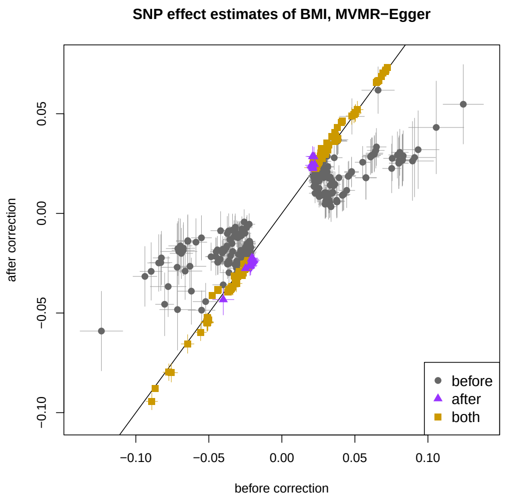
SNP effect estimates of BMI, MVMR−Egger
after correction
before correction
−0.10
−0.05
0.00
0.05
0.10
before
after
both

(c)

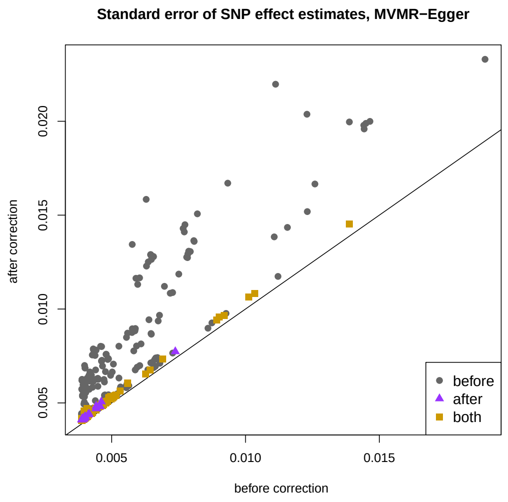
Standard error of SNP effect estimates, MVMR−Egger
after correction
before correction
0.005
0.010
0.015
0.020
before
after
both

(d)

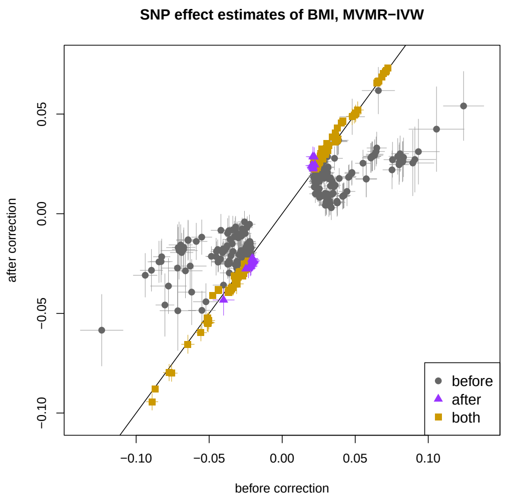
SNP effect estimates of BMI, MVMR−IVW
after correction
before correction
−0.10
−0.05
0.00
0.05
0.10
before
after
both

(e)

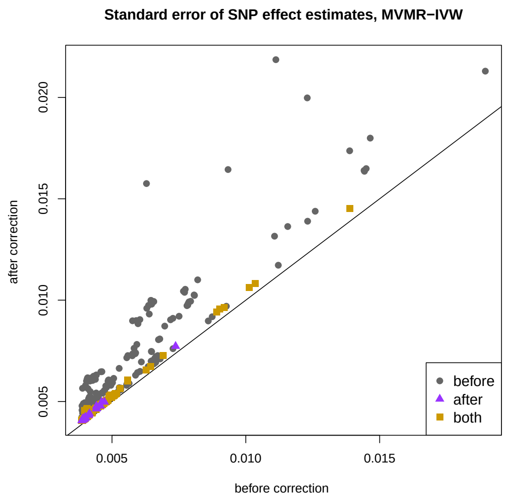
Standard error of SNP effect estimates, MVMR−IVW
after correction
before correction
0.005
0.010
0.015
0.020
before
after
both

(f)

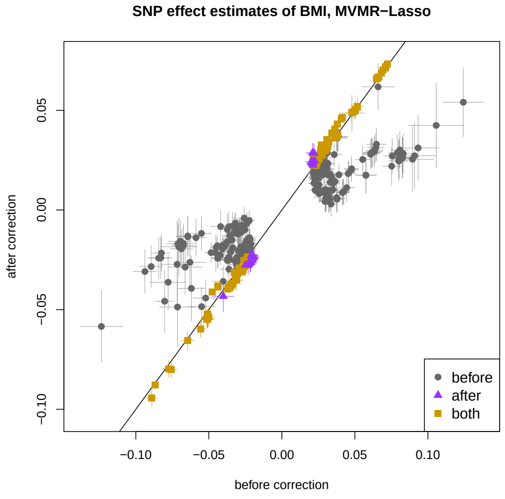

(g)

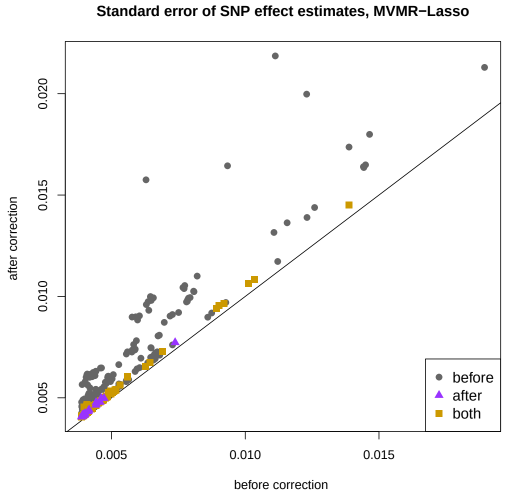

(h)

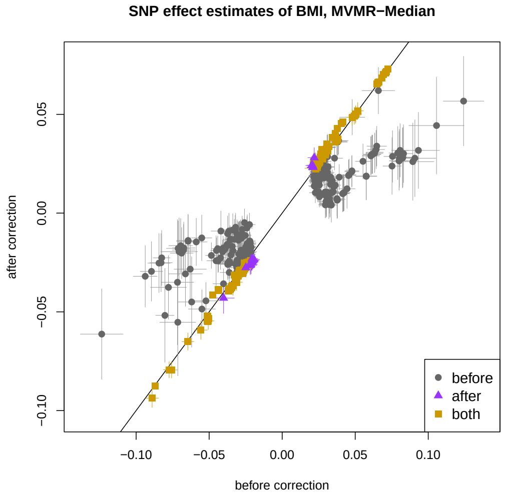

(i)

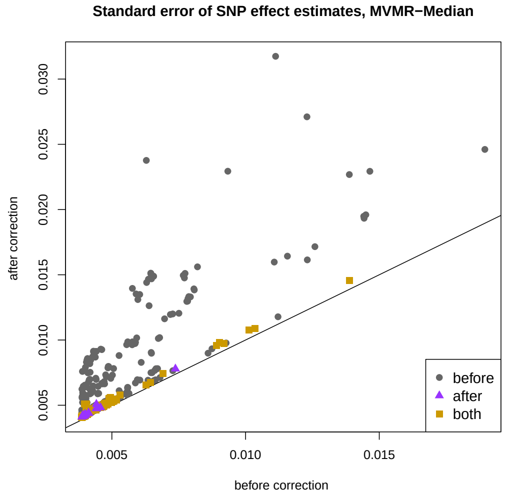

(j)

Fig AF. Effect estimates (in M_1) of BMI before and after bias correction. Horizontal and vertical bars represent 1 SE of an estimate before and after correction respectively. SEs are given in the right column. 2 metabolomic PCs are used. In the legends, "before" refers to the SNPs that are significant only before applying bias correction, "after" refers to the SNPs that are significant only after bias correction, "both" refers to the SNPs that are significant both before and after bias correction.

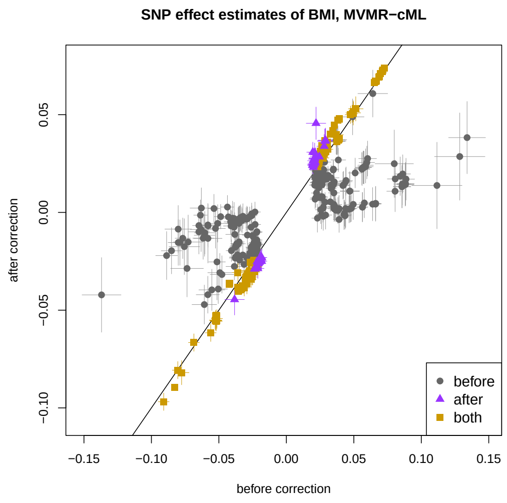
SNP effect estimates of BMI, MVMR−cML
after correction
before correction
before
after
both

(a)

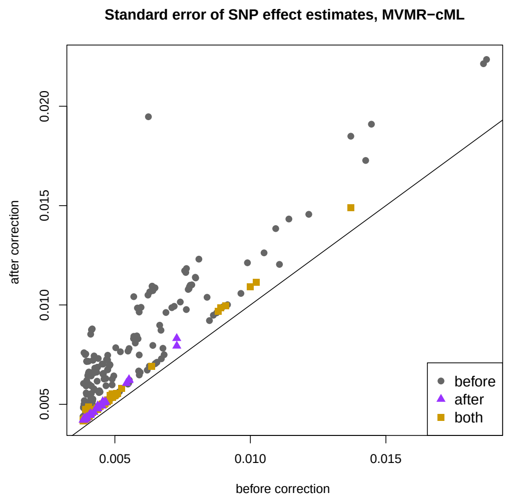
Standard error of SNP effect estimates, MVMR−cML
after correction
before correction
before
after
both

(b)

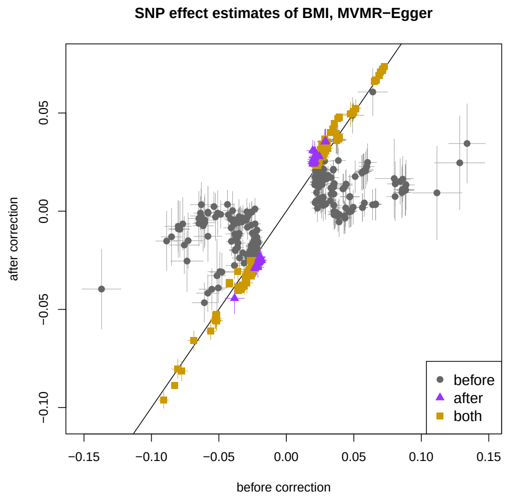
SNP effect estimates of BMI, MVMR−Egger
after correction
before correction
before
after
both

(c)

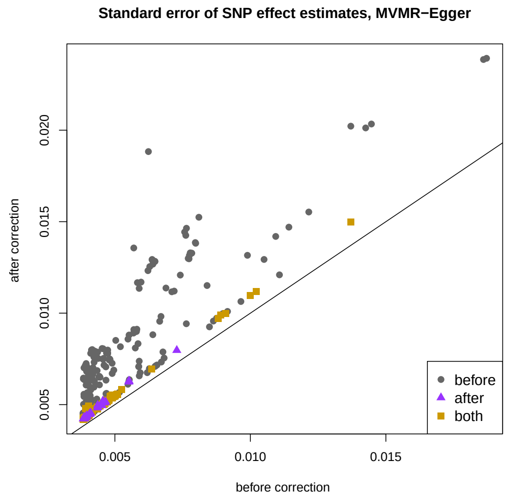
Standard error of SNP effect estimates, MVMR−Egger
after correction
before correction
before
after
both

(d)

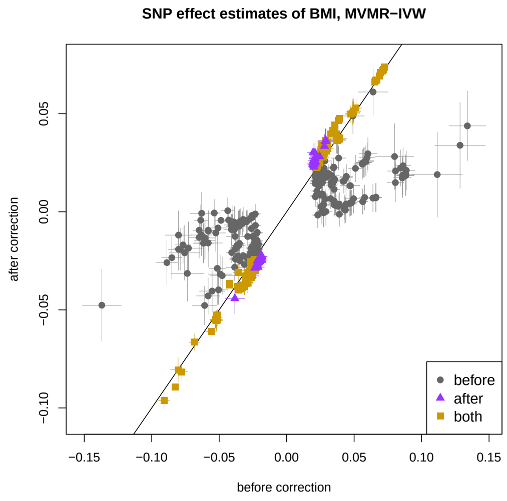
SNP effect estimates of BMI, MVMR−IVW
after correction
before correction
before
after
both

(e)

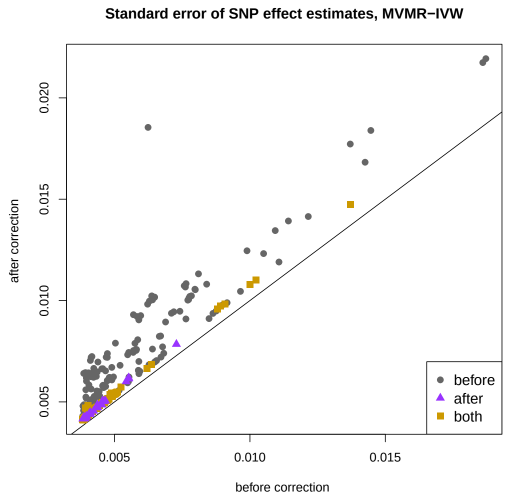
Standard error of SNP effect estimates, MVMR−IVW
after correction
before correction
before
after
both

(f)

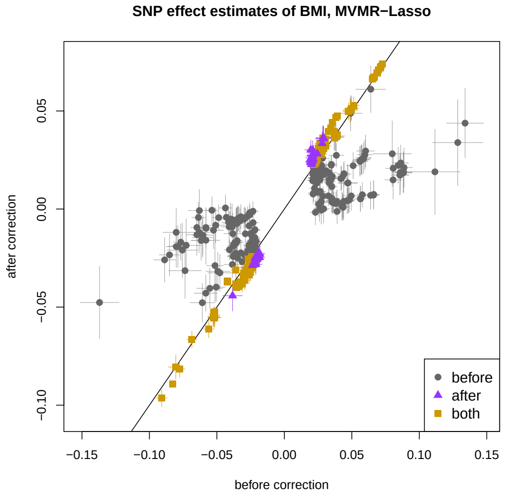

(g)

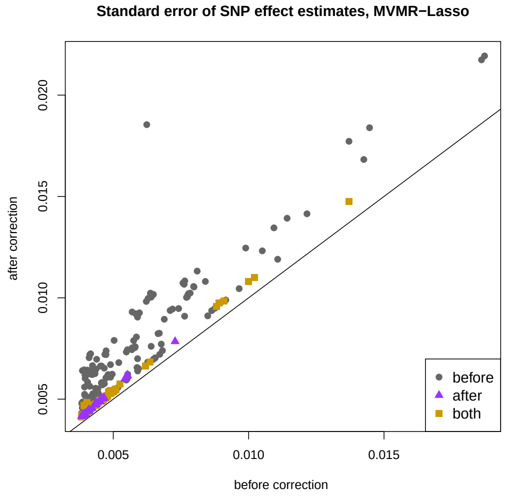

(h)

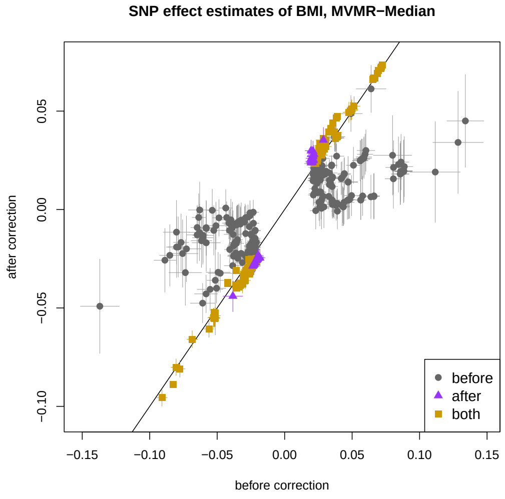

(i)

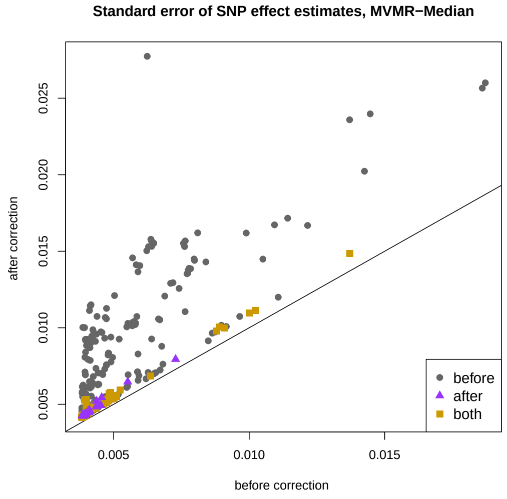

(j)

Fig AG. Effect estimates (in M_1) of BMI before and after bias correction. Horizontal and vertical bars represent 1 SE of an estimate before and after correction respectively. SEs are given in the right column. 3 metabolomic PCs are used. In the legends, "before" refers to the SNPs that are significant only before applying bias correction, "after" refers to the SNPs that are significant only after bias correction, "both" refers to the SNPs that are significant both before and after bias correction.

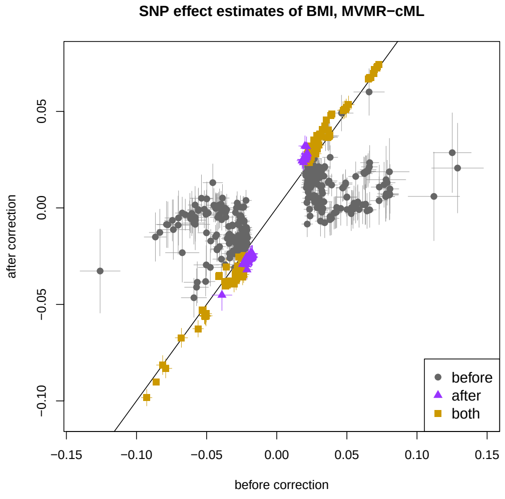
SNP effect estimates of BMI, MVMR−cML
after correction
before correction
−0.10
−0.05
0.00
0.05
−0.15
−0.10
−0.05
0.00
0.05
0.10
0.15
before
after
both

(a)

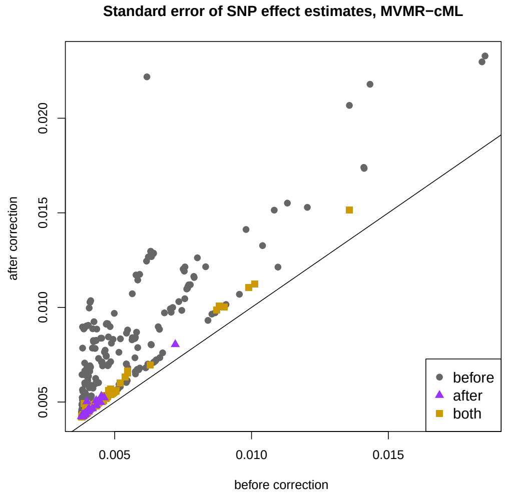
Standard error of SNP effect estimates, MVMR−cML
after correction
before correction
0.005
0.010
0.015
0.020
0.005
0.010
0.015
before
after
both

(b)

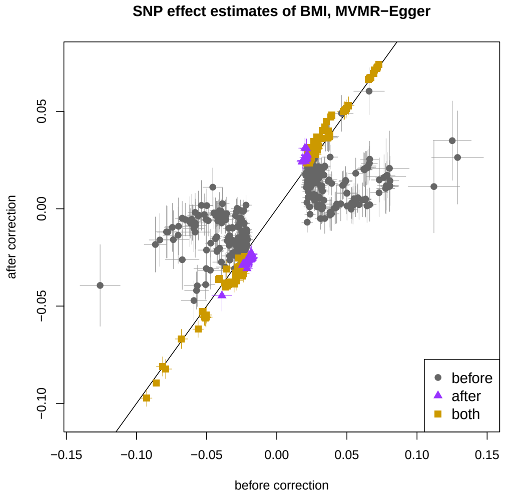
SNP effect estimates of BMI, MVMR−Egger
after correction
before correction
−0.10
−0.05
0.00
0.05
−0.15
−0.10
−0.05
0.00
0.05
0.10
0.15
before
after
both

(c)

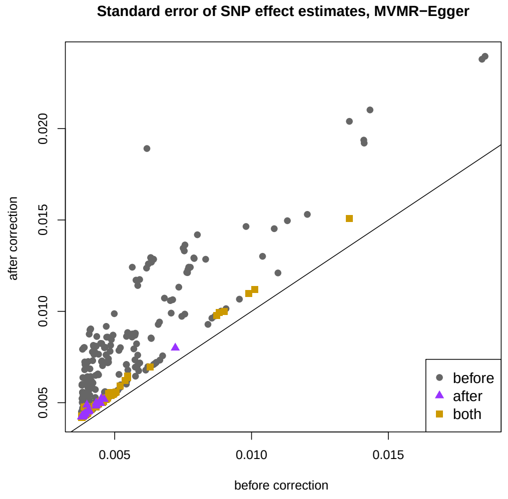
Standard error of SNP effect estimates, MVMR−Egger
after correction
before correction
0.005
0.010
0.015
0.020
0.005
0.010
0.015
before
after
both

(d)

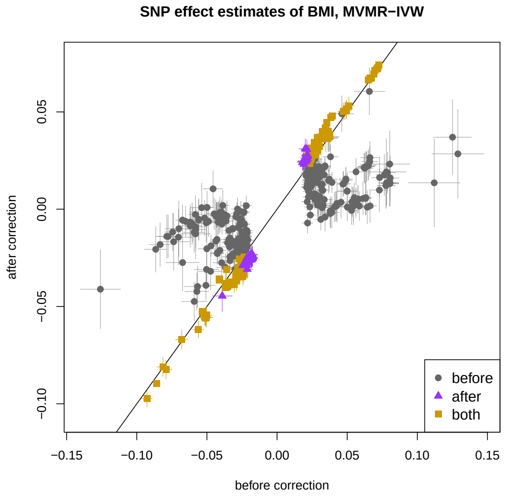
SNP effect estimates of BMI, MVMR−IVW
after correction
before correction
−0.10
−0.05
0.00
0.05
−0.15
−0.10
−0.05
0.00
0.05
0.10
0.15
before
after
both

(e)

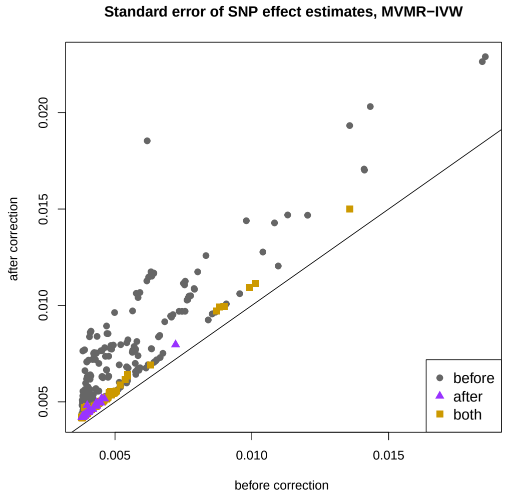
Standard error of SNP effect estimates, MVMR−IVW
after correction
before correction
0.005
0.010
0.015
0.020
0.005
0.010
0.015
before
after
both

(f)

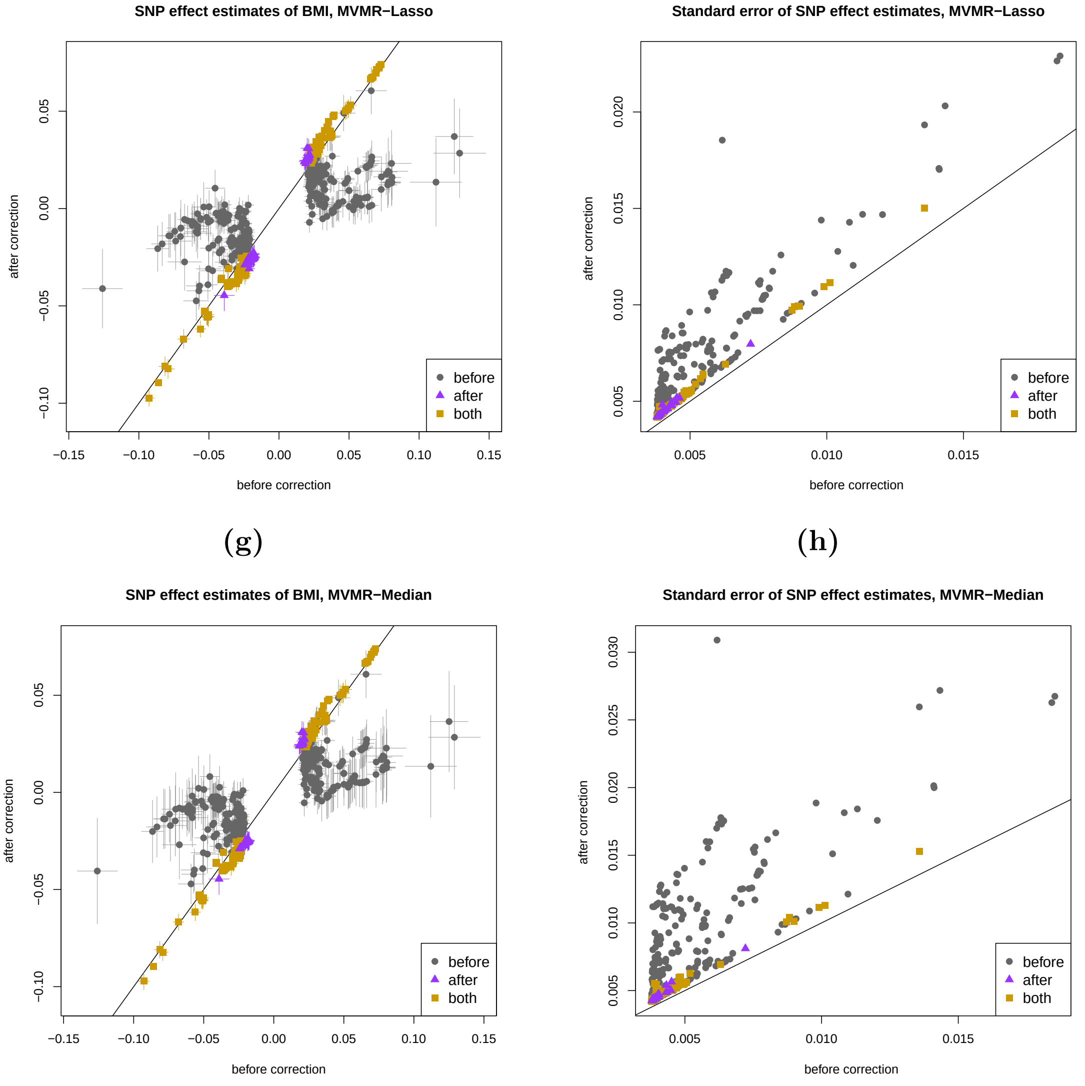

(g) (h)

(i) (j)

Fig AH. Effect estimates (in M_1) of BMI before and after bias correction. Horizontal and vertical bars represent 1 SE of an estimate before and after correction respectively. SEs are given in the right column. 4 metabolomic PCs are used. In the legends, “before” refers to the SNPs that are significant only before applying bias correction, “after” refers to the SNPs that are significant only after bias correction, “both” refers to the SNPs that are significant both before and after bias correction.

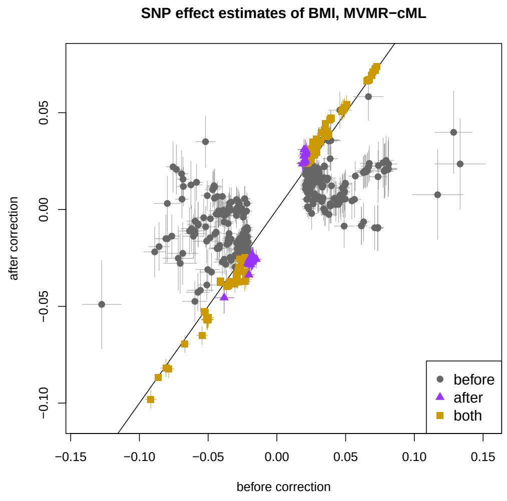
SNP effect estimates of BMI, MVMR−cML
after correction
before correction
before
after
both
−0.15 −0.10 −0.05 0.00 0.05 0.10 0.15
−0.10 −0.05 0.00 0.05

(a)

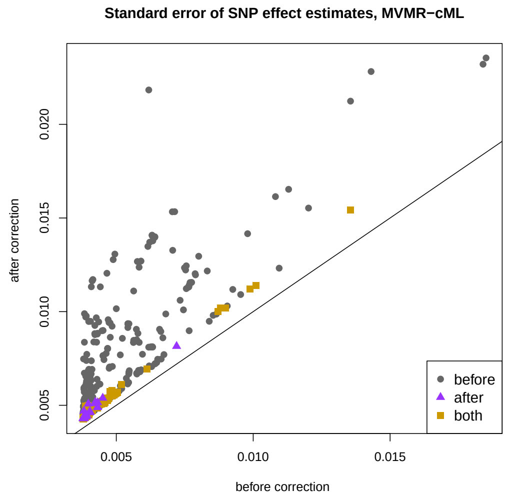
Standard error of SNP effect estimates, MVMR−cML
after correction
before correction
before
after
both
0.005 0.010 0.015
0.005 0.010 0.015 0.020

(b)

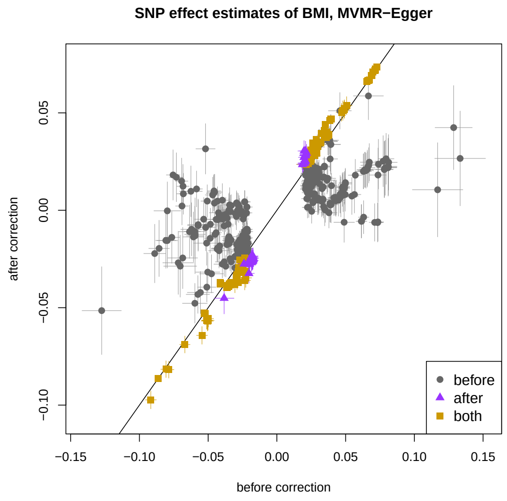
SNP effect estimates of BMI, MVMR−Egger
after correction
before correction
before
after
both
−0.15 −0.10 −0.05 0.00 0.05 0.10 0.15
−0.10 −0.05 0.00 0.05

(c)

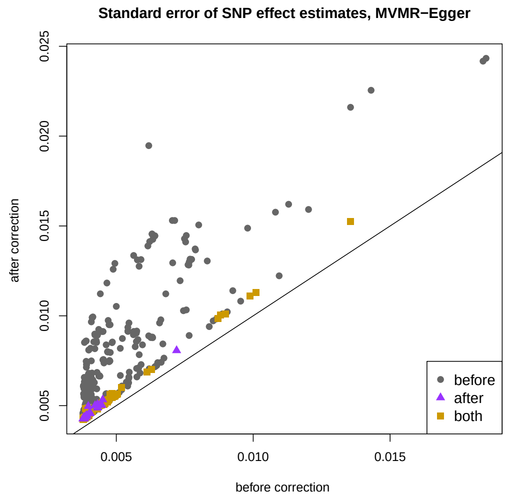
Standard error of SNP effect estimates, MVMR−Egger
after correction
before correction
before
after
both
0.005 0.010 0.015
0.005 0.010 0.015 0.020 0.025

(d)

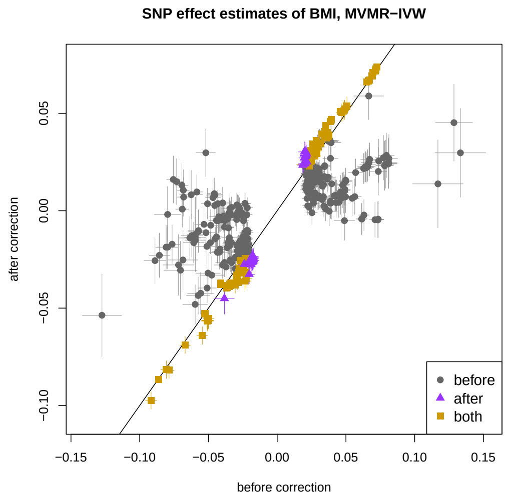
SNP effect estimates of BMI, MVMR−IVW
after correction
before correction
before
after
both
−0.15 −0.10 −0.05 0.00 0.05 0.10 0.15
−0.10 −0.05 0.00 0.05

(e)

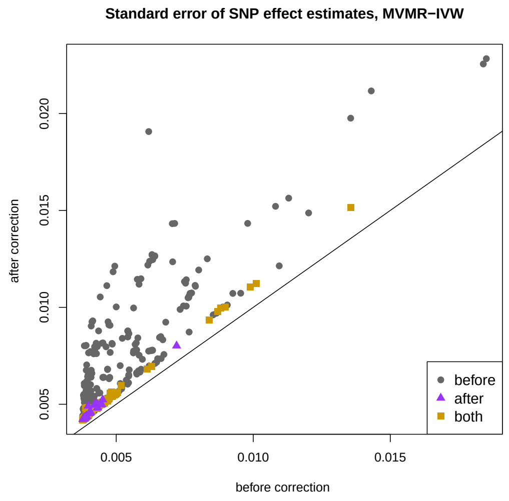
Standard error of SNP effect estimates, MVMR−IVW
after correction
before correction
before
after
both
0.005 0.010 0.015
0.005 0.010 0.015 0.020

(f)

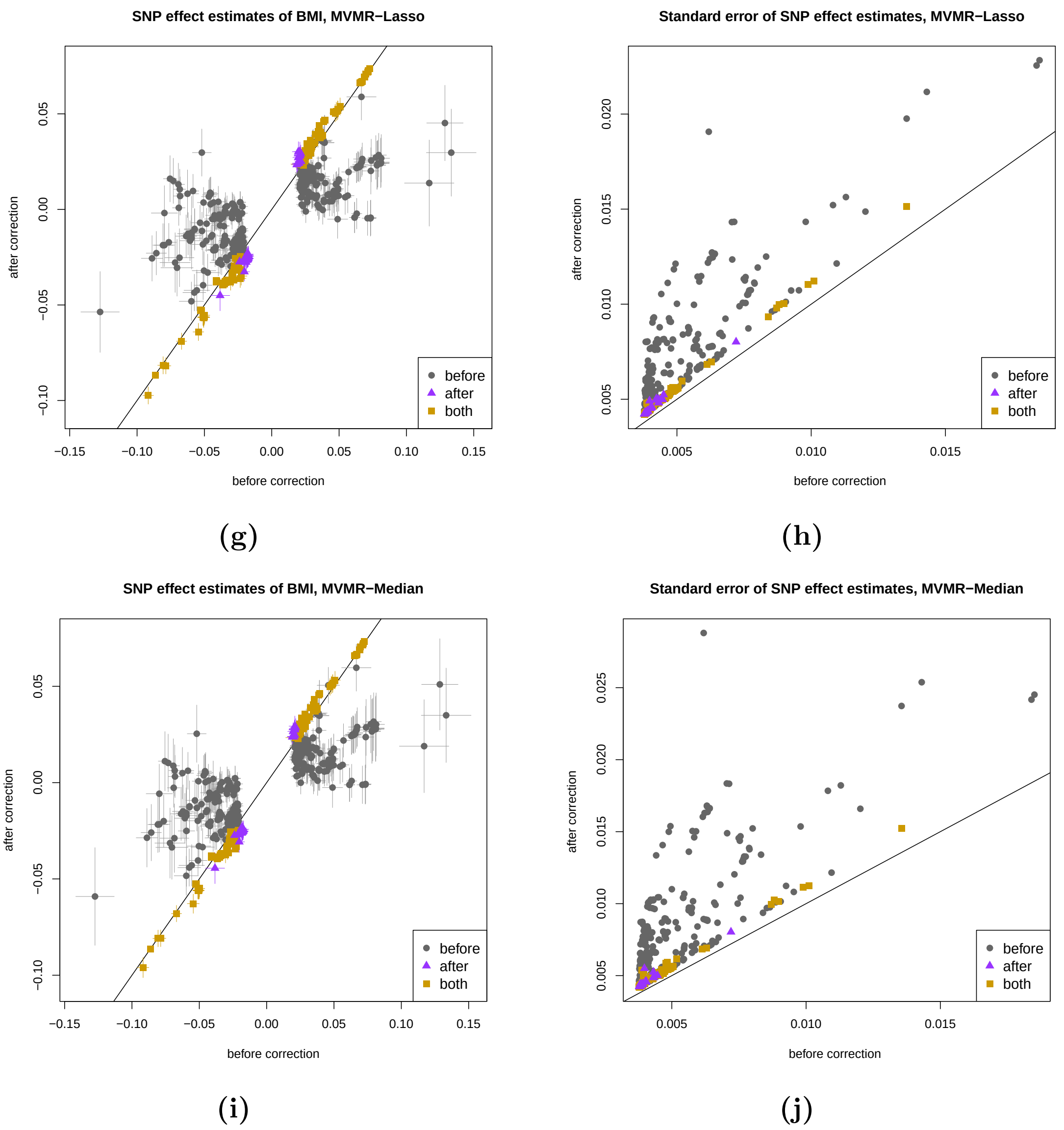

Fig AI. Effect estimates (in M_1) of BMI before and after bias correction. Horizontal and vertical bars represent 1 SE of an estimate before and after correction respectively. SEs are given in the right column. 5 metabolomic PCs are used. In the legends, “before” refers to the SNPs that are significant only before applying bias correction, “after” refers to the SNPs that are significant only after bias correction, “both” refers to the SNPs that are significant both before and after bias correction.

G.2.4 Comparison of SNP effect estimates before and after apply different bias-correction methods on M_1, all 20 metabolomic PCs are used in M_1

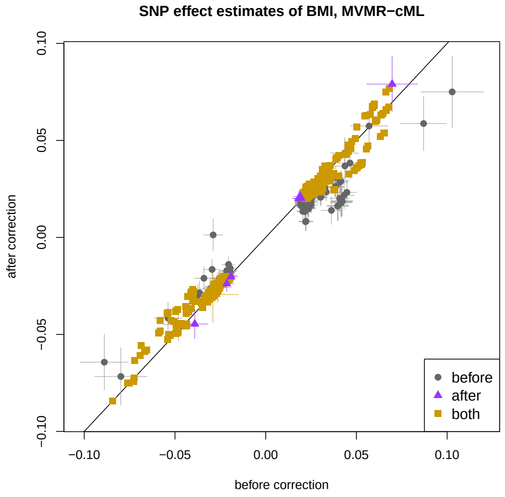

(a)

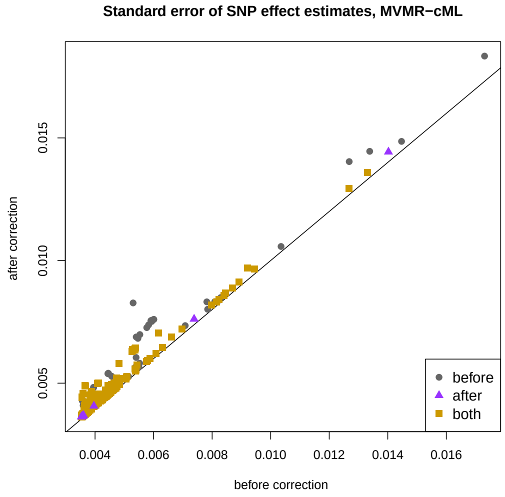

(b)

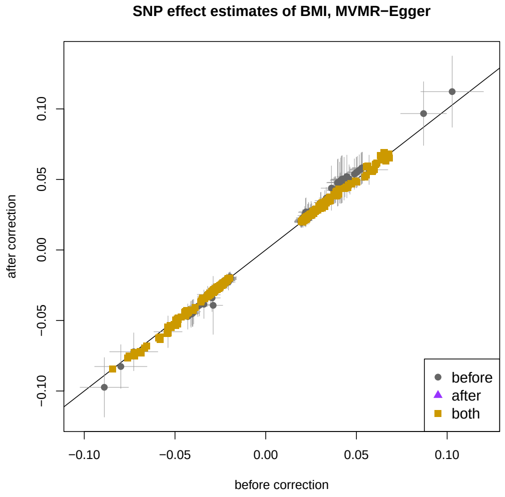

(c)

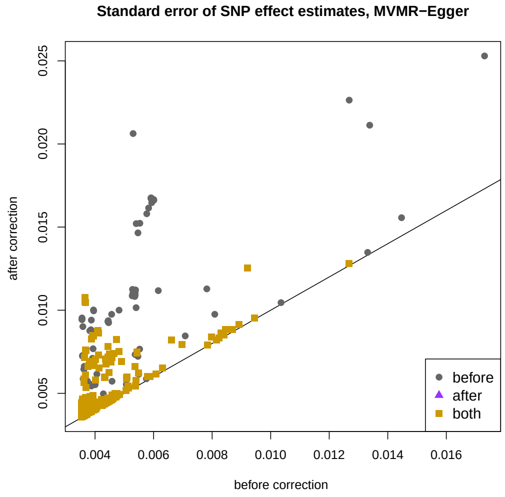

(d)

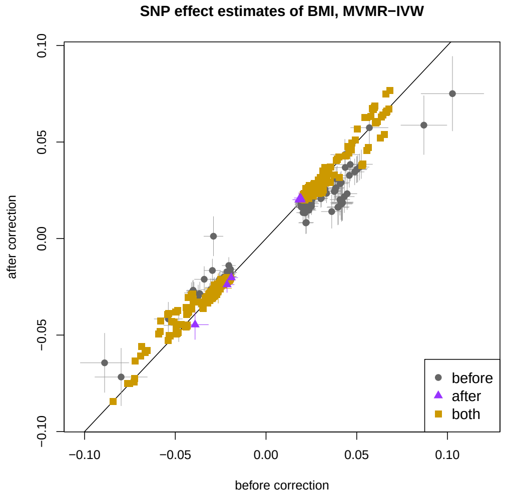

(e)

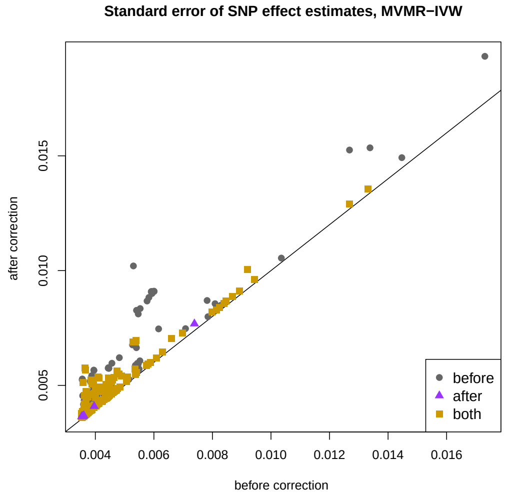

(f)

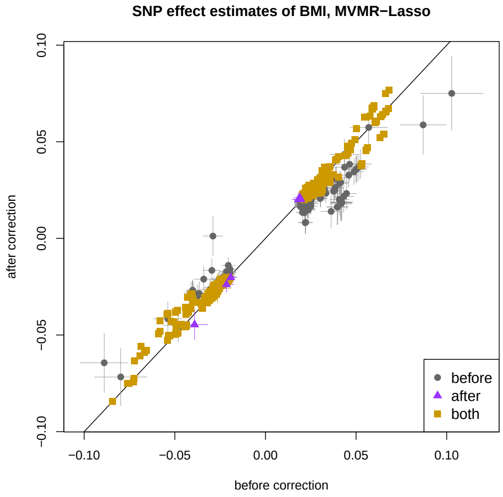
SNP effect estimates of BMI, MVMR−Lasso
after correction
before correction
before
after
both

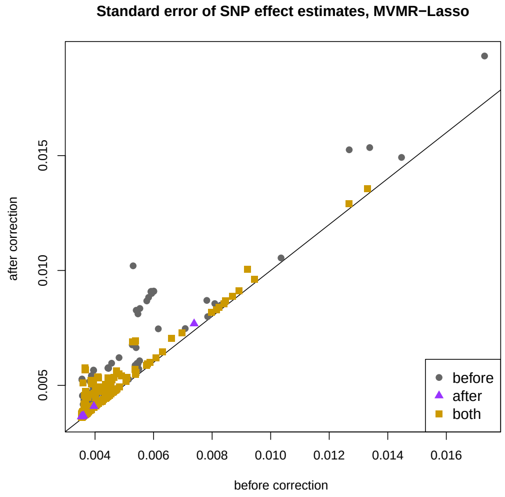
Standard error of SNP effect estimates, MVMR−Lasso
after correction
before correction
before
after
both

(g)

(h)

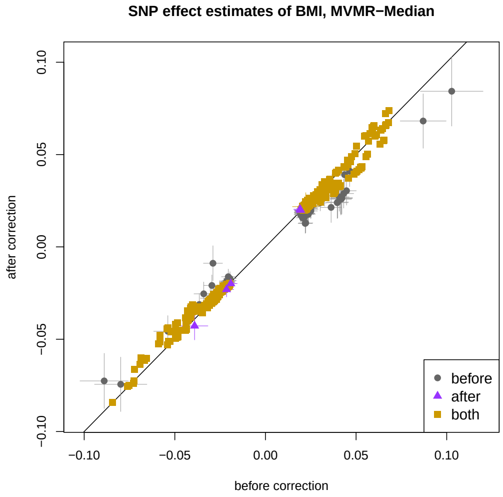
SNP effect estimates of BMI, MVMR−Median
after correction
before correction
before
after
both

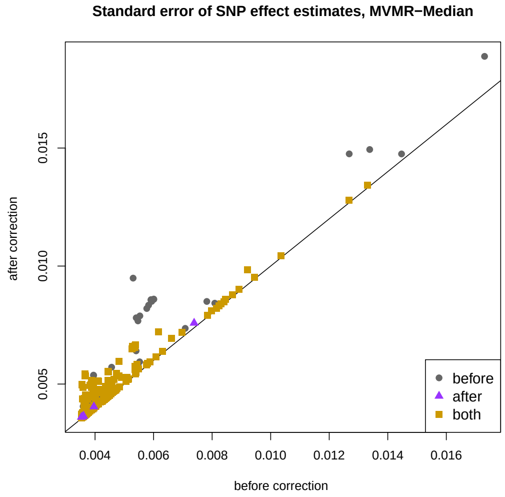
Standard error of SNP effect estimates, MVMR−Median
after correction
before correction
before
after
both

(i)

(j)

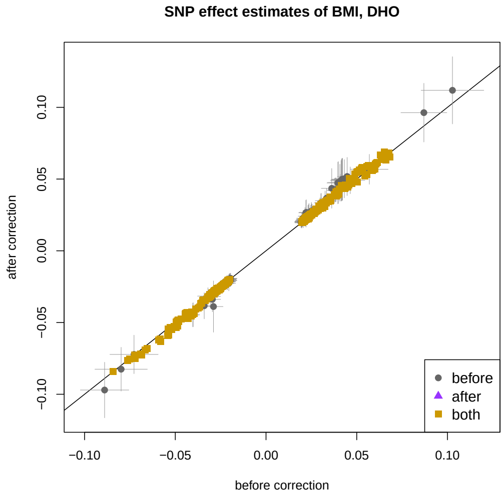
SNP effect estimates of BMI, DHO
after correction
before correction
before
after
both

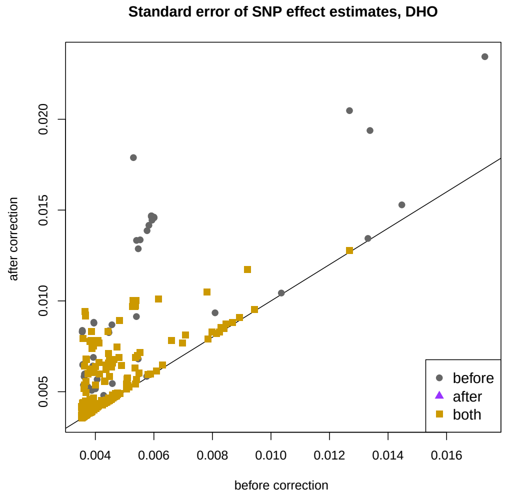
Standard error of SNP effect estimates, DHO
after correction
before correction
before
after
both

(k)

(l)

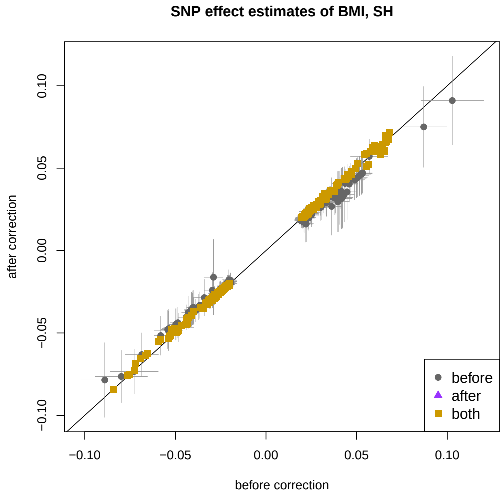

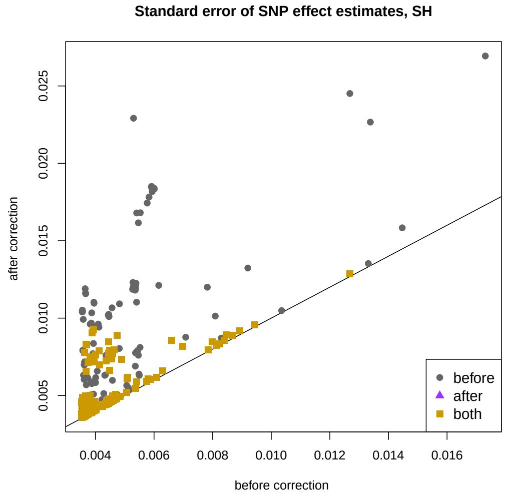

(m) **(n)**

Fig AJ. Effect estimates (in M_1) of BMI before and after bias correction. Horizontal and vertical bars represent 1 SE of an estimate before and after correction respectively. SEs are given in the right column. All 20 metabolomic PCs are used. 1 metabolomic PC is adjusted for bias correction. In the legends, "before" refers to the SNPs that are significant only before applying bias correction, "after" refers to the SNPs that are significant only after bias correction, "both" refers to the SNPs that are significant both before and after bias correction.

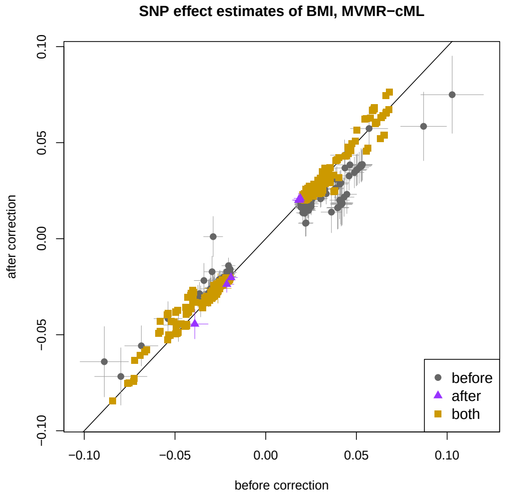
SNP effect estimates of BMI, MVMR−cML
after correction
before correction
before
after
both

(a)

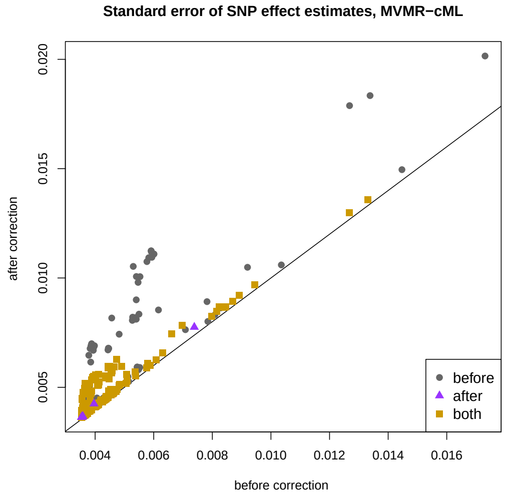
Standard error of SNP effect estimates, MVMR−cML
after correction
before correction
before
after
both

(b)

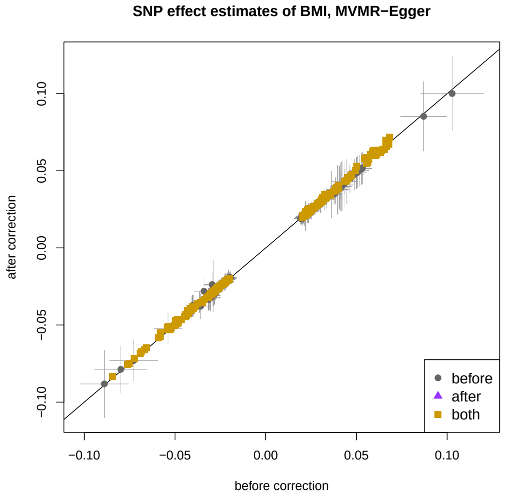
SNP effect estimates of BMI, MVMR−Egger
after correction
before correction
before
after
both

(c)

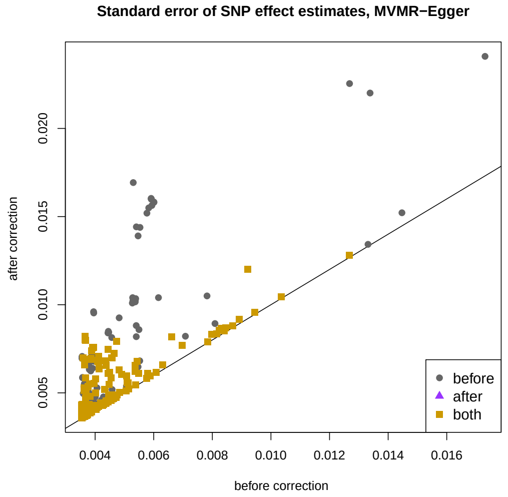
Standard error of SNP effect estimates, MVMR−Egger
after correction
before correction
before
after
both

(d)

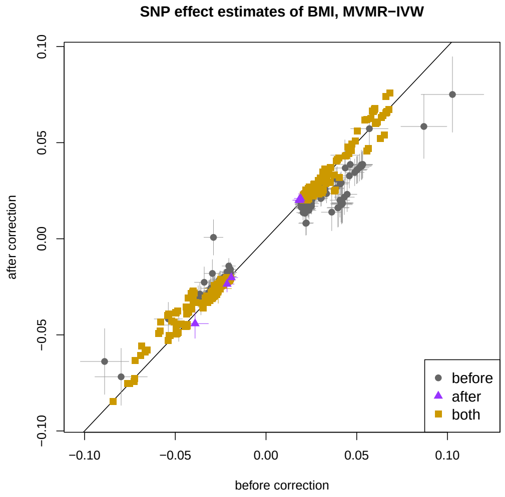
SNP effect estimates of BMI, MVMR−IVW
after correction
before correction
before
after
both

(e)

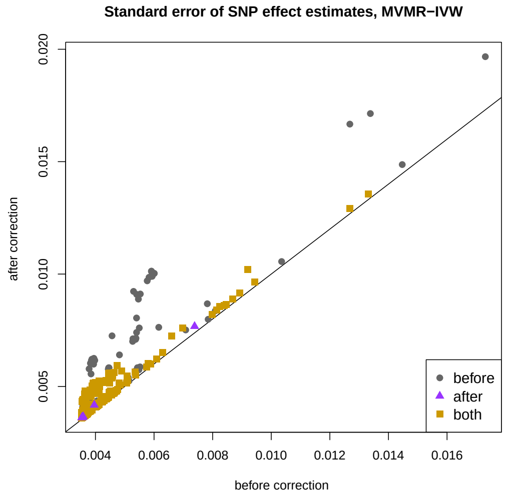
Standard error of SNP effect estimates, MVMR−IVW
after correction
before correction
before
after
both

(f)

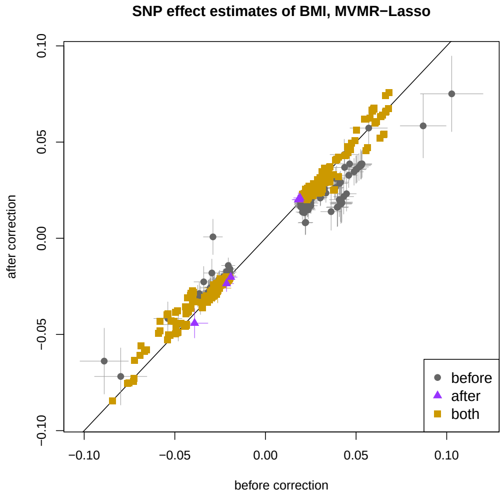

(g)

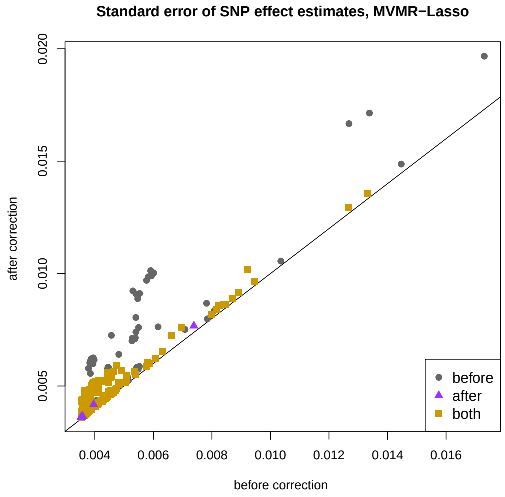

(h)

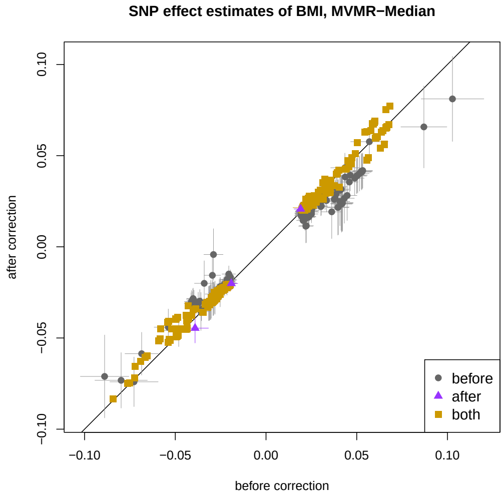

(i)

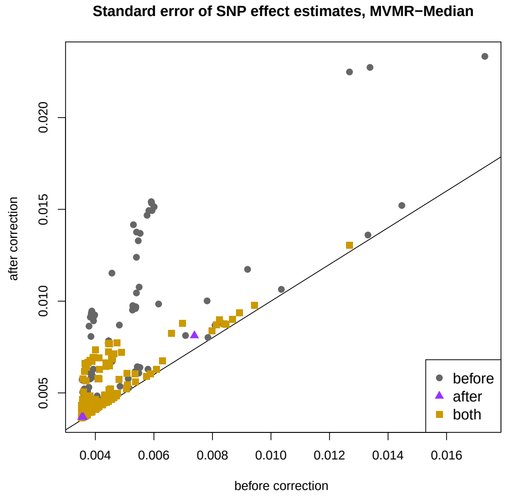

(j)

Fig AK. Effect estimates (in M_1) of BMI before and after bias correction. Horizontal and vertical bars represent 1 SE of an estimate before and after correction respectively. SEs are given in the right column. All 20 metabolomic PCs are used. 2 metabolomic PCs are adjusted for bias correction. In the legends, "before" refers to the SNPs that are significant only before applying bias correction, "after" refers to the SNPs that are significant only after bias correction, "both" refers to the SNPs that are significant both before and after bias correction.

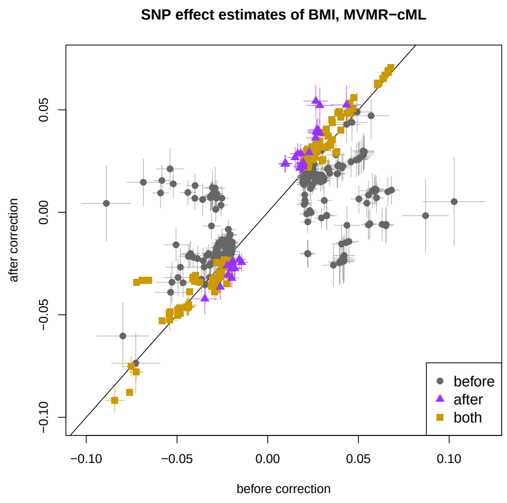
SNP effect estimates of BMI, MVMR−cML
after correction
before correction
before
after
both

(a)

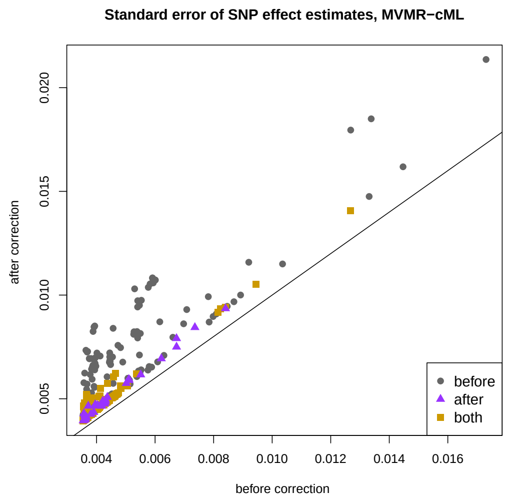
Standard error of SNP effect estimates, MVMR−cML
after correction
before correction
before
after
both

(b)

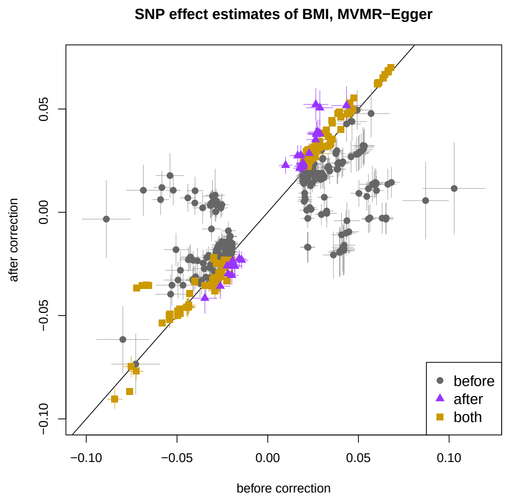
SNP effect estimates of BMI, MVMR−Egger
after correction
before correction
before
after
both

(c)

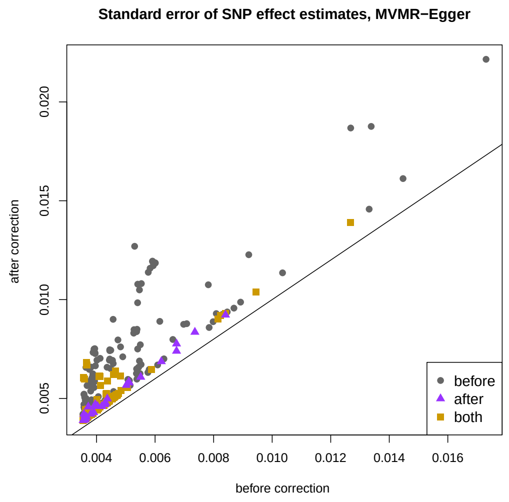
Standard error of SNP effect estimates, MVMR−Egger
after correction
before correction
before
after
both

(d)

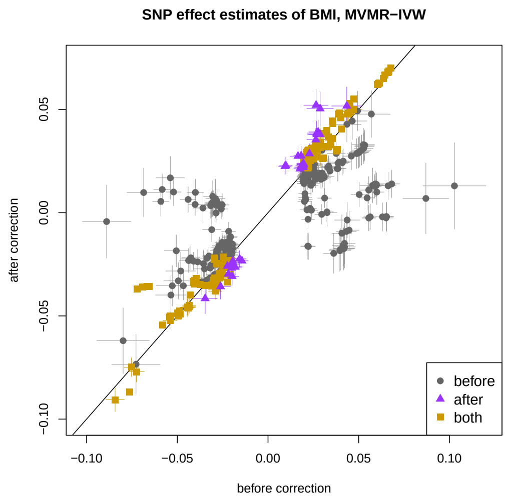
SNP effect estimates of BMI, MVMR−IVW
after correction
before correction
before
after
both

(e)

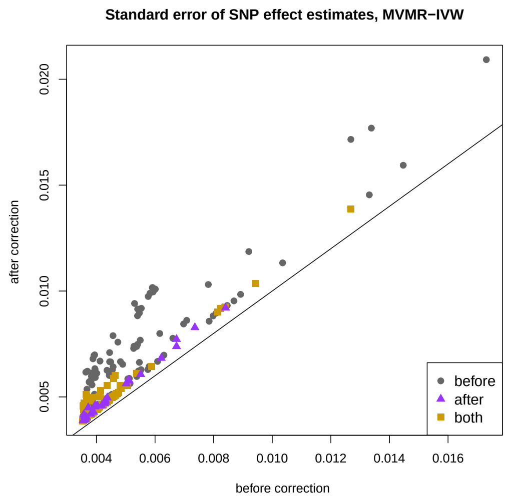
Standard error of SNP effect estimates, MVMR−IVW
after correction
before correction
before
after
both

(f)

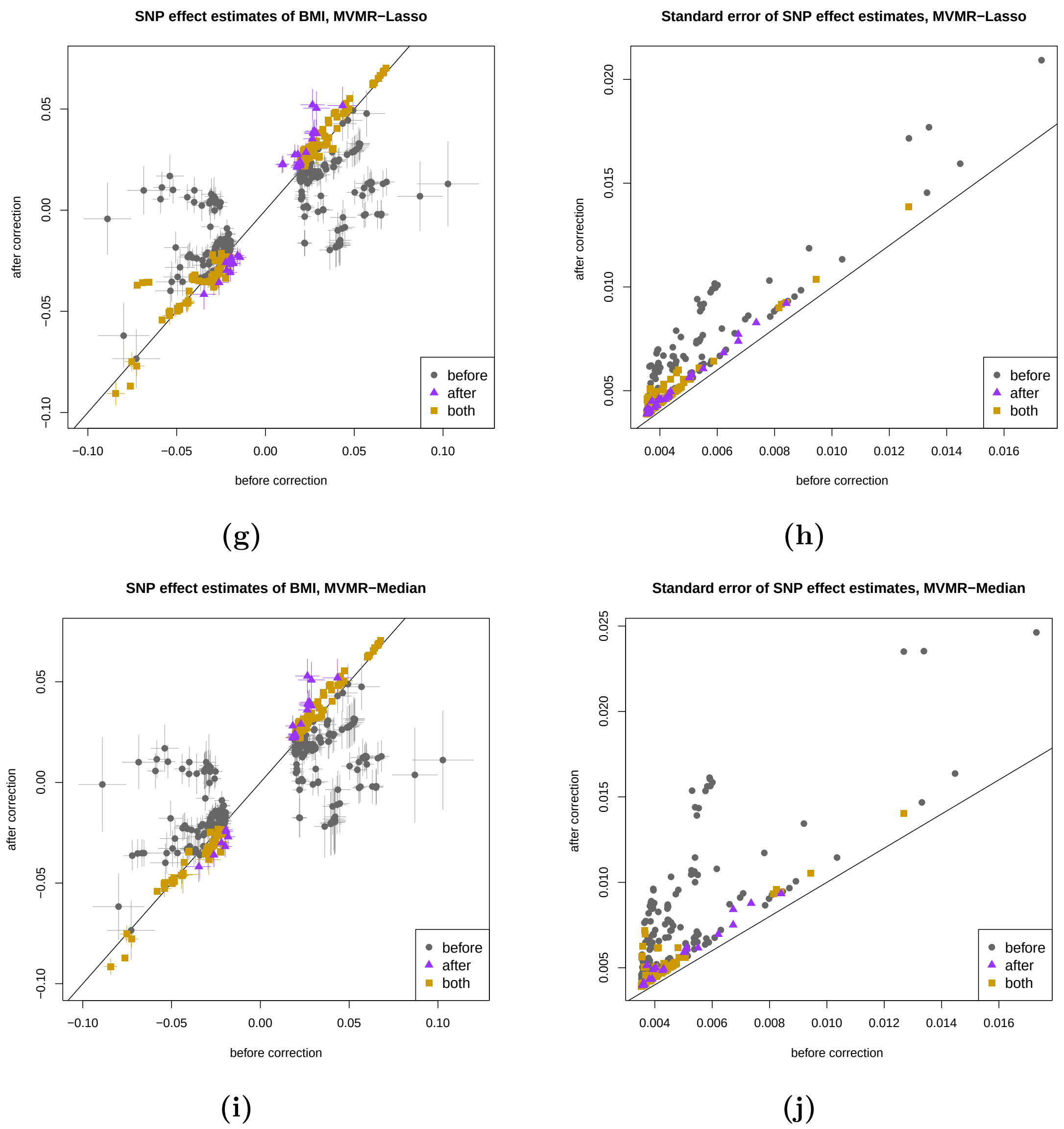

Fig AL. Effect estimates (in M_1) of BMI before and after bias correction. Horizontal and vertical bars represent 1 SE of an estimate before and after correction respectively. SEs are given in the right column. All 20 metabolomic PCs are used. 3 metabolomic PCs are adjusted for bias correction. In the legends, "before" refers to the SNPs that are significant only before applying bias correction, "after" refers to the SNPs that are significant only after bias correction, "both" refers to the SNPs that are significant both before and after bias correction.

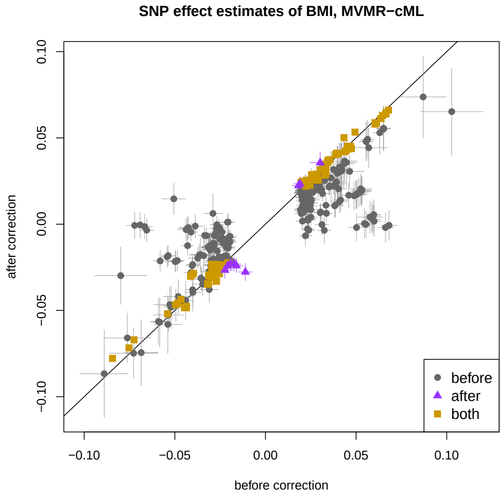
SNP effect estimates of BMI, MVMR−cML
after correction
before correction
−0.10
−0.05
0.00
0.05
0.10
before
after
both

(a)

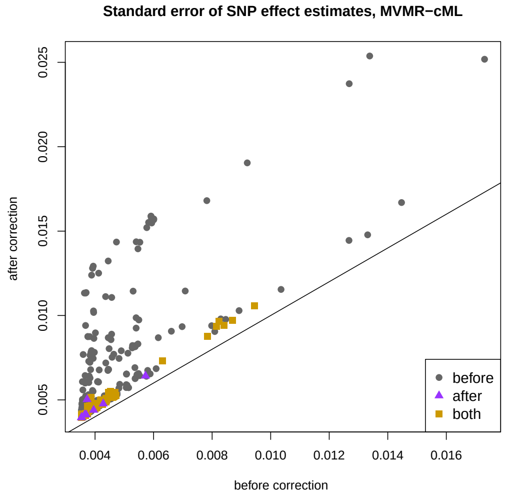
Standard error of SNP effect estimates, MVMR−cML
after correction
before correction
0.005
0.010
0.015
0.020
0.025
0.004
0.006
0.008
0.010
0.012
0.014
0.016
before
after
both

(b)

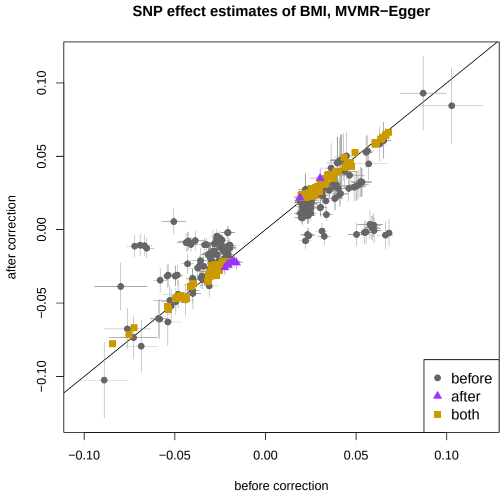
SNP effect estimates of BMI, MVMR−Egger
after correction
before correction
−0.10
−0.05
0.00
0.05
0.10
before
after
both

(c)

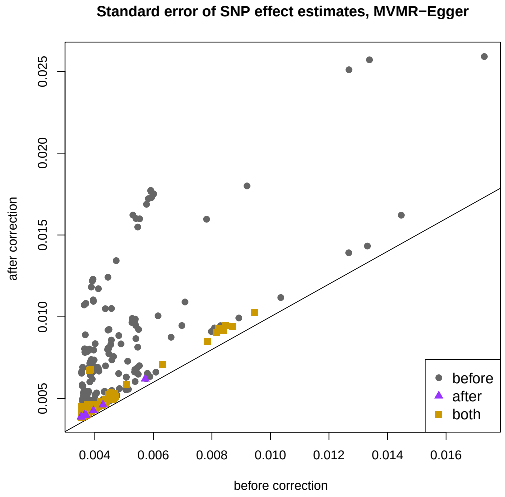
Standard error of SNP effect estimates, MVMR−Egger
after correction
before correction
0.005
0.010
0.015
0.020
0.025
0.004
0.006
0.008
0.010
0.012
0.014
0.016
before
after
both

(d)

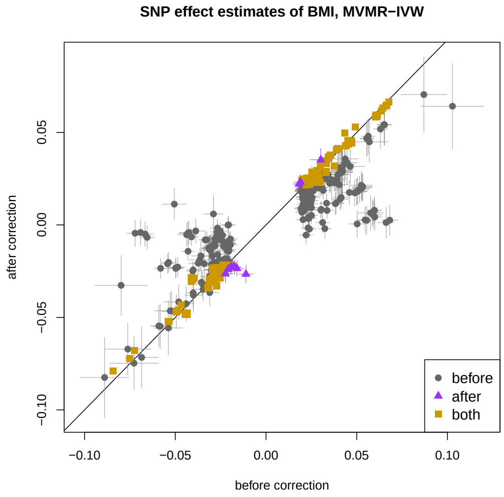
SNP effect estimates of BMI, MVMR−IVW
after correction
before correction
−0.10
−0.05
0.00
0.05
0.10
before
after
both

(e)

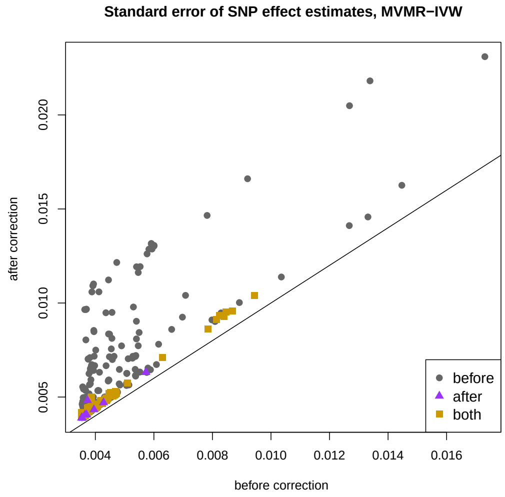
Standard error of SNP effect estimates, MVMR−IVW
after correction
before correction
0.005
0.010
0.015
0.020
0.004
0.006
0.008
0.010
0.012
0.014
0.016
before
after
both

(f)

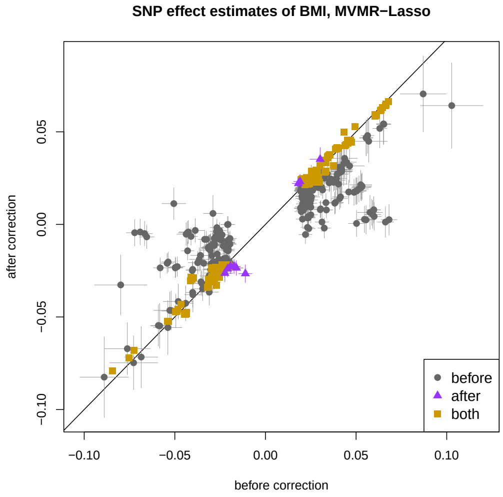

(g)

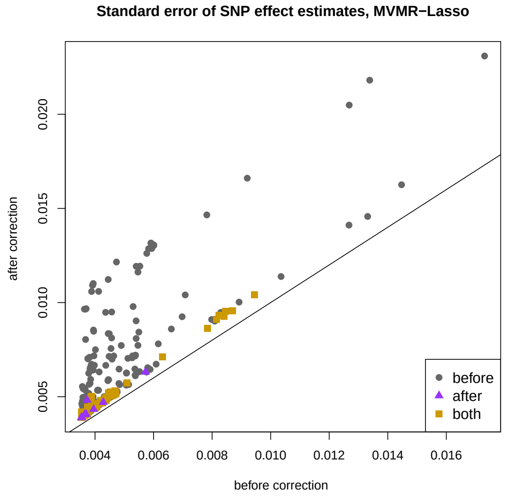

(h)

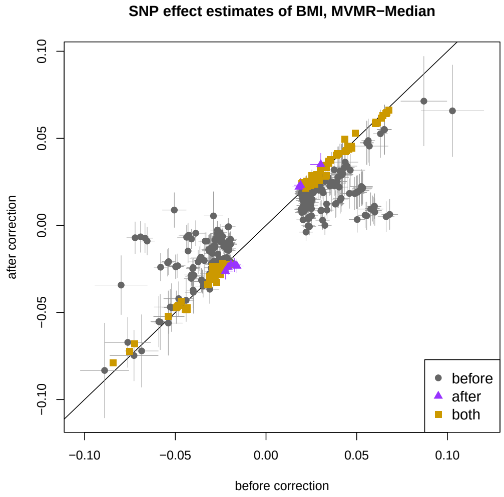

(i)

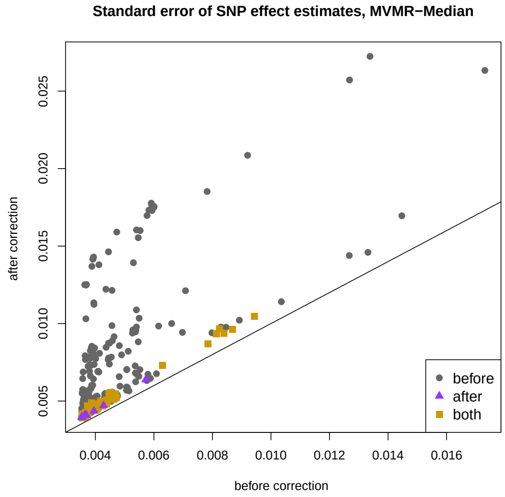

(j)

Fig AM. Effect estimates (in M_1) of BMI before and after bias correction. Horizontal and vertical bars represent 1 SE of an estimate before and after correction respectively. SEs are given in the right column. All 20 metabolomic PCs are used. 4 metabolomic PCs are adjusted for bias correction. In the legends, "before" refers to the SNPs that are significant only before applying bias correction, "after" refers to the SNPs that are significant only after bias correction, "both" refers to the SNPs that are significant both before and after bias correction.

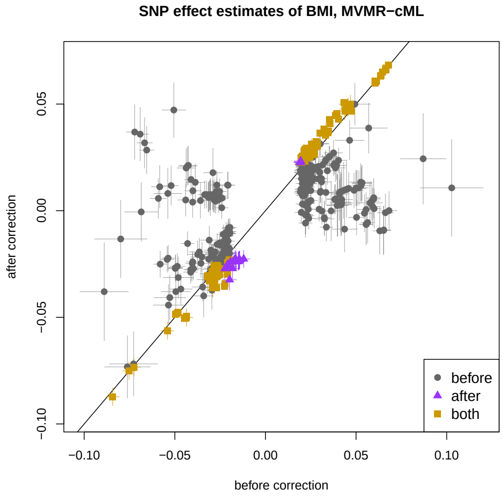
SNP effect estimates of BMI, MVMR−cML
after correction
before correction
−0.10
−0.05
0.00
0.05
0.10
before
after
both

(a)

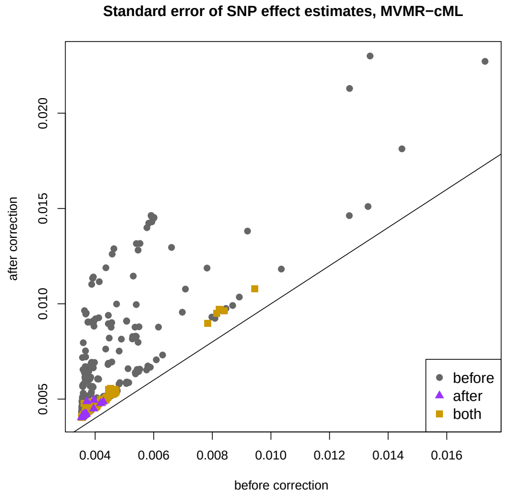
Standard error of SNP effect estimates, MVMR−cML
after correction
before correction
0.005
0.010
0.015
0.020
0.004
0.006
0.008
0.010
0.012
0.014
0.016
before
after
both

(b)

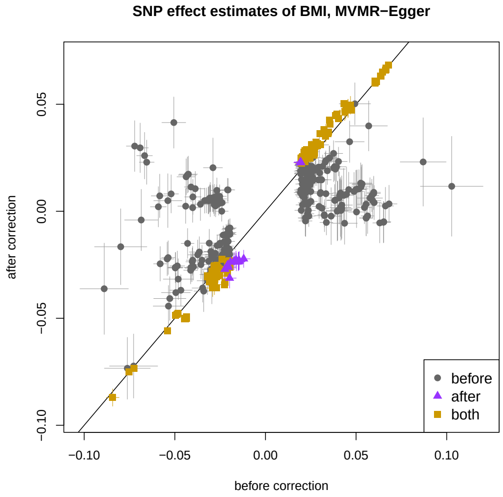
SNP effect estimates of BMI, MVMR−Egger
after correction
before correction
−0.10
−0.05
0.00
0.05
0.10
before
after
both

(c)

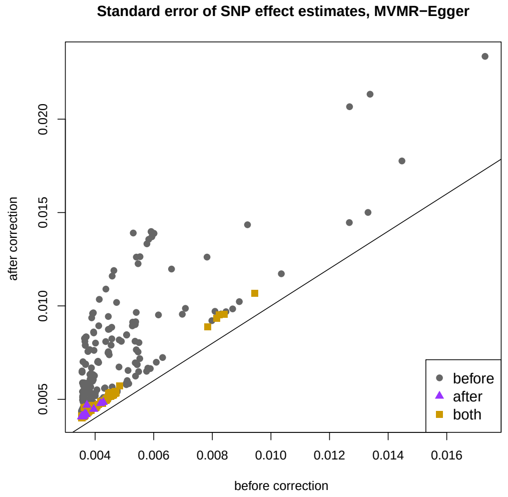
Standard error of SNP effect estimates, MVMR−Egger
after correction
before correction
0.005
0.010
0.015
0.020
0.004
0.006
0.008
0.010
0.012
0.014
0.016
before
after
both

(d)

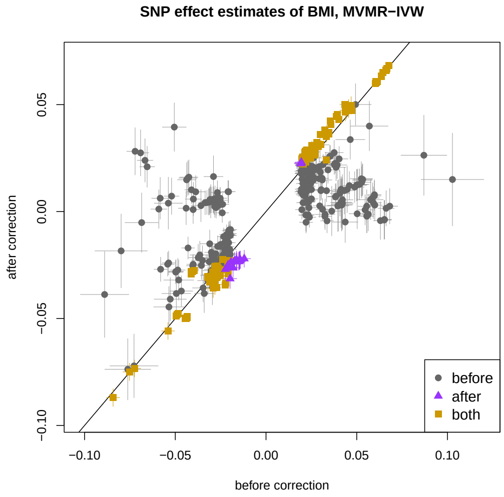
SNP effect estimates of BMI, MVMR−IVW
after correction
before correction
−0.10
−0.05
0.00
0.05
0.10
before
after
both

(e)

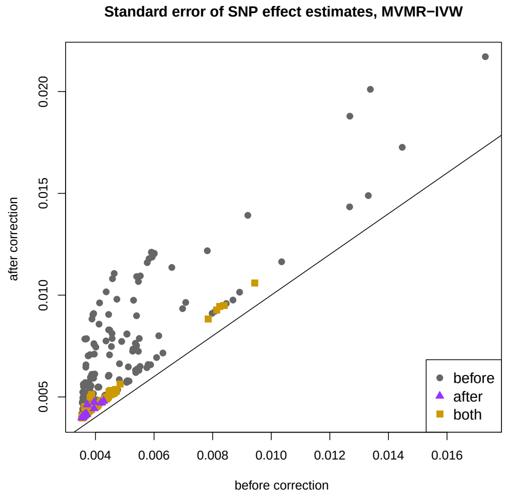
Standard error of SNP effect estimates, MVMR−IVW
after correction
before correction
0.005
0.010
0.015
0.020
0.004
0.006
0.008
0.010
0.012
0.014
0.016
before
after
both

(f)

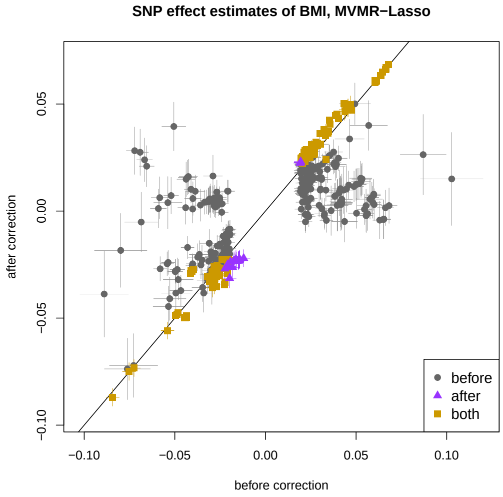

(g)

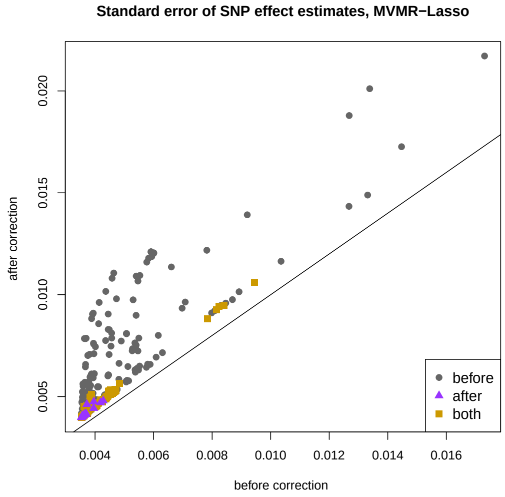

(h)

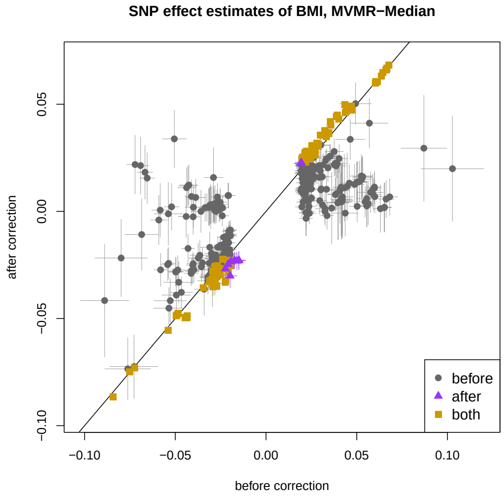

(i)

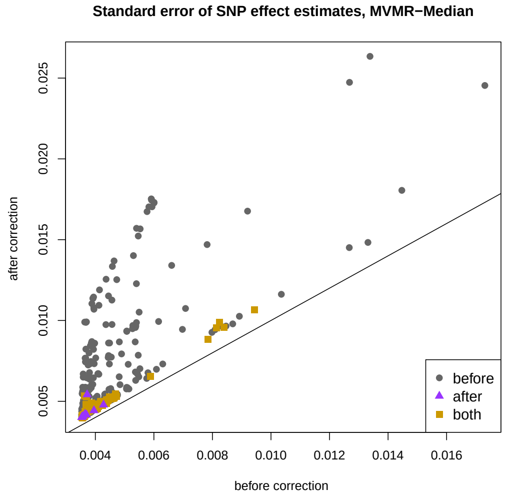

(j)

Fig AN. Effect estimates (in M_1) of BMI before and after bias correction. Horizontal and vertical bars represent 1 SE of an estimate before and after correction respectively. SEs are given in the right column. All 20 metabolomic PCs are used. 5 metabolomic PCs are adjusted for bias correction. In the legends, "before" refers to the SNPs that are significant only before applying bias correction, "after" refers to the SNPs that are significant only after bias correction, "both" refers to the SNPs that are significant both before and after bias correction.

G.2.5 Comparison of SNP effect estimates before and after apply different bias-correction methods on M_2

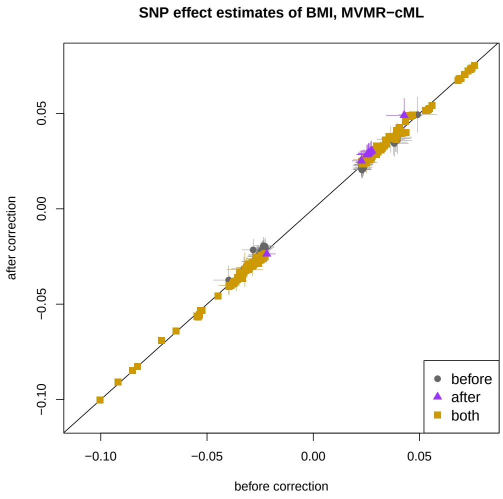

(a)

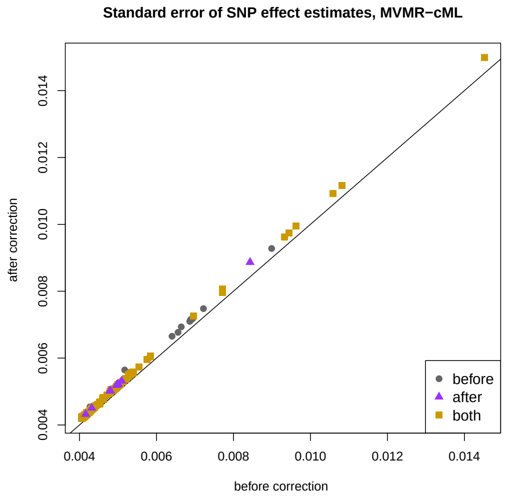

(b)

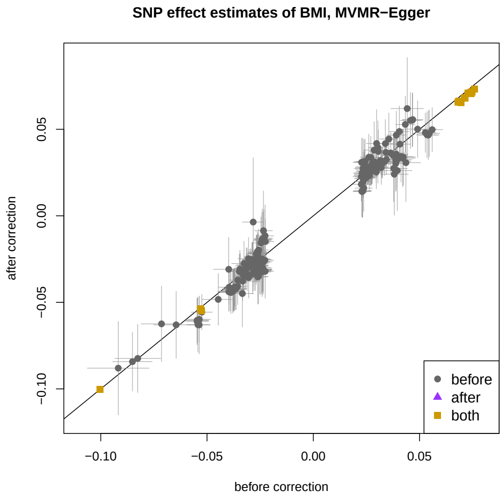

(c)

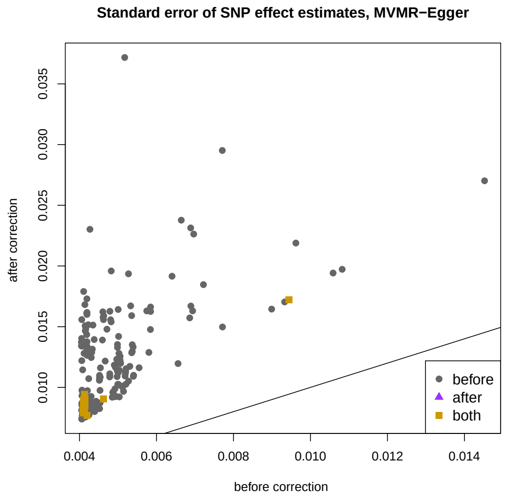

(d)

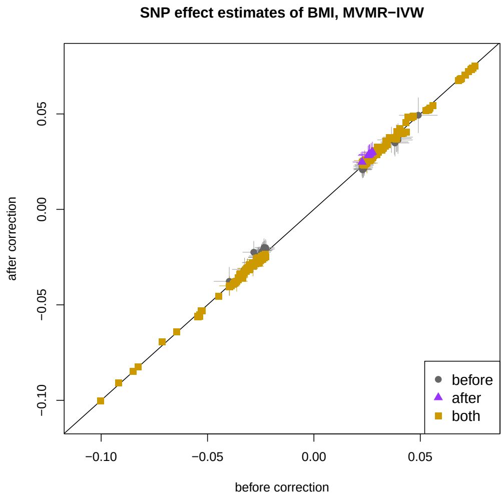

(e)

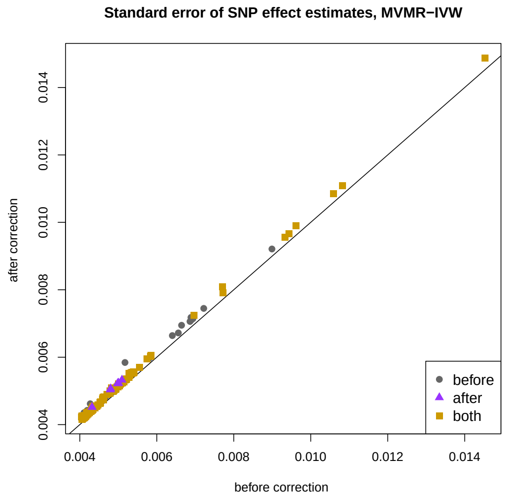

(f)

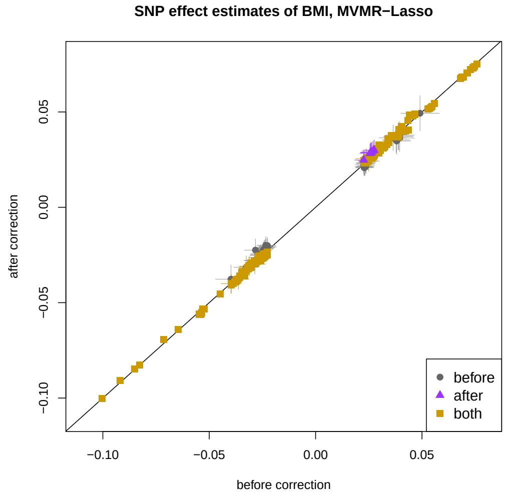
SNP effect estimates of BMI, MVMR−Lasso
after correction
before correction
before
after
both

(g)

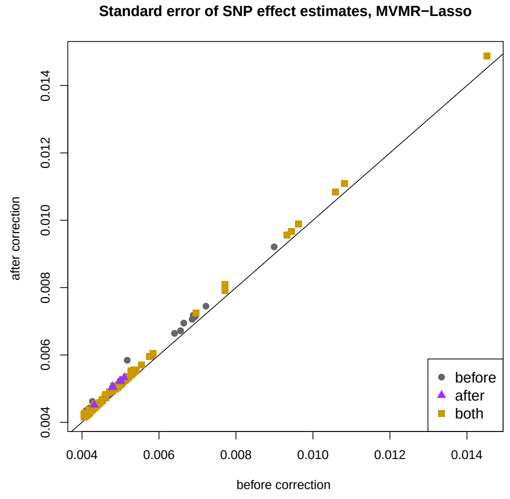
Standard error of SNP effect estimates, MVMR−Lasso
after correction
before correction
before
after
both

(h)

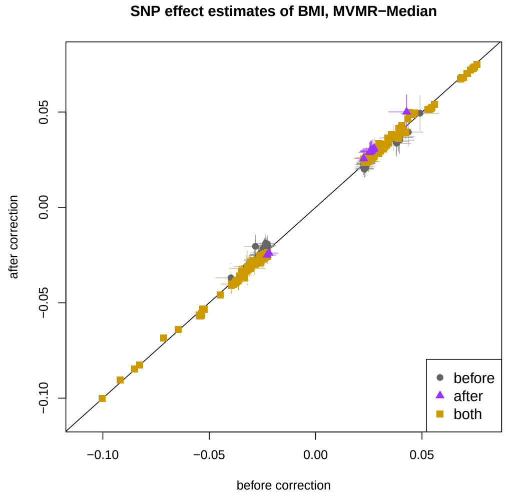
SNP effect estimates of BMI, MVMR−Median
after correction
before correction
before
after
both

(i)

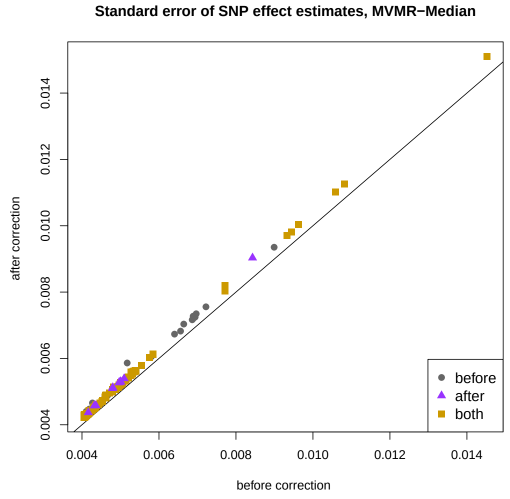
Standard error of SNP effect estimates, MVMR−Median
after correction
before correction
before
after
both

(j)

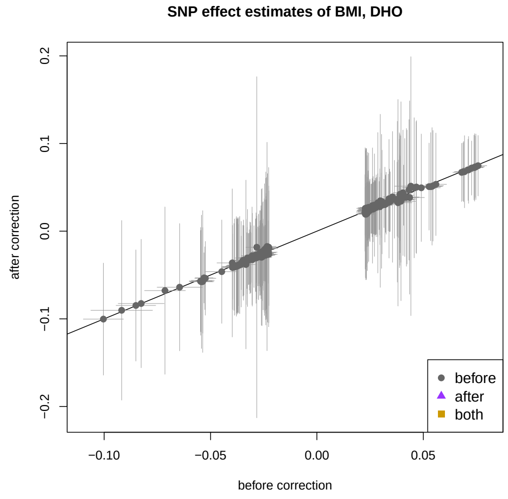
SNP effect estimates of BMI, DHO
after correction
before correction
before
after
both

(k)

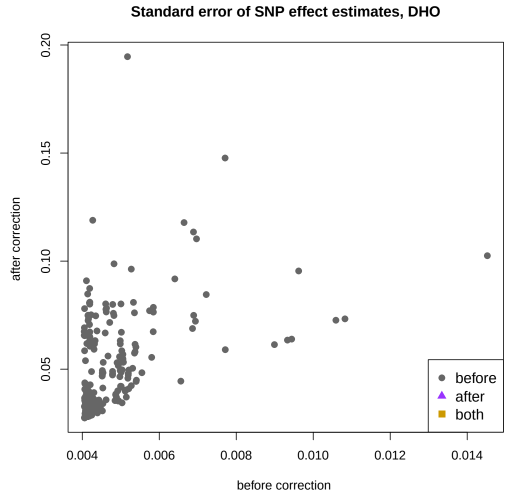
Standard error of SNP effect estimates, DHO
after correction
before correction
before
after
both

(l)

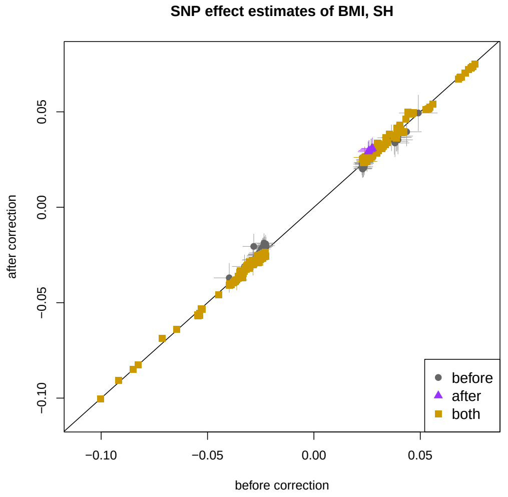

(m)

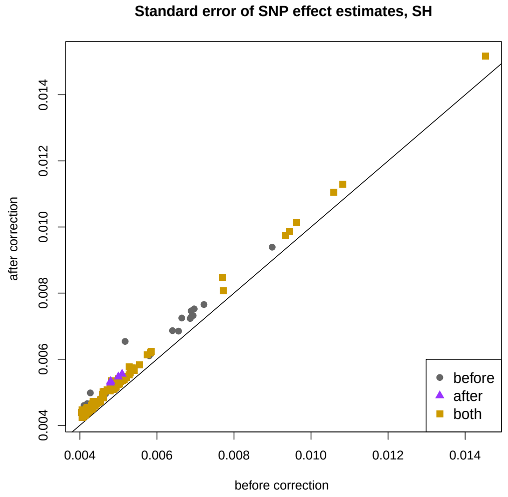

(n)

Fig AO. Effect estimates (in M_2) of BMI before and after bias correction. Horizontal and vertical bars represent 1 SE of an estimate before and after correction respectively. SEs are given in the right column. 1 metabolomic PC is used. In the legends, “before” refers to the SNPs that are significant only before applying bias correction, “after” refers to the SNPs that are significant only after bias correction, “both” refers to the SNPs that are significant both before and after bias correction.

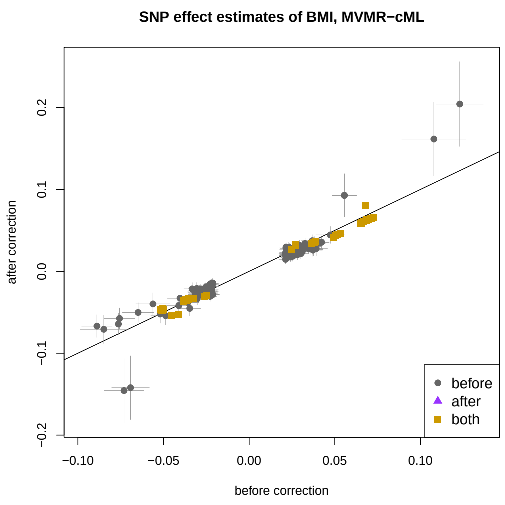
SNP effect estimates of BMI, MVMR−cML
after correction
before correction
before
after
both

(a)

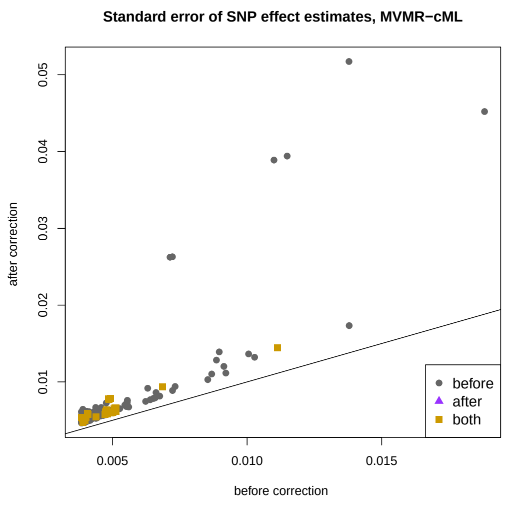
Standard error of SNP effect estimates, MVMR−cML
after correction
before correction
before
after
both

(b)

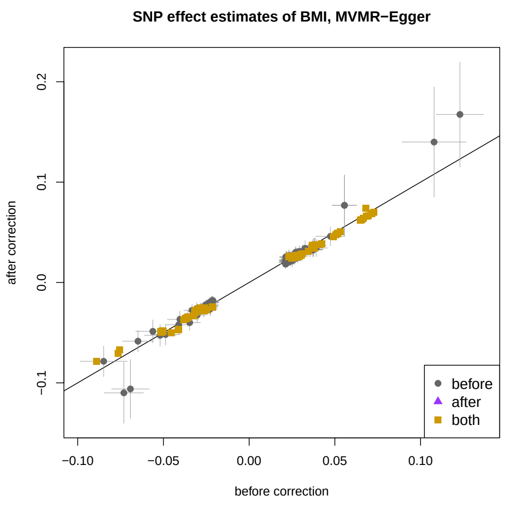
SNP effect estimates of BMI, MVMR−Egger
after correction
before correction
before
after
both

(c)

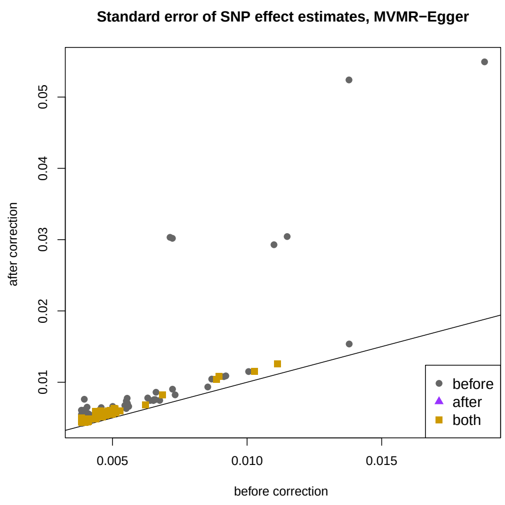
Standard error of SNP effect estimates, MVMR−Egger
after correction
before correction
before
after
both

(d)

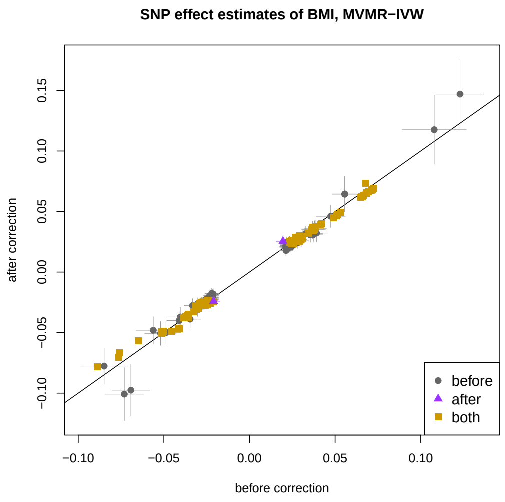
SNP effect estimates of BMI, MVMR−IVW
after correction
before correction
before
after
both

(e)

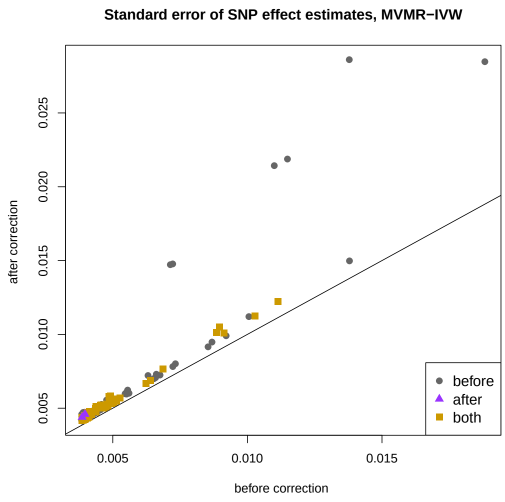
Standard error of SNP effect estimates, MVMR−IVW
after correction
before correction
before
after
both

(f)

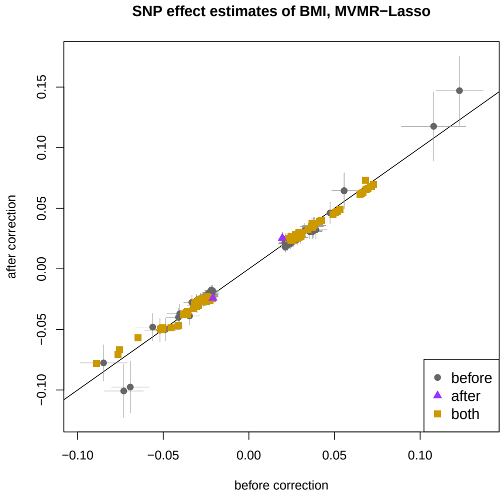

(g)

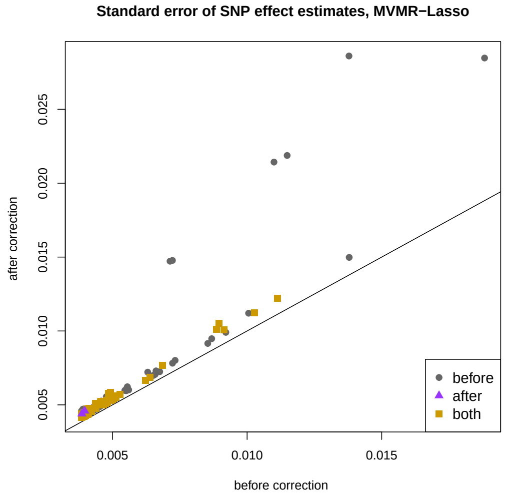

(h)

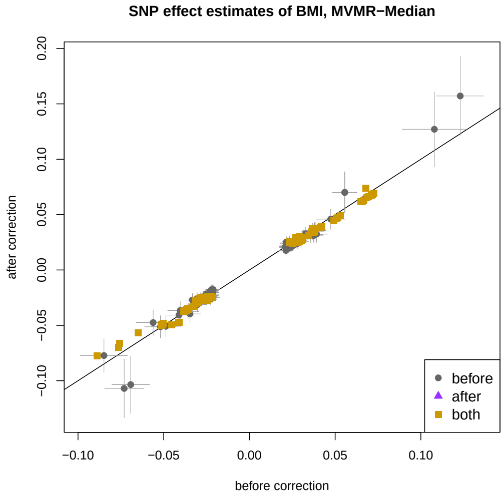

(i)

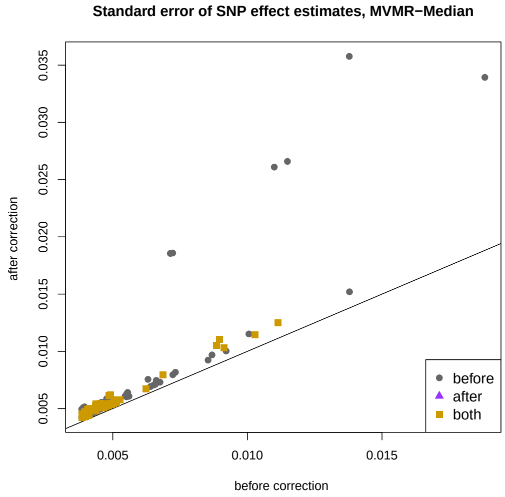

(j)

Fig AP. Effect estimates (in M_2) of BMI before and after bias correction. Horizontal and vertical bars represent 1 SE of an estimate before and after correction respectively. SEs are given in the right column. 2 metabolomic PCs are used. In the legends, “before” refers to the SNPs that are significant only before applying bias correction, “after” refers to the SNPs that are significant only after bias correction, “both” refers to the SNPs that are significant both before and after bias correction.

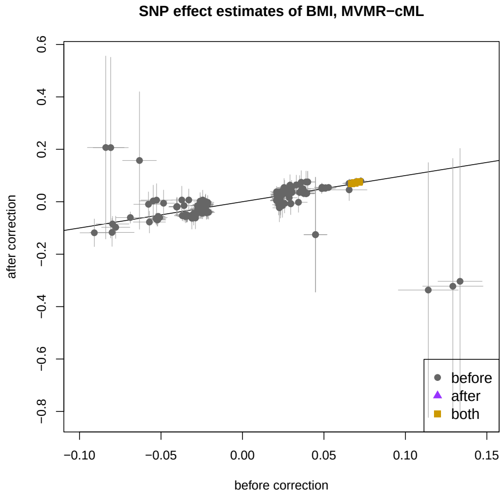
SNP effect estimates of BMI, MVMR−cML
after correction
before correction
−0.8
−0.6
−0.4
−0.2
0.0
0.2
0.4
0.6
−0.10
−0.05
0.00
0.05
0.10
0.15
before
after
both

(a)

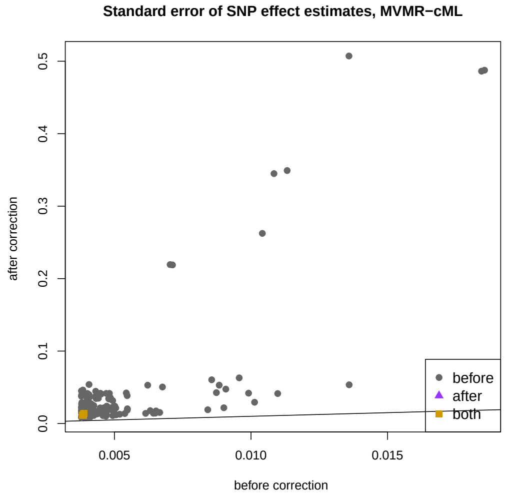
Standard error of SNP effect estimates, MVMR−cML
after correction
before correction
0.0
0.1
0.2
0.3
0.4
0.5
0.005
0.010
0.015
before
after
both

(b)

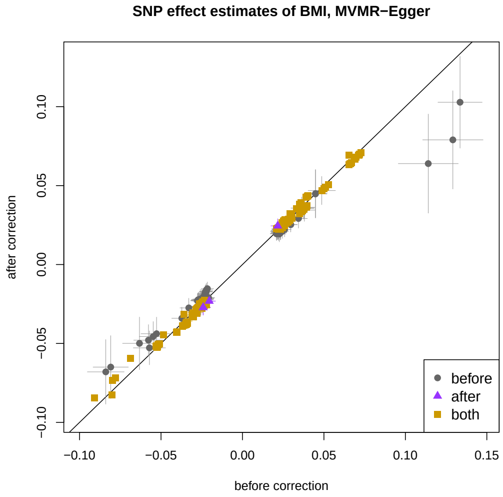
SNP effect estimates of BMI, MVMR−Egger
after correction
before correction
−0.10
−0.05
0.00
0.05
0.10
−0.10
−0.05
0.00
0.05
0.10
0.15
before
after
both

(c)

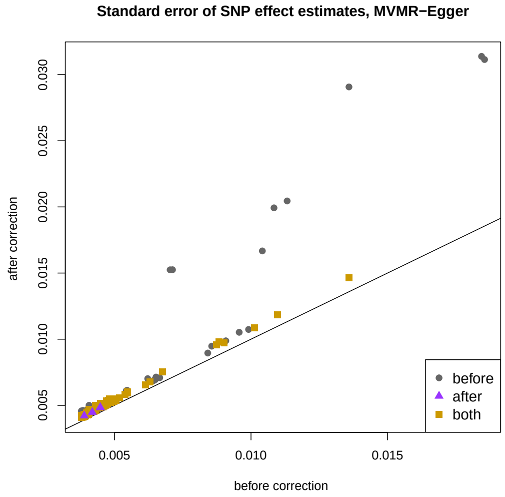
Standard error of SNP effect estimates, MVMR−Egger
after correction
before correction
0.005
0.010
0.015
0.020
0.025
0.030
0.005
0.010
0.015
before
after
both

(d)

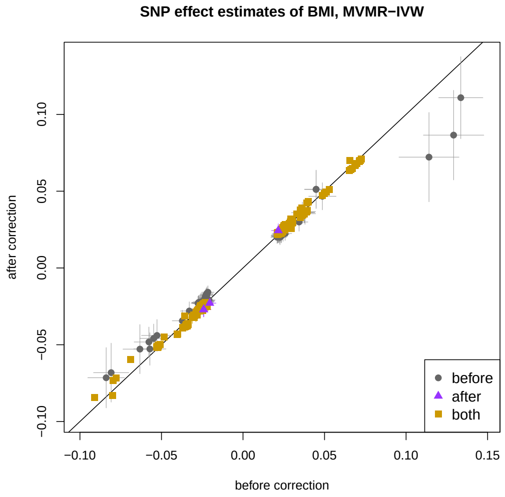
SNP effect estimates of BMI, MVMR−IVW
after correction
before correction
−0.10
−0.05
0.00
0.05
0.10
−0.10
−0.05
0.00
0.05
0.10
0.15
before
after
both

(e)

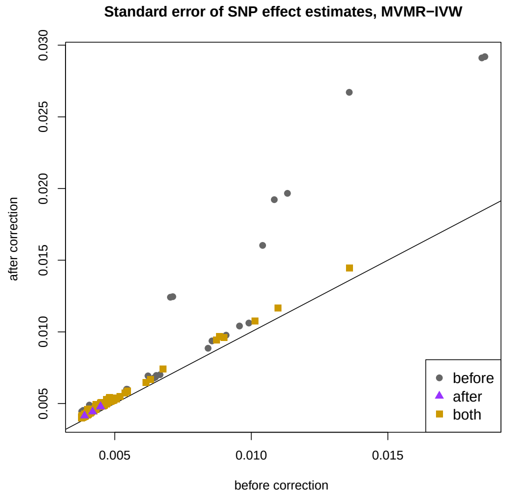
Standard error of SNP effect estimates, MVMR−IVW
after correction
before correction
0.005
0.010
0.015
0.020
0.025
0.030
0.005
0.010
0.015
before
after
both

(f)

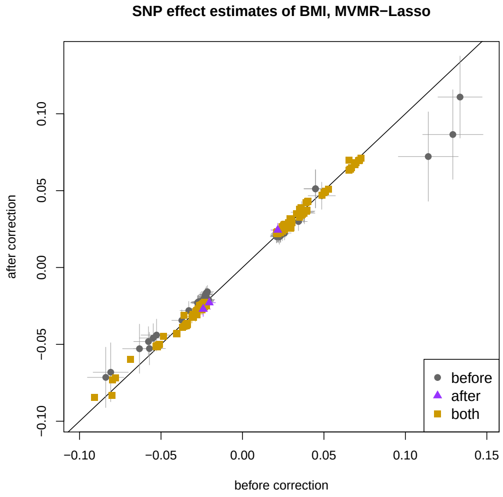

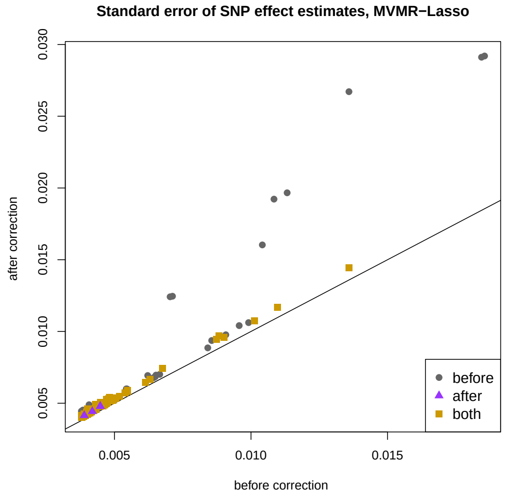

(g) **(h)**

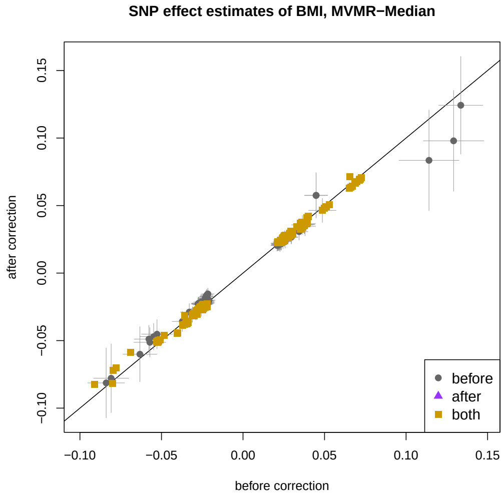

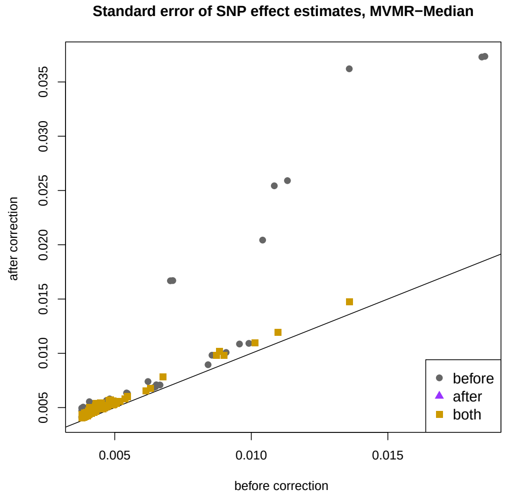

(i) **(j)**

Fig AQ. Effect estimates (in M_2) of BMI before and after bias correction. Horizontal and vertical bars represent 1 SE of an estimate before and after correction respectively. SEs are given in the right column. 3 metabolomic PCs are used. In the legends, "before" refers to the SNPs that are significant only before applying bias correction, "after" refers to the SNPs that are significant only after bias correction, "both" refers to the SNPs that are significant both before and after bias correction.

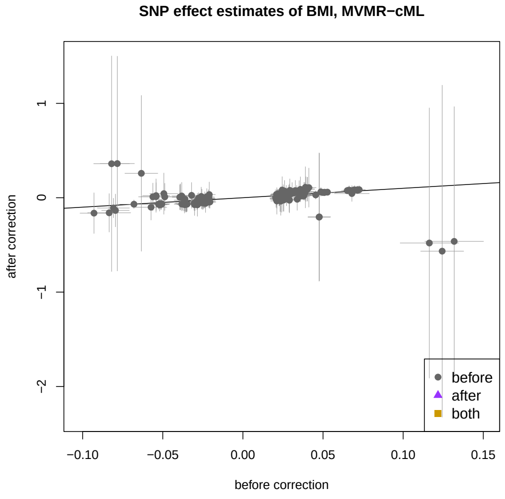
SNP effect estimates of BMI, MVMR−cML
after correction
before correction
before
after
both

(a)

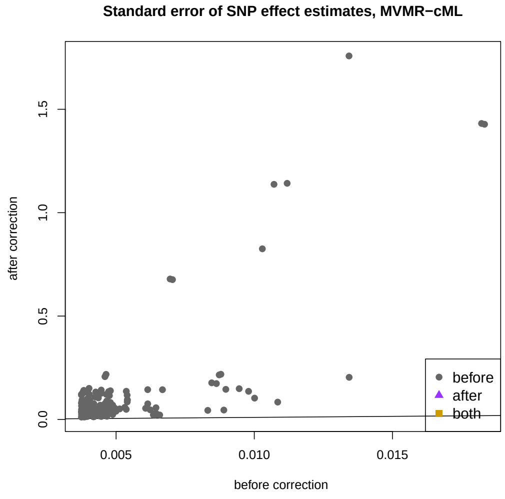
Standard error of SNP effect estimates, MVMR−cML
after correction
before correction
before
after
both

(b)

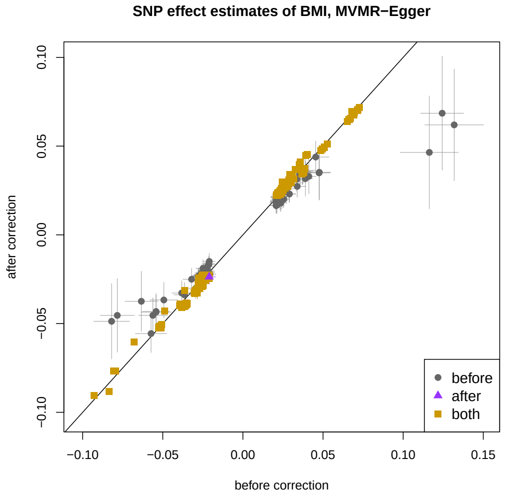
SNP effect estimates of BMI, MVMR−Egger
after correction
before correction
before
after
both

(c)

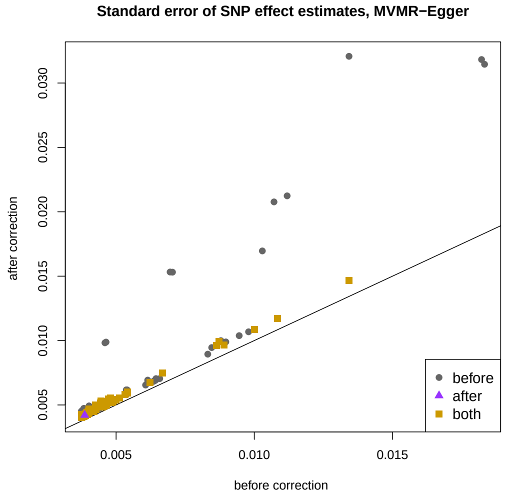
Standard error of SNP effect estimates, MVMR−Egger
after correction
before correction
before
after
both

(d)

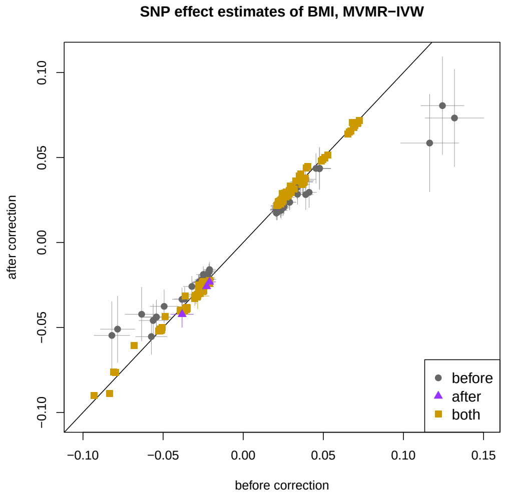
SNP effect estimates of BMI, MVMR−IVW
after correction
before correction
before
after
both

(e)

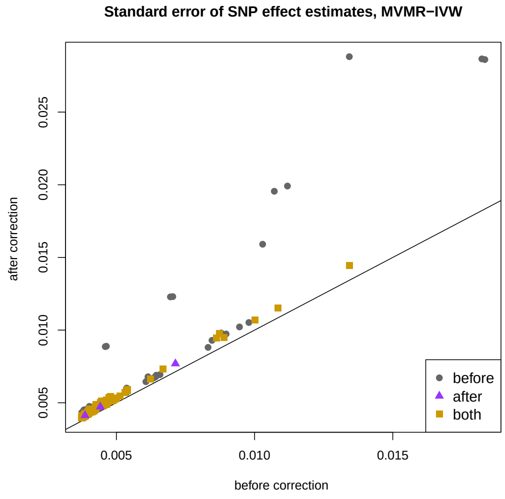
Standard error of SNP effect estimates, MVMR−IVW
after correction
before correction
before
after
both

(f)

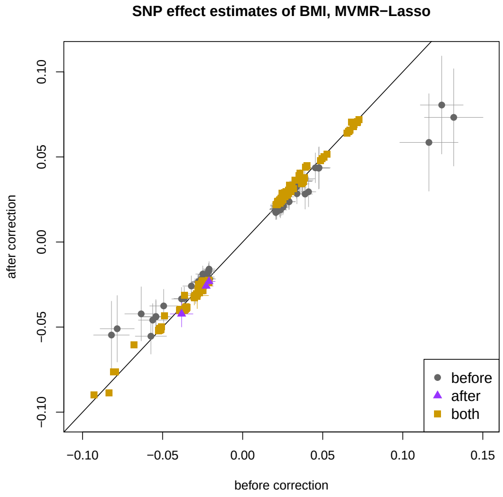

(g)

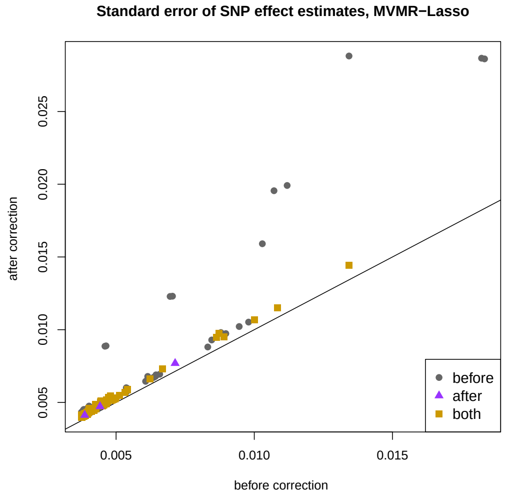

(h)

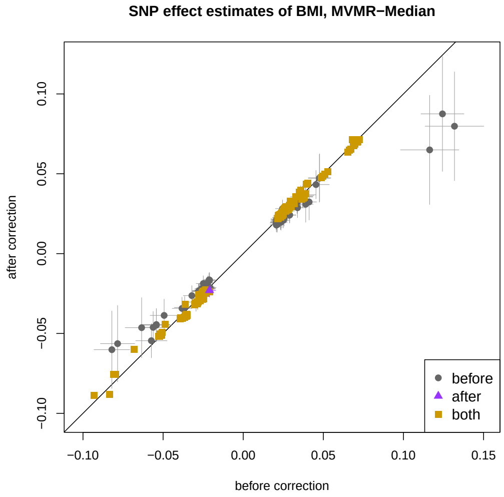

(i)

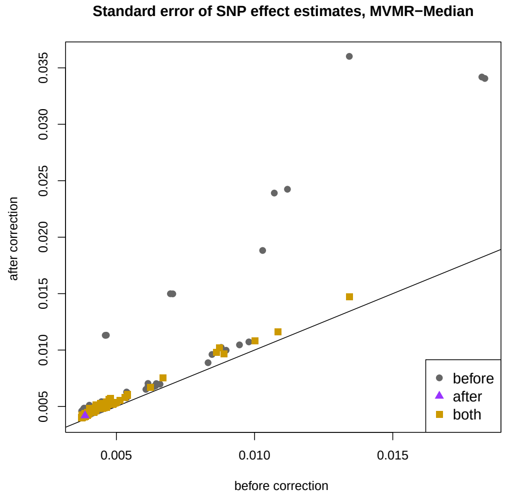

(j)

Fig AR. Effect estimates (in M_2) of BMI before and after bias correction. Horizontal and vertical bars represent 1 SE of an estimate before and after correction respectively. SEs are given in the right column. 4 metabolomic PCs are used. In the legends, "before" refers to the SNPs that are significant only before applying bias correction, "after" refers to the SNPs that are significant only after bias correction, "both" refers to the SNPs that are significant both before and after bias correction.

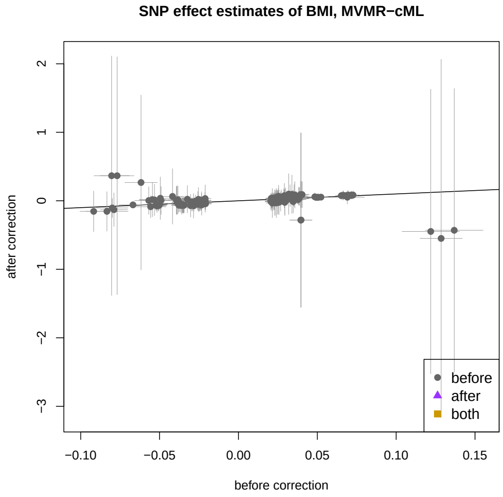
SNP effect estimates of BMI, MVMR−cML
after correction
before correction
before
after
both

(a)

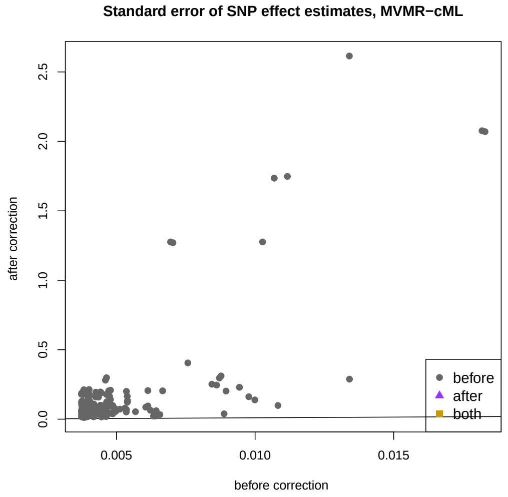
Standard error of SNP effect estimates, MVMR−cML
after correction
before correction
before
after
both

(b)

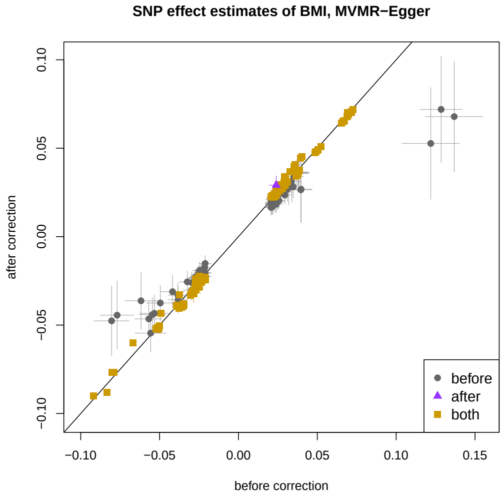
SNP effect estimates of BMI, MVMR−Egger
after correction
before correction
before
after
both

(c)

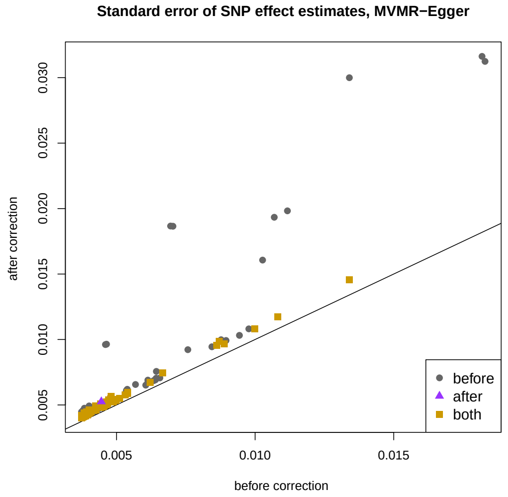
Standard error of SNP effect estimates, MVMR−Egger
after correction
before correction
before
after
both

(d)

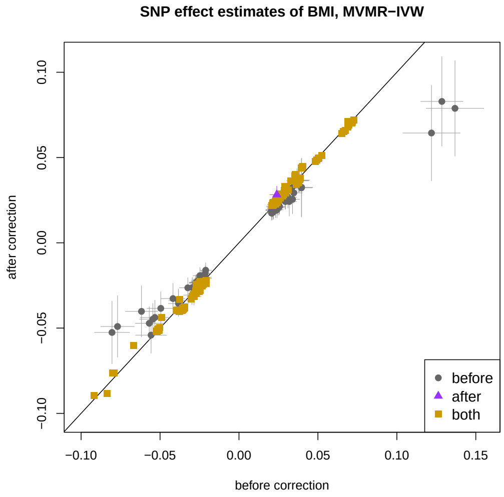
SNP effect estimates of BMI, MVMR−IVW
after correction
before correction
before
after
both

(e)

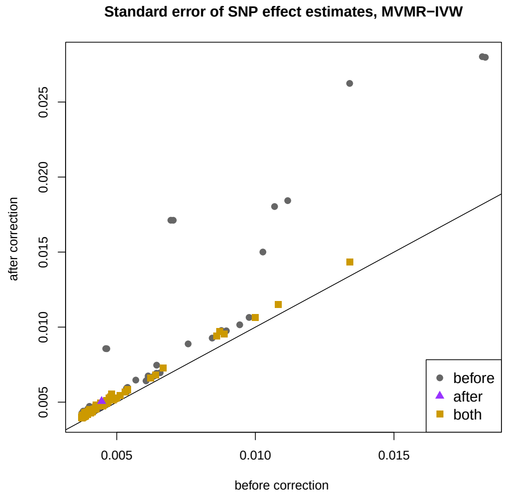
Standard error of SNP effect estimates, MVMR−IVW
after correction
before correction
before
after
both

(f)

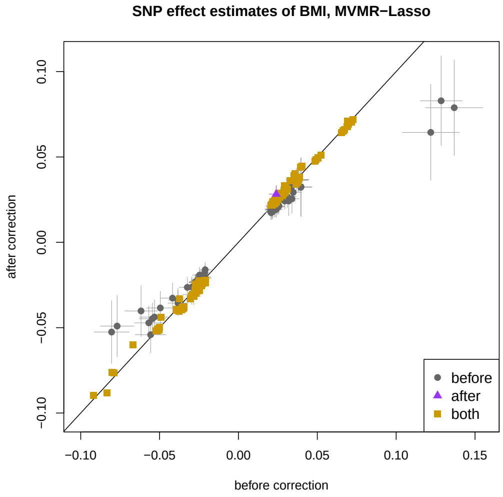

(g)

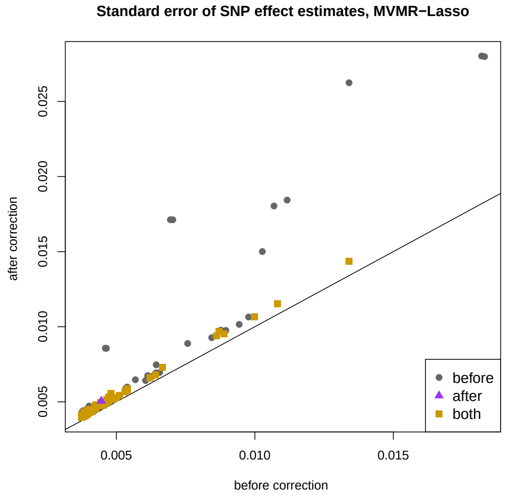

(h)

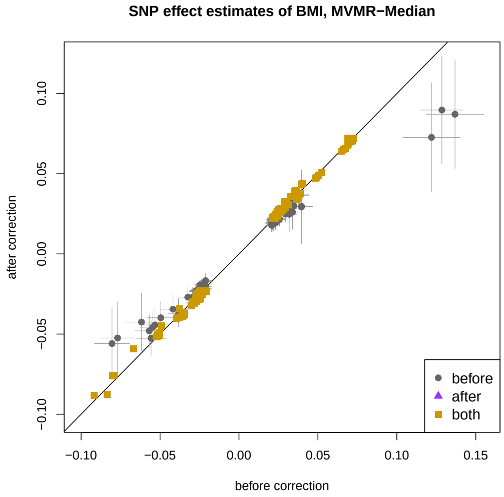

(i)

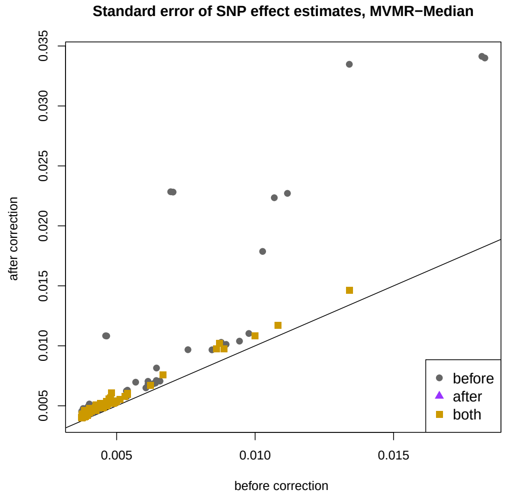

(j)

Fig AS. Effect estimates (in M_2) of BMI before and after bias correction. Horizontal and vertical bars represent 1 SE of an estimate before and after correction respectively. SEs are given in the right column. 5 metabolomic PCs are used. In the legends, "before" refers to the SNPs that are significant only before applying bias correction, "after" refers to the SNPs that are significant only after bias correction, "both" refers to the SNPs that are significant both before and after bias correction.

G.2.6 Comparison of SNP effect estimates befor and after apply different bias-correction methods on M_2, all 20 metabolomic PCs are used in M_2

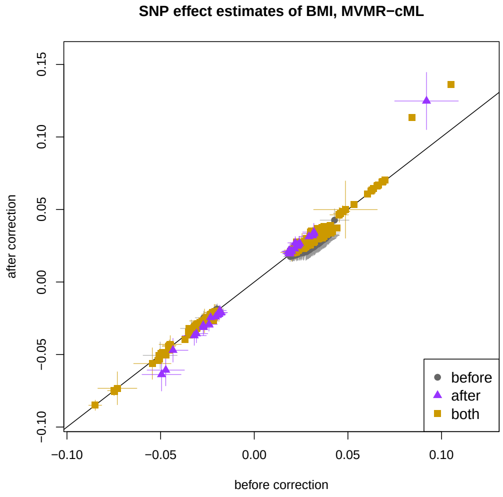

(a)

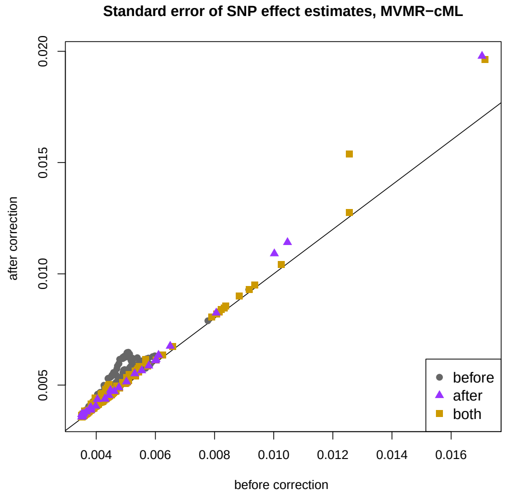

(b)

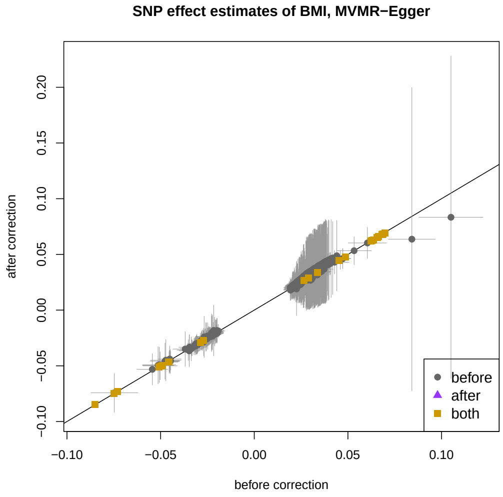

(c)

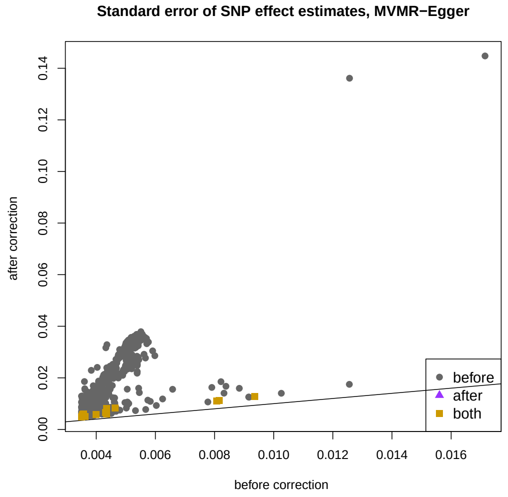

(d)

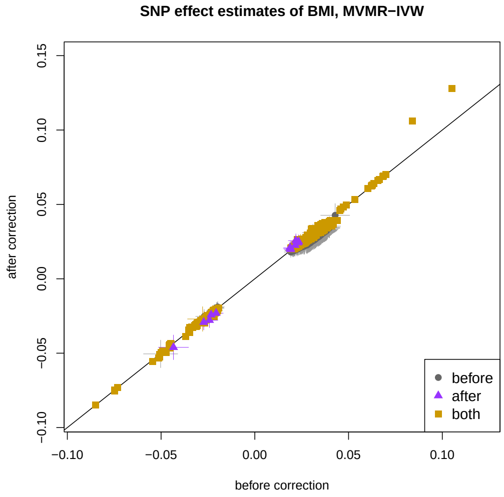

(e)

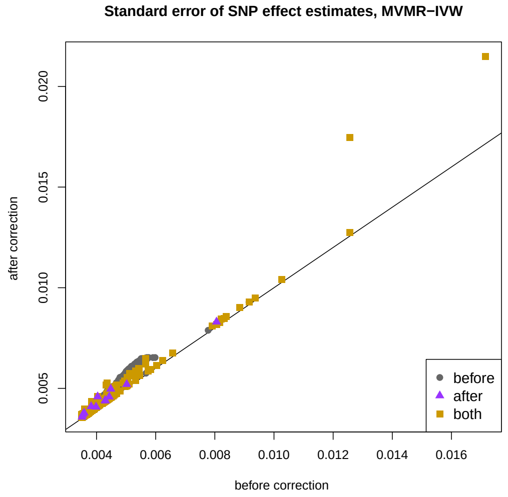

(f)

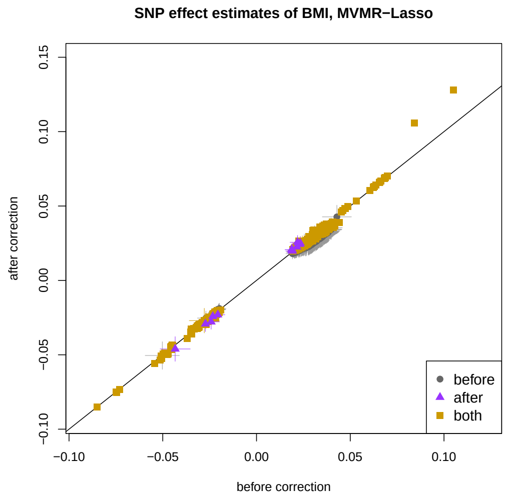
SNP effect estimates of BMI, MVMR−Lasso
after correction
before correction
−0.10
−0.05
0.00
0.05
0.10
0.15
before
after
both

(g)

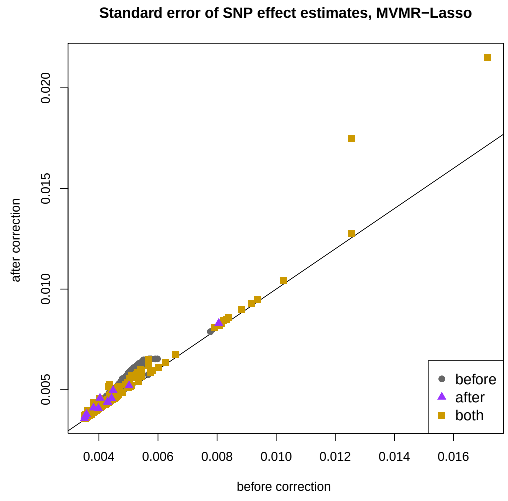
Standard error of SNP effect estimates, MVMR−Lasso
after correction
before correction
0.005
0.010
0.015
0.020
0.004
0.006
0.008
0.010
0.012
0.014
0.016
before
after
both

(h)

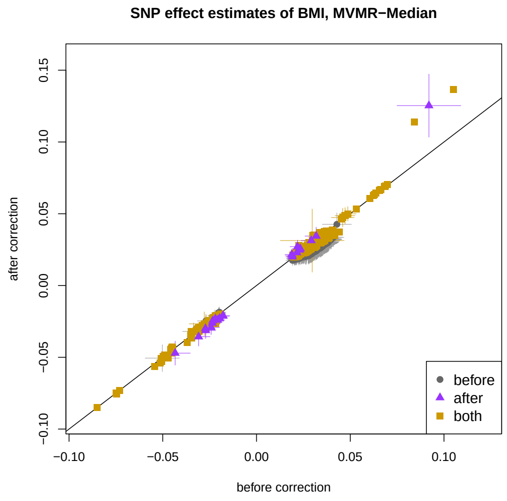
SNP effect estimates of BMI, MVMR−Median
after correction
before correction
−0.10
−0.05
0.00
0.05
0.10
0.15
before
after
both

(i)

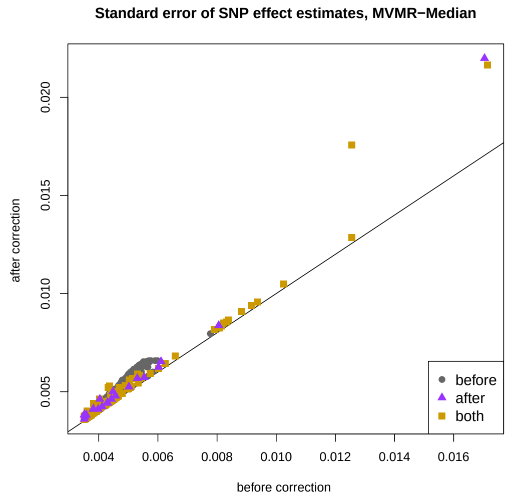
Standard error of SNP effect estimates, MVMR−Median
after correction
before correction
0.005
0.010
0.015
0.020
0.004
0.006
0.008
0.010
0.012
0.014
0.016
before
after
both

(j)

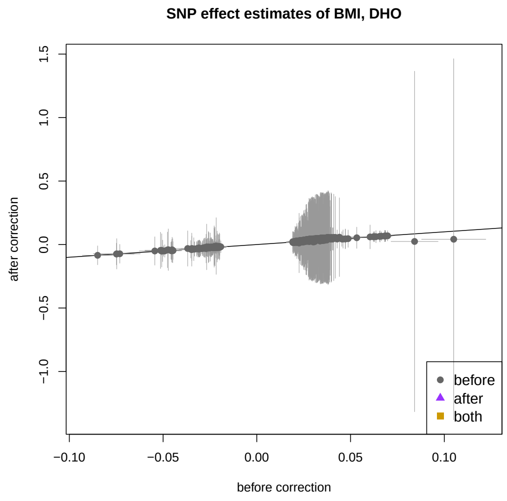
SNP effect estimates of BMI, DHO
after correction
before correction
−1.0
−0.5
0.0
0.5
1.0
1.5
−0.10
−0.05
0.00
0.05
0.10
before
after
both

(k)

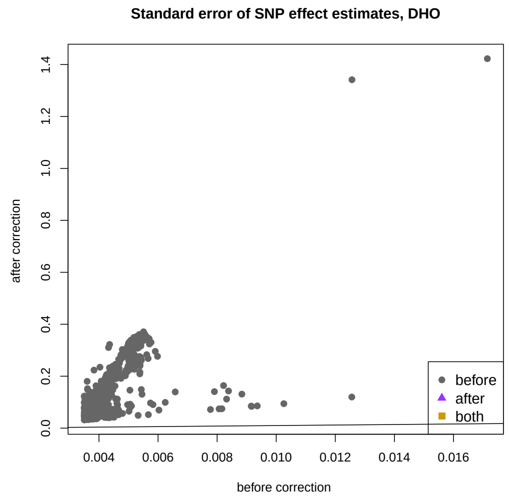
Standard error of SNP effect estimates, DHO
after correction
before correction
0.0
0.2
0.4
0.6
0.8
1.0
1.2
1.4
0.004
0.006
0.008
0.010
0.012
0.014
0.016
before
after
both

(l)

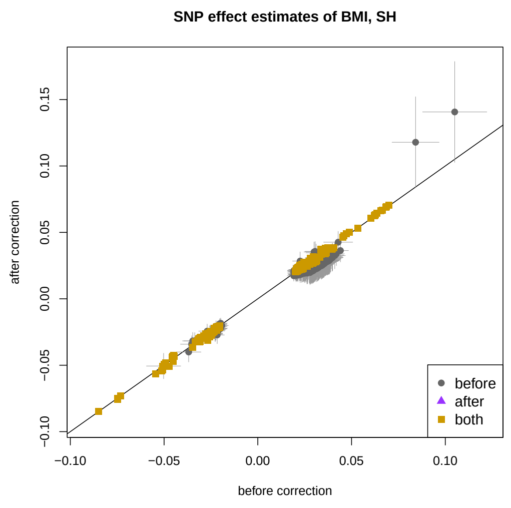

(m)

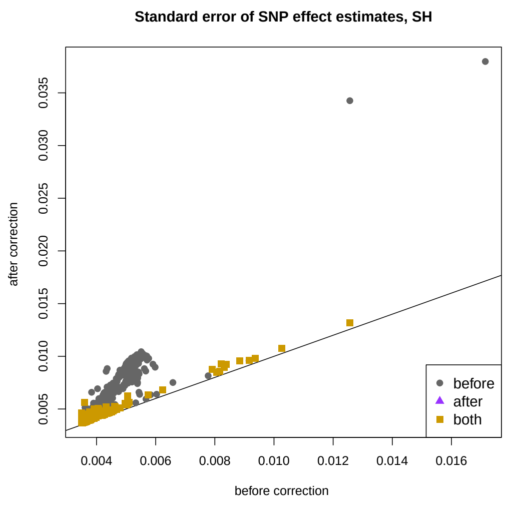

(n)

Fig AT. Effect estimates (in M_2) of BMI before and after bias correction. Horizontal and vertical bars represent 1 SE of an estimate before and after correction respectively. SEs are given in the right column. All 20 metabolomic PCs are used. 1 metabolomic PC is adjusted for bias correction. In the legends, "before" refers to the SNPs that are significant only before applying bias correction, "after" refers to the SNPs that are significant only after bias correction, "both" refers to the SNPs that are significant both before and after bias correction.

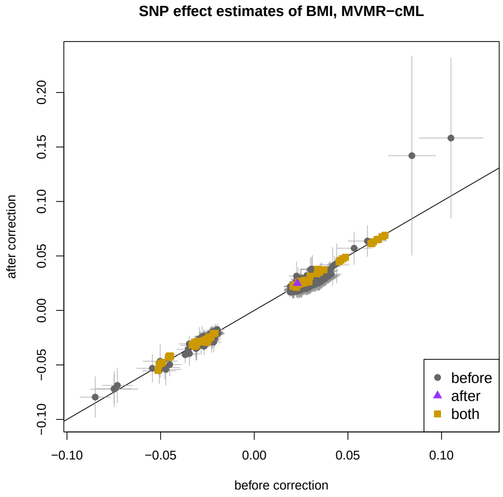
SNP effect estimates of BMI, MVMR−cML
after correction
before correction
−0.10 −0.05 0.00 0.05 0.10
−0.10 −0.05 0.00 0.05 0.10 0.15 0.20
before
after
both

(a)

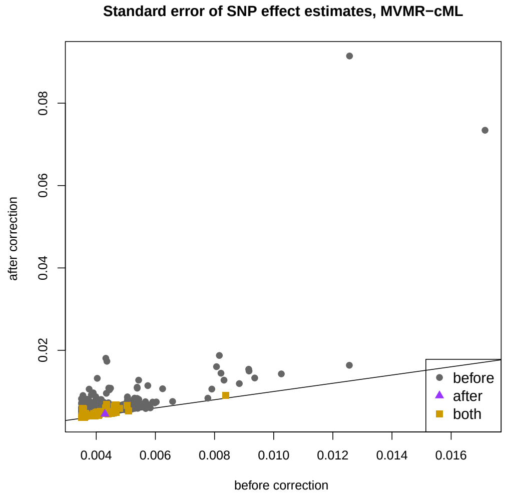
Standard error of SNP effect estimates, MVMR−cML
after correction
before correction
0.004 0.006 0.008 0.010 0.012 0.014 0.016
0.02 0.04 0.06 0.08
before
after
both

(b)

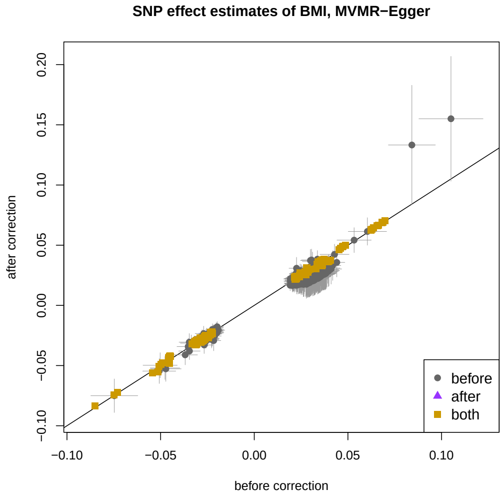
SNP effect estimates of BMI, MVMR−Egger
after correction
before correction
−0.10 −0.05 0.00 0.05 0.10
−0.10 −0.05 0.00 0.05 0.10 0.15 0.20
before
after
both

(c)

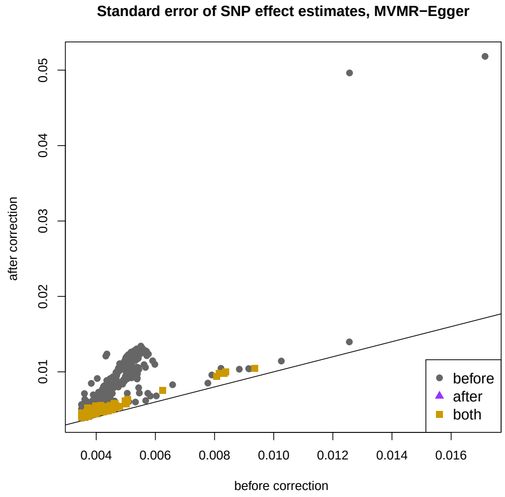
Standard error of SNP effect estimates, MVMR−Egger
after correction
before correction
0.004 0.006 0.008 0.010 0.012 0.014 0.016
0.01 0.02 0.03 0.04 0.05
before
after
both

(d)

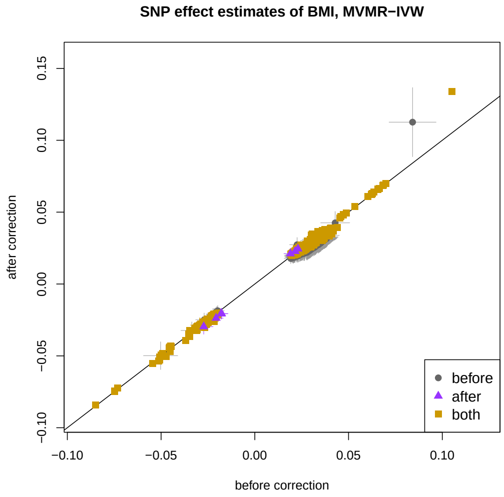
SNP effect estimates of BMI, MVMR−IVW
after correction
before correction
−0.10 −0.05 0.00 0.05 0.10
−0.10 −0.05 0.00 0.05 0.10 0.15
before
after
both

(e)

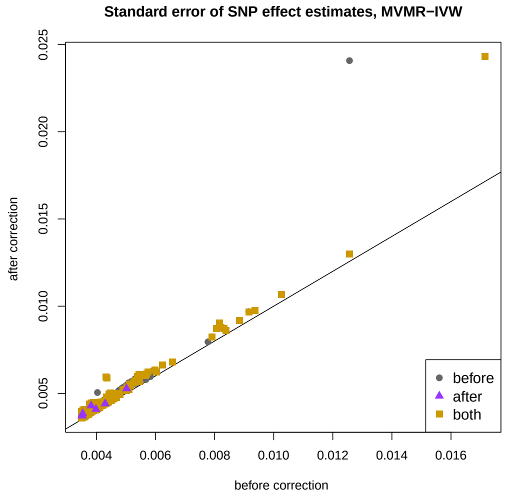
Standard error of SNP effect estimates, MVMR−IVW
after correction
before correction
0.004 0.006 0.008 0.010 0.012 0.014 0.016
0.005 0.010 0.015 0.020 0.025
before
after
both

(f)

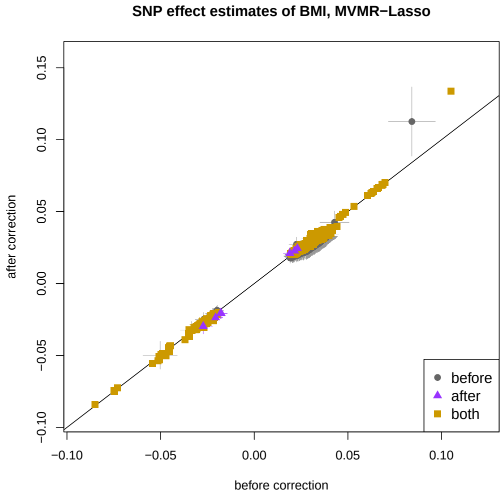

(g)

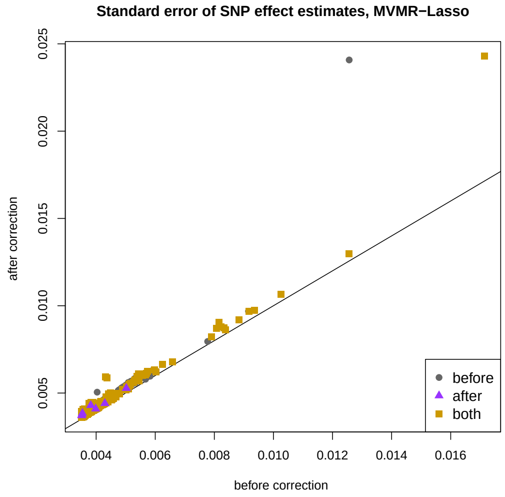

(h)

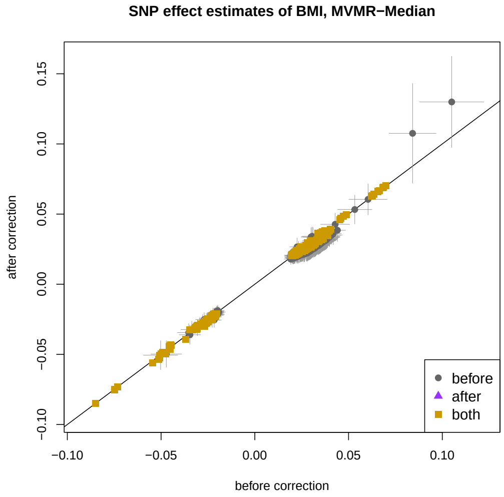

(i)

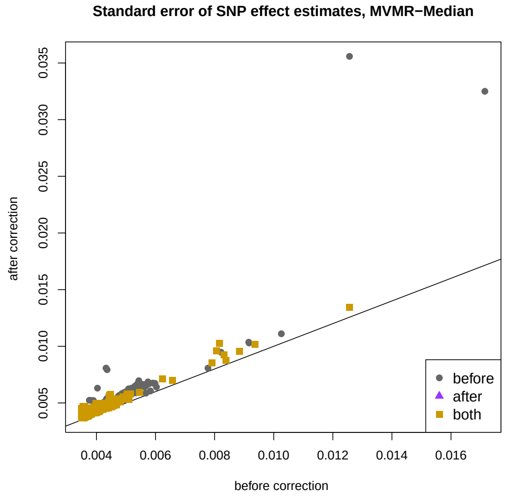

(j)

Fig AU. Effect estimates (in M_2) of BMI before and after bias correction. Horizontal and vertical bars represent 1 SE of an estimate before and after correction respectively. SEs are given in the right column. All 20 metabolomic PCs are used. 2 metabolomic PCs are adjusted for bias correction. In the legends, "before" refers to the SNPs that are significant only before applying bias correction, "after" refers to the SNPs that are significant only after bias correction, "both" refers to the SNPs that are significant both before and after bias correction.

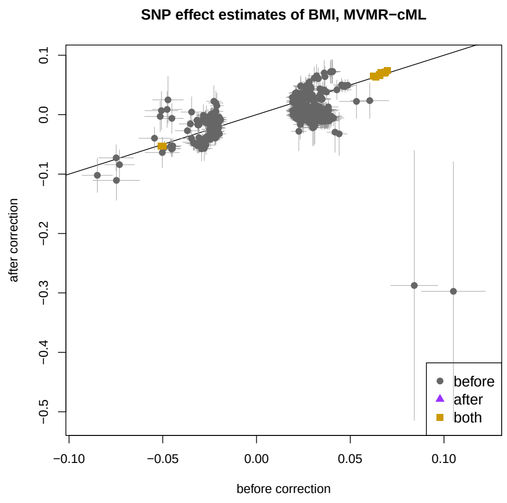
SNP effect estimates of BMI, MVMR−cML
after correction
before correction
before
after
both

(a)

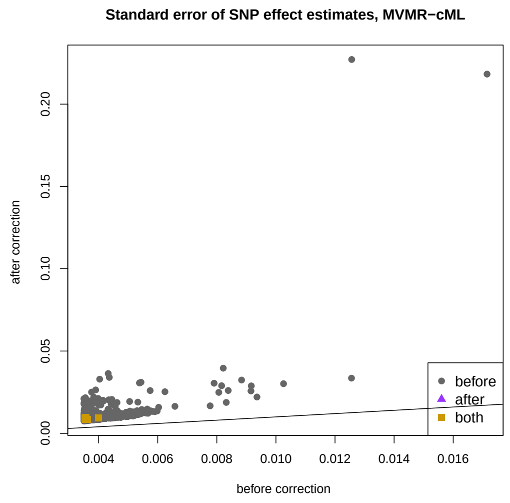
Standard error of SNP effect estimates, MVMR−cML
after correction
before correction
before
after
both

(b)

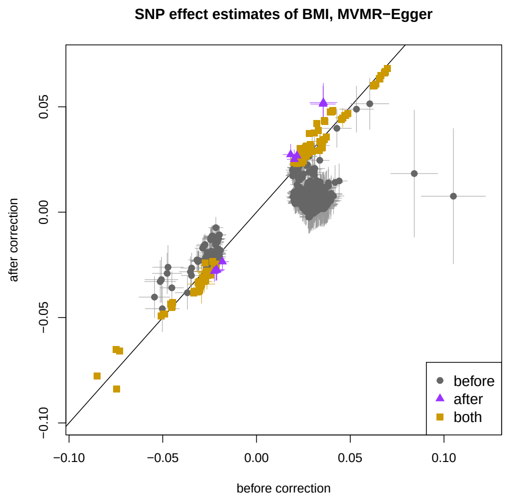
SNP effect estimates of BMI, MVMR−Egger
after correction
before correction
before
after
both

(c)

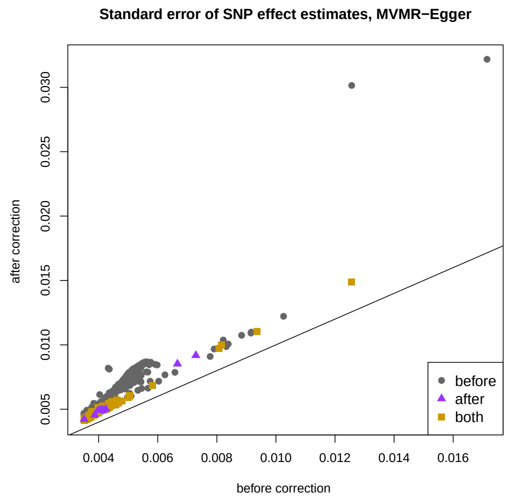
Standard error of SNP effect estimates, MVMR−Egger
after correction
before correction
before
after
both

(d)

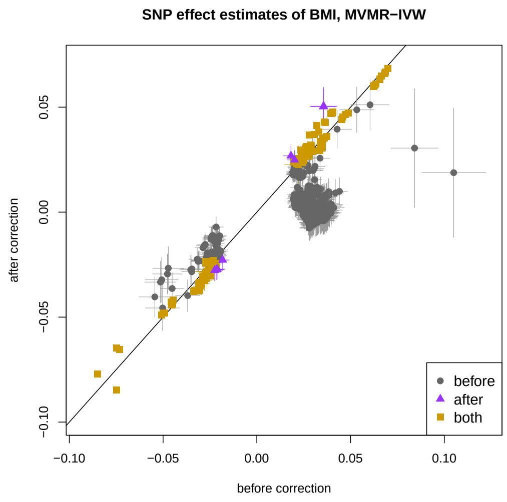
SNP effect estimates of BMI, MVMR−IVW
after correction
before correction
before
after
both

(e)

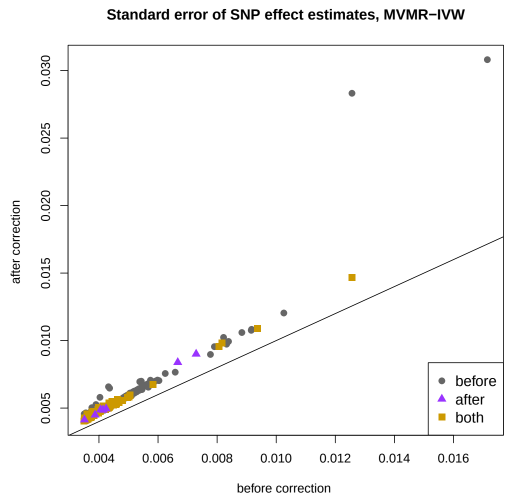
Standard error of SNP effect estimates, MVMR−IVW
after correction
before correction
before
after
both

(f)

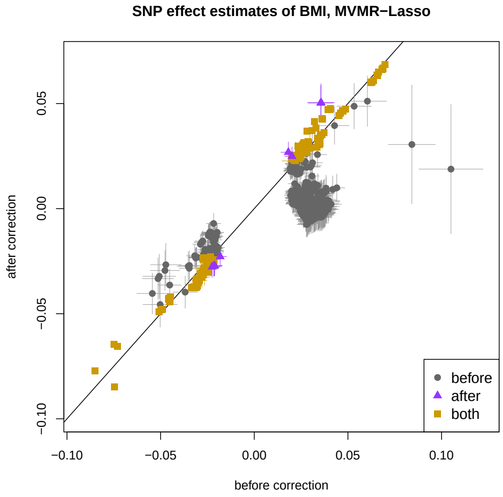

(g)

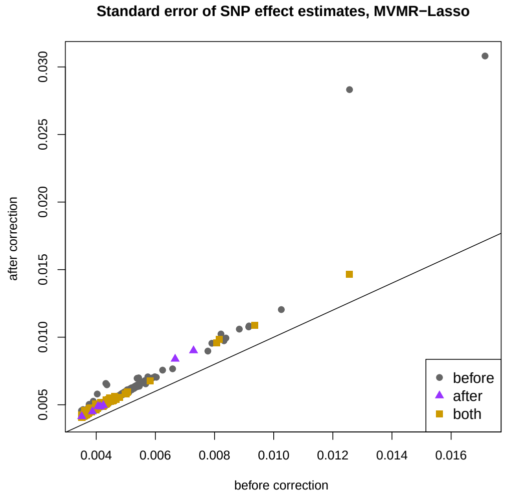

(h)

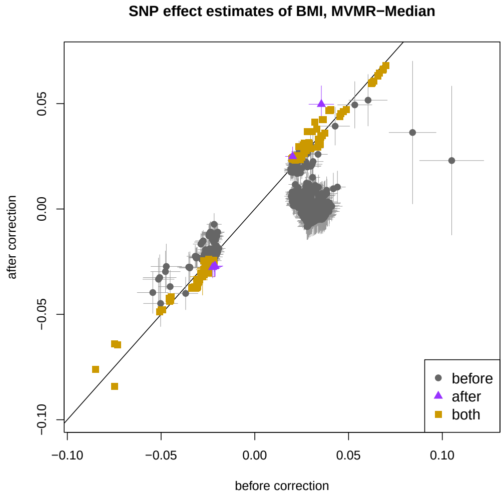

(i)

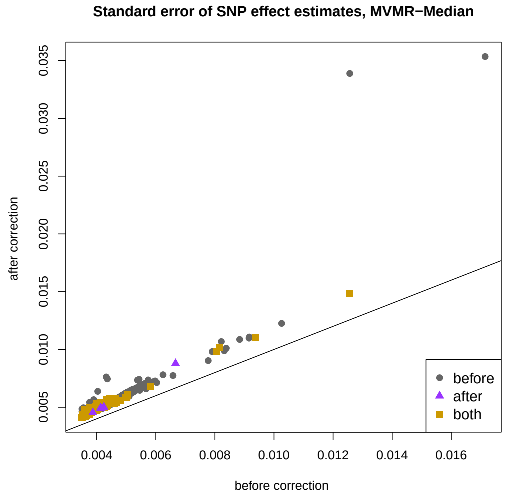

(j)

Fig AV. Effect estimates (in M_2) of BMI before and after bias correction. Horizontal and vertical bars represent 1 SE of an estimate before and after correction respectively. SEs are given in the right column. All 20 metabolomic PCs are used. 3 metabolomic PCs are adjusted for bias correction. In the legends, "before" refers to the SNPs that are significant only before applying bias correction, "after" refers to the SNPs that are significant only after bias correction, "both" refers to the SNPs that are significant both before and after bias correction.

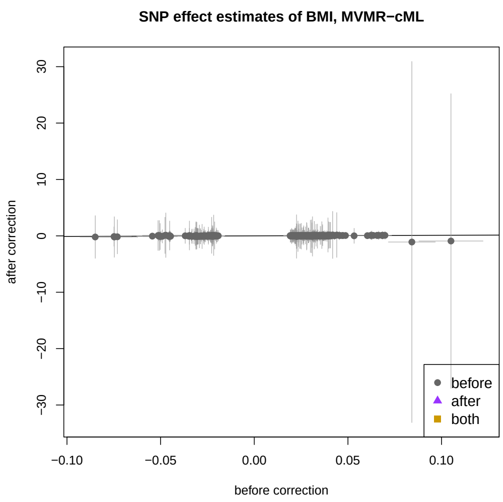
SNP effect estimates of BMI, MVMR−cML
after correction
before correction
before
after
both

(a)

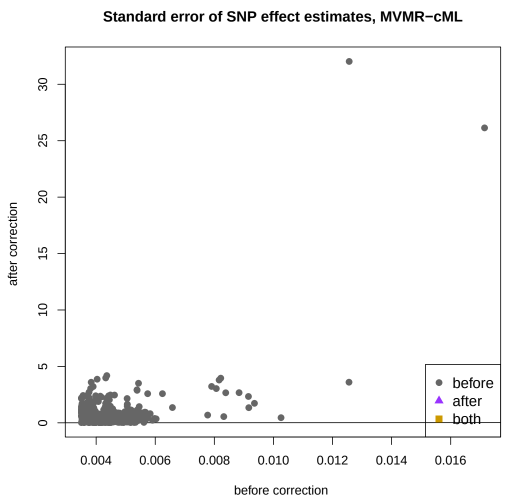
Standard error of SNP effect estimates, MVMR−cML
after correction
before correction
before
after
both

(b)

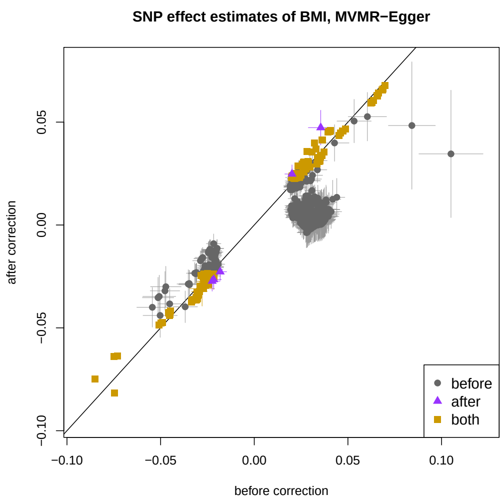
SNP effect estimates of BMI, MVMR−Egger
after correction
before correction
before
after
both

(c)

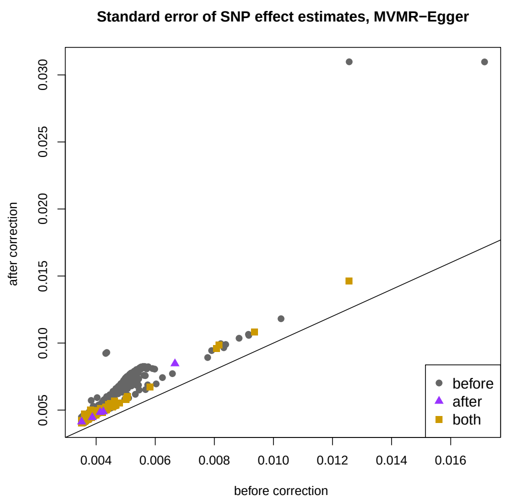
Standard error of SNP effect estimates, MVMR−Egger
after correction
before correction
before
after
both

(d)

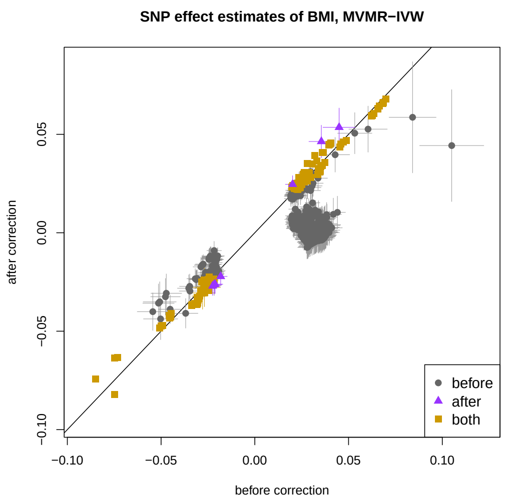
SNP effect estimates of BMI, MVMR−IVW
after correction
before correction
before
after
both

(e)

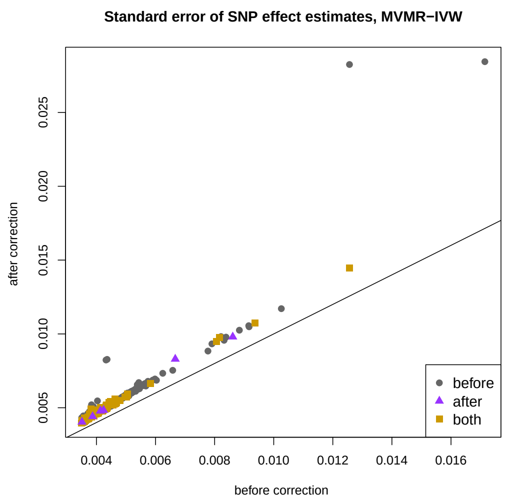
Standard error of SNP effect estimates, MVMR−IVW
after correction
before correction
before
after
both

(f)

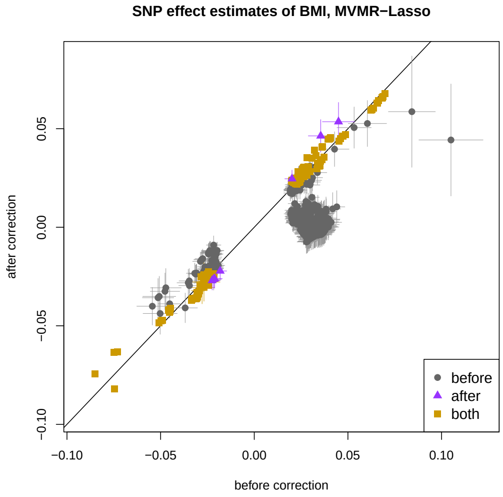

(g)

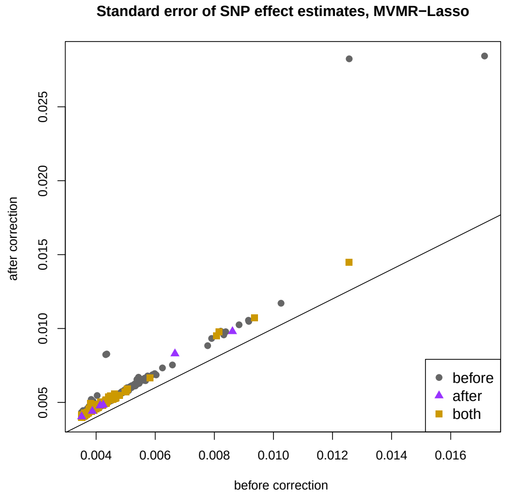

(h)

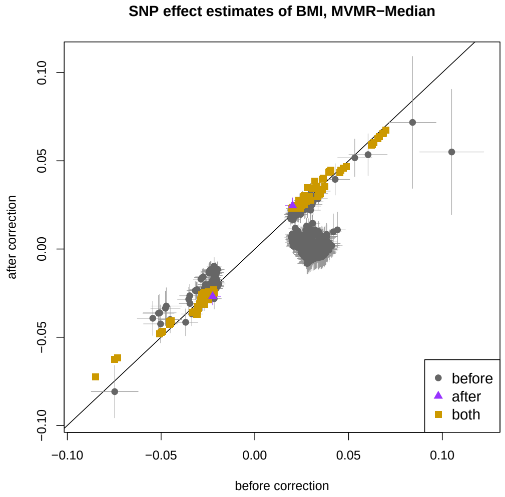

(i)

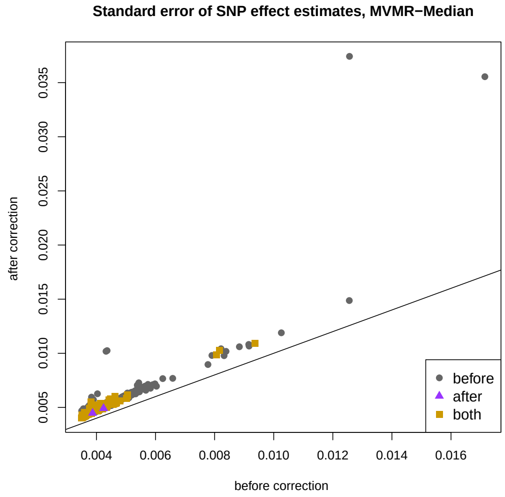

(j)

Fig AW. Effect estimates (in M_2) of BMI before and after bias correction. Horizontal and vertical bars represent 1 SE of an estimate before and after correction respectively. SEs are given in the right column. All 20 metabolomic PCs are used. 4 metabolomic PCs are adjusted for bias correction. In the legends, “before” refers to the SNPs that are significant only before applying bias correction, “after” refers to the SNPs that are significant only after bias correction, “both” refers to the SNPs that are significant both before and after bias correction.

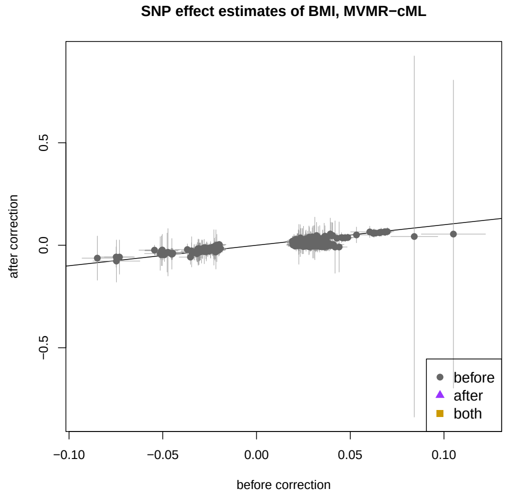
SNP effect estimates of BMI, MVMR−cML
after correction
before correction
−0.5
0.0
0.5
−0.10
−0.05
0.00
0.05
0.10
before
after
both

(a)

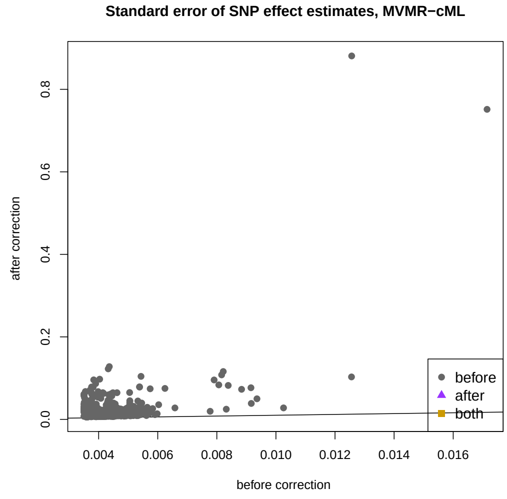
Standard error of SNP effect estimates, MVMR−cML
after correction
before correction
0.0
0.2
0.4
0.6
0.8
0.004
0.006
0.008
0.010
0.012
0.014
0.016
before
after
both

(b)

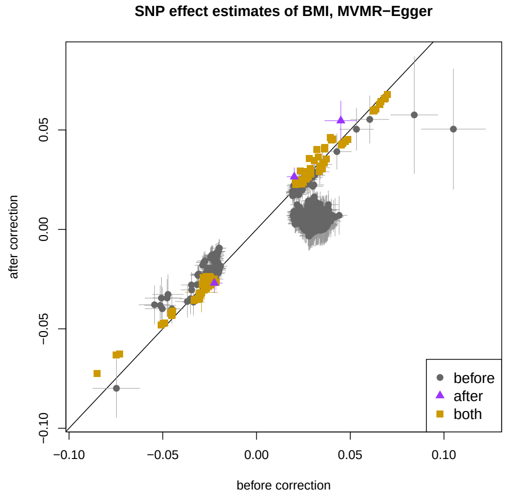
SNP effect estimates of BMI, MVMR−Egger
after correction
before correction
−0.10
−0.05
0.00
0.05
−0.10
−0.05
0.00
0.05
0.10
before
after
both

(c)

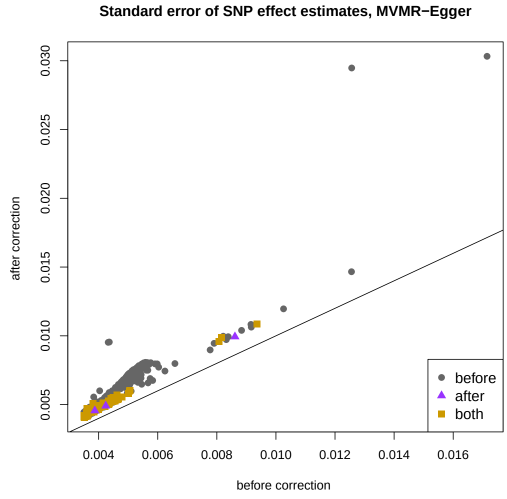
Standard error of SNP effect estimates, MVMR−Egger
after correction
before correction
0.005
0.010
0.015
0.020
0.025
0.030
0.004
0.006
0.008
0.010
0.012
0.014
0.016
before
after
both

(d)

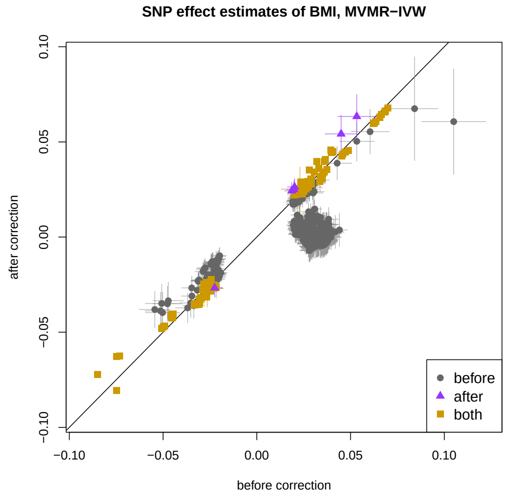
SNP effect estimates of BMI, MVMR−IVW
after correction
before correction
−0.10
−0.05
0.00
0.05
0.10
−0.10
−0.05
0.00
0.05
0.10
before
after
both

(e)

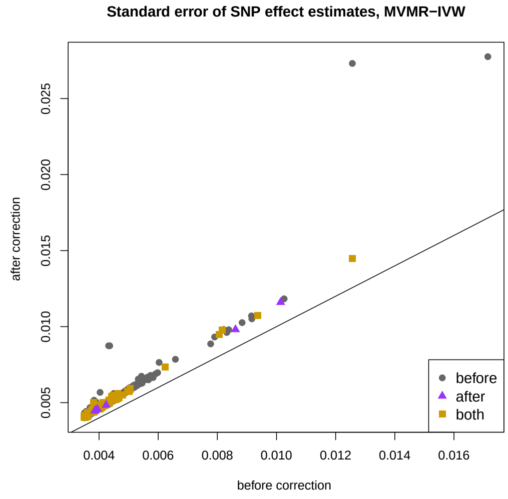
Standard error of SNP effect estimates, MVMR−IVW
after correction
before correction
0.005
0.010
0.015
0.020
0.025
0.004
0.006
0.008
0.010
0.012
0.014
0.016
before
after
both

(f)

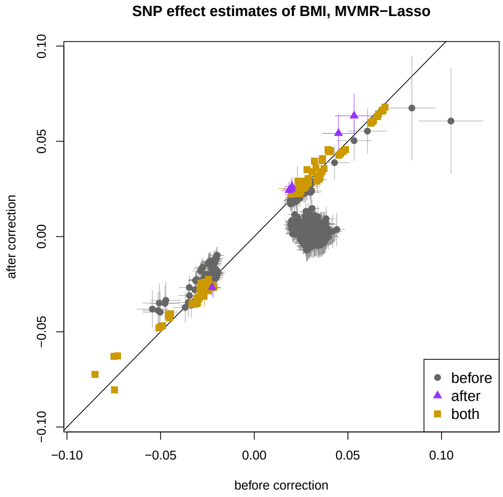

(g)

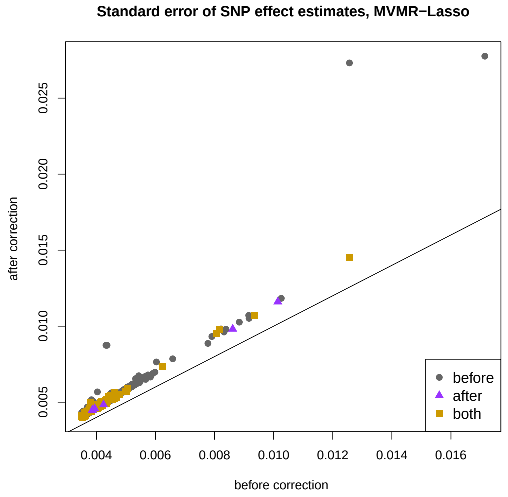

(h)

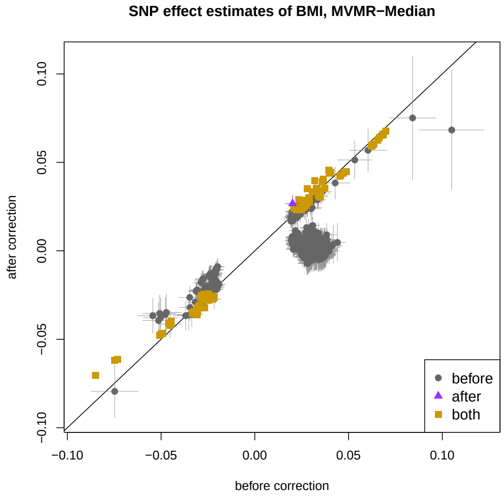

(i)

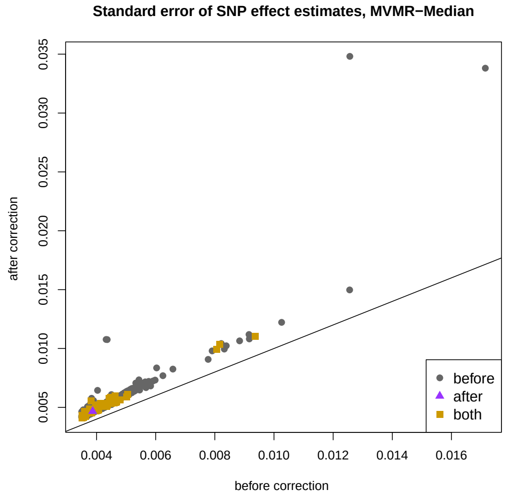

(j)

Fig AX. Effect estimates (in M_2) of BMI before and after bias correction. Horizontal and vertical bars represent 1 SE of an estimate before and after correction respectively. SEs are given in the right column. All 20 metabolomic PCs are used. 5 metabolomic PCs are adjusted for bias correction. In the legends, "before" refers to the SNPs that are significant only before applying bias correction, "after" refers to the SNPs that are significant only after bias correction, "both" refers to the SNPs that are significant both before and after bias correction.

G.2.7 Comparison of Manhattan plots before and after apply different bias-correction methods on M_1

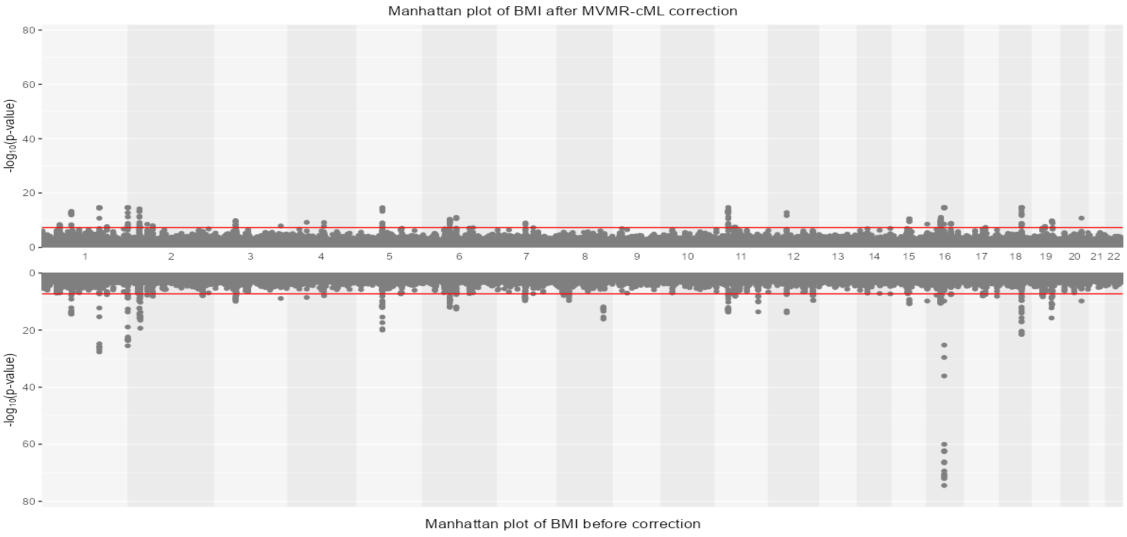

(a)

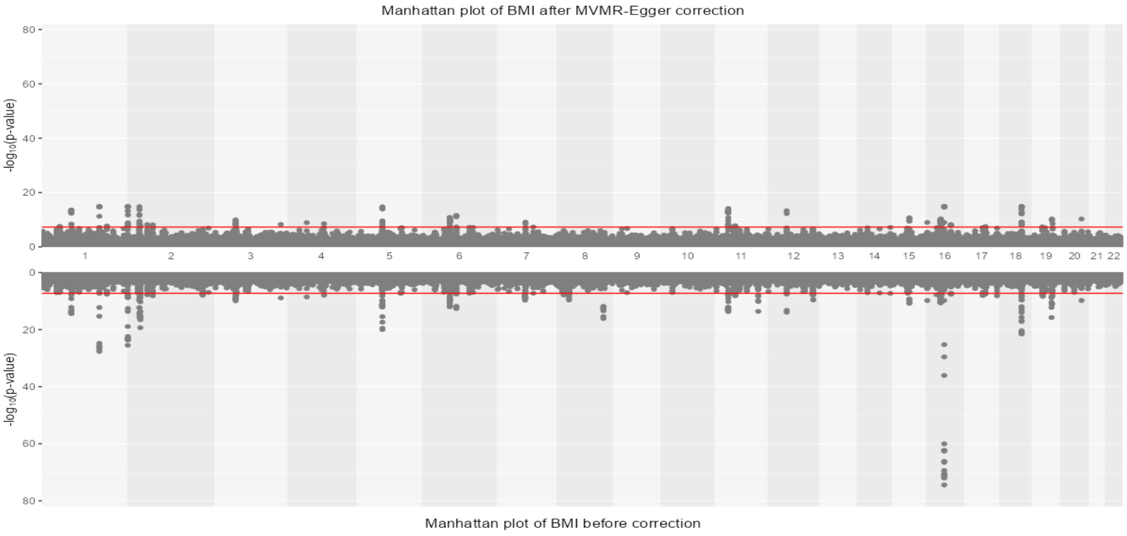

(b)

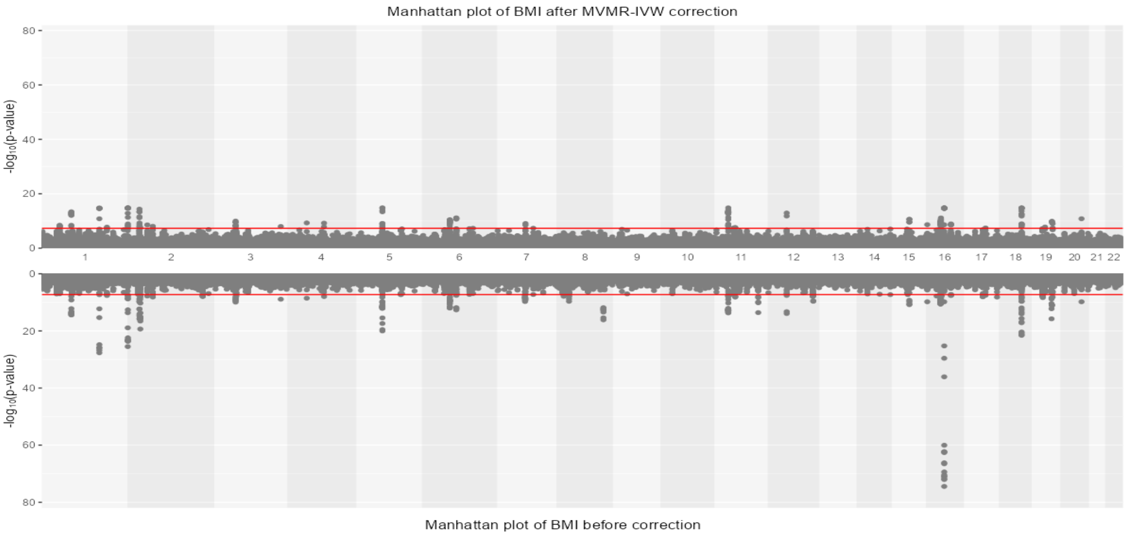

(c)

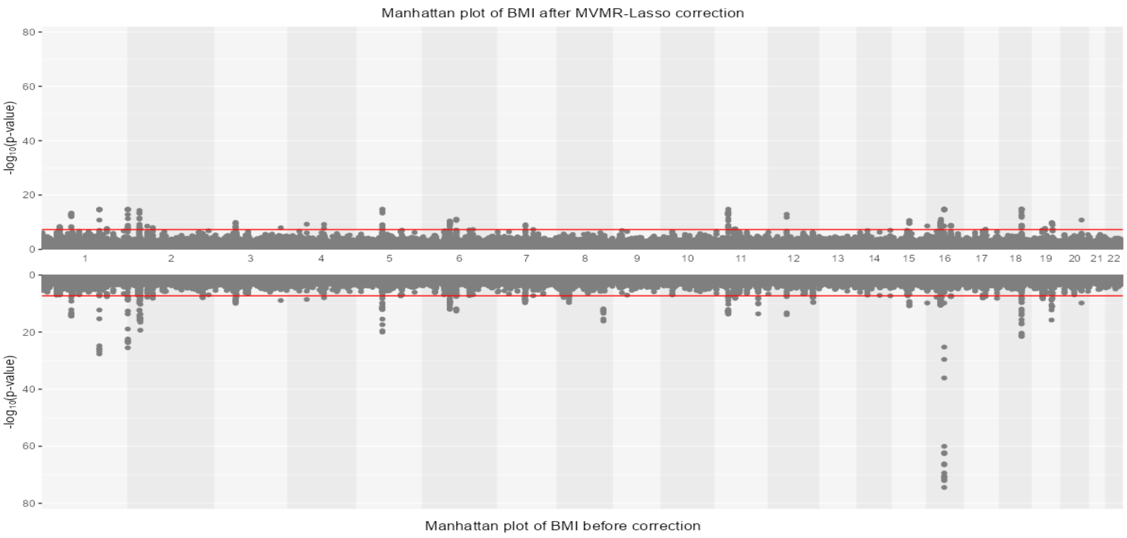
Manhattan plot of BMI after MVMR-Lasso correction
-log10(p-value)
Manhattan plot of BMI before correction

(d)

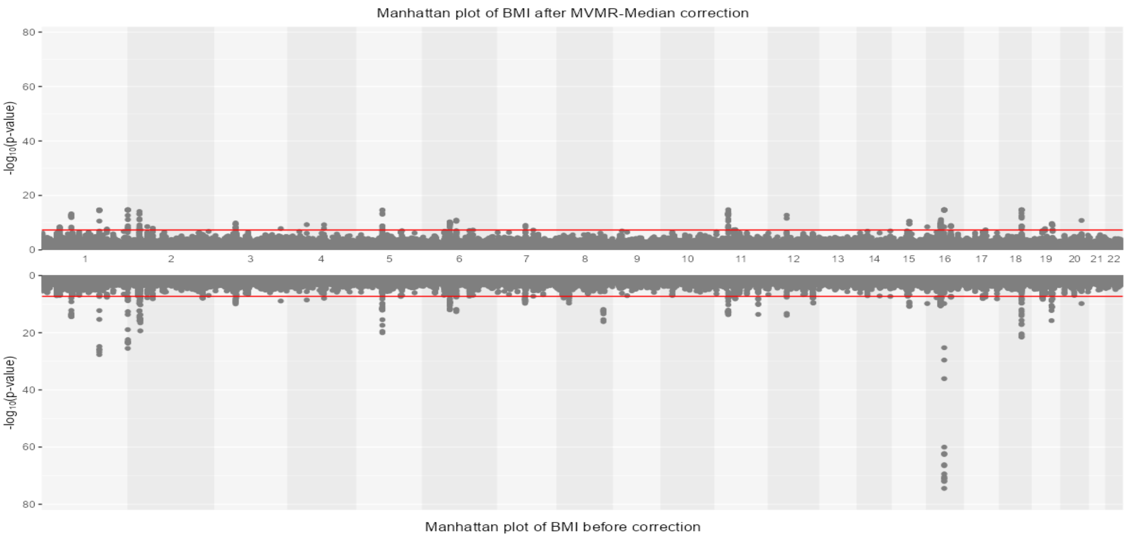
Manhattan plot of BMI after MVMR-Median correction
-log10(p-value)
Manhattan plot of BMI before correction

(e)

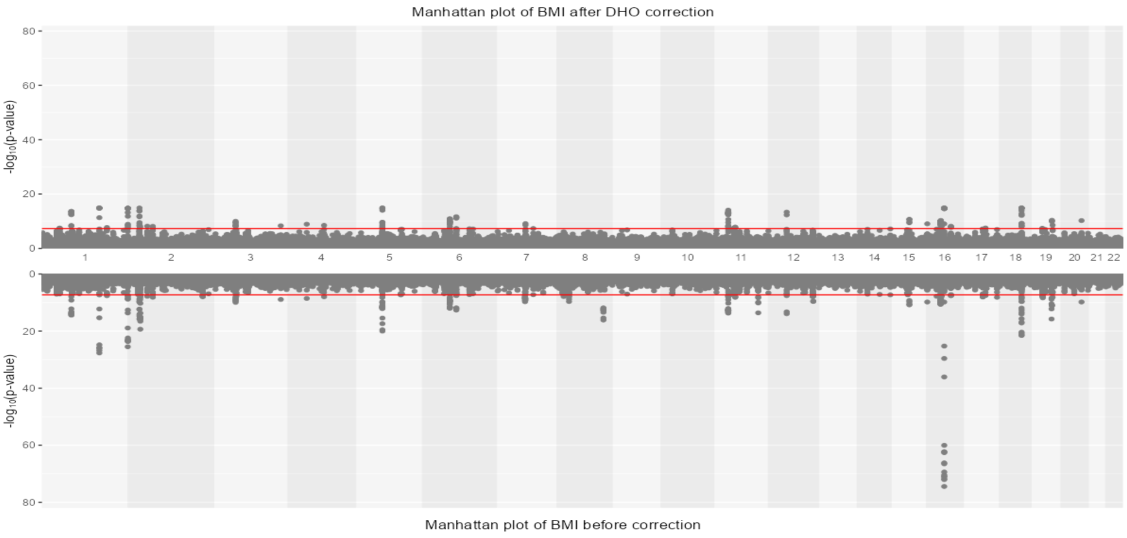
Manhattan plot of BMI after DHO correction
-log10(p-value)
Manhattan plot of BMI before correction

(f)

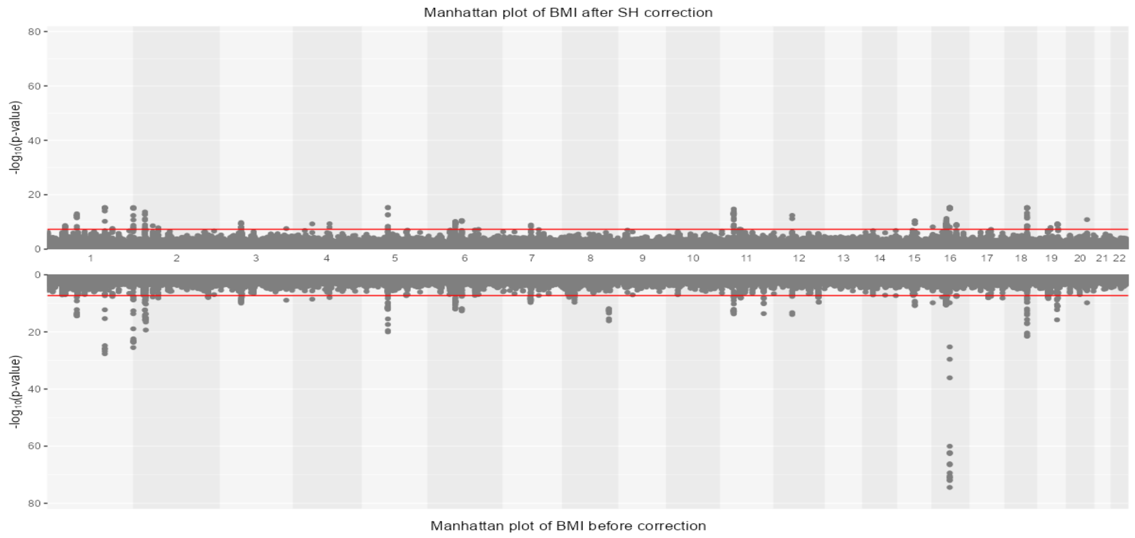

(g)

Fig AY. Manhattan plot of BMI before (upper panel) and after (lower panel) applying bias correction (in M_1). 1 metabolomic PC is adjusted.

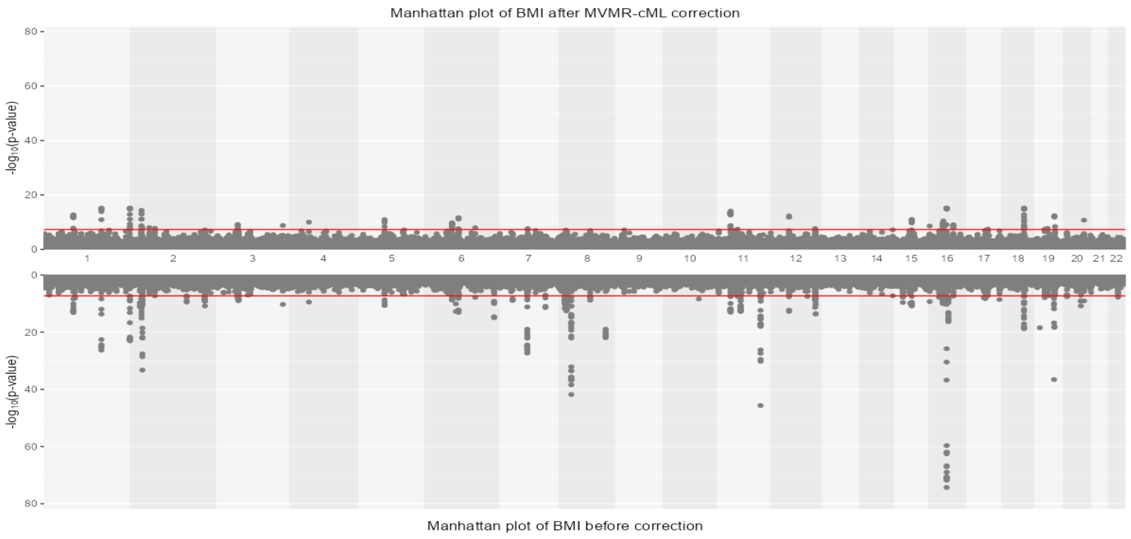
Manhattan plot of BMI after MVMR-cML correction
-log10(p-value)
Manhattan plot of BMI before correction

(a)

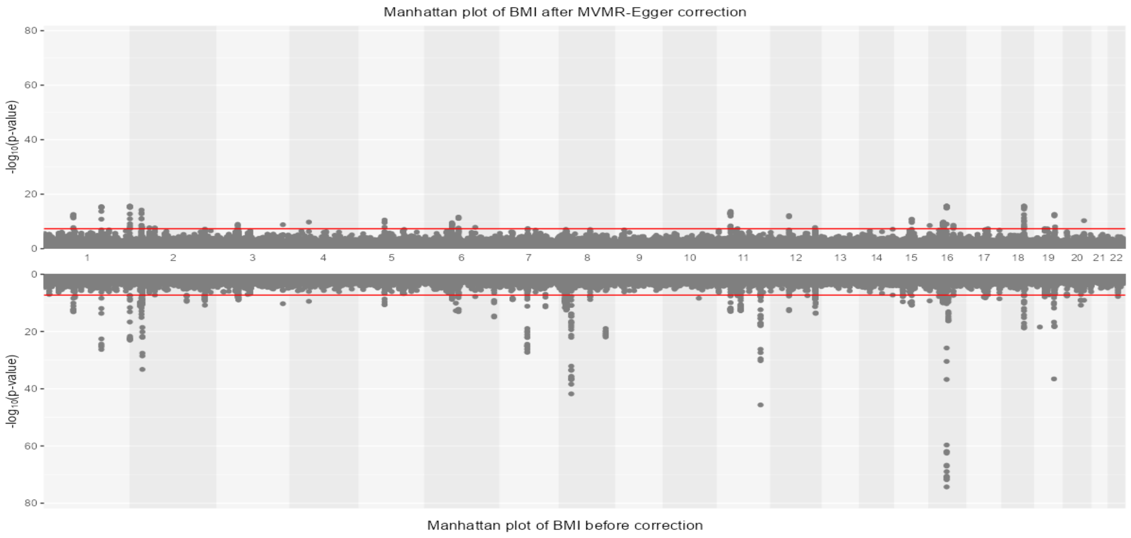
Manhattan plot of BMI after MVMR-Egger correction
-log10(p-value)
Manhattan plot of BMI before correction

(b)

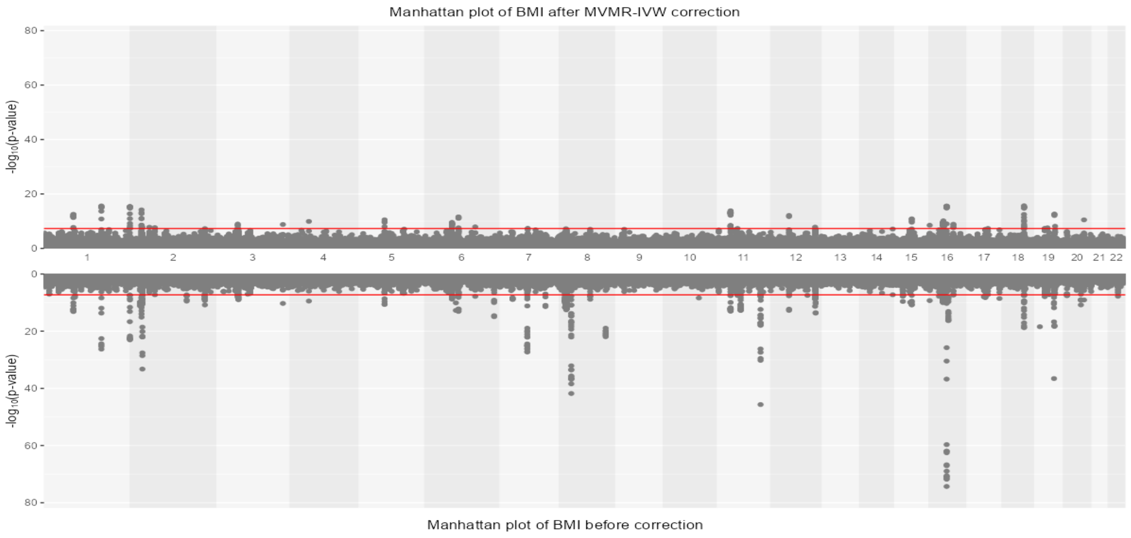
Manhattan plot of BMI after MVMR-IVW correction
-log10(p-value)
Manhattan plot of BMI before correction

(c)

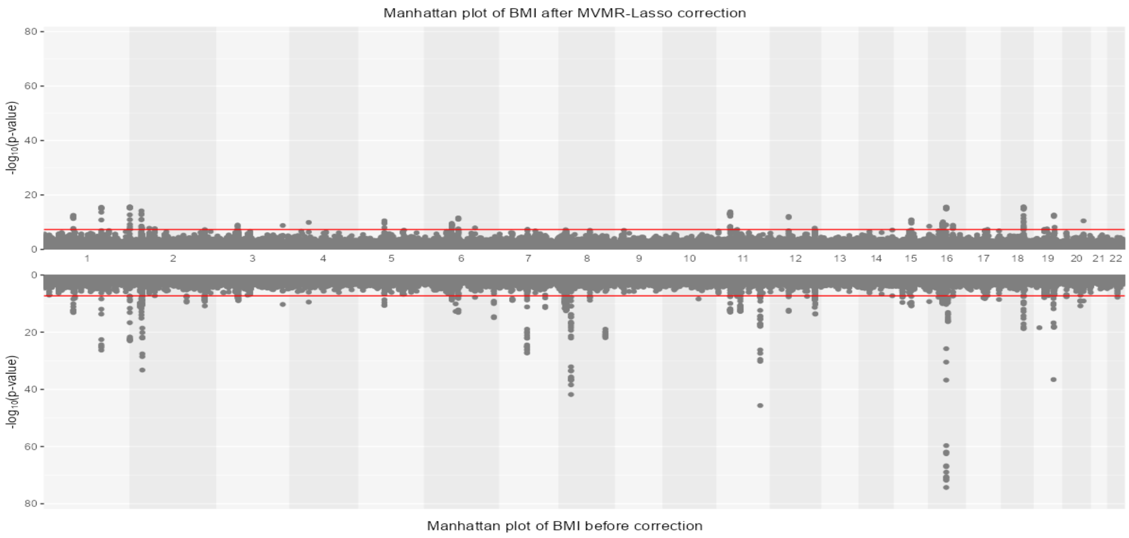

(d)

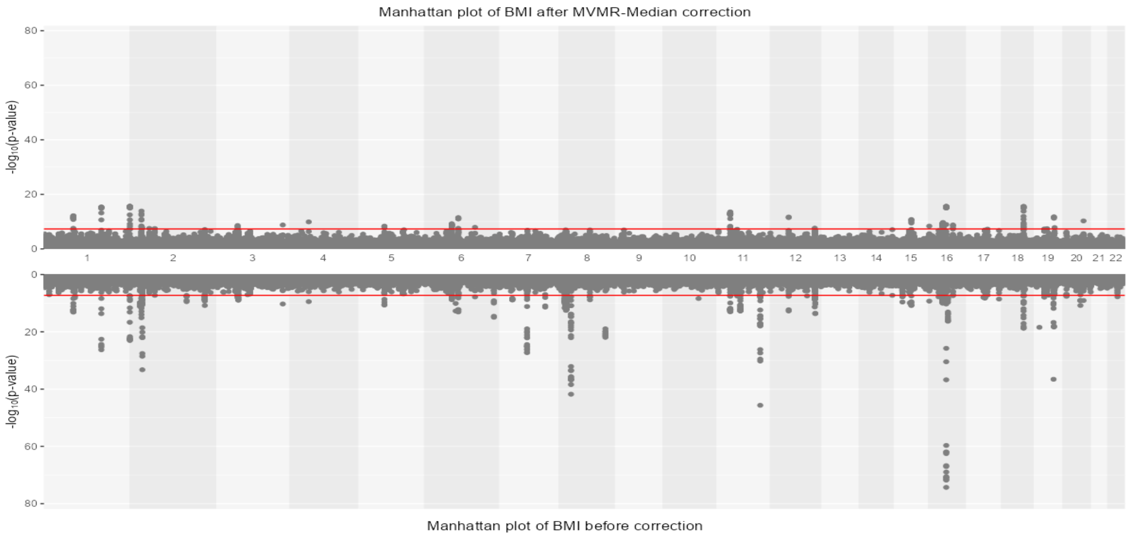

(e)

Fig AZ. Manhattan plot of BMI before (upper panel) and after (lower panel) applying bias correction (in M_1). 2 metabolomic PCs are adjusted.

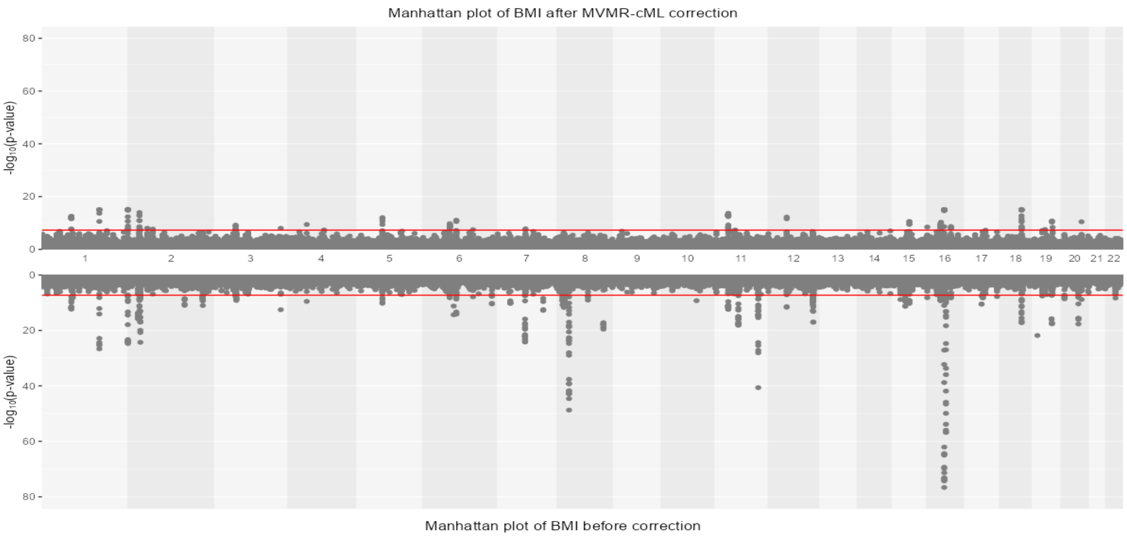
Manhattan plot of BMI after MVMR-cML correction
-log10(p-value)
0
20
40
60
80
1 2 3 4 5 6 7 8 9 10 11 12 13 14 15 16 17 18 19 20 21 22
Manhattan plot of BMI before correction

(a)

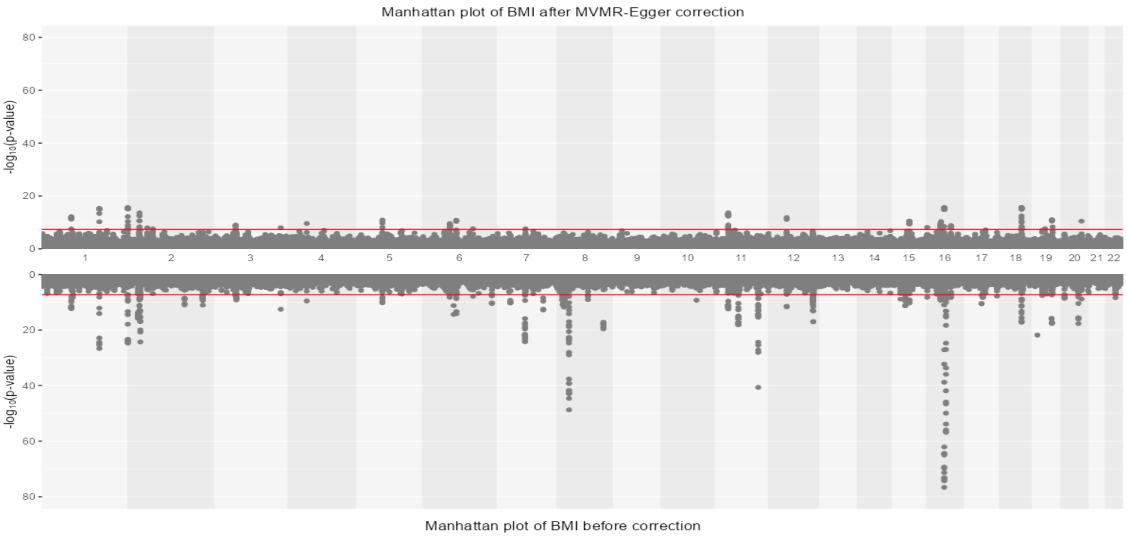
Manhattan plot of BMI after MVMR-Egger correction
-log10(p-value)
0
20
40
60
80
1 2 3 4 5 6 7 8 9 10 11 12 13 14 15 16 17 18 19 20 21 22
Manhattan plot of BMI before correction

(b)

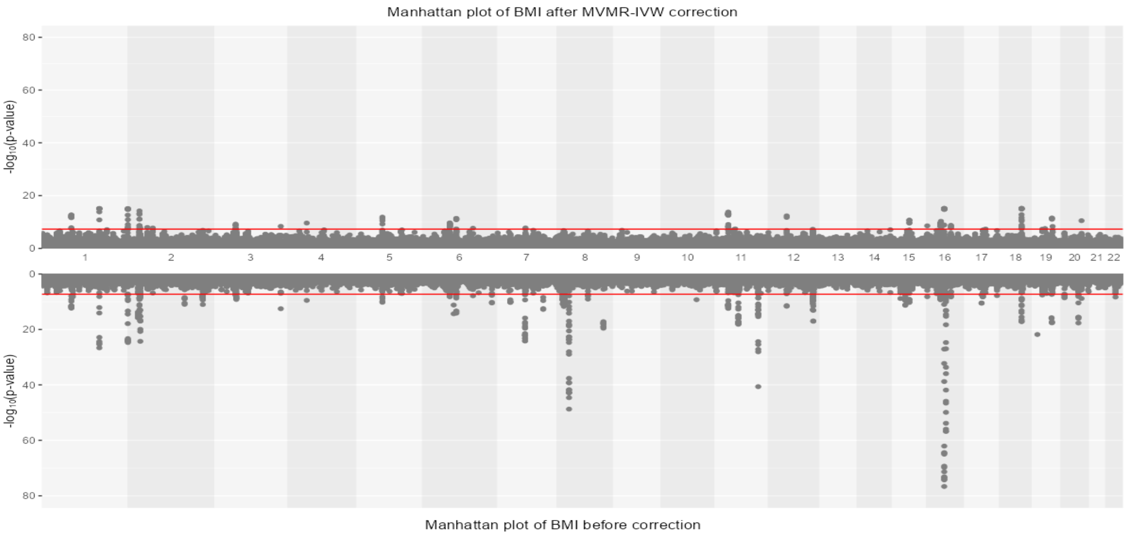
Manhattan plot of BMI after MVMR-IVW correction
-log10(p-value)
0
20
40
60
80
1 2 3 4 5 6 7 8 9 10 11 12 13 14 15 16 17 18 19 20 21 22
Manhattan plot of BMI before correction

(c)

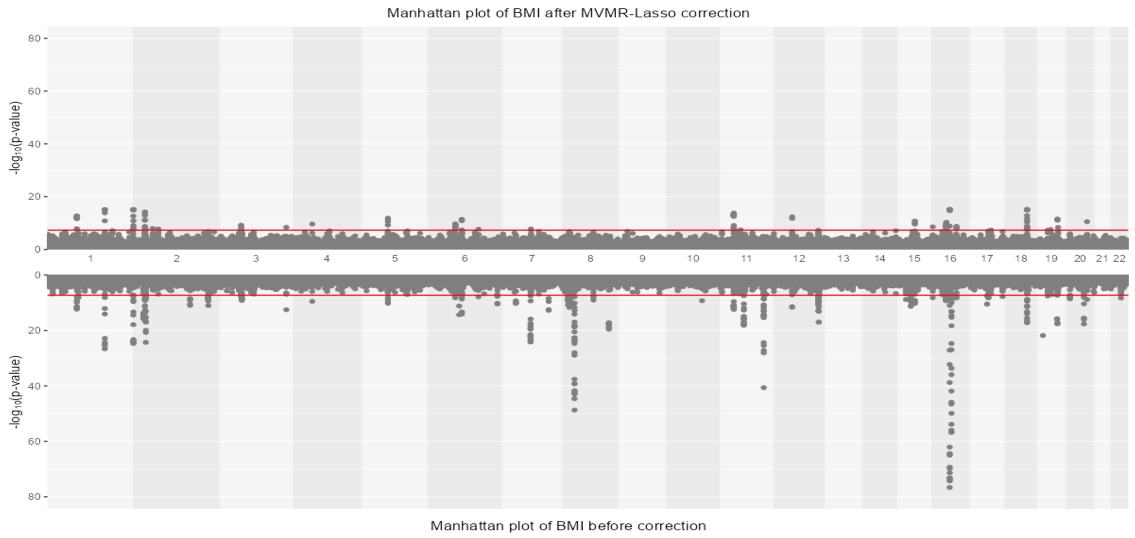

(d)

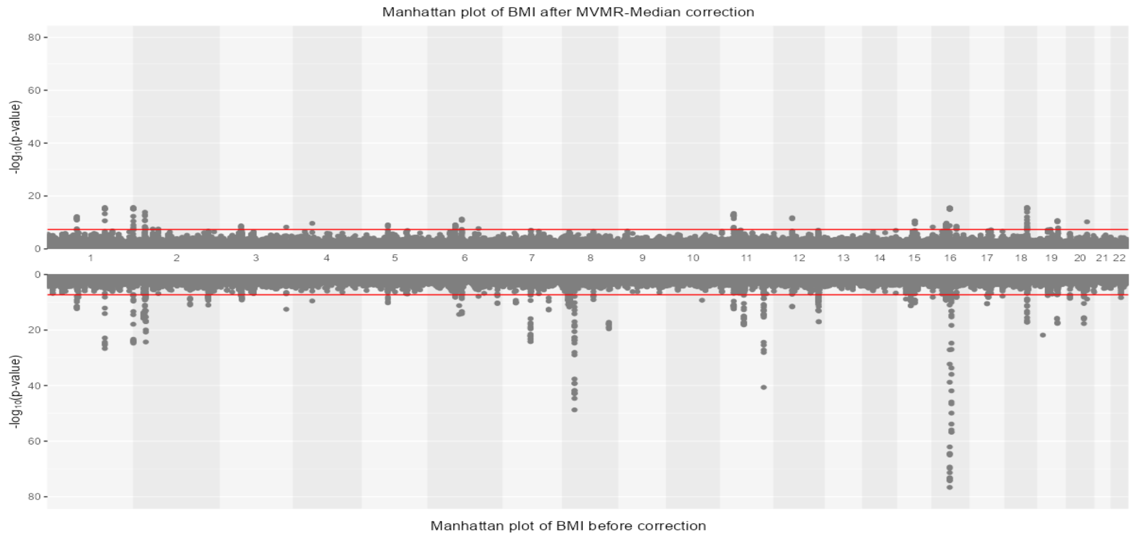

(e)

Fig BA. Manhattan plot of BMI before (upper panel) and after (lower panel) applying bias correction (in M_1). 3 metabolomic PCs are adjusted.

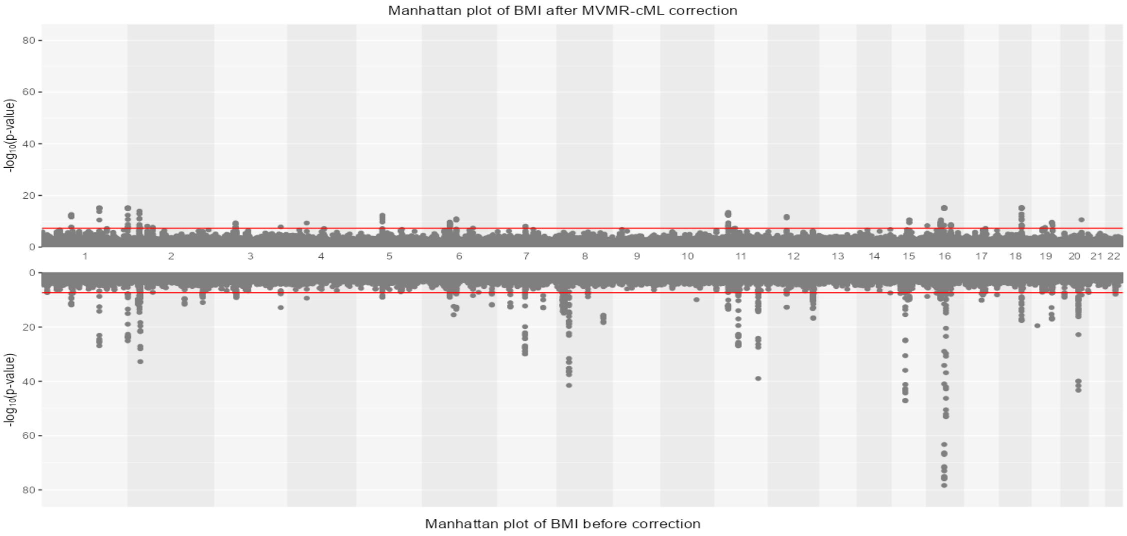
Manhattan plot of BMI after MVMR-cML correction
-log10(p-value)
1 2 3 4 5 6 7 8 9 10 11 12 13 14 15 16 17 18 19 20 21 22
Manhattan plot of BMI before correction

(a)

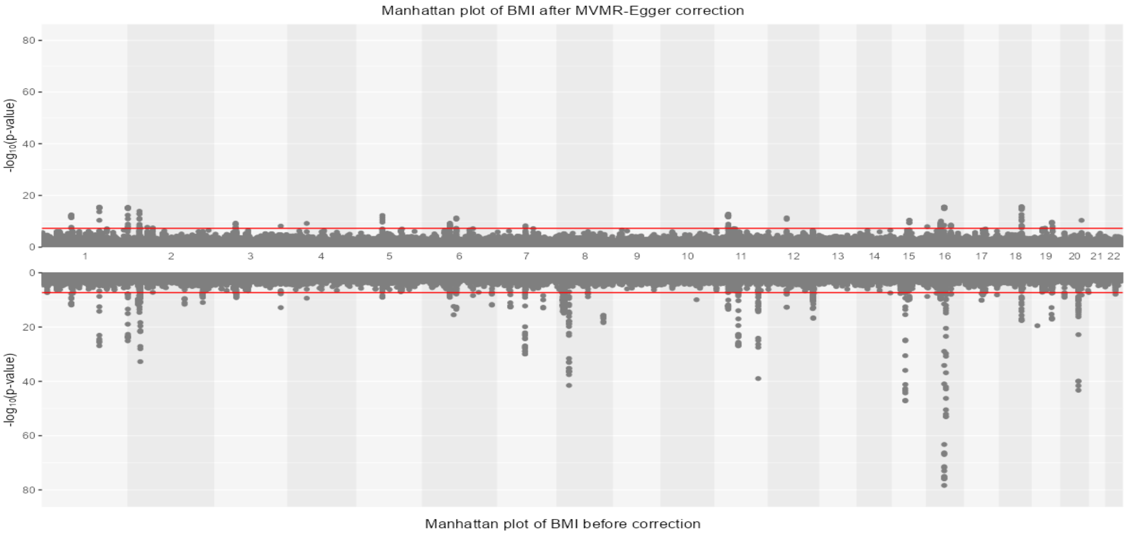
Manhattan plot of BMI after MVMR-Egger correction
-log10(p-value)
1 2 3 4 5 6 7 8 9 10 11 12 13 14 15 16 17 18 19 20 21 22
Manhattan plot of BMI before correction

(b)

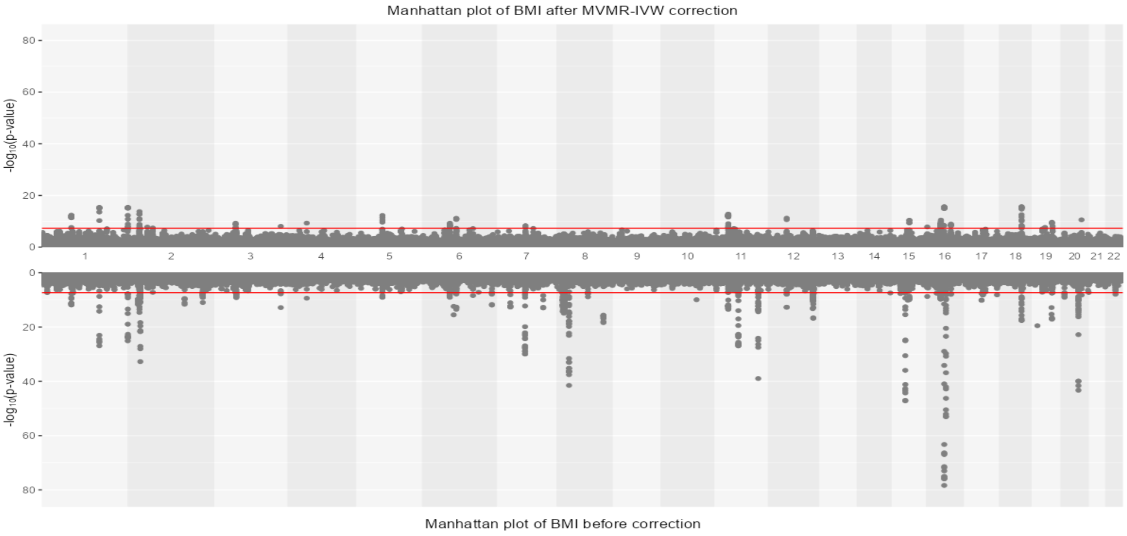
Manhattan plot of BMI after MVMR-IVW correction
-log10(p-value)
1 2 3 4 5 6 7 8 9 10 11 12 13 14 15 16 17 18 19 20 21 22
Manhattan plot of BMI before correction

(c)

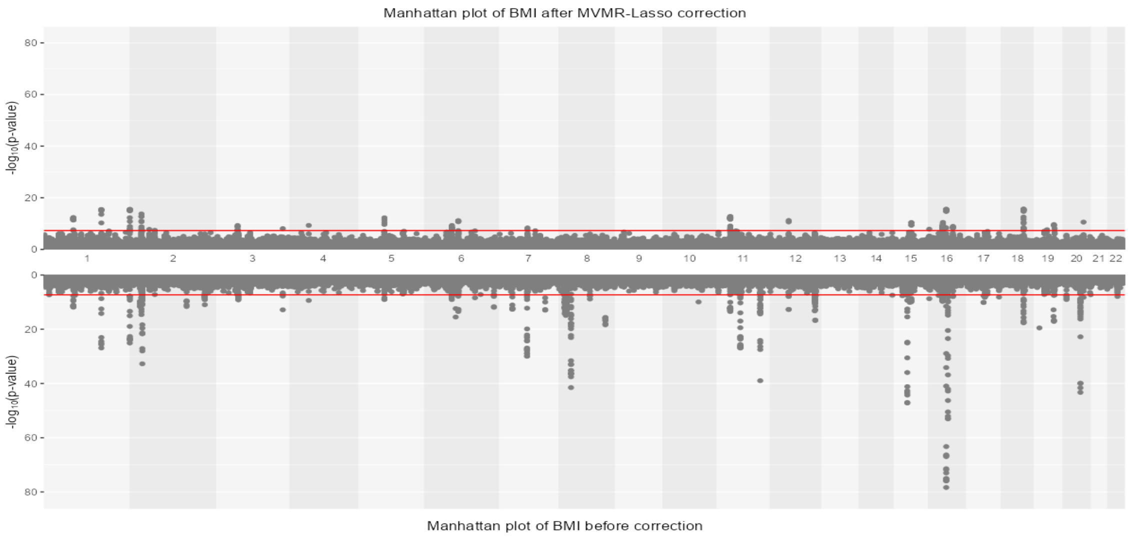

(d)

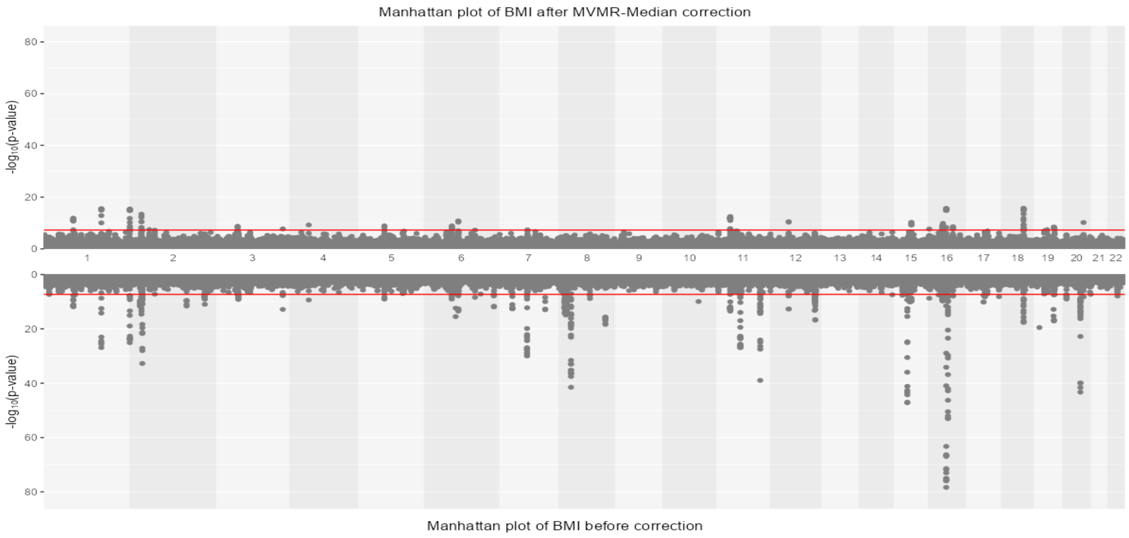

(e)

Fig BB. Manhattan plot of BMI before (upper panel) and after (lower panel) applying bias correction (in M_1). 4 metabolomic PCs are adjusted.

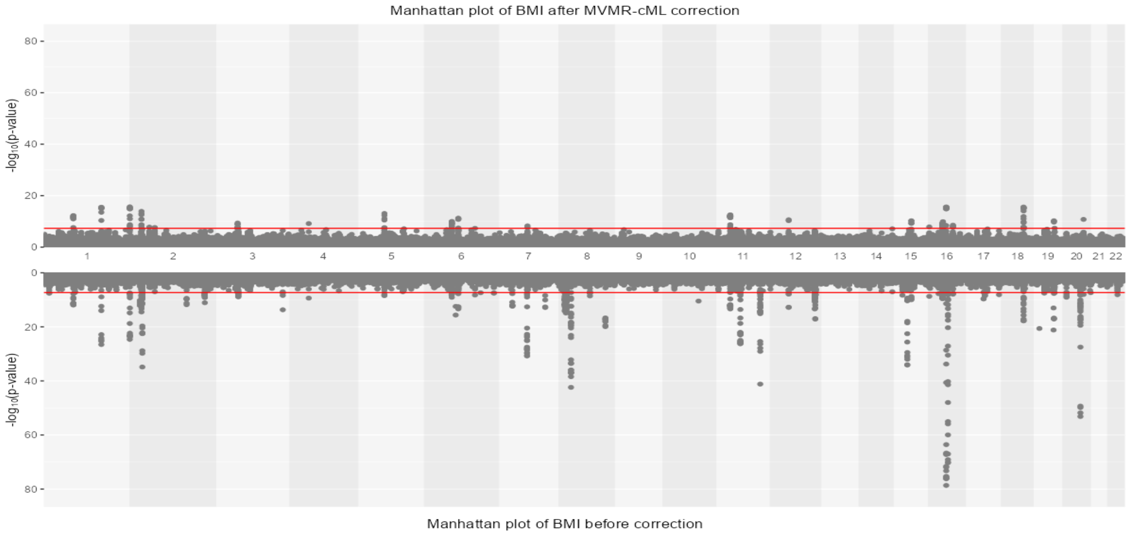
Manhattan plot of BMI after MVMR-cML correction
-log10(p-value)
Manhattan plot of BMI before correction

(a)

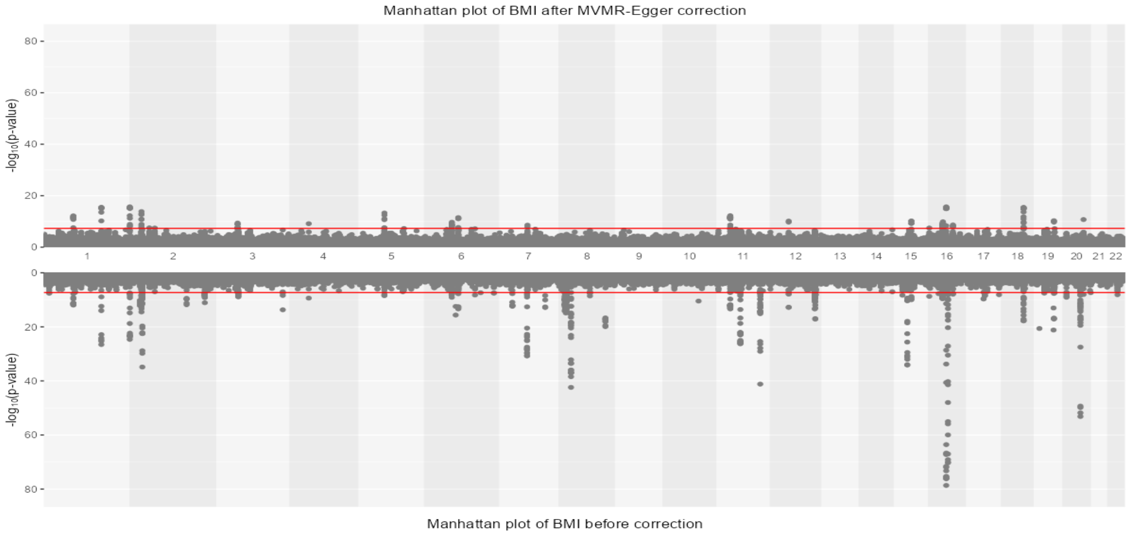
Manhattan plot of BMI after MVMR-Egger correction
-log10(p-value)
Manhattan plot of BMI before correction

(b)

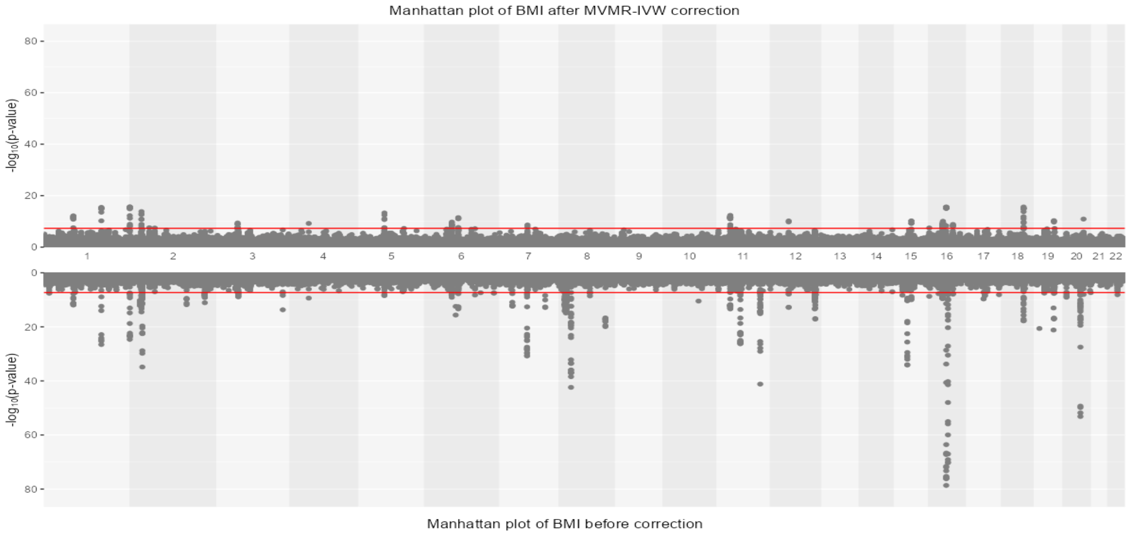
Manhattan plot of BMI after MVMR-IVW correction
-log10(p-value)
Manhattan plot of BMI before correction

(c)

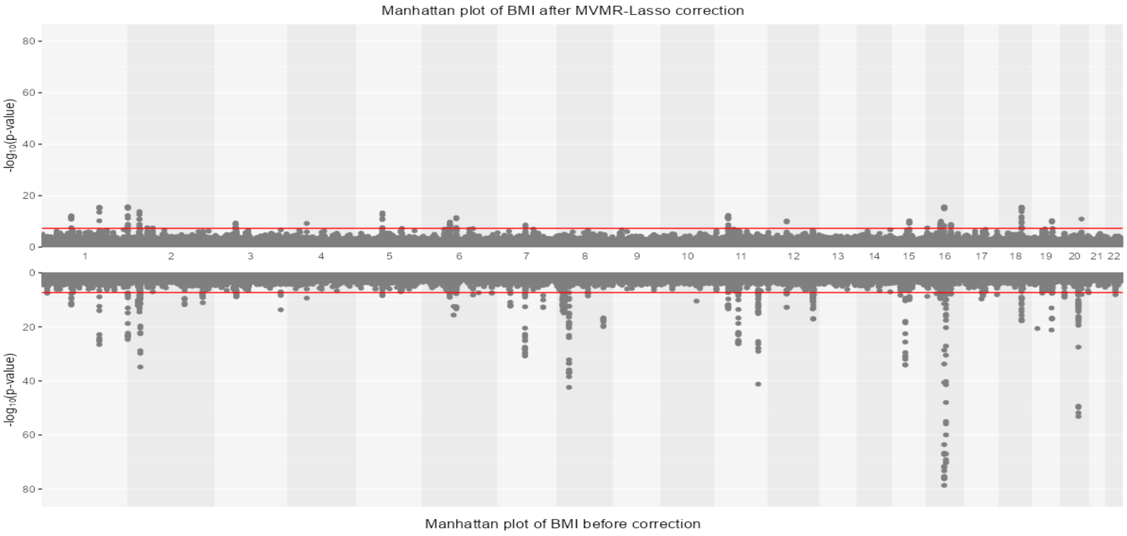

(d)

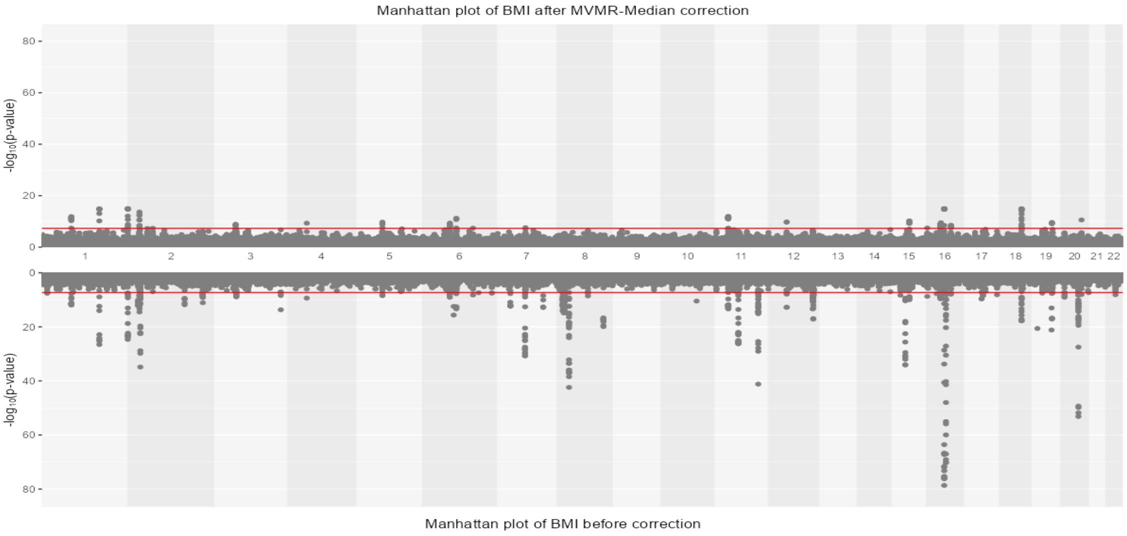

(e)

Fig BC. Manhattan plot of BMI before (upper panel) and after (lower panel) applying bias correction (in M_1). 5 metabolomic PCs are adjusted.

G.2.8 Comparison of Manhattan plots before and after apply different bias-correction methods on M_1, all 20 metabolomic PCs are used in M_1

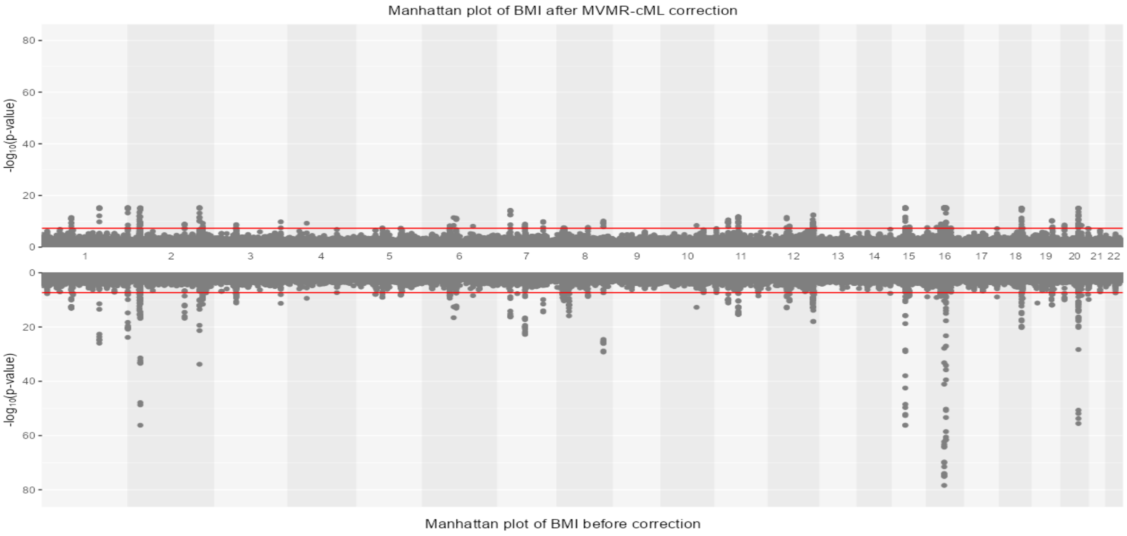

(a)

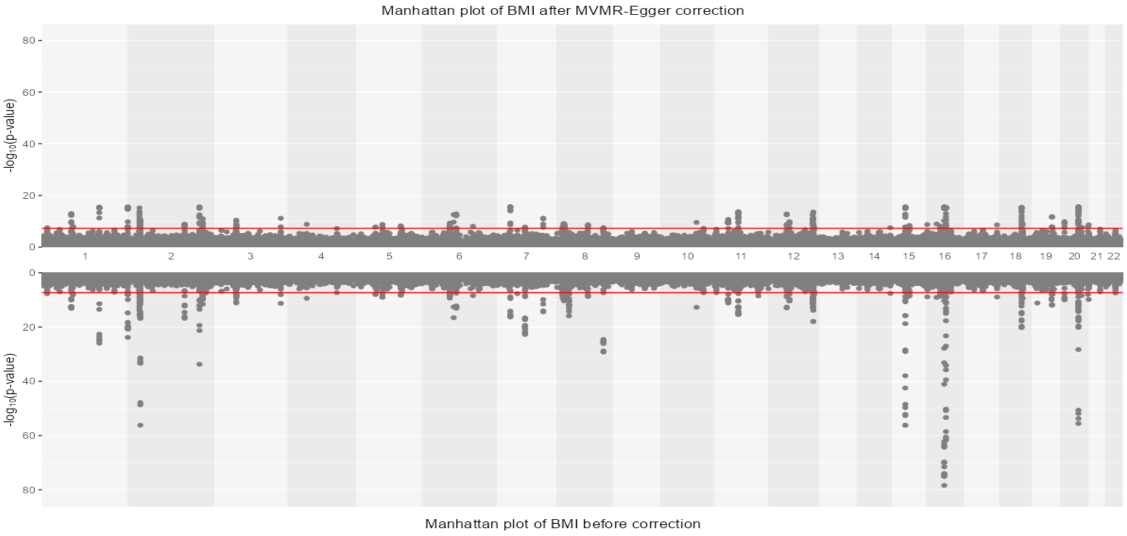

(b)

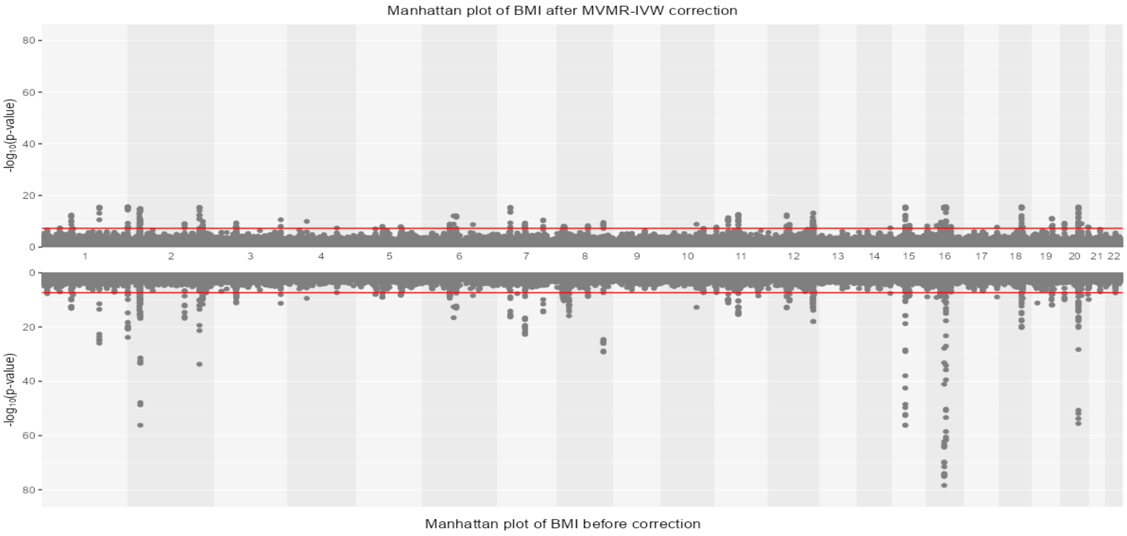

(c)

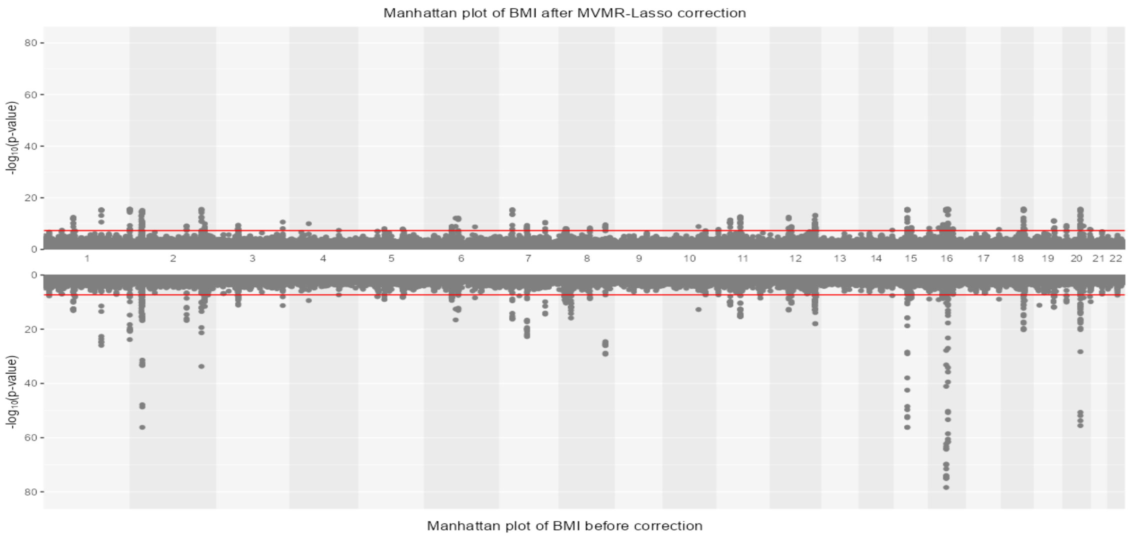
Manhattan plot of BMI after MVMR-Lasso correction
-log10(p-value)
Manhattan plot of BMI before correction

(d)

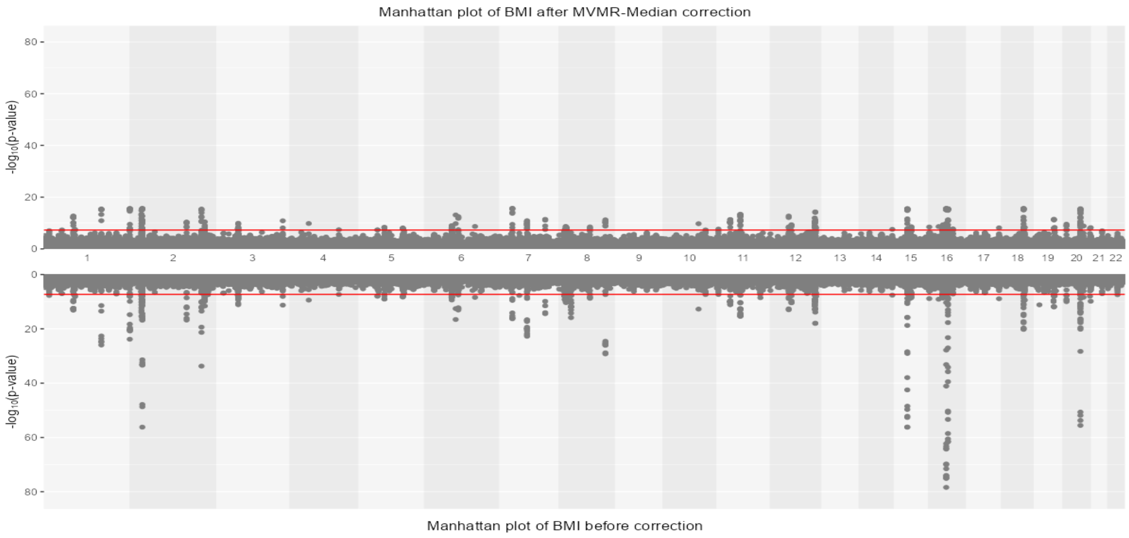
Manhattan plot of BMI after MVMR-Median correction
-log10(p-value)
Manhattan plot of BMI before correction

(e)

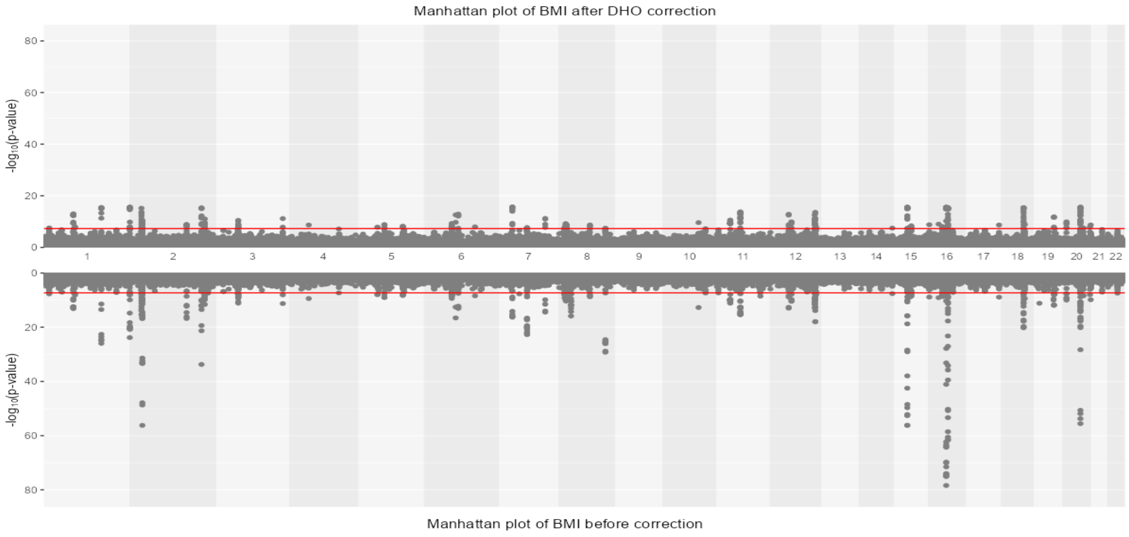
Manhattan plot of BMI after DHO correction
-log10(p-value)
Manhattan plot of BMI before correction

(f)

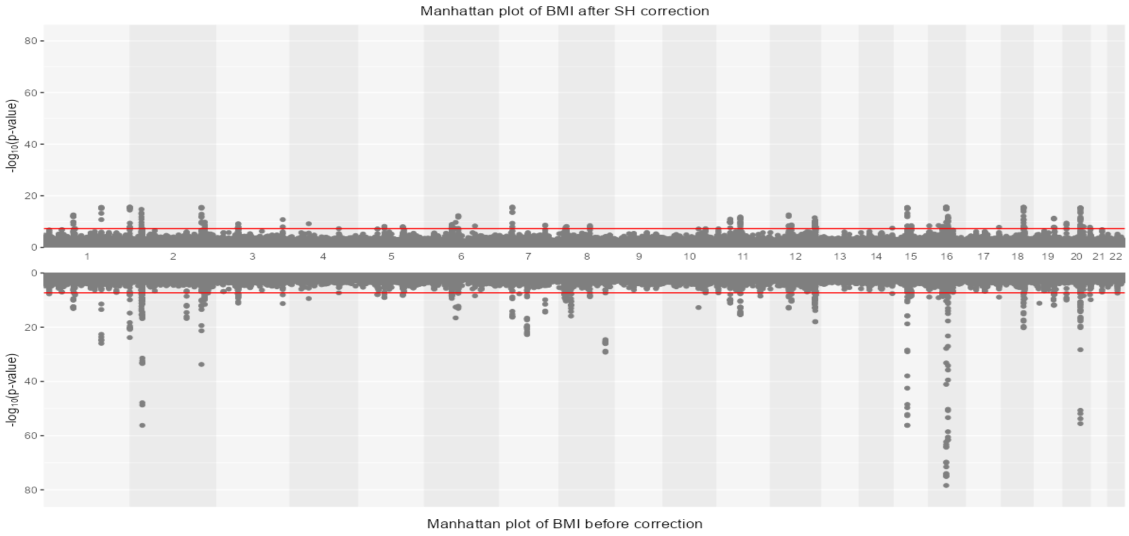

(g)

Fig BD. Manhattan plot of BMI before (upper panel) and after (lower panel) applying bias correction (in M_1). All 20 metabolomic PCs are used in M_1. 1 metabolomic PC is adjusted for bias correction.

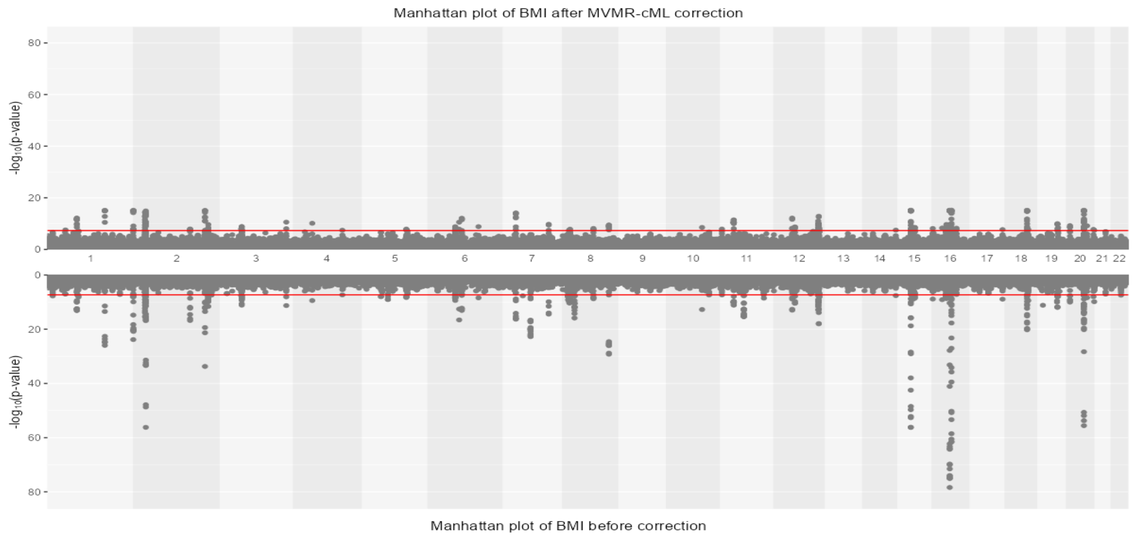
Manhattan plot of BMI after MVMR-cML correction
$-\log_{10}$(p-value)
Manhattan plot of BMI before correction

(a)

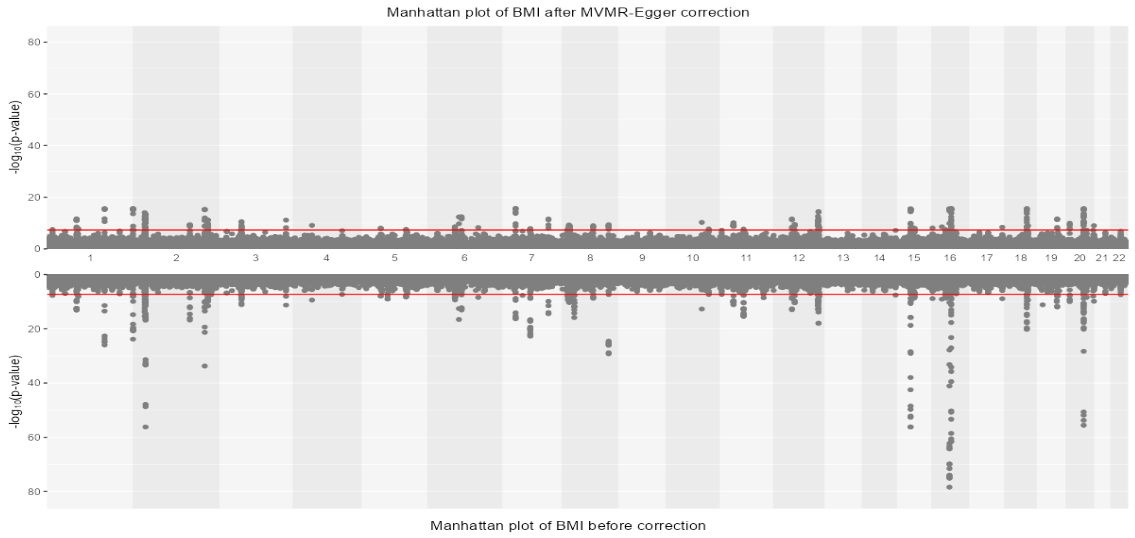
Manhattan plot of BMI after MVMR-Egger correction
$-\log_{10}$(p-value)
Manhattan plot of BMI before correction

(b)

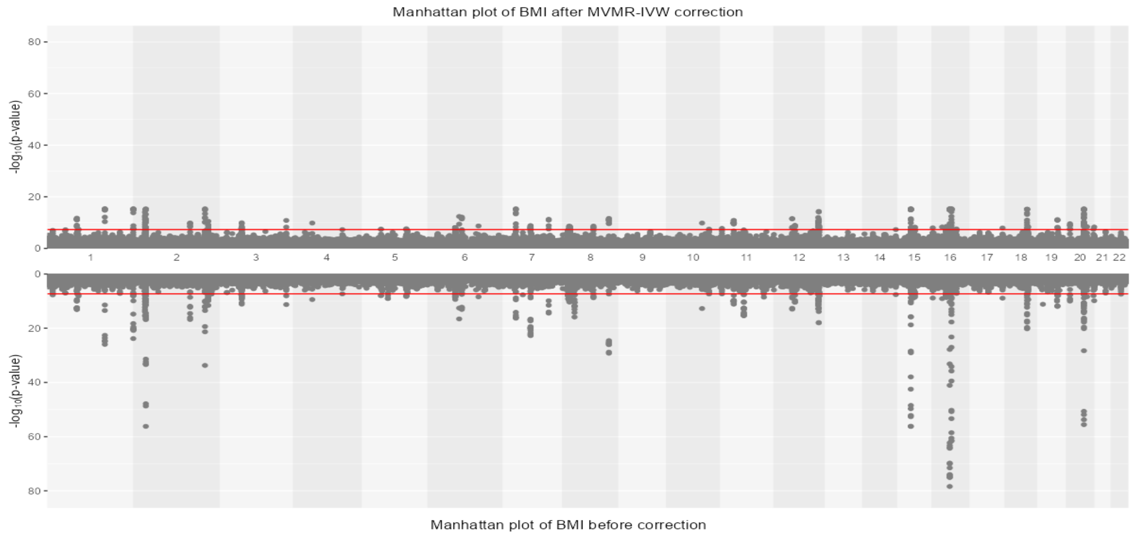
Manhattan plot of BMI after MVMR-IVW correction
$-\log_{10}$(p-value)
Manhattan plot of BMI before correction

(c)

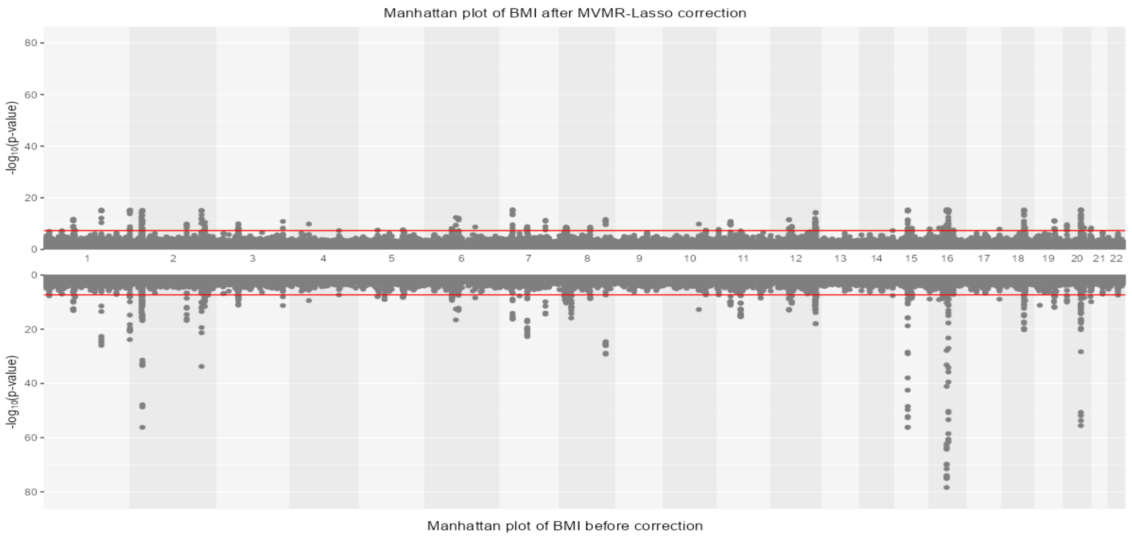

(d)

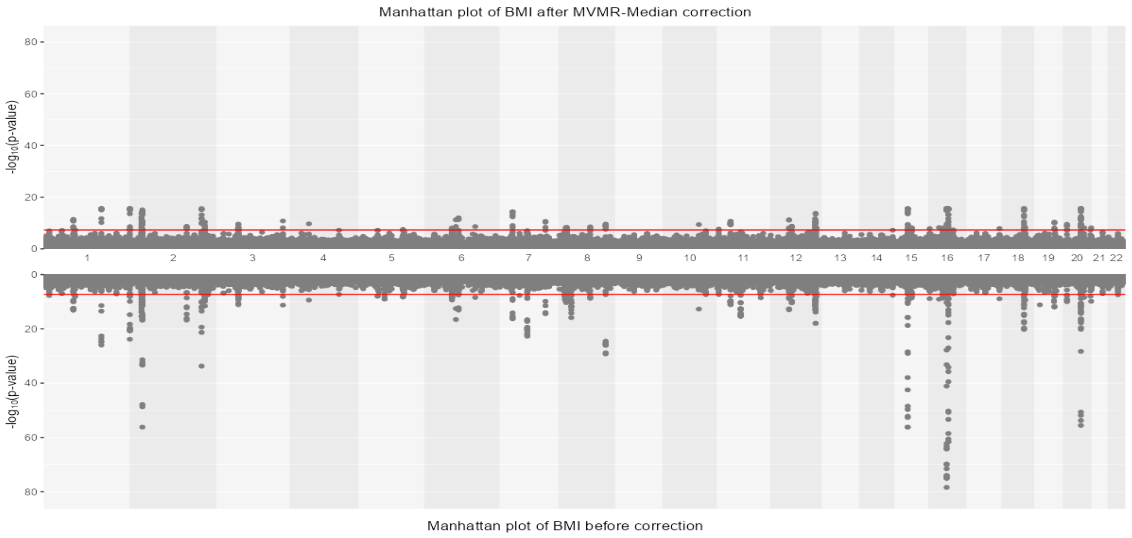

(e)

Fig BE. Manhattan plot of BMI before (upper panel) and after (lower panel) applying bias correction (in M_1). All 20 metabolomic PCs are used in M_1. 2 metabolomic PCs are adjusted for bias correction.

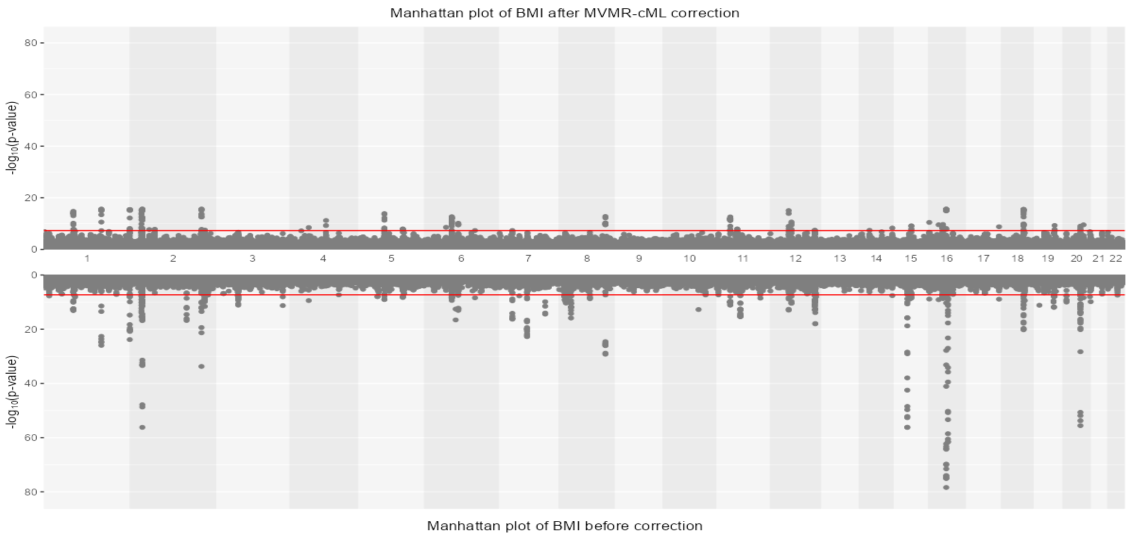
Manhattan plot of BMI after MVMR-cML correction
-log10(p-value)
Manhattan plot of BMI before correction

(a)

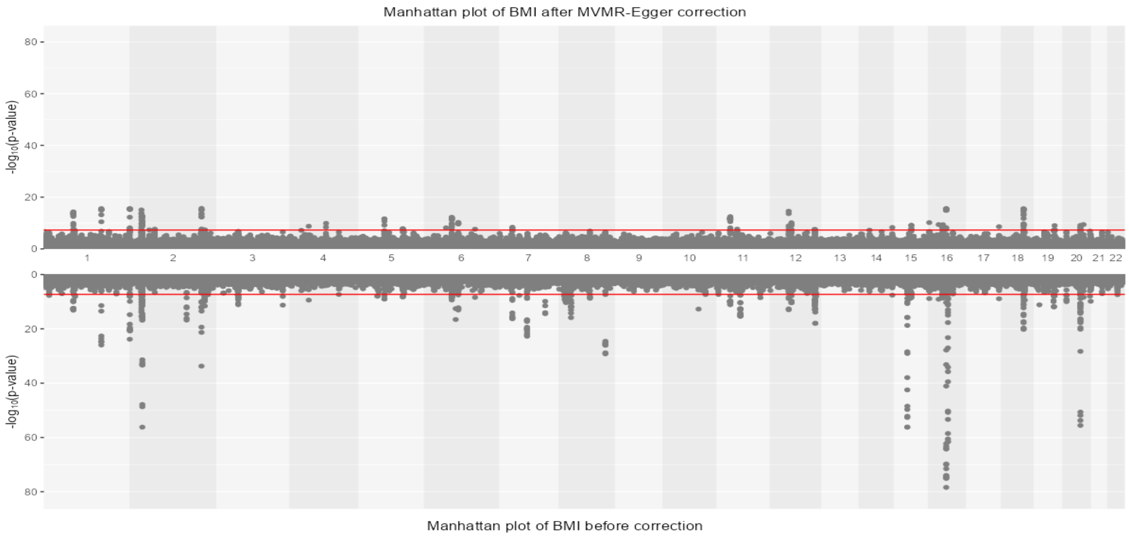
Manhattan plot of BMI after MVMR-Egger correction
-log10(p-value)
Manhattan plot of BMI before correction

(b)

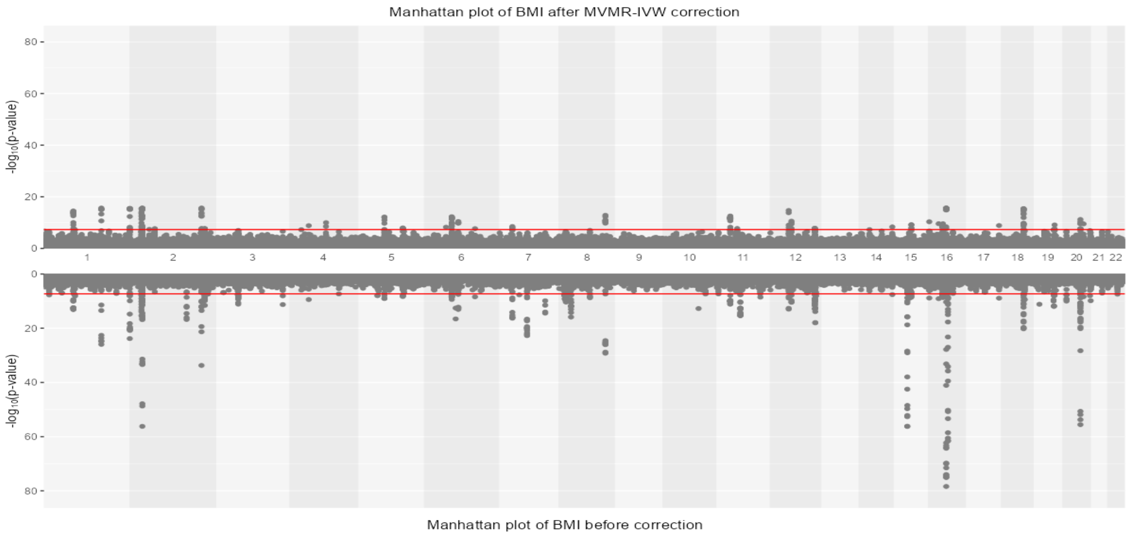
Manhattan plot of BMI after MVMR-IVW correction
-log10(p-value)
Manhattan plot of BMI before correction

(c)

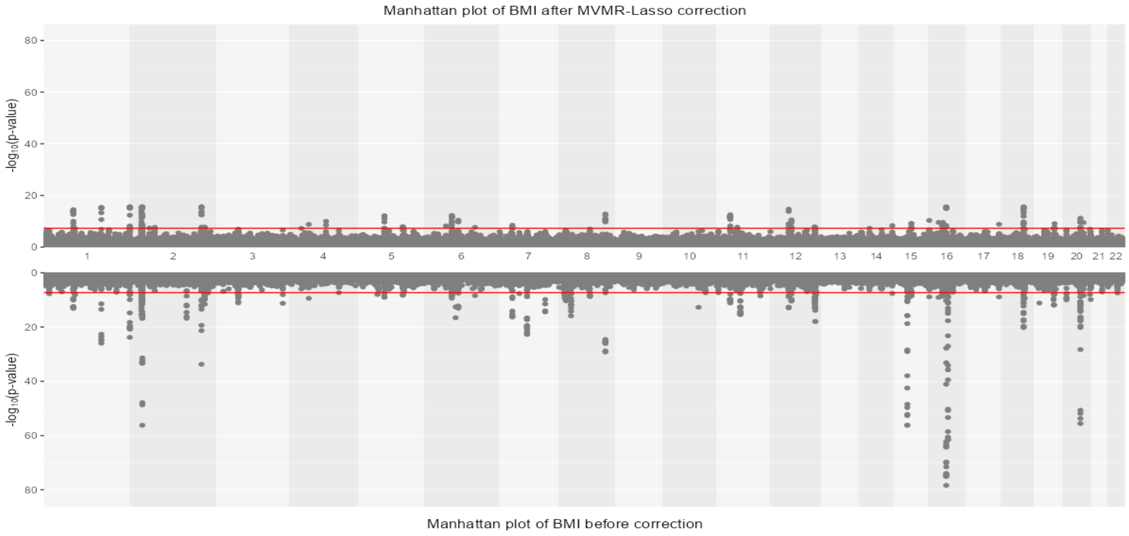

(d)

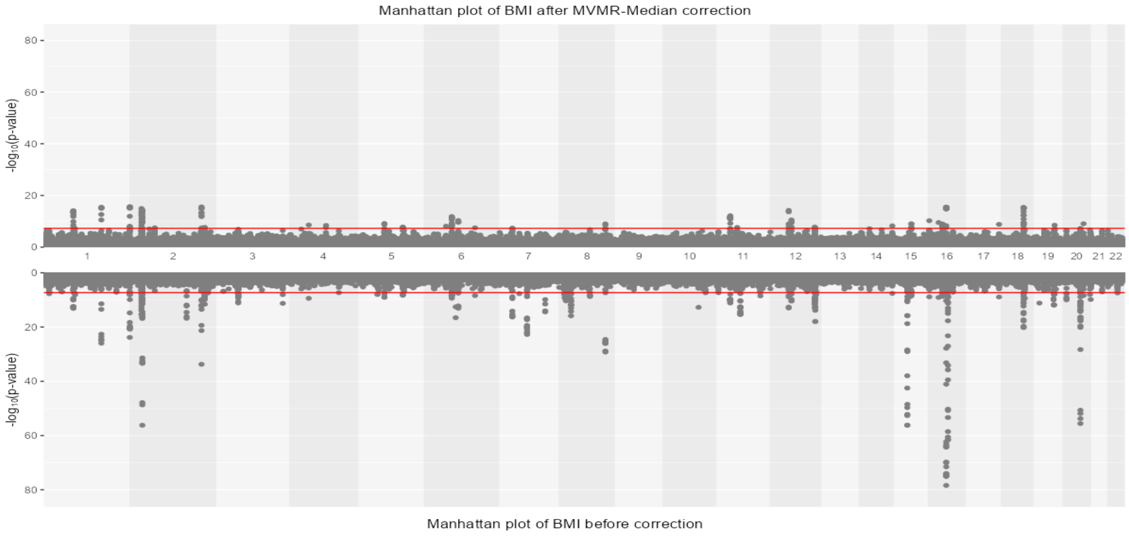

(e)

Fig BF. Manhattan plot of BMI before (upper panel) and after (lower panel) applying bias correction (in M_1). All 20 metabolomic PCs are used in M_1. 3 metabolomic PCs are adjusted for bias correction.

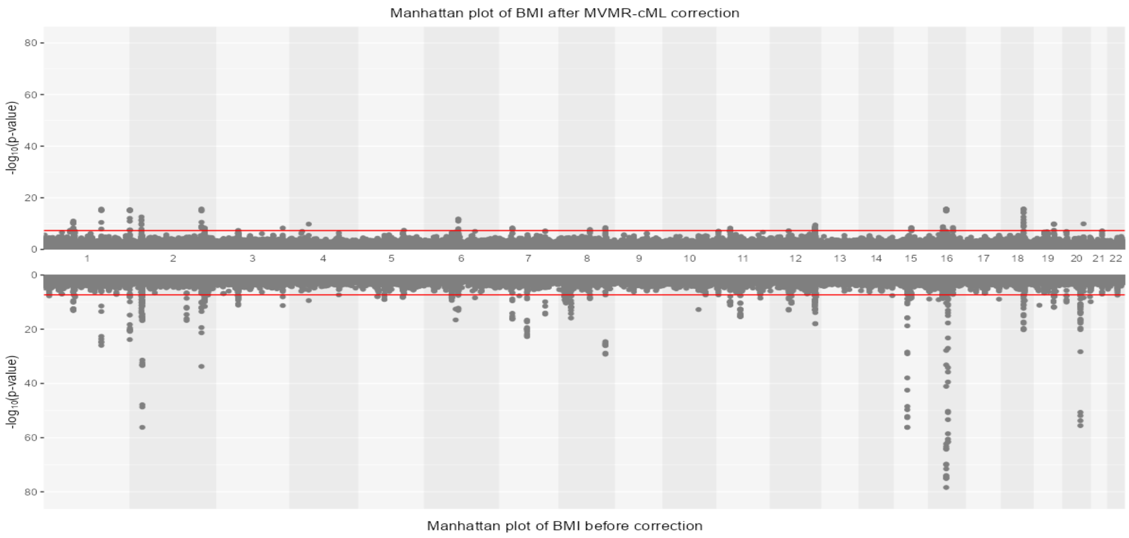
Manhattan plot of BMI after MVMR-cML correction
-log10(p-value)
Manhattan plot of BMI before correction

(a)

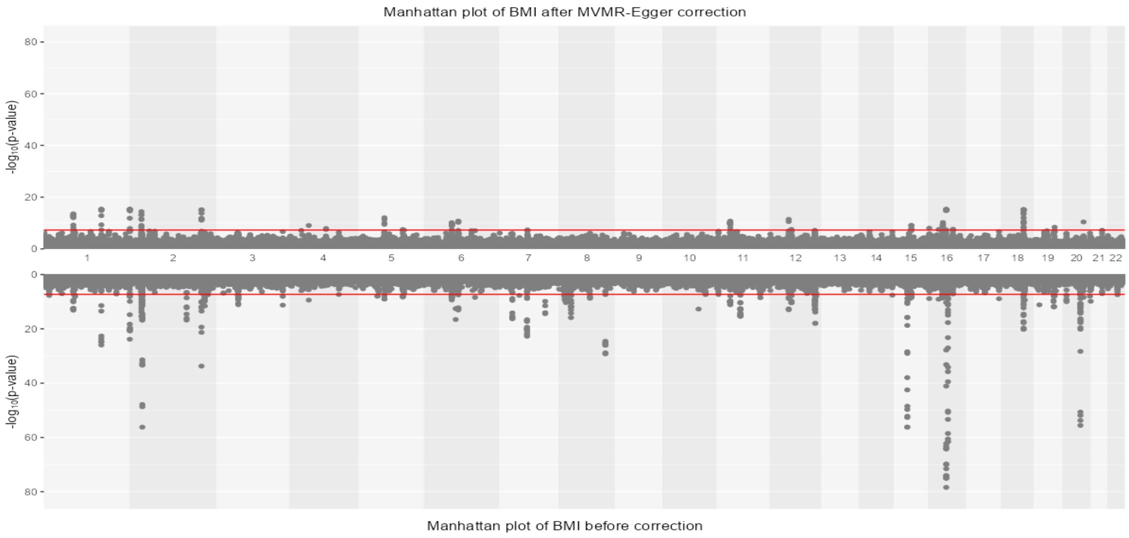
Manhattan plot of BMI after MVMR-Egger correction
-log10(p-value)
Manhattan plot of BMI before correction

(b)

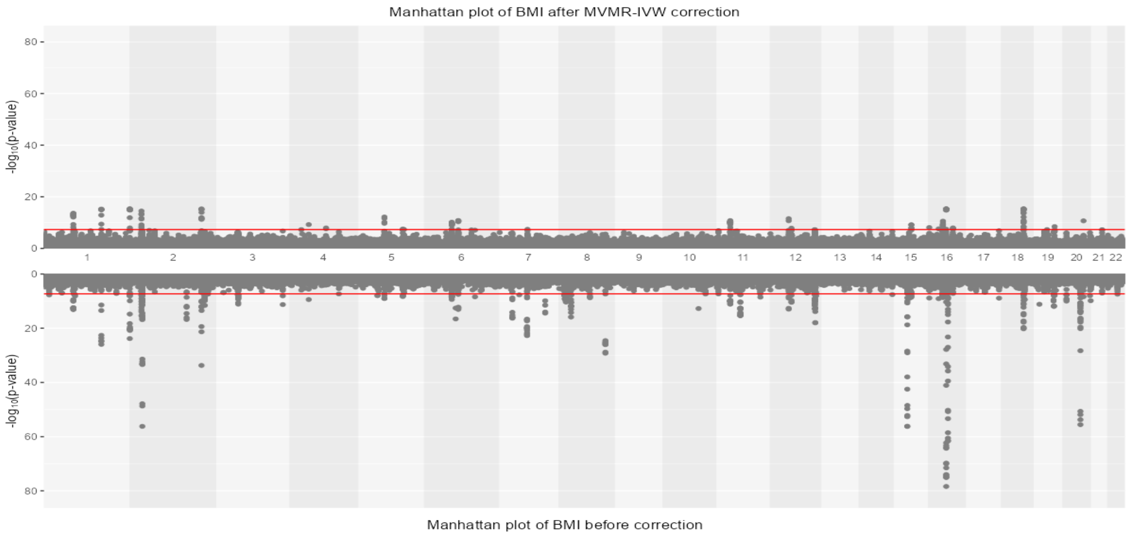
Manhattan plot of BMI after MVMR-IVW correction
-log10(p-value)
Manhattan plot of BMI before correction

(c)

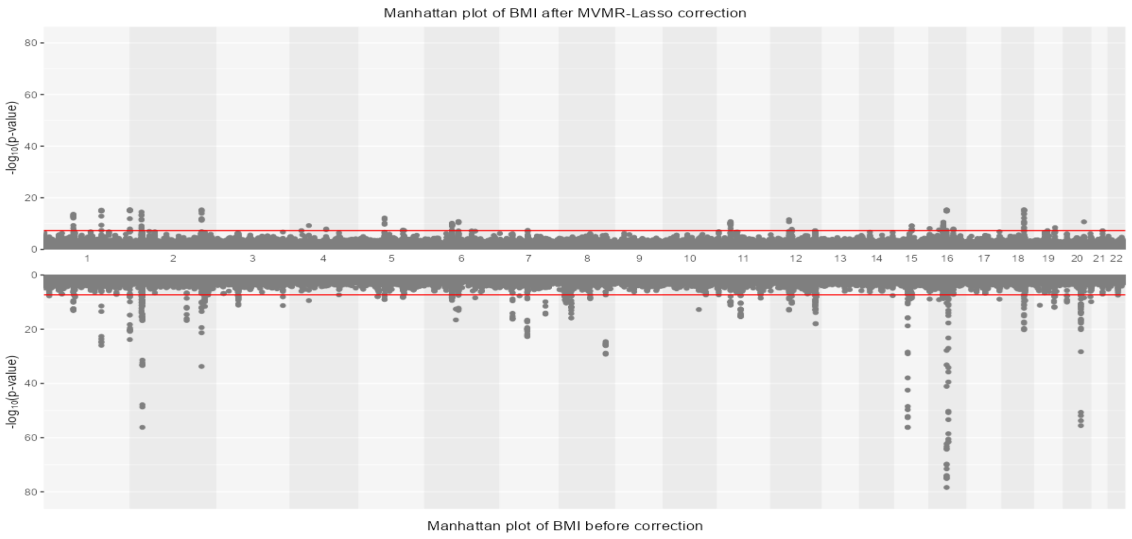

(d)

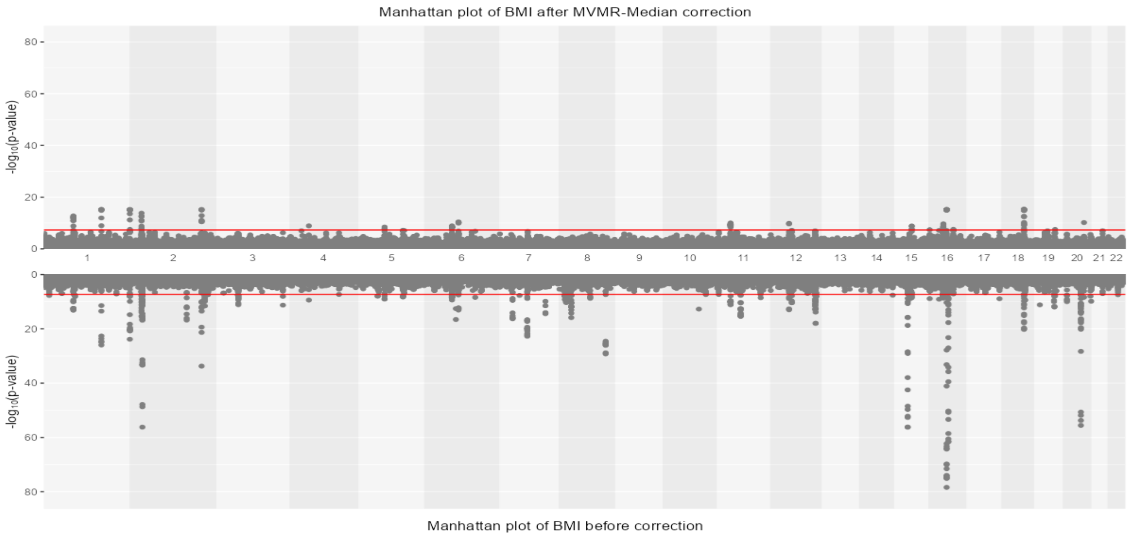

(e)

Fig BG. Manhattan plot of BMI before (upper panel) and after (lower panel) applying bias correction (in M_1). All 20 metabolomic PCs are used in M_1. 4 metabolomic PCs are adjusted for bias correction.

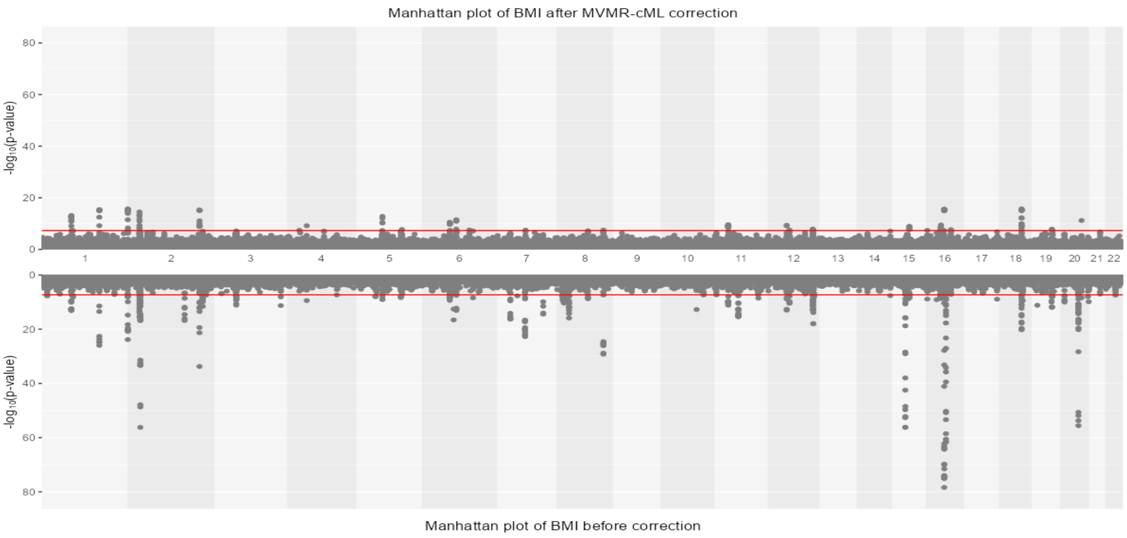
Manhattan plot of BMI after MVMR-cML correction
-log10(p-value)
80
60
40
20
0
1 2 3 4 5 6 7 8 9 10 11 12 13 14 15 16 17 18 19 20 21 22
Manhattan plot of BMI before correction

(a)

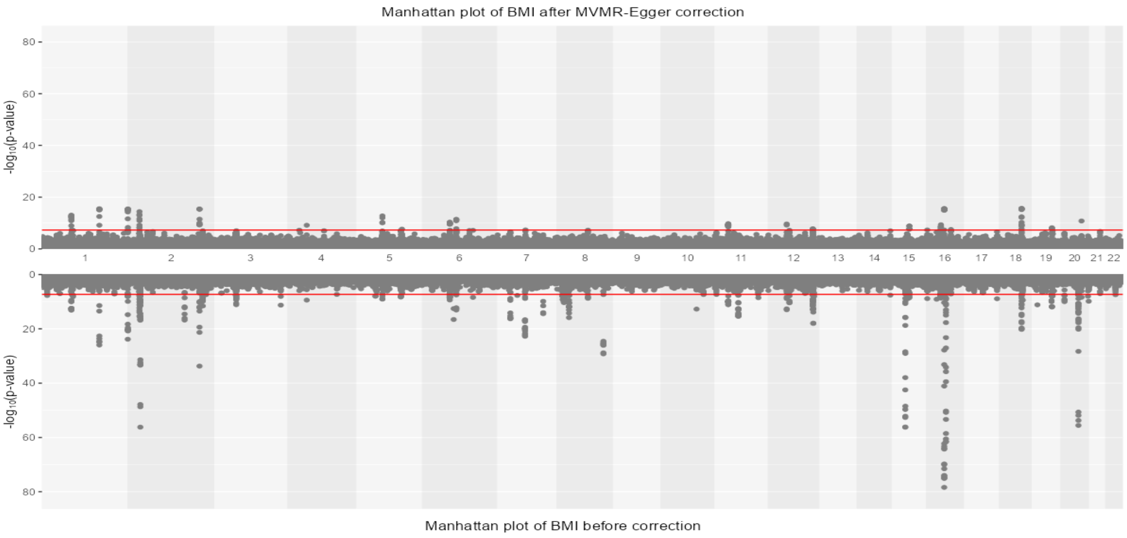
Manhattan plot of BMI after MVMR-Egger correction
-log10(p-value)
80
60
40
20
0
1 2 3 4 5 6 7 8 9 10 11 12 13 14 15 16 17 18 19 20 21 22
Manhattan plot of BMI before correction

(b)

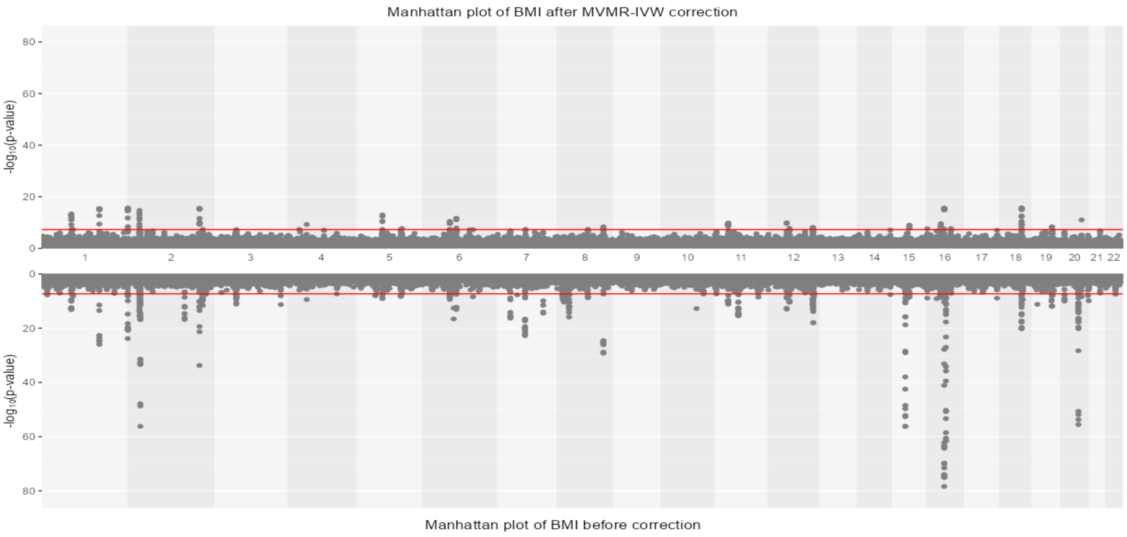
Manhattan plot of BMI after MVMR-IVW correction
-log10(p-value)
80
60
40
20
0
1 2 3 4 5 6 7 8 9 10 11 12 13 14 15 16 17 18 19 20 21 22
Manhattan plot of BMI before correction

(c)

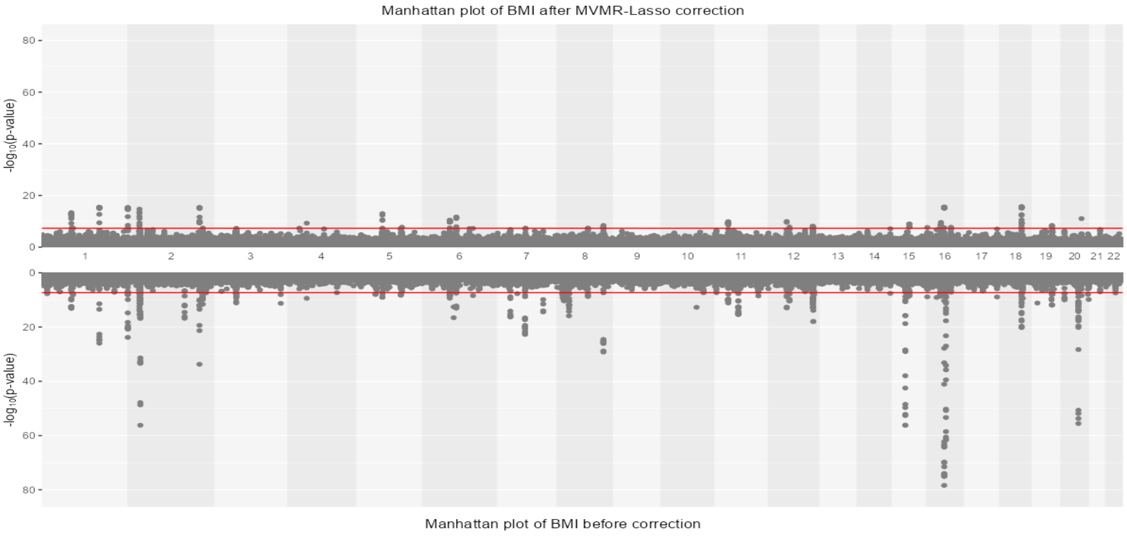

(d)

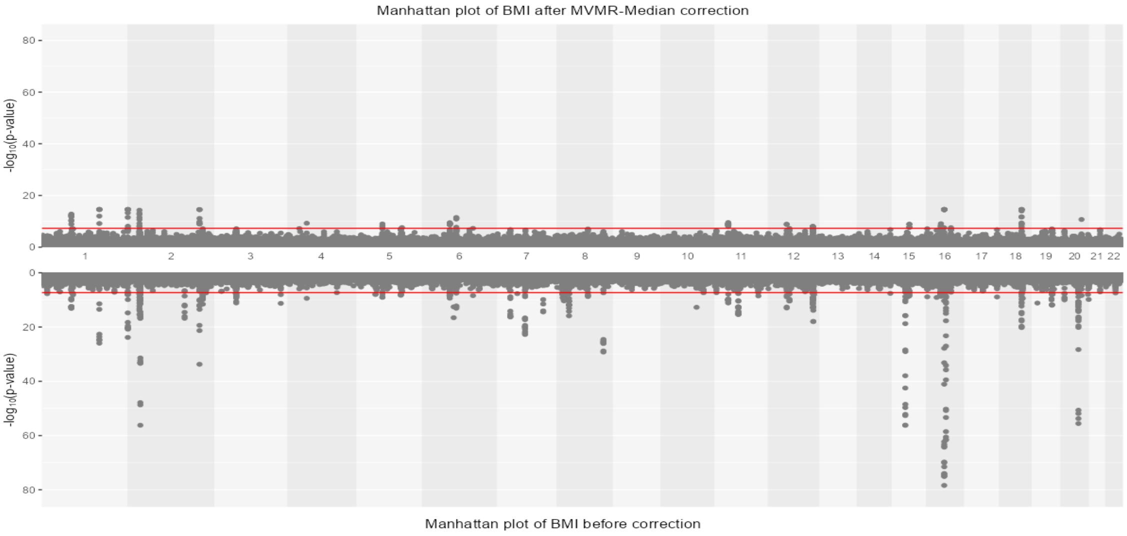

(e)

Fig BH. Manhattan plot of BMI before (upper panel) and after (lower panel) applying bias correction (in M_1). All 20 metabolomic PCs are used in M_1. 5 metabolomic PCs are adjusted for bias correction.

G.2.9 Comparison of Manhattan plots before and after apply different bias-correction methods on M_2

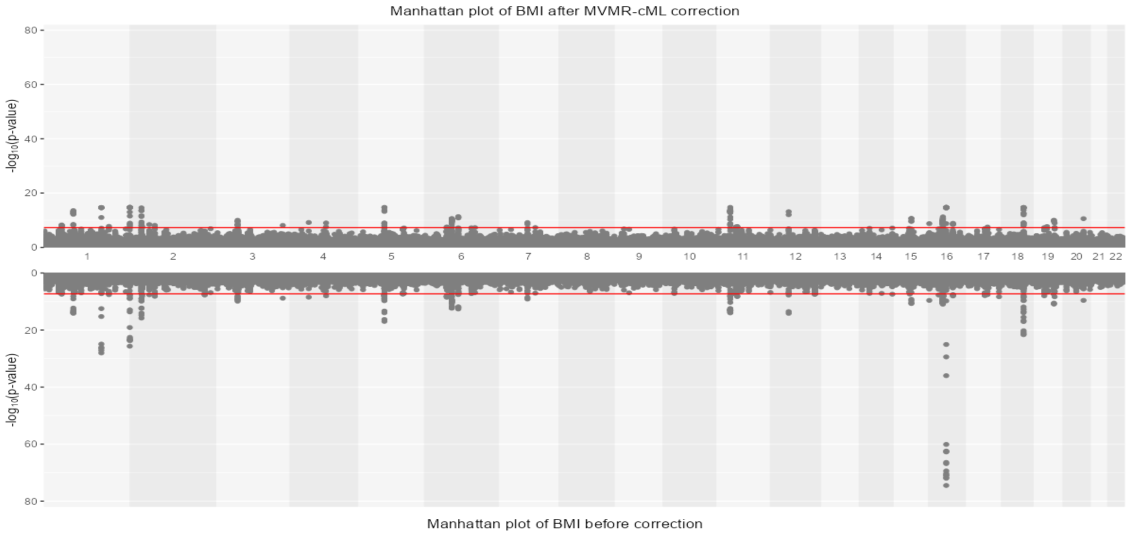

(a)

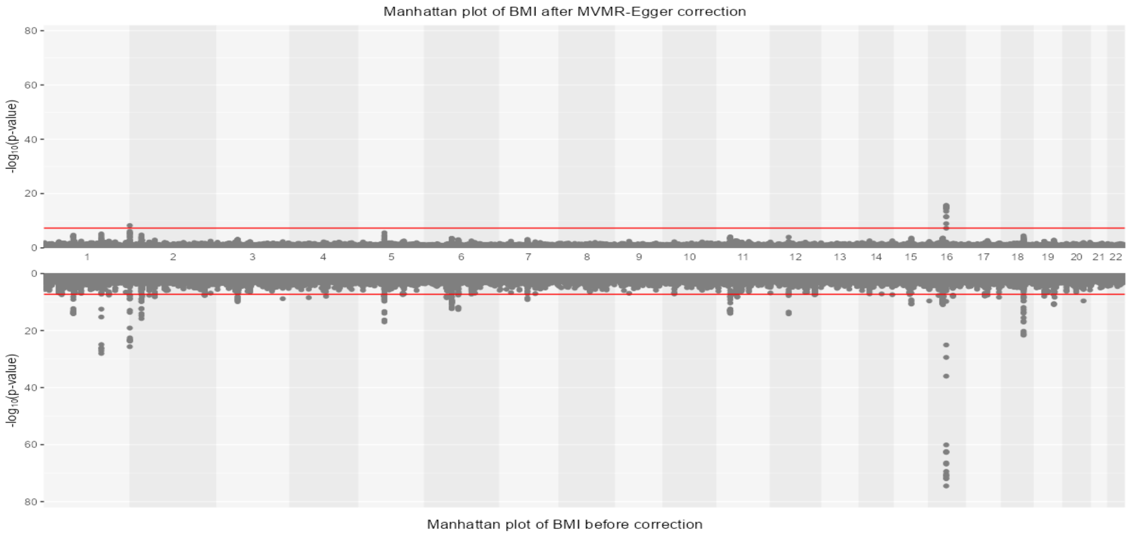

(b)

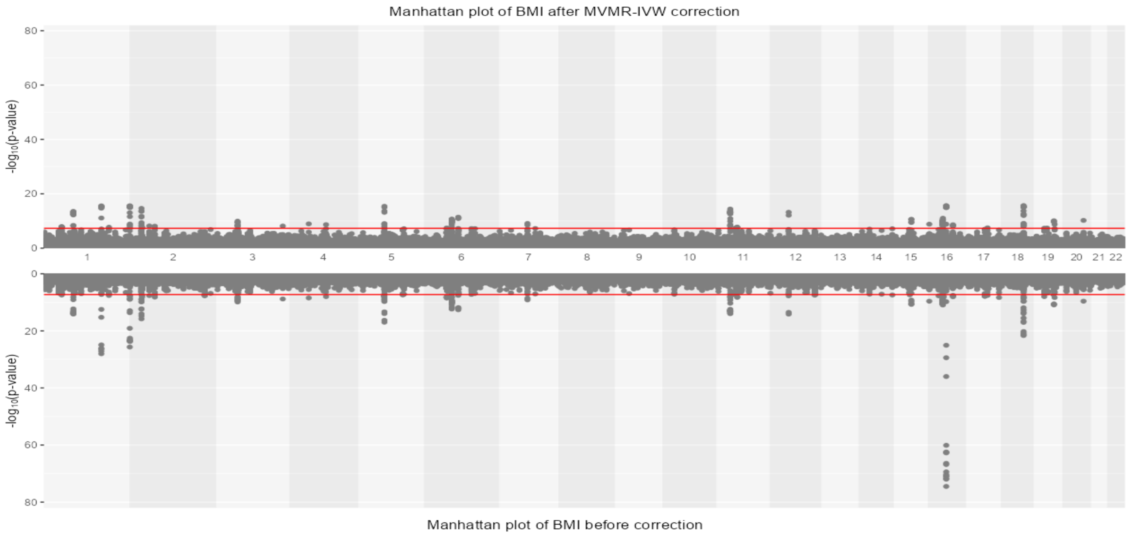

(c)

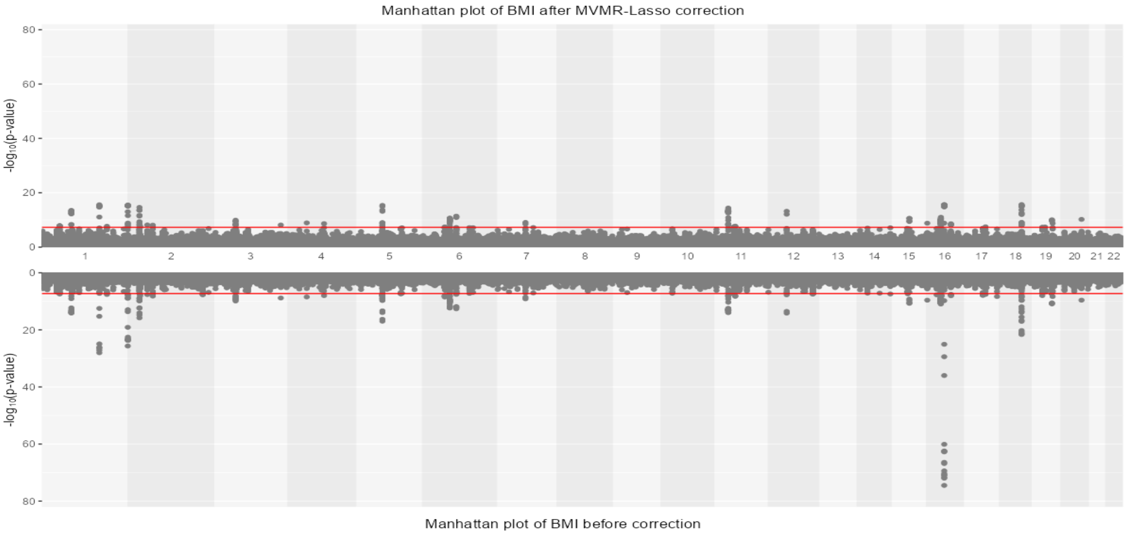
Manhattan plot of BMI after MVMR-Lasso correction
Manhattan plot of BMI before correction

(d)

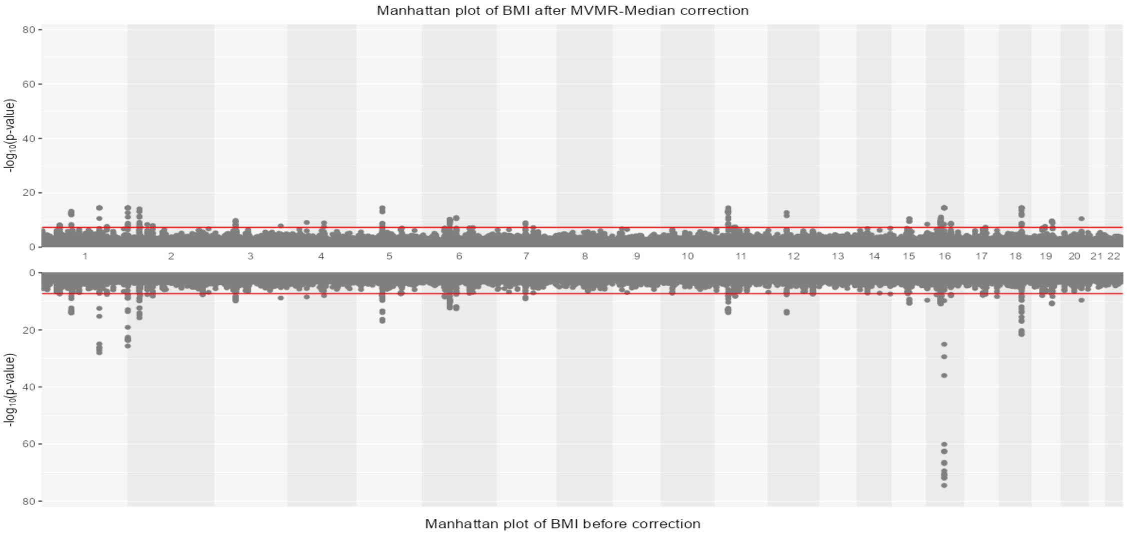
Manhattan plot of BMI after MVMR-Median correction
Manhattan plot of BMI before correction

(e)

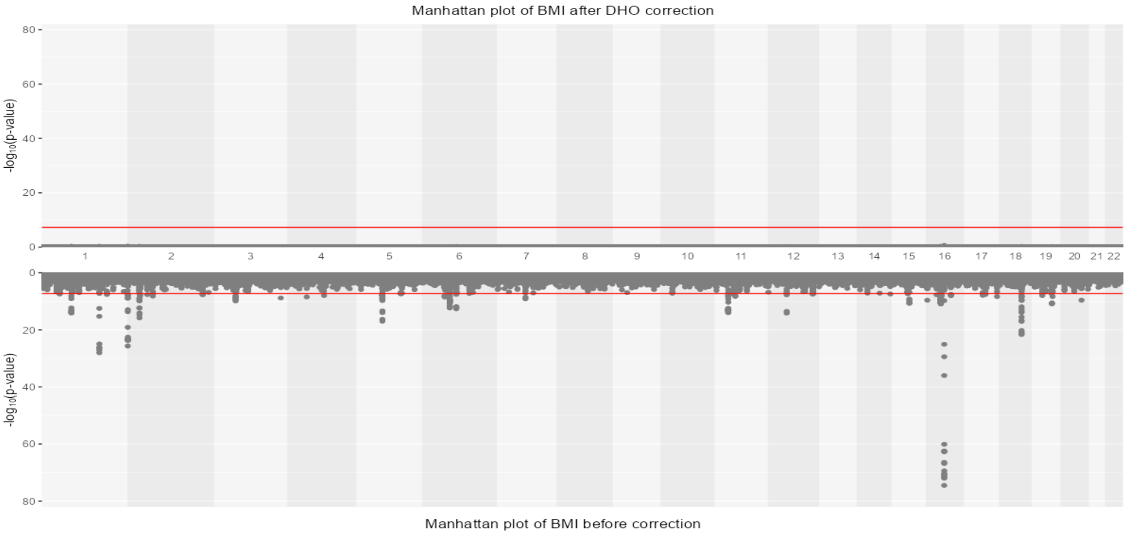
Manhattan plot of BMI after DHO correction
Manhattan plot of BMI before correction

(f)

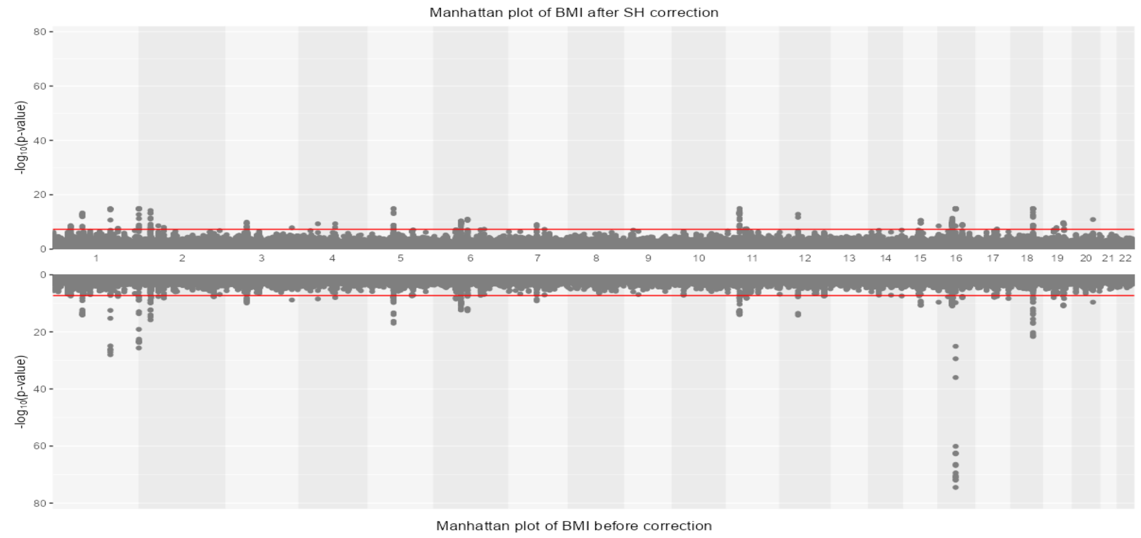

(g)

Fig BI. Manhattan plot of BMI before (upper panel) and after (lower panel) applying bias correction (in M_2). 1 metabolomic PC is adjusted.

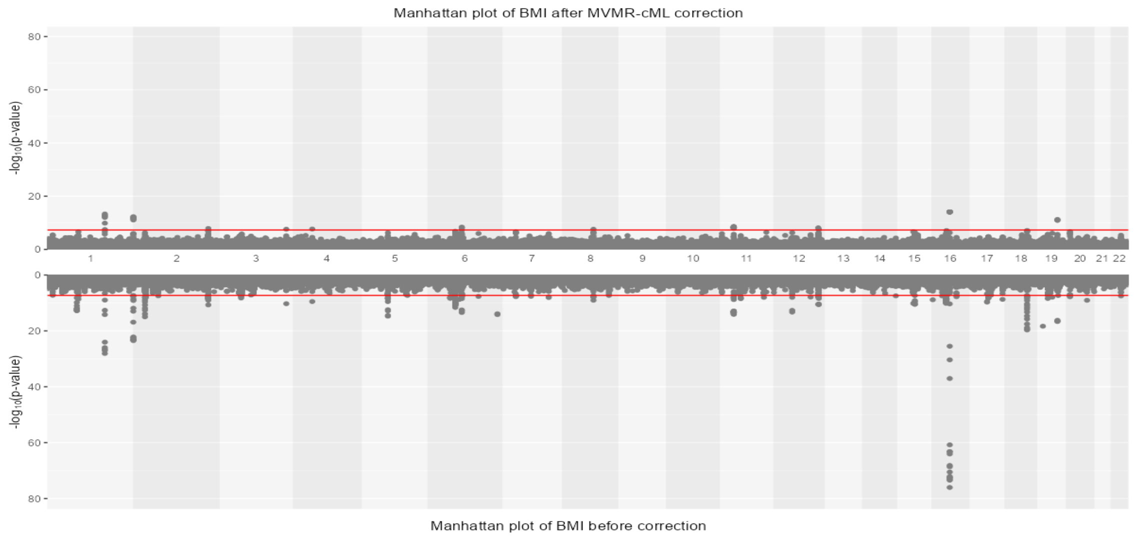
Manhattan plot of BMI after MVMR-cML correction
-log10(p-value)
0
20
40
60
80
1 2 3 4 5 6 7 8 9 10 11 12 13 14 15 16 17 18 19 20 21 22
-log10(p-value)
Manhattan plot of BMI before correction

(a)

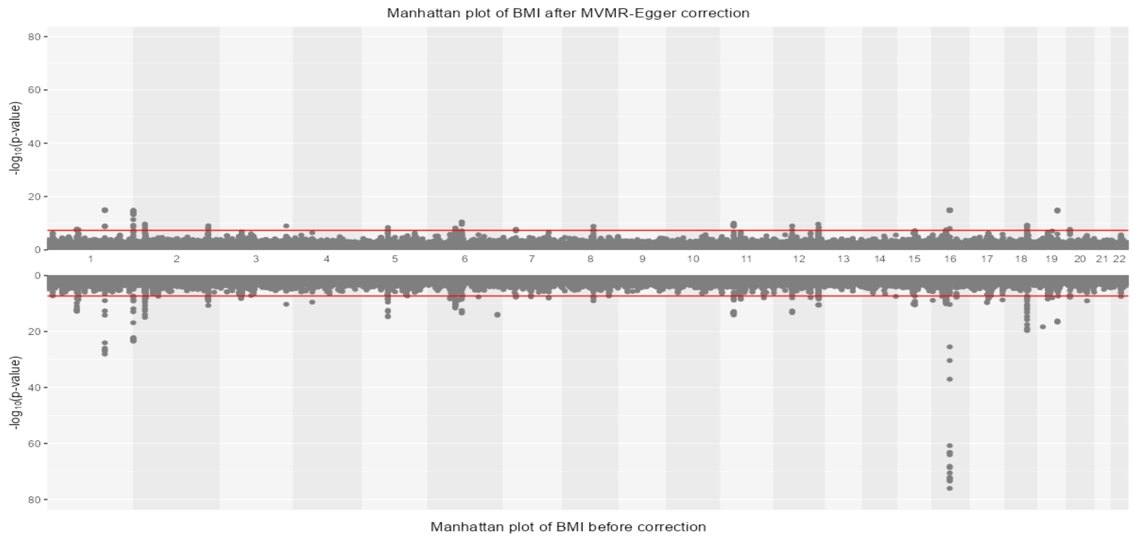
Manhattan plot of BMI after MVMR-Egger correction
-log10(p-value)
0
20
40
60
80
1 2 3 4 5 6 7 8 9 10 11 12 13 14 15 16 17 18 19 20 21 22
-log10(p-value)
Manhattan plot of BMI before correction

(b)

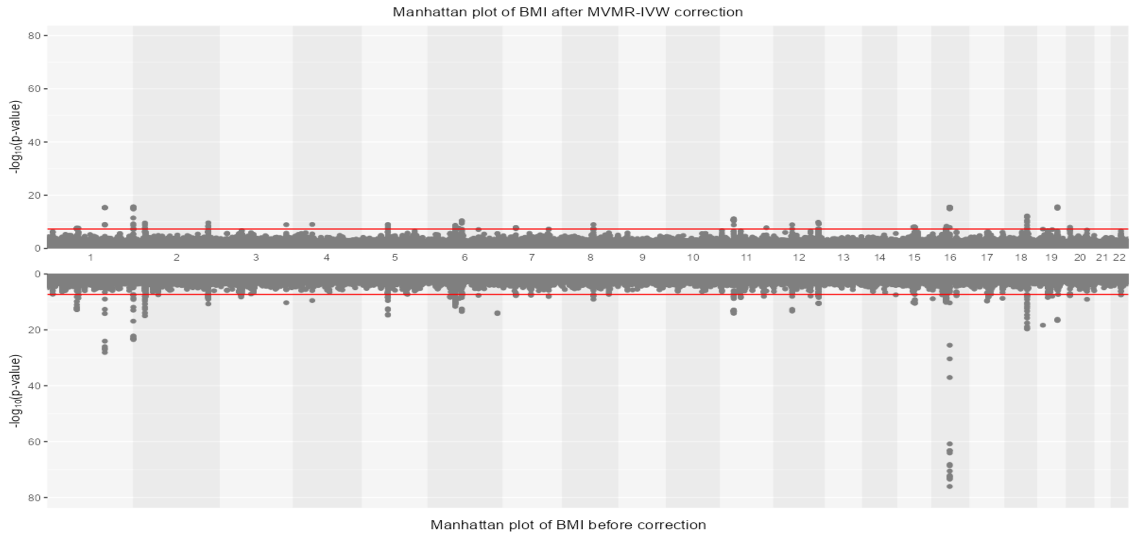
Manhattan plot of BMI after MVMR-IVW correction
-log10(p-value)
0
20
40
60
80
1 2 3 4 5 6 7 8 9 10 11 12 13 14 15 16 17 18 19 20 21 22
-log10(p-value)
Manhattan plot of BMI before correction

(c)

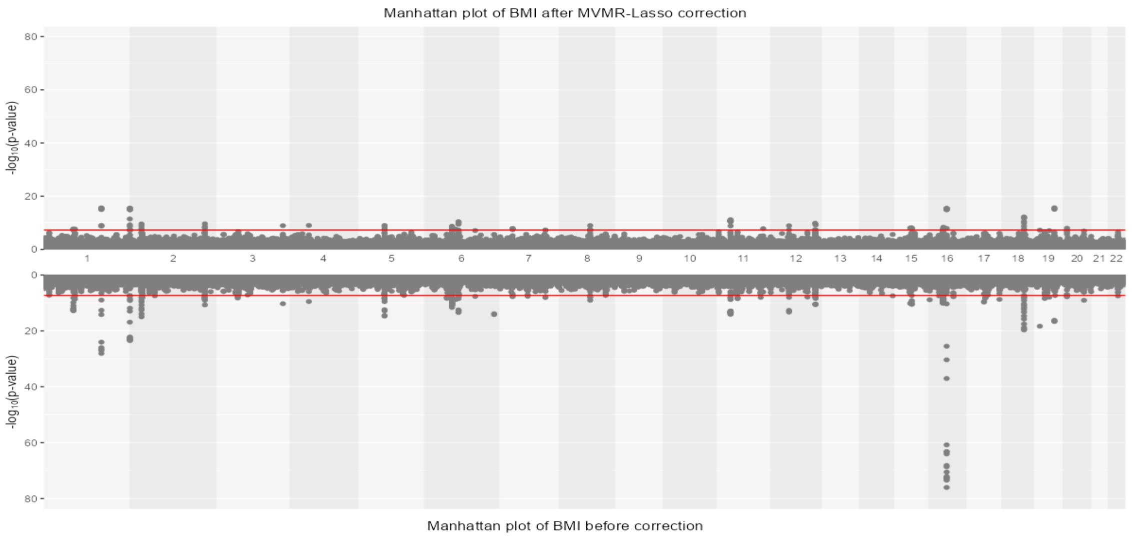

(d)

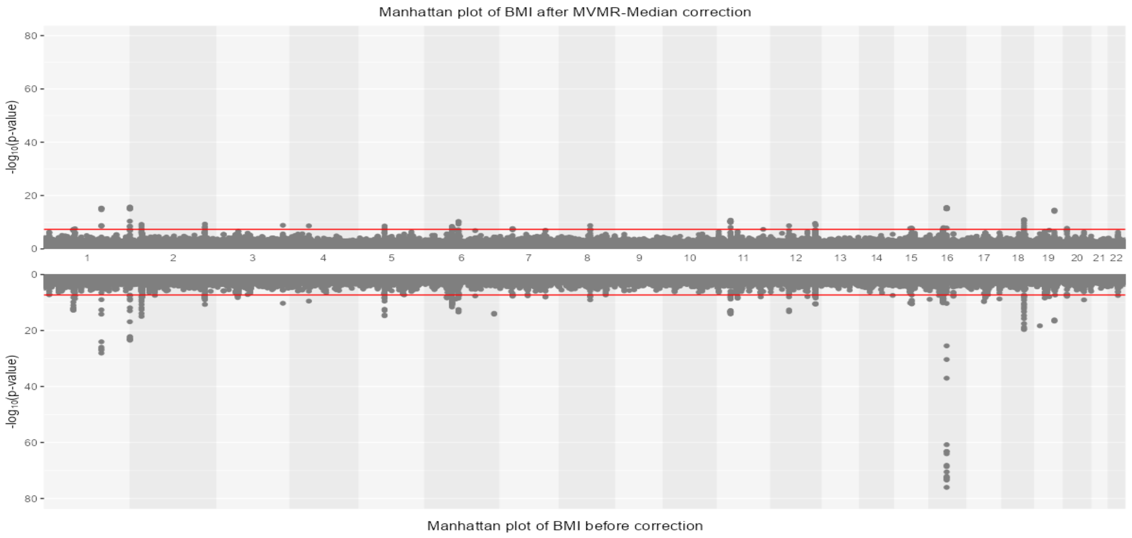

(e)

Fig BJ. Manhattan plot of BMI before (upper panel) and after (lower panel) applying bias correction (in M_2). 2 metabolomic PCs are adjusted.

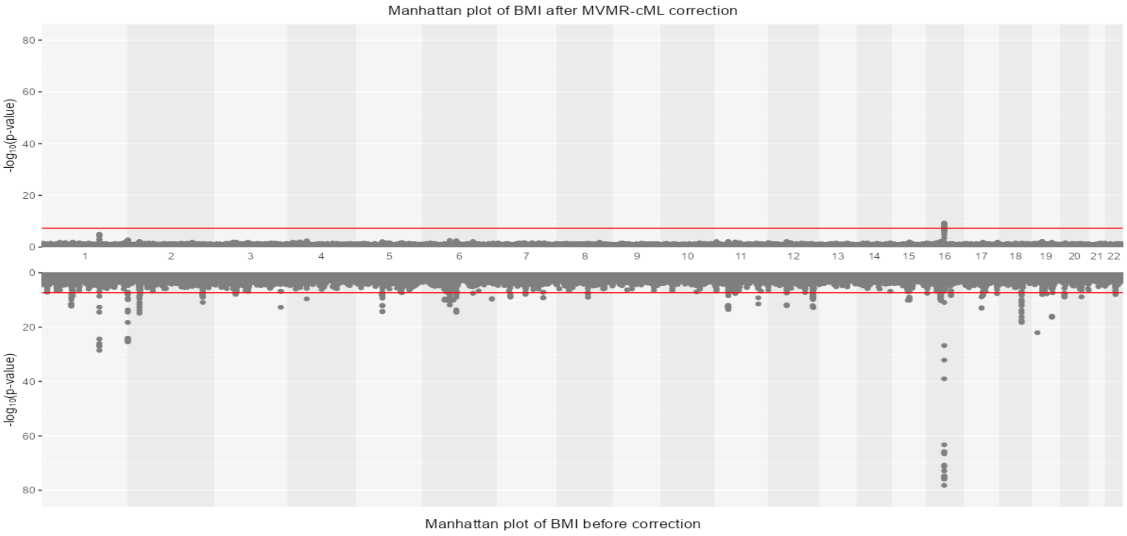
Manhattan plot of BMI after MVMR-cML correction
-log10(p-value)
80
60
40
20
0
1 2 3 4 5 6 7 8 9 10 11 12 13 14 15 16 17 18 19 20 21 22
Manhattan plot of BMI before correction

(a)

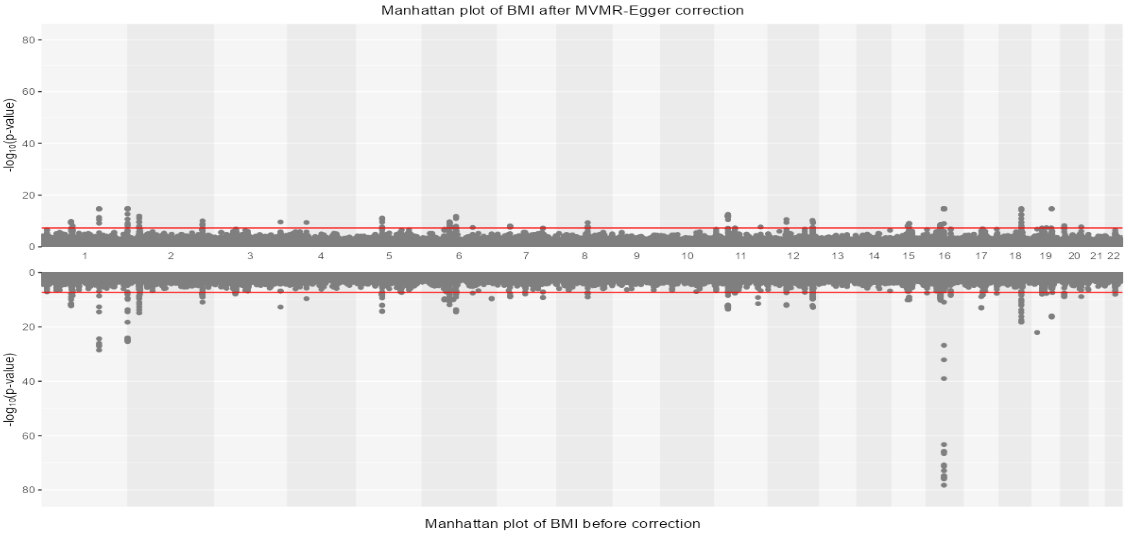
Manhattan plot of BMI after MVMR-Egger correction
-log10(p-value)
80
60
40
20
0
1 2 3 4 5 6 7 8 9 10 11 12 13 14 15 16 17 18 19 20 21 22
Manhattan plot of BMI before correction

(b)

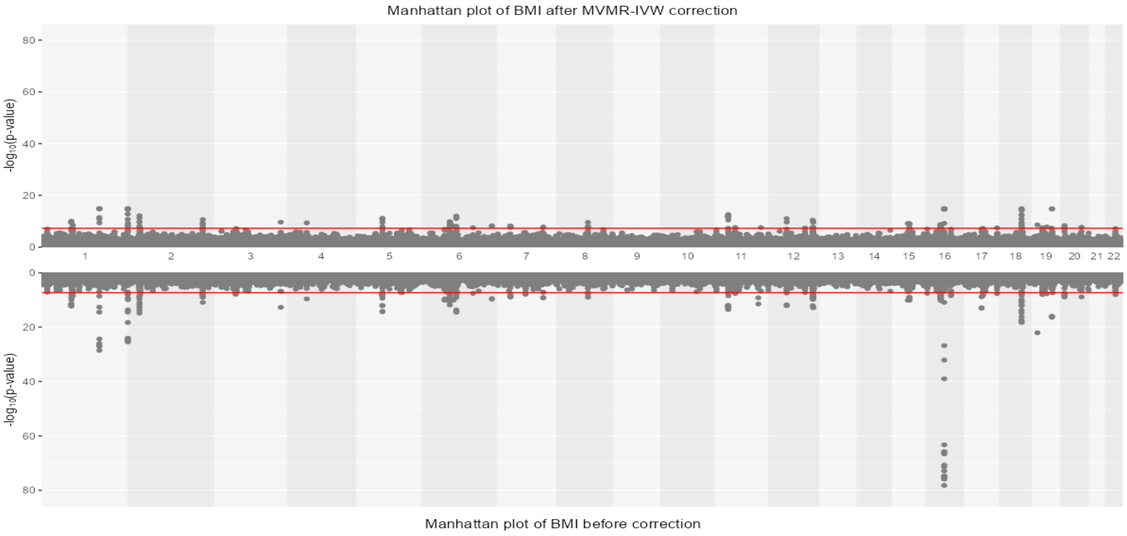
Manhattan plot of BMI after MVMR-IVW correction
-log10(p-value)
80
60
40
20
0
1 2 3 4 5 6 7 8 9 10 11 12 13 14 15 16 17 18 19 20 21 22
Manhattan plot of BMI before correction

(c)

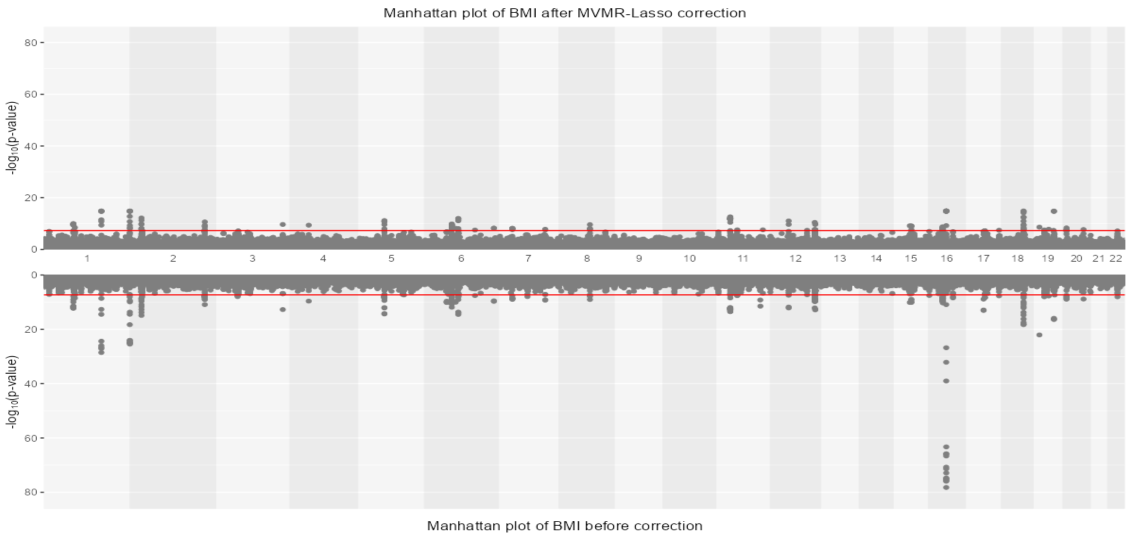

(d)

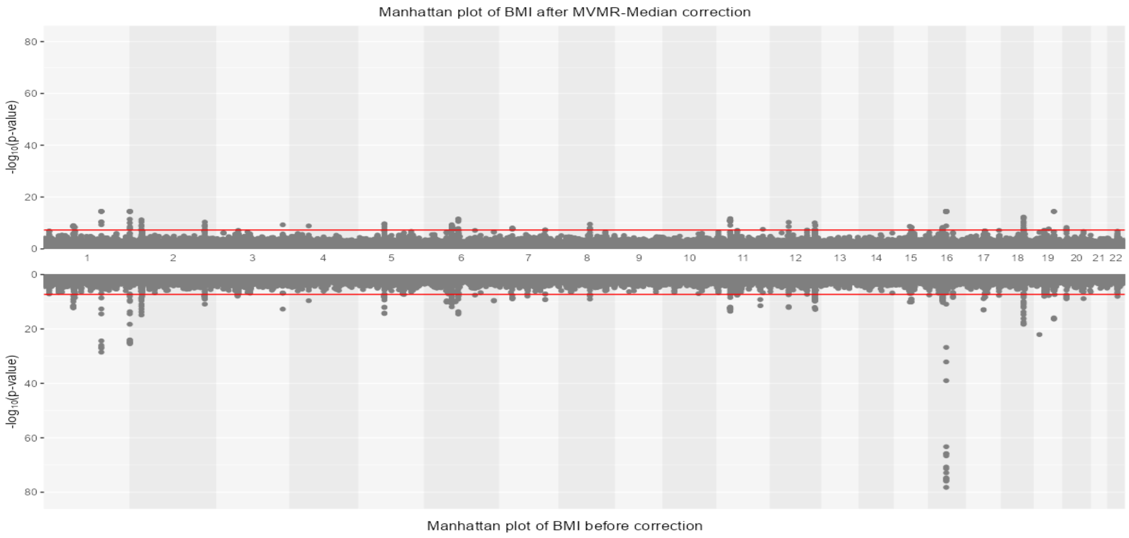

(e)

Fig BK. Manhattan plot of BMI before (upper panel) and after (lower panel) applying bias correction (in M_2). 3 metabolomic PCs are adjusted.

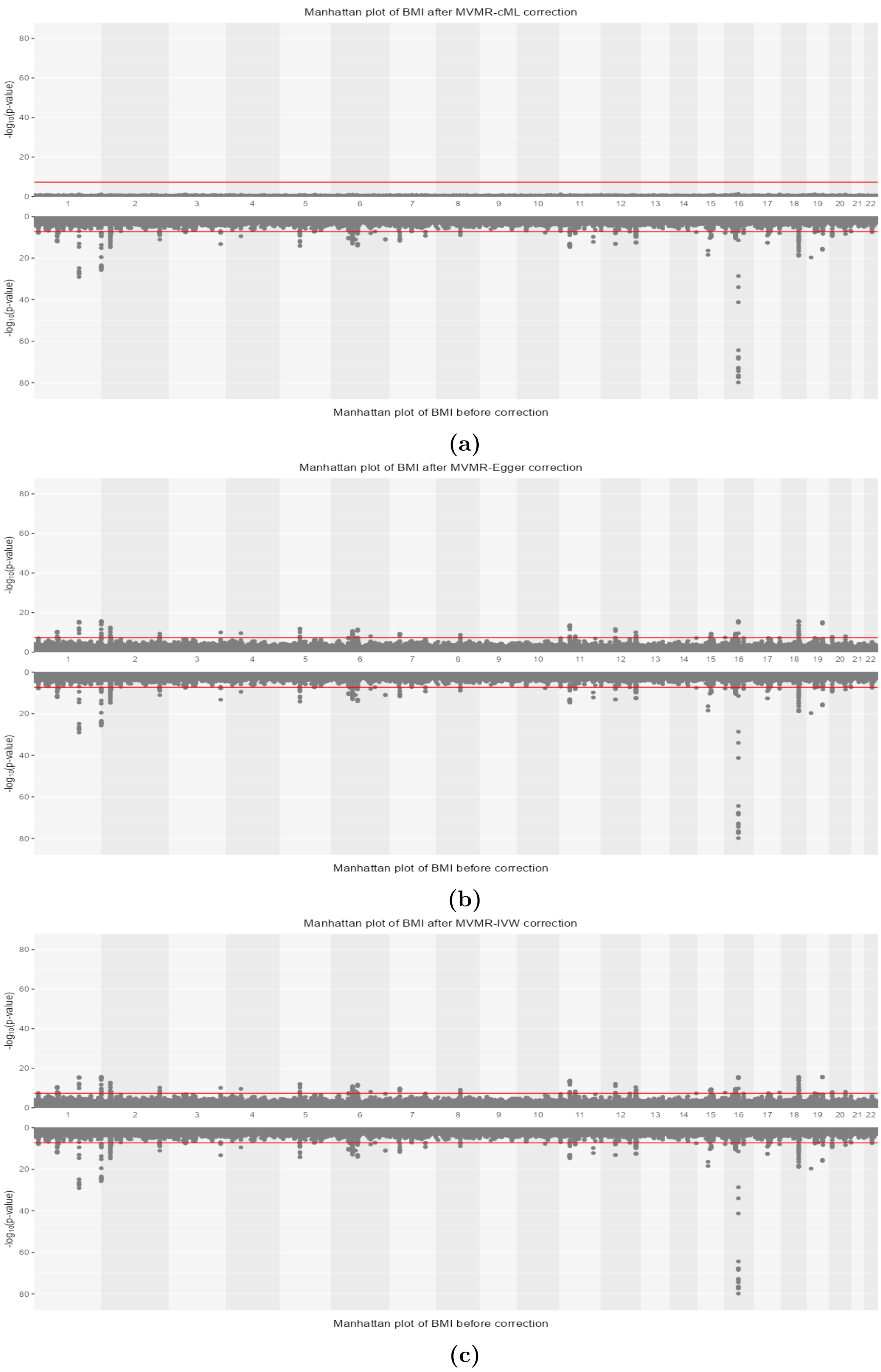
Manhattan plot of BMI after MVMR-cML correction
-log10(p-value)
Manhattan plot of BMI before correction
Manhattan plot of BMI after MVMR-Egger correction
Manhattan plot of BMI after MVMR-IVW correction

(a)

(b)

(c)

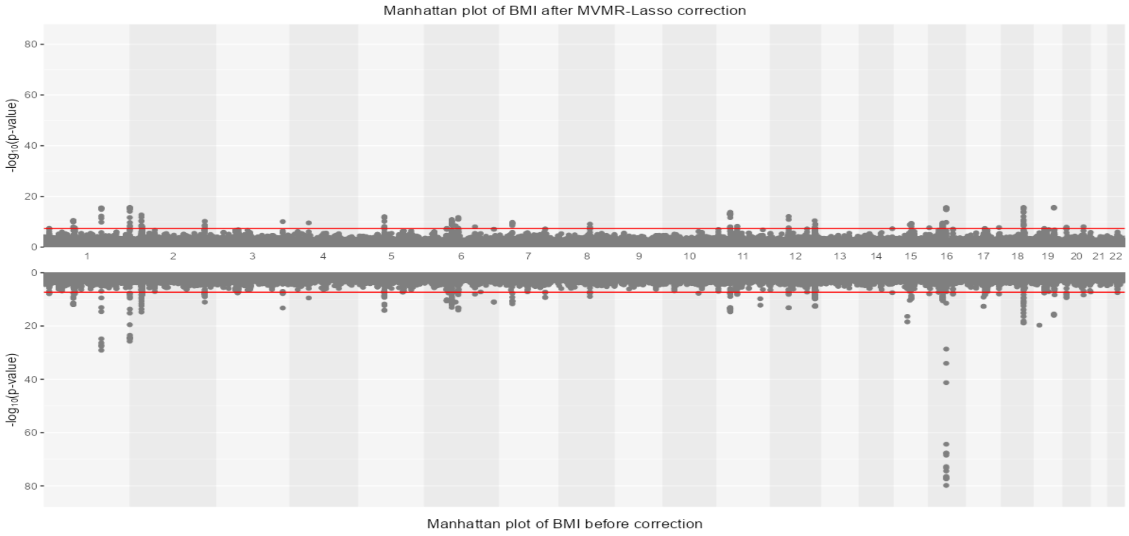

(d)

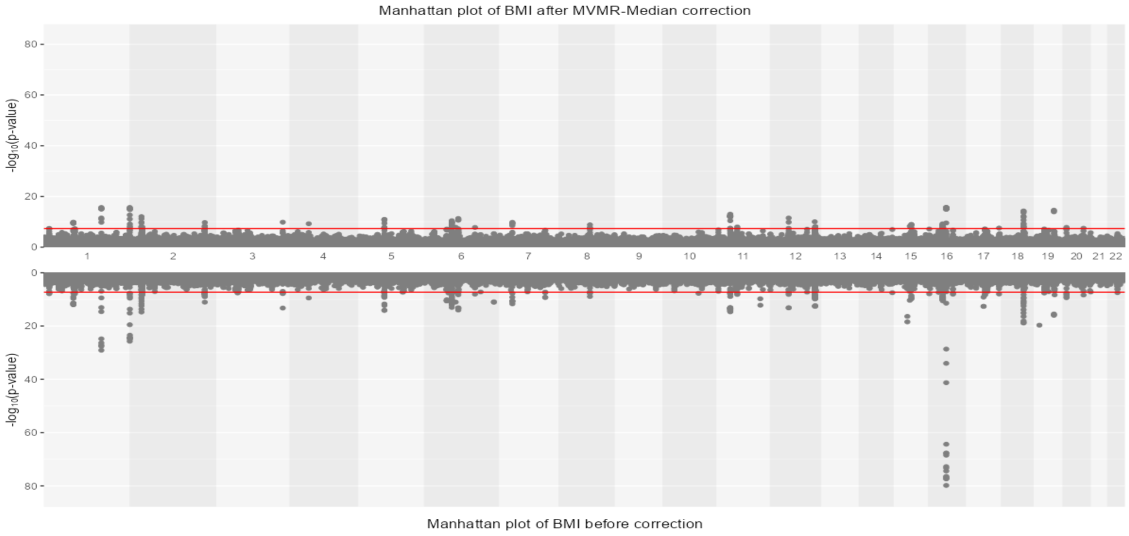

(e)

Fig BL. Manhattan plot of BMI before (upper panel) and after (lower panel) applying bias correction (in M_2). 4 metabolomic PCs are adjusted.

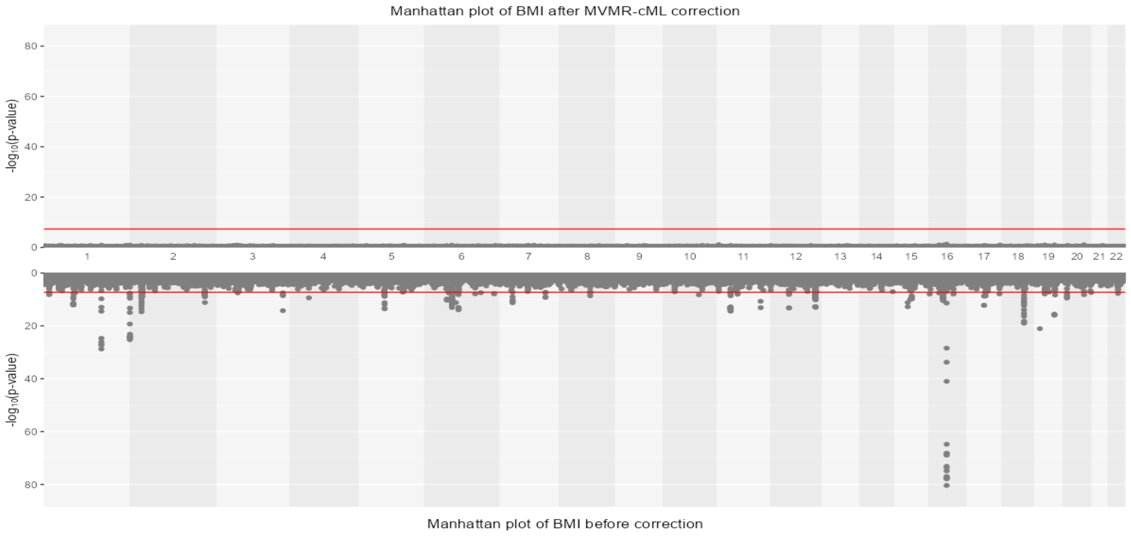
Manhattan plot of BMI after MVMR-cML correction
-log10(p-value)
0 20 40 60 80
1 2 3 4 5 6 7 8 9 10 11 12 13 14 15 16 17 18 19 20 21 22
Manhattan plot of BMI before correction

(a)

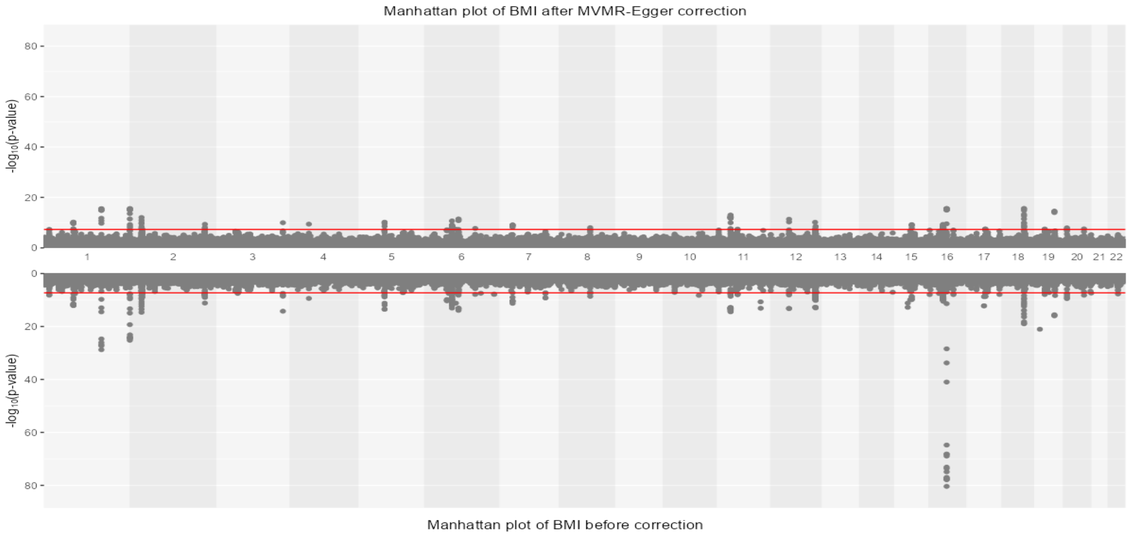
Manhattan plot of BMI after MVMR-Egger correction
-log10(p-value)
0 20 40 60 80
1 2 3 4 5 6 7 8 9 10 11 12 13 14 15 16 17 18 19 20 21 22
Manhattan plot of BMI before correction

(b)

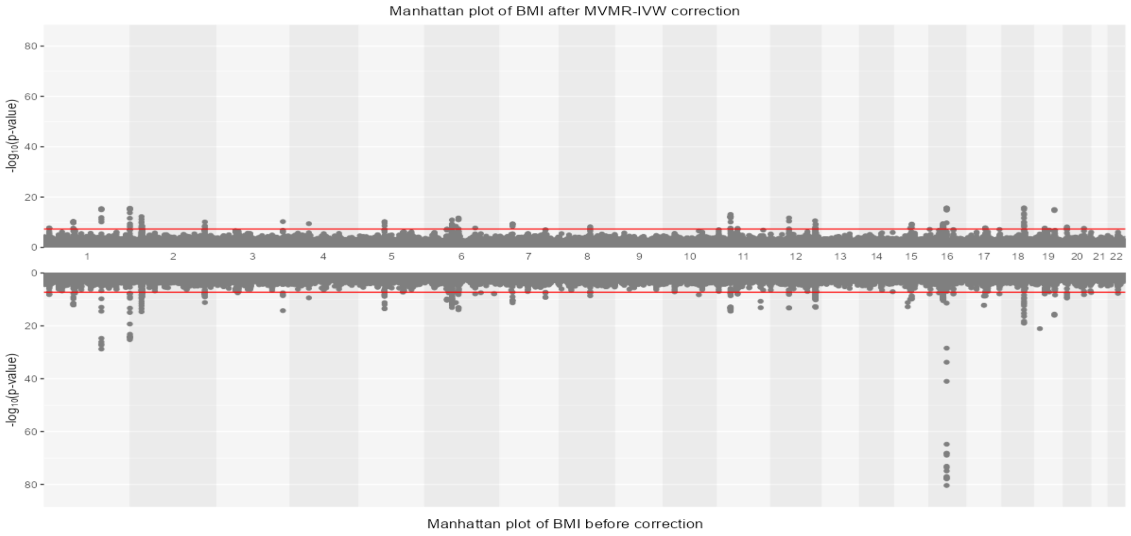
Manhattan plot of BMI after MVMR-IVW correction
-log10(p-value)
0 20 40 60 80
1 2 3 4 5 6 7 8 9 10 11 12 13 14 15 16 17 18 19 20 21 22
Manhattan plot of BMI before correction

(c)

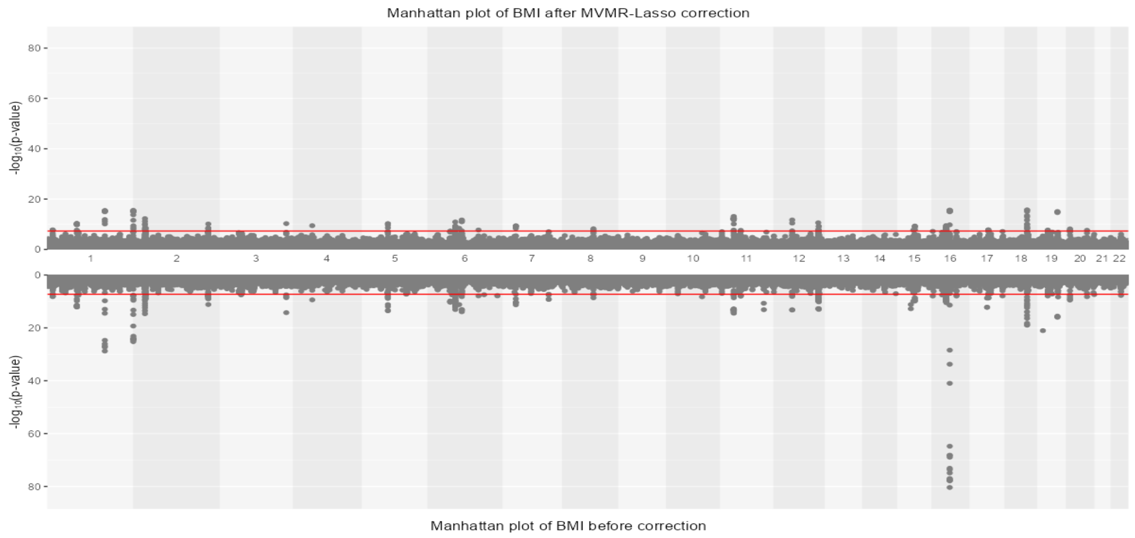

(d)

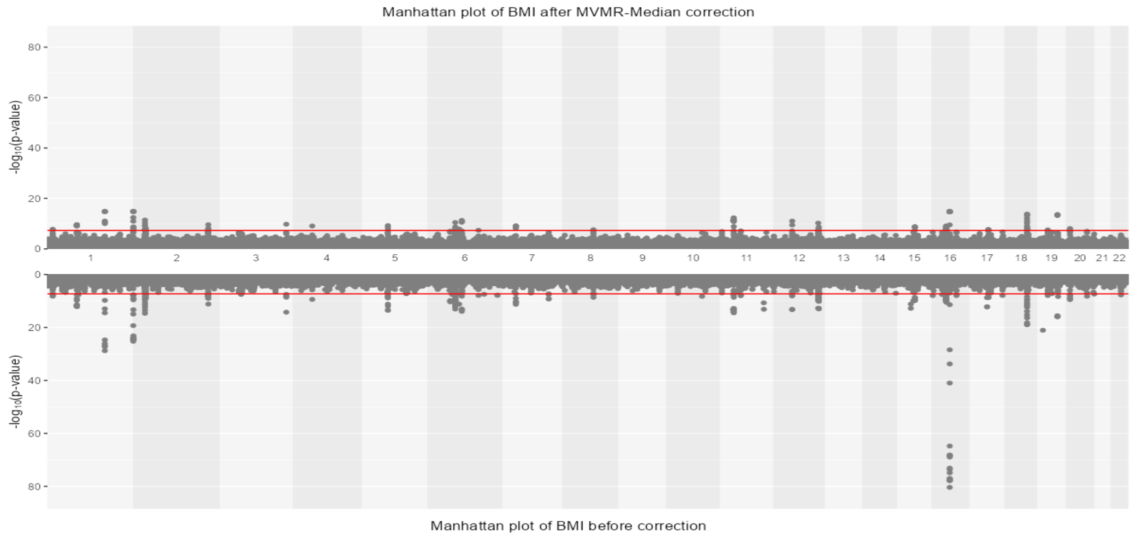

(e)

Fig BM. Manhattan plot of BMI before (upper panel) and after (lower panel) applying bias correction (in M_2). 5 metabolomic PCs are adjusted.

G.2.10 Comparison of Manhattan plots before and after apply different bias-correction methods on M_2, all 20 metabolomic PCs are used in M_2

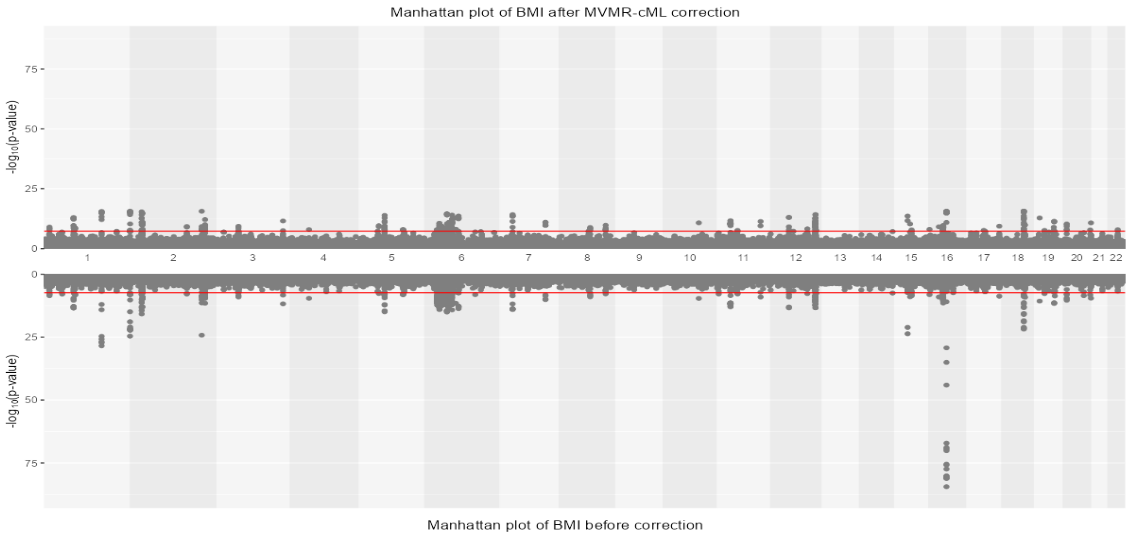

(a)

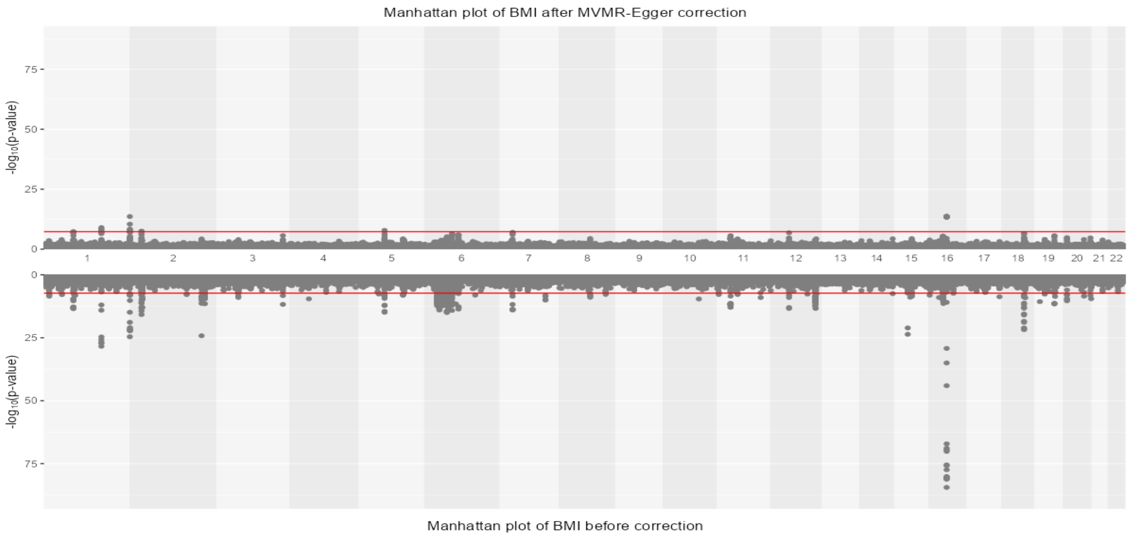

(b)

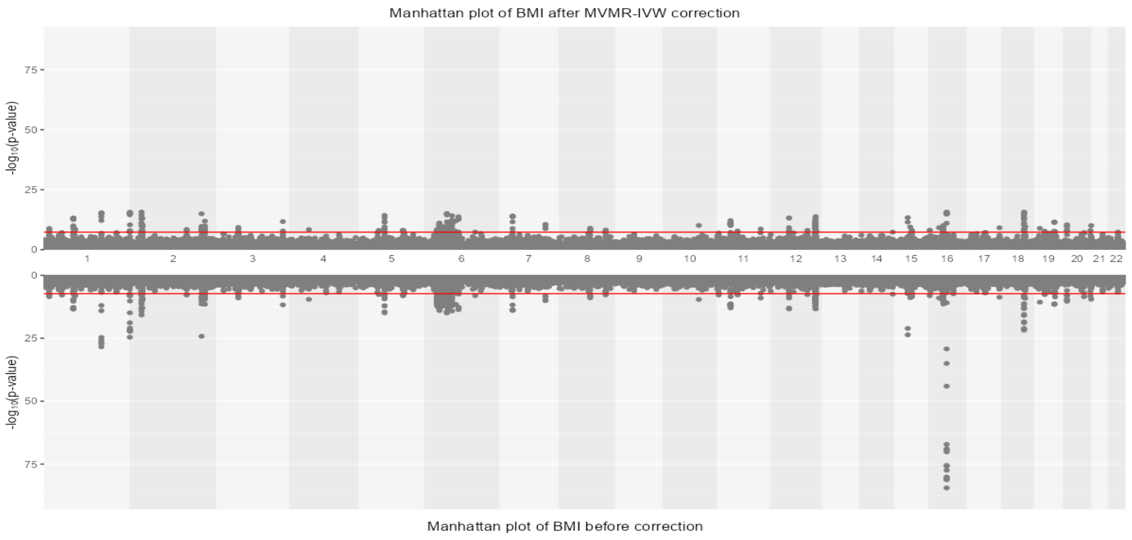

(c)

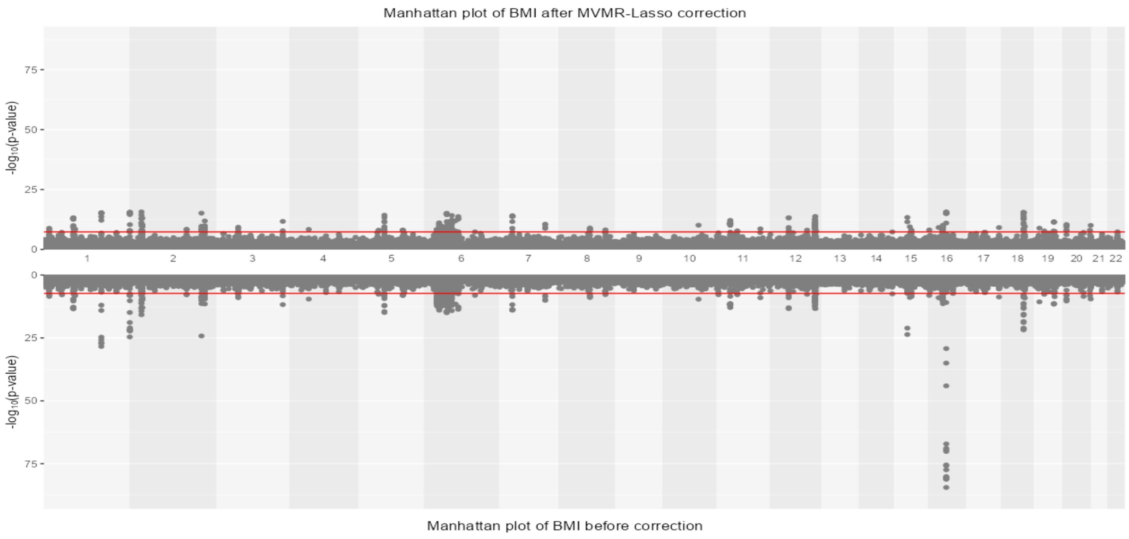
Manhattan plot of BMI after MVMR-Lasso correction
-log10(p-value)
75
50
25
0
1 2 3 4 5 6 7 8 9 10 11 12 13 14 15 16 17 18 19 20 21 22
-log10(p-value)
Manhattan plot of BMI before correction

(d)

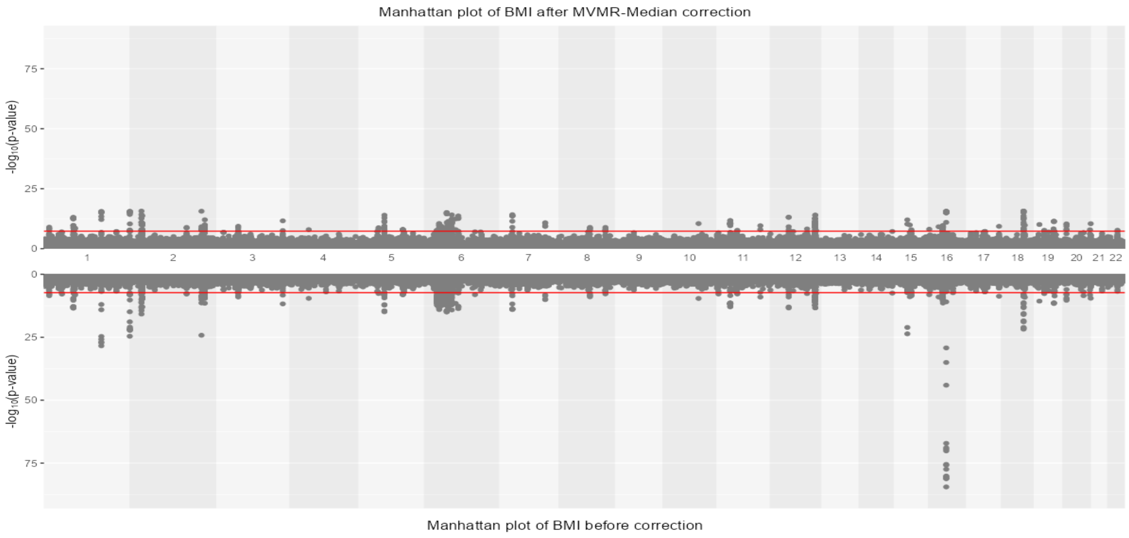
Manhattan plot of BMI after MVMR-Median correction
-log10(p-value)
75
50
25
0
1 2 3 4 5 6 7 8 9 10 11 12 13 14 15 16 17 18 19 20 21 22
-log10(p-value)
Manhattan plot of BMI before correction

(e)

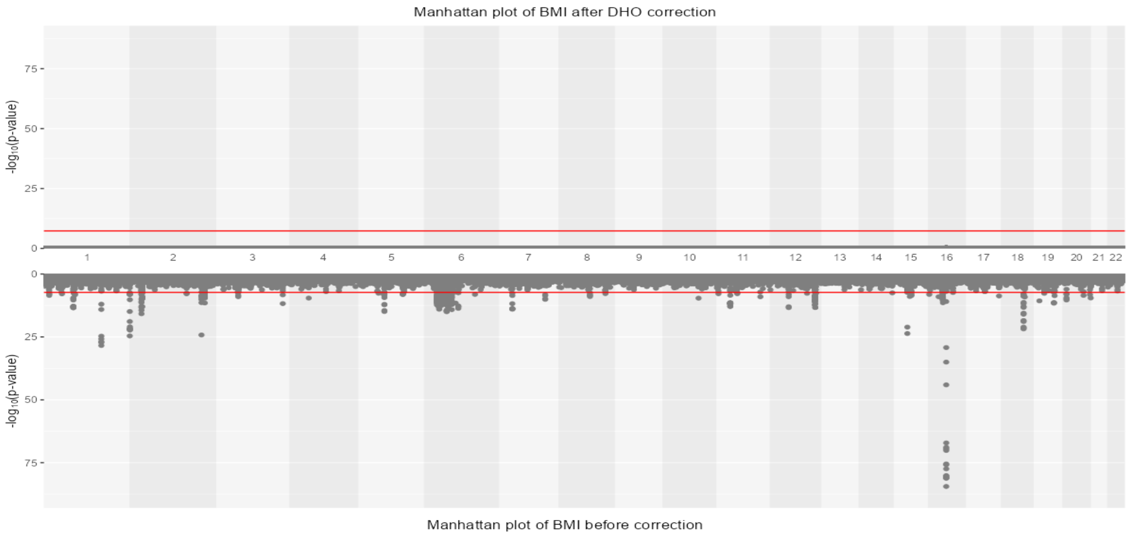
Manhattan plot of BMI after DHO correction
-log10(p-value)
75
50
25
0
1 2 3 4 5 6 7 8 9 10 11 12 13 14 15 16 17 18 19 20 21 22
-log10(p-value)
Manhattan plot of BMI before correction

(f)

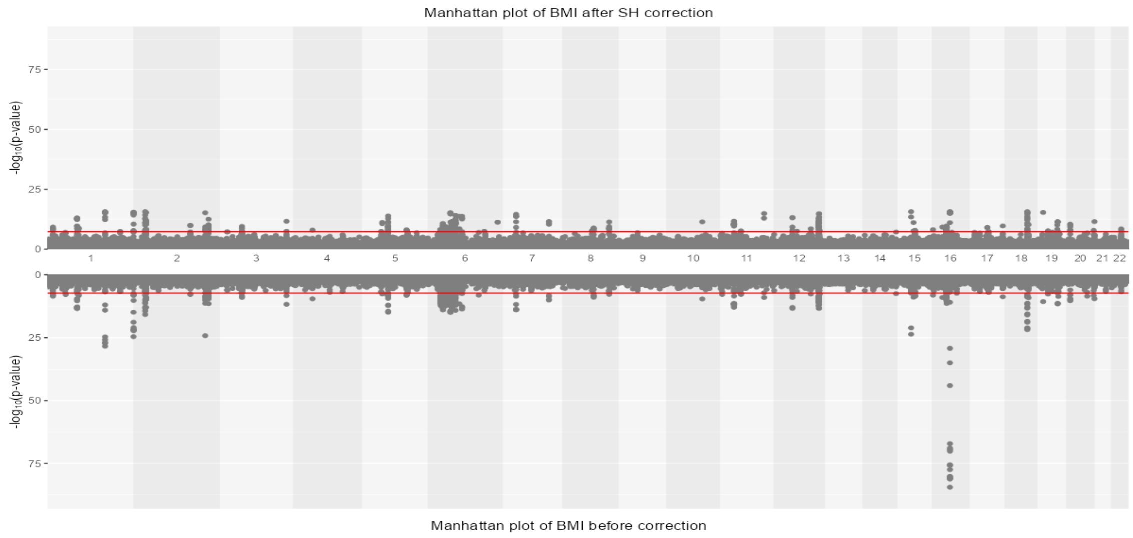

(g)

Fig BN. Manhattan plot of BMI before (upper panel) and after (lower panel) applying bias correction (in M_2). All 20 metabolomic PCs are used in M_2. 1 metabolomic PC is adjusted for bias correction.

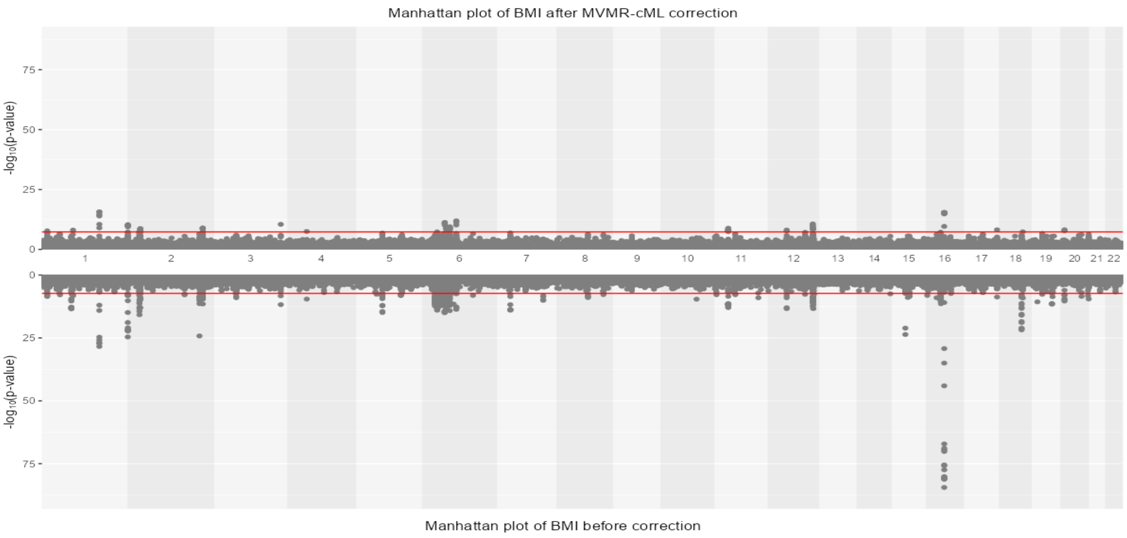
Manhattan plot of BMI after MVMR-cML correction
-log10(p-value)
Manhattan plot of BMI before correction

(a)

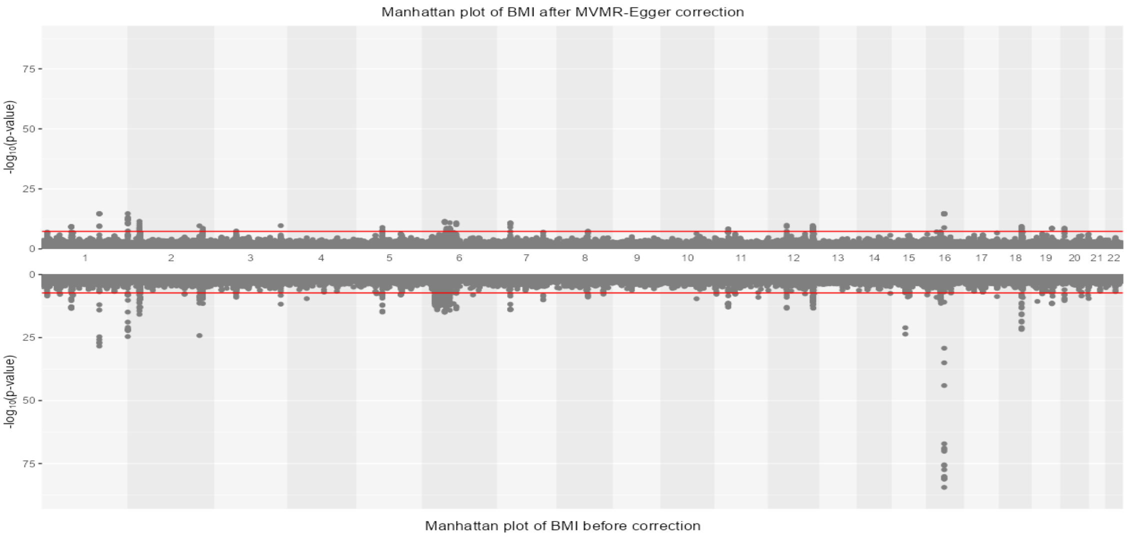
Manhattan plot of BMI after MVMR-Egger correction
-log10(p-value)
Manhattan plot of BMI before correction

(b)

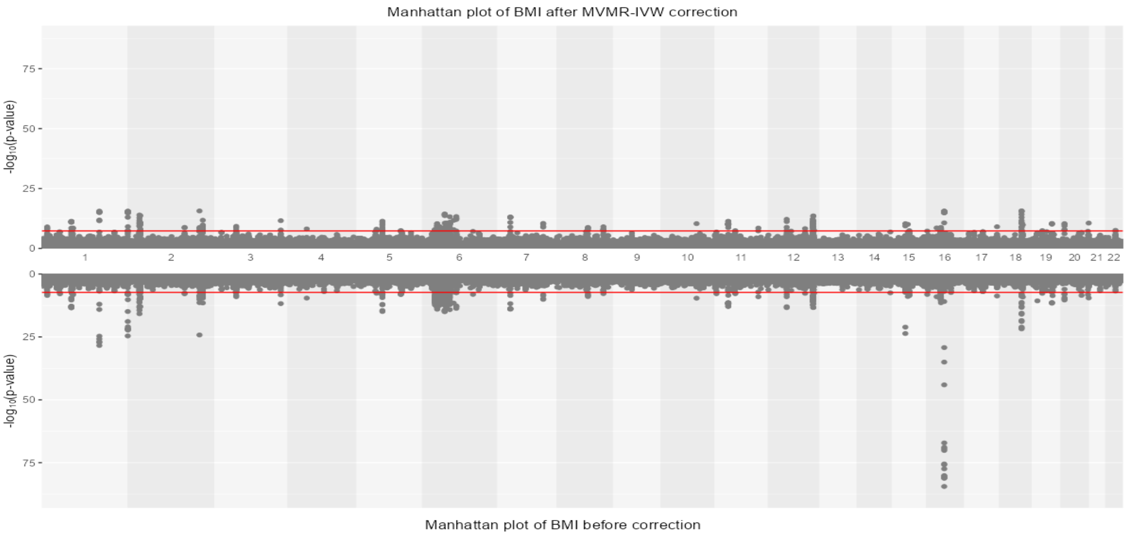
Manhattan plot of BMI after MVMR-IVW correction
-log10(p-value)
Manhattan plot of BMI before correction

(c)

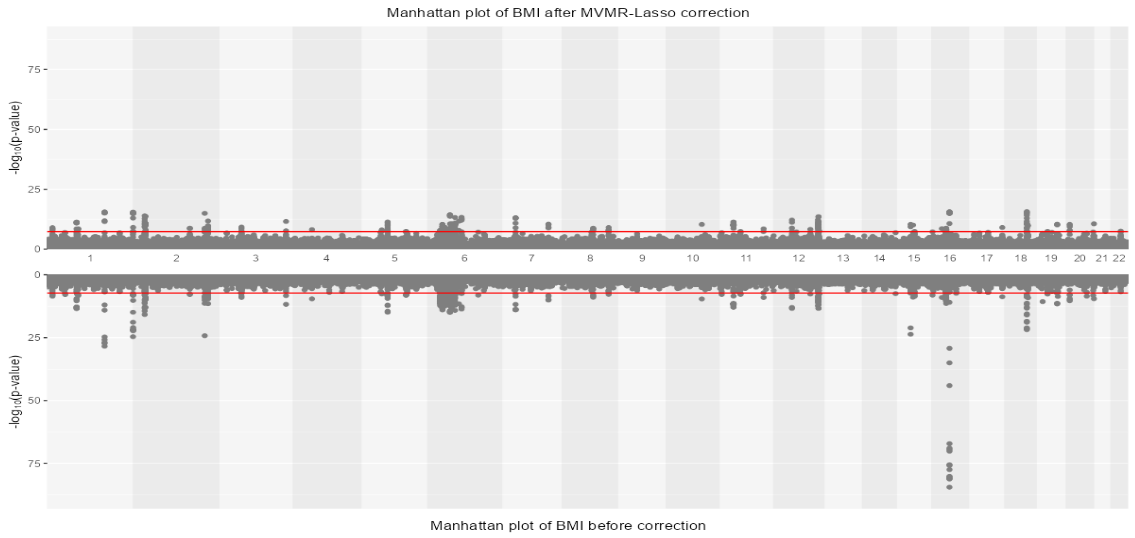

(d)

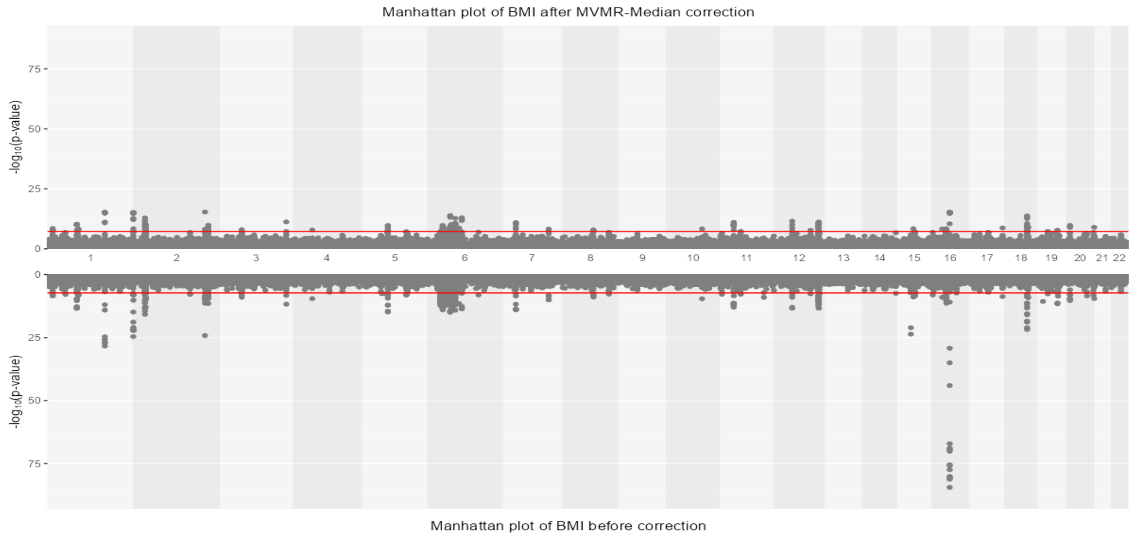

(e)

Fig BO. Manhattan plot of BMI before (upper panel) and after (lower panel) applying bias correction (in M_2). All 20 metabolomic PCs are used in M_2. 2 metabolomic PCs are adjusted for bias correction.

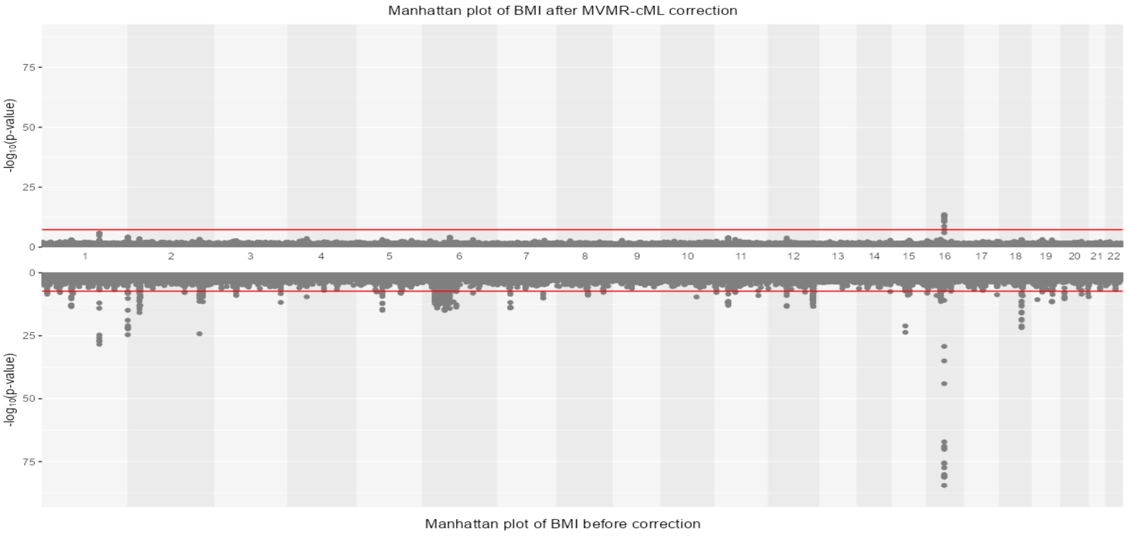
Manhattan plot of BMI after MVMR-cML correction
-log10(p-value)
Manhattan plot of BMI before correction

(a)

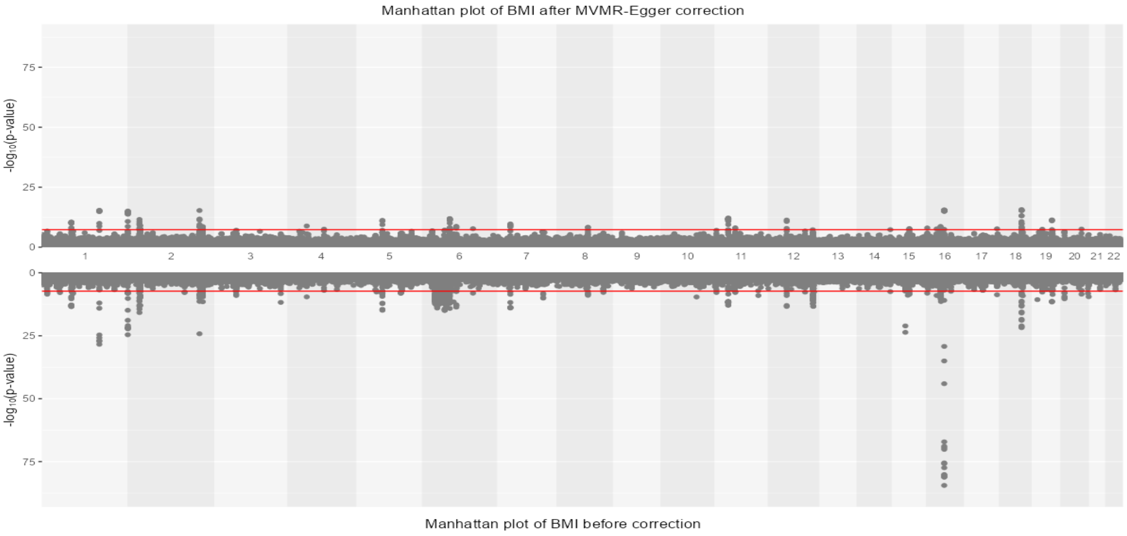
Manhattan plot of BMI after MVMR-Egger correction
-log10(p-value)
Manhattan plot of BMI before correction

(b)

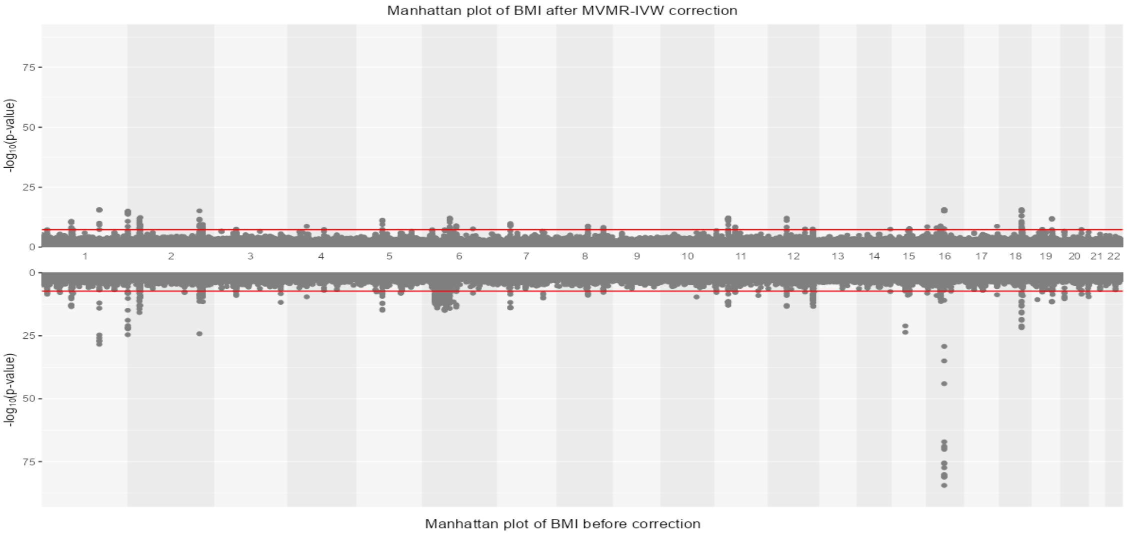
Manhattan plot of BMI after MVMR-IVW correction
-log10(p-value)
Manhattan plot of BMI before correction

(c)

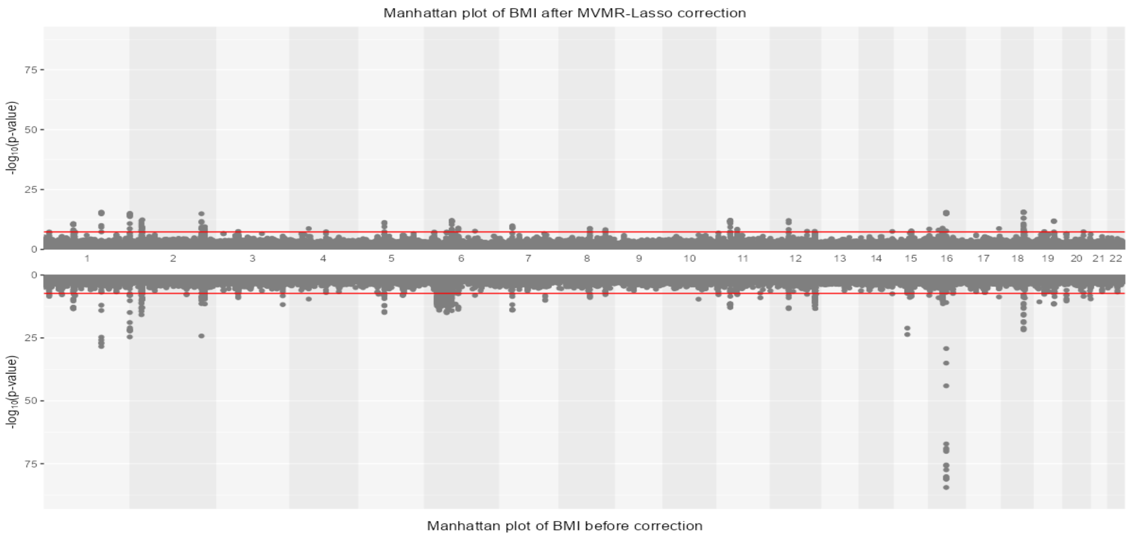

(d)

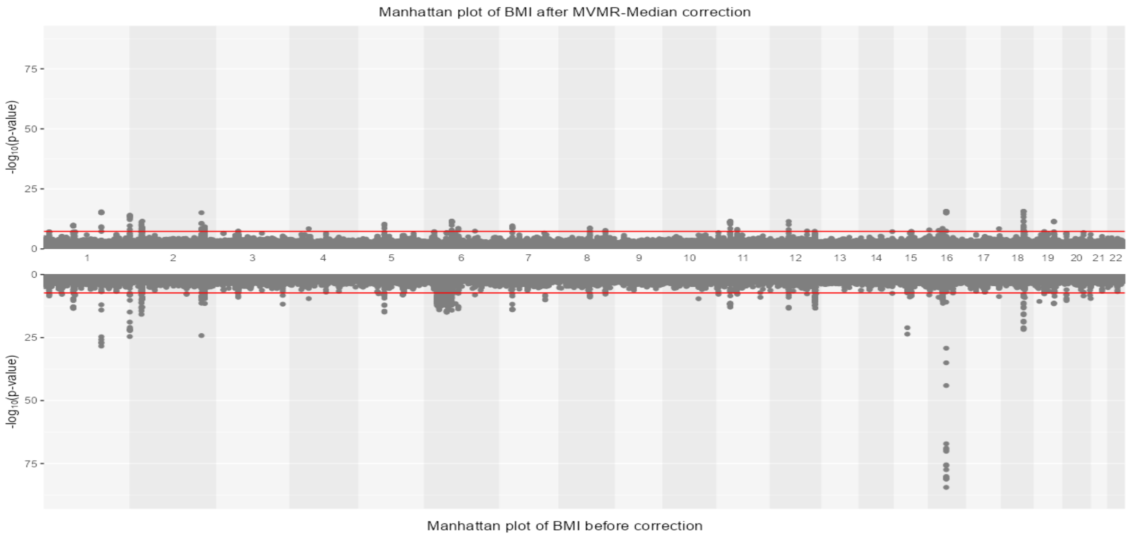

(e)

Fig BP. Manhattan plot of BMI before (upper panel) and after (lower panel) applying bias correction (in M_2). All 20 metabolomic PCs are used in M_2. 3 metabolomic PCs are adjusted for bias correction.

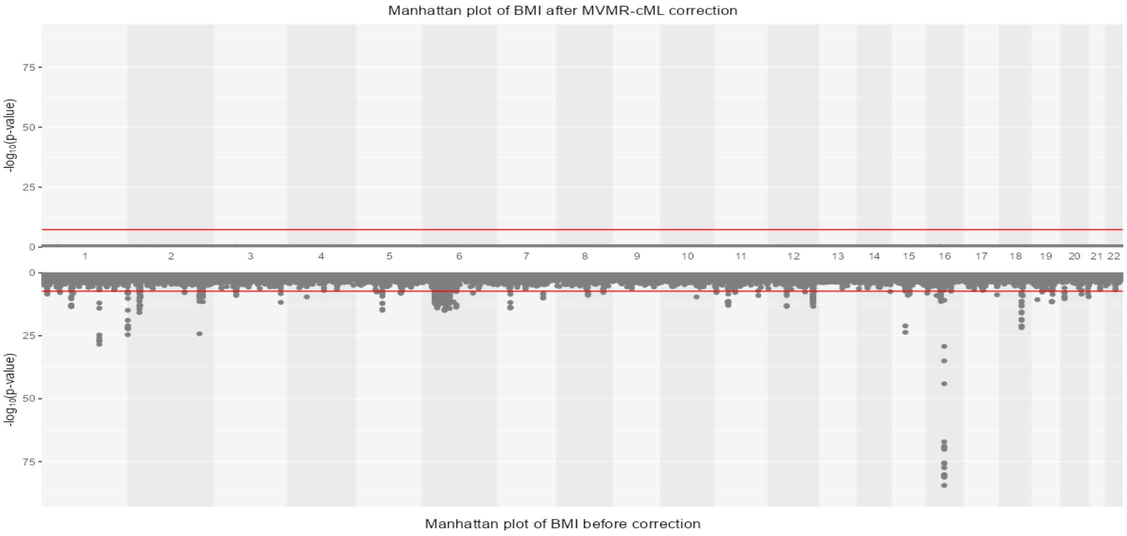
Manhattan plot of BMI after MVMR-cML correction
-log10(p-value)
75
50
25
0
1 2 3 4 5 6 7 8 9 10 11 12 13 14 15 16 17 18 19 20 21 22
0
25
50
75
-log10(p-value)
Manhattan plot of BMI before correction

(a)

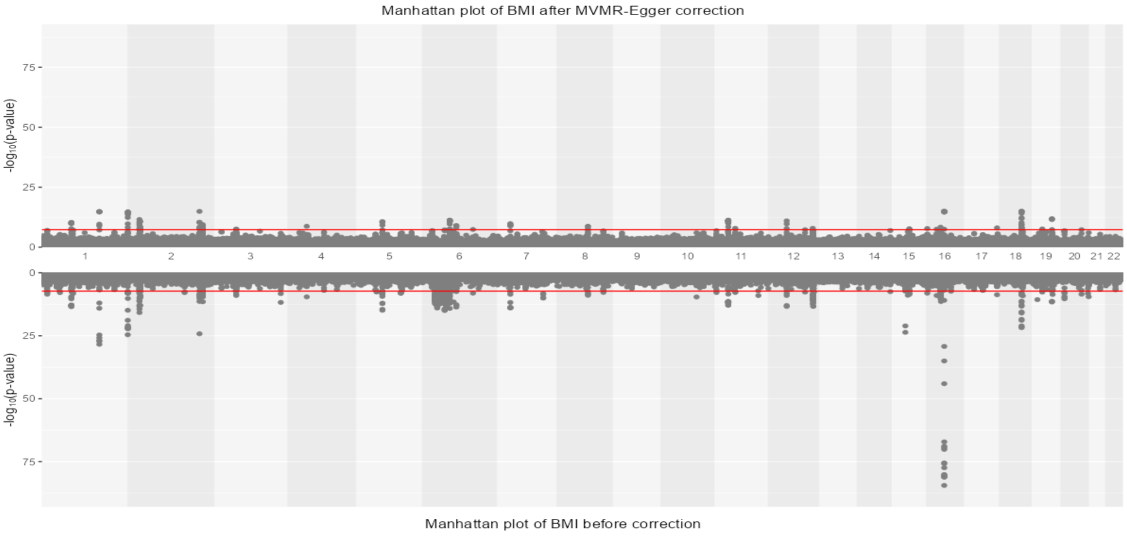
Manhattan plot of BMI after MVMR-Egger correction
-log10(p-value)
75
50
25
0
1 2 3 4 5 6 7 8 9 10 11 12 13 14 15 16 17 18 19 20 21 22
0
25
50
75
-log10(p-value)
Manhattan plot of BMI before correction

(b)

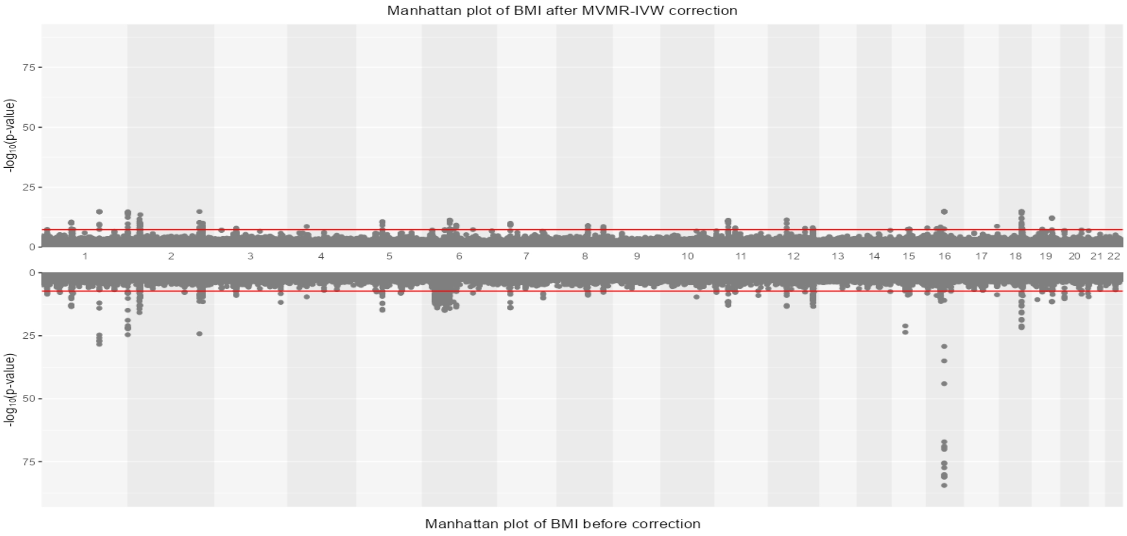
Manhattan plot of BMI after MVMR-IVW correction
-log10(p-value)
75
50
25
0
1 2 3 4 5 6 7 8 9 10 11 12 13 14 15 16 17 18 19 20 21 22
0
25
50
75
-log10(p-value)
Manhattan plot of BMI before correction

(c)

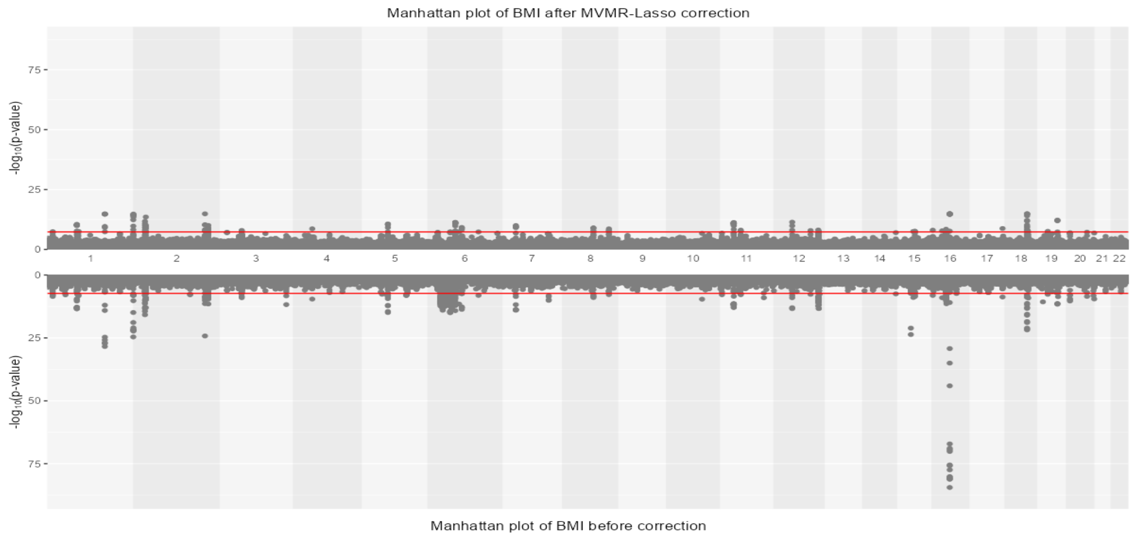

(d)

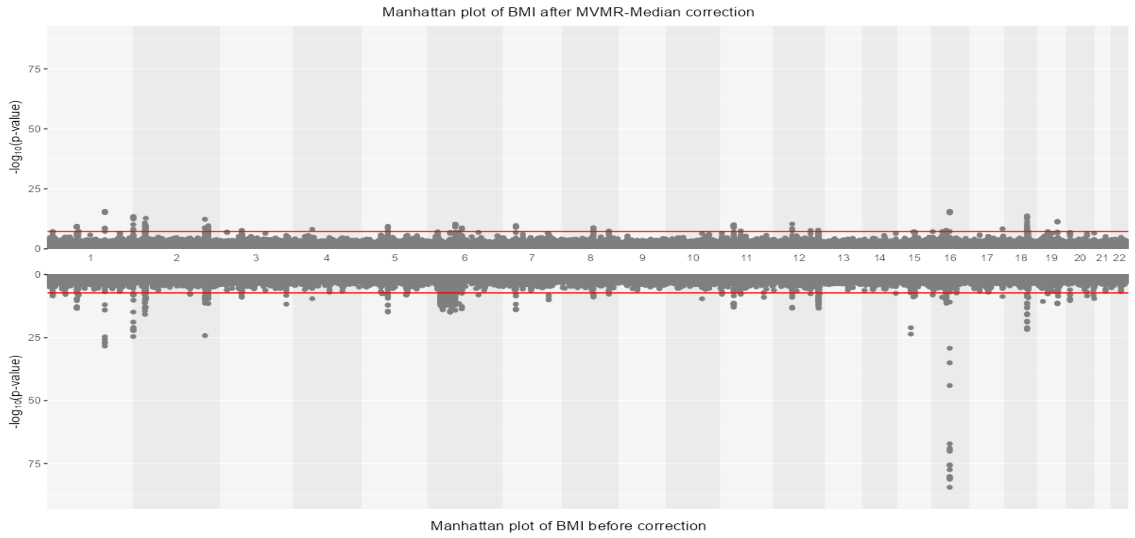

(e)

Fig BQ. Manhattan plot of BMI before (upper panel) and after (lower panel) applying bias correction (in M_2). All 20 metabolomic PCs are used in M_2. 4 metabolomic PCs are adjusted for bias correction.

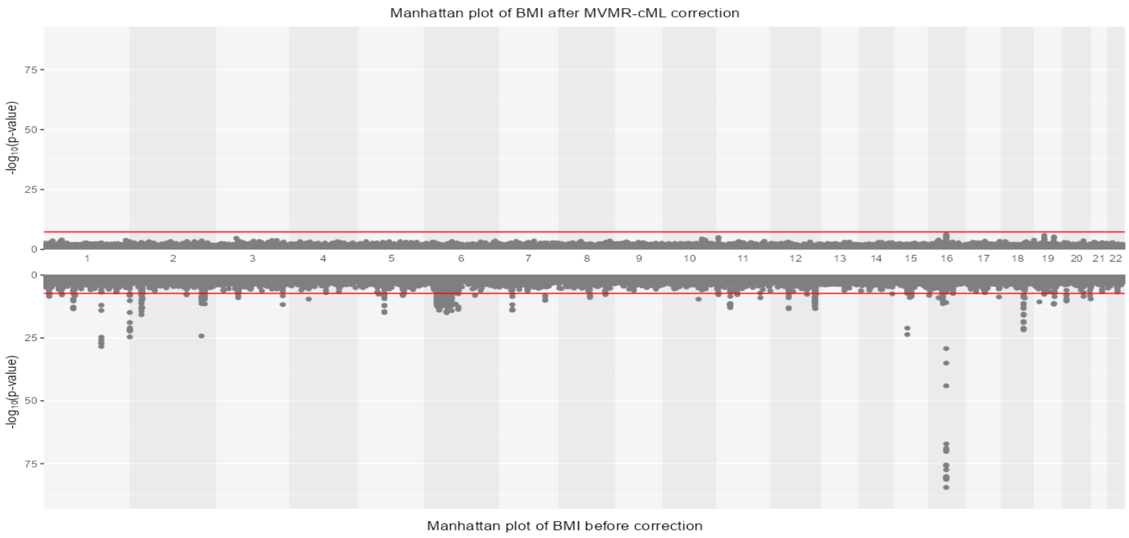
Manhattan plot of BMI after MVMR-cML correction
-log10(p-value)
Manhattan plot of BMI before correction

(a)

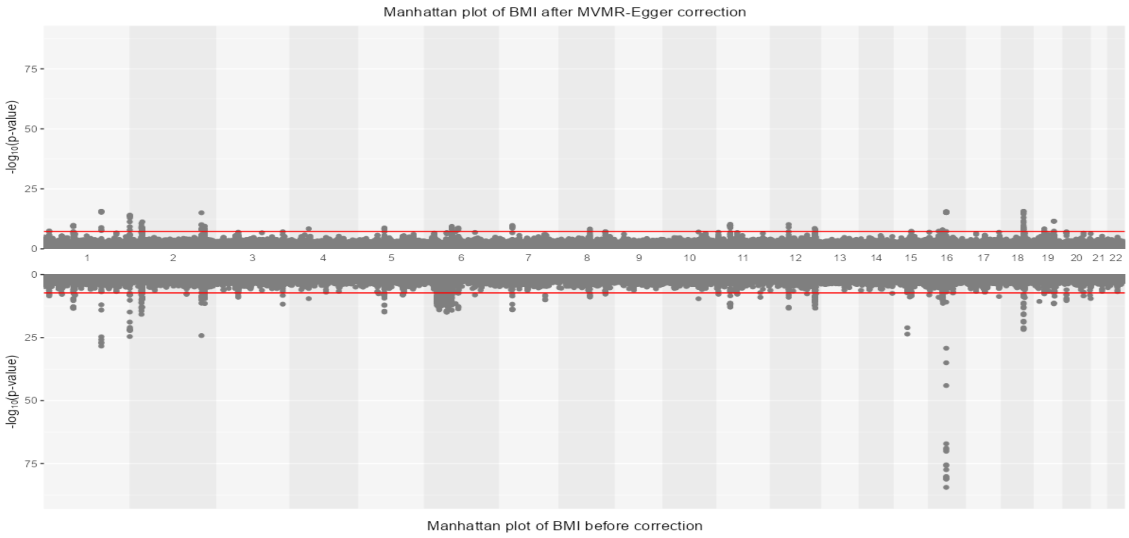
Manhattan plot of BMI after MVMR-Egger correction
-log10(p-value)
Manhattan plot of BMI before correction

(b)

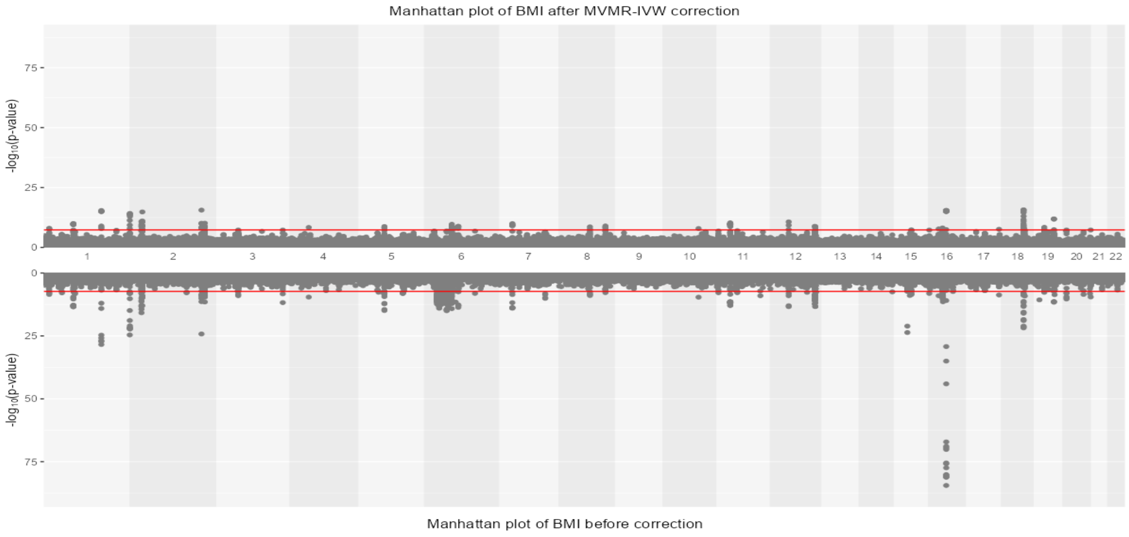
Manhattan plot of BMI after MVMR-IVW correction
-log10(p-value)
Manhattan plot of BMI before correction

(c)

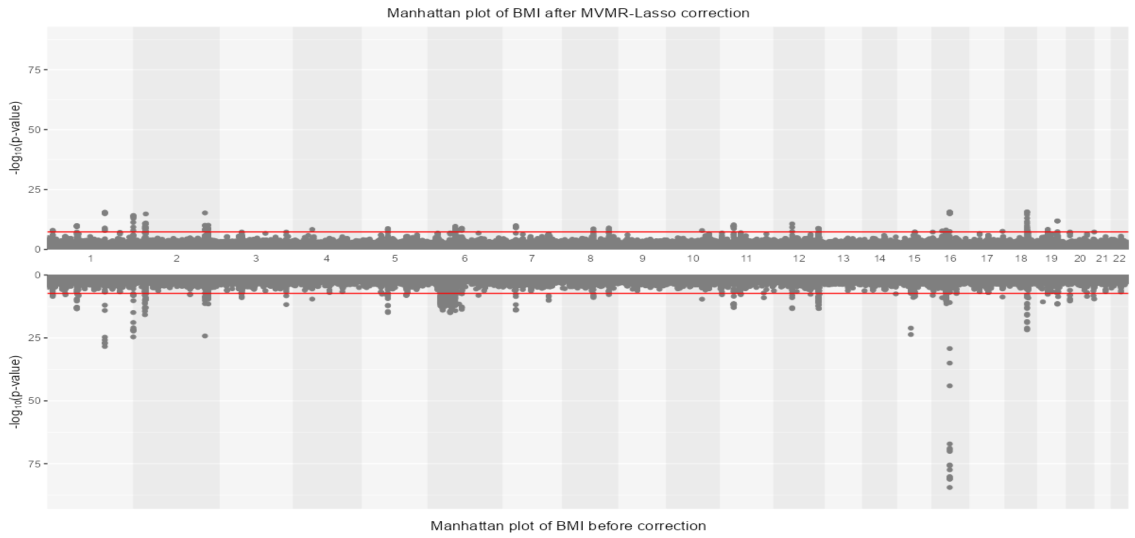

(d)

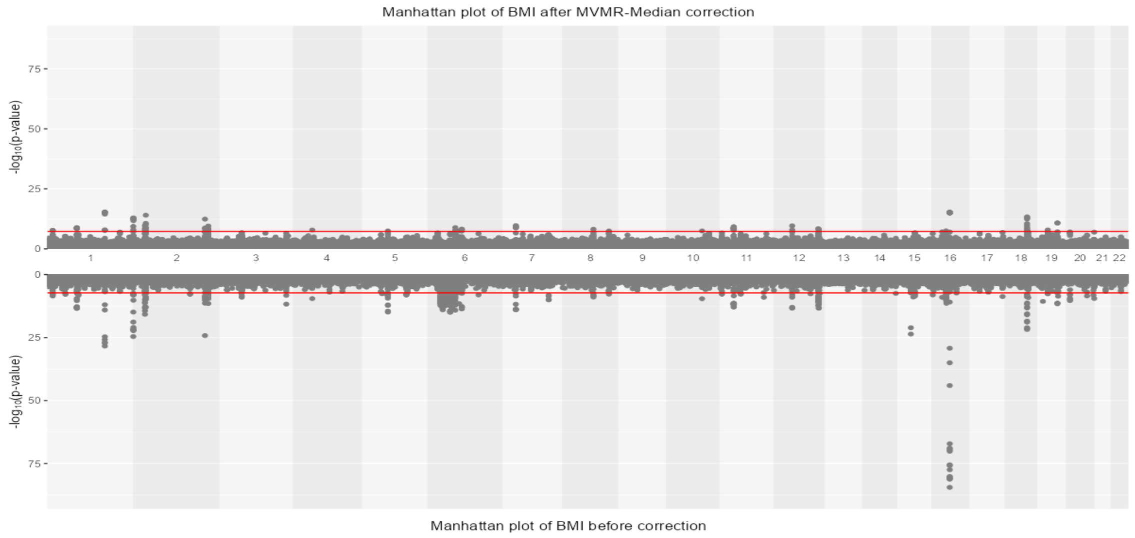

(e)

Fig BR. Manhattan plot of BMI before (upper panel) and after (lower panel) applying bias correction (in M_2). All 20 metabolomic PCs are used in M_2. 5 metabolomic PCs are adjusted for bias correction.

G.2.11 Comparison of QQ plots before and after apply different bias-correction methods on M_1

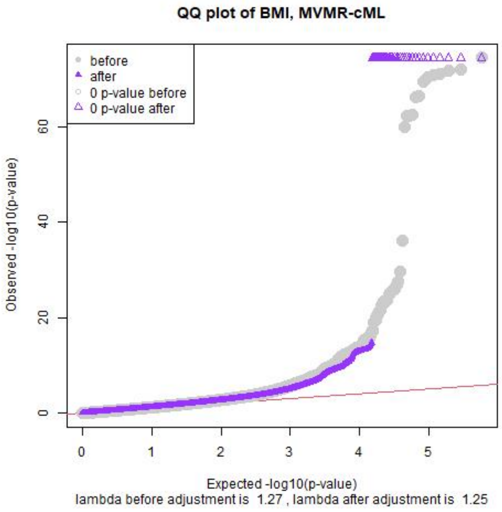

(a)

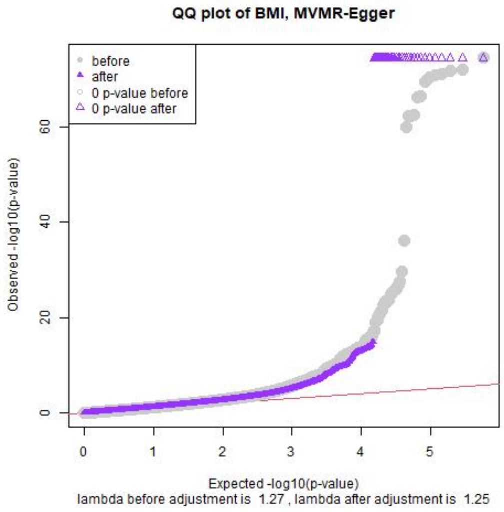

(b)

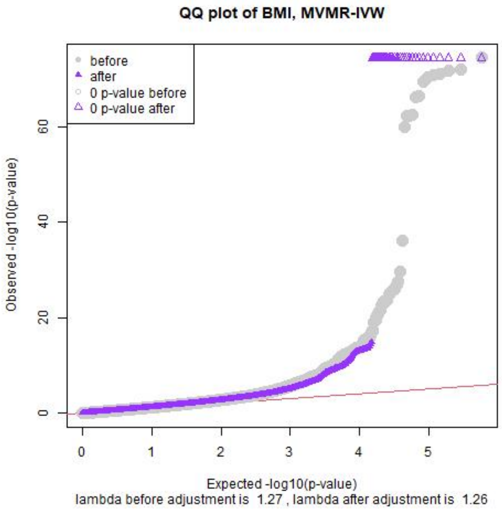

(c)

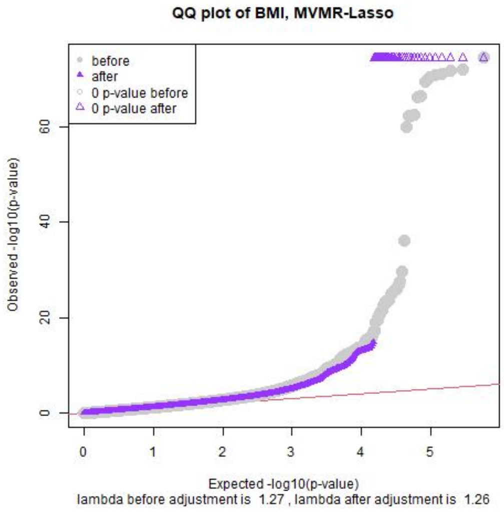

(d)

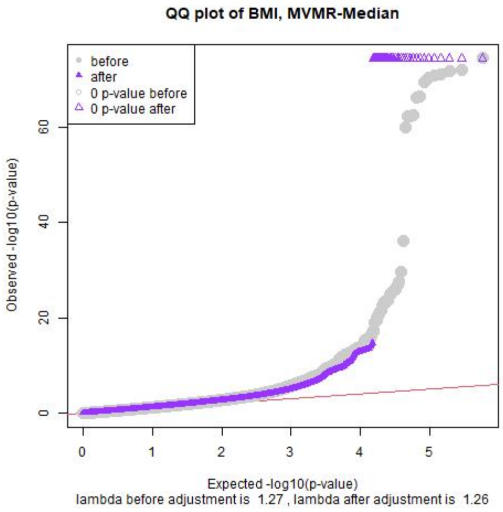

(e)

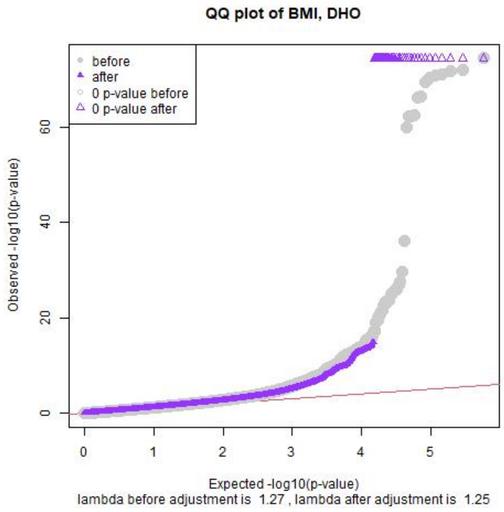

(f)

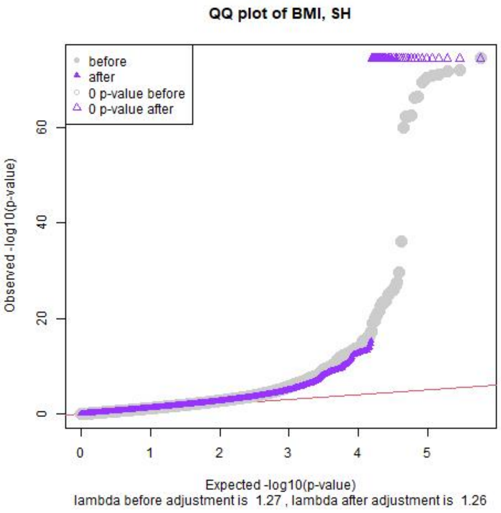

(g)

Fig BS. QQ plots of BMI (of M_1) before and after applying bias correction. 1 metabololic PC is adjusted. In the legends, "before" means the result before correction, "after" means the result after correction. "0 p-value before" represents the SNPs having a p-value of 0 before correction. "0 p-value after" represents the SNPs having a p-value of 0 after correction. The SNPs having p-values of 0 before or after correction are truncated at the top of plots.

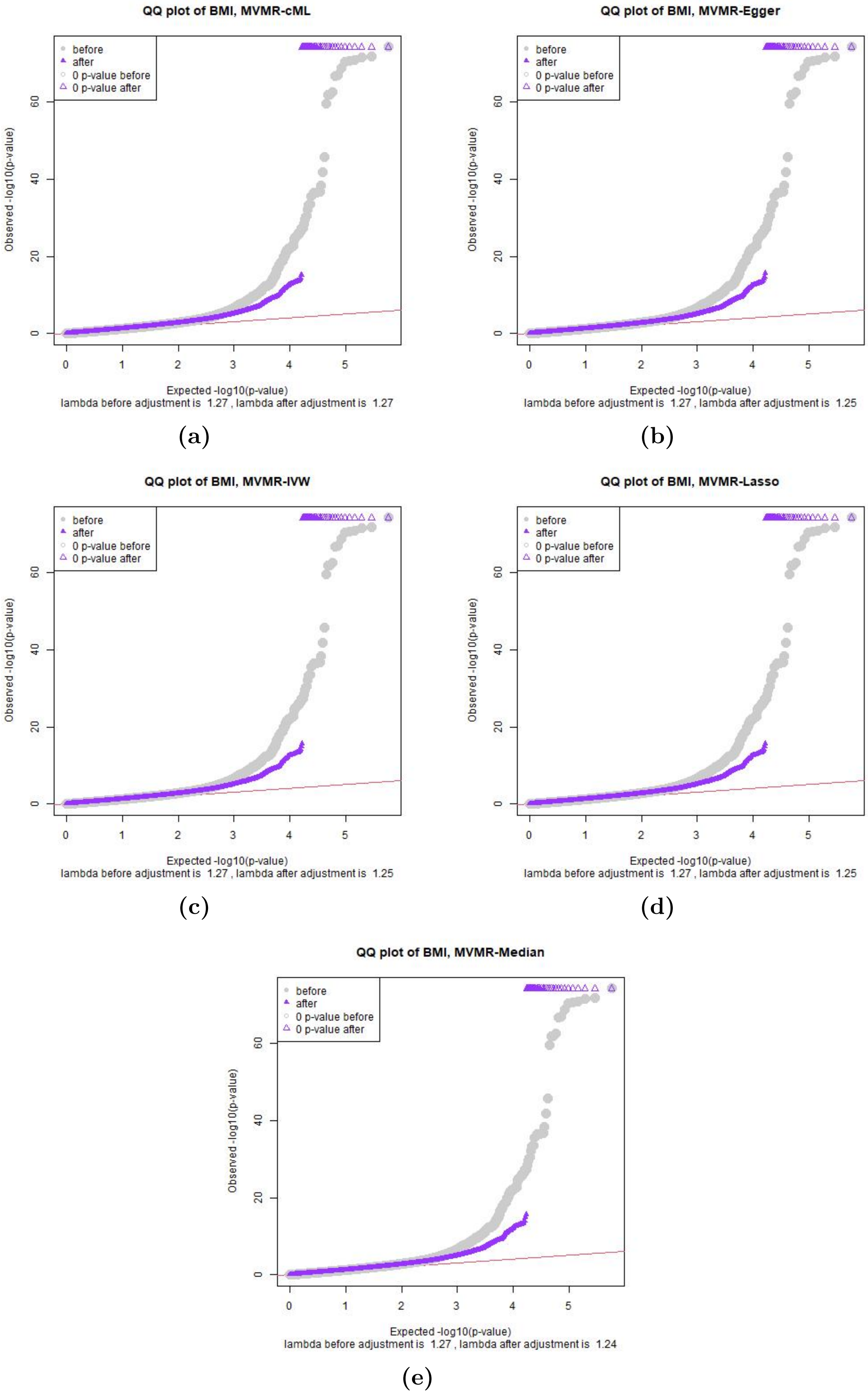

Fig BT. QQ plots of BMI (of M_1) before and after applying bias correction. 2 metabololic PCs are adjusted. In the legends, “before” means the result before correction, “after” means the result after correction. “0 p-value before” represents the SNPs having a p-value of 0 before correction. “0 p-value after” represents the SNPs having a p-value of 0 after correction. The SNPs having p-values of 0 before or after correction are truncated at the top of plots.

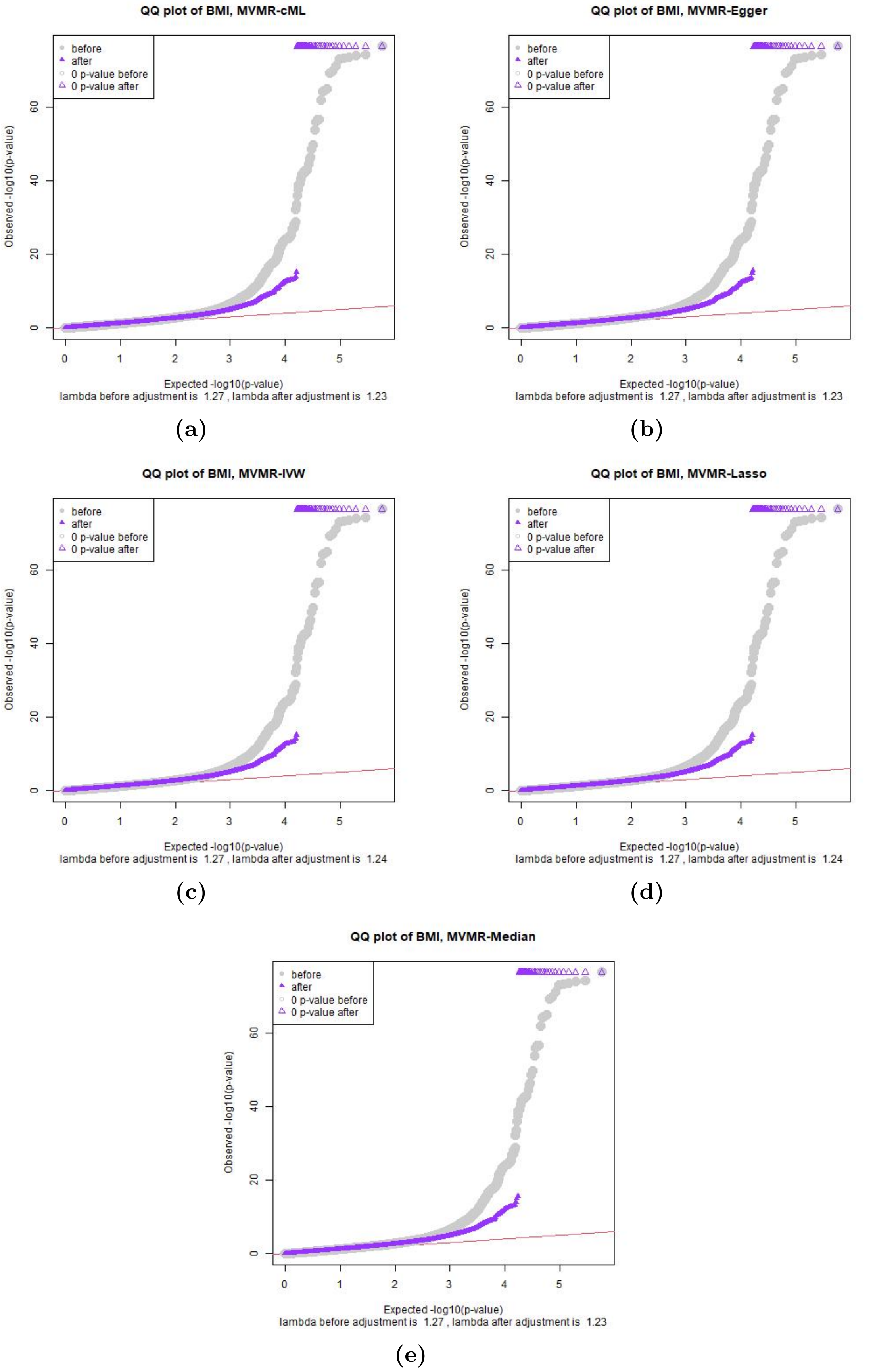

Fig BU. QQ plots of BMI (of M_1) before and after applying bias correction. 3 metabololic PCs are adjusted. In the legends, "before" means the result before correction, "after" means the result after correction. "0 p-value before" represents the SNPs having a p-value of 0 before correction. "0 p-value after" represents the SNPs having a p-value of 0 after correction. The SNPs having p-values of 0 before or after correction are truncated at the top of plots.

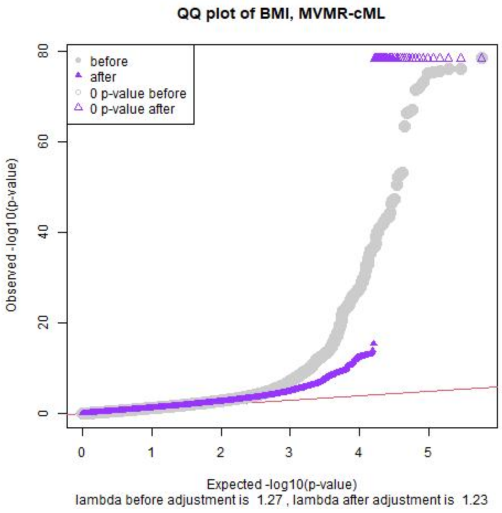

(a)

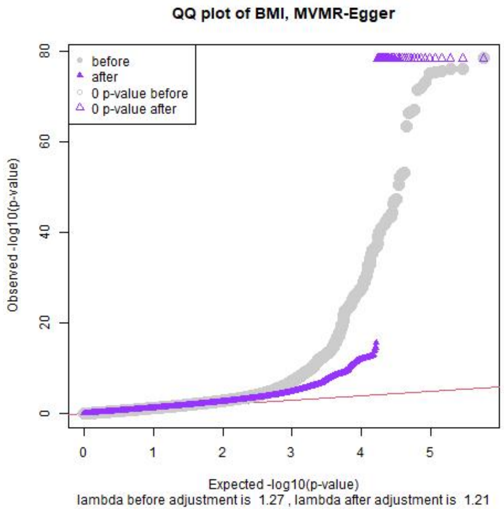

(b)

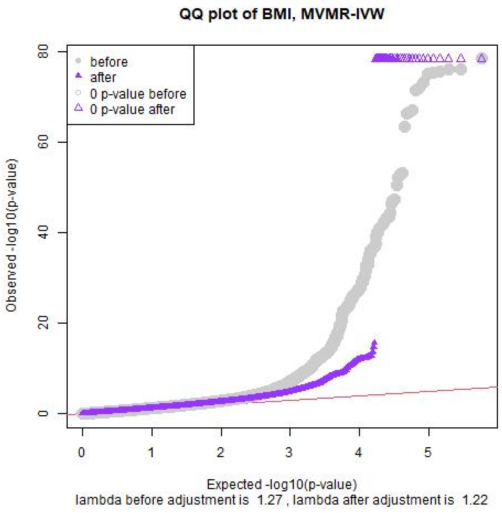

(c)

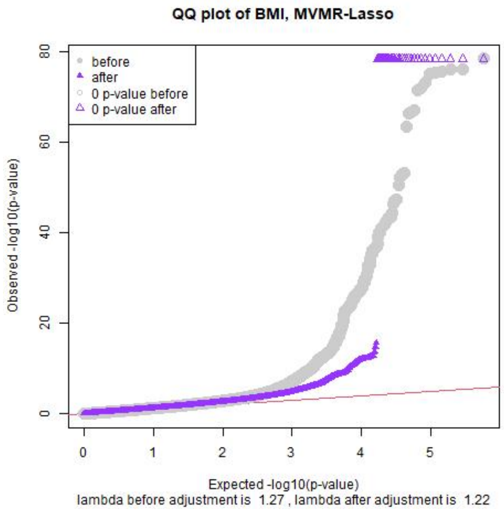

(d)

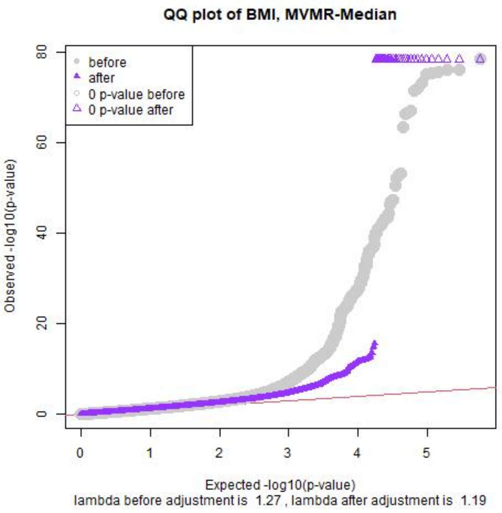

(e)

Fig BV. QQ plots of BMI (of M_1) before and after applying bias correction. 4 metabololic PCs are adjusted. In the legends, "before" means the result before correction, "after" means the result after correction. "0 p-value before" represents the SNPs having a p-value of 0 before correction. "0 p-value after" represents the SNPs having a p-value of 0 after correction. The SNPs having p-values of 0 before or after correction are truncated at the top of plots.

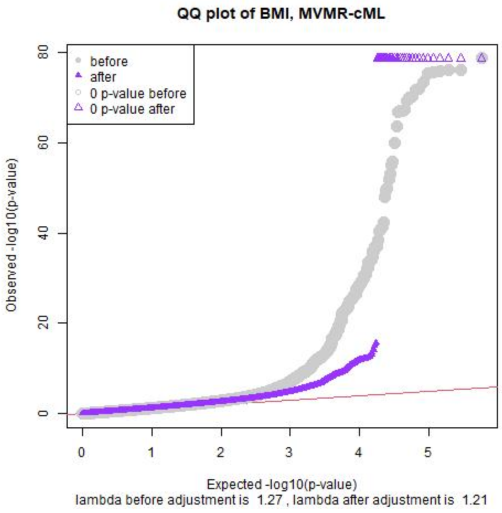

(a)

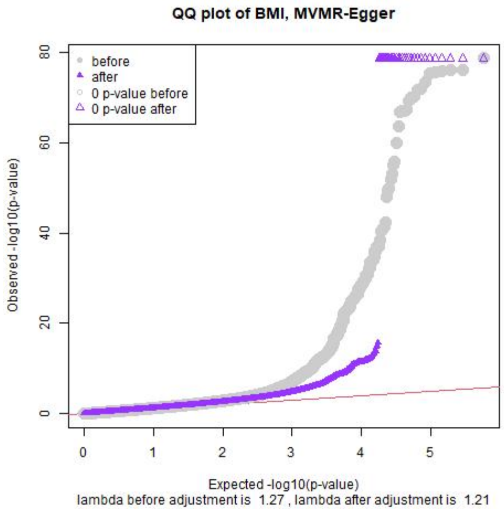

(b)

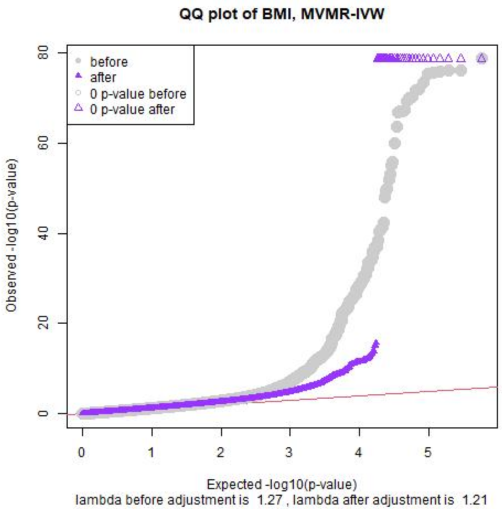

(c)

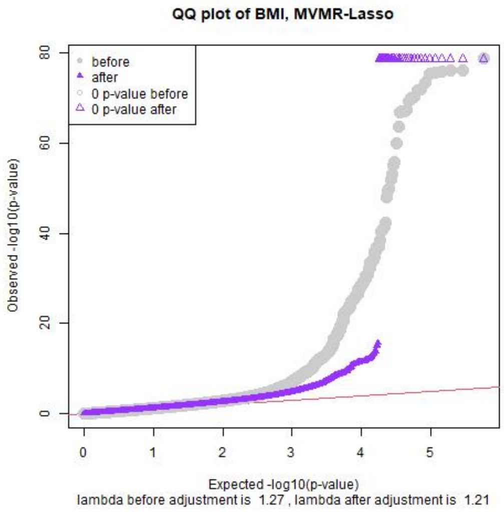

(d)

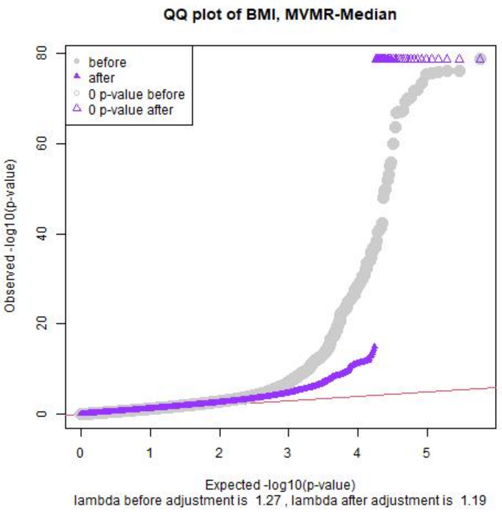

(e)

Fig BW. QQ plots of BMI (of M_1) before and after applying bias correction. 5 metabololic PCs are adjusted. In the legends, “before” means the result before correction, “after” means the result after correction. “0 p-value before” represents the SNPs having a p-value of 0 before correction. “0 p-value after” represents the SNPs having a p-value of 0 after correction. The SNPs having p-values of 0 before or after correction are truncated at the top of plots.

G.2.12 Comparison of QQ plots before and after apply different bias-correction methods on M_1, all 20 metabolomic PCs are used in M_1

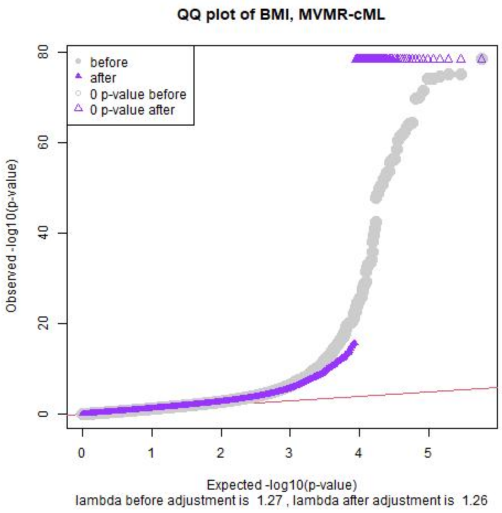

(a)

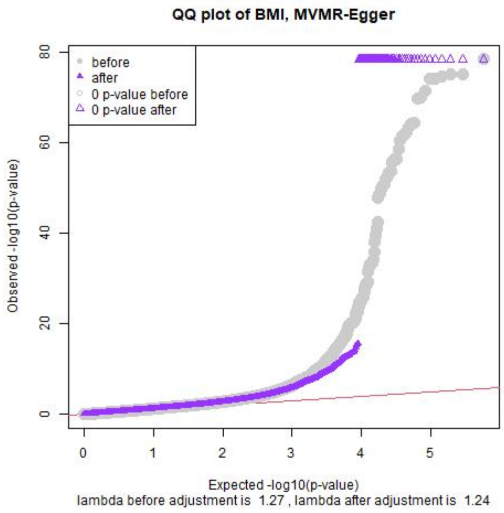

(b)

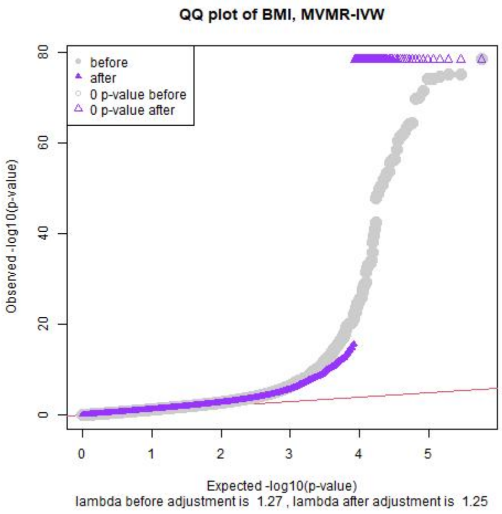

(c)

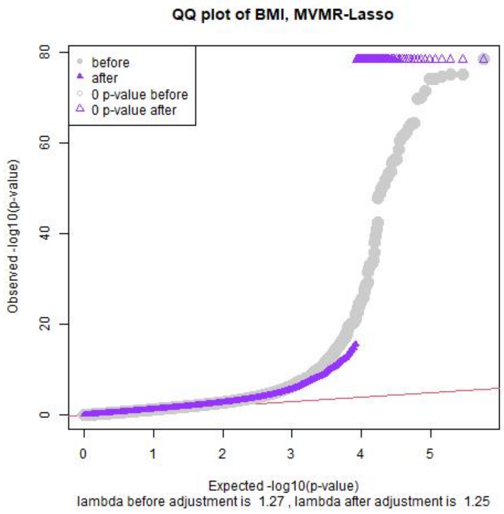

(d)

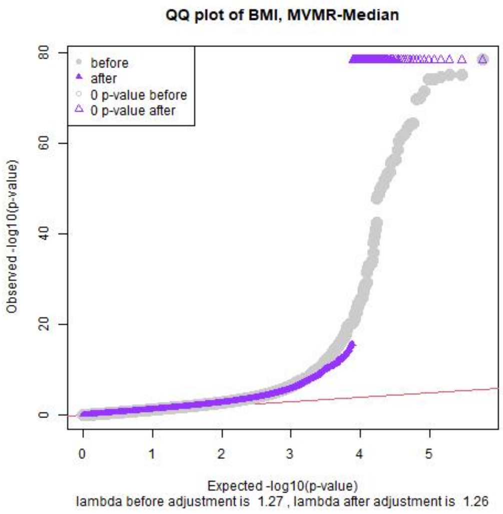

(e)

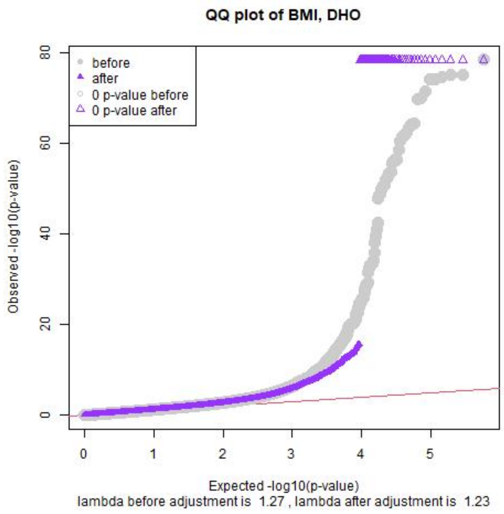

(f)

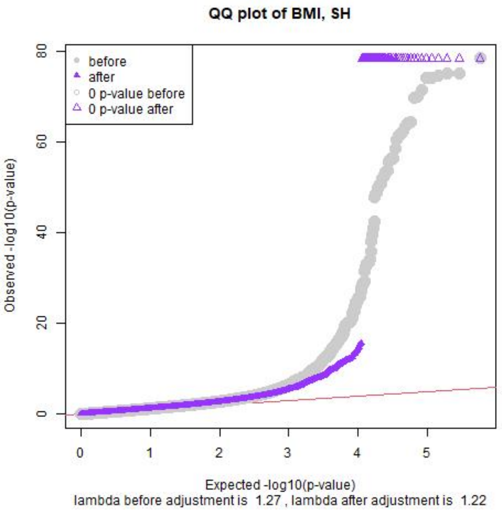

(g)

Fig BX. QQ plots of BMI (of M_1) before and after applying bias correction. All 20 metabolomic PCs are used in M_1. 1 metabololic PC is adjusted for bias correction. In the legends, "before" means the result before correction, "after" means the result after correction. "0 p-value before" represents the SNPs having a p-value of 0 before correction. "0 p-value after" represents the SNPs having a p-value of 0 after correction. The SNPs having p-values of 0 before or after correction are truncated at the top of plots.

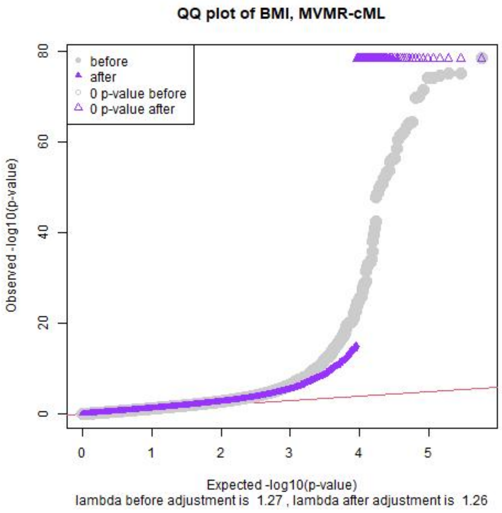

(a)

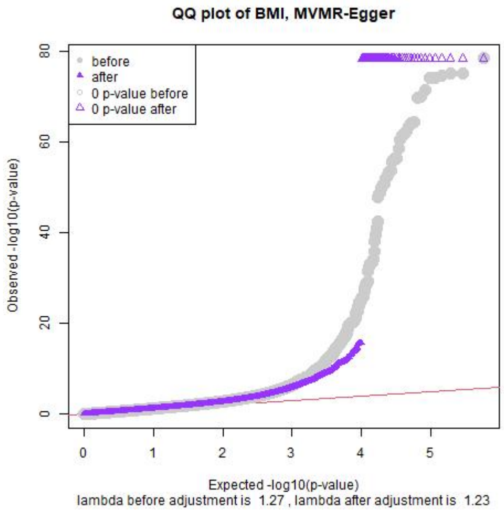

(b)

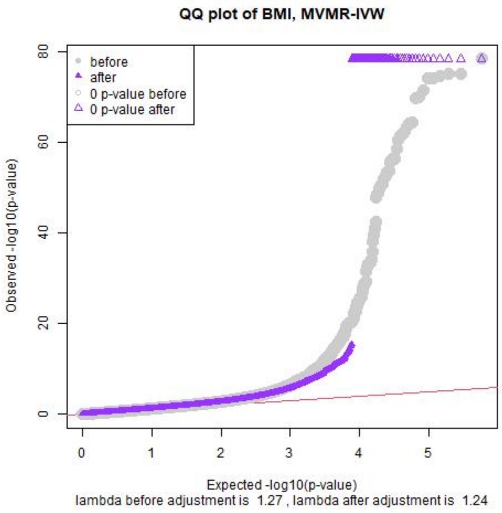

(c)

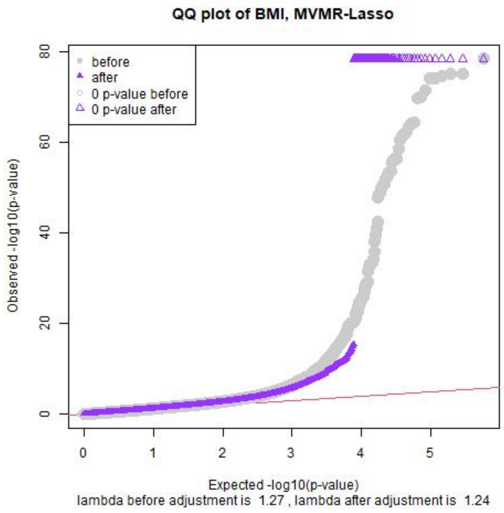

(d)

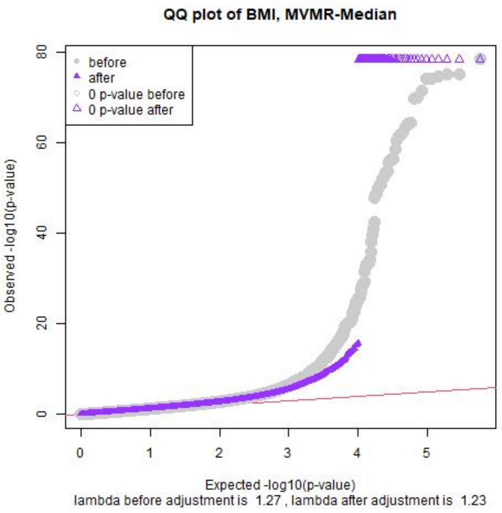

(e)

Fig BY. QQ plots of BMI (of M_1) before and after applying bias correction. All 20 metabolomic PCs are used in M_1. 2 metabololic PCs are adjusted for bias correction. In the legends, "before" means the result before correction, "after" means the result after correction. "0 p-value before" represents the SNPs having a p-value of 0 before correction. "0 p-value after" represents the SNPs having a p-value of 0 after correction. The SNPs having p-values of 0 before or after correction are truncated at the top of plots.

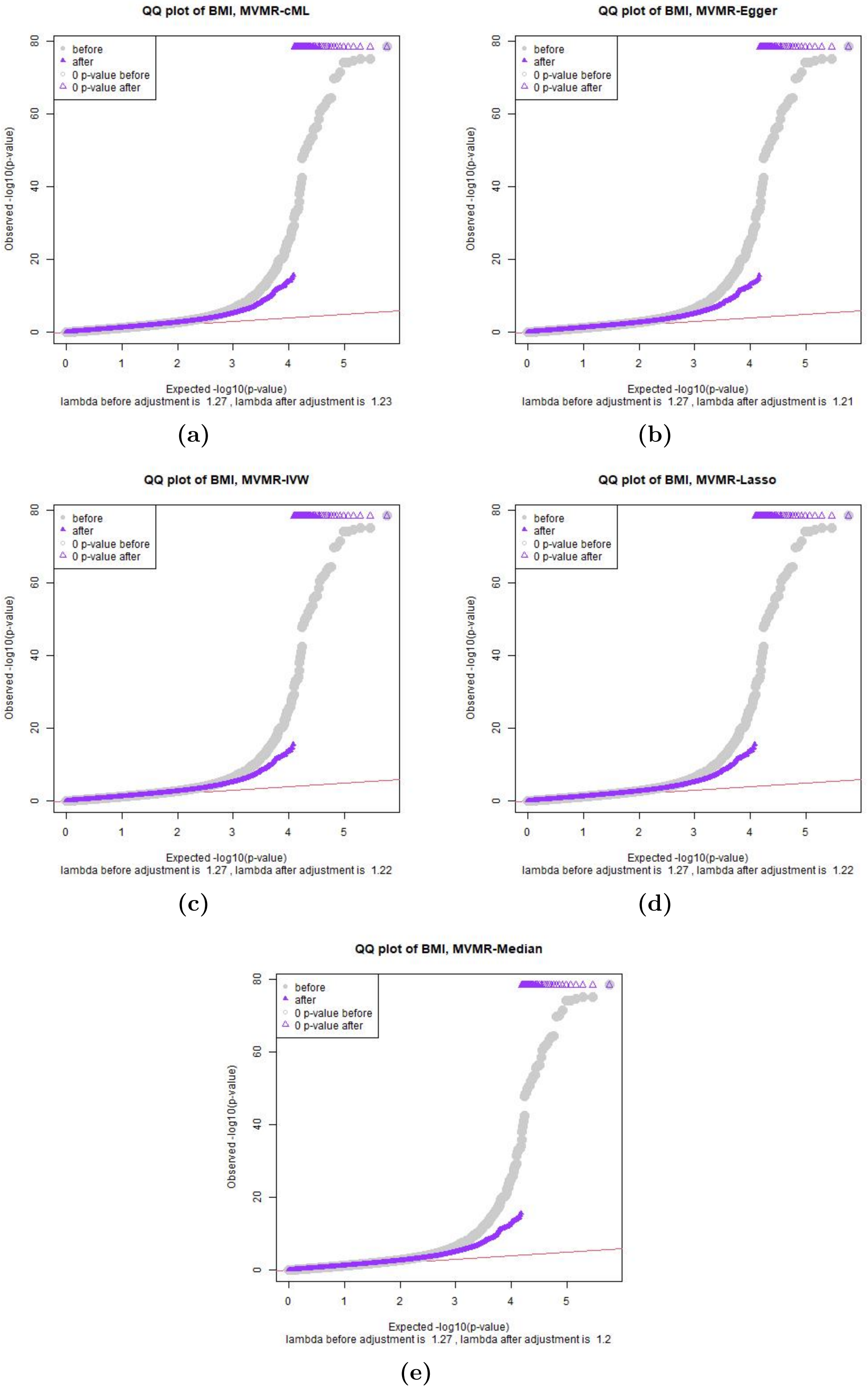

Fig BZ. QQ plots of BMI (of M_1) before and after applying bias correction. All 20 metabolomic PCs are used in M_1. 3 metabololic PCs are adjusted for bias correction. In the legends, "before" means the result before correction, "after" means the result after correction. "0 p-value before" represents the SNPs having a p-value of 0 before correction. "0 p-value after" represents the SNPs having a p-value of 0 after correction. The SNPs having p-values of 0 before or after correction are truncated at the top of plots.

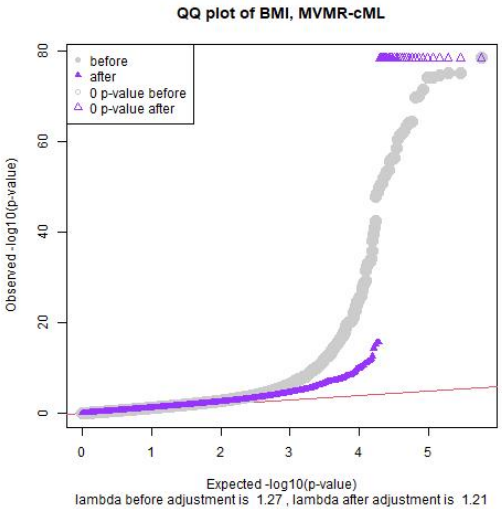

(a)

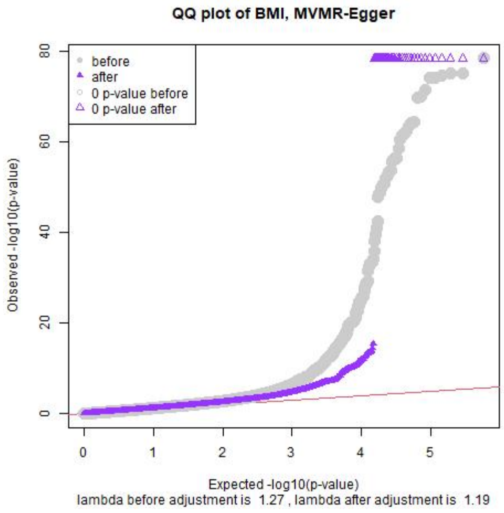

(b)

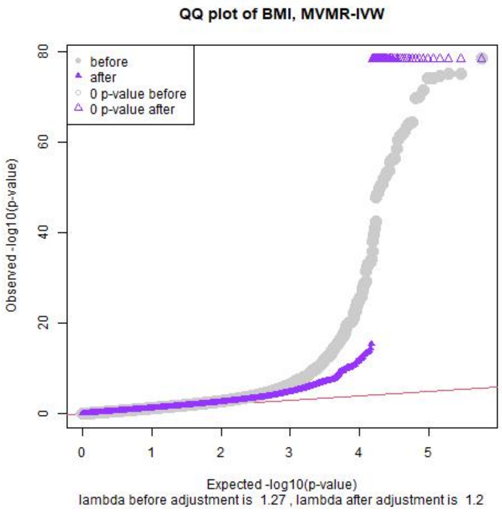

(c)

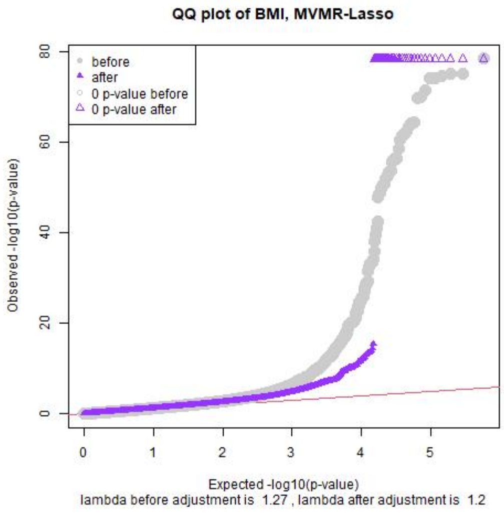

(d)

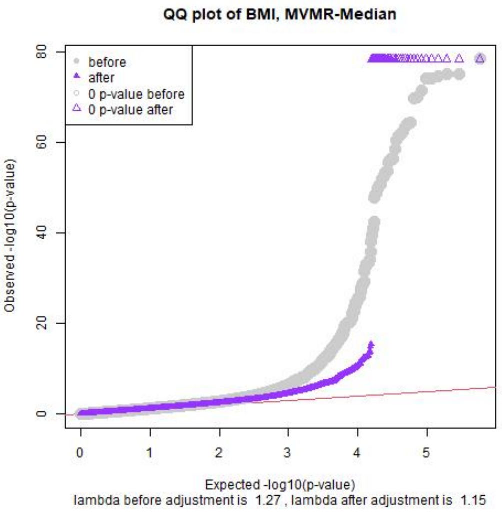

(e)

Fig CA. QQ plots of BMI (of M_1) before and after applying bias correction. All 20 metabolomic PCs are used in M_1. 4 metabololic PCs are adjusted for bias correction. In the legends, "before" means the result before correction, "after" means the result after correction. "0 p-value before" represents the SNPs having a p-value of 0 before correction. "0 p-value after" represents the SNPs having a p-value of 0 after correction. The SNPs having p-values of 0 before or after correction are truncated at the top of plots.

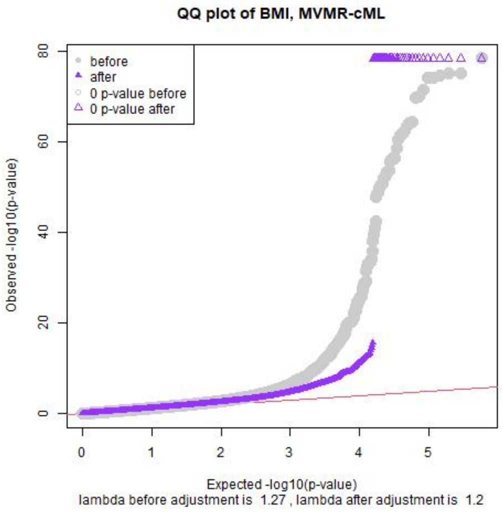

(a)

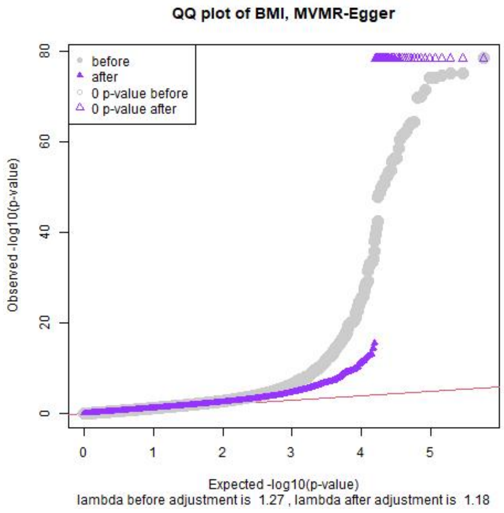

(b)

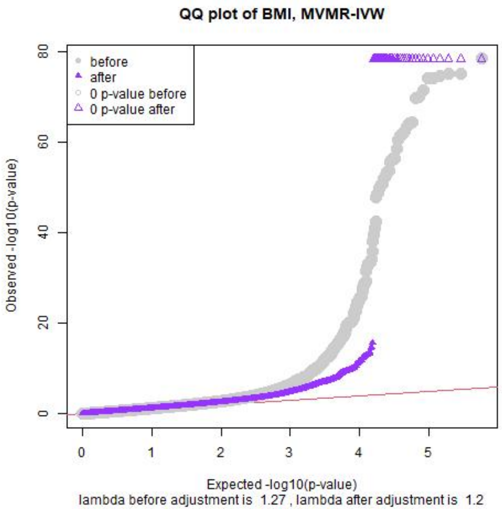

(c)

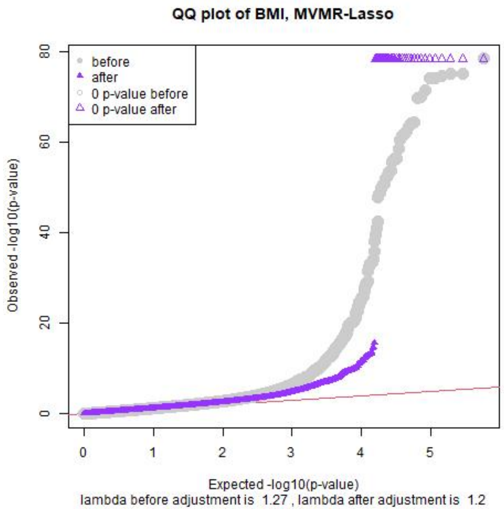

(d)

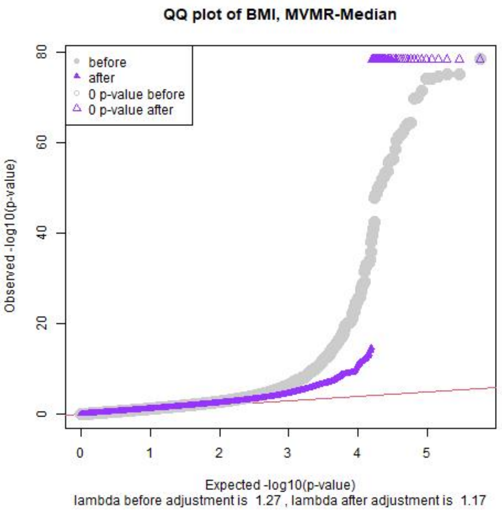

(e)

Fig CB. QQ plots of BMI (of M_1) before and after applying bias correction. All 20 metabolomic PCs are used in M_1. 5 metabololic PCs are adjusted for bias correction. In the legends, "before" means the result before correction, "after" means the result after correction. "0 p-value before" represents the SNPs having a p-value of 0 before correction. "0 p-value after" represents the SNPs having a p-value of 0 after correction. The SNPs having p-values of 0 before or after correction are truncated at the top of plots.

G.2.13 Comparison of QQ plots before and after apply different bias-correction methods on M_2

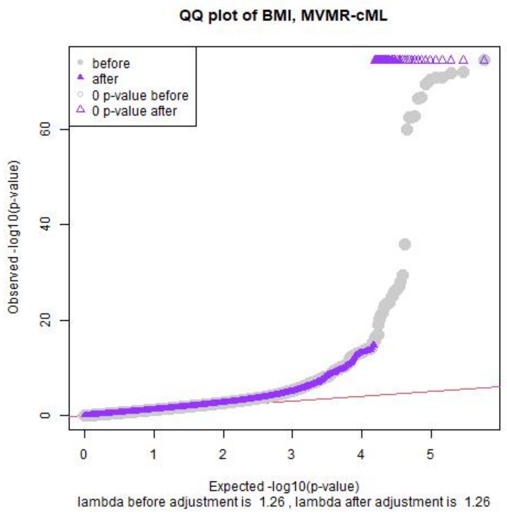

(a)

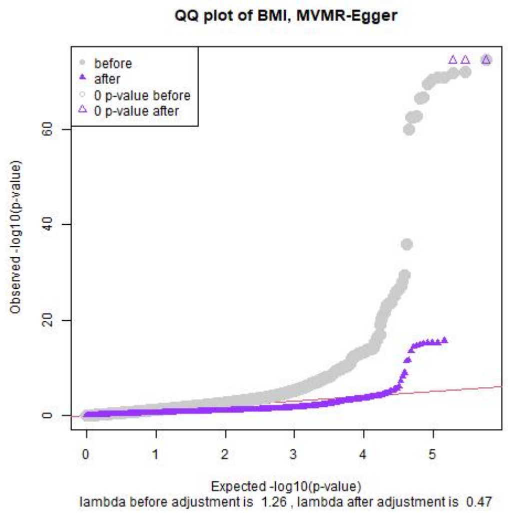

(b)

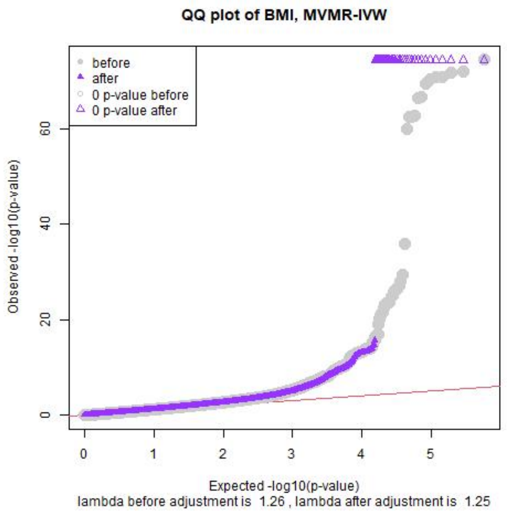

(c)

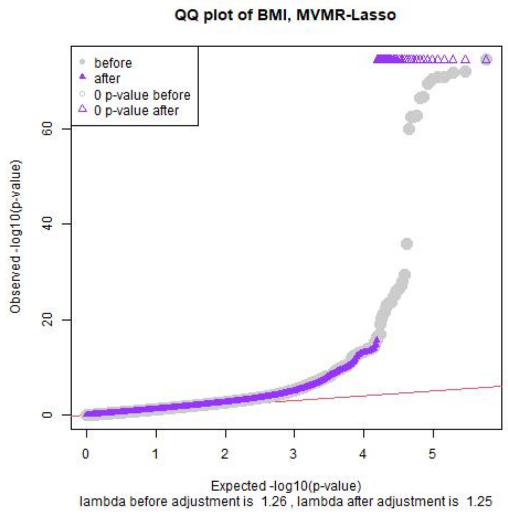

(d)

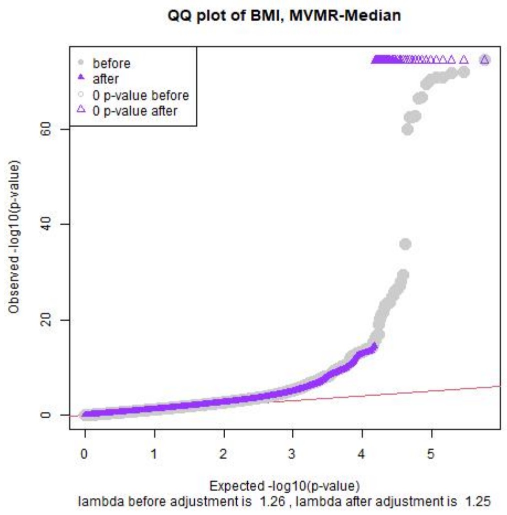

(e)

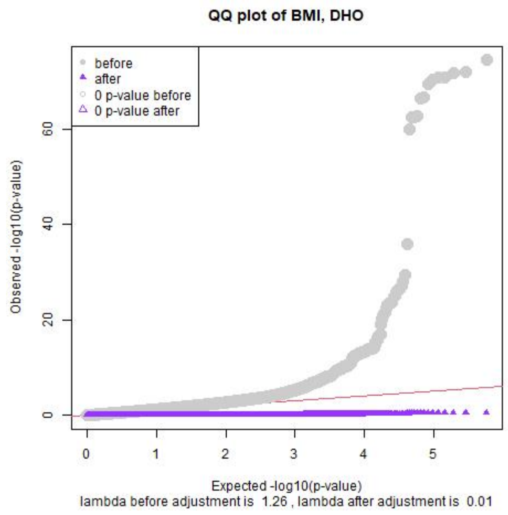

(f)

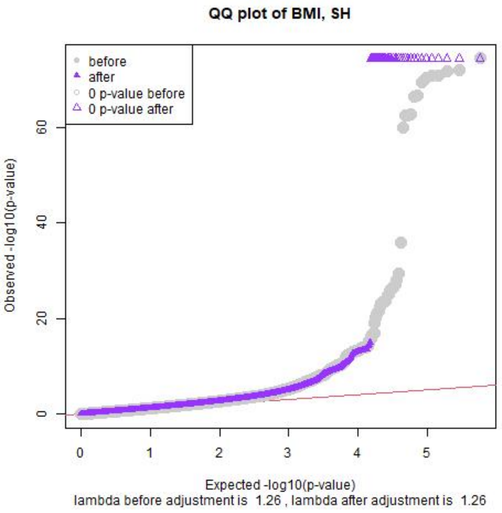

(g)

Fig CC. QQ plots of BMI (of M_2) before and after applying bias correction. 1 metabololic PC is adjusted. In the legends, "before" means the result before correction, "after" means the result after correction. "0 p-value before" represents the SNPs having a p-value of 0 before correction. "0 p-value after" represents the SNPs having a p-value of 0 after correction. The SNPs having p-values of 0 before or after correction are truncated at the top of plots.

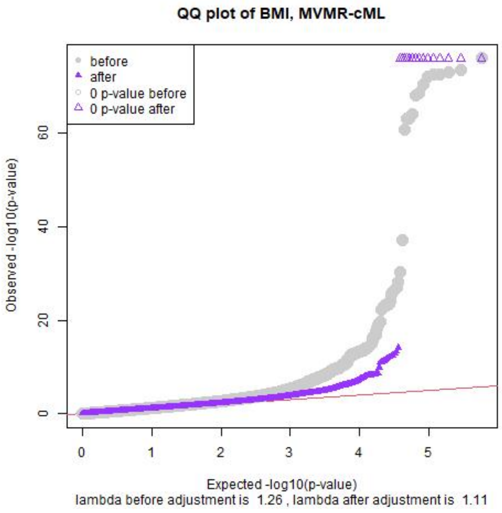

(a)

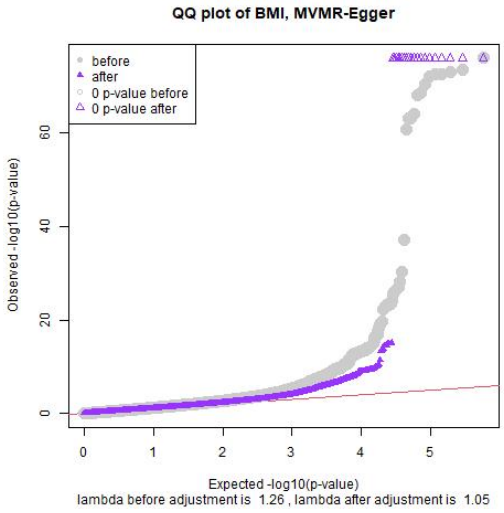

(b)

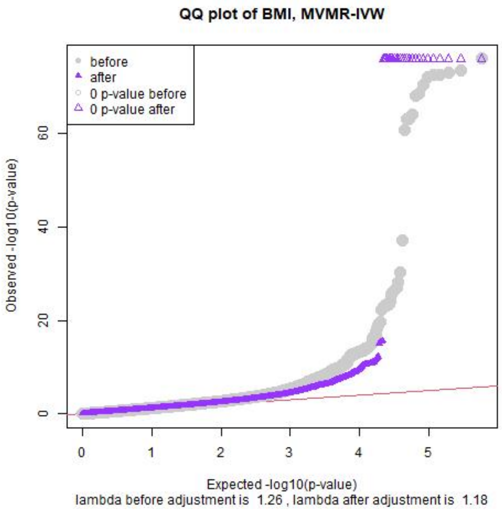

(c)

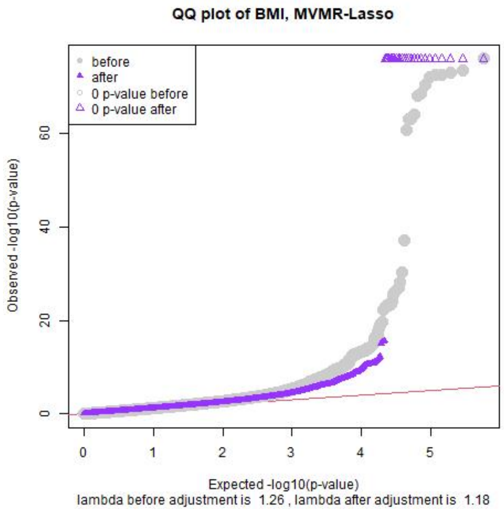

(d)

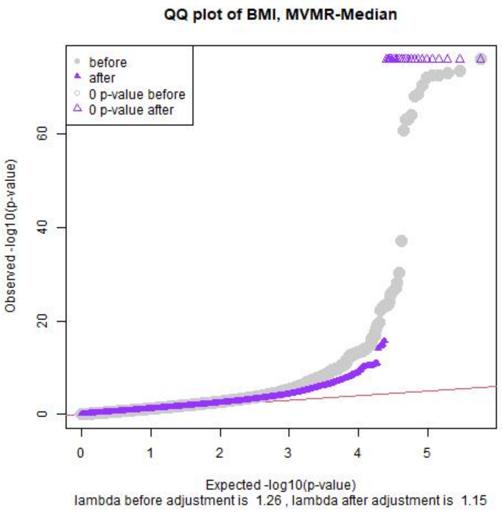

(e)

Fig CD. QQ plots of BMI (of M_2) before and after applying bias correction. 2 metabololic PCs are adjusted. In the legends, "before" means the result before correction, "after" means the result after correction. "0 p-value before" represents the SNPs having a p-value of 0 before correction. "0 p-value after" represents the SNPs having a p-value of 0 after correction. The SNPs having p-values of 0 before or after correction are truncated at the top of plots.

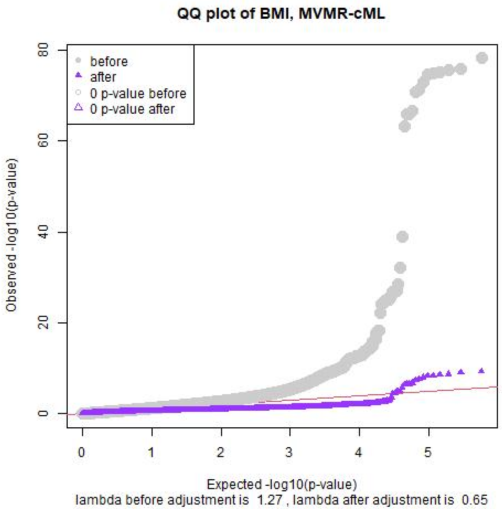

(a)

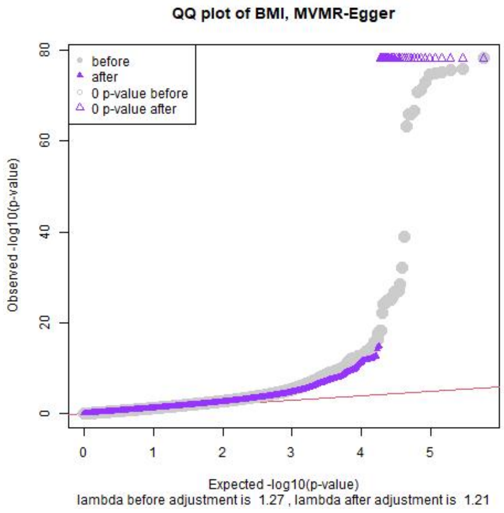

(b)

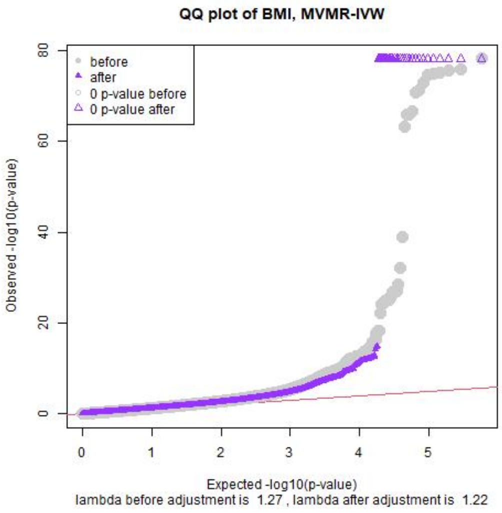

(c)

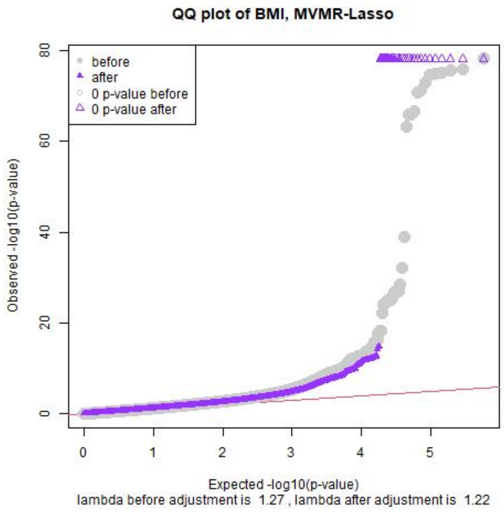

(d)

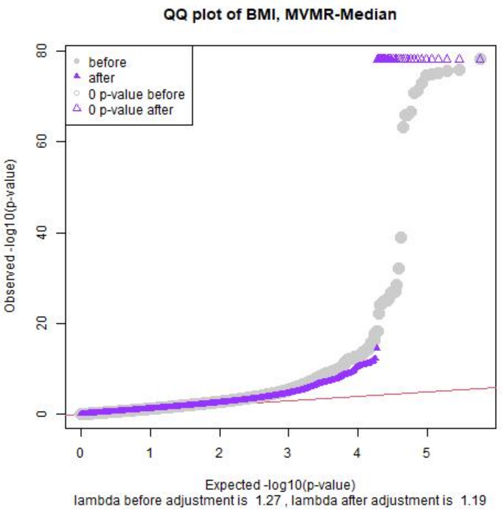

(e)

Fig CE. QQ plots of BMI (of M_2) before and after applying bias correction. 3 metabololic PCs are adjusted. In the legends, "before" means the result before correction, "after" means the result after correction. "0 p-value before" represents the SNPs having a p-value of 0 before correction. "0 p-value after" represents the SNPs having a p-value of 0 after correction. The SNPs having p-values of 0 before or after correction are truncated at the top of plots.

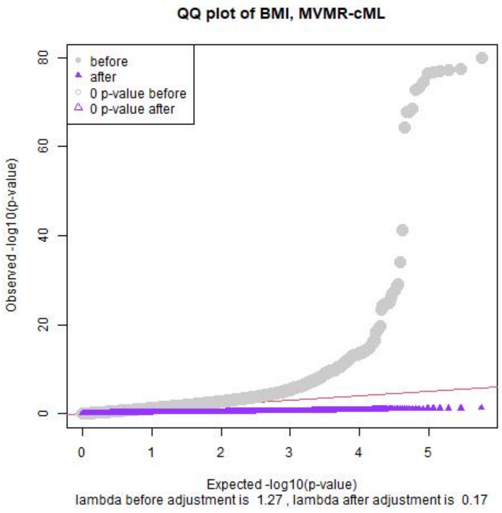

(a)

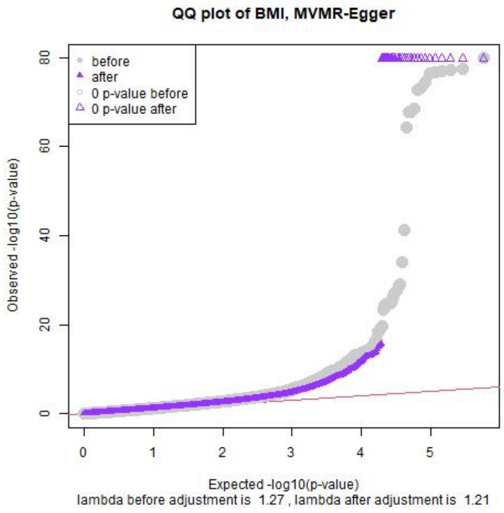

(b)

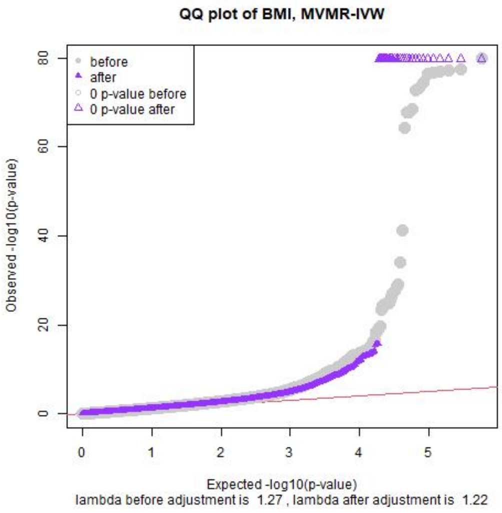

(c)

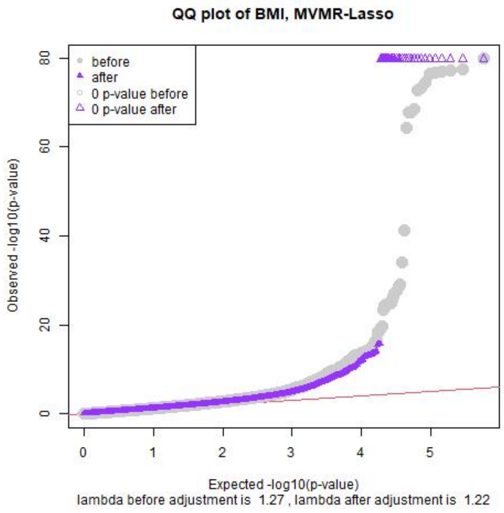

(d)

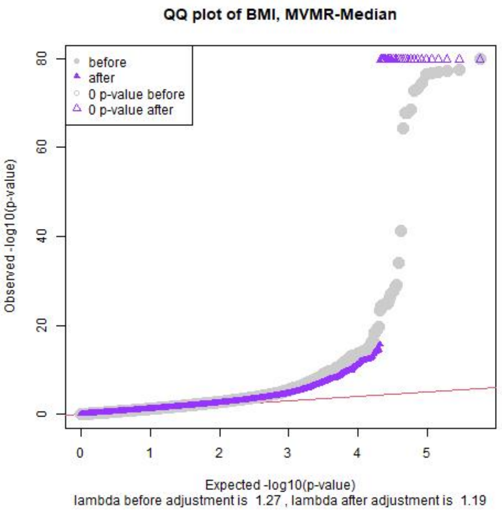

(e)

Fig CF. QQ plots of BMI (of M_2) before and after applying bias correction. 4 metabololic PCs are adjusted. In the legends, “before” means the result before correction, “after” means the result after correction. “0 p-value before” represents the SNPs having a p-value of 0 before correction. “0 p-value after” represents the SNPs having a p-value of 0 after correction. The SNPs having p-values of 0 before or after correction are truncated at the top of plots.

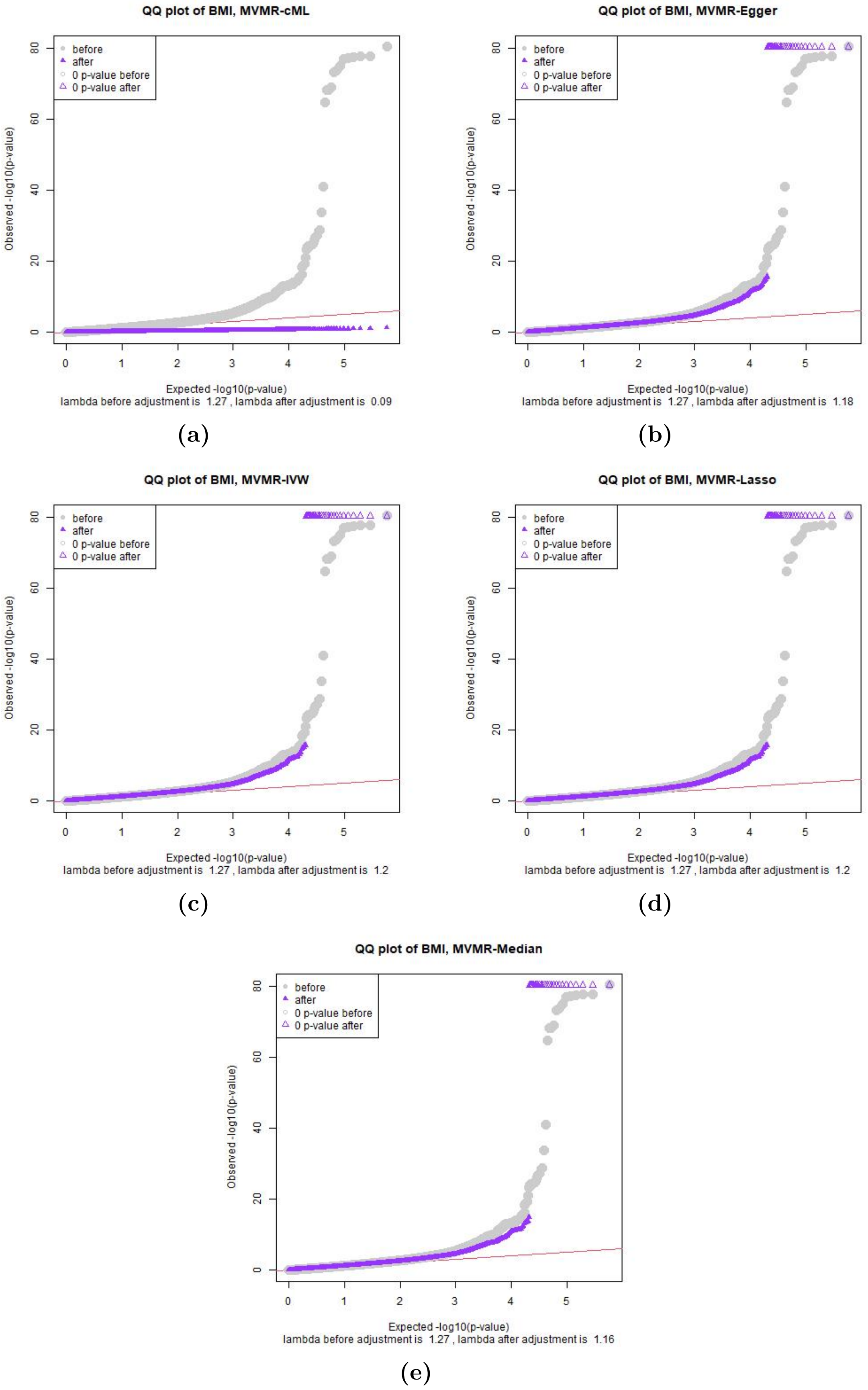

Fig CG. QQ plots of BMI (of M_2) before and after applying bias correction. 5 metabololic PCs are adjusted. In the legends, “before” means the result before correction, “after” means the result after correction. “0 p-value before” represents the SNPs having a p-value of 0 before correction. “0 p-value after” represents the SNPs having a p-value of 0 after correction. The SNPs having p-values of 0 before or after correction are truncated at the top of plots.

G.2.14 Comparison of QQ plots before and after apply different bias-correction methods on M_2, all 20 metabolomic PCs are used in M_2

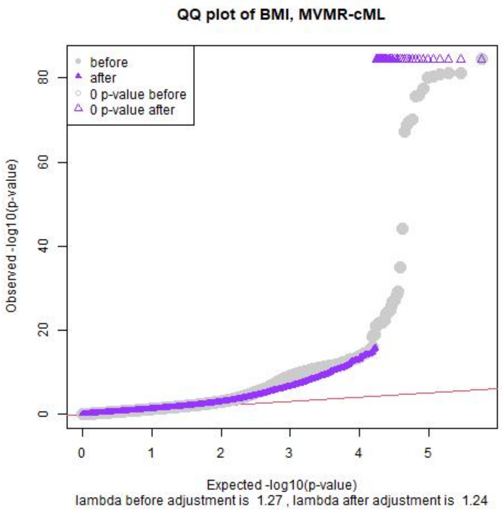

(a)

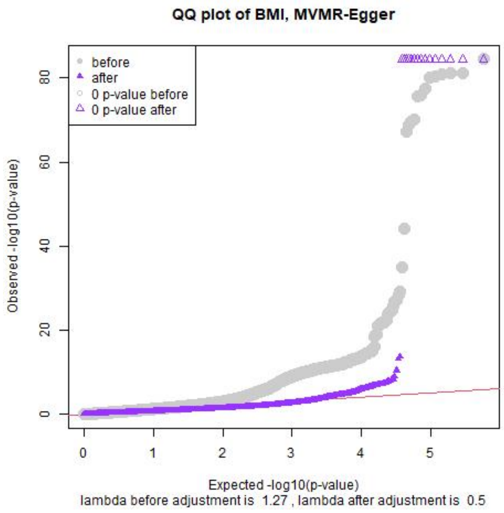

(b)

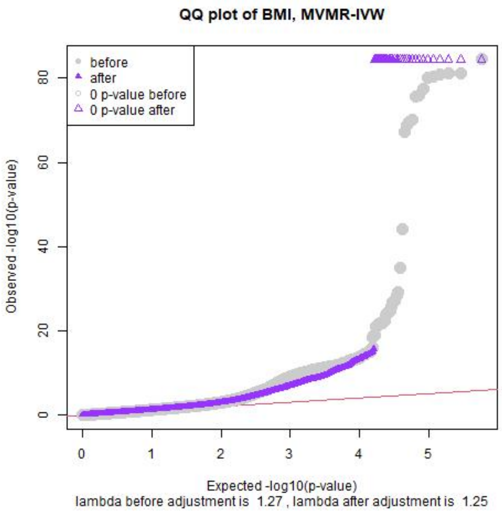

(c)

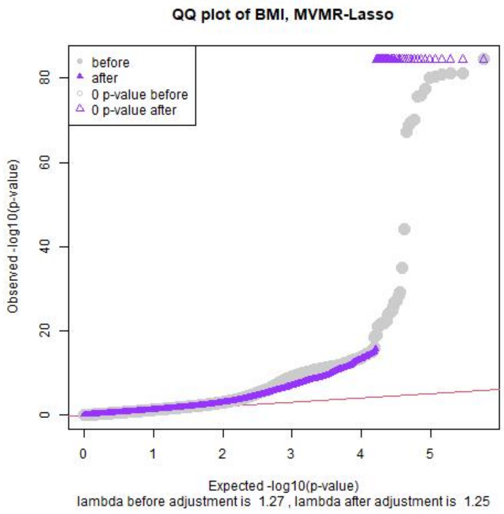

(d)

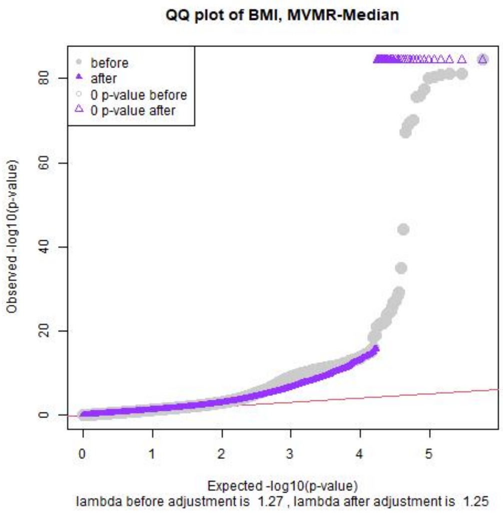

(e)

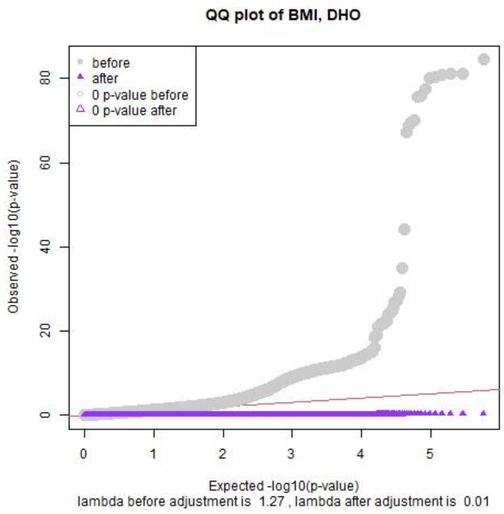

(f)

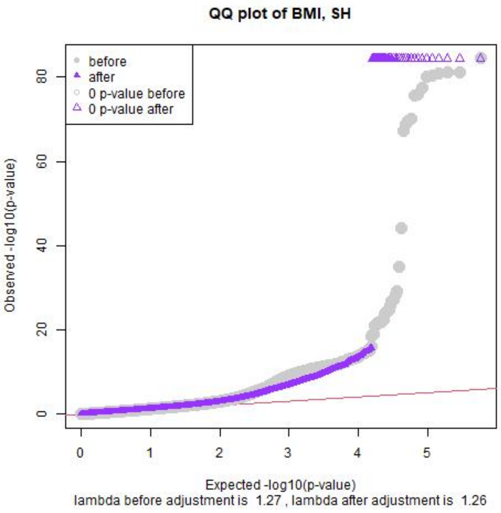

(g)

Fig CH. QQ plots of BMI (of M_2) before and after applying bias correction. All 20 metabolomic PCs are used in M_2. 1 metabololic PC is adjusted for bias correction. In the legends, "before" means the result before correction, "after" means the result after correction. "0 p-value before" represents the SNPs having a p-value of 0 before correction. "0 p-value after" represents the SNPs having a p-value of 0 after correction. The SNPs having p-values of 0 before or after correction are truncated at the top of plots.

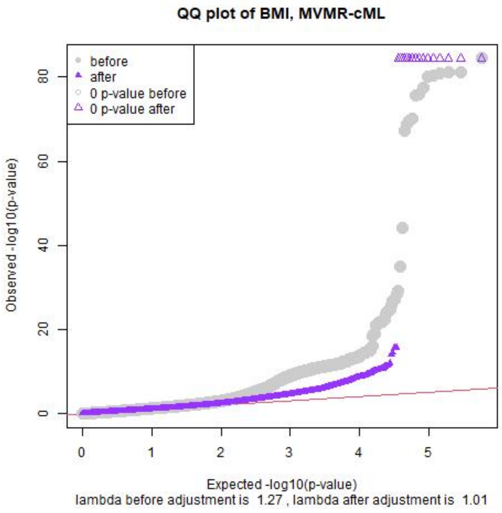

(a)

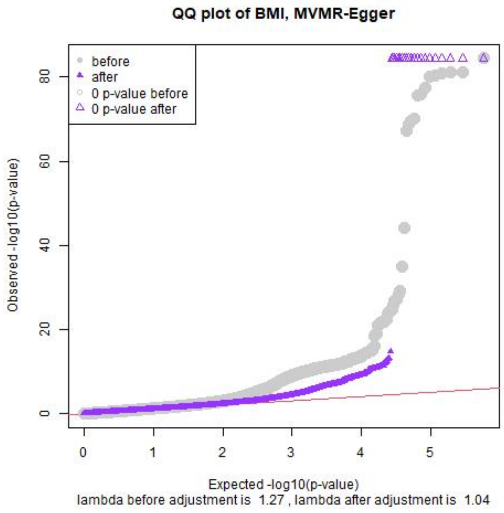

(b)

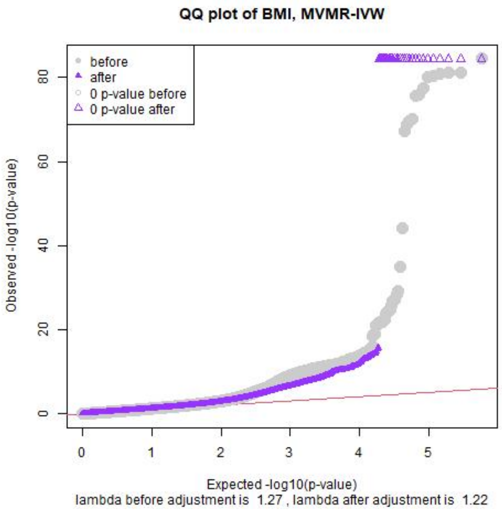

(c)

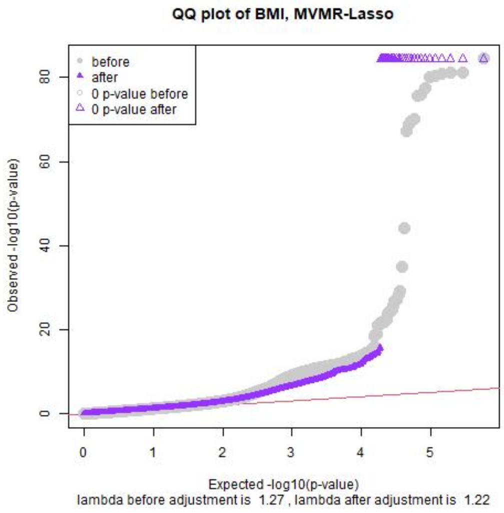

(d)

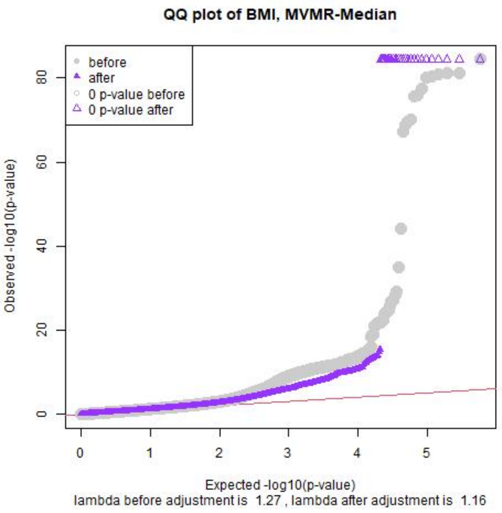

(e)

Fig CI. QQ plots of BMI (of M_2) before and after applying bias correction. All 20 metabolomic PCs are used in M_2. 2 metabololic PCs are adjusted for bias correction. In the legends, "before" means the result before correction, "after" means the result after correction. "0 p-value before" represents the SNPs having a p-value of 0 before correction. "0 p-value after" represents the SNPs having a p-value of 0 after correction. The SNPs having p-values of 0 before or after correction are truncated at the top of plots.

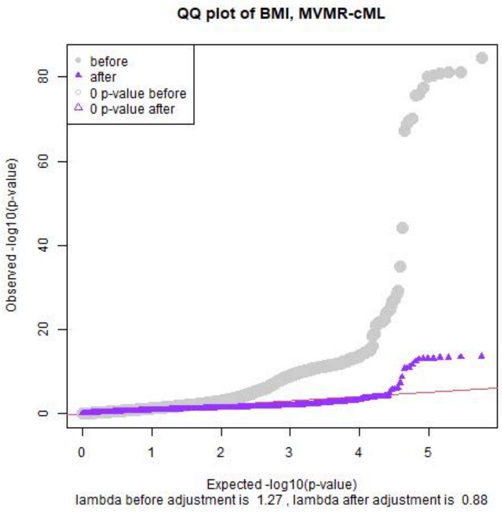

(a)

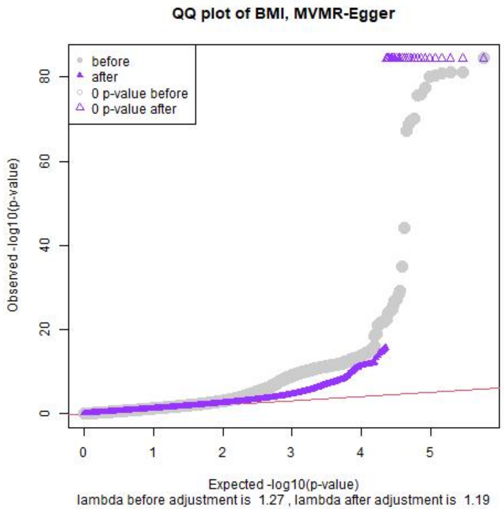

(b)

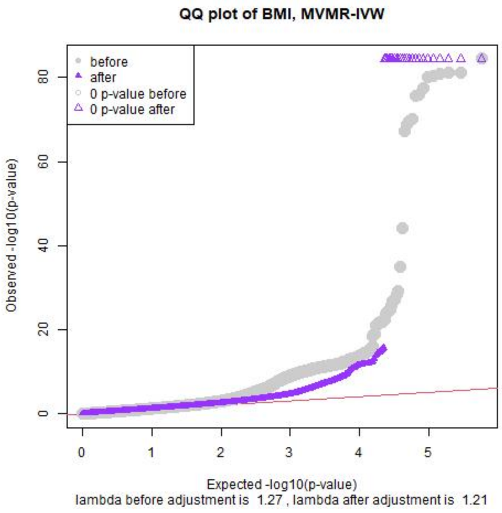

(c)

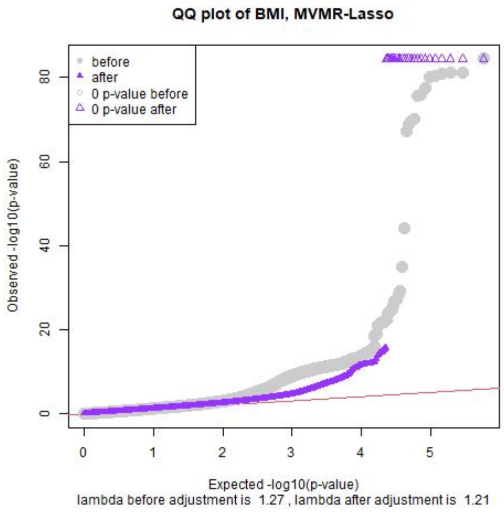

(d)

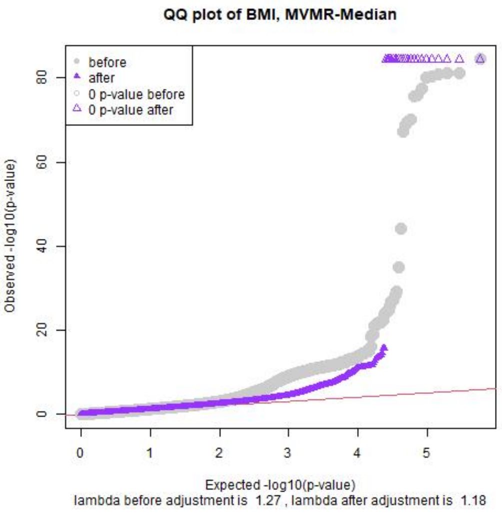

(e)

Fig CJ. QQ plots of BMI (of M_2) before and after applying bias correction. All 20 metabolomic PCs are used in M_2. 3 metabololic PCs are adjusted for bias correction. In the legends, "before" means the result before correction, "after" means the result after correction. "0 p-value before" represents the SNPs having a p-value of 0 before correction. "0 p-value after" represents the SNPs having a p-value of 0 after correction. The SNPs having p-values of 0 before or after correction are truncated at the top of plots.

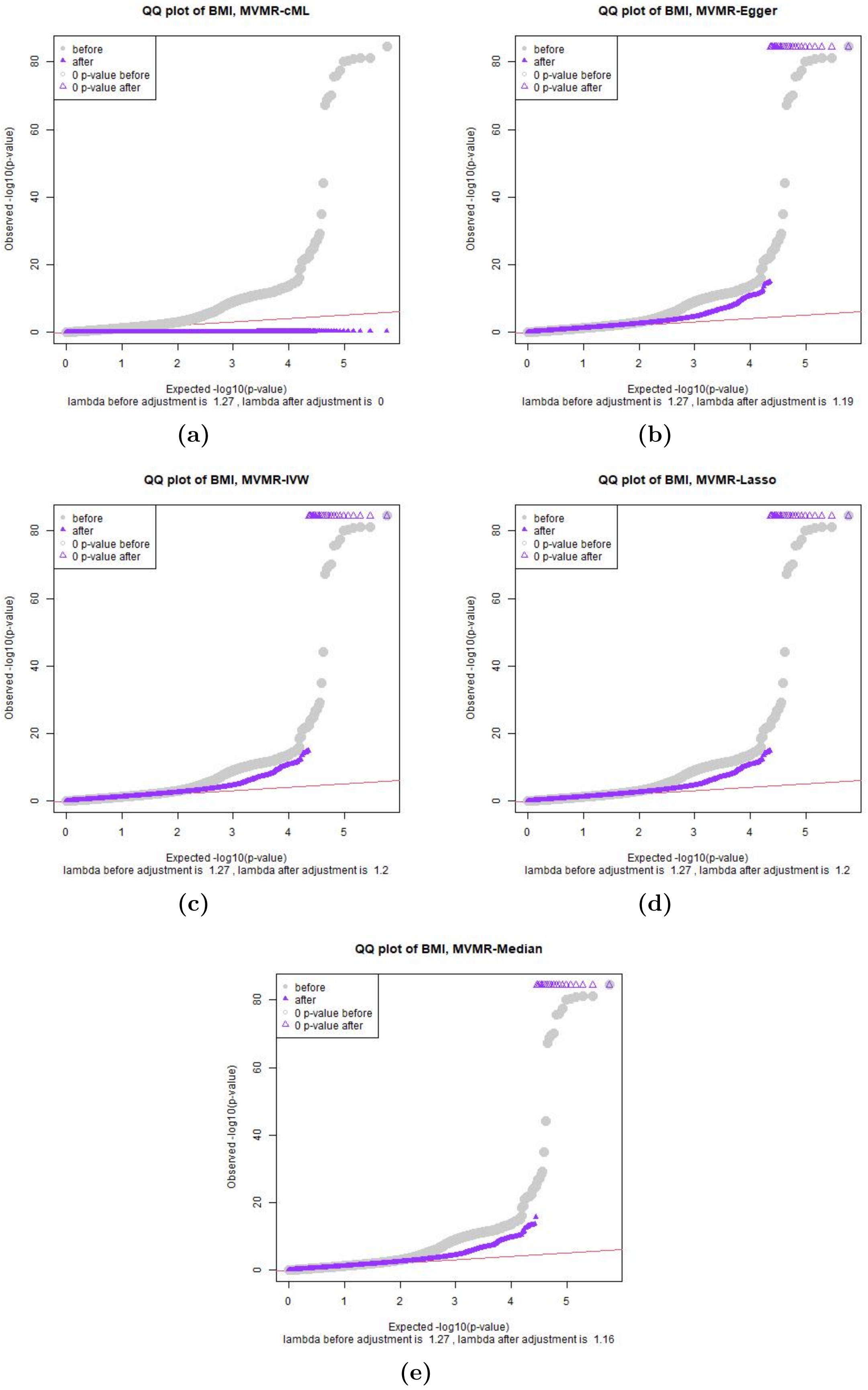

Fig CK. QQ plots of BMI (of M_2) before and after applying bias correction. All 20 metabolomic PCs are used in M_2. 4 metabololic PCs are adjusted for bias correction. In the legends, "before" means the result before correction, "after" means the result after correction. "0 p-value before" represents the SNPs having a p-value of 0 before correction. "0 p-value after" represents the SNPs having a p-value of 0 after correction. The SNPs having p-values of 0 before or after correction are truncated at the top of plots.

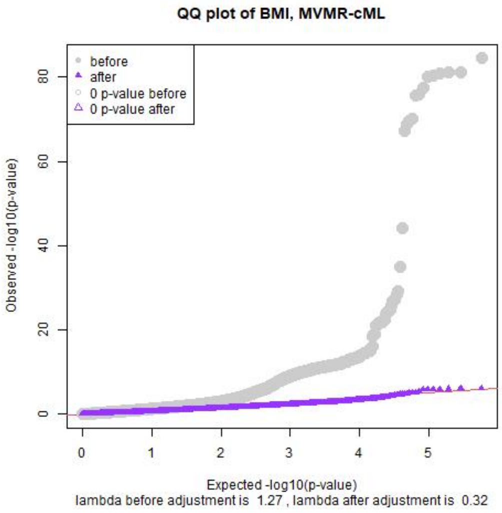

(a)

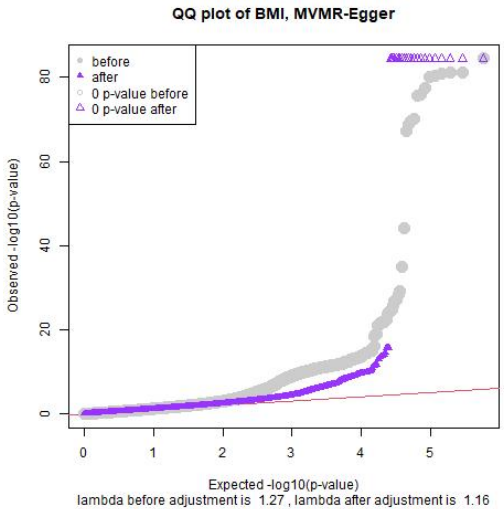

(b)

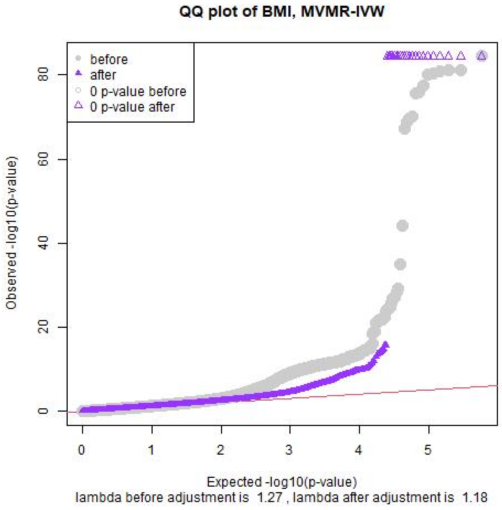

(c)

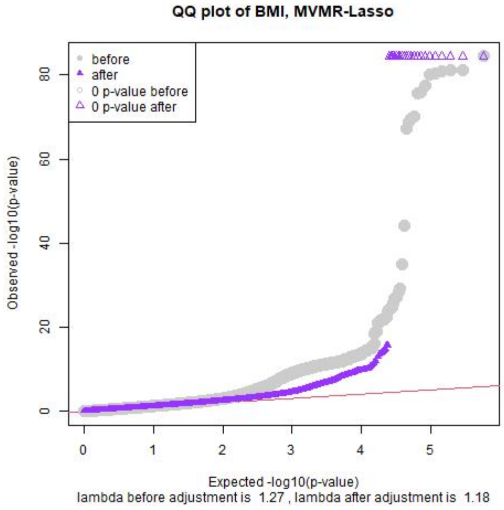

(d)

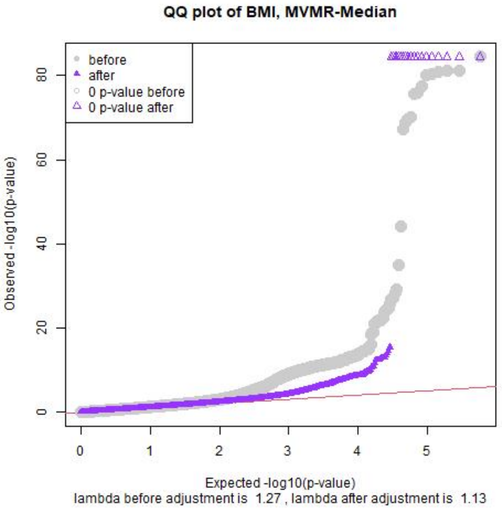

(e)

Fig CL. QQ plots of BMI (of M_2) before and after applying bias correction. All 20 metabolomic PCs are used in M_2. 5 metabololic PCs are adjusted for bias correction. In the legends, "before" means the result before correction, "after" means the result after correction. "0 p-value before" represents the SNPs having a p-value of 0 before correction. "0 p-value after" represents the SNPs having a p-value of 0 after correction. The SNPs having p-values of 0 before or after correction are truncated at the top of plots.

References

1. Dudbridge F, Allen RJ, Sheehan NA, Schmidt AF, Lee JC, Jenkins RG, et al. Adjustment for index event bias in genome-wide association studies of subsequent events. Nat Commun. 2019;10(1):1561. doi:10.1038/s41467-019-09381-w.

2. Lin Z, Haoran X, Wei P. Combining Mendelian randomization and network deconvolution for inference of causal networks with GWAS summary data. PloS Genet. 2023;19(5):e1010762. doi:10.1371/journal.pgen.1010762.

3. Lin Z, Xue H, Pan W. Robust multivariable Mendelian randomization based on constrained maximum likelihood. Am J Hum Genet. 2023;110(4):592–605. doi:https://doi.org/10.1016/j.ajhg.2023.02.014.

4. Xue H, Shen X, Pan W. Constrained maximum likelihood-based Mendelian randomization robust to both correlated and uncorrelated pleiotropic effects. Am J Hum Genet. 2021;108(7):1251–1269. doi:10.1016/j.ajhg.2021.05.014.

5. Bowden J, Davey Smith G, Burgess S. Mendelian randomization with invalid instruments: effect estimation and bias detection through Egger regression. Int J Epidemiol. 2015;44(2):512–525. doi:10.1093/ije/dyv080.

6. Mahmoud O, Dudbridge F, Davey Smith G, Munafo M, Tilling K. A robust method for collider bias correction in conditional genome-wide association studies. Nat Commun. 2022;13(1):619. doi:10.1038/s41467-022-28119-9.

7. Berisa T, Pickrell JK. Approximately independent linkage disequilibrium blocks in human populations. Bioinformatics. 2016;32(2):283–285. doi:10.1093/bioinformatics/btv546.

8. Ge T, Chen CY, Ni Y, Feng YCA, Smoller JW. Polygenic prediction via Bayesian regression and continuous shrinkage priors. Nat Commun. 2019;10(1):1776. doi:10.1038/s41467-019-09718-5.

9. Lin Z, Knutson KA, Pan W. Leveraging omics data to boost the power of genome-wide association studies. HGG Adv. 2022;3(4):100144. doi:10.1016/j.xhgg.2022.100144.

10. Sanderson E, Spiller W, Bowden J. Testing and correcting for weak and pleiotropic instruments in two-sample multivariable Mendelian randomization. Stat Med. 2021;40(25):5434–5452. doi:10.1002/sim.9133.
