# Supplementary material for: Collider bias correction for multiple covariates in GWAS using robust multivariable Mendelian randomization
Source: PLoS Genet. 2024 Apr 22;20(4):e1011246. doi: 10.1371/journal.pgen.1011246 (PMC11065275; doi:10.1371/journal.pgen.1011246)
Supplement: Attachment — Submitted filename: author_one_to_one_response.pdf [file pgen.1011246.s002.pdf]

Authors' one-to-one responses

We appreciate the insightful, constructive and helpful comments from the three reviewers. Accordingly, we have made concerted efforts to carefully address each question raised as detailed below. The new or modified parts are highlighted in blue in the main text and Supplementary.

1 Reviewer 1

1. *In Figure 2, H_1 is both the mediator in the pathway $G \to H_1 \to Y$ and a collider in the pathway $G \to H_1 \leftarrow U \to Y$. I wonder how this can be determined in practice, that is, how can we test whether H_1 plays both roles as those depicted in Figure 2?*

Response: Thanks for your question. First, we do not actually test the roles of H_1. It is hypothesized (or implicitly modeled) based on prior knowledge that if the roles of H_1 as described in Figure 2 is possible, then collider bias may result and some correction may be needed, as shown in our real data examples and by others in the literature. If the hypothesis is incorrect, then bias correction may not be needed; as shown in our new simulation study, in such a case the loss of power by bias correction is minimal. Second, to answer your question directly, i) in $G \to H_1 \to Y$, $G \to H_1$ can be tested using fine mapping with GWAS data of H_1, and $H_1 \to Y$ can be evaluated using some MR methods; ii) in general $G \to H_1 \leftarrow U \to Y$ cannot be directly tested because U is not observed.

2. *" I suggested the authors conducted additional simulations to compare the performance of the traditional GWAS and the proposed bias correction method when H is not a collider?"*

Response: Yes, we have conducted a new simulation where there was no common confounder for the covariates and outcome ($\boldsymbol{b} = \mathbf{0}$), and hence no collider bias. This simulation was based on independent SNPs and only considered valid IVs. The means that the effect estimates are close to the true values both before and after applying our

bias correction method. There is a small inflation of the variances of the point estimates after bias correction. The detailed results are in section 3 in the supplementary file.

3. *"If H_1 is a mediator, it should not be accounted for in GWAS analysis. In addition, if we remove the edge $H \rightarrow Y$ and render H_1 a complete collider, it is generally not recommended adjust for it in GWAS. Thus, Figure 2 is confusing."*

Response: If H_1 is a mediator between SNP G and outcome Y, as shown in the literature one may include it in a *conditional* GWAS analysis of Y in order to estimate the direct association/effect of G on Y through pathways *not* mediated via H_1[2]. For example, adjusting for BMI in a conditional GWAS analysis of whist-to-hip ratio (WHR) could estimate the direct association between G and WHR not mediated via BMI[4], which may be the question of interest. Yes, we agree that if H_1 is a merely a collider and is indeed known to have no effect on Y, there is no need to adjust for it in GWAS.

4. *" In Figure 4, the effect estimates of WHR before and after bias correction appear to be quite similar. I suggested the authors to try different data set to highlight the advantage of the proposed method."*

Response: Thanks for comment. Actually we'd like to use this example to represent a "null" case with no or negligible collider bias, in which our bias-correction method yielded similar results to those without correction. Our conclusion was consistent with a previous study showing that adjusting for BMI in the conditional GWAS of WHR introduced minimal collider bias[2]. In contrast, in the example of the GWAS of BMI adjusted for multiple metabolomic principle components (PCs), collider bias was detected, and our approach demonstrated effectiveness in correcting the bias of SNP effect estimates.

5. *" The manuscript attempts to utilize MVMR to correct collider bias in GWAS, but the depiction of direct effect or indirect effect in Figure 2 is confusing."*

Response: Thanks for comment. In Figure 2, we illustrate a scenario of causal relationships among variables. The direct effect β_{G_iY} is the target parameter to be estimated throughout the paper. The SNP effects on Y consists of the direct effect β_{G_iY}, as well as the indirect effect through covariates H_1 and H_2, namely $\beta_{G_iH_1}\beta_{H_1Y} + \beta_{G_iH_1}\beta_{H_1Y}$. We adjust for the covariates H_1 and H_2 in a conditional GWAS of Y in order to estimate the direct effect. However, as both G_i and the confounder U have effects on both H_1 and H_2, conditioning on the two covariates induces an indirect association $G \rightarrow U \rightarrow Y$, which will bias the estimation of the direct effect β_{G_iY}, known as collider

bias. We explained this in section 2.1 in the second paragraph and marked it blue. Hence, Figure 2 is only used to illustrate a scenario where collider bias would arise (and thsu the need to correct the collider bias by possible use of MVMR), instead of the causal framework of MVMR, which is not the main focus of this paper. MVMR is only used to estimate the slope vector $\boldsymbol{b}$, which is then used to estimate the collider bias.

6. *The symbols in the manuscript are highly confusing and most of them are not explained, which seriously impacts the readability of the article. For example, in formula (3), K represents variables, but in formula (9), K denotes the number of invalid IVs. Furthermore, in section 2.5, rho represents correlation, whereas in section 3.1, rho denotes the proportion of invalid instrumental variables. What does the sudden appearance of V in formula (1) represent? What are its practical implications? In the simulation settings, V disappears again. Is V a vector of covariates or SNPs?"*

Response: Thanks for pointing this out, and accordingly we have modified some notations. We now use capital letter $\boldsymbol{J}$ to represent the non-heritable covariates in the GWAS of Y instead. In section 2.5, ρ denote the correlation between pleiotropy effects instead of the proportion of invalid IVs. However, in the description "$\rho = 0$, 30% invalid IVs" (the first paragraph of section 3.1) "ρ" and "30" might be confusing, so we have removed "$\rho = 0$". Our bias-correction method requires the GWAS summary data of both Y and each covariate in $\boldsymbol{H}$, and in practice one may use other covariates in the GWAS of each element in $\boldsymbol{H}$. Hence, we use $\boldsymbol{V}$ in the formula (1) to denote the covariates that might appear in the GWAS of $\boldsymbol{H}$. Our derivation shows that our conclusion in equation (6) holds only if $\boldsymbol{V}$ is not associated with SNPs G_i and confounder U. This also implies we need the assumption that the GWAS summary data of covariates in $\boldsymbol{H}$ does not suffer from bias. Because $\boldsymbol{V}$ is not expected to influence the results much, we omitted $\boldsymbol{V}$ in simulations for simplicity and clarity.

7. *If Y is a binary variable, such as diseases, does this method remain effective?*

Response: Thanks for the comments. In this paper we have only considered quantitative traits, and our bias correction method is based on linear regression (as in the several closely related papers cited therein). For a binary outcome, a linear regression model may be used to approximate a logistic regression model to estimate the marginal SNP effects, given the small effect sizes of SNPs. However, in the current context, some heritable covariates are adjusted in GWAS, which may have large effects on the outcome. In this case, a linear model is not adequate and our bias-correction method is not applicable. Given the challenges with binary traits, it is beyond the scope of this paper and we leave it as future work. We have mentioned this limitation in the

Discussion section.

8. *"MVMR assumes the IV is associated with at least one of H, and the SNP directly associated with Y is invalid SNP. GWAS aims to find the SNPs directly associated with Y, but MVMR-cML assume the plurality condition, and in formula (9) the number of invalid IV is constrained to K. How to interpret this?"*

Response: Thank you for the comment. Since we only require and use a few SNPs as IVs, it will not influence the remaining overwhelming majority of the SNPs in GWAS as the targets of GWAS. This might be better seen as in our simulation set-ups.

9. *"How to use this method in practice? Did this method be applied to thousands of SNPs one by one? What is the computational time?"*

Response: Our method first (and for once) estimate $\boldsymbol{b}$ using a few IVs and MVMR, then the bias-correction method is applied to every SNP in the GWAS summary data of Y. Because the parameter estimation needed for bias-correction is conducted only once, the computational time is similar to a usual GWAS scan. As described in our second real data example, for the GWAS data of BMI and 5 metabolomic principle components in $\boldsymbol{H}$, the computational time was about 30 minutes on a personal computer, if we use the simplified variance estimator in equation (14). If we use the variance estimator in Theorem 1, the time will be about 30 minutes with parallel computing. Totally 80 cores were used. In total 500,000 SNPs were in the GWAS data of BMI. Since the two variance estimators gave similar results, in practice the simplified version can be used to save computational time.

10. *I strongly suggested the authors to re-organize the introduction of the manuscript, to further highlight the significance and motivation of the proposed method."*

Response: Thanks for the suggestion. Yes, we have rewritten many parts of the Introduction section, including removing some unnecessary parts such as the long descriptions of MVMR methods, and emphasizing the collider bias problem and our contributions.

2 Reviewer 2

1. *" In several places the authors state that their method can account for overlapping samples, and even say (section 2.4) that they have simulations confirming that previous methods have inflated type-I errors in this case. However I could find no such simulations in the paper. Moreover, Dudbridge et al (2019)[3] argued that their approach was*

robust to overlapping samples, and confirmed it in their simulations. This was further established by Barry et al. (PLoS Genet 2021)[1]. Please explain how your situation differs from theirs, and provide simulations to back up any new claims."

Response: Thanks for pointing this out. Actually in the previous submitted version, when applying MVMR-cML, we used the phenotype correlations to estimate the correlations between the GWAS summary statistics[7], which led to an overestimation of the correlation and hence the variance of the final estimate $\hat{\beta}_{G_iY}$. As a result MVMR-cML produced a seemingly lower type-I error rate than other methods. In the current revised version we used the correlation between the null z-scores to estimate the correlation of summary statistics[6]. Then the variance of $\hat{\beta}_{G_iY}$ were well-estimated for most SNPs, as shown in Table S16-Table S18; the standard errors (SEs) of cML were close to the empirical standard deviation (SD) for most SNPs. In the new simulation and real data results, sample overlap and LD among SNPs had minior influence on the variance estimate of $\hat{\beta}_{G_iY}$, and hence on the type-I errors. When more than 1 covariate was adjsuted, or when the InSIDE assumption was violated, some methods had slightly inflated type-I error rates. Yes, we agree with the reviewer that the method of Dudbridge et al. (2021)[3] is robust to sample overlap, which was also confirmed in Table 1 in our paper, where it reduced the type-I error rate to the nominal level under 1-sample setting. We have pointed this out in section 3.1.1 (marked in blue).

2. *" The authors claim to have derived the variance of their estimator, and that this goes beyond previous work. However, across all the results the new method's SE is consistently overestimated compared to the empirical SD, and the type-1 errors are conservative, whereas previous methods appear to be well calibrated. So something is not right here."*

Response: As mentioned in the response to the previous question, in the last submitted version, when applying MVMR-cML, we used the phenotype correlations to estimate the correlations between the GWAS summary statistics[7], which led to an overestimation of the correlation, and hence the variance of the final estimate $\hat{\beta}_{G_iY}$. In the revised version we have now used the correlations between null z-scores to estimate the correlations between two GWAS summary datasets[6]. Then for MVMR-cML the standard errors of $\hat{\beta}_{G_iY}$ were well-estimated for most SNPs, close to the empirical standard deviations (SD). The results are given in Table S16-Table S18 in supplementary.

3. *In some places the superscript T means "transpose", and in others "total" – this is confusing. See e.g. equations (2)-(3) and the text above."*

Response: Thanks for pointing this out. In the revision, we have used the superscript "*Total*" ($\beta_{G_iY}^{Total}$) to denote the total effect of SNPs G_i on Y. This only appears twice in the paper.

4. *P9 "The correlation parameters in* $\boldsymbol{\Sigma}_j$*" repeats earlier text."*

Response: Thank you for your careful reading! We have corrected this typo.

5. *The simulation used 1000 SNPs from chromosome 6. Why was this chromosome used, and was the HLA region avoided?"*

Response: Thanks for the comment. Chromosome 6 was arbitrarily chosen, and actually the high LD region on chromosome 6 might have caused some problems. In our revision, we have redone the simulation using (weakly) correlated SNPs from chromosome 1. We have also now ensured that the non-null or effective SNPs (affecting the outcome or covariates) were independent of null SNPs. More details were given in the simulation set up in the paper. We have used correlated SNPs because we want to compare our newly proposed variance estimator based on MVMR-cML with the variance estimator in equation (14); the former one accounts for the LD among SNPs, and the latter one was an extension of previous work[3]. Our new simulation and real data results showed that the LD among SNPs had minor influence on variance estimation.

6. *" Section 3.1.1, I couldn't understand why the type-I errors were not correctly controlled, not just "better controlled", especially at* $p_2 = 4$*. I see that the simulation had invalid IVs, but the pleiotropy appears to be balanced with the InSIDE assumption valid, so the methods should all be OK. What is happening here? It would help to give a simulation under the assumptions of the method, showing that it does work in that case."*

Response: Thanks for the comments. In our previous simulation, some null SNP were correlated with non-null SNPs (because they were randomly sampled from chromosome 6), causing various problems. Accordingly we have redone the simulation using (weakly) correlated SNPs from chromosome 1, and the effective SNPs affecting the outcome Y or covariates $\boldsymbol{H}$ were correlated with each other. Hence, the inflation of type-I error when $p_2 = 4$ is mainly caused by the inter-SNP correlations and invalid IVs. More specifically, for the effective SNPs their summary statistics of covariates $\hat{\boldsymbol{\beta}}_{G_i\boldsymbol{H}}$ exhibited slight bias due to inter-SNP correlation, as our GWAS is conducted marginally for each SNP. When $p_2 = 1$, the bias was small and had negligible impact on bias correction. However, with the inclusion of more covariates in the analysis, the collective estimation error of the

entire vector $\hat{\boldsymbol{\beta}}_{G_i\boldsymbol{H}}$ increased. According to equation (12) in paper, this reduced the accuracy of our bias-correction method, especially when $p_2 = 4$. In Table 2, $\hat{\boldsymbol{b}}$ was slightly biased from its true value due to invalid IVs. Even the bias was small for a single element, as more covariates joined the analysis, the aggregated estimation error of the entire vector $\hat{\boldsymbol{b}}$ also got large, which will further reduce the effectiveness of our bias correction method.

In section 3 of supplementary, we show a new simulation without pleiotropy, where SNPs were independent. In this ideal case, only valid IVs were used. After bias correction, the type-I errors were correctly controlled and type-I error rates were close or equal to the nominal level 0.05.

7. *I was surprised that the authors have considered Egger regression, since the same authors have published a convincing refutation of that method."*

Response:

Yes, we used Egger regression as a benchmark because it was used in the previous papers about collider bias[2]. The paper of Lin et al. (2022)[5] discussed its potential issue: the result of univariate Egger regression depends on SNP coding. They found that under directional pleiotropy, Egger regression may bias the causal estimate under the default coding (all SNPs are flipped to have positive effects on the exposure), even all IVs are valid. Under balanced pleiotropy, Egger regression is accurate or only slightly biased under different coding schemes, while its estimates may have a large variance under default coding. Our simulation only considered balanced pleiotropy, and SNPs were oriented to have positive effects on the fist covariate. In the univariate case where only 1 covariate is adjusted, our result is consistent with Lin et al. (2022)[5] such that the mean estimate $\hat{\boldsymbol{b}}$ (a scalar) of Egger regression is close to the true value, while the variance of $\hat{\boldsymbol{b}}$ (shown by SD and SE) of Egger regression is larger compared to other methods, as indicated in Table 2 in the paper. We have explained this in section 2.2.

8. *"P15 "This deviation was attributed to ... more covariates". This seems to attribute bias to an increase in sampling variance, which is not right."*

Thanks for the comments. Actually we have updated the simulation using weakly correlated SNPs from chromosome 1, and the effective SNPs (affectting the outcome Y or covariates $\boldsymbol{H}$) were correlated with each other. Hence, similar to question 6, this deviation from the identity line is mainly caused by the inter-SNP correlations and invalid IVs. For the effective SNPs their summary statistics $\hat{\boldsymbol{\beta}}_{G_i\boldsymbol{H}}$ exhibited slight bias

owing to inter-SNP correlations, as our GWAS analysis was conducted marginally for each SNP. And the aggregated estimation error of the vectors $\hat{\boldsymbol{\beta}}_{G_i\boldsymbol{H}}$ and $\hat{\boldsymbol{b}}$ increased with the inclusion of more covariates, which will further reduce the effectiveness of our bias correction method. Hence, in Figure 3, the points were slightly deviated from the identity line when $p_2 = 2$ or 4. We have explained this in section 3.1.2 in the paper.

9. *" Supp P6. Second line of proof, "negative likelihood" should be log likelihood."*

Response: Thanks for pointing this out! We corrected this typo.

3 Reviewer 3

1. *" Foundation of the causal framework setup. In Figure 2, the authors draw the causal DAG and claims that the true parameter to estimate should be the direct effect of SNP i β_{G_iY}. I think this is very confusing and misleading as β_{G_iY} will never be the direct effect of SNP i even when there is no collider bias as other SNPs are not jointly considered. Even without collider bias, the marginal associations between SNP i and Y without adjusting for other SNPs are never claimed to be any causal effect of SNP i. Figure 2 is also not qualified to be a DAG as only a single SNP is considered here. Given that what GWAS summary statistics provide are never any causal effect of SNP i, I think the target of the problem in the paper to adjust for collider bias is very vague. I do agree that collider bias exists but I'm only convinced that it should be adjusted when we want to identify causal SNPs using methods that jointly consider multiple SNP such as SUSIE. I'm not convinced for GWAS summary statistics. Thus, I think the authors should clarify the setup and maybe give a few more concrete examples to address the necessity."*

Response: Thanks for pointing this out. Yes, we need to be more careful about our definitions. By saying "direct effect", we refer to the direct effect of the SNP on outcome Y not mediated through covariates in $\boldsymbol{H}$, instead of the causal effect of the SNP on Y; we agree that in general the direct effect is only an association parameter, not a causal parameter, as shown in our first example. When estimating this direct effect, covariates in $\boldsymbol{H}$ may be in the middle of the causal pathway from SNPs to Y, as Figure 2 illustrated. Figure 2 just gives a simple scenario of the causal relationships among the variables of interest, where we show one SNP, and we aim to explain the collider bias problem of the single SNP instead of the complete picture of all associated SNPs in the same block. In this case, the effect estimates may suffer from collider bias due to both the hidden confounder U and SNP are causal on covariates $\boldsymbol{H}$. We have expanded some discussions related to Figure 2 and hopefully it is clearer now.

2. *"Another problem for the DAG in Figure 2 is that covariates such as population ancestry (maybe denoted as $\boldsymbol{V}$ in the paper?) that are confounders of U and G are not included. In the structural equations (1) the authors simplify that $\boldsymbol{V}$ is not correlated with U nor G. If $\boldsymbol{V}$ are population ancestries, this assumption will not be true and I'm not sure whether it will affect the subsequent calculations or not."*

Reponse: In this paper we have followed the previous framework and assumed SNPs and confounders U were independent in the populations[3], and hence the confounders of U and G, such as population ancestry, are not considered. We have commented on this in the second paragraph in section 2.1. If G and U are associated, more bias will be introduced, and its handling is beyond the current paper (and our approach). Because one might use other/different covariates in the GWAS of variables in $\boldsymbol{H}$, we used $\boldsymbol{V}$ to represent these covariates. Our derivation showed that the conclusion in equation (5) and (6) holds only if the covariates in $\boldsymbol{V}$ are not associated with SNPs and counder U. This also implies that the GWAS summary data of covariates $\boldsymbol{H}$ are not biased. If $\boldsymbol{V}$ is associated with G or U, our analytical expression of the bias term does not hold, and our bias-correction method is not applicable.

3. *"For the MVMR-cML method the authors claim that in equation (9), the number of invalid IVs K can be as large as $l - p_2 - 1$. I'm not sure if this is possible. I think the slope $\boldsymbol{b}$ in (5) would not be identifiable if β_{G_iY} is not sparse enough. I hope the authors can clarify more."*

Response: Thanks for pointing this out. Actually $\boldsymbol{b}$ is identifiable for MVMR-cML if and only if the multivariable plurality condition is satisfied as discussed/proven in a previous paper[6].

4. *"Since the authors need to work with individual GWAS data anyway, what is the benefit of using summary GWAS MR?"*

Response: Although in the real GWAS data example we used individual level data (because we had access to individual level data), our proposed method is applicable when only GWAS summary data of outcome Y and covariates $\boldsymbol{H}$ are available, as shown in simulations, where simulated individuals-level data were first converted to GWAS summary data before all methods were applied.

5. *"Related to the previous question, if the trait is binary, than a linear model might not be appropriate, is the method proposed in the paper still applicable?"*

Response: Thanks for the question. In this paper we have only considered linear

models for quantitative traits; our method cannot be applied to binary traits (with logistic regression); it remains open how to extend the methods to binary traits. We have acknowledged this limitation of our method in the Discussion section.

6. *"To make the paper more convincing, I think the authors need to have some real data example to illustrate that univariate MR is not enough in practice."*

Response: Yes, as you suggested, we have added a new simulation study and a new analysis for the second real data example to demonstrate the inadequacy of univariate MR (UVMR). The new results are in section 5 in supplementary. When implementing UVMR, the elements in $\boldsymbol{b}$ were estimated separately. In the simulation, the collider bias was introduced by two covariates H_1 and H_2, and each IV Z affected both covariates. In UVMR where only one covariate was considered, there was a pleiotropy path $Z \to H_1 \to Y$ or $Z \to H_2 \to Y$. Although all IVs were valid for MVMR, none were valid for UVMR. UVMR produced biased point estimates of $\boldsymbol{b}$ and were not as effective as MVMR in reducing collider bias. In the real data example, we used the GWAS of BMI adjusted for two metabolomic principle components (PCs), as mentioned in the paper. In this example, some IVs were associated with both two PCs at a p-value $5e-10$ and might not be valid for UVMR. While MVMR gave similar results after bias correction, the results of different UVMR methods were not consistent. Given that MVMR have an advantage in accounting for pleiotropic paths through covariates, we believe the results of MVMR were more trustworthy.

"A minor comment: Section 2.1 the authors briefly mentioned mtCOJO. I think that part is very hard to follow without reading the mtCOJO paper and the authors should make the section more self-contained."

Response: Yes, we have added more explanations on the implementation of mtCOJO[8] in the second paragraph in section 2.1. We have also updated Figure 1 to accommodate our explanation.

References

[1] C. Barry, J. Liu, R. Richmond, M. K. Rutter, D. A. Lawlor, F. Dudbridge, and J. Bowden. Exploiting collider bias to apply two-sample summary data mendelian randomization methods to one-sample individual level data. *PLoS Genetics*, 17(8): e1009703, 2021.

[2] S. Cai, A. Hartley, O. Mahmoud, K. Tilling, and F. Dudbridge. Adjusting for

collider bias in genetic association studies using instrumental variable methods. *Genet. Epidemiol.*, 46(5):1–14, 2022.

[3] F. Dudbridge, R. J. Allen, N. A. Sheehan, A. F. Schmidt, J. C. Lee, R. G. Jenkins, L. V. Wain, A. D. Hingorani, and R. S. Patel. Adjustment for index event bias in genome-wide association studies of subsequent events. *Nat. Commun.*, 10(1): 1561, Apr 2019. ISSN 2041-1723. doi: 10.1038/s41467-019-09381-w. URL `https://doi.org/10.1038/s41467-019-09381-w`.

[4] I. M. Heid, A. U. Jackson, J. C. Randall, T. W. Winkler, L. Qi, V. Steinthorsdottir, G. Thorleifsson, M. C. Zillikens, E. K. Speliotes, R. Mägi, T. Workalemahu, C. C. White, N. Bouatia-Naji, and et al. Meta-analysis identifies 13 new loci associated with waist-hip ratio and reveals sexual dimorphism in the genetic basis of fat distribution. *Nat. Genet.*, 42(11):949–960, Nov. 2010.

[5] Z. Lin, I. Pan, and W. Pan. A practical problem with egger regression in mendelian randomization. *PLoS Genet.*, 18(5):e1010166, 2022.

[6] Z. Lin, H. Xue, and W. Pan. Robust multivariable mendelian randomization based on constrained maximum likelihood. *Am. J. Hum. Genet.*, 110(4):592–605, 2023. ISSN 0002-9297. doi: https://doi.org/10.1016/j.ajhg.2023.02.014. URL `https://www.sciencedirect.com/science/article/pii/S0002929723000848`.

[7] E. Sanderson, W. Spiller, and J. Bowden. Testing and correcting for weak and pleiotropic instruments in two-sample multivariable mendelian randomization. *Stat. Med.*, 40(25):5434–5452, Nov. 2021.

[8] Z. Zhu, Z. Zheng, F. Zhang, Y. Wu, M. Trzaskowski, R. Maier, M. R. Robinson, J. J. McGrath, P. M. Visscher, N. R. Wray, and J. Yang. Causal associations between risk factors and common diseases inferred from gwas summary data. *Nature Communications*, 9(1):224, Jan 2018. ISSN 2041-1723. doi: 10.1038/s41467-017-02317-2. URL `https://doi.org/10.1038/s41467-017-02317-2`.
