# Supplementary material for: Collider bias correction for multiple covariates in GWAS using robust multivariable Mendelian randomization
Source: PLoS Genet. 2024 Apr 22;20(4):e1011246. doi: 10.1371/journal.pgen.1011246 (PMC11065275; doi:10.1371/journal.pgen.1011246)
Supplement: Attachment — Submitted filename: author_one_to_one_response.pdf [file pgen.1011246.s003.pdf]

Authors' one-to-one responses

We appreciate the positive and helpful comments from the three reviewers. For the remaining issues raised, we have made every effort to carefully address them as detailed below. The modified parts of second revision are highlighted in blue in the main text.

1 Reviewer 1

1. *"The authors hava addressed all my comments."*

Response: Thank you!

2 Reviewer 2

1. *"The authors responded to my comments, and I now understand the paper better. Thank you. There are a number of typos in the new text, and some remaining in the old. The most serious of which is 'casually' instead of 'causally'".*

Response: Thank you for your careful reading and pointing this out! We have tried our best to correct all the typos in the paper and supplementary.

3 Reviewer 3

1. *" Foundation of the casual framework setup. I appreciate authors' effort in providing better description of the setup, but frankly speaking, it is still very confusing. The description now is a mixture of causal inference language and associations where the definitions are not coherent. If the direct effects that the authors care about are just conditional associations of a single SNP G on Y conditional on X, which has a clear definition by its own, seems that the casual concepts like mediation and collider bias, and the DAG in Fig1 are not relevant. The authors have also created new terms like direct association, indirect associations and spurious associations, which I don't think are clearly defined concepts and can be confusing. While the authors change from causal effects to different types of associations in the text, they still call models (1)-(3) on a*

single SNP as the "true causal model for each SNP G_i". These descriptions seem to be conflicting with each other. I feel that it is possible that the authors describe the problem clearly by starting with a causal model considering all SNPs, discuss the collider bias the mediation in that model and then discuss how it affects the single-SNP associations. This might be beyond the scope of the paper. The authors may add the limitation of their framework in the discussion section."

Response: Thanks for the question! As you suggested, we have explicitly pointed out at the beginning of section 2.1 that we frist present the causal models, then at the end generalize the results to association analysis (as done in our real data examples). We hope that this arrangement will make it much easier to follow. In addition, we have also added a comment that our figures and terms followed the previous research of collider bias in GWAS[2;1].

2. *"For my previous point 3, I did not understand the authors responses. Does the authors mean that b is still identifiable when 'invalid IVs K can be as large as $l - p_2 - 1$"? How is this relavant to the 'multivariable plurality condition'?"*

Reponse: For MVMR-cML, $\boldsymbol{b}$ is identifiable only if the multivariable plurality condition is satisfied. The multivariable plurality condition requires at least one valid IV Z for each exposure (covariate), and the matrix of true effects of valid IVs $[\boldsymbol{\beta}_{Z_j \boldsymbol{H}}]_{j=1}^{l-K}$ has full column rank. Hence, when there are p_2 covariates, there should be at least $p_2 + 1$ valid IVs to guarantee the identifiability of $\boldsymbol{b}$. When the multivariable plurality condition is satisfied, the number of invalid IVs K can be up to $l - (p_2 + 1)$. The details about multivariable plurality condition can be found in the previous paper[3].

References

[1] S. Cai, A. Hartley, O. Mahmoud, K. Tilling, and F. Dudbridge. Adjusting for collider bias in genetic association studies using instrumental variable methods. *Genet. Epidemiol.*, 46(5):1–14, 2022.

[2] F. Dudbridge, R. J. Allen, N. A. Sheehan, A. F. Schmidt, J. C. Lee, R. G. Jenkins, L. V. Wain, A. D. Hingorani, and R. S. Patel. Adjustment for index event bias in genome-wide association studies of subsequent events. *Nat. Commun.*, 10(1): 1561, Apr 2019. ISSN 2041-1723. doi: 10.1038/s41467-019-09381-w. URL `https://doi.org/10.1038/s41467-019-09381-w`.

[3] Z. Lin, H. Xue, and W. Pan. Robust multivariable mendelian randomization based

on constrained maximum likelihood. *Am. J. Hum. Genet.*, 110(4):592–605, 2023. ISSN 0002-9297. doi: https://doi.org/10.1016/j.ajhg.2023.02.014. URL https://www.sciencedirect.com/science/article/pii/S0002929723000848.
